# Long non-coding RNA LINC01559 exerts oncogenic role via enhancing autophagy in lung adenocarcinoma

**DOI:** 10.1186/s12935-021-02338-4

**Published:** 2021-11-25

**Authors:** Zhuochen Zhao, Junhu Wan, Manman Guo, Zhengwu Yang, Zhuofang Li, Yangxia Wang, Liang Ming

**Affiliations:** Department of Clinical Laboratory, The First Affiliated Hospital of Zhengzhou University and the Key Clinical Laboratory of Henan Province, Henan, China

**Keywords:** Lung adenocarcinoma, Long non-coding RNA, Autophagy, LINC01559, In vitro validation

## Abstract

**Background:**

Long non-coding RNAs (lncRNAs) have been verified to play fatal role in regulating the progression of lung adenocarcinoma (LUAD). Although lncRNAs play important role in regulating the autophagy of tumor cells, the function and molecular mechanism of LINC01559 in regulating lung cancer development remain to be elucidated.

**Method and materials:**

In this study, we used bioinformatics to screen out autophagy-related lncRNAs from TCGA-LUAD repository. Then the least absolute shrinkage and selection operator (LASSO) regression was applied to establish the signature of autophagy-related lncRNAs so that clinical characteristics and survival in LUAD patients be evaluated. Finally, we selected the most significant differences lncRNA, LINC01559, to verify its function in regulating LUAD progression in vitro.

**Results:**

We found high expression of LINC01559 indicates lymph node metastasis and poor prognosis. Besides, LINC01559 promotes lung cancer cell proliferation and migration in vitro, by enhancing autophagy signal pathway via sponging hsa-miR-1343-3p.

**Conclusion:**

We revealed a novel prognostic model based on autophagy-related lncRNAs, and provide a new therapeutic target and for patients with lung adenocarcinoma named LINC01559.

**Supplementary Information:**

The online version contains supplementary material available at 10.1186/s12935-021-02338-4.

## Introduction

Lung cancer is the second leading cause of human malignancy, as well the most common of death of cancer [1, 2]. Because of inconspicuous symptom in early stage lung cancer, patients were often diagnosed at an advanced stage, which resulting in lung cancer with only 15% 5-year survival rate [3]. LUSC (lung squamous carcinoma) and lung adenocarcinoma (LUAD) are two major types of lung cancer. The incidence of LUAD has been increasing these years and has surpassed that of LUSC [4]. LncRNAs are a class of non-coding RNAs with their length more than 200 nucleotides, which are often localized in nuclear and function in cytoplasm [5]. LncRNAs participate in various pathways and biological progress via cis- or trans-regulation in cancers [6]. Some lncRNAs can act on neighboring genes as transcription factors or transcription enhancers, namely cis regulatory elements, so as to activate or enhance the transcription of neighboring genes [7, 8]. Seung et al. demonstrated that lncRNA PVT1 contains enhancer RNA elements sequence that activate transcription of adjacent oncogene MYC, which can be competitively suppressed by the PVT1 promoter [9]. LncRNAs can also recruit proteins or act as miRNA sponge. LncRNA LINC00857 can influence lung cancer cell proliferation and migration via regulating autophagy by recruiting RNA-binding protein YBX1 [10]. LncRNAs’ expression is often dysregulated in tumors, so it can be regard as a potential therapeutic and prognostic target [11].

Autophagy, usually referred to macrophage, is a series of cell self-eating activities in order to keep homeostasis [12, 13]. The complete autophagy flux is regulated by a series of core enzymes, driven by the metabolic stress of cells, initiated by the formation of autophagosome membrane, and finally ended in the lysosome [14]. Therefore, autophagy plays a critical role in hypermetabolic tumor cells [15, 16]. Keisuke et al. found that inhibition of autophagy in pancreatic adenocarcinoma cells can increase the level of MHC-1 on the tumor cells surface and promote the number of CD8 + T cells, thus inhibit tumor immune evasion [17]. However, it has also been reported that there is deletion of the autophagy related genes ATG3 and ATG7 in RAS-driven early stage tumor mouse models [18, 19]. Autophagy is closely related to lung cancer. Early reported shown that autophagy-deficient Ras-driven NSCLC mouse have reduced its tumor malignancy and growth capacity, and Atg7-deficient tumors have more active apoptosis and immune response level [20]. In addition, Gizem Karsli-Uzunbas et al. also found that acute autophagy ablation of lung tumor mouse models resulted in tumor necrosis, suggesting that autophagy inhibition may be a new idea for the treatment of lung cancer [21]. Although autophagy is a critical factor affecting the occurrence and development of tumors, but the role of autophagy in LUAD is still unclear.

LINC01559 is a novel long intergenic non-coding RNA whose molecular biological functions has only recently begun to be investigate. LINC01559 was reported up-regulated in a variety of tumors and can be as a proto-oncogene. It has been shown that LINC01559 plays a cancer-promoting role in gastric cancer and can promote the progression of pancreatic cancer via enhancing autophagy [22–25]. However, whether LINC01559 can regulate autophagy in LUAD remains unclear and needs further clarifying.

In this study, we analyzed the autophagy-related lncRNAs, screened out a series of lncRNAs by constructing a risk signature, and carried out LINC01559 into vitro experimental verification. Our study provides a new therapeutic target and prognostic method for patients with lung adenocarcinoma.

## Method and materials

### Repositories

Patients’ transcriptome data and clinical data were all downloaded in the Cancer Genome Atlas repository (https://portal.gdc.cancer.gov/). Data included a total of 535 tumor samples and 59 normal samples. All patients’ information was displayed in the Additional file 1: Table S1. 232 autophagy-related genes were selected from the Human Autophagy Database (http://www.autophagy.lu/). Names of these genes were listed in Additional file 2: Table S2.

### Construction and bioinformatic validation of signature

We obtained the lncRNA expression data from TCGA repository and analyzed the correlation between lncRNAs and autophagy-related genes, |cor|> 0.4 was regarded as the inclusion criteria. Then we screened the lncRNAs by difference analysis and univariate regression analysis and obtained the prognostic differential autophagy-related lncRNAs. Finally, LASSO was used to construct a risk signature and each patient’s riskscore was calculated. The calculation formula of risk score is as follows: $Risk score={\sum }_{j=1}^{n}Co\mathrm{e}f\,j*ij$. In order to verify the accuracy of the model, we divided all patients into high-risk and low-risk groups according to median riskscore, and plotted risk curves and survival curves to distinguish the prognosis under different risk modes as well. Then univariate and multivariate independent prognostic analyses were applied to compare the prognostic efficacy between the signature and clinical characteristics (age, gender, TNM). ROC curves were also plotted to show the sensitivity and specificity of the prognostic risk signature.

### Bioinformatics mechanism exploration

Gene set variation analysis (GSVA) and gene set enrichment analysis (GSEA) were performed to analyze the differences in pathway enrichment under different risk patterns. The eligibility criteria for GSEA are: |NES|> 1, NOM p value < 0.05, FDR < 0.25. GSEA runs for 1000 times.

### Cell culture and transfection

The human lung adenocarcinoma cell lines A549 and H1299, as well human normal bronchial epithelial cell line BEAS-2B were purchased from the American Type Culture Collection (ATCC). Cell lines A549 and H1299 were cultured in RPMI-1640 medium (Yuanpei Biotechnology Co., Ltd, Shanghai) with 10% fetal bovine serum (Sorfa life science, South America) and 1% penicillin – streptomycin (New cell & Molecular Biotech Co., Ltd, Suzhou). BEAS-2B cell was cultured in Opti-DMEM (Yuanpei Biotechnology Co., Ltd, Shanghai) with 10% fetal bovine serum and 1% penicillin – streptomycin. All cells were incubated at 37° C, 5% carbon dioxide.

Si-LINC01559#1, si-LINC01559#2, si-NC, miRNA NC and hsa-miR-1343-3p inhibitor were designed and synthesized in Ribobio technology and co-transfected into cells with Lipotransfectamine 2000 transfection reagent (Sangon Bioengineering Co., Ltd, Shanghai). Sequences of si-LINC01559#1 and si-LINC01559#2 were shown in Additional file 3: Table S3. The final concentration of siRNA and miRNA inhibitor transfection was 30 nM.

### RNA extraction and quantitative real-time PCR

Total RNA of cells was extracted by TRIzol reagent (Thermo Fisher, United States). The complementary DNA was reverse transcribed buy PrimeScript™ RT reagent Kit (Takara, Kyoto, Japan). Quantitative real-time PCR (qPCR) was applied to detect expression quantity by using TB green Premix Ex Taq™ II kit (Takara, Kyoto, Japan) on LightCycler480 system (Roche, Swiss). Actin was used as internal reference. Sequences of forward and reverse primer of actin, RAB11A, hsa-miR-1343-3p and LINC01559 were shown in Table 1. The relative expression was calculated by the method of 2^−ΔΔCT^. All experiments were repeated for 3 times.

**Table 1:** Sequences of primers used for qRT-PCR

Name	Sequence
Actin Forward	CCTGGCACCCAGCACAAT
Actin Reverse	GGGCCGGACTCGTCATAC
LINC01559 Forward	TCCCTCAGCCAAGTCCTTCCTTAC
LINC01559 Reverse	GTCCAGTTCATGCTCTGACAGTCC
RAB11A Forward	GATATGGGACACAGCAGGGCAAG
RAB11A Reverse	CCAATAAGGCACCTACAGCTCCAC
miR-1343-3p Forward	CTCCTGGGGCCCGCAC
miR-1343-3p Reverse	AGTGCAGGGTCCGAGGTATT
miR-1343-3p RT	GTCGTATCCAGTGCAGGGTCCGAGGTATTCGCACTGGATACGACGCGAGA

### Cell proliferation assays

2000 cells were inoculated in each well of 96-well plates for 5 replicate wells. Cell viability was measured by using cell counting kit-8 (CCK-8) reagent (Dojindo Laboratories, Kumamoto, Japan), and OD450 was measured at 0, 24, 48 and 72 h respectively. The cell proliferation ability was detected by EdU (Beyotime technology, Shanghai) assay according to manufacturer’s instructions. All experiments were repeated for 3 times.

### Wound healing assays

2 × 10^5^ cells were seeded into the 6-well plate and proliferated to over 90% fusion. Swept the wound by using aseptic 200 µL pipette tip in a straight line and washed with PBS for 3 times. Cells were cultured in serum-free medium for 24 h, and images at 0 and 24 h were recorded. Experiments were repeated for 3 times.

### Transwell assays

Transwell assays were performed as described before [26, 27]. Briefly, transfected cells were inoculated in transwell chambers with 200 µL serum-free medium suspension which containing 2 × 10^4^ cells. The bottom chamber was added with 600 µL complete culture medium without penicillin – streptomycin. The migratory cells at the bottom of the chamber were observed with inverted phase contrast microscope at 24 h and counted. A total of 5 fields were observed and photographed at 50 field magnification per chamber. The experiment was repeated three times.

### Flow cytometry assays

H1299 cells were collected after transfected with siRNA for 48 h. Resuspended the cells with 500 μL buffers and add 5 μL of Annexin-V FITC and propidium iodide (PI) respectively. Incubated in dark for 15 min and then determined on flow cytometry. All experiments were repeated for 3 times.

### Western blot

The transfected cells were lysed with RIPA lysate for 30 min to extract the total protein. Then total protein was divided by electrophoresis in SDS-PAGE system for 120 V, 2 h. Polyvinylidene fluoride (PVDF) membrane as the carrier and protein transferred for 400 mA, 1 h. The following antibodies were used: LC3B (Proteintech: 14,600–1-AP, 1:1000), p62 (Abmart: T59081, 1:1000), tubulin (Abmart: M20005, 1:1000), Caspase 3 (Abmart: T40044, 1:1000), bcl-2 (Abmart: T40056, 1:1000), CDK1 (Abways: CY5176, 1:1000), cyclin B1 (Abways: CY5378, 1:1000), cyclin D1 (Abways: CY5404, 1:1000), RAB11A (Abways: CY5301, 1:1000).

### Immunofluorescence staining

The cells were evenly spread in the 6-well plate for cell slide. After treatment with paraformaldehyde and tritonX-100, cell was blocked with TBST (with 5% bovine serum albumin) for 1 h. Then cells were incubated overnight with LC3B primary antibody (Proteintech: 14,600–1-AP, dilution ratio 1:200). Cells were incubated in FITC-labeled secondary antibodies for 1 h and finally observed under fluorescence microscope.

### Statistics

All bioinformatics analyses were performed in R version 4.0.2, and all experimental data analysis was carried out in GraghPad Prism 9. The significance of the differences between the groups was assessed by the Student’s t test. Correlation coefficient between lncRNAs and autophagy-related genes expression were analyzed by spearman correlation analysis. Kaplan–Meier method was performed to plotting survival curves. Univariate and multivariate regression analysis were used to evaluating association between parameters and clinical characteristics. *p < 0.05, **p < 0.01, ***p < 0.001 and ****p < 0.0001 were considered significant; ns intended no significance.

## Results

### Screening of autophagy-related lncRNAs and construction of risk signature

**Fig. 1:**
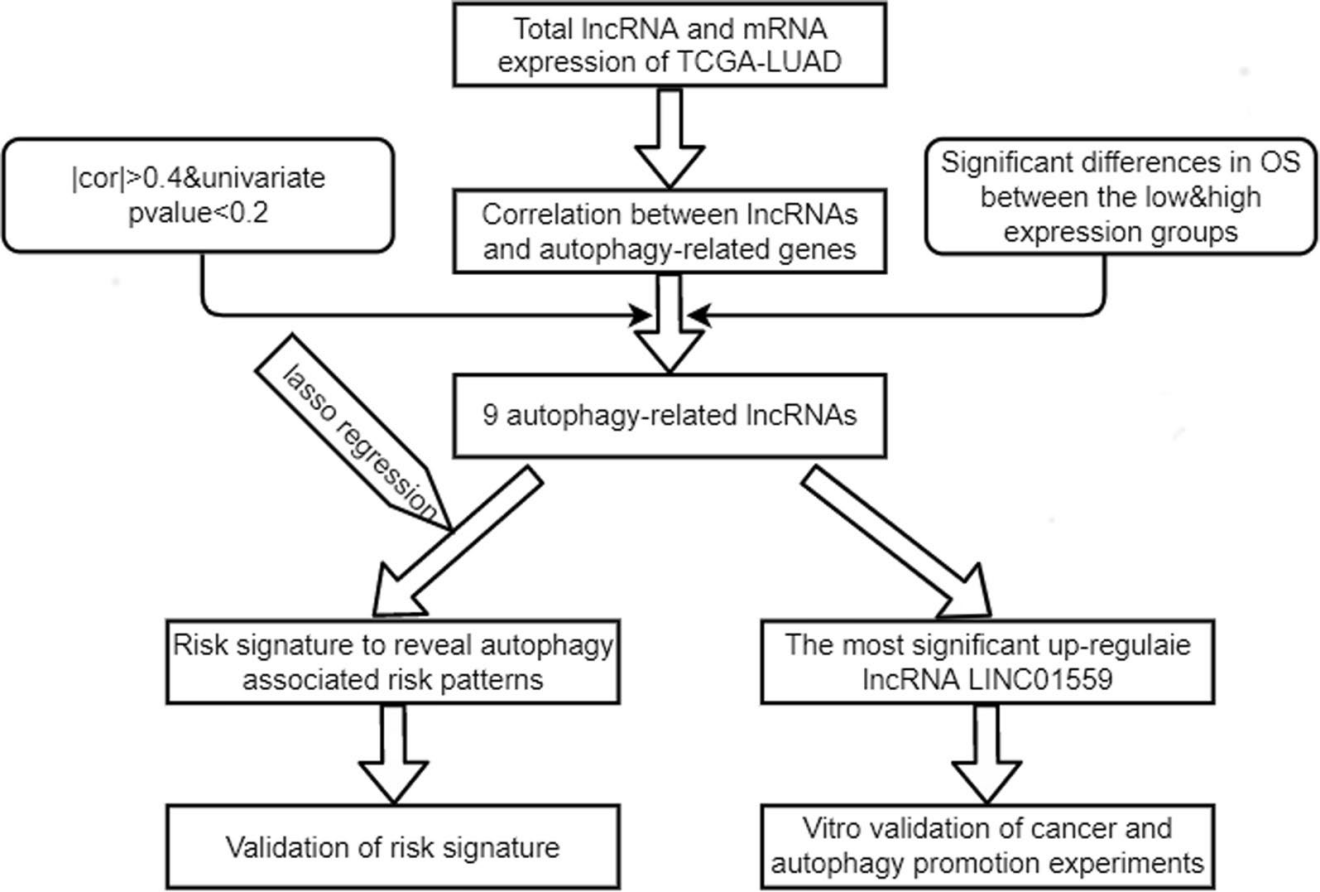
The work flow of whole study

**Fig. 2:**
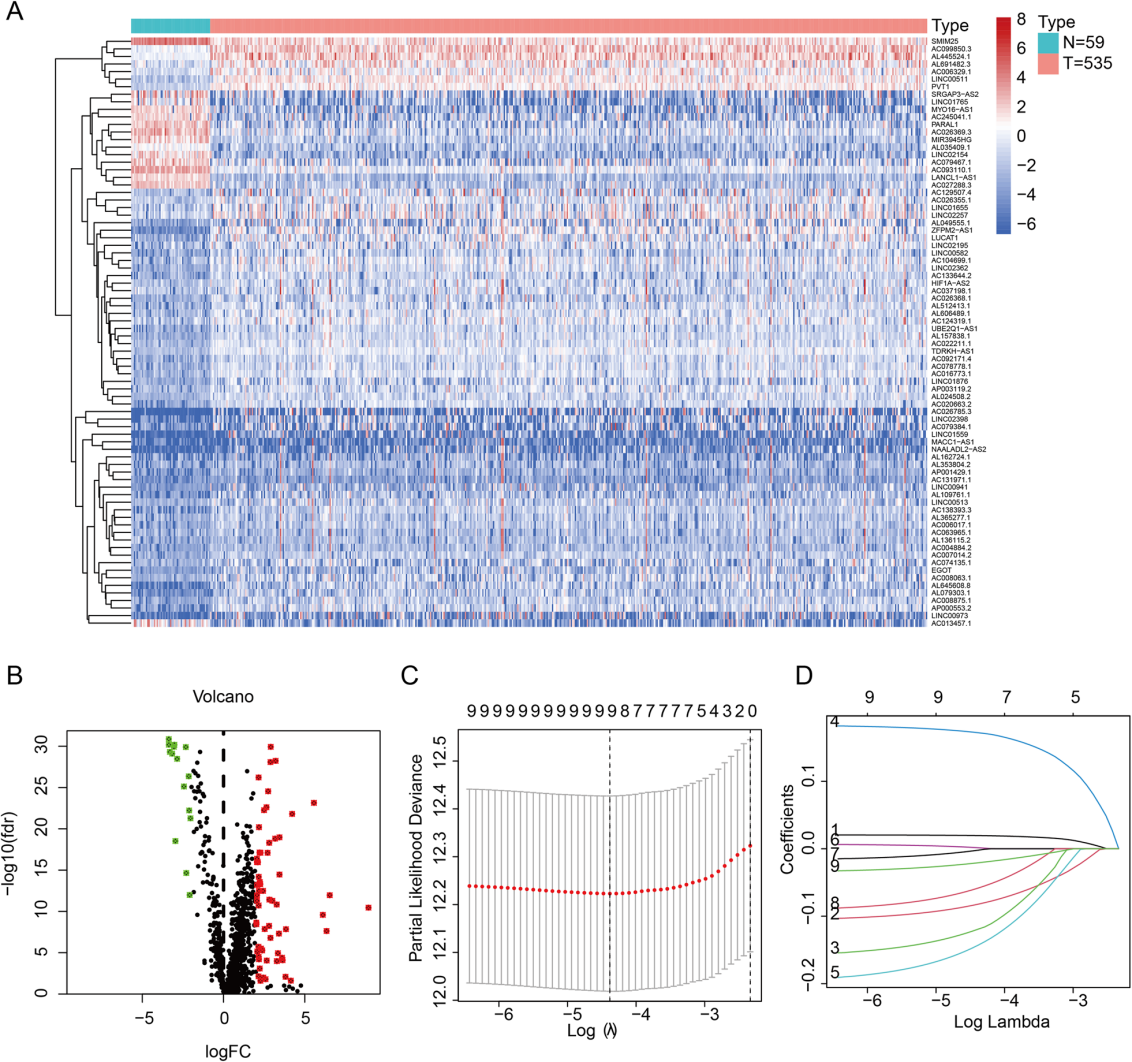
Difference analysis and construction of autophagy-related lncRNA risk signature. **A**, **B** Heatmap and volcano plot of differently expression autophagy-related lncRNAs. **C**, **D** LASSO analysis of 9 autophagy-related lncRNAs

The work flow was shown in Fig. 1. We downloaded 594 samples from TCGA repository, then obtained the lncRNA expression matrix and autophagy-related expression matrix of every individual. Then we calculated the correlation coefficient between lncRNAs and mRNAs by using spearman correlation analysis and omitted the relationship between the correlation coefficient less than 0.4. The correlation between lncRNAs and mRNAs were shown in Additional file 4: Table S4. We then obtained 78 lncRNAs (DElncRNAs) that abnormally expressed in tumors by differential analysis (Fig. 2A, B). Next, we performed univariate regression analysis to screen the 78 DElncRNAs, and 18 prognostic-associated lncRNAs were finally obtained by considering p value < 0.2 as the cutoff value. By comparing the relationship between these 18 DElncRNAs expression levels and survival time, we finally selected 9 lncRNAs with significant differences between high and low expression cohorts (Grouping based on the median expression respectively). The risk signature was constructed by lasso regression (Fig. 2C, D). The coefficient value of each lncRNA was listed in Table 2.

**Table 2:** Coefficient values of 9 autophagy-related lncRNAs

LncRNAs	Coefficient values
AC099850.3	0.0201191701846034
AC026355.1	− 0.09316896989732
LANCL1-AS1	− 0.137677249078995
AL606489.1	0.177587224643457
TDRKH-AS1	− 0.16853642168246
LINC01559	0.00429839508480869
AC027288.3	− 0.00874668960449896
AC026369.3	− 0.0743248258223173
SRGAP3-AS2	− 0.0267156775695313

### Risk patterns can reflect the prognostic of patients

**Fig. 3:**
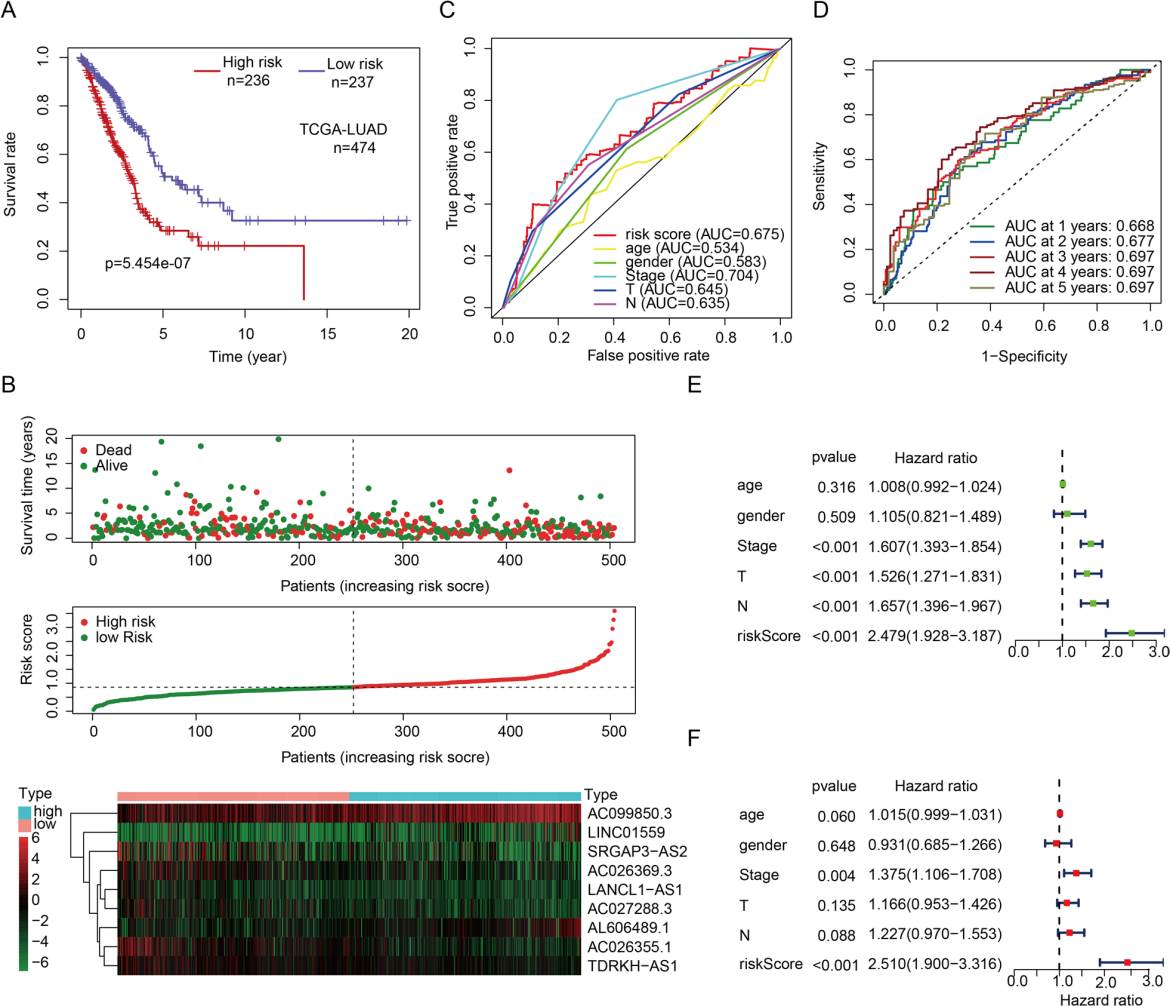
Crosstalk between risk patterns and LUAD patients’ prognosis. **A** Kaplan–Meier survival curve of patients in different risk patterns. **B** Risk plot and risk curve of LUAD patients in different risk group. **C**, **D** ROC curve of risk score in predicting prognostic of LUAD patients. **E**, **F** Univariate and multivariate prognostic analysis of T, N, age, gender, stage and risk score

According to median of all individuals’ risk score, we divided patients to low-risk (n = 236) and high-risk group (n = 237). Kaplan–Meier curve showed that there was a significantly difference between two risk modes (Fig. 3A). Combined with the trend of risk curve and risk plot, we found that patients in high-risk mode had lower survival rate (Fig. 3B). By comparing with the area under curve (AUC) of risk score, TNM, age and sex in ROC curves, we found that the risk signature had the same excellent sensitivity and accuracy in predicting prognostic (Fig. 3C, D). In univariate and multivariate independent prognostic analysis, the hazard ratio (HR) of risk score was 2.479 and 2.510 respectively (p < 0.001), which was much higher than other clinical characteristics (Fig. 3E, F). These results indicated that the signature of autophagy-related lncRNAs was a potential indicator of prognosis in patients with lung adenocarcinoma, and it has a significant effect in assessing the risk of patients.

### Risk modes can distinguish different clinicopathologic features

**Fig. 4:**
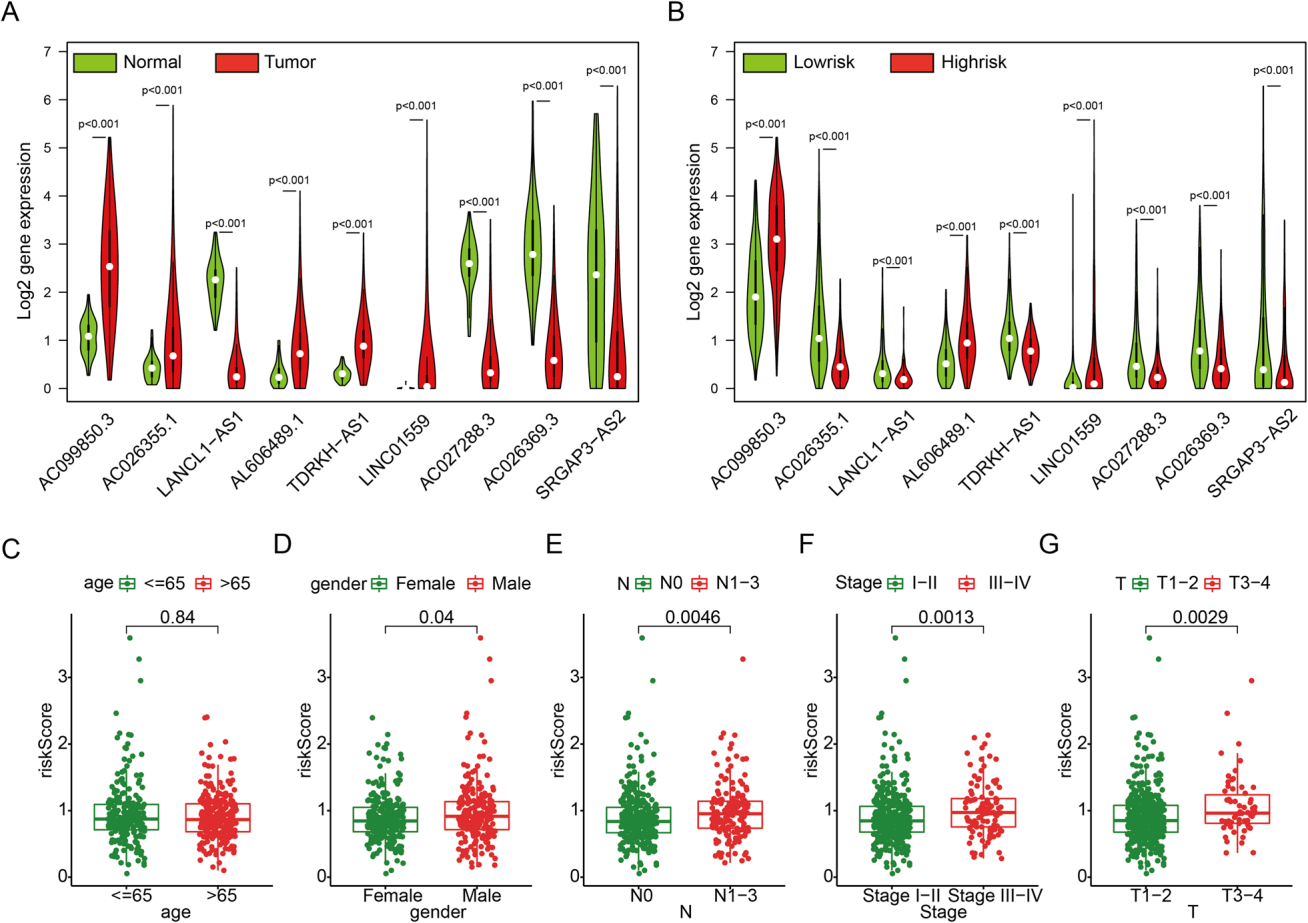
Risk pattern revealed difference clinicopathology. **A** Expression of each autophagy-related lncRNA between normal and tumor patients. **B** Expression of each autophagy-related lncRNA between low- and high-risk cohorts. **C**–**G** Association between riskscore and clinical characteristics (age, gender, T, N and stage)

The single-gene survival curves of 9 lncRNAs were plotted and displayed in Additional file 5: Fig. S1. The differential expression of 9 lncRNAs between tumor and normal tissues was shown in Fig. 4A. We then compared the expression of these lncRNAs under different risk modes. It was found that all 9 lncRNAs were all differentially expressed between the high- and low-risk groups (Fig. 4B). By comparing the risk score between the features of different clinicopathologic, we found that male, patients with positive lymph node metastasis, larger tumor lesions and advanced stage patients had a greater risk of prognosis. The risk score was independent of age (Fig. 4C−G).

Next, we briefly discussed the mechanism of autophagy-related lncRNA in affecting LUAD. GSEA was performed to finding pathways that enriched in high-risk group significantly. We found that the most significantly enriched functions were as follows: cell cycle, DNA replication, homologous recombination, mismatch repair, oocyte meiosis and ubiquitin mediated proteolysis. This result showed that the high-risk group may has a different cell cycle from the low-risk group, which also seen in GSVA results (Additional file 6: Fig. S2).

### High expression of LINC01559 indicates lymph node metastasis and poor prognosis

**Fig. 5:**
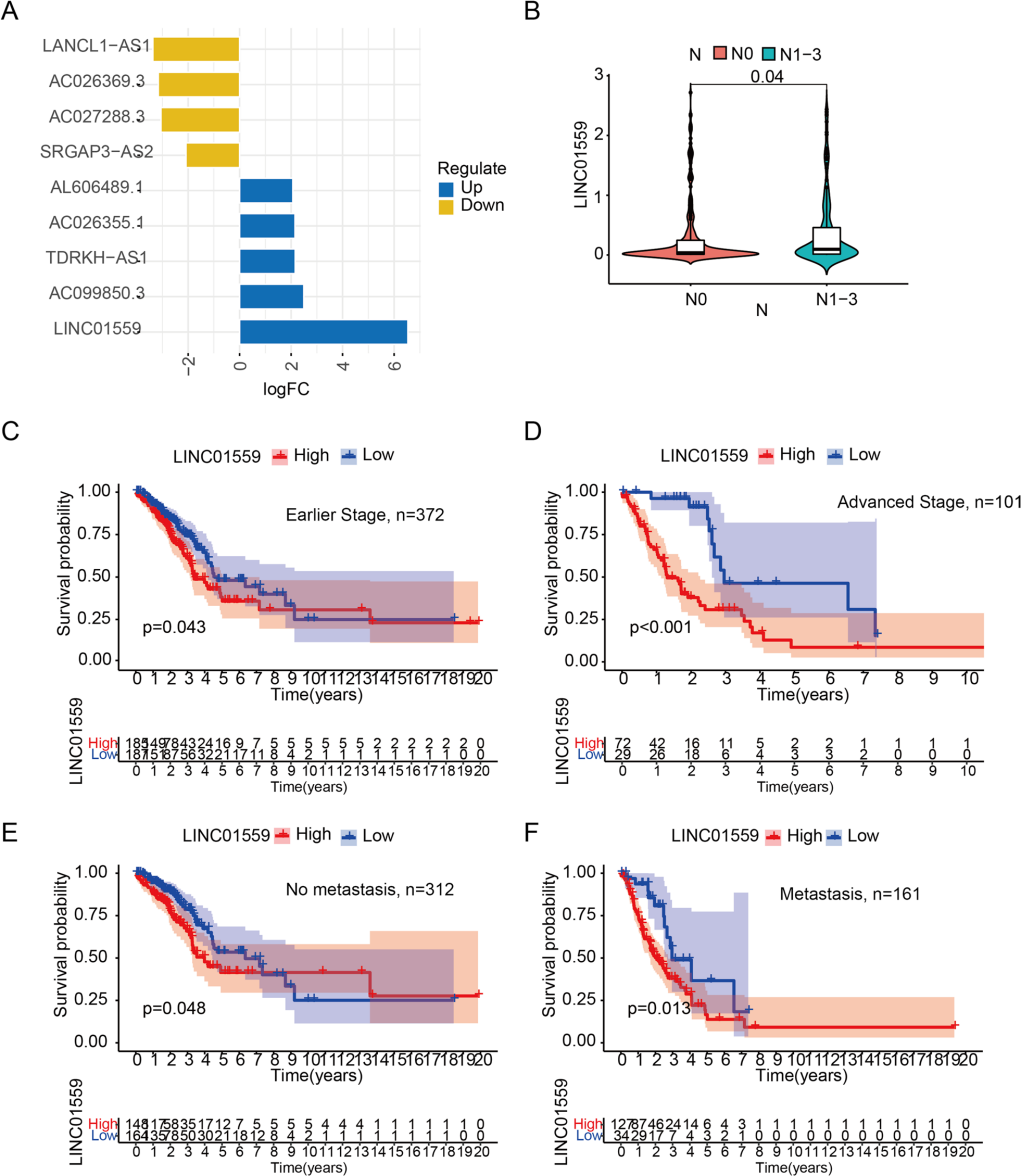
LINC01559 is an oncogene that related patients’ lymphatic metastasis and survival. **A** Log2 fold change of 9 autophagy-related lncRNAs. **B** Differential expression of LINC01559 between negative and positive lymphatic metastasis (N0 and N1-3). **C**, **D** Kaplan–Meier curves between low and high LINC01559 expression in early and advanced stage patients. **E**, **F** Kaplan–Meier curves between low and high LINC01559 expression in no metastasis and metastasis patients

We found that LINC01559 was the most differential expression lncRNA in 9 autophagy-related lncRNAs, so we then delved into the oncogene properties of LINC01559 (Fig. 5A). We found that the expression level of LINC01559 was significantly higher in patients with lymph node metastasis than without lymph node metastasis (Fig. 5B). That proved LINC01559 may promote the migration of LUAD cells. Patients with high expression of LINC01559, whether in an earlier (Stage I-II) or advanced stage (Stage III-IV), had poorer survival rate (Fig. 5C, D). Otherwise, regardless of whether lymph node metastasis occurs, the high expression of LINC01559 indicated a poor prognosis (Fig. 5E, F). These evidences proved that LINC01559 can promote lymph node metastasis of LUAD and affect the prognosis of patients.

### LINC01559 promotes LUAD cell proliferation and migration in vitro

**Fig. 6:**
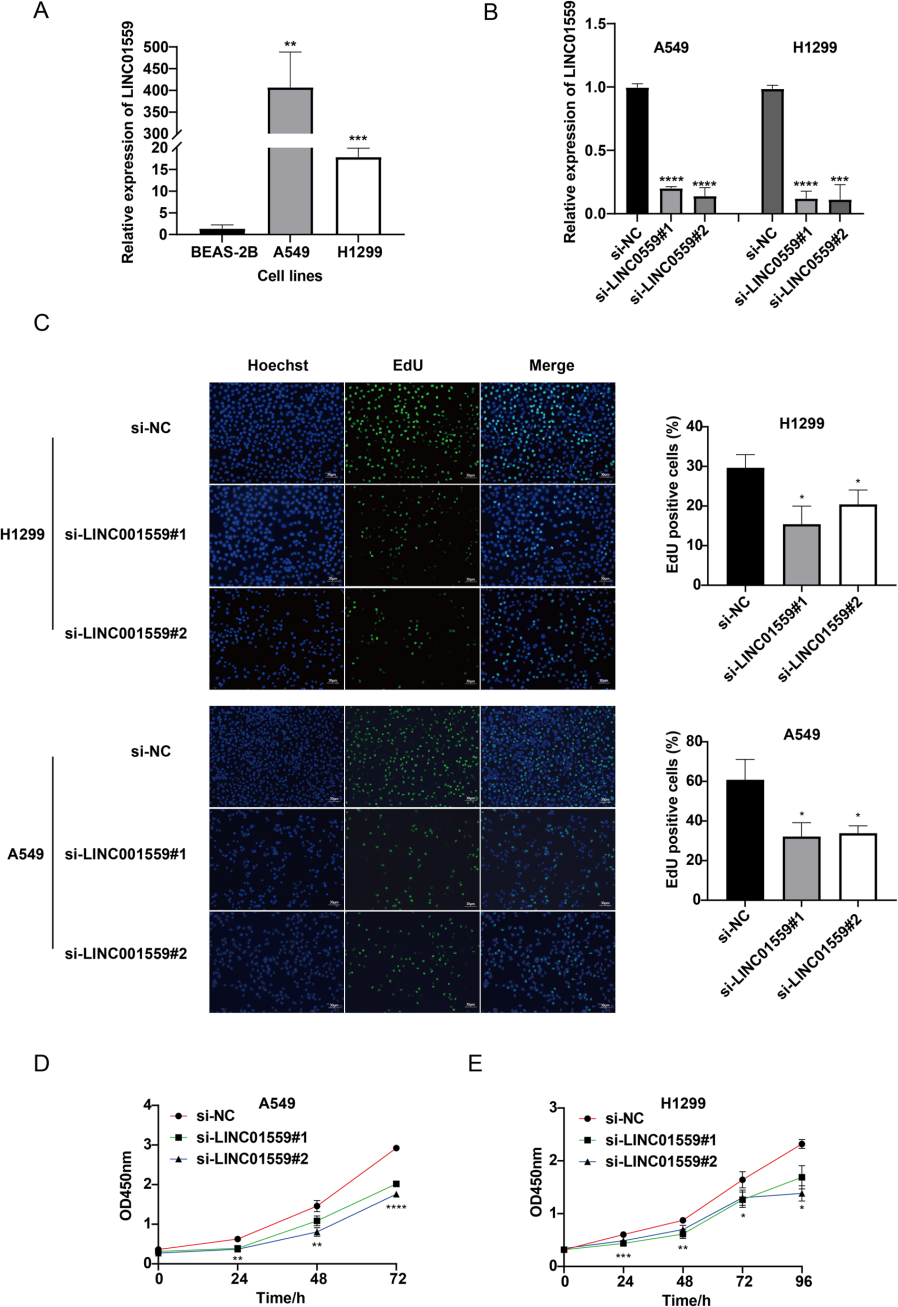
LINC01559 was up-regulated in LUAD cell and promoted proliferation of LUAD. **A** Expression of LINC01559 among BEAS-2B, A549 and H1299. **B** The efficiency of si-LINC01559#1 and si-LINC01559#2 in A549 and H1299 cell lines. **C** EdU assays to examine proliferation capacity. **D** Cell activity assessed by CCK-8 assay. *p < 0.05; **p < 0.01; ***p < 0.005; ****p < 0.001

By the method of qPCR, we found that LINC01559 over expressed in LUAD cells (A549 and H1299) in comparing with normal cell BEAS-2B (Fig. 6A). In order to explore the function of LINC01559, we transfected two lung adenocarcinoma cell lines with small interfering RNA. The silencing efficiency was detected by qPCR, and the expression of LINC01559 was significantly decreased in two cell lines, what’s more, both si#1 and si#2 can silence LINC01559 expr ession in cells (Fig. 6B). EdU assay showed that compared with the negative control group, the number of EdU positive cells in LINC01559 silenced group was significantly reduced, whether in A549 or H1299 cell line (Fig. 6C). CCK-8 experiments demonstrated that after LINC01559 silenced, OD450nm was remarkably reduced (Fig. 6D, E). These results indicated that lncRNA LINC01559 could enhance the proliferation ability of LUAD cells, and the proliferation of LUAD cells was significantly inhibited while LINC01559 was silenced.

**Fig. 7:**
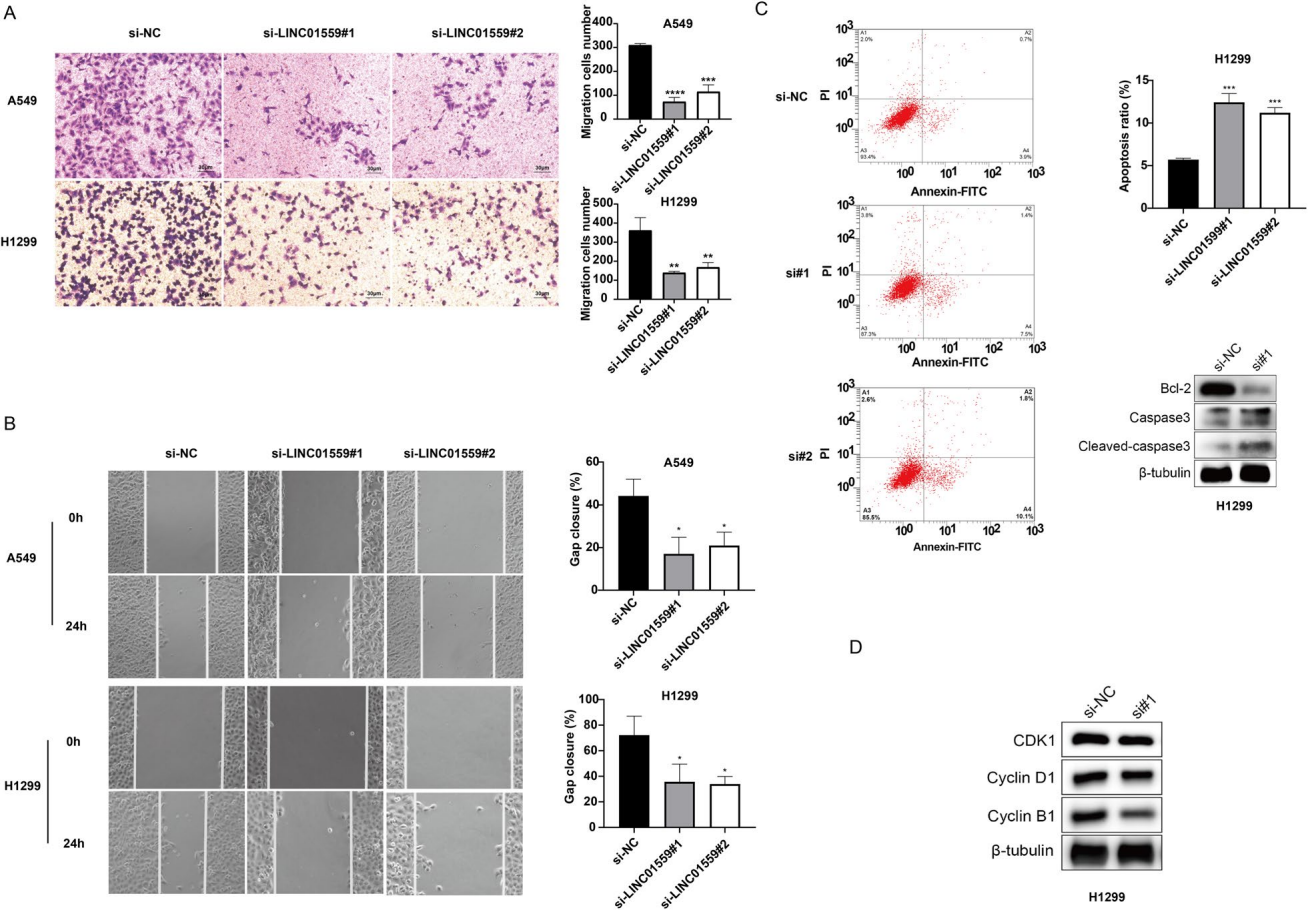
LINC01559 influenced migration, apoptosis and cell cycle in LUAD cell. **A** Transwell assay to assess migration cells in bottom chambers. **B** Wound healing assays to evaluate migration capacity. **C** Detection of apoptotic phenotype in si-NC and si-LINC01559#1 transfected cell. **D** Intracellular levels of apoptosis and cell cycle related proteins. *p < 0.05; **p < 0.01; ***p < 0.005; ****p < 0.001

We then investigated the role of LINC01559 in tumor migration. As results of transwell experiment showed, the capacity of passing through the chamber of LINC01559-silenced A549 and H1299 cells had reduced (Fig. 7A). Wound healing assay revealed that after LINC01559 silenced, the gap closure rate decreased significantly (Fig. 7B). These results suggested that silencing LINC01559 can inhibit tumor migration in vitro.

### LINC01559 affected cell cycle and diminished cell apoptosis of LUAD

The role of LINC01559 on the anti-apoptotic and cell cycle ability of tumor cells was analyzed by flow cytometry and western blot. As shown in Fig. 7C, after LINC01559 was knocked down, the number of early and late apoptotic cells increased significantly. Western blot for intracellular apoptotic protein showed that the knockdown of LINC01559 resulted in a decrease in the anti-apoptotic protein bcl-2 and a significant diminished in capase3 and cleaved-capase3 levels. And the content of cell cycle-related proteins (CDK1, cyclin B1 and cyclinD1) in LINC01559-silenced H1299 cells was also significantly reduced, indicating that knockdown of LINC01559 blocked the cycle of tumor cells (Fig. 7D). These results suggested that LINC01559 endowed tumor cells with the capacity of anti-apoptosis and had a positive regulation effect on cell cycle.

### LINC01559 exerts oncogenic role by enhancing autophagy of LUAD

**Fig. 8:**
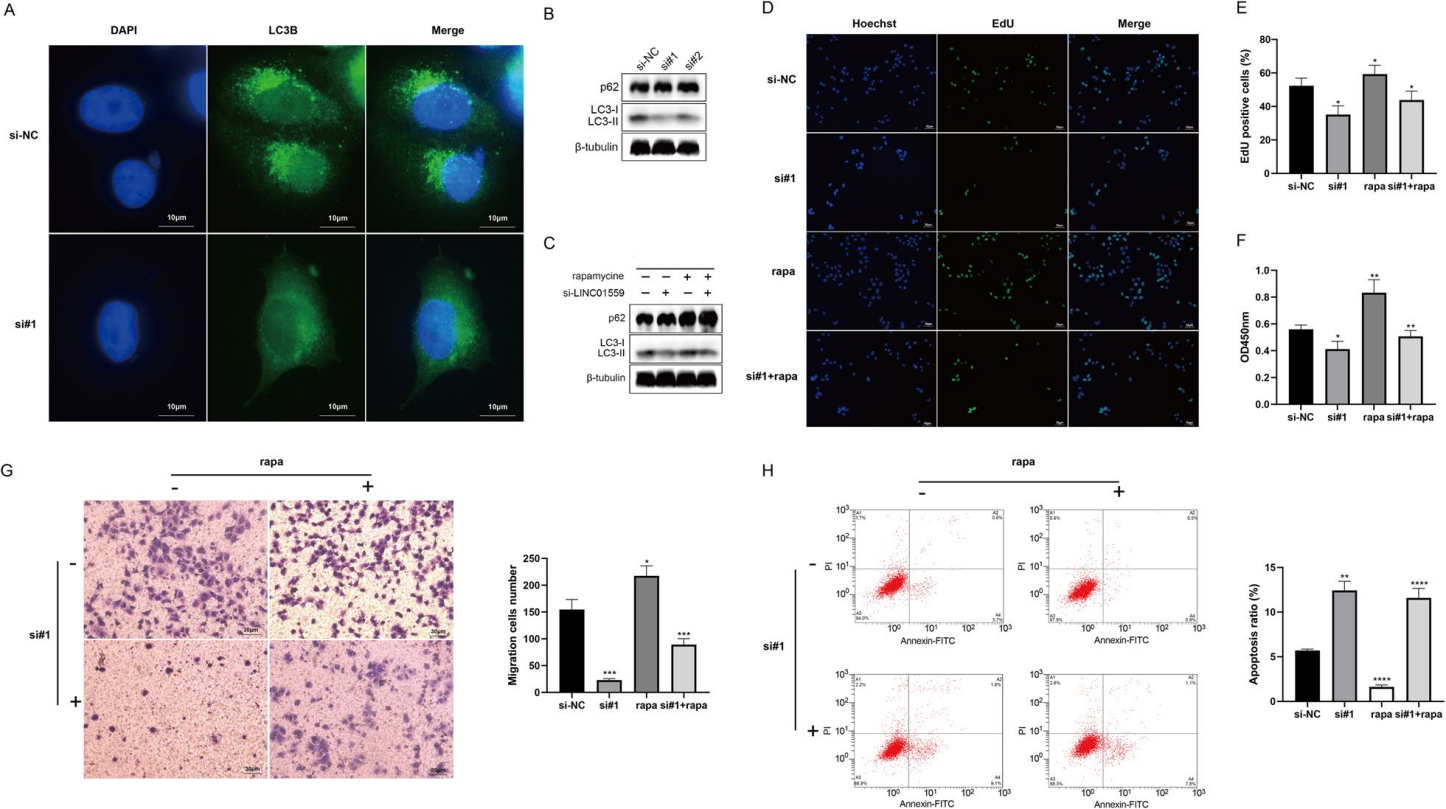
LINC01559 exerts oncogenic role by enhancing autophagy of LUAD. **A** Immunofluorescence staining of H1299 cells to detect LC3B plots. **B**, **C** Western blot detection of autophagy-related proteins (LC3B and p62). **D**, **E** Proliferation of H1299 cells in every group in rescue assay. **F**–**H** Cell activity evaluated, migration and apoptosis evaluated by CCK-8, transwell and low cytometry. *p < 0.05; **p < 0.01; ***p < 0.005; ****p < 0.001

Next, we systematically investigated the cross-talk between LINC01559, autophagy and oncogenesis. Immunofluorescence staining showed that the LINC01559 knocked out H1299 cells had less LC3B protein aggregation than the negative control group, which suggested a restraint level of autophagy flux (Fig. 8A). Western blot results showed that the level of LC3B-II protein in H1299 cells after LINC01559 knockout was significantly diminished, and LC3-II/I was decreased (Fig. 8B). In order to correlated autophagy with the cellular phenotypes previously observed, we conducted a series of rescue experiments. Cells were treated with 100 µM concentrations of rapamycin to increase their autophagy flux (Additional file 7: Fig. S3). We evaluated the levels of autophagy-related proteins (LC3B and p62) in the negative control group, the LINC01559-silenced group, the rapamycin-treated group and the rescue group (treated by RNAi as well rapamycin). The results showed that rapamycin could augment the levels of autophagy-related proteins in cells, indicating that rapamycin could be used as an autophagy inducer in H1299 cells (Fig. 8C). Finally, we investigated the phenotype of cells co-treated with rapamycin. EdU experiments demonstrated that the autophagy induced by rapamycin compensates for the proliferation inhibition after the silence of LINC01559 (Fig. 8D, E and Additional file 7: Fig. S3). Rapamycin also relieved the attenuated of cell activity (measured by 24 h CCK-8 OD value) caused by silencing LINC01559 (Fig. 8F). Transwell assay showed that migration capacity of LINC01599-silenced cells was enhanced after induced autophagy (Fig. 8G). Moreover, cells disposed with rapamycin possessed a stronger resistance to apoptosis than LINC01559-silenced cells (Fig. 8H). In conclusion, LINC01559 can enhance the autophagy of LUAD cells to exert its cancer-promoting properties.

### LINC01559 may regulate autophagy through the ceRNA axis

**Fig. 9:**
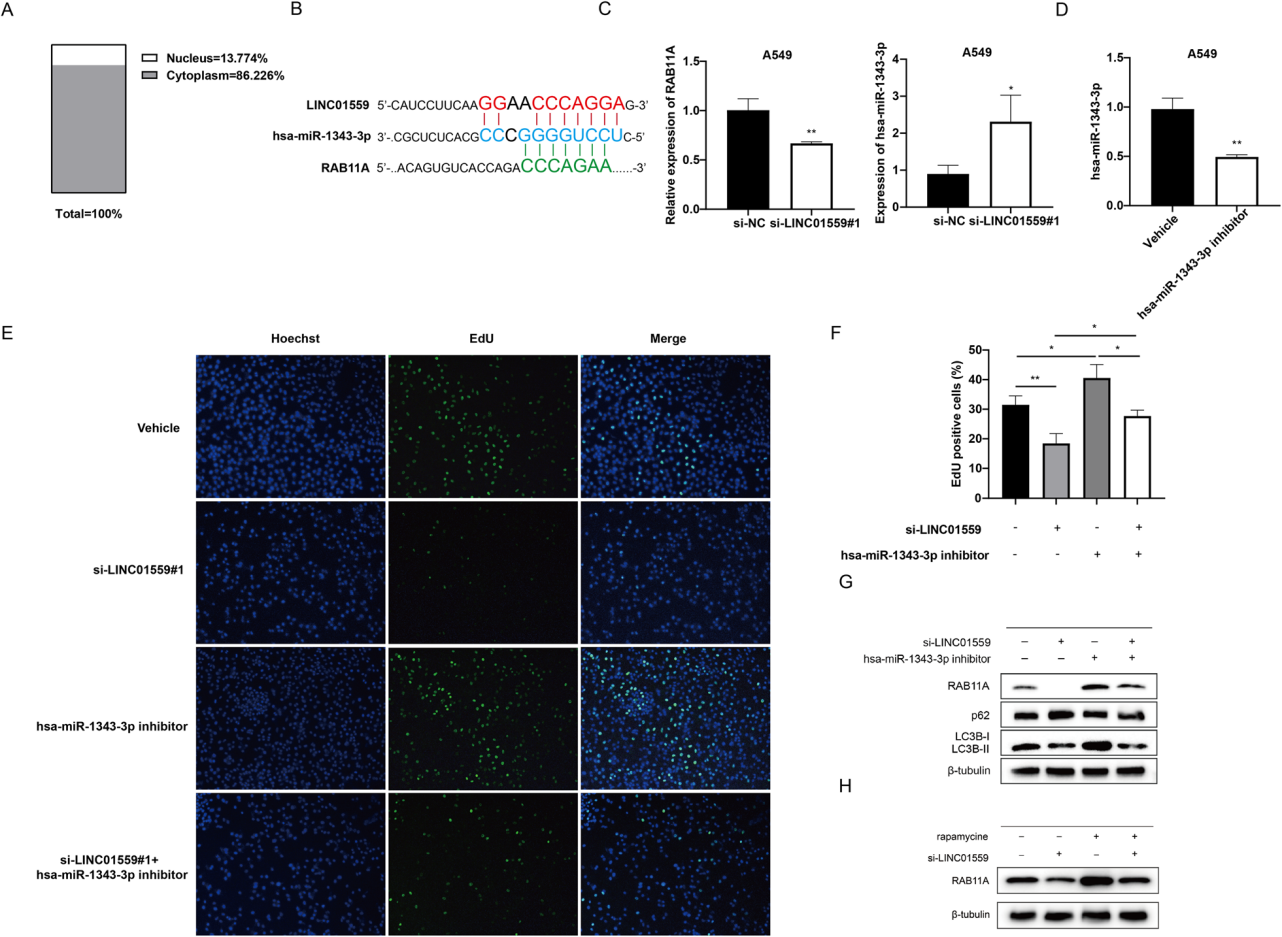
LINC01559 influenced autophagy gene RAB11A via ceRNA. **A** Subcellular localization of LINC01559. **B** Both LINC01559 and RAB11A had miRNA-response element of hsa-miR-1343-3p. **C** Correlation of LINC01559, hsa-miR-1343-3p and RAB11A. **D** Expression level of hsa-miR-1343-3p was downregulated after miRNA inhibitor transfection. **E**, **F** EdU assay to explore cell proliferation ability after si-LINC01559 and miRNA inhibitor co-transfected. **G** Relative protein expression of LC3B, p62 and RAB11A after si-LINC01559 and miRNA inhibitor co-transfected. **H** Variation of cell RAB11A protein level after treatment by si-LINC01559 and rapamycin

We then discussed how LINC01559 regulates autophagy in lung adenocarcinoma cells. We first predicted the subcellular localization of LIN01559 using the lncATLAS (http://lncatlas.crg.eu/) database. The results showed that most of LINC01559 were localized in the cytoplasm (Fig. 9A). Therefore, we hypothesized that LINC01559 might regulate autophagy through competitive adsorption of miRNAs [28]. The previous correlation analysis showed a strong positive correlation between LINC01559 and RAB11A, so we expect to find miRNAs that can competitively bind to LINC01559 and RAB11A. By searching starbase (http://starbase.sysu.edu.cn/) and TargetScan (http://targetscan.org/) databases, we found that both of them have miRNA binding domain of hsa-miR-1343-3p (Fig. 9B). The correlation analysis of TCGA-LUAD showed that LINC01559 was negatively correlated with has-miR-1343-3p (Additional file 8: Fig. S4). We detected the expression levels of RAB11A and has-miR-1343-3p relatively in A549 cell that knocked down LINC01559 and found that the expression of RAB11A was significantly reduced, while the expression of hsa-miR-1343-3p was significantly up-regulated (Fig. 9C). Then we design a group of rescue experiments to prove the relationship among LINC01559, hsa-miR-1343-3p and RAB11A. We transfected A549 cells by hsa-miR-1343-3p inhibitor, and qPCR results showed the expression level of has-miR-1343-3p was restrained compared with the vehicle group (Fig. 9D). Moreover, we co-transfected A549 cells with si-LINC01559 and miRNA inhibitor, and we found that proliferation inhibition caused by LINC01559-silence can be reversed via inhibiting hsa-miR-1343-3p (Fig. 9E, F). We evaluated the protein level of LC3B, p62 and RAB11A, as shown in Fig. 9F, has-miR-1343-3p could relieve the autophagy promotion of LINC01559 to LUAD. Meanwhile, the protein level of RAB11A was also reduced while silencing LINC01559, and was enhanced while hsa-miR-1343-3p were inhibited (Fig. 9G). Moreover, the RAB11A protein level went up when the autophagy of LUAD cells was activated by rapamycin (100 nM) (Fig. 9H). All the results implied that LINC01559 may regulate autophagy via ceRNA axis.

## Discussion

Autophagy is known as type II programmed cell death [29]. Autophagy flux can be divided into several steps: the occurrence of autophagy, the formation of autophagosome, the docking of autophagosome and lysosome, the degradation and reuse of autophagosome and its contents, involving in a conserve class of proteins [30]. These proteins are called autophagy related proteins (ATG). The formation of pre-autophagosomal structure (PAS) induced by ULK1/2-ATG13-FIP200 complex is the initiation of autophagy flux. PAS is subjected to ATG-mediated ubiquitin-like reaction, and modified by VPS34 complex to bind LC3 and phosphatidylethanolamine (PE) together, which called LC3-II. LC3-II is encapsulated on the autophagosome membrane and participates in the expansion of the autophagosome membrane [31, 32]. Autophagy is a conserved metabolic capacity of eukaryotic cell, as well an important component of complicated pathogenesis of cancers. Both normal somatic cells and tumor cells have a certain level of autophagy flux [33]. High metabolism tumor cells often rely on autophagy to provide the raw materials to maintain stemness or create a suitable survival tumor microenvironment [16, 34].

The study of autophagy pathway and autophagy regulation is brand new version for tumor detection and treatment. Many molecules and methods to inhibit tumor autophagy has put into clinical, these treatment act in any stage of autophagy flux, but blocking of autophagy pathway are all observed. Spautin-1 inhibits the degradation of beclin-1, a component of the VPS34 complex, by the deubiquitination peptidases USP10 and USP13. It has been found to promote tumor cell apoptosis by affecting autophagy-induced drug resistance in chronic myelogenous leukemia (CML) [35]. Chloroquine inhibits autophagy by blocking the fusion of autophagosome and lysosome, and can induce tumor ablation, which has been clinically demonstrated [36, 37]. S130 acts as an inhibitor of ATG4B, reacting on the early stage of autophagy via reducing splicing of LC3 by ATG4B in colorectal cancer [38, 39].

In this study, we explored the roles of long non-coding RNAs from the perspective of the regulation of autophagy by lncRNAs. LncRNAs has been proved to be a key factor in driving tumor development, and its effect on autophagy has also been reported [8, 40]. Endogenous competing RNAs are the most commonly reported trans-regulation of lncRNAs. For example, long non-coding RNA PVT1 is reported to be closely related to autophagy and has been proved to affect tumor autophagy via miR-365/ATG3, miR-20a-5p/ULK1, miR-619-5p/ATG14 or multiple axes [41–43]. LncRNA SNHG3 can act as a sponge of miR-485 to involving ATG7-related autophagy-induced apoptosis in neuronal cells [44]. Recruitment of RNA-binding proteins (RBP) is also an important method for lncRNA to affect autophagy. Tianliang et al. concluded that the interaction between lncRNA NBAT1 and protein PSMD10 could enhance the degradation of PSMD10, thus inhibiting its transcriptional facilitation of ATG7 in lung cancer cells [45]. In our study, we focused on the regulation of lncRNAs in lung adenocarcinoma autophagy, and screened 9 autophagy-related lncRNAs through bioinformatics analysis. Based on the expression of these lncRNAs, a risk signature was established to describe the risk pattern of patients from the perspective of autophagy. By integrating clinical information from the TCGA repository, we found that there were significant differences in survival rates among patients with different risk patterns (p = 5.454e-07), which could be served as an independent prognostic factor (95%CI: 1.9–3.316, p < 0.001). Patients with high risk were associated with positive lymph node metastases (p = 0.0046), larger primary lesions (p = 0.0029), and advancer grades (p = 0.0013). Next, we focused on the most significantly upregulated LINC01559 of 9 lncRNAs. There have been few reports of tumor promotion capacity of LINC01559. It can expedite the proliferation and migration of gastric cancer cells through IGF2BP3-involved ZEB1 mediated self-stabilization and PI3K/AKT signaling pathway [22, 25]. LINC01559 can also accelerate the progression of cancer through the YAP protein, or by promoting autophagy in pancreatic cancer cells [23, 46]. We found that patients with high expression of LINC01559 had poor survival rates. In vitro experiments demonstrated that the expression of LINC01559 was up-regulated in both lung adenocarcinoma cell lines A549 and H1299, and silenced LINC01559 can mitigate the proliferation, migration and anti-apoptotic ability of tumor cells in vitro. Subsequently, we found that knockdown of LINC01559 inhibited autophagy flux, and the rapamycin-induced rescue experiment proved that the oncogenesis of LINC01559 was caused by enhancing the autophagy flux in LUAD cells. Subsequent studies found that LINC01559 may affect RAB11A through ceRNA network and thus regulate autophagy in LUAD. RAB11A is a protein involved in regulation of autophagy pathway. Some evidence suggested that RAB11A was a component of the autophagosome membrane and participated in the binding process of autophagosome to lysosome [47, 48]. Our study demonstrated that RAB11A was over expression while autophagy was activated and it can be regulated by sponge role between LINC01559 and hsa-miR-1343-3p.

In general, we constructed a prognostic signature of autophagy-related lncRNAs and found that lncRNA LINC01559 leads to tumor formation by enhancing autophagy in vitro, which provided a new target for the diagnosis and treatment of lung adenocarcinoma.

## Supplementary Information

**Additional file 1**. Table S1.

**Additional file 2**. Table S2.

**Additional file 3**. Table S3.

**Additional file 4**. Table S4.

**Additional file 5**. Figure S1.

**Additional file 6**. Figure S2.

**Additional file 7**. Figure S3.

**Additional file 8**. Figure S4.

## Data Availability

All the data in this study are download from public databases as described in the passage. Data could be acquired from TCGA (https://portal.gdc.cancer.gov/) and HADb (http://www.autophagy.lu/) repositories.

## Abbreviations

LUAD: Lung adenocarcinoma
LUSC: Lung squamous carcinoma
ROC: Receiver operating curve
ATCC: American Type Culture Collection
CCK-8: Cell counting kit-8
PI: Propidium iodide
