# Supplementary material for: Long non-coding RNA LINC01559 exerts oncogenic role via enhancing autophagy in lung adenocarcinoma
Source: Cancer Cell Int. 2021 Nov 25;21:624. doi: 10.1186/s12935-021-02338-4 (PMC8614059; doi:10.1186/s12935-021-02338-4)
Supplement: Supplementary file 1 — Additional file 1. Table S1. [file 12935_2021_2338_MOESM1_ESM.docx]

Table S1. Information of 584 patients in TCGA-LUAD cohort.

| Patients ID | Sample Type | Histology Type |
| --- | --- | --- |
| TCGA-55-6980-11A-01R-1949-07 | Normal | Normal Tissue |
| TCGA-55-6982-11A-01R-1949-07 | Normal | Normal Tissue |
| TCGA-73-4676-11A-01R-1755-07 | Normal | Normal Tissue |
| TCGA-44-6778-11A-01R-1858-07 | Normal | Normal Tissue |
| TCGA-91-6847-11A-01R-1949-07 | Normal | Normal Tissue |
| TCGA-91-6831-11A-02R-1858-07 | Normal | Normal Tissue |
| TCGA-55-6979-11A-01R-1949-07 | Normal | Normal Tissue |
| TCGA-91-6849-11A-01R-1949-07 | Normal | Normal Tissue |
| TCGA-50-5936-11A-01R-1628-07 | Normal | Normal Tissue |
| TCGA-55-6986-11A-01R-1949-07 | Normal | Normal Tissue |
| TCGA-55-6985-11A-01R-1949-07 | Normal | Normal Tissue |
| TCGA-49-4490-11A-01R-1858-07 | Normal | Normal Tissue |
| TCGA-49-6743-11A-01R-1858-07 | Normal | Normal Tissue |
| TCGA-44-6145-11A-01R-1858-07 | Normal | Normal Tissue |
| TCGA-44-6144-11A-01R-1755-07 | Normal | Normal Tissue |
| TCGA-50-5939-11A-01R-1628-07 | Normal | Normal Tissue |
| TCGA-50-5935-11A-01R-1858-07 | Normal | Normal Tissue |
| TCGA-91-6829-11A-01R-1858-07 | Normal | Normal Tissue |
| TCGA-55-6978-11A-01R-1949-07 | Normal | Normal Tissue |
| TCGA-55-6981-11A-01R-1949-07 | Normal | Normal Tissue |
| TCGA-44-2661-11A-01R-1758-07 | Normal | Normal Tissue |
| TCGA-38-4627-11A-01R-1758-07 | Normal | Normal Tissue |
| TCGA-55-6971-11A-01R-1949-07 | Normal | Normal Tissue |
| TCGA-55-6975-11A-01R-1949-07 | Normal | Normal Tissue |
| TCGA-50-5932-11A-01R-1755-07 | Normal | Normal Tissue |
| TCGA-38-4626-11A-01R-1758-07 | Normal | Normal Tissue |
| TCGA-49-6761-11A-01R-1949-07 | Normal | Normal Tissue |
| TCGA-44-6776-11A-01R-1858-07 | Normal | Normal Tissue |
| TCGA-44-2662-11A-01R-1758-07 | Normal | Normal Tissue |
| TCGA-44-6777-11A-01R-1858-07 | Normal | Normal Tissue |
| TCGA-55-6970-11A-01R-1949-07 | Normal | Normal Tissue |
| TCGA-38-4632-11A-01R-1755-07 | Normal | Normal Tissue |
| TCGA-49-4512-11A-01R-1858-07 | Normal | Normal Tissue |
| TCGA-44-6148-11A-01R-1858-07 | Normal | Normal Tissue |
| TCGA-50-5930-11A-01R-1755-07 | Normal | Normal Tissue |
| TCGA-55-6969-11A-01R-1949-07 | Normal | Normal Tissue |
| TCGA-91-6836-11A-01R-1858-07 | Normal | Normal Tissue |
| TCGA-44-2657-11A-01R-1758-07 | Normal | Normal Tissue |
| TCGA-50-5931-11A-01R-1858-07 | Normal | Normal Tissue |
| TCGA-44-6146-11A-01R-1858-07 | Normal | Normal Tissue |
| TCGA-44-3396-11A-01R-1758-07 | Normal | Normal Tissue |
| TCGA-50-6595-11A-01R-1858-07 | Normal | Normal Tissue |
| TCGA-44-2655-11A-01R-1758-07 | Normal | Normal Tissue |
| TCGA-44-5645-11A-01R-1628-07 | Normal | Normal Tissue |
| TCGA-44-2668-11A-01R-1758-07 | Normal | Normal Tissue |
| TCGA-49-6745-11A-01R-1858-07 | Normal | Normal Tissue |
| TCGA-44-3398-11B-01R-1758-07 | Normal | Normal Tissue |
| TCGA-55-6968-11A-01R-1949-07 | Normal | Normal Tissue |
| TCGA-55-6984-11A-01R-1949-07 | Normal | Normal Tissue |
| TCGA-91-6835-11A-01R-1858-07 | Normal | Lung Adenocarcinoma |
| TCGA-55-6972-11A-01R-1949-07 | Normal | Lung Adenocarcinoma |
| TCGA-91-6828-11A-01R-1858-07 | Normal | Lung Adenocarcinoma |
| TCGA-50-5933-11A-01R-1755-07 | Normal | Lung Adenocarcinoma |
| TCGA-49-6742-11A-01R-1858-07 | Normal | Lung Adenocarcinoma |
| TCGA-49-6744-11A-01R-1858-07 | Normal | Lung Adenocarcinoma |
| TCGA-55-6983-11A-01R-1949-07 | Normal | Lung Adenocarcinoma |
| TCGA-38-4625-11A-01R-1758-07 | Normal | Lung Adenocarcinoma |
| TCGA-44-6147-11A-01R-1858-07 | Normal | Lung Adenocarcinoma |
| TCGA-44-2665-11A-01R-1758-07 | Normal | Lung Adenocarcinoma |
| TCGA-50-6590-01A-12R-1858-07 | Tumor | Lung Adenocarcinoma |
| TCGA-69-8255-01A-11R-2287-07 | Tumor | Lung Adenocarcinoma |
| TCGA-73-7498-01A-12R-2187-07 | Tumor | Lung Adenocarcinoma |
| TCGA-78-7148-01A-11R-2039-07 | Tumor | Lung Adenocarcinoma |
| TCGA-95-A4VN-01A-11R-A262-07 | Tumor | Lung Adenocarcinoma |
| TCGA-78-7152-01A-11R-2039-07 | Tumor | Lung Adenocarcinoma |
| TCGA-MP-A4TD-01A-32R-A262-07 | Tumor | Lung Adenocarcinoma |
| TCGA-55-7914-01A-11R-2170-07 | Tumor | Lung Adenocarcinoma |
| TCGA-44-A47A-01A-21R-A24H-07 | Tumor | Lung Adenocarcinoma |
| TCGA-97-A4M3-01A-11R-A24X-07 | Tumor | Lung Adenocarcinoma |
| TCGA-67-3773-01A-01R-0946-07 | Tumor | Lung Adenocarcinoma |
| TCGA-50-6593-01A-11R-1755-07 | Tumor | Lung Adenocarcinoma |
| TCGA-73-4659-01A-01R-1206-07 | Tumor | Lung Adenocarcinoma |
| TCGA-38-4630-01A-01R-1206-07 | Tumor | Lung Adenocarcinoma |
| TCGA-78-7540-01A-11R-2066-07 | Tumor | Lung Adenocarcinoma |
| TCGA-J2-A4AD-01A-11R-A24H-07 | Tumor | Lung Adenocarcinoma |
| TCGA-44-6778-01A-11R-1858-07 | Tumor | Lung Adenocarcinoma |
| TCGA-38-A44F-01A-11R-A24H-07 | Tumor | Lung Adenocarcinoma |
| TCGA-50-6592-01A-11R-1755-07 | Tumor | Lung Adenocarcinoma |
| TCGA-44-6775-01A-11R-A278-07 | Tumor | Lung Adenocarcinoma |
| TCGA-95-8494-01A-11R-2326-07 | Tumor | Lung Adenocarcinoma |
| TCGA-44-2656-01A-02R-0946-07 | Tumor | Lung Adenocarcinoma |
| TCGA-50-5055-01A-01R-1628-07 | Tumor | Lung Adenocarcinoma |
| TCGA-75-5147-01A-01R-1628-07 | Tumor | Lung Adenocarcinoma |
| TCGA-86-8278-01A-11R-2287-07 | Tumor | Lung Adenocarcinoma |
| TCGA-05-4250-01A-01R-1107-07 | Tumor | Lung Adenocarcinoma |
| TCGA-4B-A93V-01A-11R-A39D-07 | Tumor | Lung Adenocarcinoma |
| TCGA-86-7713-01A-11R-2066-07 | Tumor | Lung Adenocarcinoma |
| TCGA-91-A4BD-01A-11R-A24H-07 | Tumor | Lung Adenocarcinoma |
| TCGA-78-7149-01A-11R-2039-07 | Tumor | Lung Adenocarcinoma |
| TCGA-78-7535-01A-11R-2066-07 | Tumor | Lung Adenocarcinoma |
| TCGA-49-6742-01A-11R-1858-07 | Tumor | Lung Adenocarcinoma |
| TCGA-05-4249-01A-01R-1107-07 | Tumor | Lung Adenocarcinoma |
| TCGA-64-1679-01A-21R-2066-07 | Tumor | Lung Adenocarcinoma |
| TCGA-69-7760-01A-11R-2170-07 | Tumor | Lung Adenocarcinoma |
| TCGA-05-4405-01A-21R-1858-07 | Tumor | Lung Adenocarcinoma |
| TCGA-55-6987-01A-11R-1949-07 | Tumor | Lung Adenocarcinoma |
| TCGA-05-5428-01A-01R-1628-07 | Tumor | Lung Adenocarcinoma |
| TCGA-55-7724-01A-11R-2170-07 | Tumor | Lung Adenocarcinoma |
| TCGA-97-7938-01A-11R-2170-07 | Tumor | Lung Adenocarcinoma |
| TCGA-75-6206-01A-11R-1755-07 | Tumor | Lung Adenocarcinoma |
| TCGA-05-4418-01A-01R-1206-07 | Tumor | Lung Adenocarcinoma |
| TCGA-44-2661-01A-01R-1107-07 | Tumor | Lung Adenocarcinoma |
| TCGA-80-5608-01A-31R-1949-07 | Tumor | Lung Adenocarcinoma |
| TCGA-55-6984-01A-11R-1949-07 | Tumor | Lung Adenocarcinoma |
| TCGA-75-7031-01A-11R-1949-07 | Tumor | Lung Adenocarcinoma |
| TCGA-99-8032-01A-11R-2241-07 | Tumor | Lung Adenocarcinoma |
| TCGA-55-8512-01A-11R-2403-07 | Tumor | Lung Adenocarcinoma |
| TCGA-97-A4M2-01A-12R-A24X-07 | Tumor | Lung Adenocarcinoma |
| TCGA-50-6673-01A-11R-1949-07 | Tumor | Lung Adenocarcinoma |
| TCGA-67-3772-01A-01R-0946-07 | Tumor | Lung Adenocarcinoma |
| TCGA-86-7954-01A-11R-2187-07 | Tumor | Lung Adenocarcinoma |
| TCGA-44-6775-01C-02R-A277-07 | Tumor | Lung Adenocarcinoma |
| TCGA-L9-A743-01A-43R-A39D-07 | Tumor | Lung Adenocarcinoma |
| TCGA-05-4433-01A-22R-1858-07 | Tumor | Lung Adenocarcinoma |
| TCGA-91-A4BC-01A-11R-A24H-07 | Tumor | Lung Adenocarcinoma |
| TCGA-91-6829-01A-21R-1858-07 | Tumor | Lung Adenocarcinoma |
| TCGA-44-6779-01A-11R-1858-07 | Tumor | Lung Adenocarcinoma |
| TCGA-05-4432-01A-01R-1206-07 | Tumor | Lung Adenocarcinoma |
| TCGA-05-4398-01A-01R-1206-07 | Tumor | Lung Adenocarcinoma |
| TCGA-L4-A4E5-01A-11R-A24X-07 | Tumor | Lung Adenocarcinoma |
| TCGA-53-7624-01A-11R-2066-07 | Tumor | Lung Adenocarcinoma |
| TCGA-55-6968-01A-11R-1949-07 | Tumor | Lung Adenocarcinoma |
| TCGA-93-A4JP-01A-11R-A24X-07 | Tumor | Lung Adenocarcinoma |
| TCGA-44-2655-01A-01R-0946-07 | Tumor | Lung Adenocarcinoma |
| TCGA-55-1594-01A-01R-0946-07 | Tumor | Lung Adenocarcinoma |
| TCGA-50-5941-01A-11R-1755-07 | Tumor | Lung Adenocarcinoma |
| TCGA-49-6767-01A-11R-1858-07 | Tumor | Lung Adenocarcinoma |
| TCGA-75-5122-01A-01R-1755-07 | Tumor | Lung Adenocarcinoma |
| TCGA-78-7539-01A-11R-2066-07 | Tumor | Lung Adenocarcinoma |
| TCGA-55-7726-01A-11R-2170-07 | Tumor | Lung Adenocarcinoma |
| TCGA-78-7162-01A-21R-2066-07 | Tumor | Lung Adenocarcinoma |
| TCGA-55-6979-01A-11R-1949-07 | Tumor | Lung Adenocarcinoma |
| TCGA-86-8669-01A-11R-2403-07 | Tumor | Lung Adenocarcinoma |
| TCGA-44-3918-01A-01R-1107-07 | Tumor | Lung Adenocarcinoma |
| TCGA-75-6212-01A-11R-1755-07 | Tumor | Lung Adenocarcinoma |
| TCGA-44-5645-01B-04R-A277-07 | Tumor | Lung Adenocarcinoma |
| TCGA-44-3396-01A-01R-1206-07 | Tumor | Lung Adenocarcinoma |
| TCGA-50-8457-01A-11R-2326-07 | Tumor | Lung Adenocarcinoma |
| TCGA-49-AARE-01A-11R-A41B-07 | Tumor | Lung Adenocarcinoma |
| TCGA-49-4487-01A-21R-1858-07 | Tumor | Lung Adenocarcinoma |
| TCGA-44-6774-01A-21R-1858-07 | Tumor | Lung Adenocarcinoma |
| TCGA-L9-A5IP-01A-21R-A39D-07 | Tumor | Lung Adenocarcinoma |
| TCGA-69-8253-01A-11R-2287-07 | Tumor | Lung Adenocarcinoma |
| TCGA-L9-A7SV-01A-11R-A39D-07 | Tumor | Lung Adenocarcinoma |
| TCGA-97-8172-01A-11R-2287-07 | Tumor | Lung Adenocarcinoma |
| TCGA-86-A4P7-01A-11R-A24X-07 | Tumor | Lung Adenocarcinoma |
| TCGA-55-7574-01A-11R-2039-07 | Tumor | Lung Adenocarcinoma |
| TCGA-NJ-A4YI-01A-11R-A262-07 | Tumor | Lung Adenocarcinoma |
| TCGA-44-2668-01B-02R-A277-07 | Tumor | Lung Adenocarcinoma |
| TCGA-55-8620-01A-11R-2403-07 | Tumor | Lung Adenocarcinoma |
| TCGA-55-7728-01A-11R-2187-07 | Tumor | Lung Adenocarcinoma |
| TCGA-62-A46V-01A-11R-A24H-07 | Tumor | Lung Adenocarcinoma |
| TCGA-55-A48Y-01A-11R-A24H-07 | Tumor | Lung Adenocarcinoma |
| TCGA-71-6725-01A-11R-1858-07 | Tumor | Lung Adenocarcinoma |
| TCGA-55-6642-01A-11R-1858-07 | Tumor | Lung Adenocarcinoma |
| TCGA-78-7159-01A-11R-2039-07 | Tumor | Lung Adenocarcinoma |
| TCGA-55-6971-01A-11R-1949-07 | Tumor | Lung Adenocarcinoma |
| TCGA-50-5933-01A-11R-1755-07 | Tumor | Lung Adenocarcinoma |
| TCGA-95-A4VK-01A-11R-A262-07 | Tumor | Lung Adenocarcinoma |
| TCGA-64-5779-01A-01R-1628-07 | Tumor | Lung Adenocarcinoma |
| TCGA-05-4410-01A-21R-1858-07 | Tumor | Lung Adenocarcinoma |
| TCGA-44-A47G-01A-21R-A24H-07 | Tumor | Lung Adenocarcinoma |
| TCGA-97-8179-01A-11R-2287-07 | Tumor | Lung Adenocarcinoma |
| TCGA-78-7155-01A-11R-2039-07 | Tumor | Lung Adenocarcinoma |
| TCGA-49-4505-01A-01R-1206-07 | Tumor | Lung Adenocarcinoma |
| TCGA-97-8552-01A-11R-2403-07 | Tumor | Lung Adenocarcinoma |
| TCGA-MP-A4TJ-01A-51R-A262-07 | Tumor | Lung Adenocarcinoma |
| TCGA-73-4670-01A-01R-1206-07 | Tumor | Lung Adenocarcinoma |
| TCGA-55-A493-01A-11R-A24H-07 | Tumor | Lung Adenocarcinoma |
| TCGA-69-7973-01A-11R-2187-07 | Tumor | Lung Adenocarcinoma |
| TCGA-50-5932-01A-11R-1755-07 | Tumor | Lung Adenocarcinoma |
| TCGA-05-4430-01A-02R-1206-07 | Tumor | Lung Adenocarcinoma |
| TCGA-49-4486-01A-01R-1206-07 | Tumor | Lung Adenocarcinoma |
| TCGA-78-8662-01A-11R-2403-07 | Tumor | Lung Adenocarcinoma |
| TCGA-95-8039-01A-11R-2241-07 | Tumor | Lung Adenocarcinoma |
| TCGA-MP-A4TA-01A-21R-A24X-07 | Tumor | Lung Adenocarcinoma |
| TCGA-55-7284-01B-11R-2241-07 | Tumor | Lung Adenocarcinoma |
| TCGA-69-7764-01A-11R-2170-07 | Tumor | Lung Adenocarcinoma |
| TCGA-44-A4SU-01A-11R-A24X-07 | Tumor | Lung Adenocarcinoma |
| TCGA-49-4506-01A-01R-1206-07 | Tumor | Lung Adenocarcinoma |
| TCGA-78-7161-01A-11R-2039-07 | Tumor | Lung Adenocarcinoma |
| TCGA-49-AAQV-01A-11R-A39D-07 | Tumor | Lung Adenocarcinoma |
| TCGA-62-A46P-01A-11R-A24H-07 | Tumor | Lung Adenocarcinoma |
| TCGA-49-6761-01A-31R-1949-07 | Tumor | Lung Adenocarcinoma |
| TCGA-44-3919-01A-02R-1107-07 | Tumor | Lung Adenocarcinoma |
| TCGA-69-7980-01A-11R-2187-07 | Tumor | Lung Adenocarcinoma |
| TCGA-93-7348-01A-21R-2039-07 | Tumor | Lung Adenocarcinoma |
| TCGA-75-7030-01A-11R-1949-07 | Tumor | Lung Adenocarcinoma |
| TCGA-86-8074-01A-11R-2241-07 | Tumor | Lung Adenocarcinoma |
| TCGA-MP-A4TE-01A-22R-A466-07 | Tumor | Lung Adenocarcinoma |
| TCGA-55-6543-01A-11R-1755-07 | Tumor | Lung Adenocarcinoma |
| TCGA-49-AAR3-01A-11R-A41B-07 | Tumor | Lung Adenocarcinoma |
| TCGA-38-4632-01A-01R-1755-07 | Tumor | Lung Adenocarcinoma |
| TCGA-62-A46S-01A-11R-A24H-07 | Tumor | Lung Adenocarcinoma |
| TCGA-99-8033-01A-11R-2241-07 | Tumor | Lung Adenocarcinoma |
| TCGA-55-6980-01A-11R-1949-07 | Tumor | Lung Adenocarcinoma |
| TCGA-05-4244-01A-01R-1107-07 | Tumor | Lung Adenocarcinoma |
| TCGA-91-8497-01A-11R-2403-07 | Tumor | Lung Adenocarcinoma |
| TCGA-64-1680-01A-02R-0946-07 | Tumor | Lung Adenocarcinoma |
| TCGA-86-7953-01A-11R-2187-07 | Tumor | Lung Adenocarcinoma |
| TCGA-55-8616-01A-11R-2403-07 | Tumor | Lung Adenocarcinoma |
| TCGA-86-8055-01A-11R-2241-07 | Tumor | Lung Adenocarcinoma |
| TCGA-73-4668-01A-01R-1206-07 | Tumor | Lung Adenocarcinoma |
| TCGA-55-6982-01A-11R-1949-07 | Tumor | Lung Adenocarcinoma |
| TCGA-67-6217-01A-11R-1755-07 | Tumor | Lung Adenocarcinoma |
| TCGA-44-6146-01A-11R-A278-07 | Tumor | Lung Adenocarcinoma |
| TCGA-49-4512-01A-21R-1858-07 | Tumor | Lung Adenocarcinoma |
| TCGA-64-5774-01A-01R-1628-07 | Tumor | Lung Adenocarcinoma |
| TCGA-97-8177-01A-11R-2287-07 | Tumor | Lung Adenocarcinoma |
| TCGA-78-8640-01A-11R-2403-07 | Tumor | Lung Adenocarcinoma |
| TCGA-95-7947-01A-11R-2187-07 | Tumor | Lung Adenocarcinoma |
| TCGA-44-6777-01A-11R-1858-07 | Tumor | Lung Adenocarcinoma |
| TCGA-55-7816-01A-11R-2170-07 | Tumor | Lung Adenocarcinoma |
| TCGA-49-AAR4-01A-12R-A41B-07 | Tumor | Lung Adenocarcinoma |
| TCGA-55-7907-01A-11R-2170-07 | Tumor | Lung Adenocarcinoma |
| TCGA-86-8359-01A-11R-2326-07 | Tumor | Lung Adenocarcinoma |
| TCGA-50-5051-01A-21R-1858-07 | Tumor | Lung Adenocarcinoma |
| TCGA-05-4402-01A-01R-1206-07 | Tumor | Lung Adenocarcinoma |
| TCGA-50-8459-01A-11R-2326-07 | Tumor | Lung Adenocarcinoma |
| TCGA-NJ-A7XG-01A-12R-A39D-07 | Tumor | Lung Adenocarcinoma |
| TCGA-49-4488-01A-01R-1755-07 | Tumor | Lung Adenocarcinoma |
| TCGA-64-5815-01A-01R-1628-07 | Tumor | Lung Adenocarcinoma |
| TCGA-86-8056-01A-11R-2241-07 | Tumor | Lung Adenocarcinoma |
| TCGA-49-6745-01A-11R-1858-07 | Tumor | Lung Adenocarcinoma |
| TCGA-38-4631-01A-01R-1755-07 | Tumor | Lung Adenocarcinoma |
| TCGA-38-4626-01A-01R-1206-07 | Tumor | Lung Adenocarcinoma |
| TCGA-44-6147-01B-06R-A277-07 | Tumor | Lung Adenocarcinoma |
| TCGA-55-7227-01A-11R-2039-07 | Tumor | Lung Adenocarcinoma |
| TCGA-55-A57B-01A-12R-A39D-07 | Tumor | Lung Adenocarcinoma |
| TCGA-69-7979-01A-11R-2187-07 | Tumor | Lung Adenocarcinoma |
| TCGA-44-A4SS-01A-11R-A24X-07 | Tumor | Lung Adenocarcinoma |
| TCGA-05-4424-01A-22R-1858-07 | Tumor | Lung Adenocarcinoma |
| TCGA-MN-A4N5-01A-11R-A24X-07 | Tumor | Lung Adenocarcinoma |
| TCGA-99-8028-01A-11R-2241-07 | Tumor | Lung Adenocarcinoma |
| TCGA-75-5146-01A-01R-1628-07 | Tumor | Lung Adenocarcinoma |
| TCGA-55-1596-01A-01R-0946-07 | Tumor | Lung Adenocarcinoma |
| TCGA-55-7910-01A-11R-2170-07 | Tumor | Lung Adenocarcinoma |
| TCGA-80-5611-01A-01R-1628-07 | Tumor | Lung Adenocarcinoma |
| TCGA-91-6828-01A-11R-1858-07 | Tumor | Lung Adenocarcinoma |
| TCGA-69-7761-01A-11R-2170-07 | Tumor | Lung Adenocarcinoma |
| TCGA-91-6849-01A-11R-1949-07 | Tumor | Lung Adenocarcinoma |
| TCGA-MP-A4TH-01A-31R-A262-07 | Tumor | Lung Adenocarcinoma |
| TCGA-44-7669-01A-21R-2066-07 | Tumor | Lung Adenocarcinoma |
| TCGA-44-2662-01A-01R-A278-07 | Tumor | Lung Adenocarcinoma |
| TCGA-73-7499-01A-11R-2187-07 | Tumor | Lung Adenocarcinoma |
| TCGA-55-8505-01A-11R-2403-07 | Tumor | Lung Adenocarcinoma |
| TCGA-86-8671-01A-11R-2403-07 | Tumor | Lung Adenocarcinoma |
| TCGA-95-7948-01A-11R-2187-07 | Tumor | Lung Adenocarcinoma |
| TCGA-95-7043-01A-11R-1949-07 | Tumor | Lung Adenocarcinoma |
| TCGA-50-5044-01A-21R-1858-07 | Tumor | Lung Adenocarcinoma |
| TCGA-J2-A4AG-01A-11R-A24H-07 | Tumor | Lung Adenocarcinoma |
| TCGA-86-7955-01A-11R-2187-07 | Tumor | Lung Adenocarcinoma |
| TCGA-78-7158-01A-11R-2039-07 | Tumor | Lung Adenocarcinoma |
| TCGA-05-5425-01A-02R-1628-07 | Tumor | Lung Adenocarcinoma |
| TCGA-91-6831-01A-11R-1858-07 | Tumor | Lung Adenocarcinoma |
| TCGA-97-8175-01A-11R-2287-07 | Tumor | Lung Adenocarcinoma |
| TCGA-86-8672-01A-21R-2403-07 | Tumor | Lung Adenocarcinoma |
| TCGA-44-5645-01A-01R-A278-07 | Tumor | Lung Adenocarcinoma |
| TCGA-50-6594-01A-11R-1755-07 | Tumor | Lung Adenocarcinoma |
| TCGA-86-7714-01A-12R-2170-07 | Tumor | Lung Adenocarcinoma |
| TCGA-86-6562-01A-11R-1755-07 | Tumor | Lung Adenocarcinoma |
| TCGA-55-8090-01A-11R-2241-07 | Tumor | Lung Adenocarcinoma |
| TCGA-55-7911-01A-11R-2170-07 | Tumor | Lung Adenocarcinoma |
| TCGA-55-8507-01A-11R-2403-07 | Tumor | Lung Adenocarcinoma |
| TCGA-93-8067-01A-11R-2287-07 | Tumor | Lung Adenocarcinoma |
| TCGA-44-3918-01A-01R-A278-07 | Tumor | Lung Adenocarcinoma |
| TCGA-97-7553-01A-21R-2039-07 | Tumor | Lung Adenocarcinoma |
| TCGA-55-8205-01A-11R-2241-07 | Tumor | Lung Adenocarcinoma |
| TCGA-05-4390-01A-02R-1755-07 | Tumor | Lung Adenocarcinoma |
| TCGA-86-8668-01A-11R-2403-07 | Tumor | Lung Adenocarcinoma |
| TCGA-62-8398-01A-11R-2326-07 | Tumor | Lung Adenocarcinoma |
| TCGA-55-8513-01A-11R-2403-07 | Tumor | Lung Adenocarcinoma |
| TCGA-83-5908-01A-21R-2287-07 | Tumor | Lung Adenocarcinoma |
| TCGA-05-4422-01A-01R-1206-07 | Tumor | Lung Adenocarcinoma |
| TCGA-44-2662-01A-01R-0946-07 | Tumor | Lung Adenocarcinoma |
| TCGA-50-5045-01A-01R-1628-07 | Tumor | Lung Adenocarcinoma |
| TCGA-44-6146-01B-04R-A277-07 | Tumor | Lung Adenocarcinoma |
| TCGA-44-4112-01B-06R-A277-07 | Tumor | Lung Adenocarcinoma |
| TCGA-91-6836-01A-21R-1858-07 | Tumor | Lung Adenocarcinoma |
| TCGA-55-6975-01A-11R-1949-07 | Tumor | Lung Adenocarcinoma |
| TCGA-55-A491-01A-11R-A24H-07 | Tumor | Lung Adenocarcinoma |
| TCGA-93-7347-01A-11R-2187-07 | Tumor | Lung Adenocarcinoma |
| TCGA-MP-A4T9-01A-11R-A24X-07 | Tumor | Lung Adenocarcinoma |
| TCGA-49-AAR9-01A-21R-A41B-07 | Tumor | Lung Adenocarcinoma |
| TCGA-75-6203-01A-11R-1755-07 | Tumor | Lung Adenocarcinoma |
| TCGA-55-8091-01A-11R-2241-07 | Tumor | Lung Adenocarcinoma |
| TCGA-97-7547-01A-11R-2039-07 | Tumor | Lung Adenocarcinoma |
| TCGA-05-4417-01A-22R-1858-07 | Tumor | Lung Adenocarcinoma |
| TCGA-62-A46R-01A-11R-A24H-07 | Tumor | Lung Adenocarcinoma |
| TCGA-49-4490-01A-21R-1858-07 | Tumor | Lung Adenocarcinoma |
| TCGA-05-4395-01A-01R-1206-07 | Tumor | Lung Adenocarcinoma |
| TCGA-62-A46Y-01A-11R-A24H-07 | Tumor | Lung Adenocarcinoma |
| TCGA-97-A4M7-01A-11R-A24X-07 | Tumor | Lung Adenocarcinoma |
| TCGA-78-8655-01A-11R-2403-07 | Tumor | Lung Adenocarcinoma |
| TCGA-MP-A4TF-01A-11R-A262-07 | Tumor | Lung Adenocarcinoma |
| TCGA-55-8203-01A-11R-2241-07 | Tumor | Lung Adenocarcinoma |
| TCGA-91-6848-01A-11R-1949-07 | Tumor | Lung Adenocarcinoma |
| TCGA-86-8073-01A-11R-2241-07 | Tumor | Lung Adenocarcinoma |
| TCGA-78-7220-01A-11R-2039-07 | Tumor | Lung Adenocarcinoma |
| TCGA-L9-A8F4-01A-11R-A39D-07 | Tumor | Lung Adenocarcinoma |
| TCGA-35-5375-01A-01R-1628-07 | Tumor | Lung Adenocarcinoma |
| TCGA-50-5939-01A-11R-1628-07 | Tumor | Lung Adenocarcinoma |
| TCGA-55-8510-01A-11R-2403-07 | Tumor | Lung Adenocarcinoma |
| TCGA-38-6178-01A-11R-1755-07 | Tumor | Lung Adenocarcinoma |
| TCGA-MN-A4N4-01A-12R-A24X-07 | Tumor | Lung Adenocarcinoma |
| TCGA-44-7662-01A-11R-2066-07 | Tumor | Lung Adenocarcinoma |
| TCGA-MP-A4T4-01A-11R-A262-07 | Tumor | Lung Adenocarcinoma |
| TCGA-95-A4VP-01A-21R-A262-07 | Tumor | Lung Adenocarcinoma |
| TCGA-55-8206-01A-11R-2241-07 | Tumor | Lung Adenocarcinoma |
| TCGA-86-8585-01A-11R-2403-07 | Tumor | Lung Adenocarcinoma |
| TCGA-95-7562-01A-11R-2241-07 | Tumor | Lung Adenocarcinoma |
| TCGA-05-4426-01A-01R-1206-07 | Tumor | Lung Adenocarcinoma |
| TCGA-55-8508-01A-11R-2403-07 | Tumor | Lung Adenocarcinoma |
| TCGA-99-8025-01A-11R-2241-07 | Tumor | Lung Adenocarcinoma |
| TCGA-44-7672-01A-11R-2066-07 | Tumor | Lung Adenocarcinoma |
| TCGA-NJ-A4YG-01A-22R-A262-07 | Tumor | Lung Adenocarcinoma |
| TCGA-44-8117-01A-11R-2241-07 | Tumor | Lung Adenocarcinoma |
| TCGA-62-A46O-01A-11R-A24H-07 | Tumor | Lung Adenocarcinoma |
| TCGA-93-A4JO-01A-21R-A24X-07 | Tumor | Lung Adenocarcinoma |
| TCGA-44-2666-01A-01R-0946-07 | Tumor | Lung Adenocarcinoma |
| TCGA-67-3774-01A-01R-0946-07 | Tumor | Lung Adenocarcinoma |
| TCGA-86-8358-01A-11R-2326-07 | Tumor | Lung Adenocarcinoma |
| TCGA-62-8394-01A-11R-2326-07 | Tumor | Lung Adenocarcinoma |
| TCGA-MP-A4T6-01A-32R-A262-07 | Tumor | Lung Adenocarcinoma |
| TCGA-55-8514-01A-11R-2403-07 | Tumor | Lung Adenocarcinoma |
| TCGA-44-6147-01A-11R-A278-07 | Tumor | Lung Adenocarcinoma |
| TCGA-64-1681-01A-11R-2066-07 | Tumor | Lung Adenocarcinoma |
| TCGA-50-5944-01A-11R-1755-07 | Tumor | Lung Adenocarcinoma |
| TCGA-44-A47B-01A-11R-A24H-07 | Tumor | Lung Adenocarcinoma |
| TCGA-L4-A4E6-01A-11R-A24H-07 | Tumor | Lung Adenocarcinoma |
| TCGA-49-AARR-01A-11R-A41B-07 | Tumor | Lung Adenocarcinoma |
| TCGA-49-4507-01A-01R-1206-07 | Tumor | Lung Adenocarcinoma |
| TCGA-75-7027-01A-11R-1949-07 | Tumor | Lung Adenocarcinoma |
| TCGA-38-4625-01A-01R-1206-07 | Tumor | Lung Adenocarcinoma |
| TCGA-44-8120-01A-11R-2241-07 | Tumor | Lung Adenocarcinoma |
| TCGA-44-6776-01A-11R-1858-07 | Tumor | Lung Adenocarcinoma |
| TCGA-62-8402-01A-11R-2326-07 | Tumor | Lung Adenocarcinoma |
| TCGA-75-5125-01A-01R-1755-07 | Tumor | Lung Adenocarcinoma |
| TCGA-64-1678-01A-01R-0946-07 | Tumor | Lung Adenocarcinoma |
| TCGA-95-7567-01A-11R-2066-07 | Tumor | Lung Adenocarcinoma |
| TCGA-99-AA5R-01A-11R-A39D-07 | Tumor | Lung Adenocarcinoma |
| TCGA-97-8174-01A-11R-2287-07 | Tumor | Lung Adenocarcinoma |
| TCGA-55-8089-01A-11R-2241-07 | Tumor | Lung Adenocarcinoma |
| TCGA-69-A59K-01A-11R-A262-07 | Tumor | Lung Adenocarcinoma |
| TCGA-55-6978-01A-11R-1949-07 | Tumor | Lung Adenocarcinoma |
| TCGA-78-7153-01A-11R-2039-07 | Tumor | Lung Adenocarcinoma |
| TCGA-05-5715-01A-01R-1628-07 | Tumor | Lung Adenocarcinoma |
| TCGA-69-8254-01A-11R-2287-07 | Tumor | Lung Adenocarcinoma |
| TCGA-J2-A4AE-01A-21R-A24H-07 | Tumor | Lung Adenocarcinoma |
| TCGA-78-7150-01A-21R-2039-07 | Tumor | Lung Adenocarcinoma |
| TCGA-93-A4JN-01A-11R-A24X-07 | Tumor | Lung Adenocarcinoma |
| TCGA-J2-8194-01A-11R-2241-07 | Tumor | Lung Adenocarcinoma |
| TCGA-55-6712-01A-11R-1858-07 | Tumor | Lung Adenocarcinoma |
| TCGA-44-2668-01A-01R-A278-07 | Tumor | Lung Adenocarcinoma |
| TCGA-55-7815-01A-11R-2170-07 | Tumor | Lung Adenocarcinoma |
| TCGA-50-5049-01A-01R-1628-07 | Tumor | Lung Adenocarcinoma |
| TCGA-49-4510-01A-01R-1206-07 | Tumor | Lung Adenocarcinoma |
| TCGA-73-4666-01A-01R-1206-07 | Tumor | Lung Adenocarcinoma |
| TCGA-MP-A5C7-01A-11R-A262-07 | Tumor | Lung Adenocarcinoma |
| TCGA-55-7994-01A-11R-2187-07 | Tumor | Lung Adenocarcinoma |
| TCGA-95-7039-01A-11R-1949-07 | Tumor | Lung Adenocarcinoma |
| TCGA-78-7156-01A-11R-2039-07 | Tumor | Lung Adenocarcinoma |
| TCGA-55-8299-01A-11R-2287-07 | Tumor | Lung Adenocarcinoma |
| TCGA-44-7661-01A-11R-2066-07 | Tumor | Lung Adenocarcinoma |
| TCGA-50-5066-02A-11R-2090-07 | Tumor | Lung Adenocarcinoma |
| TCGA-97-7554-01A-11R-2039-07 | Tumor | Lung Adenocarcinoma |
| TCGA-78-7167-01A-11R-2066-07 | Tumor | Lung Adenocarcinoma |
| TCGA-50-6595-01A-12R-1858-07 | Tumor | Lung Adenocarcinoma |
| TCGA-64-1677-01A-01R-0946-07 | Tumor | Lung Adenocarcinoma |
| TCGA-97-A4M6-01A-11R-A24X-07 | Tumor | Lung Adenocarcinoma |
| TCGA-97-8547-01A-11R-2403-07 | Tumor | Lung Adenocarcinoma |
| TCGA-62-A470-01A-11R-A24H-07 | Tumor | Lung Adenocarcinoma |
| TCGA-97-8171-01A-11R-2287-07 | Tumor | Lung Adenocarcinoma |
| TCGA-44-6148-01A-11R-1755-07 | Tumor | Lung Adenocarcinoma |
| TCGA-MP-A4T7-01A-11R-A24X-07 | Tumor | Lung Adenocarcinoma |
| TCGA-49-4514-01A-21R-1858-07 | Tumor | Lung Adenocarcinoma |
| TCGA-50-5946-01A-11R-1755-07 | Tumor | Lung Adenocarcinoma |
| TCGA-55-8621-01A-11R-2403-07 | Tumor | Lung Adenocarcinoma |
| TCGA-55-8614-01A-11R-2403-07 | Tumor | Lung Adenocarcinoma |
| TCGA-71-8520-01A-11R-2403-07 | Tumor | Lung Adenocarcinoma |
| TCGA-69-7978-01A-11R-2187-07 | Tumor | Lung Adenocarcinoma |
| TCGA-L9-A443-01A-12R-A24H-07 | Tumor | Lung Adenocarcinoma |
| TCGA-55-7573-01A-11R-2039-07 | Tumor | Lung Adenocarcinoma |
| TCGA-78-7154-01A-11R-2039-07 | Tumor | Lung Adenocarcinoma |
| TCGA-44-3918-01B-02R-A277-07 | Tumor | Lung Adenocarcinoma |
| TCGA-S2-AA1A-01A-12R-A39D-07 | Tumor | Lung Adenocarcinoma |
| TCGA-05-5423-01A-01R-1628-07 | Tumor | Lung Adenocarcinoma |
| TCGA-50-6591-01A-11R-1755-07 | Tumor | Lung Adenocarcinoma |
| TCGA-62-8395-01A-11R-2326-07 | Tumor | Lung Adenocarcinoma |
| TCGA-55-7727-01A-11R-2170-07 | Tumor | Lung Adenocarcinoma |
| TCGA-49-4494-01A-01R-1206-07 | Tumor | Lung Adenocarcinoma |
| TCGA-49-4501-01A-01R-1206-07 | Tumor | Lung Adenocarcinoma |
| TCGA-91-6847-01A-11R-1949-07 | Tumor | Lung Adenocarcinoma |
| TCGA-75-5126-01A-01R-1755-07 | Tumor | Lung Adenocarcinoma |
| TCGA-86-A4JF-01A-11R-A24X-07 | Tumor | Lung Adenocarcinoma |
| TCGA-49-AAR2-01A-11R-A39D-07 | Tumor | Lung Adenocarcinoma |
| TCGA-75-6211-01A-11R-1755-07 | Tumor | Lung Adenocarcinoma |
| TCGA-73-4675-01A-01R-1206-07 | Tumor | Lung Adenocarcinoma |
| TCGA-44-2668-01A-01R-0946-07 | Tumor | Lung Adenocarcinoma |
| TCGA-38-4628-01A-01R-1206-07 | Tumor | Lung Adenocarcinoma |
| TCGA-O1-A52J-01A-11R-A262-07 | Tumor | Lung Adenocarcinoma |
| TCGA-05-4396-01A-21R-1858-07 | Tumor | Lung Adenocarcinoma |
| TCGA-44-7660-01A-11R-2066-07 | Tumor | Lung Adenocarcinoma |
| TCGA-86-8279-01A-11R-2287-07 | Tumor | Lung Adenocarcinoma |
| TCGA-97-7937-01A-11R-2170-07 | Tumor | Lung Adenocarcinoma |
| TCGA-05-5420-01A-01R-1628-07 | Tumor | Lung Adenocarcinoma |
| TCGA-78-7542-01A-21R-2066-07 | Tumor | Lung Adenocarcinoma |
| TCGA-67-3771-01A-01R-0946-07 | Tumor | Lung Adenocarcinoma |
| TCGA-38-7271-01A-11R-2039-07 | Tumor | Lung Adenocarcinoma |
| TCGA-78-7163-01A-12R-2066-07 | Tumor | Lung Adenocarcinoma |
| TCGA-73-4658-01A-01R-1755-07 | Tumor | Lung Adenocarcinoma |
| TCGA-75-6207-01A-11R-1755-07 | Tumor | Lung Adenocarcinoma |
| TCGA-78-7160-01A-11R-2039-07 | Tumor | Lung Adenocarcinoma |
| TCGA-49-6743-01A-11R-1858-07 | Tumor | Lung Adenocarcinoma |
| TCGA-05-4425-01A-01R-1755-07 | Tumor | Lung Adenocarcinoma |
| TCGA-78-7143-01A-11R-2039-07 | Tumor | Lung Adenocarcinoma |
| TCGA-55-8087-01A-11R-2241-07 | Tumor | Lung Adenocarcinoma |
| TCGA-55-6986-01A-11R-1949-07 | Tumor | Lung Adenocarcinoma |
| TCGA-44-4112-01A-01R-1107-07 | Tumor | Lung Adenocarcinoma |
| TCGA-69-7765-01A-11R-2170-07 | Tumor | Lung Adenocarcinoma |
| TCGA-55-7281-01A-11R-2039-07 | Tumor | Lung Adenocarcinoma |
| TCGA-50-6597-01A-11R-1858-07 | Tumor | Lung Adenocarcinoma |
| TCGA-73-4677-01A-01R-1206-07 | Tumor | Lung Adenocarcinoma |
| TCGA-05-4434-01A-01R-1206-07 | Tumor | Lung Adenocarcinoma |
| TCGA-MP-A4SW-01A-21R-A24X-07 | Tumor | Lung Adenocarcinoma |
| TCGA-55-6972-01A-11R-1949-07 | Tumor | Lung Adenocarcinoma |
| TCGA-78-7633-01A-11R-2066-07 | Tumor | Lung Adenocarcinoma |
| TCGA-44-2659-01A-01R-0946-07 | Tumor | Lung Adenocarcinoma |
| TCGA-55-6983-01A-11R-1949-07 | Tumor | Lung Adenocarcinoma |
| TCGA-97-A4M0-01A-11R-A24X-07 | Tumor | Lung Adenocarcinoma |
| TCGA-50-5068-01A-01R-1628-07 | Tumor | Lung Adenocarcinoma |
| TCGA-86-A4P8-01A-11R-A24X-07 | Tumor | Lung Adenocarcinoma |
| TCGA-86-A4D0-01A-11R-A24H-07 | Tumor | Lung Adenocarcinoma |
| TCGA-55-8085-01A-11R-2241-07 | Tumor | Lung Adenocarcinoma |
| TCGA-50-5935-01A-11R-1755-07 | Tumor | Lung Adenocarcinoma |
| TCGA-53-7813-01A-11R-2170-07 | Tumor | Lung Adenocarcinoma |
| TCGA-73-4662-01A-01R-1206-07 | Tumor | Lung Adenocarcinoma |
| TCGA-62-8399-01A-21R-2326-07 | Tumor | Lung Adenocarcinoma |
| TCGA-44-7671-01A-11R-2066-07 | Tumor | Lung Adenocarcinoma |
| TCGA-97-7552-01A-11R-2039-07 | Tumor | Lung Adenocarcinoma |
| TCGA-44-6145-01A-11R-1755-07 | Tumor | Lung Adenocarcinoma |
| TCGA-44-7670-01A-11R-2066-07 | Tumor | Lung Adenocarcinoma |
| TCGA-49-AARO-01A-12R-A41B-07 | Tumor | Lung Adenocarcinoma |
| TCGA-44-2657-01A-01R-1107-07 | Tumor | Lung Adenocarcinoma |
| TCGA-55-8204-01A-11R-2241-07 | Tumor | Lung Adenocarcinoma |
| TCGA-64-1676-01A-01R-0946-07 | Tumor | Lung Adenocarcinoma |
| TCGA-91-8496-01A-11R-2403-07 | Tumor | Lung Adenocarcinoma |
| TCGA-35-3615-01A-01R-0946-07 | Tumor | Lung Adenocarcinoma |
| TCGA-55-8096-01A-11R-2241-07 | Tumor | Lung Adenocarcinoma |
| TCGA-67-6215-01A-11R-1755-07 | Tumor | Lung Adenocarcinoma |
| TCGA-53-A4EZ-01A-12R-A24X-07 | Tumor | Lung Adenocarcinoma |
| TCGA-MP-A4SY-01A-21R-A24X-07 | Tumor | Lung Adenocarcinoma |
| TCGA-73-4676-01A-01R-1755-07 | Tumor | Lung Adenocarcinoma |
| TCGA-86-8075-01A-11R-2241-07 | Tumor | Lung Adenocarcinoma |
| TCGA-78-8660-01A-11R-2403-07 | Tumor | Lung Adenocarcinoma |
| TCGA-91-6840-01A-11R-1949-07 | Tumor | Lung Adenocarcinoma |
| TCGA-44-2665-01A-01R-0946-07 | Tumor | Lung Adenocarcinoma |
| TCGA-44-6775-01A-11R-1858-07 | Tumor | Lung Adenocarcinoma |
| TCGA-75-7025-01A-12R-1949-07 | Tumor | Lung Adenocarcinoma |
| TCGA-44-A479-01A-31R-A24H-07 | Tumor | Lung Adenocarcinoma |
| TCGA-44-3917-01B-02R-A277-07 | Tumor | Lung Adenocarcinoma |
| TCGA-62-A472-01A-11R-A24H-07 | Tumor | Lung Adenocarcinoma |
| TCGA-44-6146-01A-11R-1755-07 | Tumor | Lung Adenocarcinoma |
| TCGA-MP-A4SV-01A-11R-A24X-07 | Tumor | Lung Adenocarcinoma |
| TCGA-49-AARN-01A-21R-A41B-07 | Tumor | Lung Adenocarcinoma |
| TCGA-50-5072-01A-21R-1858-07 | Tumor | Lung Adenocarcinoma |
| TCGA-55-5899-01A-11R-1628-07 | Tumor | Lung Adenocarcinoma |
| TCGA-97-A4LX-01A-11R-A24X-07 | Tumor | Lung Adenocarcinoma |
| TCGA-L9-A444-01A-21R-A24H-07 | Tumor | Lung Adenocarcinoma |
| TCGA-55-8615-01A-11R-2403-07 | Tumor | Lung Adenocarcinoma |
| TCGA-50-7109-01A-11R-2039-07 | Tumor | Lung Adenocarcinoma |
| TCGA-91-7771-01A-11R-2170-07 | Tumor | Lung Adenocarcinoma |
| TCGA-97-A4M1-01A-11R-A24X-07 | Tumor | Lung Adenocarcinoma |
| TCGA-44-5644-01A-21R-2039-07 | Tumor | Lung Adenocarcinoma |
| TCGA-78-7536-01A-11R-2066-07 | Tumor | Lung Adenocarcinoma |
| TCGA-MP-A4TI-01A-21R-A24X-07 | Tumor | Lung Adenocarcinoma |
| TCGA-05-4403-01A-01R-1206-07 | Tumor | Lung Adenocarcinoma |
| TCGA-44-2666-01B-02R-A277-07 | Tumor | Lung Adenocarcinoma |
| TCGA-99-7458-01A-11R-2039-07 | Tumor | Lung Adenocarcinoma |
| TCGA-64-5778-01A-01R-1628-07 | Tumor | Lung Adenocarcinoma |
| TCGA-78-7145-01A-11R-2039-07 | Tumor | Lung Adenocarcinoma |
| TCGA-NJ-A4YP-01A-11R-A262-07 | Tumor | Lung Adenocarcinoma |
| TCGA-55-7283-01A-11R-2039-07 | Tumor | Lung Adenocarcinoma |
| TCGA-55-8208-01A-11R-2241-07 | Tumor | Lung Adenocarcinoma |
| TCGA-55-8302-01A-11R-2326-07 | Tumor | Lung Adenocarcinoma |
| TCGA-05-4384-01A-01R-1755-07 | Tumor | Lung Adenocarcinoma |
| TCGA-55-8511-01A-11R-2403-07 | Tumor | Lung Adenocarcinoma |
| TCGA-97-7941-01A-11R-2187-07 | Tumor | Lung Adenocarcinoma |
| TCGA-62-8397-01A-11R-2326-07 | Tumor | Lung Adenocarcinoma |
| TCGA-91-6830-01A-11R-1949-07 | Tumor | Lung Adenocarcinoma |
| TCGA-44-3398-01A-01R-1107-07 | Tumor | Lung Adenocarcinoma |
| TCGA-55-7913-01B-11R-2241-07 | Tumor | Lung Adenocarcinoma |
| TCGA-44-2656-01A-02R-A278-07 | Tumor | Lung Adenocarcinoma |
| TCGA-55-1592-01A-01R-0946-07 | Tumor | Lung Adenocarcinoma |
| TCGA-64-5775-01A-01R-1628-07 | Tumor | Lung Adenocarcinoma |
| TCGA-44-7659-01A-11R-2066-07 | Tumor | Lung Adenocarcinoma |
| TCGA-55-A4DG-01A-11R-A24H-07 | Tumor | Lung Adenocarcinoma |
| TCGA-55-6985-01A-11R-1949-07 | Tumor | Lung Adenocarcinoma |
| TCGA-86-8054-01A-11R-2241-07 | Tumor | Lung Adenocarcinoma |
| TCGA-38-4629-01A-02R-1206-07 | Tumor | Lung Adenocarcinoma |
| TCGA-78-7147-01A-11R-2039-07 | Tumor | Lung Adenocarcinoma |
| TCGA-55-8092-01A-11R-2241-07 | Tumor | Lung Adenocarcinoma |
| TCGA-75-6205-01A-11R-1755-07 | Tumor | Lung Adenocarcinoma |
| TCGA-86-A456-01A-11R-A24H-07 | Tumor | Lung Adenocarcinoma |
| TCGA-05-5429-01A-01R-1628-07 | Tumor | Lung Adenocarcinoma |
| TCGA-55-A48X-01A-11R-A24H-07 | Tumor | Lung Adenocarcinoma |
| TCGA-86-8673-01A-11R-2403-07 | Tumor | Lung Adenocarcinoma |
| TCGA-86-8674-01A-21R-2403-07 | Tumor | Lung Adenocarcinoma |
| TCGA-49-6744-01A-11R-1858-07 | Tumor | Lung Adenocarcinoma |
| TCGA-44-5643-01A-01R-1628-07 | Tumor | Lung Adenocarcinoma |
| TCGA-05-4382-01A-01R-1206-07 | Tumor | Lung Adenocarcinoma |
| TCGA-49-AARQ-01A-11R-A41B-07 | Tumor | Lung Adenocarcinoma |
| TCGA-55-8094-01A-11R-2241-07 | Tumor | Lung Adenocarcinoma |
| TCGA-MP-A4TC-01A-11R-A24X-07 | Tumor | Lung Adenocarcinoma |
| TCGA-49-AAR0-01A-21R-A39D-07 | Tumor | Lung Adenocarcinoma |
| TCGA-05-4427-01A-21R-1858-07 | Tumor | Lung Adenocarcinoma |
| TCGA-75-6214-01A-41R-1949-07 | Tumor | Lung Adenocarcinoma |
| TCGA-69-7763-01A-11R-2170-07 | Tumor | Lung Adenocarcinoma |
| TCGA-86-8076-01A-31R-2241-07 | Tumor | Lung Adenocarcinoma |
| TCGA-78-7537-01A-11R-2066-07 | Tumor | Lung Adenocarcinoma |
| TCGA-78-7166-01A-12R-2066-07 | Tumor | Lung Adenocarcinoma |
| TCGA-55-7995-01A-11R-2187-07 | Tumor | Lung Adenocarcinoma |
| TCGA-35-4123-01A-01R-1107-07 | Tumor | Lung Adenocarcinoma |
| TCGA-55-7576-01A-11R-2066-07 | Tumor | Lung Adenocarcinoma |
| TCGA-64-5781-01A-01R-1628-07 | Tumor | Lung Adenocarcinoma |
| TCGA-L9-A50W-01A-12R-A39D-07 | Tumor | Lung Adenocarcinoma |
| TCGA-97-A4M5-01A-11R-A24X-07 | Tumor | Lung Adenocarcinoma |
| TCGA-35-4122-01A-01R-1107-07 | Tumor | Lung Adenocarcinoma |
| TCGA-55-A48Z-01A-12R-A24X-07 | Tumor | Lung Adenocarcinoma |
| TCGA-86-6851-01A-11R-1949-07 | Tumor | Lung Adenocarcinoma |
| TCGA-NJ-A4YF-01A-12R-A262-07 | Tumor | Lung Adenocarcinoma |
| TCGA-05-4389-01A-01R-1206-07 | Tumor | Lung Adenocarcinoma |
| TCGA-NJ-A4YQ-01A-11R-A262-07 | Tumor | Lung Adenocarcinoma |
| TCGA-55-6981-01A-11R-1949-07 | Tumor | Lung Adenocarcinoma |
| TCGA-97-7546-01A-11R-2039-07 | Tumor | Lung Adenocarcinoma |
| TCGA-MN-A4N1-01A-11R-A24X-07 | Tumor | Lung Adenocarcinoma |
| TCGA-62-A471-01A-12R-A24H-07 | Tumor | Lung Adenocarcinoma |
| TCGA-55-7570-01A-11R-2039-07 | Tumor | Lung Adenocarcinoma |
| TCGA-44-3917-01A-01R-A278-07 | Tumor | Lung Adenocarcinoma |
| TCGA-55-A4DF-01A-11R-A24H-07 | Tumor | Lung Adenocarcinoma |
| TCGA-50-5946-02A-11R-2090-07 | Tumor | Lung Adenocarcinoma |
| TCGA-78-8648-01A-11R-2403-07 | Tumor | Lung Adenocarcinoma |
| TCGA-67-3770-01A-01R-0946-07 | Tumor | Lung Adenocarcinoma |
| TCGA-50-5936-01A-11R-1628-07 | Tumor | Lung Adenocarcinoma |
| TCGA-67-6216-01A-11R-1755-07 | Tumor | Lung Adenocarcinoma |
| TCGA-69-7974-01A-11R-2187-07 | Tumor | Lung Adenocarcinoma |
| TCGA-86-8281-01A-11R-2287-07 | Tumor | Lung Adenocarcinoma |
| TCGA-44-2662-01B-02R-A277-07 | Tumor | Lung Adenocarcinoma |
| TCGA-93-A4JQ-01A-11R-A24X-07 | Tumor | Lung Adenocarcinoma |
| TCGA-55-8207-01A-11R-2241-07 | Tumor | Lung Adenocarcinoma |
| TCGA-97-8176-01A-11R-2403-07 | Tumor | Lung Adenocarcinoma |
| TCGA-86-8280-01A-11R-2287-07 | Tumor | Lung Adenocarcinoma |
| TCGA-MP-A4T8-01A-11R-A24X-07 | Tumor | Lung Adenocarcinoma |
| TCGA-55-A492-01A-11R-A24H-07 | Tumor | Lung Adenocarcinoma |
| TCGA-J2-8192-01A-11R-2241-07 | Tumor | Lung Adenocarcinoma |
| TCGA-50-8460-01A-11R-2326-07 | Tumor | Lung Adenocarcinoma |
| TCGA-80-5607-01A-31R-1949-07 | Tumor | Lung Adenocarcinoma |
| TCGA-NJ-A55A-01A-11R-A262-07 | Tumor | Lung Adenocarcinoma |
| TCGA-55-8097-01A-11R-2241-07 | Tumor | Lung Adenocarcinoma |
| TCGA-44-6147-01A-11R-1755-07 | Tumor | Lung Adenocarcinoma |
| TCGA-55-A490-01A-11R-A466-07 | Tumor | Lung Adenocarcinoma |
| TCGA-86-7711-01A-11R-2066-07 | Tumor | Lung Adenocarcinoma |
| TCGA-MP-A4TK-01A-11R-A24X-07 | Tumor | Lung Adenocarcinoma |
| TCGA-50-5066-01A-01R-1628-07 | Tumor | Lung Adenocarcinoma |
| TCGA-44-2666-01A-01R-A278-07 | Tumor | Lung Adenocarcinoma |
| TCGA-50-5942-01A-21R-1755-07 | Tumor | Lung Adenocarcinoma |
| TCGA-69-8453-01A-12R-2326-07 | Tumor | Lung Adenocarcinoma |
| TCGA-44-8119-01A-11R-2241-07 | Tumor | Lung Adenocarcinoma |
| TCGA-78-7146-01A-11R-2039-07 | Tumor | Lung Adenocarcinoma |
| TCGA-91-8499-01A-11R-2403-07 | Tumor | Lung Adenocarcinoma |
| TCGA-55-6970-01A-11R-1949-07 | Tumor | Lung Adenocarcinoma |
| TCGA-05-4420-01A-01R-1206-07 | Tumor | Lung Adenocarcinoma |
| TCGA-86-7701-01A-11R-2170-07 | Tumor | Lung Adenocarcinoma |
| TCGA-NJ-A55R-01A-11R-A262-07 | Tumor | Lung Adenocarcinoma |
| TCGA-05-4397-01A-01R-1206-07 | Tumor | Lung Adenocarcinoma |
| TCGA-53-7626-01A-12R-2066-07 | Tumor | Lung Adenocarcinoma |
| TCGA-95-7944-01A-11R-2187-07 | Tumor | Lung Adenocarcinoma |
| TCGA-44-2656-01B-06R-A277-07 | Tumor | Lung Adenocarcinoma |
| TCGA-73-A9RS-01A-11R-A41B-07 | Tumor | Lung Adenocarcinoma |
| TCGA-44-7667-01A-31R-2066-07 | Tumor | Lung Adenocarcinoma |
| TCGA-NJ-A55O-01A-11R-A262-07 | Tumor | Lung Adenocarcinoma |
| TCGA-55-8301-01A-11R-2287-07 | Tumor | Lung Adenocarcinoma |
| TCGA-05-4415-01A-22R-1858-07 | Tumor | Lung Adenocarcinoma |
| TCGA-91-6835-01A-11R-1858-07 | Tumor | Lung Adenocarcinoma |
| TCGA-55-7725-01A-11R-2170-07 | Tumor | Lung Adenocarcinoma |
| TCGA-55-8506-01A-11R-2403-07 | Tumor | Lung Adenocarcinoma |
| TCGA-50-5931-01A-11R-1755-07 | Tumor | Lung Adenocarcinoma |
| TCGA-44-5645-01A-01R-1628-07 | Tumor | Lung Adenocarcinoma |
| TCGA-55-A494-01A-11R-A24X-07 | Tumor | Lung Adenocarcinoma |
| TCGA-50-5930-01A-11R-1755-07 | Tumor | Lung Adenocarcinoma |
| TCGA-38-4627-01A-01R-1206-07 | Tumor | Lung Adenocarcinoma |
| TCGA-67-4679-01B-01R-1755-07 | Tumor | Lung Adenocarcinoma |
| TCGA-55-7903-01A-11R-2170-07 | Tumor | Lung Adenocarcinoma |
| TCGA-55-8619-01A-11R-2403-07 | Tumor | Lung Adenocarcinoma |
