# Supplementary material for: Long non-coding RNA LINC01559 exerts oncogenic role via enhancing autophagy in lung adenocarcinoma
Source: Cancer Cell Int. 2021 Nov 25;21:624. doi: 10.1186/s12935-021-02338-4 (PMC8614059; doi:10.1186/s12935-021-02338-4)
Supplement: Supplementary file 3 — Additional file 3. Table S3. [file 12935_2021_2338_MOESM3_ESM.docx]

Table S3. Sequences of siRNAs.

| Names | Sequences |
| --- | --- |
| Si-LINC01559#1 | GTAGGTGACTACAGTTAAT |
| Si-LINC01559#2 | GCAAGAAGCTGGAAATCGA |
