# Supplementary material for: Long non-coding RNA LINC01559 exerts oncogenic role via enhancing autophagy in lung adenocarcinoma
Source: Cancer Cell Int. 2021 Nov 25;21:624. doi: 10.1186/s12935-021-02338-4 (PMC8614059; doi:10.1186/s12935-021-02338-4)
Supplement: Supplementary file 4 — Additional file 4. Table S4. [file 12935_2021_2338_MOESM4_ESM.docx]

Table S4. Correlation of autophagy genes and lncRNAs.

| ARGgene | lncRNA | cor | pvalue |
| --- | --- | --- | --- |
| GABARAP | Z68871.1 | -0.50461 | 6.61E-36 |
| GABARAP | AC073046.1 | -0.48992 | 1.20E-33 |
| GABARAP | AC073569.2 | -0.48403 | 9.02E-33 |
| GABARAP | AC108010.1 | -0.48302 | 1.27E-32 |
| BIRC5 | AL133355.1 | -0.47467 | 2.05E-31 |
| HSPA8 | AC108673.3 | -0.46315 | 8.42E-30 |
| EIF2S1 | AC010503.4 | -0.45909 | 3.02E-29 |
| HSPA8 | AC073896.4 | -0.45751 | 4.93E-29 |
| GABARAP | LINC00630 | -0.45742 | 5.07E-29 |
| GABARAP | AL049840.2 | -0.45641 | 6.93E-29 |
| HSPA8 | AC135050.6 | -0.45634 | 7.09E-29 |
| GABARAP | AC022150.4 | -0.4522 | 2.53E-28 |
| BIRC5 | TBX5-AS1 | -0.44864 | 7.44E-28 |
| PELP1 | LINC01094 | -0.44853 | 7.68E-28 |
| FADD | PSMA3-AS1 | -0.44634 | 1.48E-27 |
| GABARAP | AC004918.3 | -0.44633 | 1.49E-27 |
| BIRC5 | AC021016.2 | -0.44128 | 6.66E-27 |
| GABARAP | AL049840.1 | -0.44124 | 6.75E-27 |
| HSPA8 | AC005261.3 | -0.441 | 7.23E-27 |
| GABARAP | AC020915.2 | -0.43754 | 1.99E-26 |
| GABARAP | AC015849.3 | -0.43613 | 2.99E-26 |
| MLST8 | AC096921.2 | -0.43605 | 3.07E-26 |
| PIK3R4 | FLJ20021 | -0.43485 | 4.33E-26 |
| GABARAP | RHOA-IT1 | -0.43457 | 4.70E-26 |
| ATG7 | AC010503.4 | -0.4336 | 6.21E-26 |
| GABARAP | AC007216.4 | -0.4318 | 1.04E-25 |
| GABARAP | Z82243.1 | -0.43156 | 1.11E-25 |
| HSPA8 | AC103691.1 | -0.43093 | 1.33E-25 |
| HSPA8 | AC024075.2 | -0.42898 | 2.31E-25 |
| GAPDH | AC135050.6 | -0.42882 | 2.42E-25 |
| GABARAP | AC092611.2 | -0.42839 | 2.73E-25 |
| GABARAP | AC021078.1 | -0.42802 | 3.03E-25 |
| GABARAP | ERVK13-1 | -0.42768 | 3.34E-25 |
| GABARAP | AL133243.2 | -0.42724 | 3.78E-25 |
| EIF2S1 | Z69706.1 | -0.42723 | 3.79E-25 |
| GABARAP | AC002128.2 | -0.42683 | 4.24E-25 |
| KIF5B | FLJ20021 | -0.42616 | 5.12E-25 |
| GABARAP | AC138932.5 | -0.42609 | 5.22E-25 |
| GABARAP | CR936218.1 | -0.42536 | 6.39E-25 |
| GABARAP | AL109614.1 | -0.42488 | 7.32E-25 |
| GABARAP | AC004492.1 | -0.42476 | 7.56E-25 |
| GABARAP | NPTN-IT1 | -0.42438 | 8.40E-25 |
| HSPA8 | AL136295.7 | -0.42413 | 9.01E-25 |
| EIF2S1 | AL109936.2 | -0.42381 | 9.84E-25 |
| GABARAP | AP000692.1 | -0.42367 | 1.02E-24 |
| GABARAP | AC008906.1 | -0.42352 | 1.07E-24 |
| GABARAP | Z98884.2 | -0.42268 | 1.35E-24 |
| P4HB | AC024075.1 | -0.4223 | 1.50E-24 |
| GABARAP | AC234775.3 | -0.42224 | 1.52E-24 |
| FAS | AL354892.2 | -0.42221 | 1.53E-24 |
| GABARAP | AC253536.3 | -0.42184 | 1.70E-24 |
| GABARAP | AC087481.3 | -0.42179 | 1.72E-24 |
| GABARAP | C1RL-AS1 | -0.42142 | 1.91E-24 |
| FADD | AC008124.1 | -0.42125 | 2.00E-24 |
| GABARAP | AC138393.3 | -0.42115 | 2.06E-24 |
| MLST8 | AC024075.3 | -0.42067 | 2.35E-24 |
| GABARAP | AC067852.3 | -0.42041 | 2.51E-24 |
| GABARAP | FLNB-AS1 | -0.41972 | 3.04E-24 |
| GABARAP | AL513365.2 | -0.41953 | 3.21E-24 |
| GABARAP | AC073487.1 | -0.41949 | 3.24E-24 |
| GABARAP | AC008770.3 | -0.41945 | 3.27E-24 |
| HSPA8 | AC003102.1 | -0.41844 | 4.32E-24 |
| GABARAP | ABALON | -0.41811 | 4.72E-24 |
| HSPA8 | AP000254.1 | -0.41785 | 5.06E-24 |
| CHMP4B | OIP5-AS1 | -0.41783 | 5.09E-24 |
| GABARAP | SCARNA9 | -0.41781 | 5.12E-24 |
| GABARAP | AL359915.2 | -0.41662 | 7.07E-24 |
| BIRC5 | AC093278.2 | -0.41571 | 9.05E-24 |
| GABARAP | AC090948.2 | -0.41529 | 1.01E-23 |
| GABARAP | AL031670.1 | -0.41473 | 1.18E-23 |
| GABARAP | AL354989.1 | -0.41461 | 1.22E-23 |
| GABARAP | AC007216.3 | -0.41418 | 1.37E-23 |
| BIRC5 | AP002840.2 | -0.41373 | 1.54E-23 |
| GABARAP | AL031717.1 | -0.41368 | 1.56E-23 |
| ITGB1 | AC009065.4 | -0.41272 | 2.02E-23 |
| GABARAP | AL049840.5 | -0.41266 | 2.05E-23 |
| GABARAP | AC018690.1 | -0.41254 | 2.12E-23 |
| GABARAP | LIMS1-AS1 | -0.41213 | 2.37E-23 |
| GABARAP | AC124283.3 | -0.412 | 2.45E-23 |
| HSPA8 | AL035071.1 | -0.41159 | 2.74E-23 |
| ITGB1 | RPARP-AS1 | -0.41146 | 2.83E-23 |
| GABARAP | SAP30L-AS1 | -0.41146 | 2.83E-23 |
| GABARAP | MCM3AP-AS1 | -0.41124 | 3.01E-23 |
| GABARAP | AC091057.1 | -0.41107 | 3.14E-23 |
| BAX | OIP5-AS1 | -0.41094 | 3.25E-23 |
| GABARAP | AC008982.2 | -0.41083 | 3.35E-23 |
| GABARAP | AF117829.1 | -0.41061 | 3.55E-23 |
| MAPK8 | FLJ20021 | -0.41035 | 3.81E-23 |
| GABARAP | AL021578.1 | -0.41034 | 3.81E-23 |
| NBR1 | AC073611.1 | -0.41005 | 4.12E-23 |
| ATG16L2 | ERVK13-1 | 0.400026 | 5.62E-22 |
| ATF6 | RPS6KA2-IT1 | 0.400059 | 5.57E-22 |
| FOXO3 | AL139011.1 | 0.400124 | 5.48E-22 |
| CTSB | AC145098.1 | 0.400127 | 5.48E-22 |
| ATG16L2 | AC106782.6 | 0.400133 | 5.47E-22 |
| RB1 | AL158166.1 | 0.400146 | 5.45E-22 |
| UVRAG | AC108449.2 | 0.400158 | 5.43E-22 |
| KLHL24 | UGDH-AS1 | 0.400177 | 5.41E-22 |
| CCR2 | LINC02345 | 0.400192 | 5.39E-22 |
| CASP8 | SP2-AS1 | 0.400213 | 5.36E-22 |
| IFNG | AL357992.1 | 0.400254 | 5.30E-22 |
| GOPC | AC138956.2 | 0.400283 | 5.26E-22 |
| ATG12 | AC027117.1 | 0.400308 | 5.23E-22 |
| ATG12 | AC009948.4 | 0.400308 | 5.23E-22 |
| CCR2 | PAXIP1-AS2 | 0.400318 | 5.21E-22 |
| DAPK2 | AL162586.1 | 0.400326 | 5.20E-22 |
| ATG2B | AC004771.1 | 0.400339 | 5.19E-22 |
| BIRC6 | AC012360.3 | 0.400343 | 5.18E-22 |
| PIK3C3 | AC004908.3 | 0.400398 | 5.11E-22 |
| NAF1 | AC112496.1 | 0.400411 | 5.09E-22 |
| EDEM1 | LINC-PINT | 0.400422 | 5.08E-22 |
| MBTPS2 | AC108449.2 | 0.400433 | 5.06E-22 |
| RPS6KB1 | LINC01534 | 0.400462 | 5.03E-22 |
| ITGB1 | AP000695.2 | 0.400475 | 5.01E-22 |
| CFLAR | TAPT1-AS1 | 0.4005 | 4.98E-22 |
| ERN1 | AC012181.2 | 0.400503 | 4.97E-22 |
| MBTPS2 | AC090948.2 | 0.400516 | 4.96E-22 |
| UVRAG | AL133243.2 | 0.400535 | 4.93E-22 |
| CCR2 | AC147651.3 | 0.40054 | 4.93E-22 |
| PRKAR1A | AC008669.1 | 0.400571 | 4.89E-22 |
| MTOR | AC022150.4 | 0.400576 | 4.88E-22 |
| DRAM1 | HAGLR | 0.400581 | 4.87E-22 |
| RAB5A | AC090181.2 | 0.400584 | 4.87E-22 |
| NAF1 | USP46-AS1 | 0.400584 | 4.87E-22 |
| FOXO1 | MIR222HG | 0.400585 | 4.87E-22 |
| PTEN | AC004241.3 | 0.400594 | 4.86E-22 |
| MBTPS2 | AC011939.2 | 0.400594 | 4.86E-22 |
| RPS6KB1 | AC026367.3 | 0.400598 | 4.85E-22 |
| RAB24 | LINC00265 | 0.400601 | 4.85E-22 |
| FOXO3 | AL158166.2 | 0.400619 | 4.83E-22 |
| PIK3C3 | AC020915.3 | 0.400625 | 4.82E-22 |
| CASP4 | USP30-AS1 | 0.400638 | 4.80E-22 |
| GOPC | ATP1B3-AS1 | 0.400651 | 4.79E-22 |
| EIF2AK2 | AC026368.1 | 0.400661 | 4.77E-22 |
| RB1 | AL354989.1 | 0.400685 | 4.75E-22 |
| ATG4D | AL024508.2 | 0.400695 | 4.73E-22 |
| GNAI3 | AC026356.1 | 0.40071 | 4.72E-22 |
| MBTPS2 | AL359962.2 | 0.400741 | 4.68E-22 |
| KIF5B | AC026356.1 | 0.400758 | 4.66E-22 |
| NAF1 | AC004656.1 | 0.400771 | 4.64E-22 |
| MAPK8 | AC020571.1 | 0.400776 | 4.64E-22 |
| ATG16L2 | Z98884.2 | 0.40078 | 4.63E-22 |
| TSC1 | AC090517.2 | 0.400789 | 4.62E-22 |
| TSC1 | ARHGAP31-AS1 | 0.400825 | 4.58E-22 |
| NLRC4 | AC025857.2 | 0.400827 | 4.58E-22 |
| RB1 | FAM13A-AS1 | 0.400828 | 4.57E-22 |
| DAPK2 | PSMA3-AS1 | 0.400841 | 4.56E-22 |
| ATG2B | DGCR11 | 0.400856 | 4.54E-22 |
| GOPC | AC079907.1 | 0.400868 | 4.53E-22 |
| CAPN10 | AL353622.1 | 0.40087 | 4.52E-22 |
| IFNG | DLEU2 | 0.400879 | 4.52E-22 |
| TSC1 | AL136320.1 | 0.400895 | 4.50E-22 |
| TSC1 | STARD7-AS1 | 0.40091 | 4.48E-22 |
| ATG12 | AC073896.3 | 0.400911 | 4.48E-22 |
| TSC1 | AL359697.1 | 0.400949 | 4.43E-22 |
| KLHL24 | AC025171.2 | 0.400977 | 4.40E-22 |
| TM9SF1 | DHRS4-AS1 | 0.40098 | 4.40E-22 |
| PIK3R4 | AC004918.3 | 0.400986 | 4.39E-22 |
| CFLAR | AC008764.2 | 0.40099 | 4.39E-22 |
| GOPC | AC138956.1 | 0.401014 | 4.36E-22 |
| ATG7 | AP001429.1 | 0.401029 | 4.34E-22 |
| NAF1 | AC133644.2 | 0.401048 | 4.32E-22 |
| GOPC | AP001528.2 | 0.401058 | 4.31E-22 |
| FOXO3 | AC099343.2 | 0.401095 | 4.27E-22 |
| KIF5B | AC087276.1 | 0.401146 | 4.22E-22 |
| SH3GLB1 | AC026356.1 | 0.401152 | 4.21E-22 |
| MAPK8 | AC048341.1 | 0.401169 | 4.19E-22 |
| RPS6KB1 | AP000786.1 | 0.40117 | 4.19E-22 |
| PIK3R4 | AC024075.3 | 0.401175 | 4.18E-22 |
| MTOR | AL158212.3 | 0.401195 | 4.16E-22 |
| FOXO1 | LINC01534 | 0.401218 | 4.14E-22 |
| CASP8 | Z68871.1 | 0.40123 | 4.12E-22 |
| RB1CC1 | AC078883.1 | 0.40124 | 4.11E-22 |
| CAPN10 | AL008582.1 | 0.40125 | 4.10E-22 |
| EIF2AK2 | TBILA | 0.401255 | 4.10E-22 |
| RAB33B | AC096741.1 | 0.40127 | 4.08E-22 |
| RAB5A | SMC5-AS1 | 0.401311 | 4.04E-22 |
| MLST8 | SNHG11 | 0.401387 | 3.96E-22 |
| KLHL24 | AC008982.2 | 0.401407 | 3.94E-22 |
| ATG4C | LINC01094 | 0.401411 | 3.94E-22 |
| ERN1 | AC138956.1 | 0.401424 | 3.92E-22 |
| UVRAG | AC027097.2 | 0.401435 | 3.91E-22 |
| FOXO3 | AL162724.1 | 0.401449 | 3.90E-22 |
| TSC1 | AC063965.1 | 0.401482 | 3.87E-22 |
| ATG12 | LINC02100 | 0.401487 | 3.86E-22 |
| CFLAR | AC090617.5 | 0.401497 | 3.85E-22 |
| TSC1 | AC011815.1 | 0.401515 | 3.83E-22 |
| UVRAG | AC063965.1 | 0.401517 | 3.83E-22 |
| PIK3C3 | SAMD12-AS1 | 0.401538 | 3.81E-22 |
| RAB33B | AC011477.2 | 0.401539 | 3.81E-22 |
| IFNG | FAM30A | 0.401551 | 3.80E-22 |
| KLHL24 | RPS6KA2-IT1 | 0.401569 | 3.78E-22 |
| MTOR | HCG11 | 0.401574 | 3.78E-22 |
| KIF5B | AC004832.5 | 0.401604 | 3.75E-22 |
| DAPK2 | AL365330.1 | 0.401607 | 3.74E-22 |
| KLHL24 | C1RL-AS1 | 0.401688 | 3.67E-22 |
| ATF6 | AC108449.2 | 0.401694 | 3.66E-22 |
| NAF1 | RPS6KA2-IT1 | 0.401716 | 3.64E-22 |
| RPS6KB1 | AC139887.2 | 0.401724 | 3.63E-22 |
| CAPN10 | AL391244.1 | 0.401726 | 3.63E-22 |
| EIF2AK3 | SOS1-IT1 | 0.401749 | 3.61E-22 |
| NAF1 | HCG11 | 0.40177 | 3.59E-22 |
| UVRAG | AC067852.3 | 0.401815 | 3.55E-22 |
| TSC1 | AC011450.1 | 0.401822 | 3.54E-22 |
| GOPC | LINC01376 | 0.401831 | 3.53E-22 |
| TSC1 | AP005131.7 | 0.401831 | 3.53E-22 |
| ATG7 | MIR222HG | 0.401832 | 3.53E-22 |
| ATG16L2 | AL139089.1 | 0.401842 | 3.52E-22 |
| HIF1A | AC099850.3 | 0.401862 | 3.50E-22 |
| UVRAG | NUTM2B-AS1 | 0.401864 | 3.50E-22 |
| ATF6 | NORAD | 0.40187 | 3.50E-22 |
| ATG4B | DBH-AS1 | 0.40188 | 3.49E-22 |
| CXCR4 | PSMB8-AS1 | 0.4019 | 3.47E-22 |
| KIF5B | AC012557.1 | 0.401904 | 3.47E-22 |
| RAB33B | AC092801.1 | 0.401919 | 3.45E-22 |
| ATG12 | AC093227.1 | 0.401952 | 3.43E-22 |
| KIF5B | AC108010.1 | 0.401955 | 3.42E-22 |
| FOXO3 | LINC01355 | 0.40197 | 3.41E-22 |
| UVRAG | AC004918.3 | 0.401977 | 3.40E-22 |
| RAB33B | AC007038.1 | 0.401979 | 3.40E-22 |
| FOXO1 | AGAP1-IT1 | 0.401983 | 3.40E-22 |
| PIK3C3 | AC015813.1 | 0.401984 | 3.40E-22 |
| FOXO1 | AL359715.3 | 0.401999 | 3.38E-22 |
| RAB33B | RHOA-IT1 | 0.40204 | 3.35E-22 |
| BIRC5 | CYTOR | 0.402057 | 3.33E-22 |
| UVRAG | AC018752.1 | 0.402063 | 3.33E-22 |
| TSC1 | AC124319.1 | 0.402085 | 3.31E-22 |
| UVRAG | AL353804.2 | 0.402096 | 3.30E-22 |
| RB1CC1 | AC068152.1 | 0.402103 | 3.29E-22 |
| UVRAG | HCG18 | 0.402116 | 3.28E-22 |
| MAPK8 | THUMPD3-AS1 | 0.402122 | 3.28E-22 |
| BIRC6 | SRD5A3-AS1 | 0.402131 | 3.27E-22 |
| FOXO1 | LINC01578 | 0.402155 | 3.25E-22 |
| ATG12 | AF131215.6 | 0.40217 | 3.24E-22 |
| MBTPS2 | A2M-AS1 | 0.402177 | 3.23E-22 |
| CFLAR | AC019117.1 | 0.402183 | 3.23E-22 |
| PIK3C3 | AC084117.1 | 0.402193 | 3.22E-22 |
| MBTPS2 | AC058791.1 | 0.402207 | 3.21E-22 |
| KLHL24 | AL157394.1 | 0.40221 | 3.20E-22 |
| PIK3C3 | ZNF460-AS1 | 0.402218 | 3.20E-22 |
| FOXO1 | AL139041.1 | 0.402222 | 3.19E-22 |
| EIF2AK2 | STARD4-AS1 | 0.402242 | 3.18E-22 |
| DLC1 | TBX5-AS1 | 0.40225 | 3.17E-22 |
| RAB33B | SCARNA9 | 0.402252 | 3.17E-22 |
| RB1CC1 | AL450263.1 | 0.402285 | 3.14E-22 |
| WDFY3 | AL355388.1 | 0.402302 | 3.13E-22 |
| ATG7 | AC234775.3 | 0.402357 | 3.08E-22 |
| RB1 | AC074032.1 | 0.40236 | 3.08E-22 |
| EEF2K | AC025171.2 | 0.402379 | 3.07E-22 |
| IFNG | LINC02100 | 0.40238 | 3.07E-22 |
| CAPN10 | AC007383.2 | 0.402479 | 2.99E-22 |
| EIF2AK2 | AL606834.1 | 0.402485 | 2.98E-22 |
| RB1 | MAST4-AS1 | 0.402488 | 2.98E-22 |
| RB1 | AC108010.1 | 0.402488 | 2.98E-22 |
| ATG4B | AC107375.1 | 0.402495 | 2.98E-22 |
| CFLAR | AC130456.3 | 0.402532 | 2.95E-22 |
| KIF5B | AC138393.3 | 0.402567 | 2.92E-22 |
| KIF5B | AL133445.2 | 0.402571 | 2.92E-22 |
| RB1 | PPP3CB-AS1 | 0.402582 | 2.91E-22 |
| GOPC | NARF-IT1 | 0.402582 | 2.91E-22 |
| MAPK8IP1 | AC106897.1 | 0.40261 | 2.89E-22 |
| ATG7 | AC068790.5 | 0.402622 | 2.88E-22 |
| PIK3C3 | AC135050.5 | 0.402628 | 2.88E-22 |
| FOXO3 | AC009318.3 | 0.40264 | 2.87E-22 |
| CAPN10 | CD27-AS1 | 0.402642 | 2.87E-22 |
| BIRC6 | AC232271.1 | 0.402643 | 2.87E-22 |
| CFLAR | AC087752.3 | 0.402658 | 2.85E-22 |
| ULK3 | AC020907.4 | 0.402668 | 2.85E-22 |
| RPS6KB1 | AC011468.5 | 0.402695 | 2.83E-22 |
| RB1CC1 | RBMS3-AS3 | 0.402707 | 2.82E-22 |
| PIK3C3 | AC079907.1 | 0.402725 | 2.80E-22 |
| ATG2B | AC004982.1 | 0.402753 | 2.78E-22 |
| ATG7 | AC018752.1 | 0.402769 | 2.77E-22 |
| ATG16L2 | AC002553.1 | 0.402848 | 2.72E-22 |
| CDKN2A | AC011445.2 | 0.402862 | 2.71E-22 |
| PIK3R4 | AC245014.3 | 0.402869 | 2.70E-22 |
| GOPC | AC009090.3 | 0.402894 | 2.68E-22 |
| GOPC | AL049840.3 | 0.402905 | 2.68E-22 |
| TSC2 | AC133919.2 | 0.402907 | 2.68E-22 |
| SPNS1 | AC087741.1 | 0.402921 | 2.67E-22 |
| MBTPS2 | AC016831.4 | 0.402924 | 2.66E-22 |
| FOXO3 | AC010536.2 | 0.40296 | 2.64E-22 |
| BIRC6 | AL138756.1 | 0.40296 | 2.64E-22 |
| KIF5B | AL049840.5 | 0.40299 | 2.62E-22 |
| BIRC6 | HCG11 | 0.402998 | 2.61E-22 |
| RAB24 | AL022328.1 | 0.403016 | 2.60E-22 |
| UVRAG | AC007878.1 | 0.403021 | 2.60E-22 |
| GOPC | NFYC-AS1 | 0.403027 | 2.59E-22 |
| RAB33B | AC025917.1 | 0.403041 | 2.58E-22 |
| KLHL24 | NUTM2A-AS1 | 0.403044 | 2.58E-22 |
| ATG2B | AC109347.2 | 0.403091 | 2.55E-22 |
| ATG4B | AL118558.3 | 0.403181 | 2.49E-22 |
| ATG7 | CR936218.1 | 0.403235 | 2.46E-22 |
| UVRAG | AL049869.3 | 0.403253 | 2.45E-22 |
| TSC1 | AC026202.2 | 0.403256 | 2.44E-22 |
| NLRC4 | AC116366.1 | 0.403265 | 2.44E-22 |
| PIK3C3 | AP003486.1 | 0.403266 | 2.44E-22 |
| PELP1 | AC073896.4 | 0.403271 | 2.44E-22 |
| FOXO3 | AL662844.3 | 0.403275 | 2.43E-22 |
| ATG16L2 | AC139100.2 | 0.403302 | 2.42E-22 |
| GOPC | LINC00513 | 0.403304 | 2.41E-22 |
| ATG16L2 | AL031670.1 | 0.403307 | 2.41E-22 |
| EDEM1 | LINC00513 | 0.403316 | 2.41E-22 |
| RPS6KB1 | AC004596.1 | 0.403316 | 2.41E-22 |
| KIF5B | MCM3AP-AS1 | 0.403318 | 2.41E-22 |
| RB1CC1 | AC097103.2 | 0.403354 | 2.38E-22 |
| UVRAG | AL049552.1 | 0.403373 | 2.37E-22 |
| RB1CC1 | AC016727.1 | 0.403387 | 2.36E-22 |
| EIF2AK2 | AL121772.3 | 0.403409 | 2.35E-22 |
| KLHL24 | AC024060.1 | 0.403415 | 2.35E-22 |
| ATG4B | SNHG10 | 0.403418 | 2.34E-22 |
| EIF2AK2 | AC004253.1 | 0.403435 | 2.33E-22 |
| RAB5A | OIP5-AS1 | 0.403442 | 2.33E-22 |
| RB1CC1 | AC011477.3 | 0.403443 | 2.33E-22 |
| RB1CC1 | PSMA3-AS1 | 0.40347 | 2.31E-22 |
| EIF2AK2 | AC080013.4 | 0.403474 | 2.31E-22 |
| UVRAG | AC005070.3 | 0.403481 | 2.31E-22 |
| RB1 | AL592148.3 | 0.40356 | 2.26E-22 |
| TSC1 | AC020907.4 | 0.403561 | 2.26E-22 |
| ATG2B | AC025165.5 | 0.403573 | 2.25E-22 |
| RB1 | AC006378.1 | 0.403578 | 2.25E-22 |
| NAF1 | AC097641.2 | 0.403579 | 2.25E-22 |
| GOPC | AC018638.7 | 0.403606 | 2.23E-22 |
| RPS6KB1 | RAB30-AS1 | 0.40362 | 2.22E-22 |
| CFLAR | ACBD3-AS1 | 0.403622 | 2.22E-22 |
| RPS6KB1 | AL512791.1 | 0.40364 | 2.21E-22 |
| FOXO1 | AC008035.1 | 0.403645 | 2.21E-22 |
| CFLAR | RPS6KA2-IT1 | 0.403655 | 2.20E-22 |
| IFNG | AC005332.4 | 0.403667 | 2.20E-22 |
| EIF2AK2 | AC025287.3 | 0.403679 | 2.19E-22 |
| FOXO3 | AC090579.1 | 0.40372 | 2.17E-22 |
| ATG7 | AC100830.2 | 0.40373 | 2.16E-22 |
| ATG7 | AC087392.1 | 0.403742 | 2.16E-22 |
| ATG2B | AC083900.1 | 0.403747 | 2.15E-22 |
| KIF5B | AC004884.2 | 0.403764 | 2.14E-22 |
| RB1CC1 | ACTA2-AS1 | 0.403788 | 2.13E-22 |
| ATG4D | ARRDC1-AS1 | 0.403789 | 2.13E-22 |
| UVRAG | AC080162.1 | 0.40379 | 2.13E-22 |
| KLHL24 | THUMPD3-AS1 | 0.403797 | 2.12E-22 |
| CASP8 | AC116366.1 | 0.403811 | 2.12E-22 |
| GOPC | AC107027.3 | 0.403819 | 2.11E-22 |
| WDFY3 | JPX | 0.403836 | 2.10E-22 |
| FOXO3 | AC005632.2 | 0.403837 | 2.10E-22 |
| WDFY3 | HMGA1P4 | 0.403842 | 2.10E-22 |
| MAPK8 | AC124319.1 | 0.403851 | 2.10E-22 |
| ULK3 | LINC00265 | 0.40386 | 2.09E-22 |
| ERN1 | AL157786.1 | 0.403898 | 2.07E-22 |
| KIF5B | KLF7-IT1 | 0.403916 | 2.06E-22 |
| UVRAG | AL138963.1 | 0.403919 | 2.06E-22 |
| FKBP1B | UBL7-AS1 | 0.403946 | 2.04E-22 |
| GOPC | AC002044.1 | 0.403954 | 2.04E-22 |
| TSC1 | AC007552.2 | 0.403959 | 2.04E-22 |
| RAB33B | AC138932.5 | 0.403966 | 2.03E-22 |
| FOXO1 | MIR181A2HG | 0.403973 | 2.03E-22 |
| ULK3 | AC010973.2 | 0.403976 | 2.03E-22 |
| WDFY3 | AGBL5-IT1 | 0.403979 | 2.03E-22 |
| EDEM1 | AC096921.2 | 0.403986 | 2.02E-22 |
| RAB24 | AP001453.3 | 0.404009 | 2.01E-22 |
| TSC1 | AC124016.2 | 0.404026 | 2.00E-22 |
| ATG4B | AC004148.2 | 0.404058 | 1.99E-22 |
| SPNS1 | AC092803.2 | 0.404059 | 1.99E-22 |
| CAPN10 | LINC01770 | 0.404066 | 1.98E-22 |
| ULK3 | LINC00174 | 0.404081 | 1.97E-22 |
| MAPK8 | LINC00861 | 0.404092 | 1.97E-22 |
| MBTPS2 | AC080162.1 | 0.404155 | 1.94E-22 |
| RAB33B | AL513365.2 | 0.404168 | 1.93E-22 |
| RB1CC1 | AC069023.1 | 0.404173 | 1.93E-22 |
| ATG12 | AC139795.2 | 0.404194 | 1.92E-22 |
| WDFY3 | AP000254.1 | 0.404203 | 1.91E-22 |
| RB1CC1 | AC018809.2 | 0.404253 | 1.89E-22 |
| EEF2K | AC234775.3 | 0.40426 | 1.88E-22 |
| ATG7 | LINC00973 | 0.404263 | 1.88E-22 |
| PTEN | UBE2Q1-AS1 | 0.404273 | 1.88E-22 |
| KLHL24 | AC139887.2 | 0.404284 | 1.87E-22 |
| FOXO1 | AL138921.2 | 0.404297 | 1.87E-22 |
| RB1CC1 | AC007566.1 | 0.404318 | 1.86E-22 |
| SH3GLB1 | AL133243.2 | 0.404366 | 1.83E-22 |
| UVRAG | AL355075.2 | 0.404366 | 1.83E-22 |
| RB1 | AC009948.1 | 0.404367 | 1.83E-22 |
| BIRC6 | AC005476.2 | 0.404388 | 1.82E-22 |
| EIF2AK2 | AC092279.1 | 0.404398 | 1.82E-22 |
| PIK3C3 | AL357060.1 | 0.404414 | 1.81E-22 |
| RB1 | AC124319.2 | 0.404437 | 1.80E-22 |
| ATG2B | HOXB-AS2 | 0.404448 | 1.79E-22 |
| SPNS1 | ASMTL-AS1 | 0.404481 | 1.78E-22 |
| UVRAG | AC138932.5 | 0.404482 | 1.78E-22 |
| ATG7 | AC073487.1 | 0.404553 | 1.75E-22 |
| ATG16L2 | AC084824.5 | 0.404565 | 1.74E-22 |
| PEA15 | LINC01150 | 0.404567 | 1.74E-22 |
| SPHK1 | LINC00973 | 0.40457 | 1.74E-22 |
| MBTPS2 | AC006059.1 | 0.404618 | 1.72E-22 |
| ATF6 | AC073046.1 | 0.404621 | 1.71E-22 |
| ATG12 | AC093726.2 | 0.404622 | 1.71E-22 |
| UVRAG | AC009032.1 | 0.4047 | 1.68E-22 |
| FOXO3 | AC104695.3 | 0.404712 | 1.67E-22 |
| KIF5B | AP005899.1 | 0.404723 | 1.67E-22 |
| CXCR4 | AC004585.1 | 0.404737 | 1.66E-22 |
| PTEN | AC004596.1 | 0.404737 | 1.66E-22 |
| PTEN | SNHG16 | 0.404767 | 1.65E-22 |
| KLHL24 | AC007991.2 | 0.404776 | 1.65E-22 |
| RB1 | AC011815.1 | 0.404815 | 1.63E-22 |
| FOXO3 | MALAT1 | 0.404836 | 1.62E-22 |
| PIK3C3 | UGDH-AS1 | 0.404899 | 1.59E-22 |
| MAPK8 | AC073655.2 | 0.404918 | 1.59E-22 |
| ARSA | AL022328.4 | 0.404933 | 1.58E-22 |
| ATG16L2 | AC008982.2 | 0.404952 | 1.57E-22 |
| RPS6KB1 | ZNF32-AS2 | 0.404963 | 1.57E-22 |
| KIF5B | AL109614.1 | 0.404985 | 1.56E-22 |
| FOXO1 | AC096992.2 | 0.405005 | 1.55E-22 |
| TSC1 | AC011939.2 | 0.405009 | 1.55E-22 |
| FAS | MIR100HG | 0.405018 | 1.55E-22 |
| PIK3C3 | AC138207.4 | 0.405043 | 1.54E-22 |
| ULK2 | AC024075.1 | 0.405053 | 1.53E-22 |
| ATG12 | AL117379.1 | 0.405066 | 1.53E-22 |
| TSC1 | AC093799.1 | 0.405073 | 1.52E-22 |
| RPS6KB1 | AC124312.2 | 0.405073 | 1.52E-22 |
| TSC1 | AC087741.1 | 0.405092 | 1.52E-22 |
| GNAI3 | ABALON | 0.405126 | 1.50E-22 |
| CCR2 | LINC01857 | 0.405145 | 1.50E-22 |
| ATG7 | AL359962.2 | 0.405145 | 1.50E-22 |
| HGS | AC132872.1 | 0.405157 | 1.49E-22 |
| KIF5B | CR936218.1 | 0.405163 | 1.49E-22 |
| UVRAG | AC012170.2 | 0.405197 | 1.48E-22 |
| GOPC | NUTM2A-AS1 | 0.405217 | 1.47E-22 |
| ATG2B | LINC02398 | 0.405217 | 1.47E-22 |
| PRKAR1A | AC092611.2 | 0.40522 | 1.47E-22 |
| ATG16L2 | AP003717.1 | 0.405242 | 1.46E-22 |
| KIF5B | AP001381.1 | 0.405254 | 1.45E-22 |
| RPS6KB1 | KCCAT333 | 0.405255 | 1.45E-22 |
| RB1CC1 | AP003392.1 | 0.405268 | 1.45E-22 |
| KLHL24 | IPO5P1 | 0.405271 | 1.45E-22 |
| KLHL24 | INE1 | 0.40529 | 1.44E-22 |
| MAPK8 | GABPB1-AS1 | 0.405296 | 1.44E-22 |
| ATG4B | AL136295.2 | 0.405303 | 1.44E-22 |
| EEF2K | AC135050.6 | 0.405338 | 1.42E-22 |
| CCR2 | AC138207.5 | 0.405338 | 1.42E-22 |
| ATG2B | AL122010.1 | 0.40537 | 1.41E-22 |
| CFLAR | AL157838.1 | 0.405374 | 1.41E-22 |
| MLST8 | AP002387.1 | 0.405376 | 1.41E-22 |
| SPNS1 | AC027796.4 | 0.405379 | 1.41E-22 |
| RAB33B | DLEU2 | 0.405386 | 1.40E-22 |
| NAF1 | LINC00861 | 0.405396 | 1.40E-22 |
| CFLAR | AP002840.2 | 0.405404 | 1.40E-22 |
| FOXO1 | STARD4-AS1 | 0.405415 | 1.39E-22 |
| ERN1 | AC022400.5 | 0.405419 | 1.39E-22 |
| PIK3C3 | AC092611.2 | 0.405422 | 1.39E-22 |
| FOXO3 | AL513550.1 | 0.405454 | 1.38E-22 |
| ATG7 | FGD5-AS1 | 0.405457 | 1.38E-22 |
| IFNG | HCP5 | 0.405477 | 1.37E-22 |
| EIF2AK2 | PSMA3-AS1 | 0.405486 | 1.37E-22 |
| RPS6KB1 | AC005519.1 | 0.405494 | 1.37E-22 |
| FOXO1 | ARHGEF38-IT1 | 0.40551 | 1.36E-22 |
| EIF2AK2 | AL078581.1 | 0.405527 | 1.35E-22 |
| TSC1 | AC016957.2 | 0.405539 | 1.35E-22 |
| RPS6KB1 | ACTA2-AS1 | 0.405544 | 1.35E-22 |
| RAB5A | AC141002.1 | 0.405602 | 1.33E-22 |
| ERN1 | AC135050.5 | 0.405609 | 1.33E-22 |
| CFLAR | AC004884.2 | 0.405609 | 1.33E-22 |
| RB1 | AC007552.2 | 0.405617 | 1.32E-22 |
| TSC1 | LINC01176 | 0.40563 | 1.32E-22 |
| STK11 | AL031600.1 | 0.405638 | 1.32E-22 |
| RAB33B | AL133243.2 | 0.405641 | 1.31E-22 |
| KIF5B | AC007878.1 | 0.405672 | 1.30E-22 |
| KIF5B | AF178030.1 | 0.405707 | 1.29E-22 |
| PIK3C3 | AC073534.1 | 0.405708 | 1.29E-22 |
| ATG16L2 | NARF-IT1 | 0.405728 | 1.28E-22 |
| MLST8 | SNHG9 | 0.405736 | 1.28E-22 |
| CFLAR | AC002044.1 | 0.40574 | 1.28E-22 |
| MAPK8 | AC010615.2 | 0.405749 | 1.28E-22 |
| WDFY3 | HCG11 | 0.405757 | 1.28E-22 |
| FOXO3 | AC025287.3 | 0.405786 | 1.27E-22 |
| NLRC4 | TRG-AS1 | 0.405792 | 1.26E-22 |
| TSC1 | AC025171.3 | 0.405794 | 1.26E-22 |
| RB1CC1 | AL354696.1 | 0.405797 | 1.26E-22 |
| MBTPS2 | LINC00216 | 0.405827 | 1.25E-22 |
| PIK3R4 | AC090425.2 | 0.405903 | 1.23E-22 |
| UVRAG | AC110792.3 | 0.405911 | 1.22E-22 |
| ATG2B | AC009948.4 | 0.405927 | 1.22E-22 |
| NLRC4 | AL133330.1 | 0.405938 | 1.22E-22 |
| TSC1 | AP002907.1 | 0.40594 | 1.22E-22 |
| KLHL24 | AL133371.2 | 0.405948 | 1.21E-22 |
| RB1 | PCBP1-AS1 | 0.40595 | 1.21E-22 |
| FOXO1 | AC138956.2 | 0.405954 | 1.21E-22 |
| TSC1 | AC114730.3 | 0.405963 | 1.21E-22 |
| RPS6KB1 | AL109761.1 | 0.405976 | 1.20E-22 |
| ATG12 | THAP9-AS1 | 0.405977 | 1.20E-22 |
| EIF2AK2 | AC026355.1 | 0.405986 | 1.20E-22 |
| TSC1 | BTBD9-AS1 | 0.406003 | 1.20E-22 |
| TSC1 | AP002336.2 | 0.406015 | 1.19E-22 |
| MBTPS2 | AP001178.2 | 0.406059 | 1.18E-22 |
| PEA15 | AC090559.1 | 0.406068 | 1.18E-22 |
| ATG4B | AC012360.3 | 0.406081 | 1.17E-22 |
| FOXO3 | AC068790.3 | 0.406106 | 1.16E-22 |
| TSC1 | AC016394.1 | 0.406106 | 1.16E-22 |
| EEF2K | AC093495.1 | 0.40611 | 1.16E-22 |
| KIF5B | SNHG16 | 0.40612 | 1.16E-22 |
| ATG5 | LINC01094 | 0.406126 | 1.16E-22 |
| FOXO1 | LINC01655 | 0.406169 | 1.14E-22 |
| CAPN10 | AC005696.1 | 0.406173 | 1.14E-22 |
| ATG16L2 | AC025287.3 | 0.406199 | 1.14E-22 |
| BIRC6 | AC004908.1 | 0.406218 | 1.13E-22 |
| FOXO3 | AL133330.1 | 0.406233 | 1.13E-22 |
| ATG2B | AC079921.2 | 0.406248 | 1.12E-22 |
| NAF1 | AC087481.3 | 0.406276 | 1.11E-22 |
| ATG12 | AC011374.2 | 0.406285 | 1.11E-22 |
| TSC2 | AC245060.2 | 0.406302 | 1.11E-22 |
| KIF5B | AC005070.3 | 0.40633 | 1.10E-22 |
| BIRC5 | LINC00511 | 0.406337 | 1.10E-22 |
| KLHL24 | AC009948.1 | 0.406344 | 1.09E-22 |
| GOPC | AL035409.1 | 0.406358 | 1.09E-22 |
| GOPC | SNHG14 | 0.406406 | 1.08E-22 |
| RAB33B | FTX | 0.406415 | 1.07E-22 |
| DAPK2 | AP000254.1 | 0.406415 | 1.07E-22 |
| NAF1 | C1RL-AS1 | 0.406416 | 1.07E-22 |
| EEF2K | AC018809.2 | 0.406431 | 1.07E-22 |
| GOPC | AP005899.1 | 0.406452 | 1.06E-22 |
| BIRC6 | AC010761.1 | 0.406494 | 1.05E-22 |
| RB1CC1 | NFYC-AS1 | 0.406499 | 1.05E-22 |
| IFNG | AC090948.2 | 0.406509 | 1.05E-22 |
| RPS6KB1 | AC025287.3 | 0.406515 | 1.05E-22 |
| ATG16L2 | AL021392.1 | 0.406567 | 1.03E-22 |
| RB1 | AC027097.1 | 0.406577 | 1.03E-22 |
| RB1 | ANKRD10-IT1 | 0.406613 | 1.02E-22 |
| RAB33B | AC006059.1 | 0.406631 | 1.01E-22 |
| ATG2B | AC244517.7 | 0.40664 | 1.01E-22 |
| RB1CC1 | KDM4A-AS1 | 0.406653 | 1.01E-22 |
| RPS6KB1 | GAS8-AS1 | 0.406654 | 1.01E-22 |
| ULK3 | AL022328.1 | 0.406684 | 1.00E-22 |
| FOXO1 | AC093278.2 | 0.406686 | 1.00E-22 |
| RAB24 | AL135999.1 | 0.406688 | 9.99E-23 |
| FOXO1 | AL354989.1 | 0.406694 | 9.98E-23 |
| CAPN10 | LINC00685 | 0.406719 | 9.91E-23 |
| FOXO1 | AC018809.2 | 0.406731 | 9.88E-23 |
| KIF5B | RHOA-IT1 | 0.406767 | 9.79E-23 |
| PARP1 | AL928654.1 | 0.406869 | 9.53E-23 |
| SPHK1 | AP000695.1 | 0.406875 | 9.52E-23 |
| RB1 | AC078846.1 | 0.4069 | 9.45E-23 |
| UVRAG | AL359076.1 | 0.406924 | 9.39E-23 |
| NAF1 | MUC20-OT1 | 0.40693 | 9.38E-23 |
| RAB24 | AC012645.3 | 0.406953 | 9.32E-23 |
| ATG16L2 | AC068580.3 | 0.406954 | 9.32E-23 |
| RAB24 | ZNF213-AS1 | 0.406963 | 9.30E-23 |
| PIK3C3 | AF131215.5 | 0.406979 | 9.26E-23 |
| EIF2AK2 | ZKSCAN2-DT | 0.407037 | 9.12E-23 |
| TSC2 | MZF1-AS1 | 0.407092 | 8.99E-23 |
| SH3GLB1 | AC006059.1 | 0.40711 | 8.95E-23 |
| ATG12 | AC008969.1 | 0.407113 | 8.94E-23 |
| RB1CC1 | AL136531.1 | 0.407126 | 8.91E-23 |
| RB1 | HIF1A-AS2 | 0.407135 | 8.89E-23 |
| FOXO3 | AC068790.2 | 0.407151 | 8.85E-23 |
| GOPC | AC141002.1 | 0.40719 | 8.76E-23 |
| EEF2K | AC008764.2 | 0.407194 | 8.75E-23 |
| NAF1 | AC087222.1 | 0.407201 | 8.74E-23 |
| TSC1 | DNM3OS | 0.407201 | 8.74E-23 |
| NLRC4 | GK-IT1 | 0.407203 | 8.73E-23 |
| ATG7 | AC025917.1 | 0.407216 | 8.70E-23 |
| RAB33B | MIR181A2HG | 0.407228 | 8.67E-23 |
| EIF4EBP1 | MIR193BHG | 0.407271 | 8.58E-23 |
| MBTPS2 | NUTM2B-AS1 | 0.407284 | 8.55E-23 |
| CAPN10 | AC107375.1 | 0.407288 | 8.54E-23 |
| RB1CC1 | AC007849.1 | 0.40729 | 8.53E-23 |
| RB1 | LINC02352 | 0.407326 | 8.45E-23 |
| EDEM1 | LINC02257 | 0.407375 | 8.35E-23 |
| WDFY3 | AC012368.1 | 0.407381 | 8.33E-23 |
| RPTOR | ST8SIA6-AS1 | 0.407429 | 8.23E-23 |
| MAP2K7 | AC012615.6 | 0.40745 | 8.18E-23 |
| EIF2AK2 | AL158166.1 | 0.407493 | 8.09E-23 |
| GOPC | AC006270.1 | 0.407493 | 8.09E-23 |
| RB1 | AC074033.1 | 0.407508 | 8.06E-23 |
| CXCR4 | FAM30A | 0.407531 | 8.01E-23 |
| KLHL24 | AL117379.1 | 0.407539 | 7.99E-23 |
| TSC1 | AP000240.1 | 0.407541 | 7.99E-23 |
| RAB24 | LINC00893 | 0.407553 | 7.96E-23 |
| RB1 | AC084871.1 | 0.407574 | 7.92E-23 |
| RB1 | AC008966.2 | 0.407612 | 7.84E-23 |
| KLHL24 | LINC00861 | 0.407626 | 7.81E-23 |
| GOPC | AC027277.2 | 0.40766 | 7.74E-23 |
| ATG12 | AC025178.1 | 0.407661 | 7.74E-23 |
| BIRC6 | JPX | 0.407671 | 7.72E-23 |
| SPHK1 | AP000695.2 | 0.407674 | 7.72E-23 |
| WDFY3 | ASH1L-AS1 | 0.407686 | 7.69E-23 |
| ERN1 | AC024075.3 | 0.407687 | 7.69E-23 |
| BNIP1 | CYTOR | 0.407696 | 7.67E-23 |
| KLHL24 | AP003486.1 | 0.407729 | 7.60E-23 |
| TSC1 | KIF26B-AS1 | 0.407736 | 7.59E-23 |
| GOPC | OCIAD1-AS1 | 0.407739 | 7.58E-23 |
| TSC1 | AC022150.2 | 0.407752 | 7.56E-23 |
| GOPC | ARMCX5-GPRASP2 | 0.407767 | 7.53E-23 |
| RB1CC1 | AC074033.1 | 0.407783 | 7.50E-23 |
| UVRAG | AC087276.1 | 0.407789 | 7.48E-23 |
| ATG7 | AC016590.2 | 0.407793 | 7.48E-23 |
| FOXO1 | AP001432.1 | 0.407829 | 7.41E-23 |
| FOXO3 | AC067852.3 | 0.407832 | 7.40E-23 |
| GOPC | AF131215.5 | 0.407836 | 7.39E-23 |
| BIRC6 | AC004982.1 | 0.407839 | 7.39E-23 |
| RB1CC1 | PWAR6 | 0.407845 | 7.38E-23 |
| EIF2AK2 | AL138921.2 | 0.407848 | 7.37E-23 |
| ARSB | LINC01094 | 0.407867 | 7.33E-23 |
| GOPC | AC037198.2 | 0.407884 | 7.30E-23 |
| RAB24 | AC020907.4 | 0.407895 | 7.28E-23 |
| RB1 | AL021578.1 | 0.407897 | 7.28E-23 |
| RB1 | AC037198.1 | 0.407961 | 7.15E-23 |
| UVRAG | ANKRD44-IT1 | 0.40797 | 7.14E-23 |
| ATG7 | AC090559.1 | 0.40798 | 7.12E-23 |
| KLHL24 | AC005479.1 | 0.407987 | 7.10E-23 |
| CAPN10 | AC018653.3 | 0.408002 | 7.08E-23 |
| CFLAR | AC048382.2 | 0.408009 | 7.06E-23 |
| MAPK8 | AL512791.1 | 0.408055 | 6.98E-23 |
| GOPC | MAGI2-AS3 | 0.408075 | 6.94E-23 |
| TSC1 | AF131215.6 | 0.408078 | 6.94E-23 |
| BIRC6 | AL049840.4 | 0.408078 | 6.94E-23 |
| ATG12 | AC093484.4 | 0.408116 | 6.87E-23 |
| FOXO1 | SNHG14 | 0.408124 | 6.85E-23 |
| CTSB | AC147651.3 | 0.408125 | 6.85E-23 |
| RB1 | AC005021.1 | 0.408148 | 6.81E-23 |
| FOXO1 | AC093495.1 | 0.408157 | 6.79E-23 |
| ULK3 | PRKCZ-AS1 | 0.40816 | 6.79E-23 |
| TSC1 | AC004656.1 | 0.408174 | 6.76E-23 |
| ULK3 | AP001453.3 | 0.408188 | 6.74E-23 |
| RPS6KB1 | SNHG14 | 0.408194 | 6.73E-23 |
| EIF2AK2 | LINC02352 | 0.408222 | 6.68E-23 |
| EEF2K | MCCC1-AS1 | 0.408226 | 6.67E-23 |
| CASP1 | LINC01871 | 0.408227 | 6.67E-23 |
| EIF2AK2 | AL159169.2 | 0.408236 | 6.65E-23 |
| PIK3C3 | AC087752.4 | 0.408242 | 6.64E-23 |
| CFLAR | AC060780.1 | 0.408245 | 6.64E-23 |
| KIF5B | AC068790.2 | 0.408256 | 6.62E-23 |
| SIRT1 | NFYC-AS1 | 0.408261 | 6.61E-23 |
| CASP4 | LINC01094 | 0.408261 | 6.61E-23 |
| CFLAR | AL359697.1 | 0.408273 | 6.59E-23 |
| NFE2L2 | AC022075.1 | 0.408275 | 6.59E-23 |
| UVRAG | AL157392.3 | 0.408275 | 6.59E-23 |
| UVRAG | PPP3CB-AS1 | 0.408341 | 6.47E-23 |
| CFLAR | HLA-F-AS1 | 0.408344 | 6.47E-23 |
| SIRT1 | NUTM2A-AS1 | 0.408399 | 6.37E-23 |
| TSC2 | AC084018.1 | 0.408429 | 6.32E-23 |
| FOXO1 | AL139011.1 | 0.408441 | 6.30E-23 |
| DLC1 | AC002563.1 | 0.408459 | 6.27E-23 |
| BIRC6 | AGBL5-IT1 | 0.408468 | 6.26E-23 |
| KIF5B | AC234775.3 | 0.408473 | 6.25E-23 |
| GOPC | AP001458.1 | 0.408481 | 6.24E-23 |
| GABARAPL2 | LINC00324 | 0.408481 | 6.24E-23 |
| RB1 | AC133644.2 | 0.408501 | 6.21E-23 |
| RPS6KB1 | AC124312.4 | 0.408506 | 6.20E-23 |
| ATG2B | RAD51-AS1 | 0.408517 | 6.18E-23 |
| TSC1 | AC009318.3 | 0.408526 | 6.16E-23 |
| PIK3C3 | AC087222.1 | 0.408535 | 6.15E-23 |
| FOXO1 | AC242426.2 | 0.408561 | 6.11E-23 |
| EIF2AK2 | AC018809.2 | 0.408577 | 6.08E-23 |
| CFLAR | AC244197.2 | 0.408578 | 6.08E-23 |
| PTEN | AC103591.3 | 0.408582 | 6.07E-23 |
| RPS6KB1 | AGAP1-IT1 | 0.40859 | 6.06E-23 |
| CFLAR | AC055822.1 | 0.408603 | 6.04E-23 |
| FOXO1 | FMR1-IT1 | 0.408608 | 6.03E-23 |
| RPS6KB1 | AC093297.2 | 0.408643 | 5.98E-23 |
| CFLAR | AC060766.7 | 0.408721 | 5.85E-23 |
| EEF2K | ZKSCAN2-DT | 0.408723 | 5.85E-23 |
| KLHL24 | AC138956.2 | 0.408745 | 5.82E-23 |
| NLRC4 | AC112496.1 | 0.408745 | 5.82E-23 |
| ATG7 | PCED1B-AS1 | 0.408757 | 5.80E-23 |
| BIRC6 | AC137894.1 | 0.408769 | 5.78E-23 |
| GOPC | AL021707.7 | 0.408843 | 5.67E-23 |
| DAPK2 | AC090589.3 | 0.408851 | 5.66E-23 |
| MAPK8 | AC024060.1 | 0.408855 | 5.65E-23 |
| FOXO3 | AC012181.1 | 0.408862 | 5.64E-23 |
| UVRAG | AC098851.1 | 0.408915 | 5.56E-23 |
| BCL2 | STARD4-AS1 | 0.408928 | 5.54E-23 |
| RAB33B | AF129075.1 | 0.408956 | 5.50E-23 |
| ATG2B | GK-IT1 | 0.408959 | 5.50E-23 |
| GABARAPL2 | AC025048.4 | 0.408968 | 5.49E-23 |
| SPNS1 | AC073335.2 | 0.408977 | 5.47E-23 |
| CFLAR | AL021878.2 | 0.408986 | 5.46E-23 |
| MAP1LC3C | AL137793.1 | 0.409034 | 5.39E-23 |
| CFLAR | AC079684.1 | 0.409038 | 5.38E-23 |
| PIK3R4 | AP001432.1 | 0.409043 | 5.38E-23 |
| FOXO1 | MAST4-AS1 | 0.409047 | 5.37E-23 |
| KIF5B | ACAP2-IT1 | 0.40906 | 5.35E-23 |
| ATG12 | DGCR11 | 0.409091 | 5.31E-23 |
| FOXO1 | AC006378.1 | 0.409112 | 5.28E-23 |
| UVRAG | MALAT1 | 0.409134 | 5.25E-23 |
| TSC2 | LINC01569 | 0.40915 | 5.23E-23 |
| ATG4B | FLJ37453 | 0.409152 | 5.23E-23 |
| KIF5B | AC087286.2 | 0.409157 | 5.22E-23 |
| UVRAG | ACAP2-IT1 | 0.409161 | 5.21E-23 |
| RPS6KB1 | AC005261.1 | 0.409161 | 5.21E-23 |
| RGS19 | LINC01150 | 0.409172 | 5.20E-23 |
| CFLAR | AC078778.1 | 0.409226 | 5.12E-23 |
| BIRC6 | AC244517.7 | 0.40923 | 5.12E-23 |
| TSC2 | CCDC183-AS1 | 0.409238 | 5.11E-23 |
| GOPC | AC078778.1 | 0.409301 | 5.02E-23 |
| UVRAG | AC008906.1 | 0.40933 | 4.99E-23 |
| RAB33B | AC018797.2 | 0.409348 | 4.96E-23 |
| RPS6KB1 | STARD4-AS1 | 0.409382 | 4.92E-23 |
| FAS | AL357060.1 | 0.409388 | 4.91E-23 |
| RB1 | AC026355.1 | 0.409406 | 4.89E-23 |
| EIF2AK2 | AC048341.2 | 0.409455 | 4.82E-23 |
| ATG7 | AL022067.1 | 0.409474 | 4.80E-23 |
| BIRC5 | AL391069.2 | 0.409499 | 4.77E-23 |
| EDEM1 | RHOA-IT1 | 0.409521 | 4.74E-23 |
| KLHL24 | AC093227.1 | 0.409526 | 4.73E-23 |
| RAB24 | AC104564.3 | 0.409536 | 4.72E-23 |
| WDFY3 | AL135818.1 | 0.409538 | 4.72E-23 |
| ATG7 | AC048344.4 | 0.409563 | 4.69E-23 |
| ATG7 | AL157932.1 | 0.409573 | 4.67E-23 |
| TSC1 | AL035563.1 | 0.409595 | 4.65E-23 |
| UVRAG | LINC00216 | 0.409605 | 4.63E-23 |
| CAPN10 | SSSCA1-AS1 | 0.409616 | 4.62E-23 |
| PTEN | AC109347.2 | 0.409626 | 4.61E-23 |
| ATG7 | N4BP2L2-IT2 | 0.409635 | 4.60E-23 |
| KIF5B | LINC01376 | 0.409642 | 4.59E-23 |
| ATG7 | AC005540.1 | 0.409678 | 4.55E-23 |
| MBTPS2 | AC010186.3 | 0.409688 | 4.53E-23 |
| ATG16L2 | MHENCR | 0.409706 | 4.51E-23 |
| WDFY3 | AP001271.1 | 0.409707 | 4.51E-23 |
| MBTPS2 | ANKRD44-IT1 | 0.409724 | 4.49E-23 |
| RPS6KB1 | MIR181A2HG | 0.409735 | 4.48E-23 |
| RAB24 | AL109811.3 | 0.40974 | 4.47E-23 |
| SIRT1 | MCM3AP-AS1 | 0.409744 | 4.47E-23 |
| TSC1 | AC005046.1 | 0.409809 | 4.39E-23 |
| PTEN | AL159169.2 | 0.409833 | 4.36E-23 |
| RHEB | AC012640.2 | 0.409841 | 4.35E-23 |
| TSC1 | AC074033.1 | 0.409873 | 4.32E-23 |
| ATG4B | AC136475.2 | 0.409881 | 4.31E-23 |
| KLHL24 | AC133644.2 | 0.409883 | 4.31E-23 |
| HGS | AC069281.2 | 0.409918 | 4.27E-23 |
| RAB5A | AC087286.2 | 0.409958 | 4.22E-23 |
| RPS6KB1 | AC010226.1 | 0.409972 | 4.21E-23 |
| EIF2AK2 | RAB30-AS1 | 0.40998 | 4.20E-23 |
| CFLAR | LINC02352 | 0.409992 | 4.18E-23 |
| ATG7 | AL357992.1 | 0.409998 | 4.18E-23 |
| CAPN10 | AP006621.4 | 0.410038 | 4.13E-23 |
| PIK3C3 | MIR222HG | 0.41004 | 4.13E-23 |
| KIF5B | AL136115.2 | 0.41008 | 4.09E-23 |
| KLHL24 | GEMIN7-AS1 | 0.410107 | 4.06E-23 |
| BIRC6 | AC008014.1 | 0.410112 | 4.05E-23 |
| MAPK8 | AL157932.1 | 0.410139 | 4.02E-23 |
| EIF2AK2 | AC113139.1 | 0.410141 | 4.02E-23 |
| RAC1 | AC004130.1 | 0.410175 | 3.99E-23 |
| GOPC | AC007319.1 | 0.410223 | 3.93E-23 |
| ATG7 | AC004223.3 | 0.410223 | 3.93E-23 |
| RAB33B | ARMCX5-GPRASP2 | 0.410224 | 3.93E-23 |
| CFLAR | AL135818.1 | 0.410235 | 3.92E-23 |
| ATG7 | AC007216.3 | 0.410235 | 3.92E-23 |
| IFNG | AC147651.3 | 0.410249 | 3.91E-23 |
| DAPK2 | RPARP-AS1 | 0.410254 | 3.90E-23 |
| FOXO3 | LINC00630 | 0.410257 | 3.90E-23 |
| RB1 | AL133371.2 | 0.410262 | 3.89E-23 |
| EIF2AK2 | LINC01578 | 0.410273 | 3.88E-23 |
| ATG4B | AC093249.6 | 0.410296 | 3.86E-23 |
| RAB5A | AC096741.1 | 0.410306 | 3.85E-23 |
| CAPN10 | AL136295.7 | 0.410318 | 3.84E-23 |
| PIK3R4 | AC005288.1 | 0.410327 | 3.83E-23 |
| TSC1 | BACE1-AS | 0.410331 | 3.82E-23 |
| BIRC6 | AL121584.1 | 0.410347 | 3.81E-23 |
| PIK3C3 | MCCC1-AS1 | 0.410421 | 3.73E-23 |
| FOXO3 | AC130650.2 | 0.410422 | 3.73E-23 |
| ATG7 | AC068790.3 | 0.410428 | 3.73E-23 |
| UVRAG | AC018926.3 | 0.410475 | 3.68E-23 |
| NLRC4 | AC069023.1 | 0.410478 | 3.68E-23 |
| BIRC6 | AC012368.1 | 0.410487 | 3.67E-23 |
| NLRC4 | ATP1B3-AS1 | 0.410487 | 3.67E-23 |
| UVRAG | AL109614.1 | 0.410488 | 3.67E-23 |
| FOXO3 | AL355075.2 | 0.410492 | 3.66E-23 |
| PIK3C3 | LINC00909 | 0.410508 | 3.65E-23 |
| NAF1 | AL157392.4 | 0.410513 | 3.64E-23 |
| KLHL24 | AC018926.2 | 0.410514 | 3.64E-23 |
| EIF2AK2 | AC129510.1 | 0.410518 | 3.64E-23 |
| ATG7 | AC096586.2 | 0.410521 | 3.64E-23 |
| RAC1 | AC092171.2 | 0.410529 | 3.63E-23 |
| RGS19 | LINC01094 | 0.410532 | 3.63E-23 |
| KLHL24 | MAGI2-AS3 | 0.410563 | 3.60E-23 |
| CASP1 | AC025048.4 | 0.410569 | 3.59E-23 |
| KLHL24 | AC016394.1 | 0.41057 | 3.59E-23 |
| SH3GLB1 | AL035409.1 | 0.410576 | 3.58E-23 |
| FOXO3 | USP46-AS1 | 0.410585 | 3.57E-23 |
| FOXO1 | NARF-IT1 | 0.410596 | 3.56E-23 |
| TSC1 | AC106028.3 | 0.410604 | 3.56E-23 |
| RPS6KB1 | AC068792.1 | 0.410617 | 3.54E-23 |
| RB1CC1 | AC011468.5 | 0.410686 | 3.48E-23 |
| CFLAR | AC016394.1 | 0.410694 | 3.47E-23 |
| GOPC | AP003392.1 | 0.410702 | 3.46E-23 |
| ATG7 | LINC00861 | 0.41077 | 3.40E-23 |
| MBTPS2 | Z83843.1 | 0.410773 | 3.40E-23 |
| KLHL24 | ZNF529-AS1 | 0.410776 | 3.40E-23 |
| NAF1 | NARF-IT1 | 0.410782 | 3.39E-23 |
| RB1CC1 | SNHG16 | 0.410799 | 3.38E-23 |
| KLHL24 | NFYC-AS1 | 0.410808 | 3.37E-23 |
| UVRAG | NPTN-IT1 | 0.410838 | 3.34E-23 |
| RB1 | AC084824.4 | 0.410843 | 3.34E-23 |
| RB1 | AL359962.2 | 0.410855 | 3.33E-23 |
| RAB5A | AL606834.1 | 0.410863 | 3.32E-23 |
| TSC1 | AC008875.1 | 0.410914 | 3.27E-23 |
| NLRC4 | C5orf56 | 0.410923 | 3.27E-23 |
| KLHL24 | AC016957.2 | 0.410924 | 3.27E-23 |
| RB1 | AC116366.1 | 0.410942 | 3.25E-23 |
| RB1 | AL359697.1 | 0.410982 | 3.22E-23 |
| PIK3C3 | AC007249.1 | 0.410986 | 3.21E-23 |
| CAPN10 | AL118558.4 | 0.41099 | 3.21E-23 |
| MBTPS2 | AC005034.5 | 0.410991 | 3.21E-23 |
| KIF5B | AC022973.3 | 0.410999 | 3.20E-23 |
| ATG7 | AC138207.5 | 0.411009 | 3.19E-23 |
| UVRAG | AL136320.1 | 0.41101 | 3.19E-23 |
| FOXO3 | AL360219.1 | 0.411054 | 3.16E-23 |
| FOXO3 | AC068790.5 | 0.411065 | 3.15E-23 |
| ATG16L2 | AC138207.4 | 0.41109 | 3.13E-23 |
| RB1CC1 | AC135050.5 | 0.411097 | 3.12E-23 |
| KLHL24 | AL513327.1 | 0.411119 | 3.10E-23 |
| KLHL24 | AL683813.1 | 0.41112 | 3.10E-23 |
| RB1CC1 | AL512791.1 | 0.411133 | 3.09E-23 |
| TSC1 | AC058791.1 | 0.411153 | 3.07E-23 |
| TSC1 | AC027097.1 | 0.411162 | 3.07E-23 |
| PIK3C3 | AC009041.4 | 0.411226 | 3.01E-23 |
| ATG4B | PAXIP1-AS1 | 0.411234 | 3.01E-23 |
| GOPC | AC008537.2 | 0.411236 | 3.01E-23 |
| RB1CC1 | AL157932.1 | 0.411239 | 3.00E-23 |
| GOPC | AC007849.1 | 0.411244 | 3.00E-23 |
| CD46 | LINC02257 | 0.411257 | 2.99E-23 |
| TSC1 | ADAMTSL4-AS1 | 0.411267 | 2.98E-23 |
| KIF5B | LINC00216 | 0.411287 | 2.97E-23 |
| RB1CC1 | MIR222HG | 0.411297 | 2.96E-23 |
| CFLAR | AC079921.2 | 0.411317 | 2.94E-23 |
| IL24 | PCED1B-AS1 | 0.411335 | 2.93E-23 |
| ATG4B | AC133919.2 | 0.411367 | 2.90E-23 |
| MAPK8 | RFX3-AS1 | 0.411383 | 2.89E-23 |
| TSC1 | TMEM9B-AS1 | 0.411389 | 2.89E-23 |
| KLHL24 | AC009090.1 | 0.411397 | 2.88E-23 |
| MBTPS2 | AC073651.1 | 0.411403 | 2.88E-23 |
| TSC1 | STARD4-AS1 | 0.411473 | 2.82E-23 |
| ATG4B | AC103691.1 | 0.411474 | 2.82E-23 |
| MAPK8 | AP003392.1 | 0.411487 | 2.81E-23 |
| EIF2AK2 | AC087392.1 | 0.411491 | 2.81E-23 |
| KIF5B | GMDS-DT | 0.411506 | 2.80E-23 |
| GOPC | MAST4-AS1 | 0.411508 | 2.80E-23 |
| NAF1 | PSMA3-AS1 | 0.411512 | 2.79E-23 |
| CTSD | AC068580.1 | 0.411539 | 2.77E-23 |
| RB1CC1 | AL133371.2 | 0.41156 | 2.76E-23 |
| GOPC | AC074032.1 | 0.411582 | 2.74E-23 |
| ATG16L2 | AC015871.3 | 0.411586 | 2.74E-23 |
| ATG16L2 | HM13-IT1 | 0.411588 | 2.74E-23 |
| TSC1 | OCIAD1-AS1 | 0.411594 | 2.73E-23 |
| FOXO3 | AC004656.1 | 0.411623 | 2.71E-23 |
| CFLAR | AC008982.2 | 0.411654 | 2.69E-23 |
| NAF1 | AP001625.2 | 0.411659 | 2.69E-23 |
| ATG12 | AC004656.1 | 0.411715 | 2.65E-23 |
| UVRAG | ERVK13-1 | 0.411738 | 2.63E-23 |
| SIRT2 | AL121820.2 | 0.411769 | 2.61E-23 |
| DAPK2 | GLIS2-AS1 | 0.411775 | 2.60E-23 |
| TSC1 | ZNF436-AS1 | 0.411835 | 2.56E-23 |
| UVRAG | PAXBP1-AS1 | 0.411888 | 2.53E-23 |
| TSC1 | AC005632.2 | 0.41189 | 2.53E-23 |
| ATG16L2 | AL157392.4 | 0.411916 | 2.51E-23 |
| RB1CC1 | AL157392.4 | 0.411928 | 2.50E-23 |
| RB1CC1 | AC124319.1 | 0.41193 | 2.50E-23 |
| MAPK8 | LINC02035 | 0.411969 | 2.47E-23 |
| IFNG | AC012181.1 | 0.411986 | 2.46E-23 |
| RB1CC1 | AC138207.4 | 0.411994 | 2.46E-23 |
| FKBP1B | MAPKAPK5-AS1 | 0.41201 | 2.45E-23 |
| GOPC | AC092611.2 | 0.412021 | 2.44E-23 |
| UVRAG | ADNP-AS1 | 0.412033 | 2.43E-23 |
| FOXO3 | AC002064.2 | 0.412053 | 2.42E-23 |
| GOPC | USP46-AS1 | 0.412122 | 2.37E-23 |
| UVRAG | SOS1-IT1 | 0.412125 | 2.37E-23 |
| FOXO3 | AP001178.2 | 0.412131 | 2.37E-23 |
| EIF2AK2 | AC010226.1 | 0.412157 | 2.35E-23 |
| ATG7 | GK-AS1 | 0.412161 | 2.35E-23 |
| PELP1 | AC012510.1 | 0.412191 | 2.33E-23 |
| TSC1 | ASH1L-AS1 | 0.412213 | 2.32E-23 |
| MLST8 | LINC01569 | 0.41222 | 2.31E-23 |
| UVRAG | AC025287.3 | 0.412239 | 2.30E-23 |
| EIF2AK2 | AP006621.2 | 0.412256 | 2.29E-23 |
| EIF2AK2 | AC007849.1 | 0.412288 | 2.27E-23 |
| FOXO3 | DNM3OS | 0.412295 | 2.27E-23 |
| RAB33B | AC005070.3 | 0.412301 | 2.26E-23 |
| MBTPS2 | AL139407.1 | 0.412311 | 2.26E-23 |
| RB1 | AC120349.1 | 0.412316 | 2.25E-23 |
| FOXO3 | AP000866.6 | 0.412339 | 2.24E-23 |
| KIF5B | AC005288.1 | 0.41237 | 2.22E-23 |
| ATG2B | AC091057.1 | 0.412371 | 2.22E-23 |
| SH3GLB1 | MAGI2-AS3 | 0.412375 | 2.22E-23 |
| ATF6 | AC018752.1 | 0.412376 | 2.22E-23 |
| RB1 | AC037198.2 | 0.412377 | 2.22E-23 |
| GOPC | AC074033.1 | 0.412405 | 2.20E-23 |
| TSC1 | AL513327.1 | 0.412408 | 2.20E-23 |
| ATG4B | AC108488.1 | 0.412408 | 2.20E-23 |
| RAB33B | AP001432.1 | 0.412435 | 2.18E-23 |
| IKBKB | HAGLR | 0.412444 | 2.18E-23 |
| ATG7 | ANKRD44-IT1 | 0.412455 | 2.17E-23 |
| UVRAG | AC073487.1 | 0.412476 | 2.16E-23 |
| MBTPS2 | AC234775.3 | 0.412497 | 2.15E-23 |
| EIF2AK2 | AC007319.1 | 0.412503 | 2.14E-23 |
| ATG4B | AC027601.3 | 0.412518 | 2.14E-23 |
| EIF2AK2 | AL354696.1 | 0.412542 | 2.12E-23 |
| BNIP1 | AC008915.2 | 0.412553 | 2.12E-23 |
| HGS | AL645608.8 | 0.412557 | 2.11E-23 |
| MBTPS2 | NPTN-IT1 | 0.412561 | 2.11E-23 |
| SPNS1 | MMP25-AS1 | 0.41258 | 2.10E-23 |
| MBTPS2 | AC007878.1 | 0.412597 | 2.09E-23 |
| CASP1 | AC018755.4 | 0.412598 | 2.09E-23 |
| PIK3C3 | LINC01290 | 0.412606 | 2.09E-23 |
| RAB24 | LINC00115 | 0.412623 | 2.08E-23 |
| ATG7 | AC010226.1 | 0.412625 | 2.08E-23 |
| MBTPS2 | CFLAR-AS1 | 0.412635 | 2.07E-23 |
| BID | CYTOR | 0.412651 | 2.06E-23 |
| SH3GLB1 | AC087286.4 | 0.412666 | 2.05E-23 |
| PIK3C3 | AL359715.3 | 0.412678 | 2.05E-23 |
| CFLAR | CBR3-AS1 | 0.412693 | 2.04E-23 |
| CFLAR | AL159169.2 | 0.412695 | 2.04E-23 |
| ATG12 | LINC01655 | 0.412699 | 2.03E-23 |
| CAPN10 | U47924.3 | 0.4127 | 2.03E-23 |
| RB1 | ADNP-AS1 | 0.412704 | 2.03E-23 |
| UVRAG | AC004492.1 | 0.412738 | 2.01E-23 |
| STK11 | AC012615.6 | 0.412767 | 2.00E-23 |
| KIF5B | AL590723.1 | 0.412795 | 1.98E-23 |
| EIF2AK2 | AL356356.1 | 0.412805 | 1.98E-23 |
| EIF2AK2 | AL133371.2 | 0.412817 | 1.97E-23 |
| KLHL24 | AL513534.1 | 0.412821 | 1.97E-23 |
| TSC2 | AL022328.3 | 0.412823 | 1.97E-23 |
| NAF1 | AC091057.1 | 0.412834 | 1.96E-23 |
| ATG7 | TRAF3IP2-AS1 | 0.412842 | 1.96E-23 |
| MAPK8 | AC008969.1 | 0.412863 | 1.95E-23 |
| BCL2 | AL079303.1 | 0.412865 | 1.95E-23 |
| FOXO1 | AC027097.1 | 0.412867 | 1.95E-23 |
| UVRAG | AL133330.1 | 0.412919 | 1.92E-23 |
| TSC1 | AC022173.1 | 0.412946 | 1.90E-23 |
| RAB33B | AC007684.1 | 0.412956 | 1.90E-23 |
| SPNS1 | AL022328.4 | 0.412968 | 1.89E-23 |
| FOXO3 | AC013403.2 | 0.412974 | 1.89E-23 |
| FOXO1 | PSMA3-AS1 | 0.412983 | 1.89E-23 |
| GOPC | AP000240.1 | 0.412983 | 1.89E-23 |
| RB1 | AC002044.1 | 0.412999 | 1.88E-23 |
| GOPC | AC009318.2 | 0.413004 | 1.87E-23 |
| BIRC6 | ASH1L-AS1 | 0.413031 | 1.86E-23 |
| KIF5B | AC007684.1 | 0.413042 | 1.86E-23 |
| MAPK8 | AC008982.2 | 0.413042 | 1.86E-23 |
| ATG12 | SBF2-AS1 | 0.413069 | 1.84E-23 |
| ATG12 | PAXIP1-AS2 | 0.41308 | 1.84E-23 |
| NLRC4 | GK-AS1 | 0.413156 | 1.80E-23 |
| MAPK8 | AC055822.1 | 0.413156 | 1.80E-23 |
| RPS6KB1 | AL136531.1 | 0.413171 | 1.79E-23 |
| RAB5A | AL138963.1 | 0.413253 | 1.75E-23 |
| CFLAR | AL139289.1 | 0.413278 | 1.74E-23 |
| TSC1 | AC020913.3 | 0.413287 | 1.74E-23 |
| KLHL24 | AC037198.1 | 0.413288 | 1.74E-23 |
| CAPN10 | LINC00174 | 0.413313 | 1.73E-23 |
| BNIP1 | AC008443.4 | 0.413345 | 1.71E-23 |
| PIK3C3 | AC093495.1 | 0.413347 | 1.71E-23 |
| ATG7 | AP005131.7 | 0.413348 | 1.71E-23 |
| EEF2K | AL031717.1 | 0.413373 | 1.70E-23 |
| CFLAR | AL136531.1 | 0.413386 | 1.69E-23 |
| MAPK8 | AL136295.6 | 0.413419 | 1.68E-23 |
| CAPN10 | AC132192.2 | 0.413426 | 1.67E-23 |
| CAPN10 | AC011481.1 | 0.413437 | 1.67E-23 |
| PTEN | DGUOK-AS1 | 0.413439 | 1.67E-23 |
| MAPK8 | AL031673.1 | 0.413449 | 1.66E-23 |
| GOPC | AC124319.2 | 0.413462 | 1.66E-23 |
| RB1 | AC090517.2 | 0.413478 | 1.65E-23 |
| UVRAG | C5orf56 | 0.413484 | 1.65E-23 |
| UVRAG | AC007038.1 | 0.41349 | 1.65E-23 |
| KIF5B | EBLN3P | 0.413491 | 1.65E-23 |
| PIK3C3 | TPT1-AS1 | 0.413536 | 1.63E-23 |
| RAB33B | LINC00216 | 0.413567 | 1.61E-23 |
| TSC1 | AP000866.6 | 0.413579 | 1.61E-23 |
| TSC1 | AC079907.1 | 0.41358 | 1.61E-23 |
| CFLAR | LINC01547 | 0.413591 | 1.60E-23 |
| CASP8 | AC138207.4 | 0.4136 | 1.60E-23 |
| ATG12 | AC025857.2 | 0.413605 | 1.60E-23 |
| RB1CC1 | AC120053.1 | 0.413638 | 1.58E-23 |
| KLHL24 | AC007406.5 | 0.413638 | 1.58E-23 |
| ATG7 | AC073651.1 | 0.413648 | 1.58E-23 |
| SIRT1 | AC087752.4 | 0.413666 | 1.57E-23 |
| EIF2AK2 | AC138956.2 | 0.413705 | 1.55E-23 |
| ATG16L2 | AC108488.1 | 0.413711 | 1.55E-23 |
| ATG12 | AL450263.1 | 0.413724 | 1.55E-23 |
| CFLAR | GK-AS1 | 0.413734 | 1.54E-23 |
| MBTPS2 | AC018752.1 | 0.41376 | 1.53E-23 |
| GOPC | AC002553.1 | 0.413765 | 1.53E-23 |
| RAB5A | AC011939.2 | 0.413775 | 1.52E-23 |
| SPNS1 | LINC01786 | 0.413792 | 1.52E-23 |
| NAF1 | NORAD | 0.413794 | 1.52E-23 |
| NAF1 | AL354733.3 | 0.413813 | 1.51E-23 |
| RPS6KB1 | AP003392.1 | 0.413825 | 1.50E-23 |
| ERN1 | AL031717.1 | 0.413842 | 1.50E-23 |
| GOPC | AL450263.1 | 0.413844 | 1.50E-23 |
| RB1CC1 | LINC01578 | 0.413873 | 1.49E-23 |
| WDFY3 | AL731567.1 | 0.413877 | 1.48E-23 |
| TSC1 | AL590723.1 | 0.413893 | 1.48E-23 |
| PIK3C3 | AL157838.1 | 0.413901 | 1.47E-23 |
| ERN1 | AL354733.3 | 0.413918 | 1.47E-23 |
| EIF2AK2 | AC092953.2 | 0.413961 | 1.45E-23 |
| KIF5B | AL138963.1 | 0.413994 | 1.44E-23 |
| NAF1 | AL117336.2 | 0.414033 | 1.42E-23 |
| MAPK8 | AC048382.2 | 0.414071 | 1.41E-23 |
| GOPC | AL031716.1 | 0.414088 | 1.40E-23 |
| TSC1 | AC004982.1 | 0.4141 | 1.40E-23 |
| ULK3 | AC012615.6 | 0.414101 | 1.40E-23 |
| CAPN10 | AL359921.2 | 0.414103 | 1.40E-23 |
| FOXO3 | AP003170.3 | 0.414129 | 1.39E-23 |
| EIF2AK2 | AC037198.1 | 0.414142 | 1.38E-23 |
| PIK3C3 | AP003392.1 | 0.41415 | 1.38E-23 |
| ITPR1 | RAP2C-AS1 | 0.414171 | 1.37E-23 |
| MAPK8 | AC012181.2 | 0.414181 | 1.37E-23 |
| ATG12 | AC093726.1 | 0.414182 | 1.37E-23 |
| EIF2AK2 | MIR181A2HG | 0.414184 | 1.37E-23 |
| KLHL24 | ACTA2-AS1 | 0.414189 | 1.36E-23 |
| FOXO1 | LINC01290 | 0.414203 | 1.36E-23 |
| TSC1 | AC096741.1 | 0.414244 | 1.34E-23 |
| RAB33B | EGOT | 0.414244 | 1.34E-23 |
| GOPC | AC105389.2 | 0.414251 | 1.34E-23 |
| MBTPS2 | AC114760.2 | 0.414257 | 1.34E-23 |
| TSC1 | AC015726.1 | 0.414258 | 1.34E-23 |
| GOPC | ARHGEF38-IT1 | 0.414262 | 1.34E-23 |
| KIF5B | AC068792.1 | 0.414262 | 1.34E-23 |
| RB1CC1 | AC008969.1 | 0.414265 | 1.34E-23 |
| RPS6KB1 | AC073655.2 | 0.414309 | 1.32E-23 |
| PIK3R4 | OIP5-AS1 | 0.414313 | 1.32E-23 |
| BIRC6 | PDXDC2P-NPIPB14P | 0.414366 | 1.30E-23 |
| ATG16L2 | KRT7-AS | 0.414388 | 1.29E-23 |
| RAB5A | RHOA-IT1 | 0.4144 | 1.29E-23 |
| NLRC4 | AC005632.2 | 0.414431 | 1.28E-23 |
| FOXO1 | AC079684.1 | 0.414455 | 1.27E-23 |
| RB1CC1 | AL117336.2 | 0.414474 | 1.26E-23 |
| ARNT | AP001469.3 | 0.414509 | 1.25E-23 |
| ATG4B | AC004918.1 | 0.41453 | 1.24E-23 |
| RB1 | ATP1A1-AS1 | 0.414563 | 1.23E-23 |
| ATG2B | AC012360.3 | 0.414599 | 1.22E-23 |
| TSC1 | AC087284.1 | 0.414617 | 1.22E-23 |
| NAF1 | AC005261.1 | 0.414618 | 1.22E-23 |
| RAB5A | AL592148.3 | 0.414641 | 1.21E-23 |
| RB1 | AC021078.1 | 0.414651 | 1.20E-23 |
| RPS6KB1 | AL159169.2 | 0.414653 | 1.20E-23 |
| FOXO3 | AC012557.1 | 0.414654 | 1.20E-23 |
| TSC1 | AC132872.3 | 0.414658 | 1.20E-23 |
| RPS6KB1 | AP001486.2 | 0.414677 | 1.20E-23 |
| CFLAR | HIF1A-AS2 | 0.414678 | 1.20E-23 |
| TSC1 | KLF7-IT1 | 0.414711 | 1.19E-23 |
| ATG7 | AP002336.2 | 0.414726 | 1.18E-23 |
| PIK3C3 | AL031717.1 | 0.41474 | 1.18E-23 |
| CAPN10 | AC026979.2 | 0.414749 | 1.17E-23 |
| ATG16L2 | AC105020.1 | 0.414751 | 1.17E-23 |
| MAPK8 | AC133644.2 | 0.414764 | 1.17E-23 |
| RB1 | AC010226.1 | 0.414855 | 1.14E-23 |
| NAF1 | AL158212.3 | 0.414858 | 1.14E-23 |
| ATG4B | SLC9A3-AS1 | 0.414875 | 1.13E-23 |
| CDKN2A | MELTF-AS1 | 0.414915 | 1.12E-23 |
| UVRAG | AC114760.2 | 0.41492 | 1.12E-23 |
| EEF2K | AC004656.1 | 0.414927 | 1.12E-23 |
| KIF5B | AP001432.1 | 0.41494 | 1.11E-23 |
| FOXO1 | MCM3AP-AS1 | 0.414952 | 1.11E-23 |
| TSC1 | AC018521.6 | 0.414954 | 1.11E-23 |
| IL24 | LINC00861 | 0.414975 | 1.10E-23 |
| UVRAG | AC090948.1 | 0.414975 | 1.10E-23 |
| RPS6KB1 | AC092953.2 | 0.41506 | 1.08E-23 |
| CFLAR | AC005332.3 | 0.415078 | 1.07E-23 |
| PIK3C3 | AC048341.2 | 0.415078 | 1.07E-23 |
| IFNG | AC012181.2 | 0.415117 | 1.06E-23 |
| KIF5B | AC124283.3 | 0.415141 | 1.06E-23 |
| PTEN | AL021878.2 | 0.415142 | 1.06E-23 |
| ULK3 | AL135999.1 | 0.415153 | 1.05E-23 |
| UVRAG | AL731566.1 | 0.415162 | 1.05E-23 |
| NAF1 | AL132780.1 | 0.415168 | 1.05E-23 |
| ATG12 | AL136531.1 | 0.415185 | 1.04E-23 |
| FOXO3 | AC024933.1 | 0.415188 | 1.04E-23 |
| TSC2 | PRKCZ-AS1 | 0.415199 | 1.04E-23 |
| UVRAG | AC010186.3 | 0.415227 | 1.03E-23 |
| FOXO3 | AC135050.5 | 0.415243 | 1.03E-23 |
| ATG4B | AL022328.4 | 0.415269 | 1.02E-23 |
| PEX14 | AC139530.1 | 0.41529 | 1.01E-23 |
| ATG12 | AL132657.1 | 0.415297 | 1.01E-23 |
| CFLAR | NFYC-AS1 | 0.415307 | 1.01E-23 |
| PTEN | AL132657.1 | 0.415323 | 1.01E-23 |
| CXCR4 | LINC00996 | 0.415324 | 1.01E-23 |
| FOXO1 | AP003486.1 | 0.415337 | 1.00E-23 |
| RAB33B | GAS5-AS1 | 0.415348 | 9.99E-24 |
| SPNS1 | AL139349.1 | 0.415365 | 9.94E-24 |
| FOXO1 | GK-AS1 | 0.415376 | 9.91E-24 |
| TSC1 | AF117829.1 | 0.415383 | 9.89E-24 |
| TSC1 | AC124045.1 | 0.415389 | 9.87E-24 |
| RAB5A | AP001033.2 | 0.415393 | 9.87E-24 |
| FOXO3 | ATP1A1-AS1 | 0.415399 | 9.85E-24 |
| BIRC6 | RMRP | 0.415404 | 9.84E-24 |
| DAPK2 | AC116407.1 | 0.415418 | 9.80E-24 |
| KIF5B | AC004918.3 | 0.415432 | 9.76E-24 |
| MBTPS2 | FTX | 0.415448 | 9.72E-24 |
| PTEN | AL512413.1 | 0.415455 | 9.70E-24 |
| FOXO3 | AL450263.1 | 0.415464 | 9.68E-24 |
| ATG16L2 | AC016957.2 | 0.415477 | 9.64E-24 |
| KLHL24 | AC048341.1 | 0.415486 | 9.62E-24 |
| GOPC | AL132989.1 | 0.41549 | 9.61E-24 |
| MAPK8 | AL157394.1 | 0.415491 | 9.61E-24 |
| ATG7 | KIF26B-AS1 | 0.415507 | 9.57E-24 |
| ATG16L2 | AC009090.1 | 0.415517 | 9.54E-24 |
| KIF5B | AC022211.1 | 0.415522 | 9.53E-24 |
| GOPC | AC067817.2 | 0.415535 | 9.49E-24 |
| UVRAG | AP003486.1 | 0.415556 | 9.44E-24 |
| EIF2AK2 | AL157392.4 | 0.415626 | 9.26E-24 |
| RB1 | AP000786.1 | 0.415646 | 9.21E-24 |
| KLHL24 | AC080013.4 | 0.415708 | 9.06E-24 |
| GOPC | AC127024.5 | 0.415709 | 9.06E-24 |
| ATG16L2 | AC135050.5 | 0.415732 | 9.00E-24 |
| RGS19 | AC090559.1 | 0.415742 | 8.98E-24 |
| BIRC6 | AC068768.1 | 0.415748 | 8.96E-24 |
| FOXO3 | AP001486.2 | 0.41577 | 8.91E-24 |
| RAB24 | AC027796.4 | 0.415781 | 8.88E-24 |
| RB1 | AC096921.2 | 0.415784 | 8.88E-24 |
| ATG7 | AL359076.1 | 0.415801 | 8.84E-24 |
| EIF2AK2 | AC084824.5 | 0.415838 | 8.75E-24 |
| KLHL24 | LINC01655 | 0.415847 | 8.73E-24 |
| MAPK8 | AL109761.1 | 0.415865 | 8.68E-24 |
| TSC1 | AL513477.2 | 0.415901 | 8.60E-24 |
| ATG4B | AC092171.4 | 0.415933 | 8.53E-24 |
| PIK3C3 | AC023043.4 | 0.415942 | 8.51E-24 |
| TSC1 | AL121839.2 | 0.415952 | 8.48E-24 |
| BIRC6 | AC019131.2 | 0.415959 | 8.47E-24 |
| ATG4B | AC012645.3 | 0.415974 | 8.43E-24 |
| TSC1 | MALAT1 | 0.416026 | 8.31E-24 |
| RB1 | AC093788.1 | 0.416027 | 8.31E-24 |
| BIRC6 | AC073957.3 | 0.416097 | 8.16E-24 |
| MAPK8 | MCCC1-AS1 | 0.416103 | 8.14E-24 |
| TSC1 | AP001381.1 | 0.416111 | 8.13E-24 |
| KIF5B | AL163051.2 | 0.416129 | 8.09E-24 |
| GABARAPL2 | AL354920.1 | 0.416134 | 8.07E-24 |
| EIF2AK2 | AL513534.1 | 0.416135 | 8.07E-24 |
| TSC1 | AC016727.1 | 0.416144 | 8.05E-24 |
| PTEN | AC011477.3 | 0.416157 | 8.02E-24 |
| PIK3R4 | AC022150.4 | 0.416192 | 7.95E-24 |
| GOPC | C5orf56 | 0.416268 | 7.79E-24 |
| RPS6KB1 | AC245884.8 | 0.416288 | 7.75E-24 |
| NLRC4 | AC018682.1 | 0.41629 | 7.74E-24 |
| PIK3C3 | ZKSCAN2-DT | 0.416292 | 7.74E-24 |
| MAPK8 | AC069023.1 | 0.416308 | 7.70E-24 |
| TSC1 | AP001160.1 | 0.416344 | 7.63E-24 |
| MTOR | AC005288.1 | 0.416354 | 7.61E-24 |
| CFLAR | AP001628.1 | 0.416357 | 7.60E-24 |
| RB1 | SNHG26 | 0.416389 | 7.54E-24 |
| UVRAG | AC015911.3 | 0.416411 | 7.49E-24 |
| GOPC | AL359962.2 | 0.416443 | 7.43E-24 |
| CAPN10 | AC011461.1 | 0.416482 | 7.35E-24 |
| UVRAG | AL117381.1 | 0.416519 | 7.28E-24 |
| TSC2 | AL691432.2 | 0.416522 | 7.27E-24 |
| TSC1 | AC130650.2 | 0.41653 | 7.25E-24 |
| TSC2 | AL135999.1 | 0.416536 | 7.24E-24 |
| MAPK8 | AC008870.2 | 0.416538 | 7.24E-24 |
| RB1CC1 | AL157394.1 | 0.416575 | 7.17E-24 |
| WDFY3 | AC244197.2 | 0.416595 | 7.13E-24 |
| NAF1 | SNHG26 | 0.41661 | 7.10E-24 |
| RAB24 | AP002807.1 | 0.416622 | 7.07E-24 |
| MBTPS2 | AC022173.1 | 0.416637 | 7.05E-24 |
| PIK3C3 | AC133644.2 | 0.416651 | 7.02E-24 |
| PTEN | AC008280.3 | 0.416672 | 6.98E-24 |
| ATG4B | LINC01770 | 0.416735 | 6.86E-24 |
| MAP2K7 | AC008735.2 | 0.41675 | 6.83E-24 |
| EIF2AK2 | ACTA2-AS1 | 0.416774 | 6.79E-24 |
| KLHL24 | ARMCX5-GPRASP2 | 0.416775 | 6.79E-24 |
| TSC1 | AL162724.1 | 0.416791 | 6.76E-24 |
| EIF2AK2 | AC025171.4 | 0.416797 | 6.75E-24 |
| ATG12 | AC025165.4 | 0.416805 | 6.73E-24 |
| PIK3R4 | ARHGAP31-AS1 | 0.416813 | 6.72E-24 |
| EIF2AK2 | AC055822.1 | 0.416834 | 6.68E-24 |
| UVRAG | FAM13A-AS1 | 0.41687 | 6.61E-24 |
| PEX14 | PIK3CD-AS2 | 0.416878 | 6.60E-24 |
| TSC2 | AC087741.1 | 0.416894 | 6.57E-24 |
| FOXO3 | AL513008.1 | 0.416907 | 6.55E-24 |
| CAPN10 | AC110285.2 | 0.416939 | 6.49E-24 |
| ARSB | AC090559.1 | 0.416979 | 6.42E-24 |
| ATG7 | Z83843.1 | 0.417011 | 6.37E-24 |
| ATG4B | AL391244.1 | 0.417023 | 6.35E-24 |
| RB1 | AC004223.3 | 0.417098 | 6.22E-24 |
| FOXO3 | AL513327.1 | 0.4171 | 6.21E-24 |
| BIRC6 | MKLN1-AS | 0.417118 | 6.18E-24 |
| KLHL24 | AC068152.1 | 0.417119 | 6.18E-24 |
| TSC1 | AL137003.2 | 0.417126 | 6.17E-24 |
| EIF2AK2 | AC074033.1 | 0.417151 | 6.13E-24 |
| RPS6KB1 | MAST4-AS1 | 0.417183 | 6.08E-24 |
| EIF2AK2 | AC087222.1 | 0.41719 | 6.06E-24 |
| UVRAG | AC066613.1 | 0.417193 | 6.06E-24 |
| KIF5B | ABALON | 0.417209 | 6.03E-24 |
| RB1 | MCM3AP-AS1 | 0.417213 | 6.03E-24 |
| GOPC | LINC00894 | 0.417214 | 6.03E-24 |
| UVRAG | SCARNA9 | 0.417224 | 6.01E-24 |
| ATG4B | AP001029.1 | 0.41723 | 6.00E-24 |
| RAB33B | AL078581.1 | 0.417237 | 5.99E-24 |
| PIK3C3 | AC078883.1 | 0.417271 | 5.93E-24 |
| FOXO3 | AC004908.2 | 0.417303 | 5.88E-24 |
| MBTPS2 | MALAT1 | 0.417307 | 5.87E-24 |
| FOXO3 | AC138956.1 | 0.417389 | 5.75E-24 |
| TSC1 | AC087286.4 | 0.417394 | 5.74E-24 |
| KLHL24 | AC098484.1 | 0.417405 | 5.72E-24 |
| ATG2B | AC093726.1 | 0.417435 | 5.67E-24 |
| RPS6KB1 | FLNB-AS1 | 0.417439 | 5.67E-24 |
| FOXO3 | AC073487.1 | 0.417468 | 5.62E-24 |
| TSC1 | AC233728.1 | 0.417473 | 5.62E-24 |
| ATG2B | AL157871.2 | 0.417482 | 5.60E-24 |
| RAB24 | AC006435.2 | 0.417485 | 5.60E-24 |
| TSC1 | AC004918.1 | 0.417513 | 5.55E-24 |
| MBTPS2 | SP2-AS1 | 0.417515 | 5.55E-24 |
| TSC1 | AC074032.1 | 0.417527 | 5.53E-24 |
| KLHL24 | AC073655.2 | 0.417567 | 5.47E-24 |
| RGS19 | SMIM25 | 0.4176 | 5.43E-24 |
| UVRAG | AL049840.5 | 0.41762 | 5.40E-24 |
| UVRAG | LIMS1-AS1 | 0.417622 | 5.39E-24 |
| NAF1 | LINC01376 | 0.417625 | 5.39E-24 |
| BNIP1 | PITPNA-AS1 | 0.417653 | 5.35E-24 |
| ATG12 | LINC01389 | 0.417657 | 5.34E-24 |
| NAF1 | AL606834.2 | 0.417682 | 5.31E-24 |
| GOPC | AL354989.1 | 0.417731 | 5.24E-24 |
| UVRAG | AL021578.1 | 0.417732 | 5.23E-24 |
| UVRAG | ABALON | 0.417737 | 5.23E-24 |
| DLC1 | AL133355.1 | 0.417748 | 5.21E-24 |
| ATG7 | MIATNB | 0.417754 | 5.20E-24 |
| RB1CC1 | AC005046.1 | 0.41777 | 5.18E-24 |
| SPNS1 | AC006942.1 | 0.417772 | 5.18E-24 |
| MAPK8 | AL117379.1 | 0.417802 | 5.14E-24 |
| FOXO3 | AC005838.2 | 0.41785 | 5.07E-24 |
| FOXO3 | LINC00861 | 0.41785 | 5.07E-24 |
| EIF2AK2 | AC093110.1 | 0.417868 | 5.04E-24 |
| IFNG | LINC00996 | 0.417871 | 5.04E-24 |
| EIF2AK2 | ZNF460-AS1 | 0.417882 | 5.02E-24 |
| ULK3 | AC011472.1 | 0.417926 | 4.96E-24 |
| ULK3 | AC104564.3 | 0.417927 | 4.96E-24 |
| ATG12 | AC011815.1 | 0.417929 | 4.96E-24 |
| ATG7 | AC087286.4 | 0.417946 | 4.94E-24 |
| RAB33B | AC242426.2 | 0.417966 | 4.91E-24 |
| RB1 | AL031666.1 | 0.417981 | 4.89E-24 |
| ATG7 | AC007038.1 | 0.418012 | 4.85E-24 |
| NAF1 | AC007566.1 | 0.418041 | 4.81E-24 |
| RB1 | AC024075.3 | 0.418103 | 4.73E-24 |
| EIF2AK2 | PWAR6 | 0.418107 | 4.73E-24 |
| RB1 | AC083843.2 | 0.418111 | 4.72E-24 |
| RPS6KB1 | AC006213.4 | 0.418114 | 4.72E-24 |
| RB1CC1 | AC067817.2 | 0.418114 | 4.72E-24 |
| ATG4B | AL391244.3 | 0.418134 | 4.69E-24 |
| SH3GLB1 | AC087286.2 | 0.418143 | 4.68E-24 |
| ATG7 | AC016831.4 | 0.418171 | 4.64E-24 |
| MTOR | AC008982.2 | 0.41818 | 4.63E-24 |
| TSC1 | AC108727.1 | 0.418195 | 4.61E-24 |
| KIF5B | NUTM2A-AS1 | 0.418197 | 4.61E-24 |
| KLHL24 | AC008035.1 | 0.418231 | 4.57E-24 |
| PIK3C3 | GABPB1-AS1 | 0.418264 | 4.53E-24 |
| EIF2AK2 | AC115989.1 | 0.418277 | 4.51E-24 |
| CAPN10 | PTOV1-AS1 | 0.418316 | 4.46E-24 |
| MBTPS2 | AC025917.1 | 0.418323 | 4.46E-24 |
| FOXO1 | AC009120.3 | 0.418324 | 4.45E-24 |
| KIF5B | AC097376.2 | 0.418327 | 4.45E-24 |
| PELP1 | AL354892.2 | 0.418358 | 4.41E-24 |
| RB1 | AGAP1-IT1 | 0.41844 | 4.32E-24 |
| NLRC4 | AL157394.1 | 0.418474 | 4.28E-24 |
| WDFY3 | LINC00426 | 0.418499 | 4.25E-24 |
| ATG12 | AL390728.6 | 0.4185 | 4.25E-24 |
| TSC1 | DLEU2 | 0.41852 | 4.22E-24 |
| RB1CC1 | AC087222.1 | 0.418562 | 4.17E-24 |
| NLRC4 | AL035409.1 | 0.418568 | 4.17E-24 |
| CFLAR | AC068594.1 | 0.418575 | 4.16E-24 |
| EDEM1 | AC092611.2 | 0.418578 | 4.16E-24 |
| TSC1 | AC124312.5 | 0.418612 | 4.12E-24 |
| ATG16L2 | AC048382.2 | 0.418624 | 4.10E-24 |
| ATG16L2 | AC025171.4 | 0.418643 | 4.08E-24 |
| MBTPS2 | ALMS1-IT1 | 0.41865 | 4.08E-24 |
| CASP8 | FAM111A-DT | 0.41866 | 4.06E-24 |
| ATG7 | AC110792.3 | 0.418702 | 4.02E-24 |
| MAPK8 | AC129510.1 | 0.418716 | 4.00E-24 |
| RPS6KB1 | UBE2Q1-AS1 | 0.418774 | 3.94E-24 |
| TSC1 | ARHGEF38-IT1 | 0.418793 | 3.92E-24 |
| NAF1 | AL359921.1 | 0.418837 | 3.87E-24 |
| CFLAR | LINC01655 | 0.418878 | 3.83E-24 |
| EEF2K | AC007216.4 | 0.418881 | 3.83E-24 |
| FOXO3 | AL596325.2 | 0.418929 | 3.78E-24 |
| UVRAG | AC124312.5 | 0.418987 | 3.72E-24 |
| ATG7 | AC253576.2 | 0.41899 | 3.71E-24 |
| BIRC6 | AC025165.5 | 0.418998 | 3.71E-24 |
| RAB5A | AC234775.3 | 0.419014 | 3.69E-24 |
| GOPC | AC090198.1 | 0.419022 | 3.68E-24 |
| KIF5B | AL139120.1 | 0.419026 | 3.68E-24 |
| TSC1 | AL136115.2 | 0.419059 | 3.65E-24 |
| ATG12 | LANCL1-AS1 | 0.419072 | 3.63E-24 |
| FOXO3 | AC090425.2 | 0.419129 | 3.58E-24 |
| PIK3C3 | SP2-AS1 | 0.419146 | 3.56E-24 |
| DAPK2 | AL109811.3 | 0.41917 | 3.54E-24 |
| TSC1 | AC107027.3 | 0.419202 | 3.50E-24 |
| UVRAG | AC008115.3 | 0.419204 | 3.50E-24 |
| ULK3 | AP006621.3 | 0.41921 | 3.50E-24 |
| FOXO3 | AC253536.3 | 0.419236 | 3.47E-24 |
| UVRAG | AP005131.7 | 0.419246 | 3.46E-24 |
| TSC2 | AC009065.8 | 0.419251 | 3.46E-24 |
| PIK3C3 | ACTA2-AS1 | 0.419273 | 3.44E-24 |
| ATG7 | AC005046.1 | 0.419279 | 3.43E-24 |
| EIF2AK2 | AC139887.2 | 0.4193 | 3.41E-24 |
| MAPK8 | AC090617.5 | 0.41931 | 3.40E-24 |
| NAF1 | AC018926.2 | 0.419331 | 3.38E-24 |
| KIF5B | AC105389.2 | 0.419348 | 3.37E-24 |
| RPS6KB1 | AC083862.2 | 0.41935 | 3.37E-24 |
| CASP8 | SAP30L-AS1 | 0.419365 | 3.35E-24 |
| UVRAG | AL513365.2 | 0.419448 | 3.28E-24 |
| PIK3C3 | AC055822.1 | 0.419451 | 3.27E-24 |
| MAPK8 | AC120053.1 | 0.419455 | 3.27E-24 |
| IFNG | AC099343.2 | 0.41946 | 3.27E-24 |
| EIF2AK2 | AL157838.1 | 0.419476 | 3.25E-24 |
| NAF1 | AC078778.1 | 0.419489 | 3.24E-24 |
| PIK3C3 | AC087481.3 | 0.41952 | 3.21E-24 |
| ATG7 | AC012181.1 | 0.419579 | 3.16E-24 |
| ATG16L2 | MZF1-AS1 | 0.419595 | 3.15E-24 |
| TSC1 | AP001033.2 | 0.419659 | 3.09E-24 |
| ATG2B | AC024560.3 | 0.419668 | 3.09E-24 |
| KLHL24 | AC011815.1 | 0.419681 | 3.07E-24 |
| CCR2 | AL357060.1 | 0.419704 | 3.06E-24 |
| FOXO1 | OSMR-AS1 | 0.419786 | 2.99E-24 |
| ATG16L2 | GABPB1-AS1 | 0.419796 | 2.98E-24 |
| RB1CC1 | AC009948.1 | 0.419827 | 2.95E-24 |
| SPHK1 | AC093673.1 | 0.419829 | 2.95E-24 |
| RPS6KB1 | AC098851.1 | 0.419844 | 2.94E-24 |
| MAPK8 | AC084871.1 | 0.419857 | 2.93E-24 |
| BIRC6 | NR2F1-AS1 | 0.419866 | 2.92E-24 |
| UVRAG | Z83843.1 | 0.419871 | 2.92E-24 |
| MAPK8 | SNHG26 | 0.41989 | 2.90E-24 |
| GOPC | OSMR-AS1 | 0.419894 | 2.90E-24 |
| KLHL24 | AP006621.2 | 0.419905 | 2.89E-24 |
| FOXO3 | AC124312.2 | 0.419915 | 2.88E-24 |
| MAPK8 | AC024361.1 | 0.419934 | 2.87E-24 |
| CFLAR | AC068580.3 | 0.419939 | 2.86E-24 |
| MAPK8IP1 | AC004540.2 | 0.419951 | 2.86E-24 |
| CFLAR | AP000254.1 | 0.419982 | 2.83E-24 |
| MAPK8 | AC234772.2 | 0.419999 | 2.82E-24 |
| RPS6KB1 | AL354733.3 | 0.420058 | 2.77E-24 |
| PTEN | AC130456.3 | 0.420084 | 2.75E-24 |
| ATG7 | AL645568.1 | 0.420101 | 2.74E-24 |
| CAPN10 | AL360181.2 | 0.420136 | 2.71E-24 |
| ATG4B | MIR503HG | 0.420138 | 2.71E-24 |
| PTEN | AC145423.3 | 0.420148 | 2.71E-24 |
| EIF2AK2 | AC008124.1 | 0.42015 | 2.70E-24 |
| MAPK8 | LINC01578 | 0.42015 | 2.70E-24 |
| UVRAG | AP001432.1 | 0.420175 | 2.69E-24 |
| ULK3 | AC006435.2 | 0.420185 | 2.68E-24 |
| ATG16L2 | AC073655.2 | 0.420202 | 2.67E-24 |
| ATG16L2 | AC245060.2 | 0.420242 | 2.64E-24 |
| RB1CC1 | GAS8-AS1 | 0.420264 | 2.62E-24 |
| FOXO3 | AL137782.1 | 0.420283 | 2.61E-24 |
| PTEN | ZNF32-AS2 | 0.420315 | 2.58E-24 |
| UVRAG | AL049840.1 | 0.420317 | 2.58E-24 |
| BIRC6 | AC093227.1 | 0.420318 | 2.58E-24 |
| CFLAR | AL445222.1 | 0.420348 | 2.56E-24 |
| RB1 | AC090948.3 | 0.420426 | 2.51E-24 |
| FOXO1 | DLEU2 | 0.420444 | 2.49E-24 |
| RPS6KB1 | AC092611.2 | 0.420476 | 2.47E-24 |
| KLHL24 | AC020915.3 | 0.420523 | 2.44E-24 |
| CFLAR | AC004477.3 | 0.420525 | 2.44E-24 |
| FOXO1 | AL132780.1 | 0.42053 | 2.44E-24 |
| MAPK8 | AC096921.2 | 0.420551 | 2.42E-24 |
| GOPC | HCG11 | 0.420556 | 2.42E-24 |
| FOXO3 | AC004223.3 | 0.420569 | 2.41E-24 |
| GOPC | AL513327.1 | 0.420579 | 2.40E-24 |
| GOPC | AF178030.1 | 0.420597 | 2.39E-24 |
| FOXO3 | AC026124.2 | 0.420598 | 2.39E-24 |
| ATG12 | AC244517.7 | 0.420607 | 2.39E-24 |
| KIF5B | AC107027.3 | 0.420609 | 2.38E-24 |
| NAF1 | LINC01578 | 0.420612 | 2.38E-24 |
| RB1CC1 | AC087752.3 | 0.420635 | 2.37E-24 |
| HDAC6 | ZNF674-AS1 | 0.420667 | 2.35E-24 |
| WDFY3 | DPYD-AS1 | 0.42072 | 2.31E-24 |
| RB1CC1 | MAST4-AS1 | 0.420721 | 2.31E-24 |
| PIK3C3 | AC005674.2 | 0.420743 | 2.30E-24 |
| GOPC | AC016394.1 | 0.420785 | 2.27E-24 |
| NAF1 | AC022211.1 | 0.420794 | 2.27E-24 |
| PTEN | AL049840.4 | 0.420798 | 2.26E-24 |
| RPS6KB1 | AL117336.2 | 0.420801 | 2.26E-24 |
| PRKAB1 | AC135050.6 | 0.420836 | 2.24E-24 |
| RB1 | AC127024.4 | 0.420867 | 2.22E-24 |
| GABARAPL1 | AL445524.1 | 0.420878 | 2.21E-24 |
| GOPC | AC010168.2 | 0.420893 | 2.20E-24 |
| RB1CC1 | AC002128.1 | 0.4209 | 2.20E-24 |
| RB1CC1 | AC009948.4 | 0.420905 | 2.20E-24 |
| RPS6KB1 | AL157786.1 | 0.420957 | 2.17E-24 |
| ATG12 | AP006621.2 | 0.420962 | 2.16E-24 |
| FOXO3 | AC022973.3 | 0.420996 | 2.14E-24 |
| TSC2 | AC020558.2 | 0.42101 | 2.13E-24 |
| FOXO1 | ABALON | 0.421012 | 2.13E-24 |
| RB1 | AL157786.1 | 0.42102 | 2.13E-24 |
| UVRAG | AL139407.1 | 0.421059 | 2.11E-24 |
| FOXO3 | SCARNA9 | 0.42107 | 2.10E-24 |
| MAPK8 | OSMR-AS1 | 0.421095 | 2.09E-24 |
| RAB33B | AL137003.2 | 0.421114 | 2.07E-24 |
| RB1 | AC098851.1 | 0.421145 | 2.06E-24 |
| ATG7 | AC078883.1 | 0.421146 | 2.06E-24 |
| EIF2AK2 | LINC00861 | 0.421174 | 2.04E-24 |
| FOXO1 | ALMS1-IT1 | 0.421228 | 2.01E-24 |
| MAPK8 | AL078581.1 | 0.4213 | 1.97E-24 |
| ATG2B | FAM160A1-DT | 0.421309 | 1.97E-24 |
| RPS6KB1 | AC083949.1 | 0.42136 | 1.94E-24 |
| FOXO1 | AL157932.1 | 0.421377 | 1.93E-24 |
| FOXO3 | AC020915.2 | 0.421416 | 1.91E-24 |
| CAPN10 | AC136475.2 | 0.421449 | 1.89E-24 |
| FOXO3 | AC018926.3 | 0.421468 | 1.88E-24 |
| ATG12 | AL035409.1 | 0.42148 | 1.88E-24 |
| FOXO3 | HCG18 | 0.421482 | 1.87E-24 |
| STK11 | AC007292.1 | 0.421503 | 1.86E-24 |
| CASP4 | PSMB8-AS1 | 0.421504 | 1.86E-24 |
| EEF2K | Z68871.1 | 0.421521 | 1.85E-24 |
| ERN1 | AC130456.3 | 0.421525 | 1.85E-24 |
| EIF2AK2 | TPT1-AS1 | 0.421565 | 1.83E-24 |
| TSC1 | AC079684.1 | 0.421567 | 1.83E-24 |
| UVRAG | GMDS-DT | 0.421576 | 1.83E-24 |
| CFLAR | AC068152.1 | 0.421583 | 1.82E-24 |
| ATG4D | AC005696.1 | 0.421583 | 1.82E-24 |
| GOPC | AC068768.1 | 0.4216 | 1.81E-24 |
| TSC1 | AC090739.1 | 0.421617 | 1.81E-24 |
| RPS6KB1 | AC002128.1 | 0.421674 | 1.78E-24 |
| TSC1 | AL158166.2 | 0.421674 | 1.78E-24 |
| TSC1 | AL139120.1 | 0.421694 | 1.77E-24 |
| TSC1 | ARHGAP27P1-BPTFP1-KPNA2P3 | 0.421717 | 1.76E-24 |
| GAPDH | AC099850.3 | 0.421741 | 1.75E-24 |
| CAPN10 | SNHG20 | 0.421751 | 1.74E-24 |
| FOXO3 | SMC5-AS1 | 0.421767 | 1.73E-24 |
| TSC2 | AC132872.3 | 0.421776 | 1.73E-24 |
| NAF1 | AC009090.3 | 0.421801 | 1.72E-24 |
| CFLAR | AC078846.1 | 0.421829 | 1.70E-24 |
| BIRC6 | THAP9-AS1 | 0.421844 | 1.70E-24 |
| PIK3R4 | AC024075.1 | 0.421849 | 1.69E-24 |
| ATG16L2 | AC145423.3 | 0.421861 | 1.69E-24 |
| PIK3C3 | AC007938.3 | 0.421862 | 1.69E-24 |
| ATG7 | LINC02100 | 0.421868 | 1.69E-24 |
| NAF1 | LINC02352 | 0.421908 | 1.67E-24 |
| PIK3C3 | AL606834.1 | 0.421912 | 1.67E-24 |
| ATG12 | THUMPD3-AS1 | 0.421915 | 1.66E-24 |
| FOXO1 | NORAD | 0.421926 | 1.66E-24 |
| ATG7 | AC090948.1 | 0.421933 | 1.66E-24 |
| UVRAG | RAP2C-AS1 | 0.421961 | 1.64E-24 |
| WIPI2 | AC018647.2 | 0.421969 | 1.64E-24 |
| ATG12 | CCDC18-AS1 | 0.42202 | 1.62E-24 |
| FKBP1B | RNASEH1-AS1 | 0.422021 | 1.62E-24 |
| FOXO3 | AC009120.3 | 0.422054 | 1.60E-24 |
| FOXO3 | AC015849.3 | 0.422065 | 1.60E-24 |
| CFLAR | AC053527.1 | 0.422067 | 1.60E-24 |
| PIK3C3 | AC007566.1 | 0.42207 | 1.59E-24 |
| UVRAG | FTX | 0.422074 | 1.59E-24 |
| TSC1 | AC010761.1 | 0.422088 | 1.59E-24 |
| CFLAR | GEMIN7-AS1 | 0.422103 | 1.58E-24 |
| SPNS1 | AL928654.2 | 0.422116 | 1.57E-24 |
| ATG16L2 | C1RL-AS1 | 0.422139 | 1.56E-24 |
| PTEN | AC018645.2 | 0.422176 | 1.55E-24 |
| FOXO1 | AP005899.1 | 0.422191 | 1.54E-24 |
| ULK3 | AC008735.2 | 0.42221 | 1.53E-24 |
| WDFY3 | AC018521.6 | 0.422214 | 1.53E-24 |
| ATG16L2 | AC138956.2 | 0.422222 | 1.53E-24 |
| MBTPS2 | AL133243.2 | 0.422247 | 1.52E-24 |
| CAPN10 | AC012510.1 | 0.422254 | 1.51E-24 |
| ATG12 | AC008014.1 | 0.422282 | 1.50E-24 |
| RPS6KB1 | AC026355.1 | 0.422365 | 1.47E-24 |
| FOXO1 | AL021707.8 | 0.422376 | 1.46E-24 |
| MBTPS2 | AC092801.1 | 0.422426 | 1.44E-24 |
| MAPK8 | UBE2Q1-AS1 | 0.422427 | 1.44E-24 |
| MAPK8 | SP2-AS1 | 0.422431 | 1.44E-24 |
| PIK3C3 | RPS6KA2-IT1 | 0.422475 | 1.43E-24 |
| FOXO3 | PAXIP1-AS2 | 0.422486 | 1.42E-24 |
| EEF2 | EPB41L4A-AS1 | 0.422487 | 1.42E-24 |
| WDFY3 | AC048382.2 | 0.42249 | 1.42E-24 |
| GOPC | AC020915.3 | 0.422514 | 1.41E-24 |
| ATG12 | XIST | 0.422531 | 1.40E-24 |
| CFLAR | AF131215.5 | 0.422546 | 1.40E-24 |
| KLHL24 | AP000240.1 | 0.422566 | 1.39E-24 |
| RAB5A | AC108449.2 | 0.422572 | 1.39E-24 |
| TSC2 | AL513320.1 | 0.422589 | 1.38E-24 |
| SPNS1 | LINC00265 | 0.422592 | 1.38E-24 |
| PIK3C3 | AC024060.1 | 0.422634 | 1.36E-24 |
| TSC1 | AC027117.1 | 0.422638 | 1.36E-24 |
| RB1CC1 | AL021707.7 | 0.422643 | 1.36E-24 |
| FOXO1 | AL078581.1 | 0.422644 | 1.36E-24 |
| PIK3C3 | AC092953.2 | 0.422671 | 1.35E-24 |
| FOXO3 | FLNB-AS1 | 0.422671 | 1.35E-24 |
| BIRC6 | AC090589.3 | 0.422681 | 1.35E-24 |
| MBTPS2 | AL353804.1 | 0.422719 | 1.33E-24 |
| ATG16L2 | AC068580.1 | 0.422796 | 1.30E-24 |
| GOPC | AC091185.1 | 0.422834 | 1.29E-24 |
| ATG7 | LINC00852 | 0.422898 | 1.27E-24 |
| EIF2AK2 | GABPB1-AS1 | 0.422907 | 1.26E-24 |
| EIF2AK2 | AC011676.1 | 0.422947 | 1.25E-24 |
| MAPK8 | AL359915.2 | 0.422956 | 1.25E-24 |
| BNIP1 | PXN-AS1 | 0.422976 | 1.24E-24 |
| ATG16L2 | AL021707.7 | 0.422986 | 1.24E-24 |
| CFLAR | AC084824.5 | 0.42299 | 1.24E-24 |
| CFLAR | AL513550.1 | 0.422998 | 1.23E-24 |
| ULK3 | AC132872.3 | 0.423015 | 1.23E-24 |
| ATG16L2 | FMR1-IT1 | 0.423043 | 1.22E-24 |
| ATG16L2 | CBR3-AS1 | 0.423066 | 1.21E-24 |
| TSC1 | AC092801.1 | 0.42308 | 1.21E-24 |
| UVRAG | AC025917.1 | 0.423084 | 1.20E-24 |
| NAF1 | MIATNB | 0.423099 | 1.20E-24 |
| KLHL24 | AC091185.1 | 0.423122 | 1.19E-24 |
| RAB24 | AC132872.1 | 0.423125 | 1.19E-24 |
| KIF5B | AC010834.3 | 0.423225 | 1.16E-24 |
| ATG7 | AC074032.1 | 0.423226 | 1.16E-24 |
| BIRC6 | PAXIP1-AS2 | 0.423244 | 1.15E-24 |
| ATG4B | AC003070.1 | 0.423257 | 1.15E-24 |
| FOXO3 | AC087286.1 | 0.423263 | 1.15E-24 |
| PTEN | AC139795.2 | 0.423291 | 1.14E-24 |
| RAB33B | AC009948.1 | 0.423305 | 1.13E-24 |
| ATG2B | AC010615.2 | 0.423349 | 1.12E-24 |
| RB1 | C5orf56 | 0.423366 | 1.11E-24 |
| ATG12 | AC093495.1 | 0.423402 | 1.10E-24 |
| TSC1 | MIATNB | 0.423444 | 1.09E-24 |
| CAPN10 | LENG8-AS1 | 0.423455 | 1.09E-24 |
| CAPN10 | AC074117.1 | 0.423482 | 1.08E-24 |
| FOXO1 | AC026368.1 | 0.423607 | 1.04E-24 |
| NAF1 | AL136531.1 | 0.42363 | 1.04E-24 |
| RB1CC1 | AC096921.2 | 0.42363 | 1.04E-24 |
| SPNS1 | AC008735.2 | 0.423656 | 1.03E-24 |
| CFLAR | GAS8-AS1 | 0.423694 | 1.02E-24 |
| UVRAG | SDCBP2-AS1 | 0.423695 | 1.02E-24 |
| ATG7 | AC058791.1 | 0.423704 | 1.01E-24 |
| RAB24 | AL031714.1 | 0.423738 | 1.00E-24 |
| MLST8 | AC008915.2 | 0.423741 | 1.00E-24 |
| FOXO1 | LINC00426 | 0.423741 | 1.00E-24 |
| RB1CC1 | ATP1A1-AS1 | 0.423758 | 9.99E-25 |
| TSC1 | MAL2-AS1 | 0.423797 | 9.88E-25 |
| NAF1 | AC021851.1 | 0.423797 | 9.88E-25 |
| MAPK8 | AC012360.3 | 0.423819 | 9.82E-25 |
| MAPK8 | AL021707.7 | 0.42383 | 9.79E-25 |
| MAPK8 | AC004908.3 | 0.423831 | 9.79E-25 |
| FOXO3 | HCG11 | 0.423849 | 9.74E-25 |
| ATG7 | LINC00630 | 0.423867 | 9.69E-25 |
| EIF2AK2 | AP000240.1 | 0.423885 | 9.64E-25 |
| FOXO3 | AC002128.2 | 0.423892 | 9.62E-25 |
| FOXO3 | AC012181.2 | 0.42392 | 9.55E-25 |
| SIRT2 | AC002398.1 | 0.423947 | 9.48E-25 |
| RB1 | ABALON | 0.423964 | 9.44E-25 |
| EIF2AK2 | LINC01389 | 0.423986 | 9.38E-25 |
| TSC1 | LINC00216 | 0.424011 | 9.31E-25 |
| CFLAR | MIR181A2HG | 0.424025 | 9.28E-25 |
| RB1CC1 | AP001625.2 | 0.424027 | 9.27E-25 |
| ATG12 | AC062037.2 | 0.424035 | 9.25E-25 |
| SIRT1 | AL731566.1 | 0.424043 | 9.23E-25 |
| RAB5A | MAL2-AS1 | 0.424076 | 9.15E-25 |
| SPNS1 | AC007292.1 | 0.424078 | 9.14E-25 |
| CFLAR | AL117379.1 | 0.424128 | 9.02E-25 |
| TSC1 | AC011442.1 | 0.424144 | 8.97E-25 |
| KIF5B | AC092611.2 | 0.424161 | 8.93E-25 |
| FOXO3 | Z98884.2 | 0.424168 | 8.91E-25 |
| TSC1 | HMGA1P4 | 0.424168 | 8.91E-25 |
| MTOR | ABALON | 0.4242 | 8.84E-25 |
| KLHL24 | AC009054.2 | 0.4242 | 8.83E-25 |
| FOXO3 | AL137779.2 | 0.424206 | 8.82E-25 |
| MTOR | AC092611.2 | 0.424209 | 8.81E-25 |
| TSC1 | AC007878.1 | 0.424217 | 8.79E-25 |
| FOXO1 | AC084824.5 | 0.424234 | 8.75E-25 |
| NFKB1 | AC092611.2 | 0.424252 | 8.71E-25 |
| CAPN10 | AL139349.1 | 0.424259 | 8.69E-25 |
| ATG12 | AC008735.4 | 0.424259 | 8.69E-25 |
| EEF2K | EP300-AS1 | 0.424305 | 8.58E-25 |
| RB1 | ATP1B3-AS1 | 0.424352 | 8.47E-25 |
| ATG12 | AC011676.1 | 0.424363 | 8.44E-25 |
| ATG7 | GMDS-DT | 0.424396 | 8.37E-25 |
| FOXO1 | AC005519.1 | 0.424433 | 8.28E-25 |
| FOXO1 | AC007849.1 | 0.42445 | 8.24E-25 |
| ERN1 | AC138956.2 | 0.424507 | 8.11E-25 |
| NLRC4 | AL133342.1 | 0.424545 | 8.03E-25 |
| UVRAG | AC053513.1 | 0.424565 | 7.98E-25 |
| FOXO1 | AC234772.2 | 0.424581 | 7.95E-25 |
| FOXO3 | LAMC1-AS1 | 0.424601 | 7.90E-25 |
| UVRAG | AL606834.2 | 0.424618 | 7.87E-25 |
| NLRC4 | AC018755.4 | 0.424629 | 7.84E-25 |
| UVRAG | AC090948.3 | 0.424639 | 7.82E-25 |
| MAPK8 | AC007319.1 | 0.424639 | 7.82E-25 |
| MAPK8 | AL080317.1 | 0.424656 | 7.78E-25 |
| NAF1 | AC020915.3 | 0.424669 | 7.76E-25 |
| MAPK8 | AC018809.2 | 0.424678 | 7.73E-25 |
| KLHL24 | AC005332.3 | 0.424705 | 7.68E-25 |
| BIRC6 | AC027117.1 | 0.42475 | 7.58E-25 |
| ATG16L2 | AL662844.4 | 0.424787 | 7.50E-25 |
| RPS6KB1 | ARHGAP31-AS1 | 0.424795 | 7.49E-25 |
| GOPC | AC090517.2 | 0.424805 | 7.47E-25 |
| BIRC5 | AC002116.2 | 0.424817 | 7.44E-25 |
| RPS6KB1 | AC124312.5 | 0.424844 | 7.39E-25 |
| RB1 | AC087392.1 | 0.424847 | 7.38E-25 |
| MAPK8IP1 | LINC00261 | 0.424852 | 7.37E-25 |
| ATG16L2 | LINC00482 | 0.424877 | 7.32E-25 |
| CFLAR | AC087752.4 | 0.424891 | 7.29E-25 |
| PIK3C3 | ARMCX5-GPRASP2 | 0.424939 | 7.19E-25 |
| CCR2 | AL590764.1 | 0.42495 | 7.17E-25 |
| EIF2AK2 | AL080317.1 | 0.424982 | 7.11E-25 |
| BIRC6 | CTBP1-AS | 0.424994 | 7.08E-25 |
| UVRAG | STARD4-AS1 | 0.424995 | 7.08E-25 |
| GOPC | AC013403.2 | 0.425009 | 7.05E-25 |
| PIK3C3 | AC139887.1 | 0.42504 | 6.99E-25 |
| ATG7 | MALAT1 | 0.425089 | 6.90E-25 |
| TSC1 | AL157932.1 | 0.425106 | 6.87E-25 |
| NAF1 | FMR1-IT1 | 0.425143 | 6.80E-25 |
| EIF2AK2 | RPS6KA2-IT1 | 0.425159 | 6.77E-25 |
| ATG2B | AC005332.3 | 0.425184 | 6.72E-25 |
| ATG7 | MIR29B2CHG | 0.425187 | 6.71E-25 |
| BIRC6 | GPRC5D-AS1 | 0.42523 | 6.63E-25 |
| ATG16L2 | AC005332.4 | 0.425231 | 6.63E-25 |
| MAPK8 | C5orf56 | 0.425231 | 6.63E-25 |
| PIK3C3 | AC011442.1 | 0.425266 | 6.57E-25 |
| SPNS1 | LINC00106 | 0.425321 | 6.47E-25 |
| PRKAB1 | SRP14-AS1 | 0.425388 | 6.35E-25 |
| RB1CC1 | AC234772.2 | 0.425399 | 6.33E-25 |
| EIF2AK2 | AC005046.1 | 0.425401 | 6.32E-25 |
| ATG16L2 | AL162274.2 | 0.425445 | 6.25E-25 |
| PTEN | AL121584.1 | 0.425449 | 6.24E-25 |
| TSC1 | AL133243.2 | 0.425451 | 6.24E-25 |
| EIF2AK2 | EBLN3P | 0.425465 | 6.21E-25 |
| GOPC | AL360219.1 | 0.425476 | 6.19E-25 |
| ATG2B | AC244197.2 | 0.425517 | 6.12E-25 |
| MAPK8 | ACTA2-AS1 | 0.42552 | 6.12E-25 |
| BECN1 | AC067852.2 | 0.425526 | 6.11E-25 |
| ATG7 | LINC-PINT | 0.425561 | 6.05E-25 |
| KIF5B | AC130895.1 | 0.425566 | 6.04E-25 |
| GOPC | KIF26B-AS1 | 0.425567 | 6.04E-25 |
| DAPK2 | AC024075.2 | 0.42559 | 6.00E-25 |
| EIF2AK2 | AC068152.1 | 0.425602 | 5.98E-25 |
| KIF5B | AC026124.2 | 0.425605 | 5.97E-25 |
| GOPC | AL157786.1 | 0.425612 | 5.96E-25 |
| RB1CC1 | AL035409.1 | 0.425616 | 5.95E-25 |
| NAF1 | GARS-DT | 0.425623 | 5.94E-25 |
| ATG16L2 | MCCC1-AS1 | 0.425628 | 5.94E-25 |
| ATG16L2 | AC073842.2 | 0.425631 | 5.93E-25 |
| CFLAR | AC012615.6 | 0.425654 | 5.89E-25 |
| ATG16L2 | AP001029.1 | 0.425673 | 5.86E-25 |
| TSC1 | AL353804.1 | 0.425703 | 5.81E-25 |
| FOXO3 | AL365277.1 | 0.425716 | 5.79E-25 |
| TSC1 | AP001628.1 | 0.425764 | 5.71E-25 |
| CFLAR | AC025857.2 | 0.425774 | 5.70E-25 |
| ATG7 | AP001178.2 | 0.425775 | 5.70E-25 |
| ATG7 | AP000692.1 | 0.4258 | 5.66E-25 |
| GOPC | AC007566.1 | 0.42583 | 5.61E-25 |
| EIF2AK2 | AL122035.1 | 0.425835 | 5.60E-25 |
| TSC1 | AC073046.1 | 0.425838 | 5.60E-25 |
| ATG7 | AC022173.1 | 0.425842 | 5.59E-25 |
| MAPK8 | NORAD | 0.425852 | 5.57E-25 |
| MAPK8 | INE1 | 0.425859 | 5.56E-25 |
| MAPK8 | AC008537.2 | 0.425868 | 5.55E-25 |
| RB1CC1 | AL360219.1 | 0.425869 | 5.55E-25 |
| MAPK8 | AL360219.1 | 0.42588 | 5.53E-25 |
| ATG16L2 | DBH-AS1 | 0.425886 | 5.52E-25 |
| TSC1 | AC020594.1 | 0.425902 | 5.50E-25 |
| ATG2B | AC012368.1 | 0.425907 | 5.49E-25 |
| FOXO3 | AC009032.1 | 0.425932 | 5.45E-25 |
| MAPK8 | AC121761.2 | 0.425956 | 5.41E-25 |
| MAPK8 | AC002128.1 | 0.425957 | 5.41E-25 |
| FOXO3 | AC008906.1 | 0.425973 | 5.39E-25 |
| GOPC | AL359697.1 | 0.425976 | 5.39E-25 |
| WDFY3 | HCG27 | 0.425991 | 5.36E-25 |
| RAB5A | FLNB-AS1 | 0.426 | 5.35E-25 |
| SPNS1 | LINC00174 | 0.426007 | 5.34E-25 |
| FOXO3 | ACTA2-AS1 | 0.426055 | 5.27E-25 |
| PIK3C3 | AC002128.1 | 0.426065 | 5.25E-25 |
| KLHL24 | AP003392.1 | 0.426074 | 5.24E-25 |
| TSC1 | AL137782.1 | 0.426079 | 5.23E-25 |
| FOXO3 | AL163051.2 | 0.426085 | 5.22E-25 |
| BIRC6 | AL021707.4 | 0.426098 | 5.20E-25 |
| ATG16L2 | AL035587.1 | 0.426112 | 5.18E-25 |
| CFLAR | AC145423.3 | 0.426182 | 5.08E-25 |
| PRKAB1 | AC079384.1 | 0.426188 | 5.07E-25 |
| MBTPS2 | AL136320.1 | 0.426202 | 5.06E-25 |
| ATG2B | AL031775.1 | 0.426212 | 5.04E-25 |
| NAF1 | AP003486.1 | 0.426214 | 5.04E-25 |
| ULK3 | ASB16-AS1 | 0.426218 | 5.03E-25 |
| KLHL24 | AL158212.3 | 0.426237 | 5.01E-25 |
| PARP1 | AC099850.3 | 0.426242 | 5.00E-25 |
| FOXO1 | AC087392.1 | 0.426246 | 4.99E-25 |
| DAPK2 | AL158071.2 | 0.426265 | 4.97E-25 |
| CAPN10 | AL513218.1 | 0.426274 | 4.95E-25 |
| SIRT1 | OTUD6B-AS1 | 0.426275 | 4.95E-25 |
| UVRAG | AL353804.1 | 0.426374 | 4.82E-25 |
| RPS6KB1 | AC011468.1 | 0.426376 | 4.81E-25 |
| PIK3C3 | AC008035.1 | 0.426385 | 4.80E-25 |
| FOXO3 | AC090948.3 | 0.426388 | 4.80E-25 |
| TSC1 | AC073651.1 | 0.4264 | 4.78E-25 |
| RAB24 | AP006284.1 | 0.426401 | 4.78E-25 |
| MLST8 | AC020663.2 | 0.42644 | 4.73E-25 |
| EEF2K | AP003486.1 | 0.426459 | 4.70E-25 |
| NAF1 | AC026355.1 | 0.426471 | 4.69E-25 |
| FOXO3 | AC008124.1 | 0.426472 | 4.69E-25 |
| KIF5B | AC068768.1 | 0.426492 | 4.66E-25 |
| GOPC | AP001160.4 | 0.426506 | 4.64E-25 |
| RPS6KB1 | AC025171.3 | 0.426524 | 4.62E-25 |
| FOXO3 | AC090181.2 | 0.426525 | 4.62E-25 |
| WDFY3 | MIR3936HG | 0.426536 | 4.60E-25 |
| RAB24 | MMP25-AS1 | 0.426552 | 4.58E-25 |
| BIRC6 | AC060780.1 | 0.426574 | 4.56E-25 |
| IFNG | AC133644.2 | 0.426576 | 4.55E-25 |
| GOPC | AC092794.1 | 0.426583 | 4.54E-25 |
| FOXO1 | AC015871.3 | 0.426594 | 4.53E-25 |
| WDFY3 | AC093297.2 | 0.426598 | 4.52E-25 |
| ATG16L2 | HLA-DQB1-AS1 | 0.426628 | 4.49E-25 |
| ATG16L2 | MUC20-OT1 | 0.426632 | 4.48E-25 |
| RPS6KB1 | AC010536.2 | 0.426646 | 4.46E-25 |
| ATG12 | AL359715.3 | 0.426648 | 4.46E-25 |
| UVRAG | AC108010.1 | 0.426675 | 4.43E-25 |
| RB1CC1 | MIATNB | 0.426766 | 4.32E-25 |
| ATG12 | ZFPM2-AS1 | 0.426786 | 4.29E-25 |
| KIF5B | AC048344.4 | 0.426788 | 4.29E-25 |
| CFLAR | AC009237.15 | 0.426798 | 4.28E-25 |
| GOPC | AC005021.1 | 0.426811 | 4.26E-25 |
| NCKAP1 | AL606489.1 | 0.426826 | 4.24E-25 |
| CFLAR | AC025171.5 | 0.426859 | 4.20E-25 |
| ATG7 | AC090948.3 | 0.426877 | 4.18E-25 |
| RB1 | AC007319.1 | 0.4269 | 4.16E-25 |
| GOPC | AC104695.3 | 0.426908 | 4.15E-25 |
| FOXO1 | LINC00641 | 0.426909 | 4.15E-25 |
| NAF1 | AC010168.2 | 0.426914 | 4.14E-25 |
| NLRC4 | CARD8-AS1 | 0.426916 | 4.14E-25 |
| ATG12 | AC026368.1 | 0.426945 | 4.10E-25 |
| TSC1 | AC007406.5 | 0.426954 | 4.09E-25 |
| RAB24 | AC135050.3 | 0.426974 | 4.07E-25 |
| CFLAR | AC010168.2 | 0.426982 | 4.06E-25 |
| ATG12 | LINC01004 | 0.426983 | 4.06E-25 |
| RB1CC1 | AC009041.4 | 0.426987 | 4.06E-25 |
| RPS6KB1 | GARS-DT | 0.427026 | 4.01E-25 |
| TSC2 | AC006435.2 | 0.427042 | 3.99E-25 |
| ATG12 | AC004067.1 | 0.427066 | 3.97E-25 |
| CFLAR | AC004908.1 | 0.427072 | 3.96E-25 |
| MAPK8 | AC005046.1 | 0.427082 | 3.95E-25 |
| EIF2AK2 | AL158212.3 | 0.427088 | 3.94E-25 |
| GOPC | AC004477.3 | 0.42709 | 3.94E-25 |
| CAPN10 | AL450384.2 | 0.427132 | 3.89E-25 |
| NAF1 | AC005519.1 | 0.427135 | 3.89E-25 |
| KLHL24 | AC010226.1 | 0.427168 | 3.86E-25 |
| TSC1 | AL365277.1 | 0.427208 | 3.81E-25 |
| RAB24 | AC006942.1 | 0.427241 | 3.78E-25 |
| UVRAG | AC008770.3 | 0.427244 | 3.77E-25 |
| FOXO3 | AC092611.2 | 0.427268 | 3.75E-25 |
| IFNG | AC116366.1 | 0.427291 | 3.72E-25 |
| FOXO1 | OCIAD1-AS1 | 0.427292 | 3.72E-25 |
| FOXO3 | ANKRD10-IT1 | 0.427324 | 3.69E-25 |
| KLHL24 | AC008870.2 | 0.427325 | 3.69E-25 |
| BIRC6 | FAM160A1-DT | 0.427378 | 3.63E-25 |
| CFLAR | AC005540.1 | 0.427384 | 3.63E-25 |
| ATG7 | MAL2-AS1 | 0.427408 | 3.60E-25 |
| KLHL24 | AL031666.1 | 0.42742 | 3.59E-25 |
| BNIP1 | LRRC75A-AS1 | 0.427422 | 3.59E-25 |
| DLC1 | AC137932.1 | 0.427436 | 3.58E-25 |
| ATG7 | AC027097.2 | 0.427593 | 3.42E-25 |
| RPS6KB1 | AC010615.2 | 0.427663 | 3.35E-25 |
| ATG2B | MIR3936HG | 0.427706 | 3.31E-25 |
| FOXO3 | AL354733.3 | 0.427765 | 3.26E-25 |
| PIK3C3 | AC007996.1 | 0.427775 | 3.25E-25 |
| FOXO1 | AC022306.2 | 0.427779 | 3.25E-25 |
| NAF1 | AC015871.3 | 0.427786 | 3.24E-25 |
| MAPK8 | MBNL1-AS1 | 0.427791 | 3.24E-25 |
| SAR1A | OIP5-AS1 | 0.427811 | 3.22E-25 |
| BIRC6 | LINC01876 | 0.427821 | 3.21E-25 |
| PTEN | AC083862.2 | 0.427829 | 3.20E-25 |
| TP73 | AL163051.1 | 0.427834 | 3.20E-25 |
| FOXO3 | AC138393.3 | 0.427855 | 3.18E-25 |
| MAPK8 | AC020978.3 | 0.427857 | 3.18E-25 |
| CFLAR | AC007319.1 | 0.427874 | 3.16E-25 |
| ATG7 | MAGI2-AS3 | 0.42789 | 3.15E-25 |
| RPS6KB1 | AC138956.2 | 0.427896 | 3.14E-25 |
| ATG16L2 | TPT1-AS1 | 0.427896 | 3.14E-25 |
| FOXO3 | AC090198.1 | 0.427904 | 3.14E-25 |
| RAB24 | AC008610.1 | 0.427908 | 3.13E-25 |
| ATG7 | AC112496.1 | 0.427919 | 3.12E-25 |
| MAPK8 | RPS6KA2-IT1 | 0.427922 | 3.12E-25 |
| ATG12 | AP003486.1 | 0.427922 | 3.12E-25 |
| KLHL24 | LINC01578 | 0.427975 | 3.07E-25 |
| RPS6KB1 | DLEU1 | 0.428026 | 3.03E-25 |
| NAF1 | AL109761.1 | 0.428038 | 3.02E-25 |
| PTEN | AC120053.1 | 0.428049 | 3.01E-25 |
| TSC1 | AC010326.3 | 0.428051 | 3.01E-25 |
| WDFY3 | AC025165.5 | 0.428058 | 3.00E-25 |
| EIF2AK2 | AC053527.1 | 0.42808 | 2.98E-25 |
| KLHL24 | AC021851.1 | 0.4281 | 2.97E-25 |
| IFNG | AC007991.2 | 0.428104 | 2.96E-25 |
| UVRAG | AC124312.2 | 0.428111 | 2.96E-25 |
| EIF2AK2 | AC008969.1 | 0.42812 | 2.95E-25 |
| RB1 | AC007014.2 | 0.42815 | 2.93E-25 |
| ATG2B | AC124045.1 | 0.428154 | 2.92E-25 |
| KLHL24 | AC025287.3 | 0.428167 | 2.91E-25 |
| RHEB | AC006333.2 | 0.428178 | 2.90E-25 |
| FOXO3 | NUTM2B-AS1 | 0.428192 | 2.89E-25 |
| KLHL24 | AC015813.1 | 0.428204 | 2.88E-25 |
| MAPK8 | AC245884.8 | 0.428236 | 2.86E-25 |
| NAF1 | AL132989.1 | 0.428238 | 2.85E-25 |
| RB1 | AC092279.1 | 0.428273 | 2.83E-25 |
| ATG16L2 | AL513477.2 | 0.428281 | 2.82E-25 |
| EIF4EBP1 | AP002360.1 | 0.42831 | 2.80E-25 |
| ATG12 | AC123595.1 | 0.428318 | 2.79E-25 |
| RPS6KB1 | AL138921.2 | 0.428322 | 2.79E-25 |
| KIF5B | AP001033.2 | 0.428343 | 2.77E-25 |
| FAS | AC083949.1 | 0.428359 | 2.76E-25 |
| NLRC4 | PCED1B-AS1 | 0.428423 | 2.71E-25 |
| ATG12 | AL359915.2 | 0.428425 | 2.71E-25 |
| EIF2AK2 | AL136531.1 | 0.428441 | 2.70E-25 |
| RAB24 | AL022328.3 | 0.428467 | 2.68E-25 |
| CFLAR | AC048344.4 | 0.428483 | 2.66E-25 |
| TSC1 | AGAP1-IT1 | 0.428485 | 2.66E-25 |
| FOXO3 | AC130895.1 | 0.428495 | 2.65E-25 |
| PIK3C3 | AL354696.1 | 0.428505 | 2.65E-25 |
| ATG7 | AC253536.3 | 0.428546 | 2.62E-25 |
| RPS6KB1 | AL359715.3 | 0.428574 | 2.60E-25 |
| GOPC | AP001486.2 | 0.428601 | 2.58E-25 |
| FOXO3 | AC245014.3 | 0.428602 | 2.58E-25 |
| CFLAR | LINC01376 | 0.428638 | 2.55E-25 |
| MBTPS2 | AL359962.1 | 0.428649 | 2.54E-25 |
| TSC1 | AL021707.3 | 0.428673 | 2.52E-25 |
| WDFY3 | AL138756.1 | 0.428701 | 2.50E-25 |
| PTEN | AL513534.1 | 0.428739 | 2.48E-25 |
| GABARAPL2 | MAPKAPK5-AS1 | 0.428761 | 2.46E-25 |
| RPS6KB1 | AC007552.2 | 0.428767 | 2.46E-25 |
| BIRC6 | AL031775.1 | 0.428778 | 2.45E-25 |
| GOPC | AL138921.2 | 0.428781 | 2.45E-25 |
| CFLAR | AC009041.4 | 0.428805 | 2.43E-25 |
| RB1 | AC007546.1 | 0.428817 | 2.42E-25 |
| KLHL24 | AC087222.1 | 0.428818 | 2.42E-25 |
| CXCR4 | AC145098.1 | 0.428868 | 2.39E-25 |
| NLRC4 | HIF1A-AS2 | 0.428914 | 2.36E-25 |
| KLHL24 | AL353804.2 | 0.428916 | 2.36E-25 |
| PIK3R4 | AC108010.1 | 0.428922 | 2.35E-25 |
| FOXO3 | LINC-PINT | 0.428969 | 2.32E-25 |
| ATG7 | AC068790.2 | 0.428992 | 2.31E-25 |
| PIK3C3 | AL360219.1 | 0.428995 | 2.30E-25 |
| KLHL24 | AC097103.2 | 0.428996 | 2.30E-25 |
| CFLAR | AC232271.1 | 0.429017 | 2.29E-25 |
| RB1 | MAGI2-AS3 | 0.429028 | 2.28E-25 |
| PIK3C3 | OCIAD1-AS1 | 0.429063 | 2.26E-25 |
| TSC1 | AL136295.2 | 0.429101 | 2.24E-25 |
| ATG2B | AL135818.1 | 0.429117 | 2.23E-25 |
| RB1CC1 | ABALON | 0.42913 | 2.22E-25 |
| TSC1 | LINC-PINT | 0.429132 | 2.22E-25 |
| PIK3C3 | AL442125.2 | 0.429141 | 2.21E-25 |
| IKBKB | AC004148.2 | 0.42916 | 2.20E-25 |
| EIF2AK2 | AC091185.1 | 0.429161 | 2.20E-25 |
| RPS6KB1 | AP001625.2 | 0.429169 | 2.19E-25 |
| TSC1 | ACAP2-IT1 | 0.429172 | 2.19E-25 |
| RAB5A | AC087286.4 | 0.429212 | 2.17E-25 |
| TSC1 | OIP5-AS1 | 0.429212 | 2.17E-25 |
| TSC1 | AL132657.1 | 0.429217 | 2.16E-25 |
| FOXO3 | MAGI2-AS3 | 0.42922 | 2.16E-25 |
| CFLAR | AC016957.2 | 0.429237 | 2.15E-25 |
| CFLAR | AL049840.4 | 0.429254 | 2.14E-25 |
| NAF1 | AC013403.2 | 0.429292 | 2.12E-25 |
| RAB33B | TRAF3IP2-AS1 | 0.429318 | 2.10E-25 |
| ATG4B | AC026740.1 | 0.429336 | 2.09E-25 |
| ATG7 | AC087276.1 | 0.429375 | 2.07E-25 |
| RAB24 | LINC01089 | 0.429391 | 2.06E-25 |
| ATG16L2 | AC245884.8 | 0.429396 | 2.06E-25 |
| RB1CC1 | C5orf56 | 0.429404 | 2.05E-25 |
| IFNG | SNHG26 | 0.429431 | 2.04E-25 |
| RB1CC1 | AP003352.1 | 0.429436 | 2.03E-25 |
| FOXO1 | AL662844.3 | 0.429438 | 2.03E-25 |
| KLHL24 | AC004253.1 | 0.429452 | 2.03E-25 |
| PIK3C3 | AC073896.3 | 0.429467 | 2.02E-25 |
| FOXO3 | AC093799.1 | 0.429478 | 2.01E-25 |
| MAPK8 | AC008035.1 | 0.429489 | 2.00E-25 |
| EDEM1 | AC026202.2 | 0.429505 | 2.00E-25 |
| CFLAR | AC004982.1 | 0.429507 | 1.99E-25 |
| BNIP1 | AC012640.2 | 0.429515 | 1.99E-25 |
| RB1CC1 | LINC02352 | 0.429601 | 1.94E-25 |
| PTEN | AC125257.1 | 0.429603 | 1.94E-25 |
| GOPC | AC008124.1 | 0.429633 | 1.92E-25 |
| PELP1 | PITPNA-AS1 | 0.429653 | 1.91E-25 |
| ATG7 | AC026124.2 | 0.429689 | 1.89E-25 |
| RAB24 | AC233728.1 | 0.429719 | 1.88E-25 |
| FOXO3 | AC026356.1 | 0.429738 | 1.87E-25 |
| WDFY3 | AC232271.1 | 0.429752 | 1.86E-25 |
| PIK3C3 | AP001625.2 | 0.429756 | 1.86E-25 |
| RPS6KB1 | AC002553.1 | 0.429758 | 1.86E-25 |
| TSC1 | AC096921.2 | 0.429766 | 1.85E-25 |
| MAPK8 | AC020915.3 | 0.429789 | 1.84E-25 |
| ERN1 | AC024075.1 | 0.429791 | 1.84E-25 |
| WDFY3 | AC006504.8 | 0.429796 | 1.84E-25 |
| TSC1 | AC124283.3 | 0.429799 | 1.84E-25 |
| CAPN10 | AC011498.6 | 0.429853 | 1.81E-25 |
| ATF6 | LINC01376 | 0.429857 | 1.81E-25 |
| ATG2B | LINC01184 | 0.429865 | 1.80E-25 |
| NAF1 | AC016542.1 | 0.42987 | 1.80E-25 |
| TSC1 | AC245014.3 | 0.429884 | 1.79E-25 |
| CFLAR | KRT7-AS | 0.429915 | 1.78E-25 |
| EIF2AK2 | AL442125.2 | 0.429916 | 1.78E-25 |
| KLHL24 | GARS-DT | 0.429936 | 1.77E-25 |
| ATG7 | AC138932.5 | 0.42995 | 1.76E-25 |
| RAB11A | AL158206.1 | 0.429955 | 1.76E-25 |
| RPS6KB1 | AC068152.1 | 0.429958 | 1.76E-25 |
| ATG4C | CARD8-AS1 | 0.429976 | 1.75E-25 |
| CCR2 | PSMB8-AS1 | 0.429976 | 1.75E-25 |
| ATG2B | ARHGAP31-AS1 | 0.429989 | 1.74E-25 |
| EIF2AK2 | AC048341.1 | 0.430019 | 1.73E-25 |
| FOXO3 | KLF7-IT1 | 0.430024 | 1.72E-25 |
| PTEN | AC012368.1 | 0.430024 | 1.72E-25 |
| NAF1 | AC007849.1 | 0.430034 | 1.72E-25 |
| ATG7 | CARD8-AS1 | 0.430039 | 1.72E-25 |
| EIF2AK2 | MAST4-AS1 | 0.430052 | 1.71E-25 |
| GOPC | PWAR6 | 0.430057 | 1.71E-25 |
| RB1CC1 | ARHGAP31-AS1 | 0.430062 | 1.70E-25 |
| GABARAPL1 | DLGAP1-AS1 | 0.430071 | 1.70E-25 |
| ATG7 | AL049869.3 | 0.430088 | 1.69E-25 |
| ATG7 | AC130895.1 | 0.430108 | 1.68E-25 |
| NLRC4 | LINC02345 | 0.430131 | 1.67E-25 |
| PRKAB1 | AC068888.1 | 0.430184 | 1.65E-25 |
| GOPC | AC005632.2 | 0.430186 | 1.65E-25 |
| NAF1 | AL445493.3 | 0.430216 | 1.63E-25 |
| ATG4B | CTBP1-AS | 0.430218 | 1.63E-25 |
| PELP1 | AC012073.1 | 0.430233 | 1.62E-25 |
| ULK3 | LINC01786 | 0.430271 | 1.61E-25 |
| ATG7 | AC108010.1 | 0.430275 | 1.60E-25 |
| RAB24 | AL031600.1 | 0.430296 | 1.59E-25 |
| RAB33B | SAMD12-AS1 | 0.430298 | 1.59E-25 |
| KLHL24 | AC015871.3 | 0.430335 | 1.58E-25 |
| EIF2AK2 | AL035409.1 | 0.430341 | 1.57E-25 |
| RPS6KB1 | AC092794.1 | 0.430353 | 1.57E-25 |
| HIF1A | FLJ22447 | 0.430374 | 1.56E-25 |
| KLHL24 | AC009237.15 | 0.430394 | 1.55E-25 |
| BIRC6 | AC026367.3 | 0.430401 | 1.55E-25 |
| WDFY3 | LINC00886 | 0.430415 | 1.54E-25 |
| MAP2K7 | AL390719.2 | 0.43043 | 1.54E-25 |
| RB1 | AC016542.1 | 0.430445 | 1.53E-25 |
| SIRT2 | SCGB1B2P | 0.43045 | 1.53E-25 |
| TSC1 | HMGN3-AS1 | 0.430454 | 1.52E-25 |
| TSC1 | RRN3P2 | 0.430564 | 1.48E-25 |
| ATG12 | AC139887.2 | 0.430576 | 1.47E-25 |
| ATG7 | AL158166.2 | 0.430595 | 1.46E-25 |
| MTOR | AC098484.1 | 0.430676 | 1.43E-25 |
| EIF2AK2 | AC021851.1 | 0.43071 | 1.42E-25 |
| MAPK8 | UGDH-AS1 | 0.430714 | 1.42E-25 |
| FOXO3 | AL117381.1 | 0.430735 | 1.41E-25 |
| CFLAR | AC087222.1 | 0.430746 | 1.40E-25 |
| ATG7 | AC011939.2 | 0.430757 | 1.40E-25 |
| SPNS1 | AL360181.2 | 0.430766 | 1.40E-25 |
| ATG16L2 | AC008969.1 | 0.430777 | 1.39E-25 |
| TSC1 | ATP1A1-AS1 | 0.430785 | 1.39E-25 |
| WDFY3 | AC004982.1 | 0.430788 | 1.39E-25 |
| PIK3C3 | SNHG26 | 0.430816 | 1.38E-25 |
| FOXO1 | AL355488.1 | 0.430862 | 1.36E-25 |
| ATG16L2 | AC008763.1 | 0.430867 | 1.36E-25 |
| BIRC6 | AC093297.2 | 0.430887 | 1.35E-25 |
| KLHL24 | GABPB1-AS1 | 0.430889 | 1.35E-25 |
| EIF2AK2 | AP003392.1 | 0.430937 | 1.33E-25 |
| ULK3 | AC009065.4 | 0.430947 | 1.33E-25 |
| NAF1 | AC009318.2 | 0.43099 | 1.31E-25 |
| TSC1 | EP300-AS1 | 0.430995 | 1.31E-25 |
| FOXO3 | AC010834.3 | 0.431008 | 1.30E-25 |
| RB1 | SAP30L-AS1 | 0.431025 | 1.30E-25 |
| CFLAR | LINC00886 | 0.431039 | 1.29E-25 |
| KLHL24 | AC087392.1 | 0.431057 | 1.28E-25 |
| TSC1 | AC055822.1 | 0.431076 | 1.28E-25 |
| SPNS1 | AC104564.3 | 0.431084 | 1.27E-25 |
| TSC1 | AL603839.3 | 0.43111 | 1.27E-25 |
| ATG2B | AC083862.2 | 0.431121 | 1.26E-25 |
| MAPK8 | HIF1A-AS2 | 0.431143 | 1.25E-25 |
| RB1 | AC124312.5 | 0.431155 | 1.25E-25 |
| RB1CC1 | AC018638.7 | 0.431178 | 1.24E-25 |
| KIF5B | MAL2-AS1 | 0.431183 | 1.24E-25 |
| ULK3 | AL390719.2 | 0.431212 | 1.23E-25 |
| RB1CC1 | AL122035.1 | 0.431224 | 1.22E-25 |
| ATG7 | ALG13-AS1 | 0.431229 | 1.22E-25 |
| FOXO1 | AL132989.1 | 0.431238 | 1.22E-25 |
| GOPC | AP001625.2 | 0.431259 | 1.21E-25 |
| FOXO1 | LINC00630 | 0.43126 | 1.21E-25 |
| PTEN | AC019131.2 | 0.431305 | 1.20E-25 |
| BIRC6 | LINC00426 | 0.431307 | 1.20E-25 |
| PTEN | AC025857.2 | 0.431314 | 1.19E-25 |
| FOXO3 | AC022150.4 | 0.431333 | 1.19E-25 |
| KIF5B | DLEU2 | 0.431342 | 1.18E-25 |
| RB1 | AC012467.1 | 0.431386 | 1.17E-25 |
| PIK3C3 | AC138956.2 | 0.431402 | 1.16E-25 |
| KLHL24 | AC005021.1 | 0.431417 | 1.16E-25 |
| RB1 | RAP2C-AS1 | 0.431426 | 1.16E-25 |
| RPS6KB1 | AC120349.1 | 0.43143 | 1.16E-25 |
| TSC1 | CFLAR-AS1 | 0.431446 | 1.15E-25 |
| ATG4B | AC106782.6 | 0.431455 | 1.15E-25 |
| RAB33B | GMDS-DT | 0.431476 | 1.14E-25 |
| KLHL24 | AL442125.2 | 0.431496 | 1.13E-25 |
| RPS6KB1 | OIP5-AS1 | 0.431512 | 1.13E-25 |
| ATG16L2 | LINC01011 | 0.43152 | 1.13E-25 |
| FOXO1 | AL442125.2 | 0.43153 | 1.12E-25 |
| CAPN10 | CTBP1-AS | 0.431537 | 1.12E-25 |
| RAB33B | DNM3OS | 0.431543 | 1.12E-25 |
| KLHL24 | AC018682.1 | 0.431561 | 1.11E-25 |
| NAF1 | OCIAD1-AS1 | 0.431565 | 1.11E-25 |
| KLHL24 | AC008969.1 | 0.431613 | 1.10E-25 |
| GOPC | AL080317.1 | 0.431619 | 1.09E-25 |
| FOXO1 | AP003170.3 | 0.431623 | 1.09E-25 |
| MAPK8 | AC037198.2 | 0.431627 | 1.09E-25 |
| MTOR | AC108010.1 | 0.431629 | 1.09E-25 |
| TSC1 | LINC00174 | 0.431632 | 1.09E-25 |
| RHEB | CYTOR | 0.431634 | 1.09E-25 |
| FOXO3 | AC037487.2 | 0.431637 | 1.09E-25 |
| UVRAG | AC024075.3 | 0.431654 | 1.08E-25 |
| ATG12 | AC018797.2 | 0.431672 | 1.08E-25 |
| CFLAR | AC022400.1 | 0.431684 | 1.07E-25 |
| FOXO3 | FAM13A-AS1 | 0.431694 | 1.07E-25 |
| TSC1 | CD44-AS1 | 0.431707 | 1.07E-25 |
| UVRAG | AC009120.3 | 0.431716 | 1.06E-25 |
| FOXO1 | AP000873.2 | 0.431717 | 1.06E-25 |
| ATG12 | JPX | 0.43172 | 1.06E-25 |
| ULK1 | AC027796.4 | 0.431773 | 1.05E-25 |
| UVRAG | AC116366.1 | 0.431817 | 1.03E-25 |
| EIF2AK2 | AC084871.1 | 0.431834 | 1.03E-25 |
| RB1 | AC018682.1 | 0.431902 | 1.01E-25 |
| EIF2AK2 | C1RL-AS1 | 0.431907 | 1.01E-25 |
| EIF2AK2 | AC011815.1 | 0.431915 | 1.01E-25 |
| RHEB | TDRKH-AS1 | 0.431918 | 1.01E-25 |
| IL24 | AL928742.1 | 0.431928 | 1.00E-25 |
| PIK3C3 | AL117336.2 | 0.431946 | 9.97E-26 |
| GOPC | RBMS3-AS3 | 0.431958 | 9.94E-26 |
| RPS6KB1 | AC012467.1 | 0.431963 | 9.92E-26 |
| RAB24 | AP006621.3 | 0.432048 | 9.68E-26 |
| UVRAG | AP000692.1 | 0.432061 | 9.65E-26 |
| BIRC6 | AC124045.1 | 0.432071 | 9.62E-26 |
| GOPC | LINC00861 | 0.432096 | 9.55E-26 |
| SPNS1 | AC139530.1 | 0.432105 | 9.53E-26 |
| NAF1 | MAGI2-AS3 | 0.432123 | 9.48E-26 |
| KLHL24 | AC093495.1 | 0.432128 | 9.47E-26 |
| ATG12 | AL606834.1 | 0.432143 | 9.42E-26 |
| ATG12 | AC008870.2 | 0.43215 | 9.41E-26 |
| RAB24 | AL390719.2 | 0.432158 | 9.38E-26 |
| ATG4B | AL359921.2 | 0.432163 | 9.37E-26 |
| MAPK8 | TBILA | 0.432179 | 9.33E-26 |
| MTOR | AP003392.4 | 0.432183 | 9.32E-26 |
| FOXO1 | AC007552.2 | 0.432191 | 9.30E-26 |
| RPS6KB1 | AL359921.1 | 0.432243 | 9.16E-26 |
| PTEN | AL031775.1 | 0.432279 | 9.07E-26 |
| TSC1 | AC005070.3 | 0.432289 | 9.04E-26 |
| RAB24 | AL031709.1 | 0.432294 | 9.03E-26 |
| RB1CC1 | AC007552.2 | 0.43234 | 8.91E-26 |
| FOXO3 | AL049840.2 | 0.432346 | 8.90E-26 |
| ATG2B | TBILA | 0.432347 | 8.89E-26 |
| TSC1 | CBR3-AS1 | 0.432365 | 8.85E-26 |
| ATG16L2 | AC026471.4 | 0.43242 | 8.71E-26 |
| ATG16L2 | AC040160.1 | 0.432448 | 8.64E-26 |
| ATG12 | MUC20-OT1 | 0.432452 | 8.63E-26 |
| PIK3C3 | AC026368.1 | 0.432457 | 8.62E-26 |
| PIK3C3 | AC020978.3 | 0.432501 | 8.51E-26 |
| GOPC | FMR1-IT1 | 0.432515 | 8.48E-26 |
| CFLAR | AC087392.1 | 0.432544 | 8.41E-26 |
| CAPNS1 | AC092295.2 | 0.432561 | 8.37E-26 |
| RB1CC1 | AC025287.3 | 0.432623 | 8.22E-26 |
| MAPK8 | AC083799.1 | 0.432631 | 8.20E-26 |
| RB1 | AL596325.2 | 0.432656 | 8.14E-26 |
| FOXO3 | AC005479.2 | 0.432682 | 8.08E-26 |
| CFLAR | AC139887.4 | 0.432684 | 8.08E-26 |
| TSC1 | AC087286.2 | 0.432684 | 8.08E-26 |
| RB1CC1 | AC084824.5 | 0.43273 | 7.97E-26 |
| KLHL24 | RAB30-AS1 | 0.432731 | 7.97E-26 |
| FOXO1 | AC124319.2 | 0.432731 | 7.97E-26 |
| GOPC | PCBP1-AS1 | 0.432752 | 7.92E-26 |
| ATG7 | AL035409.1 | 0.432776 | 7.87E-26 |
| IFNG | AC083862.2 | 0.432779 | 7.86E-26 |
| RB1CC1 | AC037198.1 | 0.432786 | 7.84E-26 |
| EIF2AK2 | AL590652.1 | 0.432797 | 7.82E-26 |
| TSC1 | LINC00115 | 0.43283 | 7.75E-26 |
| MAPK8 | AC004253.1 | 0.43285 | 7.70E-26 |
| GOPC | ADAMTSL4-AS1 | 0.432875 | 7.65E-26 |
| RB1 | AC006017.1 | 0.432878 | 7.64E-26 |
| EIF2AK2 | AC011477.3 | 0.432887 | 7.62E-26 |
| RPS6KB1 | AP000866.6 | 0.43289 | 7.61E-26 |
| ULK3 | AL513320.1 | 0.432891 | 7.61E-26 |
| PIK3C3 | AC004596.1 | 0.43292 | 7.55E-26 |
| RPS6KB1 | PCBP1-AS1 | 0.432984 | 7.41E-26 |
| MAPK8 | AC087222.1 | 0.433009 | 7.36E-26 |
| EIF2AK2 | AC125257.1 | 0.433018 | 7.34E-26 |
| ATG12 | GAS8-AS1 | 0.433032 | 7.31E-26 |
| GOPC | AL162724.2 | 0.43304 | 7.29E-26 |
| BIRC6 | AC121761.2 | 0.433066 | 7.24E-26 |
| TSC1 | CKMT2-AS1 | 0.433068 | 7.24E-26 |
| RPS6KB1 | AC087222.1 | 0.433085 | 7.20E-26 |
| GOPC | AL513008.1 | 0.43309 | 7.19E-26 |
| MAPK8 | ZKSCAN2-DT | 0.433097 | 7.18E-26 |
| PTEN | AL080317.1 | 0.4331 | 7.17E-26 |
| RB1 | RBMS3-AS3 | 0.433103 | 7.16E-26 |
| NAF1 | AC004477.3 | 0.433154 | 7.06E-26 |
| ATG16L2 | AC020594.1 | 0.433192 | 6.98E-26 |
| FOXO3 | AC048344.4 | 0.433201 | 6.97E-26 |
| SH3GLB1 | AC022150.4 | 0.433292 | 6.79E-26 |
| FOXO3 | AF129075.1 | 0.4333 | 6.77E-26 |
| CAPN10 | ZNF213-AS1 | 0.433308 | 6.76E-26 |
| PIK3C3 | AC022306.2 | 0.433322 | 6.73E-26 |
| ATG7 | AC007991.2 | 0.433342 | 6.69E-26 |
| NAF1 | AL450263.1 | 0.433349 | 6.68E-26 |
| ATG2B | AC009118.3 | 0.433349 | 6.68E-26 |
| NAF1 | AC067817.2 | 0.433356 | 6.66E-26 |
| PTEN | AC007406.5 | 0.433376 | 6.62E-26 |
| ATG2B | AP000442.2 | 0.433377 | 6.62E-26 |
| RB1 | AC005632.2 | 0.433405 | 6.57E-26 |
| ATG2B | HMGN3-AS1 | 0.433411 | 6.56E-26 |
| FOXO3 | AL049869.3 | 0.433433 | 6.52E-26 |
| DLC1 | SFTA1P | 0.433443 | 6.50E-26 |
| FOXO1 | AL021707.7 | 0.433451 | 6.48E-26 |
| ATG16L2 | AL683813.1 | 0.433472 | 6.44E-26 |
| RB1CC1 | AL031716.1 | 0.433476 | 6.44E-26 |
| CFLAR | AC008669.1 | 0.433486 | 6.42E-26 |
| FOXO3 | AC124283.3 | 0.433535 | 6.33E-26 |
| IFNG | AC008115.3 | 0.433564 | 6.28E-26 |
| MAPK8 | POLH-AS1 | 0.433602 | 6.21E-26 |
| CAPN10 | AL109811.3 | 0.433656 | 6.11E-26 |
| KLHL24 | AC004596.1 | 0.433656 | 6.11E-26 |
| TSC1 | AL160006.1 | 0.433663 | 6.10E-26 |
| WDFY3 | AC060766.7 | 0.433687 | 6.06E-26 |
| FOXO1 | AC066613.1 | 0.433704 | 6.03E-26 |
| GOPC | AC096992.2 | 0.433704 | 6.03E-26 |
| FOXO3 | AC092953.2 | 0.433726 | 5.99E-26 |
| ULK3 | AL139349.1 | 0.43373 | 5.99E-26 |
| ATG7 | DLEU2 | 0.43375 | 5.95E-26 |
| RB1 | MACC1-AS1 | 0.43376 | 5.93E-26 |
| RAB33B | RAP2C-AS1 | 0.433769 | 5.92E-26 |
| RPS6KB1 | AC027097.2 | 0.433772 | 5.91E-26 |
| MAP2K7 | AC011472.1 | 0.43379 | 5.88E-26 |
| RPS6KB1 | ERVK13-1 | 0.433802 | 5.86E-26 |
| TSC1 | AC116914.2 | 0.433826 | 5.82E-26 |
| RB1 | MIR29B2CHG | 0.433894 | 5.71E-26 |
| FOXO1 | AC025287.3 | 0.433917 | 5.67E-26 |
| ATG4B | AC110285.2 | 0.433925 | 5.66E-26 |
| ATG2B | DUBR | 0.433931 | 5.65E-26 |
| ATG7 | GK-IT1 | 0.433993 | 5.55E-26 |
| RAB24 | MHENCR | 0.434064 | 5.44E-26 |
| NAF1 | ARMCX5-GPRASP2 | 0.434098 | 5.38E-26 |
| FOXO1 | AC008537.2 | 0.434104 | 5.38E-26 |
| TSC1 | AC019080.5 | 0.434186 | 5.25E-26 |
| TSC2 | LINC00174 | 0.434202 | 5.23E-26 |
| GOPC | DLEU1 | 0.434229 | 5.19E-26 |
| KLHL24 | USP46-AS1 | 0.434242 | 5.17E-26 |
| ATG7 | LUCAT1 | 0.434251 | 5.15E-26 |
| TSC1 | AC006378.1 | 0.434259 | 5.14E-26 |
| UVRAG | PCBP1-AS1 | 0.434266 | 5.13E-26 |
| TSC1 | SCARNA9 | 0.4343 | 5.08E-26 |
| TSC1 | AL513008.1 | 0.434305 | 5.07E-26 |
| RB1 | AL078581.1 | 0.434306 | 5.07E-26 |
| EIF2AK2 | AL513327.1 | 0.434326 | 5.04E-26 |
| NLRC4 | AC013457.1 | 0.434345 | 5.02E-26 |
| NAF1 | MIR222HG | 0.434354 | 5.00E-26 |
| PIK3C3 | AL132780.1 | 0.434361 | 4.99E-26 |
| EIF2AK2 | USP46-AS1 | 0.434365 | 4.99E-26 |
| RB1 | AC010536.2 | 0.43437 | 4.98E-26 |
| EIF2AK2 | AC008537.2 | 0.434404 | 4.93E-26 |
| PTEN | NR2F1-AS1 | 0.434405 | 4.93E-26 |
| CCR2 | LINC00861 | 0.43441 | 4.92E-26 |
| FOXO1 | AC090617.5 | 0.434435 | 4.89E-26 |
| RB1CC1 | HIF1A-AS2 | 0.434436 | 4.89E-26 |
| MAPK8 | AP001628.1 | 0.434441 | 4.88E-26 |
| CFLAR | AC009948.4 | 0.434445 | 4.87E-26 |
| KLHL24 | AC005674.2 | 0.434455 | 4.86E-26 |
| GOPC | AC011472.4 | 0.434458 | 4.86E-26 |
| NAF1 | OSMR-AS1 | 0.434465 | 4.85E-26 |
| GOPC | AC012181.1 | 0.43448 | 4.82E-26 |
| EIF2AK2 | NORAD | 0.434502 | 4.79E-26 |
| RGS19 | USP30-AS1 | 0.434514 | 4.78E-26 |
| KLHL24 | AL359915.2 | 0.434531 | 4.75E-26 |
| EIF2AK2 | AC073534.1 | 0.434552 | 4.73E-26 |
| ATG12 | EBLN3P | 0.434617 | 4.64E-26 |
| RB1 | AL137782.1 | 0.43468 | 4.55E-26 |
| PIK3C3 | AL450263.1 | 0.434698 | 4.53E-26 |
| GOPC | AC009120.3 | 0.434701 | 4.53E-26 |
| ATG12 | LINC00426 | 0.434717 | 4.51E-26 |
| TSC1 | AC080162.1 | 0.434719 | 4.50E-26 |
| FAS | LINC00973 | 0.434727 | 4.49E-26 |
| RPS6KB1 | AC004253.1 | 0.434748 | 4.47E-26 |
| PIK3C3 | AC084824.5 | 0.434776 | 4.43E-26 |
| CFLAR | AP001107.4 | 0.434785 | 4.42E-26 |
| ATG4B | KMT2E-AS1 | 0.434806 | 4.39E-26 |
| CASP1 | AC147651.3 | 0.434818 | 4.38E-26 |
| RPS6KB1 | AC027277.2 | 0.434841 | 4.35E-26 |
| CCR2 | AC004585.1 | 0.434854 | 4.33E-26 |
| FOXO3 | AC012170.2 | 0.43486 | 4.33E-26 |
| ATG2B | AC027117.1 | 0.434865 | 4.32E-26 |
| CFLAR | SNHG14 | 0.434872 | 4.31E-26 |
| RPS6KB1 | AC092279.1 | 0.434875 | 4.31E-26 |
| RB1 | SP2-AS1 | 0.434884 | 4.29E-26 |
| GOPC | AL645568.1 | 0.434924 | 4.24E-26 |
| GOPC | AC073569.2 | 0.434979 | 4.18E-26 |
| IFNG | RRN3P2 | 0.434994 | 4.16E-26 |
| IFNG | AC090948.3 | 0.435007 | 4.14E-26 |
| PIK3C3 | AC093484.4 | 0.435014 | 4.14E-26 |
| WDFY3 | AC024909.2 | 0.435033 | 4.11E-26 |
| TSC1 | AC011472.4 | 0.435037 | 4.11E-26 |
| RB1CC1 | AC007991.2 | 0.435041 | 4.10E-26 |
| TSC1 | AC073896.3 | 0.435082 | 4.06E-26 |
| ATG2B | AC137932.3 | 0.435092 | 4.04E-26 |
| PEX14 | LINC00339 | 0.435093 | 4.04E-26 |
| FOXO3 | OIP5-AS1 | 0.435097 | 4.04E-26 |
| RB1CC1 | AC009090.3 | 0.435109 | 4.03E-26 |
| BIRC6 | AC079210.1 | 0.435111 | 4.02E-26 |
| CFLAR | AGBL5-IT1 | 0.435149 | 3.98E-26 |
| ATG7 | AC007684.1 | 0.435149 | 3.98E-26 |
| RB1CC1 | AC124312.2 | 0.435172 | 3.95E-26 |
| IFNG | AL022067.1 | 0.43519 | 3.93E-26 |
| FOXO1 | LANCL1-AS1 | 0.435197 | 3.92E-26 |
| NAF1 | ACTA2-AS1 | 0.435221 | 3.90E-26 |
| RB1CC1 | AL359921.1 | 0.435237 | 3.88E-26 |
| FOXO1 | AC105389.2 | 0.435246 | 3.87E-26 |
| CFLAR | ACTA2-AS1 | 0.435256 | 3.86E-26 |
| PTEN | AL049840.3 | 0.43527 | 3.84E-26 |
| TSC1 | LINC00630 | 0.435292 | 3.82E-26 |
| PIK3C3 | AC009090.3 | 0.435295 | 3.82E-26 |
| ATG12 | AP001625.2 | 0.435295 | 3.81E-26 |
| PTEN | AP003392.1 | 0.435299 | 3.81E-26 |
| GOPC | EP300-AS1 | 0.435301 | 3.81E-26 |
| RGS19 | PCED1B-AS1 | 0.435323 | 3.78E-26 |
| BIRC6 | AL512413.1 | 0.435366 | 3.74E-26 |
| ATG4B | AC011481.1 | 0.435392 | 3.71E-26 |
| ULK3 | U62317.2 | 0.435393 | 3.71E-26 |
| NAF1 | AL442125.2 | 0.43543 | 3.67E-26 |
| EIF2AK2 | AC008982.2 | 0.435434 | 3.66E-26 |
| NAF1 | AP001458.1 | 0.435459 | 3.64E-26 |
| WDFY3 | SRD5A3-AS1 | 0.43548 | 3.62E-26 |
| ATG4B | AC138028.4 | 0.435487 | 3.61E-26 |
| GABARAPL1 | AC026785.3 | 0.435487 | 3.61E-26 |
| ATG12 | AC092279.1 | 0.435495 | 3.60E-26 |
| EIF2AK3 | NORAD | 0.435502 | 3.59E-26 |
| NAF1 | AC027097.1 | 0.435517 | 3.58E-26 |
| RB1 | AC124312.4 | 0.435519 | 3.58E-26 |
| TSC1 | PTOV1-AS2 | 0.435533 | 3.56E-26 |
| NAF1 | AC011442.1 | 0.435565 | 3.53E-26 |
| FOXO3 | AC021078.1 | 0.435575 | 3.52E-26 |
| GOPC | AP003170.3 | 0.435603 | 3.49E-26 |
| CFLAR | MAGI2-AS3 | 0.435618 | 3.48E-26 |
| MAPK8 | AL606834.2 | 0.435668 | 3.43E-26 |
| RB1 | AC015911.3 | 0.43568 | 3.41E-26 |
| FOXO1 | AC010201.2 | 0.435683 | 3.41E-26 |
| SIRT1 | AC107068.1 | 0.435701 | 3.39E-26 |
| RB1 | AC007038.2 | 0.435737 | 3.36E-26 |
| RAB24 | AC093752.3 | 0.435751 | 3.34E-26 |
| PIK3C3 | AP001628.1 | 0.435763 | 3.33E-26 |
| ATG7 | Z68871.1 | 0.435789 | 3.31E-26 |
| CFLAR | AC009318.2 | 0.435806 | 3.29E-26 |
| WDFY3 | MGC32805 | 0.435815 | 3.28E-26 |
| ATG7 | AC092801.1 | 0.43582 | 3.28E-26 |
| WDFY3 | PDXDC2P-NPIPB14P | 0.43585 | 3.25E-26 |
| TSC1 | AC073842.2 | 0.435879 | 3.22E-26 |
| PIK3C3 | AL137003.2 | 0.435879 | 3.22E-26 |
| KLHL24 | SP2-AS1 | 0.436024 | 3.09E-26 |
| ATG4B | TNRC6C-AS1 | 0.43603 | 3.08E-26 |
| CFLAR | AC084824.4 | 0.436035 | 3.08E-26 |
| UVRAG | AC245014.3 | 0.436051 | 3.07E-26 |
| GOPC | AL157402.2 | 0.436057 | 3.06E-26 |
| DLC1 | AC079467.1 | 0.436089 | 3.03E-26 |
| NAF1 | AP000240.1 | 0.436099 | 3.02E-26 |
| NAF1 | AC112722.1 | 0.436134 | 2.99E-26 |
| ATG12 | MIR3936HG | 0.436161 | 2.97E-26 |
| ATG4B | AL008582.1 | 0.436181 | 2.95E-26 |
| NAF1 | AL121772.3 | 0.436216 | 2.92E-26 |
| TSC1 | LINC00861 | 0.436224 | 2.92E-26 |
| GOPC | AC018690.1 | 0.436235 | 2.91E-26 |
| FOXO3 | AF131215.5 | 0.436239 | 2.90E-26 |
| KLHL24 | AC074135.1 | 0.436296 | 2.86E-26 |
| RPS6KB1 | AP003170.3 | 0.436308 | 2.85E-26 |
| ATG7 | NEAT1 | 0.436339 | 2.82E-26 |
| PIK3C3 | AL021707.8 | 0.436378 | 2.79E-26 |
| RB1 | AC066613.1 | 0.436393 | 2.78E-26 |
| EIF2AK2 | AC025178.1 | 0.436396 | 2.77E-26 |
| PIK3C3 | LINC00861 | 0.436408 | 2.77E-26 |
| STK11 | AC069281.2 | 0.436421 | 2.75E-26 |
| GOPC | AC078846.1 | 0.436436 | 2.74E-26 |
| EIF2AK2 | AC020915.3 | 0.436439 | 2.74E-26 |
| CFLAR | AL132657.1 | 0.436468 | 2.72E-26 |
| FOXO1 | AC069023.1 | 0.436537 | 2.66E-26 |
| EIF2AK2 | AC135050.5 | 0.436572 | 2.64E-26 |
| FOXO1 | LINC02352 | 0.436575 | 2.64E-26 |
| RPS6KB1 | AC079907.1 | 0.436587 | 2.63E-26 |
| RB1CC1 | LINC00852 | 0.436598 | 2.62E-26 |
| KIF5B | AC073569.2 | 0.436611 | 2.61E-26 |
| SPNS1 | AL022328.1 | 0.436642 | 2.58E-26 |
| GOPC | AC026470.2 | 0.436653 | 2.58E-26 |
| PIK3C3 | AC234772.2 | 0.436673 | 2.56E-26 |
| TSC1 | AC087222.1 | 0.436682 | 2.55E-26 |
| RPS6KB1 | AL021707.8 | 0.436699 | 2.54E-26 |
| ATG7 | AC012557.1 | 0.436745 | 2.51E-26 |
| ATG12 | AC087222.1 | 0.436747 | 2.51E-26 |
| PIK3C3 | AC048341.1 | 0.436749 | 2.51E-26 |
| ATG2B | AC103591.3 | 0.436806 | 2.46E-26 |
| ULK3 | AL365330.1 | 0.436808 | 2.46E-26 |
| DAPK2 | AC106028.3 | 0.43682 | 2.45E-26 |
| RB1CC1 | AC098484.1 | 0.436844 | 2.44E-26 |
| FOXO1 | TMEM161B-AS1 | 0.436851 | 2.43E-26 |
| KLHL24 | AC037198.2 | 0.436863 | 2.42E-26 |
| PRKAR1A | AC015922.2 | 0.436905 | 2.39E-26 |
| RPS6KB1 | AC015871.3 | 0.43692 | 2.38E-26 |
| FOXO3 | AL512791.1 | 0.436936 | 2.37E-26 |
| FOXO3 | AC073651.1 | 0.436989 | 2.34E-26 |
| BIRC6 | AL133410.1 | 0.436997 | 2.33E-26 |
| PIK3C3 | AC090617.5 | 0.43702 | 2.32E-26 |
| BIRC6 | AC018645.2 | 0.437025 | 2.31E-26 |
| EIF2AK3 | AC073046.1 | 0.437051 | 2.30E-26 |
| VEGFA | AC132872.3 | 0.437082 | 2.27E-26 |
| FOXO3 | Z82243.1 | 0.437088 | 2.27E-26 |
| ATG16L2 | GEMIN7-AS1 | 0.437119 | 2.25E-26 |
| TSC1 | PTOV1-AS1 | 0.437122 | 2.25E-26 |
| UVRAG | AL513550.1 | 0.437149 | 2.23E-26 |
| CAPN10 | AP006621.3 | 0.437205 | 2.19E-26 |
| TSC1 | LINC01290 | 0.437231 | 2.18E-26 |
| CFLAR | TBILA | 0.437235 | 2.18E-26 |
| TSC1 | AC139887.4 | 0.437244 | 2.17E-26 |
| RB1 | AL157932.1 | 0.437267 | 2.16E-26 |
| ATG2B | AL021707.4 | 0.43728 | 2.15E-26 |
| CAMKK2 | AC068768.1 | 0.437396 | 2.08E-26 |
| MAPK8 | AC068792.1 | 0.437396 | 2.08E-26 |
| WDFY3 | AC124045.1 | 0.437438 | 2.05E-26 |
| ATG16L2 | AP003486.1 | 0.437516 | 2.01E-26 |
| RAB24 | AC012615.6 | 0.43754 | 1.99E-26 |
| ATG12 | RAB30-AS1 | 0.437559 | 1.98E-26 |
| ATG4B | AC003102.1 | 0.437564 | 1.98E-26 |
| EIF2AK2 | AC025171.3 | 0.437592 | 1.96E-26 |
| TSC1 | AC073957.3 | 0.437604 | 1.95E-26 |
| KIF5B | AC253536.3 | 0.437605 | 1.95E-26 |
| GOPC | AC068152.1 | 0.437606 | 1.95E-26 |
| RB1CC1 | AC006378.1 | 0.437636 | 1.94E-26 |
| EIF4EBP1 | AC109322.1 | 0.437673 | 1.92E-26 |
| TSC1 | AC011477.3 | 0.43768 | 1.91E-26 |
| FOXO3 | AC005046.1 | 0.437688 | 1.91E-26 |
| ATG7 | AL049840.2 | 0.4377 | 1.90E-26 |
| KLHL24 | AC007566.1 | 0.437718 | 1.89E-26 |
| WDFY3 | CBR3-AS1 | 0.437752 | 1.87E-26 |
| WDFY3 | AC026367.3 | 0.437753 | 1.87E-26 |
| PIK3C3 | AL031716.1 | 0.437754 | 1.87E-26 |
| RB1 | AL391834.1 | 0.437774 | 1.86E-26 |
| GOPC | AL662844.3 | 0.437802 | 1.85E-26 |
| GOPC | AC087392.1 | 0.437819 | 1.84E-26 |
| GOPC | AL512791.1 | 0.437821 | 1.84E-26 |
| RPS6KB1 | AC006378.1 | 0.437846 | 1.82E-26 |
| KLHL24 | AC073569.2 | 0.437847 | 1.82E-26 |
| FOXO3 | PAXBP1-AS1 | 0.437882 | 1.80E-26 |
| RPS6KB1 | AL606834.2 | 0.437902 | 1.79E-26 |
| TSC1 | AC107375.1 | 0.437904 | 1.79E-26 |
| ATG2B | AL132657.1 | 0.437945 | 1.77E-26 |
| ATG16L2 | AC133919.2 | 0.437961 | 1.76E-26 |
| RPS6KB1 | TMEM161B-AS1 | 0.437984 | 1.75E-26 |
| TSC1 | AL596325.2 | 0.437996 | 1.74E-26 |
| FOXO3 | LINC02035 | 0.438002 | 1.74E-26 |
| PIK3C3 | AL158212.3 | 0.438016 | 1.73E-26 |
| PTEN | CCDC18-AS1 | 0.438024 | 1.73E-26 |
| RAB33B | Z68871.1 | 0.438035 | 1.72E-26 |
| PIK3C3 | AL606834.2 | 0.438038 | 1.72E-26 |
| ATG7 | AC025857.2 | 0.438043 | 1.72E-26 |
| RPS6KB1 | AL731566.1 | 0.43805 | 1.72E-26 |
| RB1 | AC011477.2 | 0.438052 | 1.72E-26 |
| NAF1 | AC002553.1 | 0.438053 | 1.72E-26 |
| FOXO1 | AL137003.2 | 0.438054 | 1.71E-26 |
| RB1 | AC130650.2 | 0.438055 | 1.71E-26 |
| PIK3C3 | AC245884.8 | 0.438085 | 1.70E-26 |
| CFLAR | A2M-AS1 | 0.438099 | 1.69E-26 |
| CFLAR | AC004771.1 | 0.438111 | 1.69E-26 |
| MAPK8 | AC078883.1 | 0.438121 | 1.68E-26 |
| EIF2AK2 | AC018682.1 | 0.438125 | 1.68E-26 |
| RAC1 | AC091729.3 | 0.438138 | 1.67E-26 |
| PIK3C3 | FMR1-IT1 | 0.43814 | 1.67E-26 |
| RPS6KB1 | AC024075.3 | 0.438173 | 1.66E-26 |
| WDFY3 | AL121584.1 | 0.438174 | 1.66E-26 |
| ATG12 | LUCAT1 | 0.438192 | 1.65E-26 |
| MAP2K7 | AL513320.1 | 0.438204 | 1.64E-26 |
| GOPC | AP003486.1 | 0.43825 | 1.62E-26 |
| NAF1 | AC090617.5 | 0.438313 | 1.59E-26 |
| PTEN | EBLN3P | 0.438322 | 1.59E-26 |
| GOPC | AC005261.1 | 0.438335 | 1.58E-26 |
| CCR2 | FAM30A | 0.438371 | 1.56E-26 |
| CFLAR | AC107027.3 | 0.43839 | 1.55E-26 |
| FOXO3 | GAS5-AS1 | 0.438391 | 1.55E-26 |
| ATG12 | AC138207.4 | 0.438399 | 1.55E-26 |
| RB1CC1 | AC009120.3 | 0.438417 | 1.54E-26 |
| RPS6KB1 | EBLN3P | 0.438418 | 1.54E-26 |
| ATG7 | AL138963.1 | 0.438423 | 1.54E-26 |
| FOXO1 | AL513550.1 | 0.438466 | 1.52E-26 |
| EIF2AK2 | AC006213.4 | 0.438485 | 1.51E-26 |
| PIK3C3 | AC018926.2 | 0.438497 | 1.51E-26 |
| RPS6KB1 | AC027097.1 | 0.438537 | 1.49E-26 |
| NAF1 | AL031716.1 | 0.438556 | 1.48E-26 |
| GOPC | AL133342.1 | 0.438578 | 1.47E-26 |
| MAPK8 | AL031667.3 | 0.438592 | 1.47E-26 |
| ATG7 | LINC00216 | 0.438593 | 1.47E-26 |
| GOPC | AL354733.3 | 0.438611 | 1.46E-26 |
| TSC1 | AC135050.3 | 0.438629 | 1.45E-26 |
| PIK3C3 | AC008969.1 | 0.438632 | 1.45E-26 |
| EIF2AK2 | NARF-IT1 | 0.438644 | 1.44E-26 |
| TSC1 | TRAF3IP2-AS1 | 0.438646 | 1.44E-26 |
| FOXO1 | AC004477.3 | 0.438654 | 1.44E-26 |
| FOXO3 | CR936218.1 | 0.438659 | 1.44E-26 |
| RB1CC1 | AP000240.1 | 0.438692 | 1.42E-26 |
| NAF1 | AC096921.2 | 0.438761 | 1.40E-26 |
| ATG2B | AL158166.1 | 0.438771 | 1.39E-26 |
| PIK3R4 | AC068152.1 | 0.43878 | 1.39E-26 |
| FOXO1 | PCBP1-AS1 | 0.438791 | 1.38E-26 |
| ATG2B | AC026367.3 | 0.438812 | 1.37E-26 |
| BIRC6 | AC006504.8 | 0.438823 | 1.37E-26 |
| CAPN10 | AC104564.3 | 0.438837 | 1.36E-26 |
| TSC1 | AC002550.2 | 0.438845 | 1.36E-26 |
| TSC1 | AC025165.4 | 0.438882 | 1.35E-26 |
| ATG12 | AC135050.5 | 0.438905 | 1.34E-26 |
| PTEN | AC093726.2 | 0.438923 | 1.33E-26 |
| TSC1 | AC087286.1 | 0.43893 | 1.33E-26 |
| MAPK8 | AC135050.5 | 0.438932 | 1.33E-26 |
| ATG16L2 | AC005899.6 | 0.438982 | 1.31E-26 |
| ATG4B | AL021707.3 | 0.439007 | 1.30E-26 |
| GOPC | AL442125.2 | 0.439015 | 1.30E-26 |
| TSC1 | AC084018.1 | 0.439019 | 1.29E-26 |
| TSC1 | AC005899.6 | 0.439083 | 1.27E-26 |
| FOXO1 | AC135050.5 | 0.439084 | 1.27E-26 |
| NAF1 | UGDH-AS1 | 0.439094 | 1.27E-26 |
| GOPC | AL596325.2 | 0.4391 | 1.26E-26 |
| UVRAG | ARHGAP31-AS1 | 0.439122 | 1.26E-26 |
| MBTPS2 | Z68871.1 | 0.439131 | 1.25E-26 |
| FOXO3 | AC025171.3 | 0.439171 | 1.24E-26 |
| FOXO3 | AC011472.4 | 0.439185 | 1.23E-26 |
| GOPC | AC009041.4 | 0.439189 | 1.23E-26 |
| EIF2AK3 | USP46-AS1 | 0.439201 | 1.23E-26 |
| RPS6KB1 | A2M-AS1 | 0.439207 | 1.22E-26 |
| PIK3C3 | AC096921.2 | 0.439209 | 1.22E-26 |
| WDFY3 | LINC00893 | 0.43921 | 1.22E-26 |
| KLHL24 | AC083949.1 | 0.439257 | 1.21E-26 |
| WDFY3 | AL031775.1 | 0.439269 | 1.20E-26 |
| TSC1 | AC004832.5 | 0.439281 | 1.20E-26 |
| GOPC | AC092953.2 | 0.439291 | 1.20E-26 |
| FOXO1 | AC009090.1 | 0.439337 | 1.18E-26 |
| MTOR | AC024075.1 | 0.439345 | 1.18E-26 |
| FOXO1 | AC024075.1 | 0.439374 | 1.17E-26 |
| RPS6KB1 | RAP2C-AS1 | 0.439382 | 1.16E-26 |
| BNIP1 | ZSCAN16-AS1 | 0.439394 | 1.16E-26 |
| BIRC6 | TMEM9B-AS1 | 0.439424 | 1.15E-26 |
| FOXO1 | AC002553.1 | 0.439438 | 1.14E-26 |
| EIF2AK2 | AC005332.3 | 0.439439 | 1.14E-26 |
| RB1CC1 | AC025171.3 | 0.43946 | 1.14E-26 |
| RPS6KB1 | AL596325.2 | 0.439476 | 1.13E-26 |
| FOXO3 | RRN3P2 | 0.439482 | 1.13E-26 |
| MTOR | AC091057.1 | 0.439483 | 1.13E-26 |
| GOPC | LINC00852 | 0.439491 | 1.13E-26 |
| EIF2AK2 | AC007566.1 | 0.439524 | 1.12E-26 |
| RB1 | AL049552.1 | 0.439552 | 1.11E-26 |
| NAF1 | NFYC-AS1 | 0.43958 | 1.10E-26 |
| CFLAR | HLA-DQB1-AS1 | 0.43958 | 1.10E-26 |
| RB1CC1 | OCIAD1-AS1 | 0.439581 | 1.10E-26 |
| KLHL24 | ABALON | 0.439642 | 1.08E-26 |
| GOPC | AL157394.1 | 0.439674 | 1.07E-26 |
| TSC1 | AC011461.1 | 0.439681 | 1.07E-26 |
| NAF1 | AC090198.1 | 0.439701 | 1.06E-26 |
| ATG2B | AC080013.4 | 0.439708 | 1.06E-26 |
| CFLAR | AC109460.2 | 0.439713 | 1.06E-26 |
| RPS6KB1 | AC010201.2 | 0.439755 | 1.04E-26 |
| SPNS1 | AL022328.3 | 0.439769 | 1.04E-26 |
| FOXO1 | AC067852.3 | 0.439773 | 1.04E-26 |
| EDEM1 | OIP5-AS1 | 0.439785 | 1.03E-26 |
| ATG4B | AC022150.2 | 0.439872 | 1.01E-26 |
| GOPC | AC079684.1 | 0.439886 | 1.00E-26 |
| WDFY3 | AL136295.6 | 0.439906 | 9.98E-27 |
| TSC1 | AL021707.4 | 0.43993 | 9.91E-27 |
| RAB5A | NORAD | 0.439936 | 9.90E-27 |
| CAPN10 | CCDC183-AS1 | 0.439978 | 9.78E-27 |
| PIK3C3 | AP000240.1 | 0.440034 | 9.61E-27 |
| EIF2AK2 | AC002044.1 | 0.44005 | 9.57E-27 |
| FOXO3 | AC007038.1 | 0.44006 | 9.54E-27 |
| ATG12 | LINC01943 | 0.440061 | 9.54E-27 |
| NAF1 | AC139887.1 | 0.440086 | 9.47E-27 |
| GOPC | AL132780.1 | 0.440121 | 9.37E-27 |
| ATG16L2 | AL359921.2 | 0.440132 | 9.34E-27 |
| KLHL24 | AL122035.1 | 0.440142 | 9.32E-27 |
| PTEN | AC129510.1 | 0.440142 | 9.32E-27 |
| CFLAR | AP000240.1 | 0.440155 | 9.28E-27 |
| FOXO3 | AC100830.2 | 0.440178 | 9.22E-27 |
| ATG2B | AC006001.2 | 0.440202 | 9.15E-27 |
| NLRC4 | AC066613.1 | 0.440211 | 9.13E-27 |
| TSC1 | AC006059.1 | 0.440238 | 9.06E-27 |
| RPS6KB1 | AC138956.1 | 0.440272 | 8.97E-27 |
| WDFY3 | AC093227.1 | 0.440289 | 8.92E-27 |
| FOXO1 | AC011468.5 | 0.440292 | 8.91E-27 |
| LAMP1 | AC132192.2 | 0.440307 | 8.88E-27 |
| WDFY3 | AC121761.2 | 0.44032 | 8.84E-27 |
| GOPC | NDUFV2-AS1 | 0.440333 | 8.81E-27 |
| ATG16L2 | AC060780.1 | 0.440351 | 8.76E-27 |
| PIK3C3 | GAS8-AS1 | 0.440377 | 8.70E-27 |
| CFLAR | AC025165.4 | 0.440396 | 8.65E-27 |
| CFLAR | LINC02035 | 0.440402 | 8.63E-27 |
| RB1CC1 | AC010226.1 | 0.440425 | 8.57E-27 |
| UVRAG | AP000873.2 | 0.440435 | 8.55E-27 |
| NCKAP1 | AC073046.1 | 0.440456 | 8.50E-27 |
| NLRC4 | AL357060.1 | 0.440463 | 8.48E-27 |
| RB1 | LAMC1-AS1 | 0.440512 | 8.36E-27 |
| MAPK8 | LINC01004 | 0.440525 | 8.33E-27 |
| EIF2AK2 | AC090517.2 | 0.44053 | 8.31E-27 |
| GABARAPL2 | ENTPD3-AS1 | 0.440531 | 8.31E-27 |
| PIK3C3 | AC107027.3 | 0.440543 | 8.28E-27 |
| WDFY3 | AC091057.1 | 0.440558 | 8.25E-27 |
| ATG2B | AC137894.1 | 0.440587 | 8.18E-27 |
| RB1CC1 | EP300-AS1 | 0.440614 | 8.11E-27 |
| FOXO1 | AC080013.4 | 0.440617 | 8.10E-27 |
| ATG2B | AL109761.1 | 0.440624 | 8.09E-27 |
| RB1CC1 | AC133644.2 | 0.440681 | 7.95E-27 |
| ATG16L2 | LINC00926 | 0.4407 | 7.91E-27 |
| PTEN | XIST | 0.440705 | 7.90E-27 |
| RAB24 | AL139349.1 | 0.440707 | 7.89E-27 |
| ATG7 | AC004492.1 | 0.440719 | 7.87E-27 |
| RB1CC1 | AL121839.2 | 0.440751 | 7.79E-27 |
| NAF1 | AC015911.3 | 0.440753 | 7.79E-27 |
| FOXO3 | AP002336.2 | 0.440797 | 7.69E-27 |
| WDFY3 | AL513534.1 | 0.440803 | 7.67E-27 |
| MAPK8 | AL442125.2 | 0.440851 | 7.57E-27 |
| WDFY3 | LINC02100 | 0.440878 | 7.51E-27 |
| ATG2B | AP001271.1 | 0.440901 | 7.46E-27 |
| BIRC6 | AC139795.2 | 0.440914 | 7.43E-27 |
| EIF2AK2 | AC011442.1 | 0.440922 | 7.41E-27 |
| RPS6KB1 | AC004466.3 | 0.440945 | 7.36E-27 |
| EIF2AK2 | AC012181.2 | 0.440977 | 7.29E-27 |
| RB1 | ADAMTSL4-AS1 | 0.440979 | 7.29E-27 |
| MAPK8 | GARS-DT | 0.440981 | 7.28E-27 |
| MAPK8 | C1RL-AS1 | 0.440997 | 7.25E-27 |
| FOXO3 | AL139120.1 | 0.441001 | 7.24E-27 |
| BIRC6 | AC018797.2 | 0.441099 | 7.03E-27 |
| FOXO1 | AL353804.2 | 0.4411 | 7.03E-27 |
| RGS19 | AC147651.3 | 0.441135 | 6.96E-27 |
| UVRAG | AL645568.1 | 0.441136 | 6.96E-27 |
| PTEN | SH3BP5-AS1 | 0.441137 | 6.95E-27 |
| PRKCQ | TRG-AS1 | 0.441148 | 6.93E-27 |
| FOXO3 | PPP3CB-AS1 | 0.441158 | 6.91E-27 |
| NLRC4 | AC084871.1 | 0.441186 | 6.86E-27 |
| TP73 | MZF1-AS1 | 0.441223 | 6.78E-27 |
| EIF2AK2 | AL355075.2 | 0.441232 | 6.76E-27 |
| ATG10 | TMEM161B-AS1 | 0.44124 | 6.75E-27 |
| ATG7 | AC026202.2 | 0.441241 | 6.75E-27 |
| GOPC | AC130650.2 | 0.441249 | 6.73E-27 |
| GOPC | AC022400.5 | 0.441262 | 6.70E-27 |
| TSC1 | FTX | 0.441272 | 6.68E-27 |
| ATG7 | AL157402.2 | 0.441274 | 6.68E-27 |
| TSC1 | DGUOK-AS1 | 0.441281 | 6.67E-27 |
| PTEN | TAPT1-AS1 | 0.441364 | 6.51E-27 |
| RAB24 | AC007292.1 | 0.441391 | 6.46E-27 |
| FOXO1 | DLEU1 | 0.441413 | 6.41E-27 |
| BIRC6 | AC007406.5 | 0.441422 | 6.40E-27 |
| SPNS1 | AC132872.1 | 0.441437 | 6.37E-27 |
| RB1CC1 | PCBP1-AS1 | 0.441453 | 6.34E-27 |
| PIK3C3 | AL354733.3 | 0.441473 | 6.30E-27 |
| KLHL24 | AC084824.5 | 0.441496 | 6.26E-27 |
| NAF1 | AC020978.3 | 0.441517 | 6.22E-27 |
| ATG2B | AC004148.2 | 0.44154 | 6.18E-27 |
| PTEN | AC073896.3 | 0.441597 | 6.08E-27 |
| ATG7 | AC093799.1 | 0.441611 | 6.05E-27 |
| RPS6KB1 | C5orf56 | 0.441614 | 6.04E-27 |
| CFLAR | AP005899.1 | 0.441641 | 6.00E-27 |
| EIF2AK2 | RBMS3-AS3 | 0.441657 | 5.97E-27 |
| TSC1 | SNHG14 | 0.441676 | 5.93E-27 |
| RPS6KB1 | AL157394.1 | 0.441682 | 5.92E-27 |
| KIF5B | AL133243.2 | 0.441689 | 5.91E-27 |
| DAPK2 | AC009118.3 | 0.441732 | 5.84E-27 |
| ATG16L2 | AL118558.4 | 0.441802 | 5.72E-27 |
| TSC1 | AL133330.1 | 0.441886 | 5.58E-27 |
| TSC1 | NEAT1 | 0.441897 | 5.56E-27 |
| KLHL24 | AC025171.4 | 0.441914 | 5.53E-27 |
| DNAJB9 | LINC00324 | 0.441919 | 5.52E-27 |
| FOXO3 | AC022173.1 | 0.441935 | 5.50E-27 |
| GOPC | AC116366.1 | 0.442041 | 5.33E-27 |
| GOPC | AC010201.2 | 0.442068 | 5.29E-27 |
| TSC1 | AC093151.2 | 0.442099 | 5.24E-27 |
| CCR2 | LINC01150 | 0.442111 | 5.22E-27 |
| RB1CC1 | MIR181A2HG | 0.442113 | 5.22E-27 |
| GOPC | AC012467.1 | 0.442114 | 5.22E-27 |
| CAPN10 | AP001160.1 | 0.442142 | 5.17E-27 |
| RB1CC1 | AC026355.1 | 0.442144 | 5.17E-27 |
| NAF1 | AC096992.2 | 0.442175 | 5.12E-27 |
| FOXO1 | AL031666.1 | 0.442186 | 5.11E-27 |
| ATG12 | AP001528.2 | 0.442216 | 5.06E-27 |
| GOPC | ALMS1-IT1 | 0.442235 | 5.03E-27 |
| GOPC | SP2-AS1 | 0.442256 | 5.00E-27 |
| KLHL24 | ZNF32-AS2 | 0.442307 | 4.93E-27 |
| EIF2AK2 | AC007938.3 | 0.442317 | 4.91E-27 |
| RAB24 | AC008393.1 | 0.442328 | 4.90E-27 |
| FOXO3 | AL021578.1 | 0.442364 | 4.84E-27 |
| PIK3C3 | AL139011.1 | 0.442372 | 4.83E-27 |
| FOXO1 | AL049840.2 | 0.442389 | 4.81E-27 |
| ATG2B | AC048341.2 | 0.442431 | 4.75E-27 |
| RB1 | AL354696.1 | 0.442457 | 4.71E-27 |
| RPS6KB1 | AC005046.1 | 0.442481 | 4.68E-27 |
| RB1 | DUBR | 0.44249 | 4.67E-27 |
| UVRAG | RRN3P2 | 0.442506 | 4.65E-27 |
| ATG16L2 | AC011481.1 | 0.442539 | 4.60E-27 |
| ATG16L2 | THUMPD3-AS1 | 0.442543 | 4.60E-27 |
| CFLAR | AC097641.2 | 0.442543 | 4.59E-27 |
| UVRAG | LINC00630 | 0.442565 | 4.56E-27 |
| RB1 | AL049840.2 | 0.442589 | 4.53E-27 |
| GOPC | AC083843.2 | 0.442658 | 4.44E-27 |
| IL24 | AC243960.1 | 0.442696 | 4.39E-27 |
| WDFY3 | AC060780.1 | 0.442766 | 4.30E-27 |
| KIF5B | LINC00630 | 0.442767 | 4.30E-27 |
| TSC1 | AL109811.3 | 0.442781 | 4.28E-27 |
| RPS6KB1 | AL353804.2 | 0.442796 | 4.26E-27 |
| WDFY3 | MKLN1-AS | 0.442816 | 4.24E-27 |
| RPS6KB1 | ADNP-AS1 | 0.442842 | 4.21E-27 |
| CFLAR | AL163051.2 | 0.442864 | 4.18E-27 |
| TSC1 | AL138963.1 | 0.442876 | 4.16E-27 |
| TSC1 | AC010834.3 | 0.442897 | 4.14E-27 |
| PIK3C3 | AC005261.1 | 0.442916 | 4.11E-27 |
| PIK3C3 | DUBR | 0.442967 | 4.05E-27 |
| TSC1 | AC010883.1 | 0.443003 | 4.01E-27 |
| RPS6KB1 | AL132989.1 | 0.443007 | 4.01E-27 |
| TSC1 | AC087752.3 | 0.443009 | 4.00E-27 |
| GOPC | AC016542.1 | 0.44301 | 4.00E-27 |
| EIF2AK2 | GARS-DT | 0.443016 | 3.99E-27 |
| CAPN10 | AC132872.1 | 0.44302 | 3.99E-27 |
| ATG2B | AP001528.2 | 0.443117 | 3.88E-27 |
| SPNS1 | AL135999.1 | 0.44317 | 3.82E-27 |
| MAPK8 | AC010226.1 | 0.443184 | 3.80E-27 |
| NAF1 | AL359697.1 | 0.443199 | 3.78E-27 |
| WDFY3 | AC006213.4 | 0.443221 | 3.76E-27 |
| GOPC | AC008969.1 | 0.44323 | 3.75E-27 |
| PTEN | GEMIN7-AS1 | 0.443261 | 3.72E-27 |
| PTEN | AC005034.3 | 0.443263 | 3.71E-27 |
| UVRAG | AC234775.3 | 0.443265 | 3.71E-27 |
| FOXO3 | GMDS-DT | 0.443335 | 3.64E-27 |
| TSC1 | AC024933.1 | 0.443342 | 3.63E-27 |
| CFLAR | AF178030.1 | 0.443396 | 3.57E-27 |
| ATG7 | AL049840.1 | 0.443399 | 3.57E-27 |
| CASP4 | HCP5 | 0.443448 | 3.52E-27 |
| RB1CC1 | AP001458.1 | 0.443487 | 3.47E-27 |
| TSC1 | AF131215.5 | 0.443496 | 3.47E-27 |
| FAS | MIR222HG | 0.443519 | 3.44E-27 |
| RB1CC1 | ATP1B3-AS1 | 0.443534 | 3.43E-27 |
| GOPC | AL137003.2 | 0.443587 | 3.37E-27 |
| EDEM1 | RPS6KA2-IT1 | 0.443588 | 3.37E-27 |
| TSC1 | AC092794.1 | 0.443601 | 3.36E-27 |
| NLRC4 | AC093278.2 | 0.443656 | 3.31E-27 |
| FOXO3 | AC002553.2 | 0.443671 | 3.29E-27 |
| PIK3C3 | AC017100.1 | 0.443682 | 3.28E-27 |
| FOXO3 | AP000692.1 | 0.443762 | 3.20E-27 |
| CFLAR | MACC1-AS1 | 0.443778 | 3.19E-27 |
| GOPC | AC015911.3 | 0.443783 | 3.18E-27 |
| FOXO1 | AC068792.1 | 0.443804 | 3.16E-27 |
| CAPN10 | AC092171.4 | 0.443812 | 3.16E-27 |
| EIF2AK2 | AC087752.4 | 0.44387 | 3.10E-27 |
| RPS6KB1 | AC069023.1 | 0.443871 | 3.10E-27 |
| RB1CC1 | AC004908.2 | 0.443898 | 3.08E-27 |
| RB1 | AC078883.1 | 0.443901 | 3.07E-27 |
| RB1CC1 | LINC00894 | 0.443945 | 3.03E-27 |
| PRKAB1 | AC129507.3 | 0.443952 | 3.03E-27 |
| KIF5B | Z68871.1 | 0.443983 | 3.00E-27 |
| FOXO3 | AP002907.1 | 0.443987 | 3.00E-27 |
| KIF5B | AC107068.1 | 0.444003 | 2.98E-27 |
| EIF2AK2 | AC139887.4 | 0.444027 | 2.96E-27 |
| EIF2AK2 | ZNF32-AS2 | 0.444051 | 2.94E-27 |
| ATG2B | UBR5-AS1 | 0.444057 | 2.93E-27 |
| EIF2AK2 | AC073655.2 | 0.444059 | 2.93E-27 |
| ATG7 | OSMR-AS1 | 0.444067 | 2.93E-27 |
| PIK3C3 | AL157392.4 | 0.44407 | 2.92E-27 |
| TSC1 | AL117381.1 | 0.444079 | 2.91E-27 |
| TSC1 | AC011498.6 | 0.444081 | 2.91E-27 |
| ATG4B | AL513218.1 | 0.444111 | 2.89E-27 |
| ATG12 | AL442125.2 | 0.444117 | 2.88E-27 |
| FOXO1 | NUTM2A-AS1 | 0.444121 | 2.88E-27 |
| BIRC6 | AF131215.6 | 0.444152 | 2.85E-27 |
| PIK3C3 | AC004477.3 | 0.444162 | 2.84E-27 |
| EIF2AK2 | AC018638.7 | 0.444191 | 2.82E-27 |
| MAPK8 | ARMCX5-GPRASP2 | 0.444263 | 2.76E-27 |
| EIF2AK2 | AP001486.2 | 0.444264 | 2.76E-27 |
| ATG4B | SNHG20 | 0.444293 | 2.74E-27 |
| GOPC | CD44-AS1 | 0.444294 | 2.73E-27 |
| NAF1 | AL031666.1 | 0.444299 | 2.73E-27 |
| NAF1 | AC002044.1 | 0.444318 | 2.72E-27 |
| BIRC6 | AC131009.3 | 0.444323 | 2.71E-27 |
| FOXO3 | RHOA-IT1 | 0.444336 | 2.70E-27 |
| EIF2AK2 | AC037198.2 | 0.444402 | 2.65E-27 |
| ATG2B | AC073957.3 | 0.44443 | 2.63E-27 |
| MAPK8 | AC073569.2 | 0.444449 | 2.61E-27 |
| BIRC6 | UBR5-AS1 | 0.44447 | 2.59E-27 |
| ATG4B | AC022167.2 | 0.444478 | 2.59E-27 |
| MAPK8 | EP300-AS1 | 0.444478 | 2.59E-27 |
| TSC1 | LIMS1-AS1 | 0.444485 | 2.58E-27 |
| TSC1 | AC026367.3 | 0.444499 | 2.57E-27 |
| FOXO3 | AL391834.1 | 0.444531 | 2.55E-27 |
| KIF5B | AC108449.2 | 0.444605 | 2.49E-27 |
| MTOR | AC073569.2 | 0.444607 | 2.49E-27 |
| RPS6KB1 | AC009032.1 | 0.444617 | 2.48E-27 |
| ATG7 | AL513327.1 | 0.444638 | 2.47E-27 |
| RB1CC1 | AC138956.1 | 0.444643 | 2.47E-27 |
| ATG12 | NFYC-AS1 | 0.4447 | 2.42E-27 |
| RB1CC1 | AC025178.1 | 0.444735 | 2.40E-27 |
| EIF2AK2 | ATP1B3-AS1 | 0.444755 | 2.38E-27 |
| RPS6KB1 | AC079921.2 | 0.444756 | 2.38E-27 |
| NAF1 | AL354989.1 | 0.444765 | 2.38E-27 |
| NAF1 | AC090517.2 | 0.44477 | 2.37E-27 |
| FOXO1 | AC037198.2 | 0.444786 | 2.36E-27 |
| EIF2AK2 | EP300-AS1 | 0.444788 | 2.36E-27 |
| CFLAR | AC084117.1 | 0.444807 | 2.35E-27 |
| WDFY3 | NAALADL2-AS2 | 0.444836 | 2.33E-27 |
| MAPK8 | AC037198.1 | 0.444852 | 2.32E-27 |
| CFLAR | AC011472.4 | 0.444858 | 2.31E-27 |
| RPS6KB1 | AC009948.4 | 0.444858 | 2.31E-27 |
| ATG12 | AC097641.2 | 0.444875 | 2.30E-27 |
| NAF1 | AC138956.1 | 0.444882 | 2.30E-27 |
| PIK3C3 | AC007849.1 | 0.444886 | 2.29E-27 |
| EEF2K | AC024075.3 | 0.444892 | 2.29E-27 |
| KLHL24 | AL157838.1 | 0.444938 | 2.26E-27 |
| RB1 | AC099343.2 | 0.444978 | 2.23E-27 |
| ATG16L2 | AL049840.3 | 0.444988 | 2.22E-27 |
| BIRC6 | AC104819.3 | 0.44499 | 2.22E-27 |
| WDFY3 | AC090589.3 | 0.445009 | 2.21E-27 |
| WDFY3 | AL022323.1 | 0.445012 | 2.21E-27 |
| BIRC6 | CBR3-AS1 | 0.445014 | 2.21E-27 |
| FOXO3 | ALG13-AS1 | 0.44507 | 2.17E-27 |
| CFLAR | AC007991.2 | 0.445093 | 2.16E-27 |
| RPS6KB1 | AL157932.1 | 0.445102 | 2.15E-27 |
| PIK3C3 | AL021707.7 | 0.445113 | 2.14E-27 |
| MAPK8 | AP001469.3 | 0.445123 | 2.14E-27 |
| WDFY3 | AC109347.2 | 0.445143 | 2.12E-27 |
| ATG4B | AC026471.4 | 0.445165 | 2.11E-27 |
| MAP2K7 | AC020558.2 | 0.445178 | 2.10E-27 |
| CAPN10 | AL031600.1 | 0.445198 | 2.09E-27 |
| ATG4B | AP006284.1 | 0.445199 | 2.09E-27 |
| ATG7 | AC005838.2 | 0.44522 | 2.08E-27 |
| ATG2B | DGUOK-AS1 | 0.445227 | 2.07E-27 |
| WDR45 | ZNF674-AS1 | 0.445266 | 2.05E-27 |
| ATG12 | INE1 | 0.445277 | 2.04E-27 |
| MAPK8 | AC015813.1 | 0.445309 | 2.02E-27 |
| MAPK8 | AL157838.1 | 0.445356 | 1.99E-27 |
| RPS6KB1 | AL355075.2 | 0.445373 | 1.98E-27 |
| FOXO3 | AP001381.1 | 0.445391 | 1.97E-27 |
| CCR2 | AC104699.1 | 0.445403 | 1.97E-27 |
| TSC1 | AC026471.4 | 0.445405 | 1.96E-27 |
| PIK3C3 | AC005519.1 | 0.445408 | 1.96E-27 |
| GOPC | LINC01534 | 0.445415 | 1.96E-27 |
| RB1 | AC093388.1 | 0.445418 | 1.96E-27 |
| NAF1 | HIF1A-AS2 | 0.445422 | 1.95E-27 |
| RAB11A | LINC01559 | 0.445432 | 1.95E-27 |
| BIRC6 | AC083900.1 | 0.445454 | 1.94E-27 |
| NAF1 | AL137003.2 | 0.445464 | 1.93E-27 |
| KIF5B | USP46-AS1 | 0.445471 | 1.93E-27 |
| NAF1 | AC141002.1 | 0.445495 | 1.91E-27 |
| MAPK8 | AC015911.3 | 0.445496 | 1.91E-27 |
| GOPC | AC037487.2 | 0.445526 | 1.89E-27 |
| CFLAR | AC130895.1 | 0.445532 | 1.89E-27 |
| EIF2AK2 | AC011468.1 | 0.445577 | 1.87E-27 |
| RB1CC1 | AC139887.4 | 0.445594 | 1.86E-27 |
| FOXO3 | AL157392.3 | 0.44561 | 1.85E-27 |
| RPS6KB1 | AP000240.1 | 0.44561 | 1.85E-27 |
| RPS6KB1 | AC105389.2 | 0.445618 | 1.84E-27 |
| TSC1 | AL513534.1 | 0.445621 | 1.84E-27 |
| EIF2AK2 | AC005519.1 | 0.445624 | 1.84E-27 |
| CFLAR | SCAANT1 | 0.445629 | 1.84E-27 |
| NBR1 | AC005288.1 | 0.445676 | 1.81E-27 |
| GOPC | AC000123.1 | 0.445686 | 1.81E-27 |
| GOPC | LINC-PINT | 0.445763 | 1.77E-27 |
| FOXO3 | AL157402.2 | 0.445768 | 1.76E-27 |
| ATG7 | DNM3OS | 0.445793 | 1.75E-27 |
| FOXO1 | AC107027.3 | 0.4458 | 1.75E-27 |
| MAPK8 | AC073534.1 | 0.445825 | 1.73E-27 |
| FOXO1 | AL355075.2 | 0.445829 | 1.73E-27 |
| ATG12 | AC112496.1 | 0.445853 | 1.72E-27 |
| PIK3C3 | AC083949.1 | 0.445876 | 1.71E-27 |
| FOXO1 | C5orf56 | 0.44593 | 1.68E-27 |
| RB1CC1 | AC002553.1 | 0.445932 | 1.68E-27 |
| KLHL24 | AL132657.1 | 0.44595 | 1.67E-27 |
| PIK3C3 | AC138956.1 | 0.445952 | 1.67E-27 |
| IFNG | AC104699.1 | 0.445985 | 1.65E-27 |
| RPS6KB1 | LINC00852 | 0.446014 | 1.64E-27 |
| RB1 | AL122035.1 | 0.44603 | 1.63E-27 |
| CFLAR | NDUFV2-AS1 | 0.446043 | 1.62E-27 |
| RPS6KB1 | AC005332.6 | 0.446045 | 1.62E-27 |
| RB1 | AC073487.1 | 0.446065 | 1.61E-27 |
| ATG7 | FTX | 0.446077 | 1.61E-27 |
| ATG12 | AL031717.1 | 0.446124 | 1.58E-27 |
| UVRAG | LINC00861 | 0.446131 | 1.58E-27 |
| RB1 | SDCBP2-AS1 | 0.446211 | 1.54E-27 |
| MAPK8 | AL158166.1 | 0.446214 | 1.54E-27 |
| EIF2AK2 | AL353804.2 | 0.446227 | 1.54E-27 |
| ATG7 | TRG-AS1 | 0.446326 | 1.49E-27 |
| PIK3R4 | LINC02035 | 0.446338 | 1.49E-27 |
| GOPC | MIR29B2CHG | 0.446345 | 1.48E-27 |
| ATG4B | AP006621.4 | 0.446432 | 1.45E-27 |
| NAF1 | AC079684.1 | 0.446443 | 1.44E-27 |
| HIF1A | AL357060.1 | 0.446457 | 1.43E-27 |
| GOPC | AL353804.2 | 0.446461 | 1.43E-27 |
| RB1CC1 | STARD4-AS1 | 0.446477 | 1.43E-27 |
| NAF1 | GABPB1-AS1 | 0.446482 | 1.42E-27 |
| ATG2B | AC124016.2 | 0.446516 | 1.41E-27 |
| ULK3 | AP003419.3 | 0.446541 | 1.40E-27 |
| ATG4B | AC006942.1 | 0.446573 | 1.39E-27 |
| CAPN10 | AC245140.2 | 0.446574 | 1.38E-27 |
| ATG12 | GK-AS1 | 0.446579 | 1.38E-27 |
| PIK3C3 | MIR181A2HG | 0.446616 | 1.37E-27 |
| TSC1 | AL162724.2 | 0.446625 | 1.36E-27 |
| KLHL24 | OSMR-AS1 | 0.446644 | 1.36E-27 |
| TSC1 | AL390728.6 | 0.446647 | 1.35E-27 |
| CAPN10 | AL135999.1 | 0.446689 | 1.34E-27 |
| RB1CC1 | AL139011.1 | 0.446712 | 1.33E-27 |
| CAPN10 | AL031709.1 | 0.446714 | 1.33E-27 |
| RAB24 | LINC01786 | 0.446722 | 1.32E-27 |
| CTSB | LINC01150 | 0.446728 | 1.32E-27 |
| ATG7 | AC053513.1 | 0.446729 | 1.32E-27 |
| PTEN | AC025171.2 | 0.446735 | 1.32E-27 |
| KLHL24 | AC007938.3 | 0.446753 | 1.31E-27 |
| EIF2AK2 | AL606489.1 | 0.446764 | 1.31E-27 |
| NAF1 | NDUFV2-AS1 | 0.446802 | 1.29E-27 |
| BIRC6 | AC012467.2 | 0.446822 | 1.29E-27 |
| ATG12 | AC011442.1 | 0.446841 | 1.28E-27 |
| FOXO3 | AC093110.1 | 0.446863 | 1.27E-27 |
| PTEN | AP001330.5 | 0.447026 | 1.21E-27 |
| LAMP1 | U91328.1 | 0.447076 | 1.19E-27 |
| TSC1 | AL359915.2 | 0.447078 | 1.19E-27 |
| FOXO3 | SDCBP2-AS1 | 0.447134 | 1.17E-27 |
| EIF2AK2 | AC067817.2 | 0.447135 | 1.17E-27 |
| TSC1 | AL645568.1 | 0.447142 | 1.17E-27 |
| ATG12 | AC092953.2 | 0.447148 | 1.17E-27 |
| RAB24 | ARRDC1-AS1 | 0.447168 | 1.16E-27 |
| MAPK8 | AC016727.1 | 0.447221 | 1.14E-27 |
| MAPK8 | RBMS3-AS3 | 0.447242 | 1.13E-27 |
| TSC1 | AC244197.2 | 0.447242 | 1.13E-27 |
| TSC2 | AC073335.2 | 0.447244 | 1.13E-27 |
| TSC1 | AC005856.1 | 0.447246 | 1.13E-27 |
| ATG4B | AL450384.2 | 0.447333 | 1.10E-27 |
| ATG2B | MIR155HG | 0.447342 | 1.10E-27 |
| EIF2AK2 | AP003486.1 | 0.447361 | 1.09E-27 |
| GOPC | AC078883.1 | 0.447381 | 1.09E-27 |
| ATG12 | AC113139.1 | 0.447426 | 1.07E-27 |
| EIF2AK2 | AL117336.2 | 0.447431 | 1.07E-27 |
| WDFY3 | SNHG16 | 0.447437 | 1.07E-27 |
| TSC1 | AC253536.6 | 0.447467 | 1.06E-27 |
| TSC1 | AC084824.4 | 0.44748 | 1.06E-27 |
| GOPC | AL162724.1 | 0.447494 | 1.05E-27 |
| PIK3C3 | GARS-DT | 0.447531 | 1.04E-27 |
| GOPC | AC004884.2 | 0.447539 | 1.04E-27 |
| ATG7 | AC131971.1 | 0.447585 | 1.02E-27 |
| NAF1 | AC009041.4 | 0.447595 | 1.02E-27 |
| TSC2 | LINC00265 | 0.447599 | 1.02E-27 |
| MAPK8 | HMGN3-AS1 | 0.447617 | 1.01E-27 |
| TSC1 | AC253576.2 | 0.447677 | 9.95E-28 |
| KLHL24 | AL360219.1 | 0.447681 | 9.93E-28 |
| MAPK8 | AC022306.2 | 0.447692 | 9.90E-28 |
| FOXO3 | BTBD9-AS1 | 0.447714 | 9.84E-28 |
| RAB24 | MIR210HG | 0.447727 | 9.80E-28 |
| ATG4B | LENG8-AS1 | 0.44773 | 9.79E-28 |
| GOPC | AC010536.2 | 0.447752 | 9.73E-28 |
| RAB11A | AL049555.1 | 0.447761 | 9.70E-28 |
| RPS6KB1 | AC013403.2 | 0.447793 | 9.61E-28 |
| PIK3C3 | AC026356.1 | 0.4478 | 9.59E-28 |
| ATG2B | AC011477.3 | 0.447801 | 9.58E-28 |
| MAPK8 | LINC01389 | 0.447828 | 9.51E-28 |
| RAB33B | SMC5-AS1 | 0.447832 | 9.50E-28 |
| RB1CC1 | AL157786.1 | 0.447845 | 9.46E-28 |
| GOPC | AC026202.2 | 0.447865 | 9.40E-28 |
| MAPK8 | AC015871.3 | 0.447874 | 9.38E-28 |
| FOXO1 | AC074033.1 | 0.447905 | 9.29E-28 |
| HGS | TNRC6C-AS1 | 0.447981 | 9.08E-28 |
| FOXO3 | LINC00216 | 0.44799 | 9.05E-28 |
| NCKAP1 | AC073569.2 | 0.447997 | 9.03E-28 |
| CCR2 | LINC00582 | 0.448017 | 8.98E-28 |
| EIF2AK2 | AC245884.8 | 0.448021 | 8.97E-28 |
| GOPC | STARD4-AS1 | 0.448084 | 8.80E-28 |
| HGS | AC005332.5 | 0.448117 | 8.71E-28 |
| RPS6KB1 | AP001458.1 | 0.44812 | 8.71E-28 |
| WDFY3 | AC073957.3 | 0.448159 | 8.60E-28 |
| GOPC | PPP3CB-AS1 | 0.448163 | 8.59E-28 |
| CAPN10 | AL031714.1 | 0.448163 | 8.59E-28 |
| ATG16L2 | AL035563.1 | 0.448171 | 8.57E-28 |
| MAPK8 | LINC01534 | 0.448176 | 8.56E-28 |
| ATG16L2 | AL031186.1 | 0.448194 | 8.52E-28 |
| WDFY3 | AC007406.5 | 0.448328 | 8.18E-28 |
| WDFY3 | AL049840.4 | 0.448342 | 8.14E-28 |
| RPS6KB1 | HIF1A-AS2 | 0.448356 | 8.11E-28 |
| RB1CC1 | AL353804.2 | 0.448366 | 8.08E-28 |
| TSC1 | AC005332.3 | 0.448476 | 7.82E-28 |
| ATG12 | AL357060.1 | 0.448524 | 7.71E-28 |
| NAF1 | AC011676.1 | 0.448524 | 7.71E-28 |
| TSC1 | AC010615.2 | 0.448526 | 7.70E-28 |
| EIF2AK2 | AL021707.8 | 0.448592 | 7.55E-28 |
| EIF2AK2 | AC016542.1 | 0.44862 | 7.49E-28 |
| CASP1 | AC243960.1 | 0.448635 | 7.45E-28 |
| NAF1 | AL606834.1 | 0.448637 | 7.45E-28 |
| TSC1 | AL049869.3 | 0.448658 | 7.40E-28 |
| EIF2AK2 | AC009041.4 | 0.448712 | 7.28E-28 |
| CFLAR | AC074032.1 | 0.448734 | 7.23E-28 |
| GOPC | AL158212.3 | 0.448772 | 7.15E-28 |
| PTEN | AL445493.3 | 0.448783 | 7.13E-28 |
| RB1 | AP000866.6 | 0.448784 | 7.13E-28 |
| EIF2AK2 | DLEU1 | 0.448786 | 7.12E-28 |
| KLHL24 | LINC01290 | 0.448815 | 7.06E-28 |
| NAF1 | AC005046.1 | 0.448826 | 7.04E-28 |
| IFNG | AC080013.4 | 0.448834 | 7.02E-28 |
| TSC2 | AL360181.2 | 0.448842 | 7.00E-28 |
| TSC1 | AC068790.2 | 0.448844 | 7.00E-28 |
| RPS6KB1 | AL360219.1 | 0.448845 | 7.00E-28 |
| GOPC | AC005046.1 | 0.448854 | 6.98E-28 |
| RAB24 | SLC9A3-AS1 | 0.448866 | 6.95E-28 |
| ATG12 | AC068792.1 | 0.448867 | 6.95E-28 |
| TSC2 | AC012615.6 | 0.44889 | 6.90E-28 |
| CFLAR | KDM4A-AS1 | 0.448904 | 6.87E-28 |
| RAB33B | UGDH-AS1 | 0.448906 | 6.87E-28 |
| GOPC | AL031775.2 | 0.448914 | 6.85E-28 |
| ATG4B | AC002398.1 | 0.448924 | 6.83E-28 |
| KLHL24 | AL606834.1 | 0.448971 | 6.74E-28 |
| WDFY3 | AC103591.3 | 0.448996 | 6.69E-28 |
| RB1CC1 | LINC01290 | 0.449017 | 6.64E-28 |
| RB1CC1 | AC004241.3 | 0.449021 | 6.63E-28 |
| ATG2B | AC002563.1 | 0.449082 | 6.51E-28 |
| RPS6KB1 | AC087752.4 | 0.449097 | 6.49E-28 |
| ATG7 | AC114760.2 | 0.449099 | 6.48E-28 |
| RPS6KB1 | AC007991.2 | 0.449103 | 6.47E-28 |
| TSC1 | AC018752.1 | 0.449103 | 6.47E-28 |
| TSC1 | AC068790.3 | 0.44913 | 6.42E-28 |
| TSC2 | AP006621.4 | 0.449133 | 6.41E-28 |
| MAPK8 | AL021707.8 | 0.449186 | 6.31E-28 |
| EIF2AK2 | MUC20-OT1 | 0.449257 | 6.18E-28 |
| NLRC4 | AC037198.2 | 0.449261 | 6.17E-28 |
| MAPK8 | AC053527.1 | 0.449283 | 6.13E-28 |
| FOXO3 | AL139407.1 | 0.449314 | 6.07E-28 |
| WDFY3 | ZFPM2-AS1 | 0.449353 | 6.00E-28 |
| RB1CC1 | AC009054.2 | 0.449419 | 5.88E-28 |
| FOXO3 | Z83843.1 | 0.449426 | 5.87E-28 |
| KLHL24 | AC115989.1 | 0.449453 | 5.82E-28 |
| GOPC | AC005920.2 | 0.449491 | 5.76E-28 |
| MAPK8 | AL359962.2 | 0.449596 | 5.58E-28 |
| GOPC | AL391834.1 | 0.44967 | 5.45E-28 |
| ATG16L2 | AC010973.2 | 0.449675 | 5.45E-28 |
| KLHL24 | AC004656.1 | 0.449698 | 5.41E-28 |
| FOXO3 | AL590723.1 | 0.449774 | 5.28E-28 |
| KLHL24 | AC097641.2 | 0.449783 | 5.27E-28 |
| EIF2AK2 | OCIAD1-AS1 | 0.449842 | 5.18E-28 |
| TSC1 | AC007216.3 | 0.449849 | 5.17E-28 |
| KLHL24 | ZNF460-AS1 | 0.449957 | 5.00E-28 |
| PIK3C3 | EP300-AS1 | 0.449969 | 4.98E-28 |
| MAPK8IP1 | MIAT | 0.449999 | 4.94E-28 |
| TSC1 | AC104463.2 | 0.450023 | 4.90E-28 |
| TSC2 | AL139349.1 | 0.450029 | 4.89E-28 |
| PIK3R4 | NORAD | 0.450059 | 4.85E-28 |
| WDFY3 | AL133410.1 | 0.450073 | 4.83E-28 |
| RPS6KB1 | AL132780.1 | 0.450089 | 4.80E-28 |
| RPS6KB1 | ATP1B3-AS1 | 0.45009 | 4.80E-28 |
| ATG16L2 | UBE2Q1-AS1 | 0.4501 | 4.79E-28 |
| FOXO3 | AC097376.2 | 0.45015 | 4.71E-28 |
| ATG2B | AC020571.1 | 0.450153 | 4.71E-28 |
| TSC2 | AL928654.2 | 0.450161 | 4.70E-28 |
| RB1 | AC110792.3 | 0.450163 | 4.70E-28 |
| CFLAR | AC037198.1 | 0.450186 | 4.66E-28 |
| RB1 | AC253576.2 | 0.45019 | 4.66E-28 |
| PTEN | AC006504.8 | 0.450213 | 4.63E-28 |
| ATG12 | AC007938.3 | 0.45029 | 4.52E-28 |
| ATG16L2 | AC137932.3 | 0.450302 | 4.50E-28 |
| TSC1 | AC003070.1 | 0.450366 | 4.42E-28 |
| GOPC | AC009318.3 | 0.450371 | 4.41E-28 |
| RB1 | AC115989.1 | 0.45039 | 4.38E-28 |
| MAPK8 | AC139887.1 | 0.450405 | 4.36E-28 |
| CFLAR | AL117336.2 | 0.450411 | 4.36E-28 |
| ATG2B | THUMPD3-AS1 | 0.450426 | 4.34E-28 |
| ATG2B | AC006042.1 | 0.45043 | 4.33E-28 |
| CFLAR | AC009948.1 | 0.450443 | 4.31E-28 |
| FOXO3 | AC020913.3 | 0.450479 | 4.27E-28 |
| NAF1 | AL359715.3 | 0.450489 | 4.26E-28 |
| ATG7 | SCARNA9 | 0.450498 | 4.24E-28 |
| FOXO1 | AC139887.4 | 0.450505 | 4.23E-28 |
| FOXO1 | AF131215.5 | 0.450537 | 4.19E-28 |
| CAPN10 | AL022328.4 | 0.450571 | 4.15E-28 |
| RB1 | AC096586.2 | 0.450602 | 4.11E-28 |
| KLHL24 | ZKSCAN2-DT | 0.450609 | 4.10E-28 |
| ATG16L2 | AC018653.3 | 0.450664 | 4.03E-28 |
| RB1CC1 | AC011472.4 | 0.45067 | 4.03E-28 |
| FOXO3 | AC124312.5 | 0.450675 | 4.02E-28 |
| TSC1 | ADNP-AS1 | 0.450677 | 4.02E-28 |
| PIK3C3 | AC011472.4 | 0.450679 | 4.02E-28 |
| KLHL24 | AL512791.1 | 0.450693 | 4.00E-28 |
| ATG16L2 | AC005726.3 | 0.450741 | 3.94E-28 |
| FOXO3 | AL022067.1 | 0.450745 | 3.94E-28 |
| CFLAR | RBMS3-AS3 | 0.450779 | 3.90E-28 |
| TSC1 | NUTM2B-AS1 | 0.450801 | 3.87E-28 |
| PIK3C3 | AC009318.2 | 0.450811 | 3.86E-28 |
| RB1CC1 | AC087392.1 | 0.450819 | 3.85E-28 |
| NAF1 | LINC00894 | 0.45082 | 3.85E-28 |
| TSC1 | AC120349.1 | 0.450863 | 3.80E-28 |
| FOXO1 | ATP13A4-AS1 | 0.450879 | 3.78E-28 |
| CASP1 | MIR155HG | 0.450944 | 3.71E-28 |
| TSC1 | AL049840.5 | 0.450979 | 3.67E-28 |
| PTEN | AC093227.1 | 0.45098 | 3.67E-28 |
| TSC1 | AL359076.1 | 0.451009 | 3.63E-28 |
| ATG4B | AC135050.3 | 0.451036 | 3.60E-28 |
| RB1CC1 | AL031666.1 | 0.451043 | 3.60E-28 |
| RB1CC1 | AC120349.1 | 0.451062 | 3.57E-28 |
| MAPK8 | AC005540.1 | 0.451069 | 3.57E-28 |
| RB1 | AC009054.2 | 0.451086 | 3.55E-28 |
| RPS6KB1 | AC009318.2 | 0.451101 | 3.53E-28 |
| TSC1 | AC004908.3 | 0.451124 | 3.51E-28 |
| KLHL24 | AL021707.8 | 0.451133 | 3.50E-28 |
| RPS6KB1 | THAP9-AS1 | 0.451143 | 3.49E-28 |
| ATG16L2 | AC010245.2 | 0.451216 | 3.41E-28 |
| WDFY3 | AC083900.1 | 0.451224 | 3.40E-28 |
| RB1CC1 | UGDH-AS1 | 0.451243 | 3.38E-28 |
| ATG16L2 | AC011461.1 | 0.451284 | 3.34E-28 |
| KLHL24 | FLNB-AS1 | 0.451313 | 3.31E-28 |
| UVRAG | AC090948.2 | 0.451332 | 3.29E-28 |
| MAPK8 | AL031666.1 | 0.451341 | 3.28E-28 |
| PIK3C3 | AC012181.2 | 0.451353 | 3.27E-28 |
| CFLAR | ARHGAP27P1-BPTFP1-KPNA2P3 | 0.451373 | 3.25E-28 |
| RB1 | PSPC1-AS2 | 0.451384 | 3.24E-28 |
| MAPK8 | USP46-AS1 | 0.451387 | 3.24E-28 |
| RB1CC1 | RPS6KA2-IT1 | 0.451414 | 3.21E-28 |
| NAF1 | NUTM2A-AS1 | 0.451424 | 3.20E-28 |
| RB1 | AF129075.1 | 0.451431 | 3.20E-28 |
| ATG12 | MGC32805 | 0.451467 | 3.16E-28 |
| WDFY3 | AC018797.2 | 0.451493 | 3.14E-28 |
| HGS | AC067852.2 | 0.451523 | 3.11E-28 |
| ATG2B | AC068792.1 | 0.451527 | 3.10E-28 |
| ATG16L2 | AP000254.1 | 0.451549 | 3.08E-28 |
| FOXO3 | AC096741.1 | 0.451583 | 3.05E-28 |
| CFLAR | HCG18 | 0.451632 | 3.01E-28 |
| ATG16L2 | AP003392.1 | 0.451661 | 2.98E-28 |
| NAF1 | AC011815.1 | 0.451666 | 2.97E-28 |
| ATG16L2 | BACE1-AS | 0.451682 | 2.96E-28 |
| MAPK8 | AC009318.3 | 0.451689 | 2.95E-28 |
| RPS6KB1 | ABALON | 0.451698 | 2.95E-28 |
| FOXO3 | AC004466.3 | 0.451706 | 2.94E-28 |
| PIK3C3 | RFX3-AS1 | 0.451731 | 2.92E-28 |
| GOPC | AC006017.1 | 0.45174 | 2.91E-28 |
| EIF2AK3 | EBLN3P | 0.451787 | 2.87E-28 |
| WDFY3 | AC104083.1 | 0.451788 | 2.87E-28 |
| TSC1 | AL031673.1 | 0.451794 | 2.86E-28 |
| FOXO1 | Z98884.2 | 0.451801 | 2.86E-28 |
| FOXO3 | NEAT1 | 0.451809 | 2.85E-28 |
| ATG16L2 | AC025171.5 | 0.451833 | 2.83E-28 |
| RPS6KB1 | AC004908.2 | 0.451841 | 2.82E-28 |
| ATG7 | AC124319.2 | 0.451854 | 2.81E-28 |
| PTEN | GPRC5D-AS1 | 0.451861 | 2.80E-28 |
| TSC1 | AL590652.1 | 0.451895 | 2.77E-28 |
| WDFY3 | AC125257.1 | 0.451898 | 2.77E-28 |
| FOXO1 | Z68871.1 | 0.451905 | 2.77E-28 |
| PIK3C3 | AC009318.3 | 0.45195 | 2.73E-28 |
| RAB33B | AC098484.1 | 0.451952 | 2.73E-28 |
| TSC2 | AC022167.2 | 0.45196 | 2.72E-28 |
| ATG7 | AL136320.1 | 0.451973 | 2.71E-28 |
| GOPC | MBNL1-AS1 | 0.451992 | 2.69E-28 |
| ULK3 | AC087741.1 | 0.452016 | 2.67E-28 |
| FOXO1 | AC012467.1 | 0.452021 | 2.67E-28 |
| MAPK8 | AC026367.3 | 0.452028 | 2.66E-28 |
| GOPC | AC138207.4 | 0.452031 | 2.66E-28 |
| TSC1 | AC074117.1 | 0.452089 | 2.61E-28 |
| PTEN | AC022784.5 | 0.452118 | 2.59E-28 |
| ATG16L2 | AC093110.1 | 0.452142 | 2.57E-28 |
| TSC1 | AC027097.2 | 0.452173 | 2.55E-28 |
| RPS6KB1 | AC093799.1 | 0.452191 | 2.53E-28 |
| RPS6KB1 | AC074032.1 | 0.452193 | 2.53E-28 |
| KLHL24 | AP001625.2 | 0.452209 | 2.52E-28 |
| RPS6KB1 | AL513550.1 | 0.452212 | 2.52E-28 |
| NAF1 | FLNB-AS1 | 0.452217 | 2.51E-28 |
| ATG2B | AC004596.1 | 0.452229 | 2.51E-28 |
| CFLAR | ARHGEF38-IT1 | 0.452233 | 2.50E-28 |
| EIF2AK2 | AL359921.1 | 0.452251 | 2.49E-28 |
| RPS6KB1 | AC011477.2 | 0.452256 | 2.49E-28 |
| IKBKB | AC068594.1 | 0.452259 | 2.48E-28 |
| ATG2B | AC048382.2 | 0.45227 | 2.47E-28 |
| ATG7 | AC005070.3 | 0.452288 | 2.46E-28 |
| RB1CC1 | AC010536.2 | 0.452293 | 2.46E-28 |
| PIK3C3 | AC242426.2 | 0.452341 | 2.42E-28 |
| RPS6KB1 | TRAF3IP2-AS1 | 0.452344 | 2.42E-28 |
| EIF2AK2 | AC016394.1 | 0.452437 | 2.35E-28 |
| KLHL24 | AL157392.4 | 0.452463 | 2.33E-28 |
| TSC1 | AC015911.3 | 0.452466 | 2.33E-28 |
| EIF2AK2 | AC004067.1 | 0.452468 | 2.33E-28 |
| PTEN | AC006213.4 | 0.452474 | 2.32E-28 |
| GOPC | AL133330.1 | 0.452504 | 2.30E-28 |
| FOXO3 | A2M-AS1 | 0.452507 | 2.30E-28 |
| ATG16L2 | AC095057.3 | 0.452513 | 2.30E-28 |
| ARSA | BX537318.1 | 0.452593 | 2.24E-28 |
| PIK3C3 | PSMA3-AS1 | 0.4526 | 2.24E-28 |
| GOPC | AL137779.2 | 0.452608 | 2.23E-28 |
| NAF1 | AC084871.1 | 0.452666 | 2.19E-28 |
| CFLAR | THAP9-AS1 | 0.452715 | 2.16E-28 |
| PRKCQ | LINC00426 | 0.452729 | 2.15E-28 |
| TSC1 | SOS1-IT1 | 0.452733 | 2.15E-28 |
| WDFY3 | LUCAT1 | 0.452745 | 2.14E-28 |
| GOPC | AC087284.1 | 0.452758 | 2.13E-28 |
| RB1CC1 | AL662844.3 | 0.45276 | 2.13E-28 |
| KLHL24 | AGAP1-IT1 | 0.452769 | 2.12E-28 |
| RPS6KB1 | AC024933.1 | 0.452792 | 2.11E-28 |
| RB1CC1 | AC141002.1 | 0.452795 | 2.11E-28 |
| GOPC | AC009948.1 | 0.452796 | 2.11E-28 |
| MAPK8 | AC005021.1 | 0.45281 | 2.10E-28 |
| KLHL24 | AC087752.3 | 0.452841 | 2.08E-28 |
| PTEN | AC025178.1 | 0.452858 | 2.07E-28 |
| TSC1 | AC004923.4 | 0.452858 | 2.07E-28 |
| GOPC | AC124312.4 | 0.45292 | 2.03E-28 |
| TSC2 | AL139287.1 | 0.452979 | 1.99E-28 |
| NAF1 | AC234772.2 | 0.453004 | 1.98E-28 |
| PIK3C3 | MBNL1-AS1 | 0.453015 | 1.97E-28 |
| NAF1 | AC011468.5 | 0.453047 | 1.95E-28 |
| EIF2AK2 | MIATNB | 0.453054 | 1.95E-28 |
| TSC1 | AC022306.2 | 0.453097 | 1.92E-28 |
| ATG7 | AC004918.3 | 0.453105 | 1.92E-28 |
| MAP2K7 | AL022328.2 | 0.453127 | 1.90E-28 |
| GOPC | KDM4A-AS1 | 0.453179 | 1.87E-28 |
| GOPC | MACC1-AS1 | 0.453208 | 1.86E-28 |
| GOPC | AC025287.3 | 0.453234 | 1.84E-28 |
| FOXO1 | AC022211.1 | 0.45327 | 1.82E-28 |
| ATG16L2 | AL021878.2 | 0.453319 | 1.80E-28 |
| WDFY3 | GPRC5D-AS1 | 0.453319 | 1.80E-28 |
| GOPC | AL513534.1 | 0.453336 | 1.79E-28 |
| PTEN | AC068152.1 | 0.45337 | 1.77E-28 |
| PIK3C3 | AC008669.1 | 0.453406 | 1.75E-28 |
| UVRAG | AC008124.1 | 0.453427 | 1.74E-28 |
| ATG4B | AL035461.2 | 0.453458 | 1.72E-28 |
| ATG12 | SCAANT1 | 0.453469 | 1.71E-28 |
| RPS6KB1 | AC234772.2 | 0.453512 | 1.69E-28 |
| CFLAR | ZKSCAN2-DT | 0.453515 | 1.69E-28 |
| CFLAR | AC007552.2 | 0.453551 | 1.67E-28 |
| CFLAR | LINC00893 | 0.453601 | 1.65E-28 |
| WDFY3 | AL603839.3 | 0.453613 | 1.64E-28 |
| FOXO3 | AC092801.1 | 0.453679 | 1.61E-28 |
| NAF1 | AC011472.4 | 0.45373 | 1.58E-28 |
| PIK3C3 | AL138921.2 | 0.453739 | 1.58E-28 |
| CFLAR | HMGA1P4 | 0.453742 | 1.58E-28 |
| RB1 | AC007216.4 | 0.453818 | 1.54E-28 |
| TSC1 | AC004477.3 | 0.45384 | 1.53E-28 |
| RB1 | AL162724.2 | 0.453853 | 1.52E-28 |
| BAG1 | MYO16-AS1 | 0.453854 | 1.52E-28 |
| ATG2B | AC112496.1 | 0.453867 | 1.52E-28 |
| CFLAR | AC242426.2 | 0.453877 | 1.51E-28 |
| CFLAR | AC093799.1 | 0.453896 | 1.50E-28 |
| ATG12 | AC024075.1 | 0.453904 | 1.50E-28 |
| CCR2 | USP30-AS1 | 0.453905 | 1.50E-28 |
| TSC1 | AC007546.1 | 0.453908 | 1.50E-28 |
| FOXO3 | FTX | 0.453931 | 1.49E-28 |
| RB1 | AC026470.2 | 0.453944 | 1.48E-28 |
| PELP1 | AL355802.2 | 0.453953 | 1.48E-28 |
| ATF6 | OIP5-AS1 | 0.453954 | 1.48E-28 |
| ATG12 | AC016957.2 | 0.453967 | 1.47E-28 |
| TSC1 | AC139100.2 | 0.453972 | 1.47E-28 |
| FOXO1 | AC116366.1 | 0.453972 | 1.47E-28 |
| GOPC | AC002064.2 | 0.453983 | 1.46E-28 |
| CFLAR | OCIAD1-AS1 | 0.454022 | 1.45E-28 |
| MAPK8 | AC107027.3 | 0.454051 | 1.43E-28 |
| MAPK8 | UBR5-AS1 | 0.45412 | 1.40E-28 |
| GOPC | AC027097.2 | 0.454123 | 1.40E-28 |
| BIRC6 | RUSC1-AS1 | 0.454146 | 1.39E-28 |
| MAPK8 | AC011468.1 | 0.454156 | 1.39E-28 |
| GOPC | AC007014.2 | 0.454167 | 1.38E-28 |
| PTEN | LINC00426 | 0.454174 | 1.38E-28 |
| EIF2AK2 | AC009237.15 | 0.454175 | 1.38E-28 |
| KLHL24 | AC024560.3 | 0.454184 | 1.38E-28 |
| EIF2AK2 | AC011468.5 | 0.454229 | 1.36E-28 |
| TSC1 | AC005838.2 | 0.454244 | 1.35E-28 |
| EIF2AK2 | SAMD12-AS1 | 0.45425 | 1.35E-28 |
| FOXO1 | AC037198.1 | 0.454283 | 1.34E-28 |
| IKBKB | ARHGAP27P1-BPTFP1-KPNA2P3 | 0.454365 | 1.30E-28 |
| PTEN | AC073655.2 | 0.454371 | 1.30E-28 |
| SAR1A | NUTM2A-AS1 | 0.454374 | 1.30E-28 |
| RPS6KB1 | AC087286.1 | 0.454398 | 1.29E-28 |
| NAF1 | FAM160A1-DT | 0.454398 | 1.29E-28 |
| RB1CC1 | AL442125.2 | 0.454464 | 1.26E-28 |
| FOXO3 | AL133445.2 | 0.454466 | 1.26E-28 |
| PIK3C3 | AC096992.2 | 0.454506 | 1.25E-28 |
| CFLAR | AC010834.3 | 0.454508 | 1.25E-28 |
| RPS6KB1 | AC004241.3 | 0.454518 | 1.24E-28 |
| KLHL24 | LINC01376 | 0.454522 | 1.24E-28 |
| PIK3C3 | AC008537.2 | 0.454592 | 1.21E-28 |
| GOPC | AL158166.2 | 0.454604 | 1.21E-28 |
| FOXO3 | AC009090.3 | 0.454607 | 1.21E-28 |
| MAPK8 | TPT1-AS1 | 0.454622 | 1.20E-28 |
| TSC1 | AC107068.1 | 0.454628 | 1.20E-28 |
| KLHL24 | AC008537.2 | 0.454702 | 1.17E-28 |
| RB1CC1 | AC092953.2 | 0.454706 | 1.17E-28 |
| KLHL24 | OCIAD1-AS1 | 0.454708 | 1.17E-28 |
| CCR2 | LINC01215 | 0.454769 | 1.15E-28 |
| MAPK8 | AC006378.1 | 0.454791 | 1.14E-28 |
| RAB33B | AC097376.2 | 0.454822 | 1.13E-28 |
| NAF1 | KDM4A-AS1 | 0.454835 | 1.13E-28 |
| FOXO1 | GABPB1-AS1 | 0.454837 | 1.13E-28 |
| FOXO3 | CFLAR-AS1 | 0.454838 | 1.13E-28 |
| ATG7 | RHOA-IT1 | 0.454883 | 1.11E-28 |
| NAF1 | AC022400.5 | 0.454964 | 1.08E-28 |
| RB1CC1 | AL354733.3 | 0.454966 | 1.08E-28 |
| RAB24 | AC245140.2 | 0.454967 | 1.08E-28 |
| TSC1 | AL021578.1 | 0.454982 | 1.08E-28 |
| NAF1 | LINC01534 | 0.455013 | 1.07E-28 |
| ATG16L2 | ASB16-AS1 | 0.455061 | 1.05E-28 |
| RB1CC1 | AC011676.1 | 0.455075 | 1.05E-28 |
| ATG12 | AP001469.2 | 0.455076 | 1.05E-28 |
| GOPC | TPT1-AS1 | 0.455087 | 1.04E-28 |
| FOXO1 | AC084117.1 | 0.455088 | 1.04E-28 |
| BIRC6 | AL136295.6 | 0.455099 | 1.04E-28 |
| GOPC | AC008966.2 | 0.455112 | 1.04E-28 |
| CFLAR | AL355488.1 | 0.455141 | 1.03E-28 |
| ATG7 | AC087286.2 | 0.455164 | 1.02E-28 |
| MAPK8 | AC007406.5 | 0.455166 | 1.02E-28 |
| PIK3C3 | Z98884.2 | 0.455184 | 1.01E-28 |
| ATG12 | AC079907.1 | 0.455205 | 1.01E-28 |
| RAB24 | LINC00174 | 0.455234 | 9.97E-29 |
| PIK3C3 | MAGI2-AS3 | 0.455275 | 9.84E-29 |
| RB1CC1 | AC116366.1 | 0.455285 | 9.81E-29 |
| GOPC | AC093788.1 | 0.455352 | 9.61E-29 |
| FOXO3 | AC008966.2 | 0.455376 | 9.54E-29 |
| ATG7 | AP001432.1 | 0.4554 | 9.47E-29 |
| RPS6KB1 | AC025178.1 | 0.455421 | 9.41E-29 |
| PIK3C3 | AL359915.2 | 0.455427 | 9.39E-29 |
| RB1 | LINC00513 | 0.45543 | 9.38E-29 |
| PIK3C3 | AC018809.2 | 0.455493 | 9.20E-29 |
| NAF1 | AC069023.1 | 0.455521 | 9.13E-29 |
| PTEN | AC020915.3 | 0.45557 | 8.99E-29 |
| EIF2AK2 | AC010536.2 | 0.455586 | 8.94E-29 |
| TSC2 | Z69706.1 | 0.455658 | 8.75E-29 |
| EIF2AK2 | AC009090.3 | 0.455663 | 8.73E-29 |
| WDFY3 | THAP9-AS1 | 0.455666 | 8.72E-29 |
| WDFY3 | AC010615.2 | 0.455675 | 8.70E-29 |
| RPS6KB1 | AL662844.3 | 0.455677 | 8.70E-29 |
| FOXO3 | AL133243.2 | 0.455725 | 8.57E-29 |
| KLHL24 | AC016542.1 | 0.455727 | 8.56E-29 |
| PIK3C3 | LINC00894 | 0.455745 | 8.52E-29 |
| RPS6KB1 | AL513008.1 | 0.455758 | 8.48E-29 |
| WDFY3 | AC012467.2 | 0.455766 | 8.46E-29 |
| MAPK8 | AL157392.4 | 0.455905 | 8.11E-29 |
| TSC1 | AC024560.3 | 0.455919 | 8.07E-29 |
| ATG7 | AC007878.1 | 0.455938 | 8.02E-29 |
| PIK3C3 | AC022211.1 | 0.455961 | 7.97E-29 |
| ATG2B | AC006213.4 | 0.455986 | 7.90E-29 |
| CFLAR | AP000866.6 | 0.456001 | 7.87E-29 |
| PIK3C3 | AC139887.2 | 0.456011 | 7.84E-29 |
| EIF2AK2 | AC234772.2 | 0.456028 | 7.80E-29 |
| PTEN | AL359962.1 | 0.45603 | 7.80E-29 |
| CFLAR | CTBP1-AS | 0.456056 | 7.74E-29 |
| PIK3C3 | AL512791.1 | 0.456061 | 7.72E-29 |
| TSC1 | AC104564.3 | 0.456071 | 7.70E-29 |
| RB1 | AC026356.1 | 0.456078 | 7.68E-29 |
| WDFY3 | AC018645.2 | 0.456198 | 7.40E-29 |
| RB1 | HCG18 | 0.456201 | 7.40E-29 |
| TSC1 | AC027277.2 | 0.456203 | 7.39E-29 |
| TSC1 | AL365330.1 | 0.456243 | 7.30E-29 |
| NAF1 | AL512791.1 | 0.456265 | 7.25E-29 |
| GOPC | MALAT1 | 0.456269 | 7.24E-29 |
| FAS | MIR155HG | 0.456273 | 7.23E-29 |
| FOXO3 | NPTN-IT1 | 0.456312 | 7.15E-29 |
| RB1 | NEAT1 | 0.456318 | 7.13E-29 |
| PIK3C3 | AL391001.1 | 0.45635 | 7.06E-29 |
| GOPC | AL157932.1 | 0.456391 | 6.97E-29 |
| MAPK8 | SAMD12-AS1 | 0.456416 | 6.92E-29 |
| EIF2AK2 | AL596325.2 | 0.456429 | 6.89E-29 |
| MAPK8 | AC092953.2 | 0.45643 | 6.89E-29 |
| ATG2B | AL928654.2 | 0.456434 | 6.88E-29 |
| TSC1 | AC008906.1 | 0.456453 | 6.84E-29 |
| RB1 | AC027277.2 | 0.45647 | 6.81E-29 |
| ATG12 | AC087752.4 | 0.456489 | 6.77E-29 |
| TSC1 | Z83843.1 | 0.45651 | 6.72E-29 |
| CFLAR | AC006270.1 | 0.456538 | 6.66E-29 |
| FOXO1 | AL137782.1 | 0.45655 | 6.64E-29 |
| CAPN10 | U62317.2 | 0.45658 | 6.58E-29 |
| RPS6KB1 | RRN3P2 | 0.456598 | 6.54E-29 |
| FOXO3 | AC005920.2 | 0.45661 | 6.52E-29 |
| FOXO1 | HIF1A-AS2 | 0.456621 | 6.49E-29 |
| ATG7 | AC104695.3 | 0.456639 | 6.46E-29 |
| TSC1 | AL031709.1 | 0.45664 | 6.46E-29 |
| FOXO3 | AC010761.3 | 0.456662 | 6.41E-29 |
| RAB24 | AC009065.4 | 0.456759 | 6.22E-29 |
| PTEN | AC015813.1 | 0.456765 | 6.21E-29 |
| ATG7 | AC124283.3 | 0.456776 | 6.19E-29 |
| ATG16L2 | TNRC6C-AS1 | 0.456781 | 6.18E-29 |
| FOXO3 | AC007216.4 | 0.456784 | 6.17E-29 |
| ATG2B | AC078883.1 | 0.456787 | 6.17E-29 |
| NAF1 | AP001160.4 | 0.456807 | 6.13E-29 |
| FOXO3 | AC006059.1 | 0.456807 | 6.13E-29 |
| KLHL24 | AC090517.2 | 0.456888 | 5.98E-29 |
| KLHL24 | AC007849.1 | 0.456899 | 5.96E-29 |
| KLHL24 | AL355075.2 | 0.456908 | 5.94E-29 |
| FOXO3 | AC090948.2 | 0.456924 | 5.91E-29 |
| TSC1 | EBLN3P | 0.45701 | 5.76E-29 |
| TSC1 | AC012557.1 | 0.457015 | 5.75E-29 |
| FOXO3 | AC090948.1 | 0.457016 | 5.75E-29 |
| GOPC | AC099343.2 | 0.457043 | 5.70E-29 |
| WDFY3 | AL021707.4 | 0.457063 | 5.66E-29 |
| RB1 | AF117829.1 | 0.457103 | 5.59E-29 |
| KLHL24 | AC009120.3 | 0.457121 | 5.56E-29 |
| NAF1 | RRN3P2 | 0.457121 | 5.56E-29 |
| TSC1 | AC110792.3 | 0.457122 | 5.56E-29 |
| ATG12 | AC009120.2 | 0.457126 | 5.55E-29 |
| FOXO3 | AC063965.1 | 0.457131 | 5.55E-29 |
| ATG16L2 | TNFRSF14-AS1 | 0.457147 | 5.52E-29 |
| PIK3R4 | LINC00630 | 0.457175 | 5.47E-29 |
| ATG2B | CBR3-AS1 | 0.457186 | 5.45E-29 |
| KLHL24 | AC009090.3 | 0.457189 | 5.45E-29 |
| MAPK8 | AC009120.3 | 0.457204 | 5.42E-29 |
| CFLAR | AC011676.1 | 0.457216 | 5.40E-29 |
| EIF2AK2 | AC091057.1 | 0.457217 | 5.40E-29 |
| GOPC | AC011477.2 | 0.457368 | 5.15E-29 |
| TSC1 | AC004466.3 | 0.457436 | 5.04E-29 |
| ATG2B | AC011676.1 | 0.457465 | 5.00E-29 |
| TSC1 | AC062037.2 | 0.45747 | 4.99E-29 |
| TSC1 | AC093297.2 | 0.457472 | 4.99E-29 |
| PIK3C3 | LINC01376 | 0.457497 | 4.95E-29 |
| KLHL24 | AL359921.1 | 0.457525 | 4.91E-29 |
| RB1CC1 | LINC01376 | 0.457529 | 4.90E-29 |
| GOPC | AC253576.2 | 0.457532 | 4.90E-29 |
| PIK3C3 | AC007319.1 | 0.457541 | 4.88E-29 |
| EIF2AK2 | LINC01534 | 0.457552 | 4.87E-29 |
| MAPK8 | MUC20-OT1 | 0.457587 | 4.81E-29 |
| PIK3C3 | AC005046.1 | 0.457624 | 4.76E-29 |
| KLHL24 | AL049840.2 | 0.457629 | 4.75E-29 |
| RB1 | Z68871.1 | 0.457639 | 4.74E-29 |
| GAPDH | LINC00941 | 0.45766 | 4.70E-29 |
| RB1 | DNM3OS | 0.457664 | 4.70E-29 |
| EIF4EBP1 | AP003119.2 | 0.457676 | 4.68E-29 |
| WDFY3 | AC004241.3 | 0.457726 | 4.61E-29 |
| MAPK8 | TMEM161B-AS1 | 0.457754 | 4.57E-29 |
| NAF1 | AC074033.1 | 0.457786 | 4.52E-29 |
| RB1 | AL157394.1 | 0.45784 | 4.45E-29 |
| RB1 | AC104695.3 | 0.457861 | 4.42E-29 |
| KLHL24 | LINC01389 | 0.457883 | 4.39E-29 |
| RB1 | CD44-AS1 | 0.457888 | 4.38E-29 |
| TP63 | LINC00885 | 0.457904 | 4.36E-29 |
| MAPK8 | AC026355.1 | 0.457978 | 4.26E-29 |
| ATG7 | AC108727.1 | 0.457989 | 4.25E-29 |
| BIRC6 | AC103591.3 | 0.457994 | 4.24E-29 |
| GOPC | AC124312.2 | 0.458017 | 4.21E-29 |
| FOXO3 | EP300-AS1 | 0.458034 | 4.19E-29 |
| WDFY3 | LINC01876 | 0.458038 | 4.18E-29 |
| GOPC | AL731566.1 | 0.458054 | 4.16E-29 |
| ATG7 | AL590723.1 | 0.458078 | 4.13E-29 |
| MAPK8 | AL359921.1 | 0.458108 | 4.09E-29 |
| RB1CC1 | AGAP1-IT1 | 0.458109 | 4.09E-29 |
| BIRC6 | ZFPM2-AS1 | 0.458111 | 4.09E-29 |
| RB1CC1 | AL606834.2 | 0.458147 | 4.04E-29 |
| PIK3C3 | AC018638.7 | 0.458156 | 4.03E-29 |
| BIRC6 | AC109460.2 | 0.458158 | 4.03E-29 |
| BIRC6 | AL603839.3 | 0.458174 | 4.01E-29 |
| GOPC | AF117829.1 | 0.458185 | 4.00E-29 |
| PIK3C3 | AC079684.1 | 0.458196 | 3.98E-29 |
| RPS6KB1 | AC037198.1 | 0.45822 | 3.95E-29 |
| GOPC | AC090579.1 | 0.458247 | 3.92E-29 |
| RB1CC1 | NARF-IT1 | 0.45826 | 3.90E-29 |
| FAS | LINC01094 | 0.458272 | 3.89E-29 |
| FOXO3 | AC007684.1 | 0.458282 | 3.88E-29 |
| TSC1 | AP001271.1 | 0.458293 | 3.86E-29 |
| KLHL24 | AC135050.5 | 0.458295 | 3.86E-29 |
| NAF1 | AC008969.1 | 0.458312 | 3.84E-29 |
| PTEN | AC008735.4 | 0.45832 | 3.83E-29 |
| GOPC | FLNB-AS1 | 0.45832 | 3.83E-29 |
| KLHL24 | AC005104.1 | 0.458326 | 3.82E-29 |
| KLHL24 | AC083799.1 | 0.458327 | 3.82E-29 |
| EIF2AK2 | AC011472.4 | 0.458349 | 3.80E-29 |
| RPS6KB1 | INE1 | 0.458356 | 3.79E-29 |
| RPS6KB1 | AC083843.2 | 0.458462 | 3.67E-29 |
| RB1 | AP005131.7 | 0.45847 | 3.66E-29 |
| KLHL24 | NARF-IT1 | 0.458476 | 3.65E-29 |
| WDFY3 | AF131215.6 | 0.458488 | 3.64E-29 |
| GOPC | AC021078.1 | 0.458505 | 3.62E-29 |
| NLRC4 | RRN3P2 | 0.458522 | 3.60E-29 |
| PIK3C3 | ZNF790-AS1 | 0.458523 | 3.60E-29 |
| PIK3C3 | NFYC-AS1 | 0.458547 | 3.57E-29 |
| ATG16L2 | AC234582.1 | 0.458666 | 3.44E-29 |
| BIRC6 | AC024560.3 | 0.458681 | 3.42E-29 |
| NAF1 | Z98884.2 | 0.458709 | 3.39E-29 |
| RPS6KB1 | SAP30L-AS1 | 0.458764 | 3.34E-29 |
| NAF1 | AC073046.1 | 0.458798 | 3.30E-29 |
| RB1CC1 | AL513327.1 | 0.458802 | 3.30E-29 |
| BIRC6 | SH3BP5-AS1 | 0.458885 | 3.21E-29 |
| GOPC | AC073046.1 | 0.458924 | 3.17E-29 |
| MAPK8 | SNHG14 | 0.458935 | 3.16E-29 |
| RB1 | AC004918.3 | 0.458949 | 3.15E-29 |
| MAPK8 | AC138956.2 | 0.458954 | 3.15E-29 |
| NAF1 | AC005540.1 | 0.458956 | 3.14E-29 |
| RB1CC1 | AC021851.1 | 0.458979 | 3.12E-29 |
| NAF1 | AC006378.1 | 0.459012 | 3.09E-29 |
| ATG12 | AL359921.1 | 0.459012 | 3.09E-29 |
| ATG2B | HCG27 | 0.459023 | 3.08E-29 |
| CFLAR | AP006623.1 | 0.459066 | 3.04E-29 |
| RPS6KB1 | AL137782.1 | 0.45908 | 3.02E-29 |
| MTOR | AC068768.1 | 0.459139 | 2.97E-29 |
| RPS6KB1 | AC124283.2 | 0.459151 | 2.96E-29 |
| FOXO1 | PAXBP1-AS1 | 0.45919 | 2.92E-29 |
| KLHL24 | PWAR6 | 0.4592 | 2.91E-29 |
| EIF2AK2 | AP001458.1 | 0.459217 | 2.90E-29 |
| SIRT1 | EBLN3P | 0.45923 | 2.89E-29 |
| KLHL24 | AC092794.1 | 0.459248 | 2.87E-29 |
| FOXO3 | AP001033.2 | 0.459261 | 2.86E-29 |
| FOXO3 | N4BP2L2-IT2 | 0.459261 | 2.86E-29 |
| TSC1 | AC006547.1 | 0.459305 | 2.82E-29 |
| NAF1 | AC008669.1 | 0.459376 | 2.76E-29 |
| CFLAR | AL731567.1 | 0.459397 | 2.74E-29 |
| ATG7 | AC015911.3 | 0.459443 | 2.70E-29 |
| ATG12 | AL157932.1 | 0.459449 | 2.69E-29 |
| BIRC6 | AP000442.2 | 0.459454 | 2.69E-29 |
| RPS6KB1 | AL109614.1 | 0.459481 | 2.67E-29 |
| FOXO1 | AC120349.1 | 0.459493 | 2.66E-29 |
| FOXO3 | AC018752.1 | 0.459501 | 2.65E-29 |
| NAF1 | AC048341.1 | 0.459508 | 2.65E-29 |
| CAPN10 | SNHG11 | 0.459508 | 2.64E-29 |
| ATG16L2 | ADIRF-AS1 | 0.459523 | 2.63E-29 |
| MAPK8 | ABALON | 0.459613 | 2.56E-29 |
| TSC1 | AL928654.2 | 0.459643 | 2.54E-29 |
| RPS6KB1 | MIR29B2CHG | 0.459686 | 2.50E-29 |
| TSC1 | AC021851.1 | 0.459698 | 2.49E-29 |
| KLHL24 | AC008669.1 | 0.459708 | 2.48E-29 |
| CFLAR | AC133644.2 | 0.459712 | 2.48E-29 |
| ATG7 | AC099343.2 | 0.459718 | 2.48E-29 |
| CAPN10 | AP001453.3 | 0.459719 | 2.48E-29 |
| PIK3C3 | AC097641.2 | 0.45972 | 2.48E-29 |
| RB1 | AC012181.1 | 0.45973 | 2.47E-29 |
| RB1CC1 | USP46-AS1 | 0.459734 | 2.46E-29 |
| BIRC6 | AC005253.1 | 0.459767 | 2.44E-29 |
| ATG16L2 | AC027601.3 | 0.459768 | 2.44E-29 |
| GOPC | AL021578.1 | 0.459811 | 2.41E-29 |
| KLHL24 | AL117336.2 | 0.459823 | 2.40E-29 |
| ULK3 | AL021707.6 | 0.459837 | 2.39E-29 |
| DNAJB9 | LINC01094 | 0.459847 | 2.38E-29 |
| GOPC | AL359076.1 | 0.459871 | 2.36E-29 |
| RB1CC1 | AL078581.1 | 0.459901 | 2.34E-29 |
| CFLAR | LINC00513 | 0.45992 | 2.33E-29 |
| TP63 | AC022075.1 | 0.459943 | 2.31E-29 |
| ATG16L2 | SLC9A3-AS1 | 0.459961 | 2.30E-29 |
| KLHL24 | TMEM161B-AS1 | 0.459981 | 2.28E-29 |
| RPS6KB1 | AL031670.1 | 0.45999 | 2.28E-29 |
| CFLAR | AC009032.1 | 0.459992 | 2.27E-29 |
| CFLAR | AL596325.2 | 0.460004 | 2.26E-29 |
| ATG7 | AL133243.2 | 0.460109 | 2.19E-29 |
| NAF1 | AGAP1-IT1 | 0.460125 | 2.18E-29 |
| SIRT1 | AC022400.5 | 0.460144 | 2.17E-29 |
| TSC1 | PWAR6 | 0.460161 | 2.16E-29 |
| TSC1 | AC092953.2 | 0.460161 | 2.16E-29 |
| EIF2AK2 | AL137003.2 | 0.460162 | 2.16E-29 |
| RB1CC1 | AP001160.4 | 0.460169 | 2.15E-29 |
| TSC1 | AC084824.5 | 0.460189 | 2.14E-29 |
| PIK3C3 | AC074033.1 | 0.460191 | 2.14E-29 |
| PIK3C3 | AC067817.2 | 0.460199 | 2.13E-29 |
| RPS6KB1 | AC016590.2 | 0.460221 | 2.12E-29 |
| CXCR4 | LINC01215 | 0.460228 | 2.11E-29 |
| RPS6KB1 | AC005540.1 | 0.46024 | 2.10E-29 |
| KLHL24 | AC005479.2 | 0.460306 | 2.06E-29 |
| TSC1 | AC010542.5 | 0.460313 | 2.06E-29 |
| ATG12 | AL356356.1 | 0.460333 | 2.04E-29 |
| GOPC | AL022067.1 | 0.460341 | 2.04E-29 |
| NAF1 | AC012181.1 | 0.460342 | 2.04E-29 |
| RB1 | AP002336.2 | 0.460344 | 2.04E-29 |
| IFNG | AC083949.1 | 0.460379 | 2.01E-29 |
| TSC1 | AP006623.1 | 0.460394 | 2.00E-29 |
| CAPN10 | TMEM147-AS1 | 0.460425 | 1.99E-29 |
| ATG7 | CFLAR-AS1 | 0.460435 | 1.98E-29 |
| CFLAR | AC008124.1 | 0.460437 | 1.98E-29 |
| EIF4EBP1 | AC129507.4 | 0.46045 | 1.97E-29 |
| ATG12 | GABPB1-AS1 | 0.460455 | 1.97E-29 |
| ATG16L2 | AC004923.4 | 0.460589 | 1.89E-29 |
| RPS6KB1 | AC073046.1 | 0.4606 | 1.88E-29 |
| RB1CC1 | AP001486.2 | 0.460671 | 1.84E-29 |
| GABARAPL2 | ZSCAN16-AS1 | 0.460678 | 1.83E-29 |
| PIK3C3 | AC053527.1 | 0.46076 | 1.79E-29 |
| RB1CC1 | AC005104.1 | 0.460766 | 1.78E-29 |
| TSC1 | AC005021.1 | 0.460814 | 1.76E-29 |
| PIK3C3 | NARF-IT1 | 0.460835 | 1.75E-29 |
| EIF2AK2 | AC105389.2 | 0.460866 | 1.73E-29 |
| NAF1 | AP001628.1 | 0.460935 | 1.69E-29 |
| BIRC6 | AL162274.2 | 0.460953 | 1.68E-29 |
| TSC1 | THAP9-AS1 | 0.460963 | 1.68E-29 |
| KLHL24 | AL159169.2 | 0.460988 | 1.66E-29 |
| EIF2AK2 | AC124312.2 | 0.461004 | 1.66E-29 |
| MBTPS2 | RAP2C-AS1 | 0.461028 | 1.64E-29 |
| RPS6KB1 | RPS6KA2-IT1 | 0.461031 | 1.64E-29 |
| PIK3C3 | AP001458.1 | 0.461033 | 1.64E-29 |
| GOPC | AC066613.1 | 0.461046 | 1.63E-29 |
| MAPK8 | LINC00894 | 0.461093 | 1.61E-29 |
| ATG2B | AC095057.3 | 0.461108 | 1.60E-29 |
| RB1 | LINC00630 | 0.461116 | 1.60E-29 |
| MAPK8 | GAS8-AS1 | 0.461121 | 1.60E-29 |
| ATG16L2 | ZKSCAN2-DT | 0.46117 | 1.57E-29 |
| PTEN | AC004656.1 | 0.461171 | 1.57E-29 |
| PIK3C3 | AC073569.2 | 0.461229 | 1.54E-29 |
| RB1CC1 | AP003170.3 | 0.461238 | 1.54E-29 |
| RB1 | AL137779.2 | 0.461241 | 1.54E-29 |
| MAPK8 | AL354696.1 | 0.461271 | 1.52E-29 |
| MAPK8 | AC116366.1 | 0.461273 | 1.52E-29 |
| ATG16L2 | AC108673.3 | 0.461276 | 1.52E-29 |
| FOXO3 | AC004832.5 | 0.461291 | 1.51E-29 |
| RB1 | AL133330.1 | 0.461318 | 1.50E-29 |
| TSC1 | AL138756.1 | 0.461332 | 1.49E-29 |
| RB1 | AL513365.2 | 0.461349 | 1.48E-29 |
| ATG2B | AC139795.2 | 0.461377 | 1.47E-29 |
| ATG7 | AL731567.1 | 0.461391 | 1.47E-29 |
| FOXO1 | AL157786.1 | 0.461393 | 1.46E-29 |
| CFLAR | AC005261.1 | 0.461414 | 1.46E-29 |
| FOXO1 | AL606834.1 | 0.461444 | 1.44E-29 |
| KLHL24 | AL137782.1 | 0.461462 | 1.43E-29 |
| RB1CC1 | AC037198.2 | 0.461535 | 1.40E-29 |
| FOXO1 | AC127024.4 | 0.461539 | 1.40E-29 |
| RAB24 | AL021707.6 | 0.46157 | 1.39E-29 |
| MAPK8 | AL139011.1 | 0.461591 | 1.38E-29 |
| TSC1 | AC008115.3 | 0.461612 | 1.37E-29 |
| RB1CC1 | AC027277.2 | 0.461642 | 1.35E-29 |
| IFNG | AC243960.1 | 0.461657 | 1.35E-29 |
| KLHL24 | AC026470.2 | 0.461661 | 1.35E-29 |
| ATG12 | AC080013.4 | 0.461695 | 1.33E-29 |
| NAF1 | AC009948.1 | 0.461709 | 1.33E-29 |
| RPS6KB1 | AC037198.2 | 0.461724 | 1.32E-29 |
| RB1 | AC012170.2 | 0.461747 | 1.31E-29 |
| ATG12 | AC009041.4 | 0.461751 | 1.31E-29 |
| KLHL24 | AC027097.1 | 0.461808 | 1.29E-29 |
| PIK3C3 | AC090198.1 | 0.461826 | 1.28E-29 |
| EIF2AK2 | LINC01290 | 0.461831 | 1.28E-29 |
| RPS6KB1 | AC093388.1 | 0.461836 | 1.27E-29 |
| GOPC | AC020913.3 | 0.461867 | 1.26E-29 |
| MAPK8 | AC025287.3 | 0.461875 | 1.26E-29 |
| PIK3C3 | AL122035.1 | 0.461894 | 1.25E-29 |
| ULK3 | TNRC6C-AS1 | 0.461903 | 1.25E-29 |
| KLHL24 | AC079684.1 | 0.461907 | 1.25E-29 |
| TSC1 | AC037459.3 | 0.461946 | 1.23E-29 |
| NAF1 | AC093495.1 | 0.461972 | 1.22E-29 |
| KLHL24 | AC124312.2 | 0.462007 | 1.21E-29 |
| ATG16L2 | INE1 | 0.462052 | 1.19E-29 |
| FOXO3 | AC053513.1 | 0.462054 | 1.19E-29 |
| TSC1 | AC124312.2 | 0.462082 | 1.18E-29 |
| PIK3C3 | AC026355.1 | 0.462145 | 1.16E-29 |
| PIK3C3 | LINC01534 | 0.462172 | 1.15E-29 |
| ATG4B | LINC00115 | 0.462185 | 1.14E-29 |
| FOXO1 | AP001528.2 | 0.462263 | 1.11E-29 |
| FOXO1 | RBMS3-AS3 | 0.462297 | 1.10E-29 |
| KIF5B | SOS1-IT1 | 0.462347 | 1.08E-29 |
| GOPC | AP001469.2 | 0.462367 | 1.08E-29 |
| ATG7 | AC087284.1 | 0.462384 | 1.07E-29 |
| TSC2 | AC110285.2 | 0.462391 | 1.07E-29 |
| CFLAR | AC026124.2 | 0.4624 | 1.07E-29 |
| NAF1 | AL031670.1 | 0.462409 | 1.06E-29 |
| FOXO1 | LINC01376 | 0.462428 | 1.06E-29 |
| CFLAR | AC022973.3 | 0.462478 | 1.04E-29 |
| CFLAR | AL035409.1 | 0.462488 | 1.04E-29 |
| TSC1 | AC139887.1 | 0.462506 | 1.03E-29 |
| CFLAR | AC024060.1 | 0.462523 | 1.03E-29 |
| TSC1 | AC010536.2 | 0.462524 | 1.03E-29 |
| NLRC4 | AC108134.3 | 0.462524 | 1.03E-29 |
| RB1 | MALAT1 | 0.462538 | 1.02E-29 |
| MAPK8 | AL512413.1 | 0.462579 | 1.01E-29 |
| ATG12 | ABALON | 0.46259 | 1.00E-29 |
| FOXO1 | AP001486.2 | 0.462591 | 1.00E-29 |
| RPS6KB1 | BTBD9-AS1 | 0.462598 | 1.00E-29 |
| WDFY3 | AC024560.3 | 0.462618 | 9.95E-30 |
| KIF5B | HCG18 | 0.46268 | 9.76E-30 |
| CFLAR | LINC01534 | 0.462682 | 9.76E-30 |
| ATG7 | AL136115.2 | 0.462709 | 9.67E-30 |
| PIK3C3 | C1RL-AS1 | 0.462728 | 9.61E-30 |
| ATG7 | AL049840.5 | 0.462753 | 9.54E-30 |
| EEF2K | ERVK13-1 | 0.462795 | 9.41E-30 |
| RB1 | AL731566.1 | 0.462804 | 9.39E-30 |
| RB1CC1 | AC064807.1 | 0.462819 | 9.34E-30 |
| RB1 | AC010186.3 | 0.46283 | 9.31E-30 |
| MAPK8 | AC025178.1 | 0.462853 | 9.24E-30 |
| CTSD | SMIM25 | 0.462977 | 8.89E-30 |
| ATG7 | AC116366.1 | 0.462992 | 8.85E-30 |
| DAPK2 | AC137932.1 | 0.462999 | 8.82E-30 |
| TSC1 | AC090579.1 | 0.46301 | 8.79E-30 |
| RPS6KB1 | AC007938.3 | 0.463019 | 8.77E-30 |
| FOXO1 | AP000692.1 | 0.463031 | 8.74E-30 |
| TSC1 | HCG18 | 0.463043 | 8.70E-30 |
| WDFY3 | RUSC1-AS1 | 0.463049 | 8.69E-30 |
| CFLAR | AC005034.5 | 0.463061 | 8.65E-30 |
| ATG4B | AC104564.3 | 0.463076 | 8.61E-30 |
| ATG2B | AL513534.1 | 0.463119 | 8.50E-30 |
| FOXO3 | ADNP-AS1 | 0.463235 | 8.19E-30 |
| EIF2AK2 | AC002553.1 | 0.463278 | 8.08E-30 |
| PTEN | AL606489.1 | 0.463289 | 8.05E-30 |
| GOPC | AC020915.2 | 0.463309 | 8.00E-30 |
| RB1 | AL359076.1 | 0.463337 | 7.93E-30 |
| RB1 | AC008115.3 | 0.463342 | 7.92E-30 |
| FOXO1 | LINC00852 | 0.463345 | 7.91E-30 |
| MAPK8 | AL662844.3 | 0.463352 | 7.89E-30 |
| SPHK1 | MIR4435-2HG | 0.463364 | 7.86E-30 |
| NLRC4 | LINC00996 | 0.463392 | 7.79E-30 |
| ATG16L2 | AL354733.3 | 0.463398 | 7.78E-30 |
| ATG2B | AC125257.1 | 0.463402 | 7.77E-30 |
| BIRC6 | AC093726.2 | 0.463433 | 7.69E-30 |
| MAPK8 | AC005519.1 | 0.463473 | 7.60E-30 |
| TSC1 | AC078846.1 | 0.463475 | 7.59E-30 |
| FOXO1 | AC090579.1 | 0.46349 | 7.56E-30 |
| FOXO3 | AC007014.2 | 0.463499 | 7.53E-30 |
| RB1 | NUTM2A-AS1 | 0.463552 | 7.41E-30 |
| ATG7 | AP001381.1 | 0.463553 | 7.41E-30 |
| MAPK8 | AC073896.3 | 0.46356 | 7.39E-30 |
| ATG7 | AC133644.2 | 0.463604 | 7.29E-30 |
| TP63 | UNC5B-AS1 | 0.463626 | 7.24E-30 |
| NAF1 | AC053527.1 | 0.463663 | 7.15E-30 |
| FOXO1 | AC253576.2 | 0.463665 | 7.15E-30 |
| MAPK8 | AP000240.1 | 0.463677 | 7.12E-30 |
| ATG12 | AL078581.1 | 0.463683 | 7.11E-30 |
| RPS6KB1 | AC002128.2 | 0.463698 | 7.07E-30 |
| CFLAR | AC090425.2 | 0.463709 | 7.05E-30 |
| KLHL24 | AP003170.3 | 0.463725 | 7.01E-30 |
| PIK3C3 | HIF1A-AS2 | 0.463737 | 6.99E-30 |
| EEF2K | AC130456.3 | 0.463756 | 6.94E-30 |
| KLHL24 | AL596325.2 | 0.463757 | 6.94E-30 |
| GOPC | AC024933.1 | 0.463777 | 6.90E-30 |
| ATG7 | AL117381.1 | 0.46378 | 6.89E-30 |
| RB1 | AP000766.1 | 0.463819 | 6.81E-30 |
| GOPC | AC002128.2 | 0.463903 | 6.63E-30 |
| ATG12 | AL357992.1 | 0.463909 | 6.62E-30 |
| RB1CC1 | AL359915.2 | 0.463914 | 6.60E-30 |
| RB1CC1 | AC010168.2 | 0.46394 | 6.55E-30 |
| CAPN10 | AC009065.4 | 0.463966 | 6.50E-30 |
| TSC1 | STAG3L5P-PVRIG2P-PILRB | 0.464005 | 6.42E-30 |
| PTEN | DGCR11 | 0.464013 | 6.40E-30 |
| GOPC | AC005519.1 | 0.464015 | 6.40E-30 |
| RB1 | AL513550.1 | 0.464023 | 6.38E-30 |
| CAPN10 | AC007292.1 | 0.46405 | 6.33E-30 |
| EIF2AK2 | AL031716.1 | 0.464052 | 6.32E-30 |
| TSC1 | AL022328.3 | 0.464085 | 6.26E-30 |
| KIF5B | ALMS1-IT1 | 0.464102 | 6.22E-30 |
| PIK3C3 | AP000786.1 | 0.464122 | 6.18E-30 |
| MAPK8 | LINC00852 | 0.464136 | 6.16E-30 |
| RB1CC1 | AC084871.1 | 0.464145 | 6.14E-30 |
| SPNS1 | AL513320.1 | 0.46415 | 6.13E-30 |
| FOXO1 | AC078883.1 | 0.464183 | 6.07E-30 |
| CFLAR | AL138921.2 | 0.464189 | 6.05E-30 |
| TSC1 | AL117336.3 | 0.464199 | 6.03E-30 |
| RB1 | GAS5-AS1 | 0.464207 | 6.02E-30 |
| GNAI3 | AC099850.3 | 0.464211 | 6.01E-30 |
| TSC1 | AP000254.1 | 0.464224 | 5.99E-30 |
| RPS6KB1 | AL137779.2 | 0.464292 | 5.86E-30 |
| TSC1 | AC009090.3 | 0.464307 | 5.83E-30 |
| KIF5B | OIP5-AS1 | 0.464323 | 5.80E-30 |
| RB1CC1 | AP000692.1 | 0.464326 | 5.80E-30 |
| RB1CC1 | AC010201.2 | 0.464329 | 5.79E-30 |
| RB1CC1 | FLNB-AS1 | 0.464362 | 5.73E-30 |
| ATG2B | AC093227.1 | 0.464378 | 5.70E-30 |
| GOPC | AC084824.4 | 0.464425 | 5.62E-30 |
| ATG7 | AC022150.4 | 0.464425 | 5.62E-30 |
| ATG16L2 | AL021707.8 | 0.464482 | 5.52E-30 |
| ATG12 | KCCAT333 | 0.464489 | 5.50E-30 |
| RPS6KB1 | LUCAT1 | 0.46449 | 5.50E-30 |
| EIF2AK2 | ABALON | 0.464496 | 5.49E-30 |
| EIF2AK2 | AC009120.3 | 0.464518 | 5.45E-30 |
| KLHL24 | AP001469.2 | 0.464543 | 5.41E-30 |
| ATG12 | AL138921.2 | 0.464549 | 5.40E-30 |
| GOPC | AC022973.3 | 0.464559 | 5.38E-30 |
| UVRAG | AP000766.1 | 0.46461 | 5.30E-30 |
| KLHL24 | ATP1B3-AS1 | 0.464617 | 5.28E-30 |
| RB1CC1 | AC083949.1 | 0.464629 | 5.26E-30 |
| PIK3C3 | AC013403.2 | 0.464648 | 5.23E-30 |
| TSC1 | AC009090.1 | 0.46466 | 5.21E-30 |
| GOPC | SDCBP2-AS1 | 0.464662 | 5.21E-30 |
| RPS6KB1 | SAMD12-AS1 | 0.464678 | 5.18E-30 |
| RPS6KB1 | AC026470.2 | 0.464742 | 5.08E-30 |
| KLHL24 | KDM4A-AS1 | 0.464744 | 5.07E-30 |
| RB1 | ARHGEF38-IT1 | 0.464749 | 5.07E-30 |
| NAF1 | GAS8-AS1 | 0.464773 | 5.03E-30 |
| PIK3C3 | AC016542.1 | 0.464783 | 5.01E-30 |
| ATG7 | ABALON | 0.464794 | 4.99E-30 |
| EIF2AK2 | AL355488.1 | 0.46485 | 4.91E-30 |
| GOPC | ACTA2-AS1 | 0.464879 | 4.86E-30 |
| TP73 | LINC02541 | 0.464912 | 4.81E-30 |
| FOXO3 | SAP30L-AS1 | 0.464913 | 4.81E-30 |
| EIF2AK2 | AC083949.1 | 0.464928 | 4.79E-30 |
| GOPC | AC068790.5 | 0.464959 | 4.74E-30 |
| RB1 | AC006270.1 | 0.464983 | 4.70E-30 |
| RB1 | ALG13-AS1 | 0.465012 | 4.66E-30 |
| CCR2 | LINC01943 | 0.465037 | 4.62E-30 |
| RPS6KB1 | AL021578.1 | 0.465089 | 4.55E-30 |
| RB1 | AP001432.1 | 0.465093 | 4.54E-30 |
| PIK3C3 | AC139887.4 | 0.465108 | 4.52E-30 |
| EIF2AK2 | AC002128.1 | 0.46512 | 4.50E-30 |
| ATG7 | AC138393.3 | 0.465131 | 4.49E-30 |
| BIRC6 | AP001107.4 | 0.465135 | 4.48E-30 |
| RB1CC1 | AL137003.2 | 0.465161 | 4.44E-30 |
| MAPK8 | AC124319.2 | 0.465175 | 4.42E-30 |
| ATG12 | AC016542.1 | 0.465178 | 4.42E-30 |
| RPS6KB1 | ANKRD10-IT1 | 0.465194 | 4.40E-30 |
| KLHL24 | AC005540.1 | 0.465197 | 4.39E-30 |
| PIK3C3 | OSMR-AS1 | 0.465206 | 4.38E-30 |
| RPS6KB1 | AL513327.1 | 0.465215 | 4.37E-30 |
| GOPC | AC004466.3 | 0.465218 | 4.36E-30 |
| RPS6KB1 | MACC1-AS1 | 0.465222 | 4.36E-30 |
| BIRC6 | AC005479.1 | 0.465225 | 4.35E-30 |
| RB1CC1 | AC011442.1 | 0.465228 | 4.35E-30 |
| USP10 | AC099850.3 | 0.465312 | 4.24E-30 |
| MAPK8 | AC091185.1 | 0.465333 | 4.21E-30 |
| PIK3R4 | USP46-AS1 | 0.465338 | 4.20E-30 |
| ATG2B | ACBD3-AS1 | 0.465355 | 4.18E-30 |
| RB1 | AC138932.5 | 0.465396 | 4.12E-30 |
| FOXO1 | AC108010.1 | 0.46548 | 4.01E-30 |
| WDFY3 | AC079210.1 | 0.465496 | 3.99E-30 |
| CFLAR | ATP13A4-AS1 | 0.465498 | 3.99E-30 |
| EIF2AK2 | MBNL1-AS1 | 0.4655 | 3.99E-30 |
| RB1 | AP002907.1 | 0.465519 | 3.96E-30 |
| RAB24 | AC114730.3 | 0.465528 | 3.95E-30 |
| RB1CC1 | AC055822.1 | 0.465595 | 3.87E-30 |
| CFLAR | AC008760.1 | 0.465606 | 3.86E-30 |
| TSC1 | AC020915.3 | 0.465613 | 3.85E-30 |
| MAPK8 | AC242426.2 | 0.465624 | 3.83E-30 |
| RAB33B | LINC00630 | 0.46563 | 3.83E-30 |
| TSC1 | AP001178.2 | 0.465661 | 3.79E-30 |
| ATG12 | AC090617.5 | 0.465675 | 3.77E-30 |
| UVRAG | A2M-AS1 | 0.465675 | 3.77E-30 |
| CAPN10 | AC006942.1 | 0.465689 | 3.76E-30 |
| ATG4B | TONSL-AS1 | 0.46569 | 3.75E-30 |
| ATG2B | AC008035.1 | 0.465696 | 3.75E-30 |
| FOXO3 | AC110792.3 | 0.465699 | 3.74E-30 |
| TSC1 | AP000442.2 | 0.465702 | 3.74E-30 |
| PIK3C3 | PPP3CB-AS1 | 0.465707 | 3.73E-30 |
| NAF1 | AC242426.2 | 0.465719 | 3.72E-30 |
| RB1 | AL157402.2 | 0.46573 | 3.71E-30 |
| RPS6KB1 | AC004884.2 | 0.465731 | 3.71E-30 |
| ATG7 | AC069023.1 | 0.465773 | 3.66E-30 |
| RAB5A | FGD5-AS1 | 0.465774 | 3.66E-30 |
| FOXO3 | AC008770.3 | 0.465817 | 3.60E-30 |
| GOPC | GAS5-AS1 | 0.465837 | 3.58E-30 |
| RB1CC1 | AC007938.3 | 0.465857 | 3.56E-30 |
| GOPC | AC245014.3 | 0.465909 | 3.50E-30 |
| PIK3C3 | AC015871.3 | 0.465945 | 3.46E-30 |
| TSC1 | AC113139.1 | 0.465946 | 3.46E-30 |
| BIRC6 | AC025857.2 | 0.465994 | 3.41E-30 |
| RPS6KB1 | AP001160.4 | 0.466022 | 3.38E-30 |
| EEF2K | AC024075.1 | 0.466077 | 3.32E-30 |
| PIK3C3 | AL157932.1 | 0.466094 | 3.30E-30 |
| ATG12 | AC112722.1 | 0.4661 | 3.29E-30 |
| TSC1 | AL031600.1 | 0.466122 | 3.27E-30 |
| NAF1 | AP000786.1 | 0.46614 | 3.25E-30 |
| TSC2 | LINC01786 | 0.466183 | 3.21E-30 |
| CAPN10 | AP003419.3 | 0.466234 | 3.16E-30 |
| NAF1 | AC074032.1 | 0.466239 | 3.15E-30 |
| TSC1 | AC009118.3 | 0.466258 | 3.13E-30 |
| ATG4B | AL360181.2 | 0.46626 | 3.13E-30 |
| MAPK8 | AC024075.1 | 0.466327 | 3.06E-30 |
| FOXO3 | SOS1-IT1 | 0.466339 | 3.05E-30 |
| WDFY3 | AC004908.1 | 0.466353 | 3.04E-30 |
| NAF1 | AL139011.1 | 0.466382 | 3.01E-30 |
| GOPC | AP002336.2 | 0.466388 | 3.00E-30 |
| RB1CC1 | PPP3CB-AS1 | 0.466412 | 2.98E-30 |
| CFLAR | AC024933.1 | 0.466416 | 2.98E-30 |
| EIF2AK2 | CCDC18-AS1 | 0.46644 | 2.95E-30 |
| RPS6KB1 | AC026202.2 | 0.466445 | 2.95E-30 |
| BIRC6 | AC109347.2 | 0.466476 | 2.92E-30 |
| KLHL24 | AC139887.4 | 0.466481 | 2.92E-30 |
| KLHL24 | AC010536.2 | 0.466492 | 2.91E-30 |
| TSC1 | AC092123.1 | 0.466505 | 2.89E-30 |
| NAF1 | AC010201.2 | 0.466529 | 2.87E-30 |
| TSC1 | AC087276.1 | 0.466533 | 2.87E-30 |
| RB1 | TRAF3IP2-AS1 | 0.466566 | 2.84E-30 |
| RPS6KB1 | AC090739.1 | 0.466568 | 2.84E-30 |
| EIF2AK2 | AL360219.1 | 0.466632 | 2.78E-30 |
| KLHL24 | AC002044.1 | 0.466652 | 2.76E-30 |
| FOXO3 | AL353804.1 | 0.466673 | 2.74E-30 |
| FOXO3 | AC008115.3 | 0.466684 | 2.73E-30 |
| CCR2 | LINC02362 | 0.466699 | 2.72E-30 |
| ATG7 | AC008770.3 | 0.466708 | 2.71E-30 |
| ATG7 | AL139407.1 | 0.466728 | 2.69E-30 |
| RPS6KB1 | MIR222HG | 0.466735 | 2.69E-30 |
| RAB24 | AL591845.1 | 0.466751 | 2.67E-30 |
| GOPC | NEAT1 | 0.466753 | 2.67E-30 |
| TSC1 | AL136304.1 | 0.466753 | 2.67E-30 |
| TSC1 | AL121772.3 | 0.466762 | 2.66E-30 |
| RB1 | AC068790.5 | 0.466772 | 2.66E-30 |
| GOPC | AC026124.2 | 0.466796 | 2.64E-30 |
| CFLAR | TPT1-AS1 | 0.466814 | 2.62E-30 |
| RPS6KB1 | AL592148.3 | 0.466827 | 2.61E-30 |
| GOPC | SMC5-AS1 | 0.466891 | 2.56E-30 |
| RB1CC1 | AC093484.4 | 0.466932 | 2.52E-30 |
| CFLAR | FLNB-AS1 | 0.466934 | 2.52E-30 |
| FOXO1 | AC002128.2 | 0.466954 | 2.51E-30 |
| KLHL24 | MCCC1-AS1 | 0.466955 | 2.51E-30 |
| FOXO3 | AL136115.2 | 0.466966 | 2.50E-30 |
| CFLAR | TRAF3IP2-AS1 | 0.466993 | 2.47E-30 |
| FOXO3 | AC108727.1 | 0.467029 | 2.45E-30 |
| RB1CC1 | AL132780.1 | 0.467033 | 2.44E-30 |
| PTEN | AC093726.1 | 0.467046 | 2.43E-30 |
| FOXO3 | AC080162.1 | 0.467076 | 2.41E-30 |
| RB1CC1 | AC024075.1 | 0.467088 | 2.40E-30 |
| GOPC | AP000873.2 | 0.467121 | 2.38E-30 |
| FOXO1 | AC018638.7 | 0.467236 | 2.29E-30 |
| RB1 | RRN3P2 | 0.467245 | 2.28E-30 |
| BIRC6 | AL139289.1 | 0.467292 | 2.25E-30 |
| RPS6KB1 | Z98884.2 | 0.467295 | 2.25E-30 |
| RPS6KB1 | OCIAD1-AS1 | 0.467351 | 2.21E-30 |
| EIF2AK2 | AC079921.2 | 0.467377 | 2.19E-30 |
| TSC1 | AC138028.4 | 0.467395 | 2.18E-30 |
| TSC1 | AC010168.2 | 0.467402 | 2.17E-30 |
| RPS6KB1 | AL021707.7 | 0.467438 | 2.15E-30 |
| CFLAR | AL359921.1 | 0.467449 | 2.14E-30 |
| RB1CC1 | AC073569.2 | 0.467478 | 2.12E-30 |
| RB1 | AC005540.1 | 0.467485 | 2.11E-30 |
| RB1CC1 | AL606834.1 | 0.467511 | 2.10E-30 |
| FOXO1 | AC092953.2 | 0.46753 | 2.08E-30 |
| SPNS1 | AC069281.2 | 0.467538 | 2.08E-30 |
| MBTPS2 | LINC00630 | 0.467568 | 2.06E-30 |
| ATG12 | C1RL-AS1 | 0.467594 | 2.04E-30 |
| RB1CC1 | AC125257.1 | 0.467643 | 2.01E-30 |
| CAPN10 | AL139287.1 | 0.467647 | 2.01E-30 |
| RPS6KB1 | MUC20-OT1 | 0.4677 | 1.97E-30 |
| GOPC | A2M-AS1 | 0.467719 | 1.96E-30 |
| ATG16L2 | AC120053.1 | 0.467724 | 1.96E-30 |
| CFLAR | AC120349.1 | 0.467736 | 1.95E-30 |
| ULK3 | AC114730.3 | 0.467755 | 1.94E-30 |
| WDFY3 | AC079921.2 | 0.467794 | 1.91E-30 |
| FOXO3 | AC002550.2 | 0.467795 | 1.91E-30 |
| KLHL24 | AC125257.1 | 0.467802 | 1.91E-30 |
| EIF2AK2 | AL512791.1 | 0.467808 | 1.91E-30 |
| FOXO1 | AL354733.3 | 0.467822 | 1.90E-30 |
| RB1CC1 | AL132989.1 | 0.46784 | 1.89E-30 |
| RB1 | AL353804.1 | 0.467846 | 1.88E-30 |
| ATG7 | AL162724.2 | 0.467881 | 1.86E-30 |
| CAPN10 | AC233728.1 | 0.467913 | 1.84E-30 |
| TSC1 | AC234775.3 | 0.467949 | 1.82E-30 |
| TSC1 | AC090181.2 | 0.467966 | 1.81E-30 |
| NAF1 | PCBP1-AS1 | 0.467985 | 1.80E-30 |
| RB1 | AC234775.3 | 0.467997 | 1.79E-30 |
| RPS6KB1 | KIF26B-AS1 | 0.468008 | 1.79E-30 |
| CASP1 | LINC01150 | 0.468029 | 1.78E-30 |
| RB1CC1 | A2M-AS1 | 0.468035 | 1.77E-30 |
| TSC1 | AL592148.3 | 0.468072 | 1.75E-30 |
| CFLAR | SP2-AS1 | 0.468103 | 1.73E-30 |
| GOPC | AC073651.1 | 0.46811 | 1.73E-30 |
| GOPC | AC048344.4 | 0.468127 | 1.72E-30 |
| NAF1 | AL031775.2 | 0.468153 | 1.71E-30 |
| KLHL24 | PPP3CB-AS1 | 0.468164 | 1.70E-30 |
| MAPK8 | AC087392.1 | 0.468181 | 1.69E-30 |
| GOPC | AC124312.5 | 0.468191 | 1.68E-30 |
| NAF1 | AC120349.1 | 0.468266 | 1.64E-30 |
| FOXO1 | AL512791.1 | 0.468267 | 1.64E-30 |
| FOXO1 | AP001458.1 | 0.468292 | 1.63E-30 |
| RB1CC1 | AL355075.2 | 0.468325 | 1.61E-30 |
| TSC1 | AC242426.2 | 0.468348 | 1.60E-30 |
| ATG16L2 | AC004908.1 | 0.468352 | 1.60E-30 |
| FOXO1 | TPT1-AS1 | 0.468358 | 1.60E-30 |
| EIF2AK2 | AL359715.3 | 0.468375 | 1.59E-30 |
| EIF2AK2 | ARHGAP31-AS1 | 0.468399 | 1.58E-30 |
| MAPK8 | ZNF32-AS2 | 0.46845 | 1.55E-30 |
| ATG2B | AC020915.3 | 0.468469 | 1.54E-30 |
| TSC1 | AC124319.2 | 0.468486 | 1.53E-30 |
| PIK3C3 | ALMS1-IT1 | 0.468512 | 1.52E-30 |
| ATG16L2 | AL354836.1 | 0.468519 | 1.52E-30 |
| RB1 | AC090579.1 | 0.468551 | 1.50E-30 |
| NLRC4 | MIR155HG | 0.468557 | 1.50E-30 |
| TSC1 | AL139407.1 | 0.468564 | 1.49E-30 |
| ATG2B | AL078581.1 | 0.468573 | 1.49E-30 |
| RPS6KB1 | SMC5-AS1 | 0.468595 | 1.48E-30 |
| RB1CC1 | ERVK13-1 | 0.468598 | 1.48E-30 |
| RB1 | AF178030.1 | 0.468642 | 1.46E-30 |
| FOXO1 | AC010168.2 | 0.468663 | 1.45E-30 |
| GOPC | NORAD | 0.468694 | 1.43E-30 |
| PTEN | AC124016.2 | 0.468718 | 1.42E-30 |
| FOXO1 | AC009054.2 | 0.468725 | 1.42E-30 |
| GOPC | LINC00641 | 0.46873 | 1.42E-30 |
| RB1CC1 | AC015911.3 | 0.468731 | 1.42E-30 |
| ATG4B | ZNF213-AS1 | 0.468782 | 1.39E-30 |
| EIF2AK2 | AP001625.2 | 0.468784 | 1.39E-30 |
| ATG16L2 | AP000442.2 | 0.468822 | 1.37E-30 |
| CAPN10 | ARRDC1-AS1 | 0.468854 | 1.36E-30 |
| ATG2B | AC073896.3 | 0.468864 | 1.36E-30 |
| RAB24 | ASB16-AS1 | 0.468872 | 1.35E-30 |
| RB1 | AC009032.1 | 0.468873 | 1.35E-30 |
| CFLAR | MBNL1-AS1 | 0.468921 | 1.33E-30 |
| FOXO1 | AC245014.3 | 0.468934 | 1.33E-30 |
| ATG4B | LINC00685 | 0.46899 | 1.30E-30 |
| ATG12 | AC124016.2 | 0.468991 | 1.30E-30 |
| ATG7 | AL139120.1 | 0.469024 | 1.29E-30 |
| CFLAR | AC026202.2 | 0.469028 | 1.29E-30 |
| FOXO3 | AL136320.1 | 0.469055 | 1.28E-30 |
| RAB24 | ASMTL-AS1 | 0.469063 | 1.27E-30 |
| NAF1 | AC005104.1 | 0.46908 | 1.27E-30 |
| NAF1 | AC091185.1 | 0.469104 | 1.26E-30 |
| HIF1A | LINC00973 | 0.469108 | 1.25E-30 |
| ATG7 | AC022973.3 | 0.46911 | 1.25E-30 |
| BIRC6 | AL513534.1 | 0.469111 | 1.25E-30 |
| GOPC | KLF7-IT1 | 0.46912 | 1.25E-30 |
| TSC1 | AC012181.2 | 0.469176 | 1.23E-30 |
| PIK3C3 | AC005104.1 | 0.469189 | 1.22E-30 |
| MAPK8 | AC098484.1 | 0.469202 | 1.22E-30 |
| NAF1 | AC009318.3 | 0.469217 | 1.21E-30 |
| BIRC6 | AC079921.2 | 0.469238 | 1.20E-30 |
| CFLAR | DNM3OS | 0.469251 | 1.20E-30 |
| RPS6KB1 | NUTM2B-AS1 | 0.469253 | 1.20E-30 |
| ATG12 | ALMS1-IT1 | 0.469256 | 1.20E-30 |
| PIK3C3 | FLNB-AS1 | 0.469261 | 1.19E-30 |
| PIK3C3 | AC002553.1 | 0.469265 | 1.19E-30 |
| TSC1 | TMEM161B-AS1 | 0.469328 | 1.17E-30 |
| FOXO1 | AC005261.1 | 0.46933 | 1.17E-30 |
| ATG7 | NPTN-IT1 | 0.469345 | 1.16E-30 |
| CFLAR | AL139011.1 | 0.469441 | 1.13E-30 |
| ATG7 | AC004832.5 | 0.469478 | 1.11E-30 |
| CAPN10 | H1FX-AS1 | 0.46948 | 1.11E-30 |
| ATG2B | AC026368.1 | 0.46953 | 1.09E-30 |
| PIK3C3 | AC078778.1 | 0.469537 | 1.09E-30 |
| RPS6KB1 | AC012181.1 | 0.469562 | 1.08E-30 |
| IL24 | AC018755.4 | 0.469595 | 1.07E-30 |
| ATG2B | AL162274.2 | 0.4696 | 1.07E-30 |
| ATG2B | LINC01389 | 0.469624 | 1.06E-30 |
| RB1 | SMC5-AS1 | 0.469627 | 1.06E-30 |
| TSC1 | AC048341.2 | 0.469641 | 1.06E-30 |
| ATG12 | AC009090.1 | 0.469655 | 1.05E-30 |
| KLHL24 | AC087752.4 | 0.469665 | 1.05E-30 |
| RB1CC1 | AC124312.5 | 0.469667 | 1.05E-30 |
| GOPC | RRN3P2 | 0.469671 | 1.05E-30 |
| RB1CC1 | AC068792.1 | 0.469693 | 1.04E-30 |
| ATG12 | ARMCX5-GPRASP2 | 0.469708 | 1.03E-30 |
| FOXO1 | AP000240.1 | 0.469763 | 1.01E-30 |
| RB1 | AC002553.2 | 0.469775 | 1.01E-30 |
| ATG7 | AC006270.1 | 0.469776 | 1.01E-30 |
| FOXO1 | AC074032.1 | 0.469795 | 1.00E-30 |
| TSC1 | AC004918.3 | 0.469814 | 9.98E-31 |
| MAPK8 | AC011468.5 | 0.469823 | 9.95E-31 |
| RB1 | AC096741.1 | 0.46987 | 9.80E-31 |
| ATG12 | AL139011.1 | 0.469888 | 9.74E-31 |
| MAPK8 | AL137779.2 | 0.469897 | 9.72E-31 |
| MAPK8 | AL132780.1 | 0.469919 | 9.65E-31 |
| ATG16L2 | AL132989.1 | 0.469932 | 9.61E-31 |
| RB1CC1 | AC008124.1 | 0.469939 | 9.58E-31 |
| GOPC | FAM13A-AS1 | 0.46997 | 9.49E-31 |
| RB1CC1 | AC011815.1 | 0.470052 | 9.24E-31 |
| SQSTM1 | LINC00847 | 0.470101 | 9.10E-31 |
| IFNG | PSMB8-AS1 | 0.470104 | 9.09E-31 |
| ATG7 | ATP1B3-AS1 | 0.470134 | 9.00E-31 |
| CAPN10 | AC011462.4 | 0.470158 | 8.93E-31 |
| KLHL24 | DLEU1 | 0.470159 | 8.93E-31 |
| WDFY3 | AL139289.1 | 0.470167 | 8.90E-31 |
| FOXO1 | AC084871.1 | 0.470339 | 8.42E-31 |
| CFLAR | IGBP1-AS1 | 0.470348 | 8.40E-31 |
| FOXO3 | ERVK13-1 | 0.470354 | 8.38E-31 |
| ATG2B | AC008764.2 | 0.470392 | 8.28E-31 |
| CXCR4 | AL928742.1 | 0.470395 | 8.27E-31 |
| FOXO1 | AL731566.1 | 0.470407 | 8.24E-31 |
| WDFY3 | THUMPD3-AS1 | 0.470412 | 8.22E-31 |
| ATG16L2 | AC024060.1 | 0.470451 | 8.12E-31 |
| RB1CC1 | AC139887.2 | 0.470477 | 8.05E-31 |
| ATG7 | AC066613.1 | 0.470491 | 8.02E-31 |
| ATG12 | MBNL1-AS1 | 0.470498 | 8.00E-31 |
| KLHL24 | AC008966.2 | 0.470503 | 7.99E-31 |
| ATG2B | AL031673.1 | 0.470518 | 7.95E-31 |
| ATG7 | ACAP2-IT1 | 0.470526 | 7.93E-31 |
| RB1CC1 | OSMR-AS1 | 0.470548 | 7.87E-31 |
| ATG16L2 | AC132872.1 | 0.470594 | 7.75E-31 |
| PIK3C3 | AP003170.3 | 0.470621 | 7.69E-31 |
| CFLAR | AC011442.1 | 0.470635 | 7.65E-31 |
| RB1CC1 | RAB30-AS1 | 0.470675 | 7.55E-31 |
| EIF2AK2 | AL139011.1 | 0.470686 | 7.53E-31 |
| RB1 | Z82243.1 | 0.470705 | 7.48E-31 |
| RB1CC1 | AC013403.2 | 0.470711 | 7.46E-31 |
| NAF1 | MAST4-AS1 | 0.470744 | 7.38E-31 |
| BIRC6 | AC009237.15 | 0.470773 | 7.32E-31 |
| RB1 | AC010834.3 | 0.470829 | 7.18E-31 |
| NAF1 | AC025287.3 | 0.470846 | 7.14E-31 |
| PIK3C3 | AC002044.1 | 0.470869 | 7.09E-31 |
| FOXO3 | AC090739.1 | 0.470886 | 7.05E-31 |
| CFLAR | AC090589.3 | 0.470904 | 7.01E-31 |
| PIK3C3 | AL359921.1 | 0.470958 | 6.89E-31 |
| RB1CC1 | AC008035.1 | 0.470967 | 6.87E-31 |
| ATG2B | ARMCX5-GPRASP2 | 0.470998 | 6.80E-31 |
| FOXO1 | AC019080.5 | 0.471018 | 6.76E-31 |
| PTEN | KCCAT333 | 0.471033 | 6.72E-31 |
| FOXO1 | RPS6KA2-IT1 | 0.471041 | 6.71E-31 |
| EIF2AK2 | TMEM161B-AS1 | 0.471066 | 6.65E-31 |
| RB1 | AC020913.3 | 0.471078 | 6.63E-31 |
| MAPK8 | AC009948.1 | 0.471084 | 6.61E-31 |
| PIK3C3 | AL031666.1 | 0.47111 | 6.56E-31 |
| CAPN10 | AC012615.6 | 0.471123 | 6.53E-31 |
| KLHL24 | AL354696.1 | 0.471176 | 6.42E-31 |
| CAPN10 | LINC00115 | 0.471187 | 6.39E-31 |
| KLHL24 | AC096921.2 | 0.471214 | 6.34E-31 |
| RPS6KB1 | LINC01376 | 0.471221 | 6.33E-31 |
| TSC1 | AC083843.2 | 0.471235 | 6.30E-31 |
| BIRC6 | AL049840.3 | 0.471238 | 6.29E-31 |
| RB1 | AL022067.1 | 0.471259 | 6.25E-31 |
| EDEM1 | LINC01655 | 0.471262 | 6.24E-31 |
| FOXO3 | AC108010.1 | 0.471273 | 6.22E-31 |
| RPS6KB1 | AP005131.7 | 0.471275 | 6.21E-31 |
| PIK3C3 | AC115989.1 | 0.471292 | 6.18E-31 |
| RB1 | AC093799.1 | 0.471307 | 6.15E-31 |
| KIF5B | AC022150.4 | 0.471341 | 6.08E-31 |
| EIF2AK2 | AC096921.2 | 0.471353 | 6.06E-31 |
| RB1 | AL109614.1 | 0.471357 | 6.05E-31 |
| CAPN10 | AC011472.1 | 0.471424 | 5.92E-31 |
| TSC1 | LAMC1-AS1 | 0.471437 | 5.90E-31 |
| PTEN | LINC02035 | 0.471439 | 5.89E-31 |
| PIK3C3 | NUTM2A-AS1 | 0.471457 | 5.86E-31 |
| PIK3C3 | AP001469.2 | 0.471465 | 5.84E-31 |
| MAPK8 | RRN3P2 | 0.471472 | 5.83E-31 |
| GOPC | AC108727.1 | 0.471476 | 5.82E-31 |
| GOPC | BTBD9-AS1 | 0.471502 | 5.77E-31 |
| MAPK8 | AC011815.1 | 0.471503 | 5.77E-31 |
| EIF2AK2 | AP003170.3 | 0.471512 | 5.75E-31 |
| UVRAG | AC024075.1 | 0.471527 | 5.73E-31 |
| RB1 | AC048344.4 | 0.471533 | 5.71E-31 |
| RB1CC1 | AC098851.1 | 0.471553 | 5.68E-31 |
| RB1CC1 | AC115989.1 | 0.471582 | 5.62E-31 |
| BIRC6 | AL445493.3 | 0.471604 | 5.58E-31 |
| PIK3C3 | AC037198.1 | 0.471608 | 5.58E-31 |
| CFLAR | AL158166.1 | 0.471609 | 5.58E-31 |
| BIRC6 | LUCAT1 | 0.471619 | 5.56E-31 |
| RB1 | KIF26B-AS1 | 0.471631 | 5.54E-31 |
| ATG4B | CD27-AS1 | 0.471668 | 5.47E-31 |
| MAPK8 | AC073046.1 | 0.471669 | 5.47E-31 |
| PIK3C3 | LINC02352 | 0.471677 | 5.45E-31 |
| PIK3C3 | AL359697.1 | 0.471678 | 5.45E-31 |
| ATG2B | AC007319.1 | 0.47168 | 5.45E-31 |
| CAPNS1 | SCGB1B2P | 0.471782 | 5.27E-31 |
| ATG16L2 | AC037459.3 | 0.4718 | 5.24E-31 |
| TSC1 | AC112722.1 | 0.471828 | 5.19E-31 |
| CFLAR | AC012467.1 | 0.471839 | 5.17E-31 |
| ATG16L2 | MIR3936HG | 0.471851 | 5.15E-31 |
| EIF2AK2 | AL157394.1 | 0.471891 | 5.09E-31 |
| MAP1LC3C | ERVH48-1 | 0.471941 | 5.00E-31 |
| TSC1 | AL513365.2 | 0.471989 | 4.93E-31 |
| WDFY3 | AC093726.1 | 0.472041 | 4.84E-31 |
| RPS6KB1 | KDM4A-AS1 | 0.472077 | 4.79E-31 |
| ATG12 | AC015813.1 | 0.472078 | 4.79E-31 |
| TSC1 | SNHG20 | 0.472084 | 4.78E-31 |
| ATG7 | AC063965.1 | 0.472112 | 4.73E-31 |
| EIF4G1 | AC099850.3 | 0.472213 | 4.58E-31 |
| EIF2AK2 | AP001160.4 | 0.472238 | 4.54E-31 |
| FOXO1 | MIR29B2CHG | 0.472251 | 4.52E-31 |
| MAPK8 | ERVK13-1 | 0.472277 | 4.49E-31 |
| RB1CC1 | KIF26B-AS1 | 0.472317 | 4.43E-31 |
| NAF1 | AL138921.2 | 0.472338 | 4.40E-31 |
| RB1 | AC108727.1 | 0.472406 | 4.30E-31 |
| TSC1 | GAS5-AS1 | 0.472436 | 4.26E-31 |
| KLHL24 | AC092279.1 | 0.472457 | 4.23E-31 |
| CFLAR | PAXIP1-AS2 | 0.472493 | 4.18E-31 |
| GOPC | AP000786.1 | 0.472499 | 4.17E-31 |
| EIF2AK2 | AC138956.1 | 0.472544 | 4.11E-31 |
| ATG12 | UGDH-AS1 | 0.472561 | 4.09E-31 |
| EIF2AK2 | INE1 | 0.472564 | 4.08E-31 |
| CFLAR | AC087284.1 | 0.472569 | 4.08E-31 |
| GOPC | AC096921.2 | 0.472573 | 4.07E-31 |
| ATG7 | AC008906.1 | 0.472639 | 3.99E-31 |
| TSC1 | AC010245.2 | 0.472674 | 3.94E-31 |
| MAPK8 | MAST4-AS1 | 0.472681 | 3.93E-31 |
| BIRC6 | LINC02100 | 0.47271 | 3.90E-31 |
| TSC2 | AP006623.1 | 0.472724 | 3.88E-31 |
| PTEN | AL121839.2 | 0.47273 | 3.87E-31 |
| ATG4B | AC074117.1 | 0.472735 | 3.86E-31 |
| WDFY3 | AC083862.2 | 0.472742 | 3.85E-31 |
| RB1CC1 | AC005034.5 | 0.472745 | 3.85E-31 |
| FOXO1 | MACC1-AS1 | 0.472764 | 3.83E-31 |
| CFLAR | ATP1A1-AS1 | 0.472772 | 3.82E-31 |
| RPS6KB1 | AL049840.2 | 0.472804 | 3.78E-31 |
| GOPC | ERVK13-1 | 0.472816 | 3.76E-31 |
| MAP2K7 | AC007292.1 | 0.472825 | 3.75E-31 |
| NAF1 | AC092794.1 | 0.472834 | 3.74E-31 |
| BIRC6 | IGBP1-AS1 | 0.472852 | 3.72E-31 |
| FOXO3 | AC007878.1 | 0.472885 | 3.68E-31 |
| TSC2 | AL390719.2 | 0.472913 | 3.64E-31 |
| NAF1 | AC127024.5 | 0.472929 | 3.63E-31 |
| FOXO3 | AC005034.5 | 0.472937 | 3.62E-31 |
| GOPC | AC016590.2 | 0.472946 | 3.61E-31 |
| FOXO1 | AC133644.2 | 0.472968 | 3.58E-31 |
| ATG12 | AL157838.1 | 0.473015 | 3.53E-31 |
| ATG12 | AC005674.2 | 0.473035 | 3.50E-31 |
| RB1 | AL513008.1 | 0.473054 | 3.48E-31 |
| RPS6KB1 | AC141002.1 | 0.47306 | 3.47E-31 |
| PTEN | AC087481.3 | 0.473083 | 3.45E-31 |
| PIK3C3 | AL662844.3 | 0.473115 | 3.41E-31 |
| MTOR | Z68871.1 | 0.473161 | 3.36E-31 |
| RB1CC1 | AC009318.3 | 0.473174 | 3.35E-31 |
| RB1 | AC027097.2 | 0.473176 | 3.34E-31 |
| MAPK8 | AC067817.2 | 0.473192 | 3.33E-31 |
| FOXO3 | AC087286.2 | 0.473197 | 3.32E-31 |
| FOXO3 | LIMS1-AS1 | 0.473218 | 3.30E-31 |
| NAF1 | AP003170.3 | 0.473233 | 3.28E-31 |
| FOXO1 | AC022150.4 | 0.473266 | 3.25E-31 |
| EIF2AK2 | AC005034.3 | 0.473287 | 3.23E-31 |
| GOPC | AC087286.1 | 0.4733 | 3.21E-31 |
| ATG16L2 | AC022400.1 | 0.473301 | 3.21E-31 |
| RAB24 | STAG3L5P-PVRIG2P-PILRB | 0.473314 | 3.20E-31 |
| ATG7 | AC037198.1 | 0.473367 | 3.14E-31 |
| UVRAG | Z68871.1 | 0.47339 | 3.12E-31 |
| CAPN10 | AC010973.2 | 0.473398 | 3.11E-31 |
| BIRC6 | AL359962.1 | 0.473408 | 3.10E-31 |
| TSC1 | AL157392.4 | 0.473418 | 3.09E-31 |
| KLHL24 | AL355488.1 | 0.473522 | 2.99E-31 |
| KLHL24 | AC066613.1 | 0.473526 | 2.98E-31 |
| CFLAR | AP001625.2 | 0.473542 | 2.97E-31 |
| ITGB1 | AP000695.1 | 0.473551 | 2.96E-31 |
| RPS6KB1 | CCDC18-AS1 | 0.473559 | 2.95E-31 |
| RB1CC1 | AC092611.2 | 0.473598 | 2.91E-31 |
| NAF1 | AP005899.1 | 0.47362 | 2.89E-31 |
| FOXO3 | AL138963.1 | 0.473624 | 2.89E-31 |
| RB1CC1 | AL138921.2 | 0.473634 | 2.88E-31 |
| KLHL24 | AC053527.1 | 0.473665 | 2.85E-31 |
| EIF2AK2 | MIR222HG | 0.473673 | 2.84E-31 |
| KLHL24 | AL078581.1 | 0.473676 | 2.84E-31 |
| FOXO3 | AC087276.1 | 0.473722 | 2.80E-31 |
| SPNS1 | LINC01089 | 0.473724 | 2.80E-31 |
| ATG16L2 | AP000553.2 | 0.473731 | 2.79E-31 |
| FOXO3 | AL078581.1 | 0.473749 | 2.77E-31 |
| RPS6KB1 | AC078846.1 | 0.473757 | 2.76E-31 |
| GOPC | AC131971.1 | 0.473764 | 2.76E-31 |
| KLHL24 | RBMS3-AS3 | 0.47378 | 2.74E-31 |
| PIK3C3 | AC025171.3 | 0.473782 | 2.74E-31 |
| EIF2AK2 | AC138207.4 | 0.473818 | 2.71E-31 |
| EIF2AK2 | AC005540.1 | 0.473855 | 2.68E-31 |
| RB1CC1 | AC009318.2 | 0.473856 | 2.68E-31 |
| PIK3C3 | MAST4-AS1 | 0.473893 | 2.64E-31 |
| GOPC | AL133445.2 | 0.473942 | 2.60E-31 |
| TSC1 | AC095057.3 | 0.473955 | 2.59E-31 |
| RPS6KB1 | AL606834.1 | 0.473961 | 2.59E-31 |
| EIF2AK2 | AC010615.2 | 0.473975 | 2.57E-31 |
| RB1 | AC037487.2 | 0.473989 | 2.56E-31 |
| ATG2B | AC004908.1 | 0.473999 | 2.55E-31 |
| KLHL24 | AC004466.3 | 0.474009 | 2.55E-31 |
| CFLAR | C1RL-AS1 | 0.47401 | 2.55E-31 |
| GOPC | AC010186.3 | 0.474015 | 2.54E-31 |
| GOPC | AC058791.1 | 0.474031 | 2.53E-31 |
| EIF2AK2 | AC004466.3 | 0.474045 | 2.52E-31 |
| FOXO1 | AC015911.3 | 0.474051 | 2.51E-31 |
| EIF2AK2 | AC007552.2 | 0.474053 | 2.51E-31 |
| RPS6KB1 | LINC00894 | 0.474056 | 2.51E-31 |
| FOXO3 | AC010186.3 | 0.474064 | 2.50E-31 |
| CFLAR | AL132780.1 | 0.474076 | 2.49E-31 |
| KLHL24 | AP001458.1 | 0.474089 | 2.48E-31 |
| PTEN | AP006621.2 | 0.474111 | 2.46E-31 |
| PTEN | AC093484.4 | 0.474113 | 2.46E-31 |
| RPS6KB1 | AC133644.2 | 0.474117 | 2.46E-31 |
| ATG12 | AC004253.1 | 0.474127 | 2.45E-31 |
| NAF1 | AC007552.2 | 0.474178 | 2.41E-31 |
| RPS6KB1 | AC011676.1 | 0.474186 | 2.40E-31 |
| ATG16L2 | LINC00115 | 0.474213 | 2.38E-31 |
| EIF2AK2 | AC004908.2 | 0.474214 | 2.38E-31 |
| RPS6KB1 | SCAANT1 | 0.474242 | 2.36E-31 |
| ATG2B | AC073569.2 | 0.474254 | 2.35E-31 |
| GOPC | AP001178.2 | 0.474259 | 2.35E-31 |
| RB1 | A2M-AS1 | 0.474283 | 2.33E-31 |
| FOXO1 | AC011477.2 | 0.47431 | 2.31E-31 |
| RPS6KB1 | AC002550.2 | 0.474329 | 2.29E-31 |
| MAPK8 | AL021578.1 | 0.474346 | 2.28E-31 |
| CAPN10 | AC037459.3 | 0.474368 | 2.26E-31 |
| MAPK8 | AC025171.3 | 0.474389 | 2.25E-31 |
| GOPC | OIP5-AS1 | 0.474416 | 2.23E-31 |
| RB1 | AC002064.2 | 0.474418 | 2.23E-31 |
| GOPC | AP002907.1 | 0.47442 | 2.22E-31 |
| RPS6KB1 | AL035409.1 | 0.47442 | 2.22E-31 |
| TSC1 | LINC01376 | 0.474507 | 2.16E-31 |
| KLHL24 | AL139011.1 | 0.474534 | 2.14E-31 |
| RB1CC1 | AC016542.1 | 0.474543 | 2.14E-31 |
| RB1CC1 | AL355488.1 | 0.474548 | 2.13E-31 |
| BIRC6 | LINC01184 | 0.474559 | 2.13E-31 |
| PTEN | AC008870.2 | 0.474564 | 2.12E-31 |
| GOPC | AC093799.1 | 0.474573 | 2.12E-31 |
| RB1CC1 | RRN3P2 | 0.474603 | 2.10E-31 |
| RB1 | AC105389.2 | 0.474606 | 2.09E-31 |
| GOPC | AL136320.1 | 0.474608 | 2.09E-31 |
| FOXO1 | AC022400.5 | 0.474642 | 2.07E-31 |
| CAPNS1 | AC002398.1 | 0.474724 | 2.01E-31 |
| ATG12 | AP003170.3 | 0.474736 | 2.01E-31 |
| KLHL24 | TPT1-AS1 | 0.474752 | 1.99E-31 |
| UVRAG | TRAF3IP2-AS1 | 0.474763 | 1.99E-31 |
| FOXO1 | AL357060.1 | 0.474791 | 1.97E-31 |
| ATG2B | LANCL1-AS1 | 0.474792 | 1.97E-31 |
| ATG4B | AL136295.7 | 0.474801 | 1.96E-31 |
| NAF1 | PPP3CB-AS1 | 0.47484 | 1.94E-31 |
| SIRT2 | AC020910.4 | 0.474843 | 1.94E-31 |
| CFLAR | BTBD9-AS1 | 0.474883 | 1.91E-31 |
| PIK3C3 | AL136531.1 | 0.474891 | 1.91E-31 |
| RPS6KB1 | AC116366.1 | 0.474918 | 1.89E-31 |
| ATG2B | AC012467.2 | 0.474952 | 1.87E-31 |
| RPS6KB1 | AP000692.1 | 0.474967 | 1.86E-31 |
| CFLAR | AC092123.1 | 0.474977 | 1.85E-31 |
| ATG12 | AC115989.1 | 0.474978 | 1.85E-31 |
| TSC1 | AC138932.5 | 0.474988 | 1.85E-31 |
| EIF2AK2 | KDM4A-AS1 | 0.47502 | 1.83E-31 |
| TSC1 | RFX3-AS1 | 0.475066 | 1.80E-31 |
| PIK3C3 | AC006378.1 | 0.475085 | 1.79E-31 |
| WDFY3 | AC109460.2 | 0.475098 | 1.78E-31 |
| GOPC | AC006213.4 | 0.475104 | 1.78E-31 |
| RB1 | BTBD9-AS1 | 0.47511 | 1.77E-31 |
| NAF1 | AC115989.1 | 0.475122 | 1.77E-31 |
| GOPC | AC005856.1 | 0.47514 | 1.76E-31 |
| ATG12 | AC016727.1 | 0.475229 | 1.70E-31 |
| FOXO1 | AC124312.2 | 0.475257 | 1.69E-31 |
| IFNG | LINC00861 | 0.475259 | 1.69E-31 |
| RPS6KB1 | LINC00513 | 0.475279 | 1.68E-31 |
| FOXO1 | AC026355.1 | 0.475282 | 1.68E-31 |
| NAF1 | AL360219.1 | 0.475289 | 1.67E-31 |
| NAF1 | AL355488.1 | 0.475307 | 1.66E-31 |
| ATG12 | AL683813.1 | 0.475312 | 1.66E-31 |
| RB1 | RHOA-IT1 | 0.475355 | 1.64E-31 |
| KLHL24 | AC011468.1 | 0.475375 | 1.63E-31 |
| RB1CC1 | AC096992.2 | 0.475375 | 1.62E-31 |
| NAF1 | C5orf56 | 0.475384 | 1.62E-31 |
| NAF1 | AC025171.3 | 0.475389 | 1.62E-31 |
| ATG2B | AC048341.1 | 0.475476 | 1.57E-31 |
| TSC1 | AC022211.1 | 0.475499 | 1.56E-31 |
| GOPC | AC004908.2 | 0.475511 | 1.55E-31 |
| TSC1 | AC005034.5 | 0.475515 | 1.55E-31 |
| ATG16L2 | AC015726.1 | 0.475553 | 1.53E-31 |
| CFLAR | SOS1-IT1 | 0.475554 | 1.53E-31 |
| RAB24 | AC010973.2 | 0.47556 | 1.53E-31 |
| RB1CC1 | Z98884.2 | 0.475595 | 1.51E-31 |
| TSC1 | AC138207.4 | 0.475597 | 1.51E-31 |
| GOPC | AL355488.1 | 0.475598 | 1.51E-31 |
| CASP1 | AL590764.1 | 0.475634 | 1.49E-31 |
| KLHL24 | AC090617.5 | 0.475635 | 1.49E-31 |
| ATG12 | AF131215.5 | 0.475662 | 1.48E-31 |
| RPS6KB1 | AC100830.2 | 0.475663 | 1.48E-31 |
| ATG16L2 | AC022167.2 | 0.475791 | 1.42E-31 |
| TSC1 | AC068790.5 | 0.475797 | 1.41E-31 |
| CFLAR | AC002553.1 | 0.475832 | 1.40E-31 |
| RB1CC1 | NUTM2A-AS1 | 0.475857 | 1.39E-31 |
| FOXO3 | AL592148.3 | 0.475859 | 1.39E-31 |
| NAF1 | AC010226.1 | 0.475877 | 1.38E-31 |
| RB1 | AC253536.3 | 0.475901 | 1.37E-31 |
| RB1CC1 | LINC00641 | 0.475957 | 1.34E-31 |
| RB1CC1 | TMEM161B-AS1 | 0.475958 | 1.34E-31 |
| RPS6KB1 | AC005034.3 | 0.47598 | 1.33E-31 |
| WDFY3 | AC019131.2 | 0.476006 | 1.32E-31 |
| NAF1 | AC018690.1 | 0.476015 | 1.32E-31 |
| RB1CC1 | AC067852.3 | 0.476034 | 1.31E-31 |
| ULK3 | AP006284.1 | 0.476063 | 1.30E-31 |
| TSC1 | AC011472.1 | 0.476086 | 1.29E-31 |
| ATG2B | AL121772.3 | 0.476103 | 1.28E-31 |
| ATG2B | AC062037.2 | 0.476116 | 1.27E-31 |
| TSC1 | AC007938.3 | 0.476121 | 1.27E-31 |
| RB1 | AC018926.3 | 0.476127 | 1.27E-31 |
| ATG16L2 | AL359881.1 | 0.476177 | 1.25E-31 |
| KLHL24 | AC093799.1 | 0.476182 | 1.25E-31 |
| CFLAR | AL365277.1 | 0.476197 | 1.24E-31 |
| FOXO1 | LINC01355 | 0.476201 | 1.24E-31 |
| PIK3C3 | AC009948.1 | 0.476214 | 1.23E-31 |
| ATG7 | AC005920.2 | 0.476221 | 1.23E-31 |
| NAF1 | AC009054.2 | 0.476254 | 1.22E-31 |
| RB1CC1 | AC002044.1 | 0.476279 | 1.21E-31 |
| ATG16L2 | LINC00685 | 0.476319 | 1.19E-31 |
| FOXO3 | AC058791.1 | 0.476337 | 1.18E-31 |
| GOPC | AC007216.4 | 0.476338 | 1.18E-31 |
| TSC1 | AC004492.1 | 0.476348 | 1.18E-31 |
| TSC1 | AC018926.3 | 0.476359 | 1.17E-31 |
| KLHL24 | AC005519.1 | 0.476394 | 1.16E-31 |
| CFLAR | AP000786.1 | 0.476398 | 1.16E-31 |
| KLHL24 | AC018690.1 | 0.476413 | 1.15E-31 |
| RPS6KB1 | AC018926.3 | 0.476438 | 1.14E-31 |
| RB1CC1 | SP2-AS1 | 0.476461 | 1.14E-31 |
| PIK3C3 | KDM4A-AS1 | 0.476464 | 1.13E-31 |
| EIF2AK2 | AC141002.1 | 0.476491 | 1.12E-31 |
| PTEN | LINC01655 | 0.476525 | 1.11E-31 |
| RB1CC1 | AC002550.2 | 0.47664 | 1.07E-31 |
| PIK3C3 | AL158166.1 | 0.476643 | 1.07E-31 |
| GOPC | NUTM2B-AS1 | 0.476651 | 1.07E-31 |
| CFLAR | AC011468.5 | 0.476658 | 1.06E-31 |
| ATG16L2 | AL691482.3 | 0.476706 | 1.05E-31 |
| CCL2 | AC147651.3 | 0.476717 | 1.04E-31 |
| CFLAR | AC092611.2 | 0.476744 | 1.03E-31 |
| RB1 | AC008906.1 | 0.476792 | 1.02E-31 |
| EIF2AK2 | RRN3P2 | 0.476796 | 1.02E-31 |
| GOPC | AC068790.3 | 0.476846 | 1.00E-31 |
| EIF2AK2 | AC097641.2 | 0.47687 | 9.92E-32 |
| PIK3C3 | AP001160.4 | 0.476881 | 9.88E-32 |
| MAPK8 | AL450263.1 | 0.476906 | 9.80E-32 |
| EIF2AK2 | AC024075.1 | 0.476925 | 9.74E-32 |
| ATG12 | AC004477.3 | 0.476944 | 9.68E-32 |
| MAPK8 | LINC02352 | 0.476971 | 9.59E-32 |
| DAPK2 | AC135050.6 | 0.476992 | 9.53E-32 |
| ATG12 | AC022211.1 | 0.477008 | 9.48E-32 |
| RB1CC1 | AC097641.2 | 0.477131 | 9.10E-32 |
| ATG12 | AC092123.1 | 0.477179 | 8.95E-32 |
| ATG7 | ITCH-IT1 | 0.477183 | 8.94E-32 |
| RPS6KB1 | AC084871.1 | 0.477184 | 8.94E-32 |
| NAF1 | DUBR | 0.477251 | 8.74E-32 |
| RPS6KB1 | AC096741.1 | 0.477273 | 8.68E-32 |
| TSC1 | NDUFV2-AS1 | 0.47728 | 8.66E-32 |
| PIK3C3 | AL359962.2 | 0.477285 | 8.64E-32 |
| TSC1 | AC073389.3 | 0.477287 | 8.64E-32 |
| KLHL24 | AC138207.4 | 0.477296 | 8.61E-32 |
| CFLAR | EGOT | 0.477315 | 8.56E-32 |
| WDFY3 | IGBP1-AS1 | 0.477317 | 8.55E-32 |
| ATG10 | AC008771.1 | 0.477319 | 8.55E-32 |
| CFLAR | AL049840.3 | 0.477325 | 8.53E-32 |
| EIF2AK2 | AC092794.1 | 0.477332 | 8.51E-32 |
| GOPC | CFLAR-AS1 | 0.477342 | 8.48E-32 |
| MAPK8 | AC022784.5 | 0.477349 | 8.46E-32 |
| MAPK8 | AL137782.1 | 0.477365 | 8.42E-32 |
| KLHL24 | AC074033.1 | 0.477374 | 8.39E-32 |
| RB1 | LINC-PINT | 0.477392 | 8.34E-32 |
| PTEN | AC104083.1 | 0.477398 | 8.33E-32 |
| FOXO1 | AL157394.1 | 0.4774 | 8.32E-32 |
| GOPC | AC007546.1 | 0.47744 | 8.21E-32 |
| ATG12 | LINC01376 | 0.477465 | 8.14E-32 |
| KLHL24 | LINC02352 | 0.477479 | 8.11E-32 |
| EIF2AK2 | AL157932.1 | 0.4775 | 8.05E-32 |
| TSC1 | AP001160.4 | 0.477581 | 7.84E-32 |
| FOXO1 | AC084824.4 | 0.477606 | 7.77E-32 |
| TSC1 | SDCBP2-AS1 | 0.477612 | 7.76E-32 |
| ATG12 | AC010542.5 | 0.477612 | 7.75E-32 |
| TSC1 | AC090948.2 | 0.47762 | 7.73E-32 |
| NAF1 | ATP1A1-AS1 | 0.477635 | 7.70E-32 |
| CFLAR | ADNP-AS1 | 0.47769 | 7.56E-32 |
| RB1CC1 | AC005540.1 | 0.47771 | 7.51E-32 |
| NAF1 | AC139887.2 | 0.477742 | 7.43E-32 |
| SPNS1 | AC012615.6 | 0.477743 | 7.43E-32 |
| PTEN | AL121772.3 | 0.477766 | 7.37E-32 |
| CASP1 | LINC00996 | 0.477767 | 7.37E-32 |
| EIF2AK2 | AC006378.1 | 0.477791 | 7.31E-32 |
| RB1CC1 | AL137779.2 | 0.477792 | 7.31E-32 |
| RB1CC1 | AC022400.5 | 0.477816 | 7.25E-32 |
| PIK3C3 | AC000123.1 | 0.477839 | 7.19E-32 |
| KLHL24 | AL021707.7 | 0.477845 | 7.18E-32 |
| ATG16L2 | AC020916.1 | 0.477853 | 7.16E-32 |
| RPS6KB1 | DNM3OS | 0.477873 | 7.11E-32 |
| RB1CC1 | LINC01534 | 0.477876 | 7.11E-32 |
| RB1CC1 | AC027097.1 | 0.477879 | 7.10E-32 |
| RPS6KB1 | AC245014.3 | 0.477895 | 7.06E-32 |
| TSC1 | AC253536.3 | 0.477908 | 7.03E-32 |
| NAF1 | AL391001.1 | 0.477934 | 6.97E-32 |
| TSC1 | MIR29B2CHG | 0.477949 | 6.94E-32 |
| PIK3C3 | AC069023.1 | 0.477993 | 6.83E-32 |
| FOXO1 | AC006017.1 | 0.478007 | 6.80E-32 |
| ATG2B | AL354696.1 | 0.478042 | 6.72E-32 |
| MAPK8 | AC027277.2 | 0.478062 | 6.68E-32 |
| TSC1 | AC004223.3 | 0.478089 | 6.62E-32 |
| CAPN10 | AC006435.2 | 0.478096 | 6.60E-32 |
| PIK3C3 | AC024933.1 | 0.478102 | 6.59E-32 |
| BIRC6 | AC083862.2 | 0.478112 | 6.57E-32 |
| RB1CC1 | AC008966.2 | 0.478138 | 6.51E-32 |
| EIF2AK2 | AC010168.2 | 0.478163 | 6.46E-32 |
| NAF1 | AC000123.1 | 0.478178 | 6.43E-32 |
| FOXO3 | AL049840.5 | 0.478179 | 6.42E-32 |
| FOXO1 | AC141002.1 | 0.478211 | 6.36E-32 |
| ATG16L2 | AGBL5-IT1 | 0.478222 | 6.33E-32 |
| CFLAR | AC027097.2 | 0.478227 | 6.32E-32 |
| PEA15 | LINC01094 | 0.478234 | 6.31E-32 |
| ATG16L2 | AC009974.1 | 0.478271 | 6.23E-32 |
| KLHL24 | C5orf56 | 0.47832 | 6.13E-32 |
| GOPC | AL049869.3 | 0.478337 | 6.10E-32 |
| CFLAR | AL162586.1 | 0.478355 | 6.06E-32 |
| EIF2AK2 | OIP5-AS1 | 0.47838 | 6.01E-32 |
| RPS6KB1 | AC107027.3 | 0.478381 | 6.01E-32 |
| MAPK8 | PSMA3-AS1 | 0.4784 | 5.97E-32 |
| MAPK8 | AL031716.1 | 0.478442 | 5.89E-32 |
| RPS6KB1 | SRD5A3-AS1 | 0.47846 | 5.85E-32 |
| PIK3C3 | AC009054.2 | 0.478472 | 5.83E-32 |
| GOPC | SAP30L-AS1 | 0.478489 | 5.79E-32 |
| CAPN10 | PRKCZ-AS1 | 0.478491 | 5.79E-32 |
| GOPC | AC009032.1 | 0.478515 | 5.75E-32 |
| RPS6KB1 | AL359076.1 | 0.478538 | 5.70E-32 |
| ATG2B | AC093484.4 | 0.478546 | 5.69E-32 |
| ATG16L2 | CAPN10-DT | 0.478639 | 5.51E-32 |
| CFLAR | AC026356.1 | 0.478644 | 5.50E-32 |
| ATG4B | FLJ46906 | 0.478667 | 5.46E-32 |
| EIF2AK2 | AC015813.1 | 0.478744 | 5.32E-32 |
| RB1 | AL365277.1 | 0.478815 | 5.20E-32 |
| ATG4B | AC093752.3 | 0.478835 | 5.16E-32 |
| EIF2AK2 | AP000866.6 | 0.47884 | 5.16E-32 |
| ATG2B | AL603839.3 | 0.478846 | 5.15E-32 |
| PIK3C3 | AC141002.1 | 0.478849 | 5.14E-32 |
| ATG12 | AC021851.1 | 0.478851 | 5.14E-32 |
| FOXO3 | AL031670.1 | 0.478857 | 5.13E-32 |
| FOXO3 | AC007546.1 | 0.478861 | 5.12E-32 |
| CFLAR | MUC20-OT1 | 0.478864 | 5.11E-32 |
| TSC2 | AC114730.3 | 0.478881 | 5.09E-32 |
| TSC1 | HM13-IT1 | 0.478923 | 5.02E-32 |
| RB1CC1 | AC018682.1 | 0.478973 | 4.93E-32 |
| CFLAR | AC093388.1 | 0.47899 | 4.90E-32 |
| ATG16L2 | AC093151.2 | 0.478992 | 4.90E-32 |
| GOPC | AL606834.2 | 0.479004 | 4.88E-32 |
| SPNS1 | AC114730.3 | 0.479009 | 4.87E-32 |
| RAF1 | THUMPD3-AS1 | 0.479022 | 4.85E-32 |
| GOPC | AC005838.2 | 0.479025 | 4.85E-32 |
| FOXO3 | AC124312.4 | 0.479028 | 4.84E-32 |
| MAPK8 | AC026368.1 | 0.479056 | 4.80E-32 |
| RB1CC1 | FAM13A-AS1 | 0.47908 | 4.76E-32 |
| ATG12 | LINC01290 | 0.479095 | 4.74E-32 |
| RB1 | DLEU1 | 0.47911 | 4.71E-32 |
| ATG2B | LINC00893 | 0.479118 | 4.70E-32 |
| KLHL24 | AC011442.1 | 0.479177 | 4.61E-32 |
| NAF1 | AC004466.3 | 0.479195 | 4.58E-32 |
| CFLAR | AC083949.1 | 0.479199 | 4.57E-32 |
| RB1 | AL136320.1 | 0.479237 | 4.52E-32 |
| RPS6KB1 | AL359962.2 | 0.47931 | 4.41E-32 |
| FOXO3 | AC007216.3 | 0.479329 | 4.38E-32 |
| RPS6KB1 | AC005034.5 | 0.479345 | 4.36E-32 |
| CFLAR | ZNF460-AS1 | 0.479348 | 4.35E-32 |
| RB1CC1 | AC092794.1 | 0.479372 | 4.32E-32 |
| RPS6KB1 | AC090517.2 | 0.479382 | 4.30E-32 |
| CFLAR | AL162274.2 | 0.479389 | 4.29E-32 |
| NAF1 | AC138956.2 | 0.479391 | 4.29E-32 |
| PIK3C3 | AC005034.5 | 0.479393 | 4.29E-32 |
| PTEN | AC113139.1 | 0.479393 | 4.29E-32 |
| RPS6KB1 | NDUFV2-AS1 | 0.479415 | 4.26E-32 |
| PIK3C3 | RBMS3-AS3 | 0.479418 | 4.25E-32 |
| NAF1 | SP2-AS1 | 0.479428 | 4.24E-32 |
| ATG4B | MMP25-AS1 | 0.479437 | 4.22E-32 |
| RAB24 | TMEM147-AS1 | 0.47945 | 4.21E-32 |
| CFLAR | AC068790.2 | 0.479463 | 4.19E-32 |
| MAPK8 | AC026356.1 | 0.479468 | 4.18E-32 |
| TSC1 | AL049840.4 | 0.479481 | 4.16E-32 |
| CFLAR | AC092794.1 | 0.479481 | 4.16E-32 |
| IFNG | DBH-AS1 | 0.479543 | 4.08E-32 |
| EIF2AK2 | AC005104.1 | 0.479594 | 4.01E-32 |
| FOXO1 | AC093110.1 | 0.479612 | 3.99E-32 |
| GOPC | AC022173.1 | 0.479646 | 3.94E-32 |
| EIF2AK2 | AC120349.1 | 0.479661 | 3.92E-32 |
| FOXO3 | MAL2-AS1 | 0.479675 | 3.90E-32 |
| NAF1 | AC124319.2 | 0.479675 | 3.90E-32 |
| RB1CC1 | AC090517.2 | 0.479687 | 3.89E-32 |
| WDFY3 | AC104819.3 | 0.479721 | 3.84E-32 |
| FOXO3 | AL049552.1 | 0.479721 | 3.84E-32 |
| TSC1 | AL109614.1 | 0.479723 | 3.84E-32 |
| MAP2K7 | AC114730.3 | 0.479727 | 3.83E-32 |
| GOPC | AL355075.2 | 0.479735 | 3.82E-32 |
| KLHL24 | AP000866.6 | 0.479776 | 3.77E-32 |
| ATG2B | PDXDC2P-NPIPB14P | 0.479778 | 3.77E-32 |
| MAPK8 | NFYC-AS1 | 0.479786 | 3.76E-32 |
| ATG7 | CD44-AS1 | 0.479793 | 3.75E-32 |
| ATG2B | AL035409.1 | 0.479797 | 3.75E-32 |
| GABARAP | PITPNA-AS1 | 0.479805 | 3.74E-32 |
| FOXO3 | AC005856.1 | 0.479826 | 3.71E-32 |
| PIK3C3 | AC073046.1 | 0.479832 | 3.70E-32 |
| GOPC | ANKRD10-IT1 | 0.479899 | 3.62E-32 |
| GOPC | AC004223.3 | 0.479905 | 3.61E-32 |
| FOXO1 | AC016957.2 | 0.479914 | 3.60E-32 |
| CFLAR | AC018682.1 | 0.479933 | 3.58E-32 |
| PIK3C3 | AL031670.1 | 0.479966 | 3.54E-32 |
| RB1 | AL049869.3 | 0.479975 | 3.53E-32 |
| CFLAR | AL162724.2 | 0.479986 | 3.52E-32 |
| NAF1 | AC024933.1 | 0.480004 | 3.50E-32 |
| PIK3C3 | AC009120.3 | 0.48002 | 3.48E-32 |
| GOPC | AC002550.2 | 0.480046 | 3.45E-32 |
| EIF2AK2 | AGAP1-IT1 | 0.480057 | 3.43E-32 |
| RPS6KB1 | AL137003.2 | 0.480076 | 3.41E-32 |
| RPS6KB1 | SNHG26 | 0.480093 | 3.39E-32 |
| ATG16L2 | AC135050.6 | 0.480094 | 3.39E-32 |
| TSC1 | AL355574.1 | 0.480128 | 3.35E-32 |
| GOPC | AC015849.3 | 0.480147 | 3.33E-32 |
| CAPN10 | AC087741.1 | 0.480152 | 3.33E-32 |
| ATG16L2 | GAS6-AS1 | 0.480179 | 3.30E-32 |
| GOPC | ITCH-IT1 | 0.480227 | 3.24E-32 |
| ATG4B | AL691482.3 | 0.480245 | 3.22E-32 |
| RB1 | NUTM2B-AS1 | 0.480276 | 3.19E-32 |
| CFLAR | AC005253.1 | 0.48029 | 3.18E-32 |
| TSC1 | AC007038.1 | 0.480298 | 3.17E-32 |
| BIRC6 | ACBD3-AS1 | 0.48031 | 3.16E-32 |
| ATG12 | AC139887.1 | 0.480312 | 3.15E-32 |
| TSC1 | AC048341.1 | 0.480367 | 3.10E-32 |
| NAF1 | AC005632.2 | 0.480369 | 3.09E-32 |
| FOXO3 | AL513365.2 | 0.480376 | 3.09E-32 |
| PIK3C3 | AC084871.1 | 0.480456 | 3.00E-32 |
| ATG16L2 | AC007566.1 | 0.480479 | 2.98E-32 |
| MAPK8 | ATP1B3-AS1 | 0.480491 | 2.97E-32 |
| BIRC6 | XIST | 0.480493 | 2.97E-32 |
| NAF1 | AC107027.3 | 0.480493 | 2.97E-32 |
| ULK3 | AC003070.1 | 0.480511 | 2.95E-32 |
| CFLAR | DLEU2 | 0.48052 | 2.94E-32 |
| RPS6KB1 | FMR1-IT1 | 0.480537 | 2.92E-32 |
| MAPK8 | MIR29B2CHG | 0.480555 | 2.91E-32 |
| EIF2AK2 | AL132989.1 | 0.480578 | 2.88E-32 |
| CAPN10 | AC073842.2 | 0.480594 | 2.87E-32 |
| GOPC | AC090948.1 | 0.480618 | 2.85E-32 |
| KLHL24 | AC010168.2 | 0.480661 | 2.80E-32 |
| RB1 | AL049840.1 | 0.480705 | 2.76E-32 |
| ATG4B | LINC00106 | 0.480814 | 2.66E-32 |
| GOPC | AC067852.3 | 0.480834 | 2.65E-32 |
| PIK3C3 | AC093388.1 | 0.480869 | 2.62E-32 |
| ATG16L2 | ITGB2-AS1 | 0.480872 | 2.61E-32 |
| ATG4B | AP002807.1 | 0.480887 | 2.60E-32 |
| WDFY3 | AP000442.2 | 0.480893 | 2.60E-32 |
| KLHL24 | AC084824.4 | 0.480913 | 2.58E-32 |
| VEGFA | AL645608.8 | 0.480917 | 2.57E-32 |
| MAP2K7 | AC011498.6 | 0.480952 | 2.54E-32 |
| GOPC | AC016831.4 | 0.480987 | 2.52E-32 |
| PTEN | MUC20-OT1 | 0.481001 | 2.50E-32 |
| ITPR1 | OIP5-AS1 | 0.481012 | 2.49E-32 |
| ULK3 | SNHG12 | 0.481024 | 2.48E-32 |
| BIRC6 | AL021878.2 | 0.481089 | 2.43E-32 |
| RB1CC1 | MACC1-AS1 | 0.48111 | 2.41E-32 |
| DNAJB9 | LINC00582 | 0.481117 | 2.41E-32 |
| GOPC | AC007038.2 | 0.481125 | 2.40E-32 |
| WDFY3 | AC005253.1 | 0.481131 | 2.40E-32 |
| TSC1 | GMDS-DT | 0.481133 | 2.39E-32 |
| ATG2B | AC016727.1 | 0.481193 | 2.35E-32 |
| ATG12 | AL133371.2 | 0.481193 | 2.35E-32 |
| MAPK8 | AC084824.5 | 0.481215 | 2.33E-32 |
| CAPN10 | LINC00265 | 0.481266 | 2.29E-32 |
| RB1 | AC020571.1 | 0.481271 | 2.29E-32 |
| PIK3C3 | AL513327.1 | 0.481285 | 2.28E-32 |
| FOXO1 | AL360219.1 | 0.481296 | 2.27E-32 |
| NAF1 | AF178030.1 | 0.481299 | 2.26E-32 |
| RB1CC1 | AL157838.1 | 0.481311 | 2.26E-32 |
| CFLAR | AC068790.3 | 0.481316 | 2.25E-32 |
| ATG16L2 | AC002128.1 | 0.481352 | 2.22E-32 |
| CFLAR | AC005332.4 | 0.481387 | 2.20E-32 |
| PIK3C3 | AC016394.1 | 0.48139 | 2.20E-32 |
| ATG12 | AC005519.1 | 0.481434 | 2.16E-32 |
| MAPK8 | AC009318.2 | 0.481438 | 2.16E-32 |
| GOPC | AC019080.5 | 0.481474 | 2.14E-32 |
| BIRC6 | ATP13A4-AS1 | 0.481487 | 2.13E-32 |
| FOXO3 | AL049840.1 | 0.481497 | 2.12E-32 |
| PTEN | PAXIP1-AS2 | 0.481506 | 2.11E-32 |
| PTEN | SAMD12-AS1 | 0.481567 | 2.07E-32 |
| RAB24 | AC016773.1 | 0.481633 | 2.02E-32 |
| FOXO3 | AC005070.3 | 0.481647 | 2.01E-32 |
| PTEN | AL031673.1 | 0.481674 | 2.00E-32 |
| EIF2AK2 | AC005261.1 | 0.481675 | 2.00E-32 |
| ATG2B | CCDC18-AS1 | 0.481697 | 1.98E-32 |
| KLHL24 | AC011468.5 | 0.481722 | 1.96E-32 |
| PIK3C3 | AL132989.1 | 0.481746 | 1.95E-32 |
| MAPK8 | AC018690.1 | 0.481805 | 1.91E-32 |
| WDFY3 | ACBD3-AS1 | 0.481858 | 1.88E-32 |
| PTEN | AC009948.4 | 0.48186 | 1.88E-32 |
| FOXO1 | AC004908.2 | 0.481872 | 1.87E-32 |
| NAF1 | AL662844.3 | 0.481881 | 1.86E-32 |
| RB1CC1 | AC004466.3 | 0.481882 | 1.86E-32 |
| RB1CC1 | FMR1-IT1 | 0.481932 | 1.83E-32 |
| FOXO1 | AC090425.2 | 0.48194 | 1.83E-32 |
| MAPK8 | AC018682.1 | 0.481957 | 1.82E-32 |
| BNIP1 | TRIM52-AS1 | 0.481982 | 1.80E-32 |
| ATG12 | AC007991.2 | 0.482046 | 1.76E-32 |
| RB1 | AC026124.2 | 0.482067 | 1.75E-32 |
| ATG12 | ZKSCAN2-DT | 0.482074 | 1.75E-32 |
| ATG12 | ATP13A4-AS1 | 0.482082 | 1.74E-32 |
| KLHL24 | AC007996.1 | 0.482108 | 1.73E-32 |
| CAPN10 | AL031186.1 | 0.48213 | 1.71E-32 |
| CFLAR | AGAP1-IT1 | 0.482139 | 1.71E-32 |
| WDFY3 | AP001528.2 | 0.482164 | 1.69E-32 |
| BIRC6 | AC123595.1 | 0.482167 | 1.69E-32 |
| EIF2AK2 | PPP3CB-AS1 | 0.482169 | 1.69E-32 |
| RPS6KB1 | LINC01578 | 0.482186 | 1.68E-32 |
| KLHL24 | AL121839.2 | 0.482192 | 1.68E-32 |
| TSC1 | N4BP2L2-IT2 | 0.482209 | 1.67E-32 |
| ATG7 | AC026356.1 | 0.482257 | 1.64E-32 |
| PTEN | AL117379.1 | 0.48226 | 1.64E-32 |
| EIF2AK2 | AC004596.1 | 0.482292 | 1.62E-32 |
| ATG7 | AC018682.1 | 0.482385 | 1.57E-32 |
| GOPC | AC008906.1 | 0.482427 | 1.55E-32 |
| KLHL24 | MIATNB | 0.482448 | 1.54E-32 |
| FOXO3 | AC009041.4 | 0.482536 | 1.49E-32 |
| RB1CC1 | AC124312.4 | 0.48254 | 1.49E-32 |
| FOXO1 | AC007038.2 | 0.482545 | 1.49E-32 |
| PIK3C3 | AC011468.5 | 0.48255 | 1.49E-32 |
| EIF2AK2 | OSMR-AS1 | 0.482583 | 1.47E-32 |
| GOPC | AC090948.3 | 0.482614 | 1.45E-32 |
| KLHL24 | AC022400.5 | 0.482615 | 1.45E-32 |
| FOXO1 | AC008124.1 | 0.482669 | 1.43E-32 |
| HGS | AC139530.1 | 0.48269 | 1.42E-32 |
| FOXO3 | ITCH-IT1 | 0.482694 | 1.42E-32 |
| CFLAR | AC087481.3 | 0.482714 | 1.41E-32 |
| FOXO3 | Z68871.1 | 0.482743 | 1.39E-32 |
| FOXO1 | FLNB-AS1 | 0.482757 | 1.39E-32 |
| BIRC6 | AC130456.3 | 0.482764 | 1.38E-32 |
| RPS6KB1 | MALAT1 | 0.482768 | 1.38E-32 |
| CFLAR | KIF26B-AS1 | 0.482825 | 1.35E-32 |
| FOXO3 | MBNL1-AS1 | 0.482826 | 1.35E-32 |
| ATG12 | AC124319.1 | 0.482862 | 1.34E-32 |
| EIF2AK2 | KIF26B-AS1 | 0.482867 | 1.34E-32 |
| RB1 | AC005920.2 | 0.482887 | 1.33E-32 |
| PIK3C3 | AL354989.1 | 0.482893 | 1.32E-32 |
| BIRC6 | AC006001.2 | 0.482899 | 1.32E-32 |
| FOXO3 | ACAP2-IT1 | 0.482903 | 1.32E-32 |
| RPS6KB1 | AC018682.1 | 0.482965 | 1.29E-32 |
| TSC1 | AL022328.2 | 0.482983 | 1.28E-32 |
| ATG16L2 | AC093752.3 | 0.483007 | 1.27E-32 |
| GOPC | AC110792.3 | 0.483046 | 1.26E-32 |
| NAF1 | ATP1B3-AS1 | 0.483078 | 1.24E-32 |
| KLHL24 | AL157786.1 | 0.483082 | 1.24E-32 |
| MAPK8 | AP003170.3 | 0.483142 | 1.22E-32 |
| IFNG | AL157871.2 | 0.483157 | 1.21E-32 |
| WDFY3 | AL359962.1 | 0.483157 | 1.21E-32 |
| TSC1 | TAPT1-AS1 | 0.483205 | 1.19E-32 |
| RB1CC1 | SNHG26 | 0.483217 | 1.19E-32 |
| PTEN | AL683813.1 | 0.483279 | 1.16E-32 |
| CAPN10 | AL365330.1 | 0.4833 | 1.15E-32 |
| RB1CC1 | AL031670.1 | 0.483316 | 1.15E-32 |
| RB1 | ANKRD44-IT1 | 0.483373 | 1.13E-32 |
| RPS6KB1 | AC007216.4 | 0.483379 | 1.12E-32 |
| CFLAR | AC000123.1 | 0.483385 | 1.12E-32 |
| ATG2B | AC104083.1 | 0.483466 | 1.09E-32 |
| GOPC | AC068790.2 | 0.483554 | 1.06E-32 |
| FOXO1 | AL645568.1 | 0.483599 | 1.04E-32 |
| GOPC | AC007216.3 | 0.483602 | 1.04E-32 |
| TSC1 | AC090948.1 | 0.483606 | 1.04E-32 |
| ATG3 | LINC01094 | 0.48362 | 1.04E-32 |
| ATG2B | USP46-AS1 | 0.483628 | 1.03E-32 |
| EIF2AK2 | AP000692.1 | 0.483643 | 1.03E-32 |
| WDFY3 | DGUOK-AS1 | 0.483662 | 1.02E-32 |
| PIK3R4 | AC073569.2 | 0.483694 | 1.01E-32 |
| NAF1 | AC007014.2 | 0.483706 | 1.01E-32 |
| GOPC | AL157392.3 | 0.483718 | 1.00E-32 |
| FOXO3 | AP001429.1 | 0.483739 | 9.95E-33 |
| CAPN10 | MHENCR | 0.483745 | 9.92E-33 |
| ATG2B | SAMD12-AS1 | 0.483747 | 9.92E-33 |
| CFLAR | AL031716.1 | 0.483763 | 9.86E-33 |
| ATG7 | AC026470.2 | 0.483768 | 9.85E-33 |
| FOXO1 | KIF26B-AS1 | 0.483819 | 9.68E-33 |
| KLHL24 | AC105389.2 | 0.483822 | 9.67E-33 |
| RB1 | AC090948.1 | 0.483827 | 9.65E-33 |
| WDFY3 | XIST | 0.483851 | 9.57E-33 |
| TSC1 | AC100830.2 | 0.483907 | 9.40E-33 |
| CFLAR | AC253536.3 | 0.483914 | 9.37E-33 |
| PIK3C3 | AC090517.2 | 0.483945 | 9.27E-33 |
| CFLAR | AC011468.1 | 0.483984 | 9.15E-33 |
| PIK3C3 | AL355075.2 | 0.483991 | 9.13E-33 |
| TSC2 | AC069281.2 | 0.484001 | 9.10E-33 |
| TSC1 | AL391834.1 | 0.484008 | 9.08E-33 |
| TSC1 | LINC02352 | 0.484033 | 9.00E-33 |
| FOXO3 | AC004492.1 | 0.484091 | 8.83E-33 |
| GOPC | AC253536.3 | 0.484093 | 8.82E-33 |
| MAPK8 | AC245014.3 | 0.484116 | 8.75E-33 |
| BIRC6 | AC020571.1 | 0.484174 | 8.58E-33 |
| MAPK8 | AL391001.1 | 0.484205 | 8.49E-33 |
| ATG16L2 | AL353622.1 | 0.484208 | 8.48E-33 |
| TSC1 | AC008537.2 | 0.48426 | 8.33E-33 |
| KLHL24 | AC025178.1 | 0.484269 | 8.31E-33 |
| KLHL24 | AC073046.1 | 0.484282 | 8.27E-33 |
| FOXO1 | AC010226.1 | 0.484283 | 8.27E-33 |
| BIRC6 | AC124016.2 | 0.484285 | 8.26E-33 |
| CFLAR | AP003170.3 | 0.48429 | 8.25E-33 |
| EIF2AK2 | AC093799.1 | 0.484297 | 8.23E-33 |
| RB1 | AC004492.1 | 0.484299 | 8.22E-33 |
| TSC1 | AC011476.3 | 0.484347 | 8.09E-33 |
| PIK3C3 | AC004466.3 | 0.484372 | 8.02E-33 |
| KLHL24 | MALAT1 | 0.484376 | 8.01E-33 |
| RAB24 | SNHG12 | 0.48438 | 8.00E-33 |
| KLHL24 | AP000692.1 | 0.484412 | 7.92E-33 |
| EIF2AK2 | AC090198.1 | 0.484438 | 7.85E-33 |
| RAB24 | AC087741.1 | 0.48447 | 7.76E-33 |
| GOPC | AC010834.3 | 0.484488 | 7.71E-33 |
| RB1CC1 | OIP5-AS1 | 0.484509 | 7.66E-33 |
| SPNS1 | AL022328.2 | 0.484534 | 7.60E-33 |
| ATG2B | AC130456.3 | 0.484548 | 7.56E-33 |
| ATG7 | AC006059.1 | 0.484553 | 7.55E-33 |
| RB1CC1 | RFX3-AS1 | 0.484642 | 7.32E-33 |
| CFLAR | AC048341.2 | 0.484652 | 7.30E-33 |
| RPS6KB1 | AC008966.2 | 0.484669 | 7.26E-33 |
| CFLAR | AL731566.1 | 0.484684 | 7.22E-33 |
| RPS6KB1 | AL353804.1 | 0.484695 | 7.19E-33 |
| EEF2K | AC009041.4 | 0.484697 | 7.18E-33 |
| TSC1 | AP001432.1 | 0.48471 | 7.15E-33 |
| ATG7 | AL137779.2 | 0.484807 | 6.92E-33 |
| RPS6KB1 | AC022400.5 | 0.484815 | 6.90E-33 |
| NAF1 | AL359962.2 | 0.484858 | 6.80E-33 |
| CFLAR | AC093726.1 | 0.484864 | 6.79E-33 |
| RPS6KB1 | FAM13A-AS1 | 0.48488 | 6.75E-33 |
| SPNS1 | AC132872.3 | 0.484898 | 6.71E-33 |
| PIK3C3 | AC011468.1 | 0.484912 | 6.68E-33 |
| EIF2AK2 | AL450263.1 | 0.484914 | 6.67E-33 |
| GOPC | AP001429.1 | 0.485033 | 6.41E-33 |
| RPS6KB1 | AC090579.1 | 0.48504 | 6.39E-33 |
| ATG7 | AC080162.1 | 0.485051 | 6.37E-33 |
| TP73 | AC234582.1 | 0.485077 | 6.32E-33 |
| MAPK8 | AL354989.1 | 0.485089 | 6.29E-33 |
| EIF2AK2 | AL662844.3 | 0.485115 | 6.23E-33 |
| PIK3C3 | AC015911.3 | 0.485193 | 6.07E-33 |
| KLHL24 | AC018638.7 | 0.485233 | 5.99E-33 |
| PTEN | AC025165.4 | 0.485279 | 5.90E-33 |
| RB1 | AC097376.2 | 0.485282 | 5.89E-33 |
| NAF1 | AC084824.4 | 0.485296 | 5.86E-33 |
| ATG16L2 | AC015961.2 | 0.485301 | 5.85E-33 |
| MAPK8 | AL049840.2 | 0.485311 | 5.83E-33 |
| RB1 | AC083949.1 | 0.485318 | 5.82E-33 |
| GOPC | AP000866.6 | 0.485341 | 5.77E-33 |
| CAPN10 | AL022328.1 | 0.485346 | 5.76E-33 |
| NAF1 | AL157932.1 | 0.485359 | 5.74E-33 |
| GOPC | ANKRD44-IT1 | 0.485361 | 5.73E-33 |
| MAPK8 | AC007552.2 | 0.485386 | 5.68E-33 |
| FOXO3 | ANKRD44-IT1 | 0.485407 | 5.64E-33 |
| NAF1 | AP000692.1 | 0.485456 | 5.55E-33 |
| NAF1 | AC098484.1 | 0.485458 | 5.55E-33 |
| ATG7 | AL133445.2 | 0.485462 | 5.54E-33 |
| ATG2B | LINC01578 | 0.485477 | 5.51E-33 |
| TSC1 | AL162274.2 | 0.485478 | 5.51E-33 |
| ATG2B | AC004241.3 | 0.485484 | 5.50E-33 |
| RPS6KB1 | AC009318.3 | 0.485494 | 5.48E-33 |
| RPS6KB1 | AC073651.1 | 0.4855 | 5.47E-33 |
| NAF1 | AC048344.4 | 0.485503 | 5.46E-33 |
| ATG12 | TPT1-AS1 | 0.485512 | 5.45E-33 |
| TSC1 | NPTN-IT1 | 0.485565 | 5.35E-33 |
| TSC1 | AP003170.3 | 0.485566 | 5.35E-33 |
| FOXO1 | AC005104.1 | 0.485575 | 5.33E-33 |
| NAF1 | AC078846.1 | 0.485609 | 5.27E-33 |
| TSC1 | AC078778.1 | 0.485623 | 5.24E-33 |
| RPS6KB1 | AC090198.1 | 0.485633 | 5.23E-33 |
| GOPC | AL122035.1 | 0.485684 | 5.14E-33 |
| GOPC | AL137782.1 | 0.485709 | 5.09E-33 |
| FOXO3 | AC131971.1 | 0.485711 | 5.09E-33 |
| NAF1 | AC037198.2 | 0.485734 | 5.05E-33 |
| CTSB | AP002954.1 | 0.485745 | 5.03E-33 |
| CFLAR | AC037487.2 | 0.485764 | 5.00E-33 |
| MAPK8 | AC005632.2 | 0.485818 | 4.91E-33 |
| EIF2AK2 | FLNB-AS1 | 0.485832 | 4.88E-33 |
| CFLAR | AP001429.1 | 0.485864 | 4.83E-33 |
| NAF1 | AC027097.2 | 0.485891 | 4.79E-33 |
| ATG12 | SP2-AS1 | 0.485927 | 4.73E-33 |
| RB1 | AC073651.1 | 0.485984 | 4.64E-33 |
| NAF1 | STARD4-AS1 | 0.485991 | 4.63E-33 |
| EIF2AK2 | SP2-AS1 | 0.486016 | 4.59E-33 |
| GOPC | AC063965.1 | 0.48603 | 4.56E-33 |
| TSC2 | AC093752.3 | 0.486039 | 4.55E-33 |
| PTEN | AF131215.6 | 0.486053 | 4.53E-33 |
| MAPK8 | AC138956.1 | 0.486055 | 4.53E-33 |
| RB1CC1 | AL021578.1 | 0.486066 | 4.51E-33 |
| KLHL24 | RRN3P2 | 0.48607 | 4.50E-33 |
| CAPN10 | AL354836.1 | 0.486075 | 4.50E-33 |
| PIK3C3 | AL157786.1 | 0.486104 | 4.45E-33 |
| CFLAR | AC141002.1 | 0.486121 | 4.42E-33 |
| FOXO1 | AC015849.3 | 0.486145 | 4.39E-33 |
| WDFY3 | CTBP1-AS | 0.486156 | 4.37E-33 |
| ATG12 | AC107027.3 | 0.486186 | 4.33E-33 |
| MAPK8 | AC115989.1 | 0.486211 | 4.29E-33 |
| TSC1 | AC000123.1 | 0.486251 | 4.23E-33 |
| EIF2AK2 | AC010201.2 | 0.486291 | 4.18E-33 |
| RB1 | AC090739.1 | 0.486298 | 4.17E-33 |
| GOPC | AC005104.1 | 0.486302 | 4.16E-33 |
| PIK3C3 | ARHGEF38-IT1 | 0.48637 | 4.06E-33 |
| CAPN10 | DM1-AS | 0.486389 | 4.04E-33 |
| SPNS1 | AC020558.2 | 0.486408 | 4.01E-33 |
| FOXO3 | AP000786.1 | 0.48645 | 3.95E-33 |
| NAF1 | AL592148.3 | 0.486459 | 3.94E-33 |
| FOXO1 | AC008669.1 | 0.486504 | 3.88E-33 |
| GOPC | PSPC1-AS2 | 0.486514 | 3.87E-33 |
| ATG12 | AL132780.1 | 0.486588 | 3.77E-33 |
| NAF1 | AC007546.1 | 0.486598 | 3.76E-33 |
| CFLAR | AL353804.2 | 0.486624 | 3.73E-33 |
| CFLAR | LAMC1-AS1 | 0.486644 | 3.70E-33 |
| RB1CC1 | AL359715.3 | 0.486646 | 3.70E-33 |
| BIRC6 | HMGN3-AS1 | 0.486663 | 3.68E-33 |
| RPS6KB1 | AC097376.2 | 0.486669 | 3.67E-33 |
| RB1 | AC090181.2 | 0.486683 | 3.65E-33 |
| FOXO1 | SCARNA9 | 0.486688 | 3.65E-33 |
| EIF2AK2 | AC074032.1 | 0.486696 | 3.64E-33 |
| WDFY3 | AC009237.15 | 0.486704 | 3.63E-33 |
| FOXO3 | TRAF3IP2-AS1 | 0.486717 | 3.61E-33 |
| KLHL24 | AL157932.1 | 0.486762 | 3.55E-33 |
| ATG2B | MIR600HG | 0.486775 | 3.54E-33 |
| NAF1 | AC139887.4 | 0.486794 | 3.52E-33 |
| CCR2 | TRBV11-2 | 0.486806 | 3.50E-33 |
| RPS6KB1 | GAS5-AS1 | 0.486816 | 3.49E-33 |
| GOPC | AL353804.1 | 0.486872 | 3.42E-33 |
| NAF1 | AC002128.2 | 0.486941 | 3.34E-33 |
| RB1CC1 | AC078778.1 | 0.486955 | 3.33E-33 |
| MAPK8 | AP000692.1 | 0.48697 | 3.31E-33 |
| KLHL24 | AP001160.4 | 0.486972 | 3.31E-33 |
| TSC1 | Z68871.1 | 0.486977 | 3.30E-33 |
| GOPC | AC090181.2 | 0.486995 | 3.28E-33 |
| WDFY3 | AC016957.2 | 0.487011 | 3.26E-33 |
| GOPC | AP005131.7 | 0.487031 | 3.24E-33 |
| ATG16L2 | PSMA3-AS1 | 0.487057 | 3.21E-33 |
| NAF1 | RFX3-AS1 | 0.487085 | 3.18E-33 |
| NAF1 | AC018682.1 | 0.487108 | 3.16E-33 |
| RB1 | AC068790.3 | 0.487114 | 3.15E-33 |
| CFLAR | AC093227.1 | 0.48712 | 3.15E-33 |
| RB1 | AL162724.1 | 0.487135 | 3.13E-33 |
| LAMP1 | ZEB1-AS1 | 0.487178 | 3.08E-33 |
| EIF2AK2 | AC127024.5 | 0.487193 | 3.07E-33 |
| GOPC | AC012557.1 | 0.48721 | 3.05E-33 |
| FOXO1 | AF117829.1 | 0.487216 | 3.04E-33 |
| CAPNS1 | AC020910.4 | 0.487219 | 3.04E-33 |
| RB1 | SCARNA9 | 0.487235 | 3.02E-33 |
| CAPN10 | AL928654.2 | 0.487245 | 3.01E-33 |
| NAF1 | AC024075.3 | 0.487273 | 2.98E-33 |
| PIK3C3 | AL031775.2 | 0.487285 | 2.97E-33 |
| RB1 | Z83843.1 | 0.487292 | 2.97E-33 |
| PTEN | LINC01004 | 0.487344 | 2.91E-33 |
| ATG12 | AC002128.1 | 0.48737 | 2.89E-33 |
| RB1 | OIP5-AS1 | 0.487372 | 2.89E-33 |
| RB1 | AC016590.2 | 0.487384 | 2.87E-33 |
| TSC1 | MIR3936HG | 0.487424 | 2.83E-33 |
| PIK3C3 | ABALON | 0.487445 | 2.81E-33 |
| BIRC6 | THUMPD3-AS1 | 0.487451 | 2.81E-33 |
| GOPC | ADNP-AS1 | 0.487476 | 2.78E-33 |
| RPS6KB1 | AL162724.2 | 0.48748 | 2.78E-33 |
| KLHL24 | AC018809.2 | 0.487483 | 2.78E-33 |
| PTEN | AC048341.2 | 0.487495 | 2.77E-33 |
| RB1 | AL133342.1 | 0.487496 | 2.76E-33 |
| GOPC | AC002553.2 | 0.4875 | 2.76E-33 |
| EIF2AK2 | PCBP1-AS1 | 0.487568 | 2.70E-33 |
| MAPK8 | AC007849.1 | 0.487576 | 2.69E-33 |
| PIK3C3 | AC092794.1 | 0.487576 | 2.69E-33 |
| KLHL24 | AC063965.1 | 0.487595 | 2.67E-33 |
| WDFY3 | MIR155HG | 0.487638 | 2.63E-33 |
| RPS6KB1 | SDCBP2-AS1 | 0.487711 | 2.57E-33 |
| MAPK8 | AL136531.1 | 0.48776 | 2.53E-33 |
| RB1 | CFLAR-AS1 | 0.487763 | 2.52E-33 |
| NAF1 | AL359915.2 | 0.487775 | 2.51E-33 |
| ATG7 | AC010186.3 | 0.487783 | 2.51E-33 |
| TSC1 | AC025917.1 | 0.487792 | 2.50E-33 |
| BIRC6 | LINC01655 | 0.487801 | 2.49E-33 |
| EIF2AK2 | AL021707.7 | 0.487805 | 2.49E-33 |
| RPS6KB1 | AC018752.1 | 0.48783 | 2.47E-33 |
| CFLAR | AC026470.2 | 0.487838 | 2.46E-33 |
| MAPK8 | AL513327.1 | 0.487859 | 2.44E-33 |
| BIRC6 | AP001528.2 | 0.487867 | 2.43E-33 |
| MAPK8 | ARHGEF38-IT1 | 0.487942 | 2.37E-33 |
| ATG16L2 | ZNF436-AS1 | 0.487974 | 2.35E-33 |
| KLHL24 | AL136531.1 | 0.488002 | 2.32E-33 |
| KLHL24 | MAST4-AS1 | 0.488006 | 2.32E-33 |
| ATG12 | AC091185.1 | 0.488025 | 2.31E-33 |
| PTEN | ARHGAP31-AS1 | 0.488033 | 2.30E-33 |
| ATG16L2 | AL136304.1 | 0.488042 | 2.29E-33 |
| TSC1 | AL136295.6 | 0.488082 | 2.26E-33 |
| MAPK8 | AC004466.3 | 0.488098 | 2.25E-33 |
| FOXO3 | AL109614.1 | 0.48813 | 2.22E-33 |
| FOXO1 | AC027277.2 | 0.488256 | 2.13E-33 |
| ATG3 | NCK1-DT | 0.488292 | 2.10E-33 |
| KLHL24 | AC107068.1 | 0.488317 | 2.09E-33 |
| TSC2 | AC009065.4 | 0.488324 | 2.08E-33 |
| NAF1 | AC011477.2 | 0.488339 | 2.07E-33 |
| GOPC | AL163051.2 | 0.488358 | 2.06E-33 |
| RPS6KB1 | AL022067.1 | 0.488378 | 2.04E-33 |
| EIF2AK2 | AC004884.2 | 0.488403 | 2.02E-33 |
| GOPC | AC130895.1 | 0.488407 | 2.02E-33 |
| ATG7 | AF178030.1 | 0.488414 | 2.02E-33 |
| GOPC | AP001381.1 | 0.488416 | 2.02E-33 |
| KLHL24 | LINC02035 | 0.488441 | 2.00E-33 |
| KLHL24 | AC004477.3 | 0.488443 | 2.00E-33 |
| MAPK8 | ADAMTSL4-AS1 | 0.488459 | 1.99E-33 |
| TSC1 | SMC5-AS1 | 0.488515 | 1.95E-33 |
| FOXO1 | PWAR6 | 0.488545 | 1.93E-33 |
| KLHL24 | AC002553.1 | 0.488594 | 1.90E-33 |
| KLHL24 | AP001178.2 | 0.488632 | 1.87E-33 |
| EIF2AK2 | AL132780.1 | 0.488651 | 1.86E-33 |
| ATG12 | AL109761.1 | 0.488666 | 1.85E-33 |
| KLHL24 | ATP1A1-AS1 | 0.488678 | 1.84E-33 |
| KLHL24 | AC078778.1 | 0.488687 | 1.84E-33 |
| TSC1 | AC099343.2 | 0.488704 | 1.83E-33 |
| NAF1 | AC073569.2 | 0.488706 | 1.82E-33 |
| CFLAR | AL157402.2 | 0.488728 | 1.81E-33 |
| RB1CC1 | AC016394.1 | 0.488732 | 1.81E-33 |
| PRKAR1A | NORAD | 0.488762 | 1.79E-33 |
| MAPK8 | AL353804.2 | 0.488762 | 1.79E-33 |
| PIK3R4 | Z68871.1 | 0.488775 | 1.78E-33 |
| GOPC | AC093388.1 | 0.488821 | 1.75E-33 |
| ATG4B | SSSCA1-AS1 | 0.488827 | 1.75E-33 |
| CFLAR | MIR3936HG | 0.488829 | 1.75E-33 |
| FOXO1 | AC005479.2 | 0.488885 | 1.72E-33 |
| RPS6KB1 | AC087286.4 | 0.488897 | 1.71E-33 |
| GOPC | GMDS-DT | 0.488898 | 1.71E-33 |
| RPS6KB1 | AC068790.3 | 0.488934 | 1.69E-33 |
| RPS6KB1 | CD44-AS1 | 0.488949 | 1.68E-33 |
| RPS6KB1 | LINC00641 | 0.488961 | 1.67E-33 |
| GOPC | AL365277.1 | 0.488974 | 1.66E-33 |
| CFLAR | AC091185.1 | 0.488978 | 1.66E-33 |
| ATG7 | AC090948.2 | 0.488991 | 1.65E-33 |
| CFLAR | AL133371.2 | 0.489075 | 1.61E-33 |
| FOXO1 | AC016590.2 | 0.489112 | 1.59E-33 |
| PIK3C3 | AC008966.2 | 0.489141 | 1.57E-33 |
| RB1 | AC012557.1 | 0.489179 | 1.55E-33 |
| ATG4B | AP003419.3 | 0.489189 | 1.54E-33 |
| ATG2B | LINC00426 | 0.489219 | 1.53E-33 |
| NAF1 | AC016394.1 | 0.489231 | 1.52E-33 |
| FOXO3 | AC096921.2 | 0.489311 | 1.48E-33 |
| ULK3 | STAG3L5P-PVRIG2P-PILRB | 0.489318 | 1.48E-33 |
| KLHL24 | AL513008.1 | 0.489343 | 1.47E-33 |
| GOPC | AC127024.4 | 0.489373 | 1.45E-33 |
| CFLAR | AC127024.5 | 0.48938 | 1.45E-33 |
| WDFY3 | EBLN3P | 0.489394 | 1.44E-33 |
| TSC1 | AL138921.2 | 0.489394 | 1.44E-33 |
| ATG2B | LINC01290 | 0.489407 | 1.43E-33 |
| MAPK8 | AC005261.1 | 0.489433 | 1.42E-33 |
| KLHL24 | AL021578.1 | 0.489466 | 1.40E-33 |
| KLHL24 | PSMA3-AS1 | 0.489515 | 1.38E-33 |
| GOPC | AC018926.3 | 0.489532 | 1.37E-33 |
| CFLAR | AL162724.1 | 0.489564 | 1.36E-33 |
| EIF2AK2 | AC026470.2 | 0.489595 | 1.34E-33 |
| CAPN10 | AC004918.1 | 0.489607 | 1.34E-33 |
| TSC1 | AP000766.1 | 0.489609 | 1.34E-33 |
| GOPC | N4BP2L2-IT2 | 0.489624 | 1.33E-33 |
| CAPN10 | AL691482.3 | 0.489626 | 1.33E-33 |
| MAPK8 | DNM3OS | 0.489697 | 1.30E-33 |
| RPS6KB1 | AL391834.1 | 0.489756 | 1.27E-33 |
| ATG2B | GK-AS1 | 0.489759 | 1.27E-33 |
| FOXO1 | AC096586.2 | 0.489794 | 1.25E-33 |
| KLHL24 | AC022211.1 | 0.489872 | 1.22E-33 |
| TSC1 | LINC00893 | 0.489904 | 1.21E-33 |
| WDFY3 | AC005034.3 | 0.48991 | 1.20E-33 |
| ATG4B | AC245140.2 | 0.489911 | 1.20E-33 |
| CFLAR | AC083862.2 | 0.489933 | 1.19E-33 |
| FOXO3 | AC016831.4 | 0.489937 | 1.19E-33 |
| RB1 | AC007216.3 | 0.490013 | 1.16E-33 |
| CFLAR | AL683813.1 | 0.490034 | 1.15E-33 |
| FOXO3 | AC114760.2 | 0.490069 | 1.14E-33 |
| TSC1 | GAS8-AS1 | 0.490093 | 1.13E-33 |
| RB1CC1 | AC253576.2 | 0.490102 | 1.13E-33 |
| CFLAR | AC090198.1 | 0.490174 | 1.10E-33 |
| FOXO1 | AC000123.1 | 0.49018 | 1.10E-33 |
| ATG12 | AC048341.2 | 0.490227 | 1.08E-33 |
| KLHL24 | AC141002.1 | 0.490335 | 1.04E-33 |
| ATG7 | LIMS1-AS1 | 0.490356 | 1.03E-33 |
| MAPK8 | AC018638.7 | 0.490367 | 1.03E-33 |
| KLHL24 | AC067852.3 | 0.490371 | 1.03E-33 |
| MAPK8 | AC012181.1 | 0.490386 | 1.02E-33 |
| EIF2AK2 | AC124312.5 | 0.490389 | 1.02E-33 |
| EIF2AK2 | AC073569.2 | 0.490394 | 1.02E-33 |
| NAF1 | AL157402.2 | 0.490424 | 1.01E-33 |
| RB1CC1 | AC000123.1 | 0.490427 | 1.01E-33 |
| KLHL24 | AP000873.2 | 0.490431 | 1.01E-33 |
| TSC1 | AC005726.3 | 0.490435 | 1.00E-33 |
| PIK3C3 | AC025287.3 | 0.490436 | 1.00E-33 |
| KLHL24 | AC124312.5 | 0.490467 | 9.93E-34 |
| PIK3C3 | AGAP1-IT1 | 0.490473 | 9.91E-34 |
| WDFY3 | NR2F1-AS1 | 0.490473 | 9.91E-34 |
| NAF1 | LINC01355 | 0.49048 | 9.89E-34 |
| ATG7 | AC037487.2 | 0.490533 | 9.71E-34 |
| RPS6KB1 | AC005920.2 | 0.490536 | 9.70E-34 |
| BIRC6 | AC062037.2 | 0.490543 | 9.68E-34 |
| TSC1 | AC139887.2 | 0.49057 | 9.58E-34 |
| NAF1 | A2M-AS1 | 0.49057 | 9.58E-34 |
| ATG12 | AC141002.1 | 0.490585 | 9.53E-34 |
| TSC1 | AC016590.2 | 0.490588 | 9.53E-34 |
| MAPK8 | SAP30L-AS1 | 0.490602 | 9.48E-34 |
| GOPC | AC096741.1 | 0.490603 | 9.47E-34 |
| CFLAR | AC139795.2 | 0.490631 | 9.39E-34 |
| MAPK8 | AL133342.1 | 0.49064 | 9.35E-34 |
| KLHL24 | AC009041.4 | 0.490645 | 9.34E-34 |
| MAPK8 | AL121839.2 | 0.490684 | 9.21E-34 |
| EIF2AK2 | AC024933.1 | 0.4907 | 9.16E-34 |
| ATG2B | AC090589.3 | 0.490726 | 9.08E-34 |
| RB1CC1 | AC092279.1 | 0.490741 | 9.03E-34 |
| RPS6KB1 | PSPC1-AS2 | 0.490753 | 9.00E-34 |
| TSC2 | AL022328.1 | 0.490796 | 8.86E-34 |
| MAPK8 | AL138921.2 | 0.490837 | 8.74E-34 |
| RB1CC1 | RAP2C-AS1 | 0.490838 | 8.73E-34 |
| EIF2AK2 | ERVK13-1 | 0.490857 | 8.68E-34 |
| MAPK8 | A2M-AS1 | 0.490865 | 8.65E-34 |
| NAF1 | AL157394.1 | 0.490877 | 8.62E-34 |
| FOXO3 | AC004918.3 | 0.490914 | 8.51E-34 |
| MAPK8 | AC139887.4 | 0.490916 | 8.50E-34 |
| RPS6KB1 | AC011939.2 | 0.490918 | 8.50E-34 |
| NLRC4 | AC138207.5 | 0.490962 | 8.37E-34 |
| TSC1 | AF129075.1 | 0.490972 | 8.34E-34 |
| ATG16L2 | AL139289.1 | 0.490972 | 8.34E-34 |
| RB1 | N4BP2L2-IT2 | 0.490976 | 8.33E-34 |
| RB1CC1 | AL359962.2 | 0.490986 | 8.30E-34 |
| FOXO1 | AL031775.2 | 0.491001 | 8.25E-34 |
| EIF2AK2 | NDUFV2-AS1 | 0.491013 | 8.22E-34 |
| IL24 | LINC01215 | 0.491019 | 8.20E-34 |
| RB1 | AP001381.1 | 0.491051 | 8.11E-34 |
| ATG2B | AC232271.1 | 0.491057 | 8.10E-34 |
| FOXO3 | AP003486.1 | 0.491146 | 7.85E-34 |
| RB1CC1 | AC005021.1 | 0.491194 | 7.72E-34 |
| PTEN | LINC01184 | 0.491196 | 7.72E-34 |
| TSC1 | AC011481.1 | 0.491198 | 7.71E-34 |
| ATG2B | PAXIP1-AS2 | 0.491238 | 7.60E-34 |
| PTEN | AC068792.1 | 0.491267 | 7.53E-34 |
| RB1 | GMDS-DT | 0.491312 | 7.41E-34 |
| GOPC | AC124283.3 | 0.491326 | 7.38E-34 |
| EIF2AK2 | FMR1-IT1 | 0.491339 | 7.34E-34 |
| RB1 | CR936218.1 | 0.491405 | 7.17E-34 |
| CFLAR | SDCBP2-AS1 | 0.491453 | 7.06E-34 |
| PIK3C3 | AC012467.2 | 0.491459 | 7.04E-34 |
| ATG16L2 | AC027020.2 | 0.49146 | 7.04E-34 |
| NAF1 | AL645568.1 | 0.491476 | 7.00E-34 |
| PTEN | AC007991.2 | 0.491482 | 6.99E-34 |
| RPS6KB1 | AL157838.1 | 0.491486 | 6.98E-34 |
| RB1 | AC058791.1 | 0.491512 | 6.91E-34 |
| GOPC | AC107068.1 | 0.491524 | 6.88E-34 |
| TSC1 | AC053513.1 | 0.49153 | 6.87E-34 |
| MYC | AC006329.1 | 0.491535 | 6.86E-34 |
| CFLAR | AC018690.1 | 0.49155 | 6.82E-34 |
| EIF2AK2 | AC005632.2 | 0.491565 | 6.79E-34 |
| ATG12 | MCCC1-AS1 | 0.491582 | 6.75E-34 |
| MAPK8 | AC098851.1 | 0.491583 | 6.74E-34 |
| ATG12 | FLNB-AS1 | 0.491607 | 6.69E-34 |
| BIRC6 | AC091057.1 | 0.491616 | 6.67E-34 |
| NAF1 | AC037198.1 | 0.491652 | 6.58E-34 |
| TSC1 | TBILA | 0.491705 | 6.46E-34 |
| KLHL24 | AL606834.2 | 0.491716 | 6.44E-34 |
| GOPC | DLEU2 | 0.491729 | 6.41E-34 |
| BIRC6 | AL132657.1 | 0.491733 | 6.40E-34 |
| CFLAR | AC062037.2 | 0.491762 | 6.34E-34 |
| ATG2B | IGBP1-AS1 | 0.491774 | 6.31E-34 |
| MAPK8 | AC096992.2 | 0.491774 | 6.31E-34 |
| RAB5A | AC096921.2 | 0.491776 | 6.31E-34 |
| GOPC | RAP2C-AS1 | 0.491827 | 6.20E-34 |
| RB1CC1 | AC127024.5 | 0.491841 | 6.17E-34 |
| MAPK8 | AL354733.3 | 0.491866 | 6.11E-34 |
| GOPC | AC096586.2 | 0.491869 | 6.11E-34 |
| PIK3C3 | AC048344.4 | 0.491876 | 6.09E-34 |
| EIF2AK2 | AC006270.1 | 0.49189 | 6.06E-34 |
| RPS6KB1 | AL158166.1 | 0.491894 | 6.05E-34 |
| CTSB | AC090559.1 | 0.491909 | 6.02E-34 |
| RB1CC1 | DLEU1 | 0.491922 | 6.00E-34 |
| ATG16L2 | AC080038.1 | 0.491924 | 5.99E-34 |
| GOPC | AC138393.3 | 0.491932 | 5.97E-34 |
| MAPK8 | FMR1-IT1 | 0.491934 | 5.97E-34 |
| ATG12 | AC018690.1 | 0.491941 | 5.96E-34 |
| EIF2AK2 | AL645568.1 | 0.491941 | 5.95E-34 |
| EIF2AK2 | AL157871.2 | 0.491947 | 5.94E-34 |
| MAP2K7 | AC069281.2 | 0.491955 | 5.93E-34 |
| KLHL24 | LINC00894 | 0.491957 | 5.92E-34 |
| RB1 | AC007038.1 | 0.491979 | 5.88E-34 |
| RPS6KB1 | AC099343.2 | 0.491991 | 5.85E-34 |
| MAPK8 | AC010168.2 | 0.492053 | 5.73E-34 |
| TSC1 | AL139041.1 | 0.492072 | 5.69E-34 |
| PIK3C3 | MIATNB | 0.492086 | 5.66E-34 |
| RPS6KB1 | AC110792.3 | 0.492096 | 5.64E-34 |
| ATG12 | AC022306.2 | 0.492096 | 5.64E-34 |
| CFLAR | PCBP1-AS1 | 0.492112 | 5.61E-34 |
| GOPC | AC008115.3 | 0.492126 | 5.58E-34 |
| WDFY3 | AL157871.2 | 0.492131 | 5.57E-34 |
| WDFY3 | AC027117.1 | 0.492146 | 5.54E-34 |
| RPS6KB1 | AP000873.2 | 0.492169 | 5.50E-34 |
| RPS6KB1 | AC125257.1 | 0.49219 | 5.46E-34 |
| TSC2 | AL365330.1 | 0.492201 | 5.44E-34 |
| IFNG | C5orf56 | 0.492207 | 5.43E-34 |
| ATG12 | SNHG14 | 0.492227 | 5.39E-34 |
| PIK3C3 | AL353804.2 | 0.49225 | 5.35E-34 |
| ATG2B | AL159169.2 | 0.492289 | 5.28E-34 |
| KLHL24 | AC124319.2 | 0.492302 | 5.25E-34 |
| TSC1 | AP000873.2 | 0.492333 | 5.20E-34 |
| TSC1 | AC002553.2 | 0.49235 | 5.16E-34 |
| RB1CC1 | MBNL1-AS1 | 0.492372 | 5.12E-34 |
| EIF2AK2 | AC009032.1 | 0.492376 | 5.12E-34 |
| RPS6KB1 | AC021851.1 | 0.49247 | 4.95E-34 |
| MAPK8 | AC113139.1 | 0.492504 | 4.90E-34 |
| PIK3C3 | AC007552.2 | 0.492507 | 4.89E-34 |
| CFLAR | AC009120.2 | 0.492514 | 4.88E-34 |
| FOXO1 | AP000866.6 | 0.492538 | 4.84E-34 |
| MAPK8 | CD44-AS1 | 0.49254 | 4.83E-34 |
| ATG2B | AC093726.2 | 0.492591 | 4.75E-34 |
| CFLAR | AC023449.2 | 0.492605 | 4.72E-34 |
| CFLAR | AC005479.1 | 0.492629 | 4.69E-34 |
| RB1CC1 | AL049840.2 | 0.492694 | 4.58E-34 |
| RB1CC1 | UBR5-AS1 | 0.492714 | 4.55E-34 |
| ATG12 | Z98884.2 | 0.492715 | 4.55E-34 |
| ATG7 | AC008115.3 | 0.492744 | 4.50E-34 |
| ATG7 | HIF1A-AS2 | 0.492762 | 4.47E-34 |
| TSC1 | AC093227.1 | 0.49285 | 4.34E-34 |
| RB1CC1 | AC090948.3 | 0.492854 | 4.33E-34 |
| TSC1 | AC093110.1 | 0.492867 | 4.31E-34 |
| ATG4B | AC073896.4 | 0.492895 | 4.27E-34 |
| KLHL24 | AC005632.2 | 0.492902 | 4.26E-34 |
| MAPK8 | AL133330.1 | 0.49291 | 4.25E-34 |
| CFLAR | AC037198.2 | 0.492911 | 4.25E-34 |
| ATG2B | RUSC1-AS1 | 0.492933 | 4.21E-34 |
| CFLAR | LINC00641 | 0.492942 | 4.20E-34 |
| RB1CC1 | ALMS1-IT1 | 0.492977 | 4.15E-34 |
| RB1 | AC025917.1 | 0.493 | 4.12E-34 |
| CAPN10 | AC139530.1 | 0.49302 | 4.09E-34 |
| ATG7 | ADAMTSL4-AS1 | 0.493058 | 4.03E-34 |
| BIRC6 | AC006213.4 | 0.493138 | 3.92E-34 |
| GOPC | AC100830.2 | 0.493165 | 3.89E-34 |
| GOPC | AL359915.2 | 0.493165 | 3.89E-34 |
| RB1 | AC053513.1 | 0.493181 | 3.87E-34 |
| FOXO3 | RAP2C-AS1 | 0.493182 | 3.86E-34 |
| FOXO1 | AC020915.2 | 0.493187 | 3.86E-34 |
| RB1CC1 | AC027097.2 | 0.493242 | 3.78E-34 |
| ATG16L2 | AL450384.2 | 0.493262 | 3.76E-34 |
| ATG12 | AC096921.2 | 0.493279 | 3.74E-34 |
| KLHL24 | AC245884.8 | 0.49328 | 3.73E-34 |
| TSC1 | C5orf56 | 0.49329 | 3.72E-34 |
| TSC1 | AL122010.1 | 0.493294 | 3.71E-34 |
| MAPK8 | AP001458.1 | 0.493377 | 3.61E-34 |
| FOXO1 | ERVK13-1 | 0.493401 | 3.58E-34 |
| BIRC6 | HM13-IT1 | 0.493416 | 3.56E-34 |
| FOXO3 | AC087286.4 | 0.493426 | 3.55E-34 |
| KLHL24 | AP001486.2 | 0.49343 | 3.54E-34 |
| EIF2AK2 | AL354733.3 | 0.493445 | 3.52E-34 |
| IFNG | AC004585.1 | 0.493511 | 3.44E-34 |
| NAF1 | AC083843.2 | 0.493523 | 3.43E-34 |
| RB1CC1 | AC024933.1 | 0.493667 | 3.26E-34 |
| EIF2AK2 | AC004477.3 | 0.493727 | 3.19E-34 |
| ATG12 | AC008537.2 | 0.493759 | 3.16E-34 |
| CFLAR | AC096741.1 | 0.493775 | 3.14E-34 |
| ATG12 | NARF-IT1 | 0.493795 | 3.12E-34 |
| NAF1 | LINC00852 | 0.493843 | 3.07E-34 |
| NAF1 | AL162724.2 | 0.49389 | 3.02E-34 |
| MAPK8 | OCIAD1-AS1 | 0.493896 | 3.01E-34 |
| BIRC6 | AC009120.2 | 0.493929 | 2.98E-34 |
| PIK3C3 | AL355488.1 | 0.493932 | 2.97E-34 |
| RPS6KB1 | AP001429.1 | 0.493942 | 2.96E-34 |
| EIF2AK2 | AC107068.1 | 0.493956 | 2.95E-34 |
| GOPC | LAMC1-AS1 | 0.493957 | 2.95E-34 |
| TSC1 | AC098851.1 | 0.493974 | 2.93E-34 |
| CFLAR | GAS5-AS1 | 0.494035 | 2.87E-34 |
| NAF1 | AC006017.1 | 0.494045 | 2.86E-34 |
| RPS6KB1 | AC005838.2 | 0.494083 | 2.82E-34 |
| RPS6KB1 | AC002064.2 | 0.494093 | 2.81E-34 |
| ATG12 | AC011468.5 | 0.494097 | 2.81E-34 |
| MAPK8 | AL606834.1 | 0.494098 | 2.80E-34 |
| CFLAR | AP006621.2 | 0.494126 | 2.78E-34 |
| RPS6KB1 | AL117381.1 | 0.494192 | 2.71E-34 |
| RPS6KB1 | FTX | 0.494206 | 2.70E-34 |
| MAPK8 | ARHGAP31-AS1 | 0.494217 | 2.69E-34 |
| FOXO1 | LINC00861 | 0.494217 | 2.69E-34 |
| ATG12 | AP001486.2 | 0.494319 | 2.60E-34 |
| FOXO3 | AL359076.1 | 0.494344 | 2.57E-34 |
| GOPC | AC097376.2 | 0.494394 | 2.53E-34 |
| RB1CC1 | AL137782.1 | 0.494413 | 2.51E-34 |
| RB1CC1 | AC012467.1 | 0.494421 | 2.50E-34 |
| FOXO1 | AL359697.1 | 0.494443 | 2.48E-34 |
| FOXO1 | AC024075.3 | 0.494513 | 2.42E-34 |
| EIF2AK2 | AC009948.1 | 0.494572 | 2.37E-34 |
| MAPK8 | AC009054.2 | 0.494594 | 2.36E-34 |
| GOPC | AC114760.2 | 0.494607 | 2.35E-34 |
| PIK3C3 | AC015849.3 | 0.494611 | 2.34E-34 |
| NAF1 | AC026470.2 | 0.494623 | 2.33E-34 |
| CFLAR | AL360219.1 | 0.49464 | 2.32E-34 |
| RPS6KB1 | AL049552.1 | 0.494653 | 2.31E-34 |
| CFLAR | AC097376.2 | 0.494699 | 2.27E-34 |
| ATG4B | AL139349.1 | 0.4947 | 2.27E-34 |
| RPS6KB1 | AC015911.3 | 0.494737 | 2.24E-34 |
| ATG12 | AC096992.2 | 0.494741 | 2.24E-34 |
| CFLAR | AC007684.1 | 0.494776 | 2.21E-34 |
| EIF2AK2 | AL158166.2 | 0.494782 | 2.21E-34 |
| CFLAR | AP001469.2 | 0.494792 | 2.20E-34 |
| TSC2 | AC106782.6 | 0.494799 | 2.19E-34 |
| MAPK8 | AC092279.1 | 0.494829 | 2.17E-34 |
| WDFY3 | AC020571.1 | 0.494829 | 2.17E-34 |
| ATG16L2 | AC093726.2 | 0.494832 | 2.17E-34 |
| GOPC | AC005034.5 | 0.494882 | 2.13E-34 |
| RB1 | AC087286.1 | 0.494894 | 2.12E-34 |
| RPS6KB1 | Z83843.1 | 0.494919 | 2.10E-34 |
| WDFY3 | AC025857.2 | 0.494921 | 2.10E-34 |
| RB1CC1 | AC066613.1 | 0.494943 | 2.08E-34 |
| WDFY3 | AC130456.3 | 0.494946 | 2.08E-34 |
| RPS6KB1 | AC242426.2 | 0.494954 | 2.08E-34 |
| ATG2B | AF131215.5 | 0.494969 | 2.07E-34 |
| EIF2AK2 | AP001469.2 | 0.494971 | 2.06E-34 |
| KLHL24 | RAP2C-AS1 | 0.494981 | 2.06E-34 |
| NAF1 | AC105389.2 | 0.494997 | 2.05E-34 |
| TSC1 | AL135999.1 | 0.495034 | 2.02E-34 |
| PRKAR1A | OIP5-AS1 | 0.495083 | 1.98E-34 |
| PIK3C3 | LINC00852 | 0.495092 | 1.98E-34 |
| MAPK8 | AC097641.2 | 0.495099 | 1.97E-34 |
| ATG7 | C5orf56 | 0.495104 | 1.97E-34 |
| RPS6KB1 | AC007546.1 | 0.49511 | 1.97E-34 |
| RB1CC1 | AC018690.1 | 0.49511 | 1.97E-34 |
| PIK3C3 | AP000873.2 | 0.495118 | 1.96E-34 |
| FOXO3 | AC108449.2 | 0.495123 | 1.96E-34 |
| KLHL24 | SCAANT1 | 0.495144 | 1.94E-34 |
| GOPC | AP001033.2 | 0.495175 | 1.92E-34 |
| PIK3C3 | AL137782.1 | 0.495246 | 1.87E-34 |
| CFLAR | AP001033.2 | 0.495254 | 1.87E-34 |
| FOXO1 | AC005034.5 | 0.495263 | 1.86E-34 |
| RB1CC1 | AC015849.3 | 0.495277 | 1.85E-34 |
| PIK3C3 | AC005540.1 | 0.495299 | 1.84E-34 |
| ATG12 | AC015871.3 | 0.49531 | 1.83E-34 |
| PIK3C3 | AC007406.5 | 0.495321 | 1.83E-34 |
| PTEN | SCAANT1 | 0.495344 | 1.81E-34 |
| TSC1 | LINC01534 | 0.495364 | 1.80E-34 |
| FOXO1 | AL122035.1 | 0.495395 | 1.78E-34 |
| PIK3C3 | AC010201.2 | 0.495468 | 1.73E-34 |
| WDFY3 | AC113139.1 | 0.495495 | 1.72E-34 |
| GOPC | ALG13-AS1 | 0.495526 | 1.70E-34 |
| TSC1 | AC025287.3 | 0.495528 | 1.70E-34 |
| PTEN | RAB30-AS1 | 0.495533 | 1.69E-34 |
| RB1 | AC005856.1 | 0.495544 | 1.69E-34 |
| GOPC | AC018752.1 | 0.495557 | 1.68E-34 |
| GOPC | PAXBP1-AS1 | 0.495558 | 1.68E-34 |
| ATG2B | AC005253.1 | 0.495585 | 1.66E-34 |
| FOXO1 | AC009318.3 | 0.495588 | 1.66E-34 |
| NAF1 | AC007319.1 | 0.495589 | 1.66E-34 |
| ATG2B | AC007849.1 | 0.495591 | 1.66E-34 |
| RPS6KB1 | ANKRD44-IT1 | 0.495605 | 1.65E-34 |
| NAF1 | AP002907.1 | 0.495637 | 1.63E-34 |
| MAPK8 | STARD4-AS1 | 0.495637 | 1.63E-34 |
| RPS6KB1 | AC020915.2 | 0.495666 | 1.62E-34 |
| MAPK8 | MIR181A2HG | 0.495672 | 1.61E-34 |
| PIK3C3 | AC004908.2 | 0.495709 | 1.59E-34 |
| ATG12 | LINC02352 | 0.495713 | 1.59E-34 |
| EIF2AK2 | AC027097.2 | 0.495726 | 1.58E-34 |
| NAF1 | ARHGEF38-IT1 | 0.495758 | 1.57E-34 |
| RB1CC1 | SAP30L-AS1 | 0.49576 | 1.56E-34 |
| EIF2AK2 | TRAF3IP2-AS1 | 0.495765 | 1.56E-34 |
| NAF1 | AC004908.2 | 0.495765 | 1.56E-34 |
| GOPC | AC090739.1 | 0.495802 | 1.54E-34 |
| RB1 | AC087284.1 | 0.495829 | 1.53E-34 |
| RB1 | AC100830.2 | 0.495835 | 1.52E-34 |
| PIK3C3 | AC002550.2 | 0.495873 | 1.50E-34 |
| CFLAR | AL359962.2 | 0.495877 | 1.50E-34 |
| RPS6KB1 | AC008770.3 | 0.495886 | 1.50E-34 |
| FOXO3 | AC138932.5 | 0.495887 | 1.50E-34 |
| ATG12 | AC048341.1 | 0.495909 | 1.48E-34 |
| GOPC | AC024075.1 | 0.495917 | 1.48E-34 |
| NAF1 | AC008966.2 | 0.49592 | 1.48E-34 |
| GABARAPL2 | OSER1-DT | 0.495925 | 1.48E-34 |
| GOPC | AL117381.1 | 0.495938 | 1.47E-34 |
| FOXO1 | AP001160.4 | 0.495952 | 1.46E-34 |
| RB1 | AC016831.4 | 0.495959 | 1.46E-34 |
| PTEN | AC005479.1 | 0.495987 | 1.44E-34 |
| PIK3C3 | AC007014.2 | 0.496006 | 1.43E-34 |
| ATG16L2 | AC010542.5 | 0.49605 | 1.41E-34 |
| ATG16L2 | AC244197.2 | 0.496076 | 1.40E-34 |
| ATG16L2 | AC027796.4 | 0.496086 | 1.39E-34 |
| RB1CC1 | AC005632.2 | 0.49609 | 1.39E-34 |
| RB1CC1 | AC130650.2 | 0.496093 | 1.39E-34 |
| ATG12 | AC022400.5 | 0.496129 | 1.37E-34 |
| FOXO3 | AC025917.1 | 0.496137 | 1.37E-34 |
| CFLAR | LINC01290 | 0.496169 | 1.35E-34 |
| FOXO1 | AC012181.2 | 0.496183 | 1.35E-34 |
| KLHL24 | AC127024.5 | 0.496218 | 1.33E-34 |
| CASP1 | HCP5 | 0.49625 | 1.32E-34 |
| MAPK8 | AL117336.2 | 0.496275 | 1.30E-34 |
| EIF2AK2 | AC078778.1 | 0.496294 | 1.30E-34 |
| WDFY3 | AL031673.1 | 0.496319 | 1.28E-34 |
| ATG16L2 | TMEM147-AS1 | 0.496329 | 1.28E-34 |
| NAF1 | AL513327.1 | 0.496338 | 1.28E-34 |
| DAPK2 | PRKCZ-AS1 | 0.496355 | 1.27E-34 |
| PTEN | AC062037.2 | 0.496372 | 1.26E-34 |
| PIK3C3 | AL391834.1 | 0.496405 | 1.25E-34 |
| RB1CC1 | AC104695.3 | 0.496483 | 1.21E-34 |
| RB1 | MAL2-AS1 | 0.496514 | 1.20E-34 |
| WDFY3 | AC005479.1 | 0.496532 | 1.19E-34 |
| ATG7 | AC020913.3 | 0.496555 | 1.18E-34 |
| RPS6KB1 | AL163051.2 | 0.496579 | 1.17E-34 |
| CFLAR | GABPB1-AS1 | 0.496638 | 1.15E-34 |
| KLHL24 | AC015911.3 | 0.49664 | 1.15E-34 |
| GOPC | AC080162.1 | 0.496641 | 1.15E-34 |
| PTEN | AL162274.2 | 0.496667 | 1.14E-34 |
| PTEN | ACBD3-AS1 | 0.496698 | 1.12E-34 |
| GOPC | AC090425.2 | 0.496725 | 1.11E-34 |
| RB1 | NPTN-IT1 | 0.496726 | 1.11E-34 |
| EIF2AK2 | LINC00641 | 0.496737 | 1.11E-34 |
| ATG4B | H1FX-AS1 | 0.496763 | 1.10E-34 |
| RB1CC1 | AC079684.1 | 0.496773 | 1.09E-34 |
| GOPC | SCARNA9 | 0.496783 | 1.09E-34 |
| TSC2 | PTOV1-AS2 | 0.49681 | 1.08E-34 |
| RB1 | AP001033.2 | 0.496816 | 1.08E-34 |
| NAF1 | AL596325.2 | 0.496816 | 1.08E-34 |
| ATG12 | AC010168.2 | 0.496844 | 1.07E-34 |
| KLHL24 | AC005920.2 | 0.496845 | 1.07E-34 |
| TSC1 | ALG13-AS1 | 0.496858 | 1.06E-34 |
| PTEN | AC098484.1 | 0.49686 | 1.06E-34 |
| NAF1 | AC068152.1 | 0.496903 | 1.04E-34 |
| KLHL24 | SNHG26 | 0.496907 | 1.04E-34 |
| ATG12 | AL049840.2 | 0.496929 | 1.04E-34 |
| RB1CC1 | AL158166.2 | 0.496931 | 1.03E-34 |
| CAPN10 | AC009065.8 | 0.49694 | 1.03E-34 |
| CAPN10 | LINC00106 | 0.496953 | 1.03E-34 |
| CFLAR | AC084871.1 | 0.496985 | 1.01E-34 |
| CFLAR | AL592148.3 | 0.497017 | 1.00E-34 |
| EIF2AK2 | AC124312.4 | 0.497041 | 9.95E-35 |
| PTEN | AC009120.2 | 0.497066 | 9.86E-35 |
| EIF2AK2 | AC012181.1 | 0.497155 | 9.55E-35 |
| MAPK8 | KDM4A-AS1 | 0.497217 | 9.35E-35 |
| KLHL24 | AC002550.2 | 0.497217 | 9.35E-35 |
| FOXO3 | AC098851.1 | 0.497247 | 9.25E-35 |
| ATG16L2 | AC104463.2 | 0.497256 | 9.22E-35 |
| NAF1 | AC008124.1 | 0.497263 | 9.20E-35 |
| NAF1 | ADNP-AS1 | 0.497267 | 9.18E-35 |
| MAPK8 | AC008966.2 | 0.497287 | 9.12E-35 |
| MAPK8 | AC079907.1 | 0.497308 | 9.05E-35 |
| PIK3C3 | MUC20-OT1 | 0.497324 | 9.00E-35 |
| KLHL24 | AC004908.2 | 0.497348 | 8.92E-35 |
| CFLAR | AP002907.1 | 0.497386 | 8.81E-35 |
| EIF2AK2 | LINC00513 | 0.497419 | 8.70E-35 |
| ATG12 | AL512791.1 | 0.49749 | 8.49E-35 |
| RB1 | AC026202.2 | 0.497491 | 8.48E-35 |
| ATG16L2 | AC062037.2 | 0.497493 | 8.48E-35 |
| RB1 | AC010761.3 | 0.4975 | 8.46E-35 |
| NAF1 | AC006270.1 | 0.497501 | 8.45E-35 |
| NAF1 | ADAMTSL4-AS1 | 0.497542 | 8.33E-35 |
| RB1 | AL133445.2 | 0.497649 | 8.02E-35 |
| ATG16L2 | AC005674.2 | 0.497672 | 7.96E-35 |
| RB1 | AC114760.2 | 0.497729 | 7.80E-35 |
| RB1 | LIMS1-AS1 | 0.497734 | 7.78E-35 |
| TSC1 | AC012181.1 | 0.497767 | 7.69E-35 |
| TSC1 | AC004908.1 | 0.49777 | 7.68E-35 |
| TSC1 | AC093726.2 | 0.497796 | 7.61E-35 |
| RB1CC1 | CD44-AS1 | 0.497817 | 7.56E-35 |
| BIRC6 | LINC02035 | 0.497822 | 7.55E-35 |
| NAF1 | ANKRD10-IT1 | 0.497845 | 7.48E-35 |
| FOXO1 | MALAT1 | 0.497851 | 7.47E-35 |
| ATG4B | AC011498.6 | 0.497931 | 7.26E-35 |
| IL24 | LINC00426 | 0.497944 | 7.22E-35 |
| KLHL24 | AC010615.2 | 0.497961 | 7.18E-35 |
| RB1 | AP001178.2 | 0.497992 | 7.10E-35 |
| RPS6KB1 | SNHG16 | 0.497994 | 7.10E-35 |
| RPS6KB1 | AC007014.2 | 0.498049 | 6.96E-35 |
| RB1CC1 | MALAT1 | 0.498068 | 6.91E-35 |
| DAPK2 | AC027288.3 | 0.498084 | 6.87E-35 |
| CASP1 | AC138207.5 | 0.498099 | 6.84E-35 |
| KIF5B | NORAD | 0.498111 | 6.81E-35 |
| RPS6KB1 | AC096992.2 | 0.498179 | 6.65E-35 |
| RB1 | AC022150.4 | 0.498216 | 6.56E-35 |
| NAF1 | AL133342.1 | 0.498217 | 6.56E-35 |
| BNIP1 | MAPKAPK5-AS1 | 0.498225 | 6.54E-35 |
| RPS6KB1 | RFX3-AS1 | 0.49829 | 6.39E-35 |
| ATG16L2 | AC020558.2 | 0.498328 | 6.30E-35 |
| BIRC6 | AC010615.2 | 0.498328 | 6.30E-35 |
| KLHL24 | ARHGAP31-AS1 | 0.498328 | 6.30E-35 |
| PIK3C3 | DNM3OS | 0.498351 | 6.25E-35 |
| FOXO1 | AC138956.1 | 0.498383 | 6.18E-35 |
| GOPC | AL139120.1 | 0.498403 | 6.14E-35 |
| KLHL24 | AC055822.1 | 0.498462 | 6.01E-35 |
| GOPC | AC026356.1 | 0.498488 | 5.96E-35 |
| RPS6KB1 | AL049840.1 | 0.498541 | 5.84E-35 |
| GOPC | MCM3AP-AS1 | 0.498545 | 5.84E-35 |
| PIK3C3 | AC018682.1 | 0.49855 | 5.83E-35 |
| CFLAR | AP002336.2 | 0.498591 | 5.74E-35 |
| RB1CC1 | MIR29B2CHG | 0.498671 | 5.58E-35 |
| WDFY3 | AL162274.2 | 0.498681 | 5.56E-35 |
| KLHL24 | AC116366.1 | 0.498683 | 5.56E-35 |
| CAPN10 | SNHG12 | 0.498703 | 5.52E-35 |
| CFLAR | AC006378.1 | 0.498713 | 5.50E-35 |
| CFLAR | AC253576.2 | 0.498729 | 5.46E-35 |
| KLHL24 | KLF7-IT1 | 0.49877 | 5.39E-35 |
| FOXO3 | AC011939.2 | 0.498772 | 5.38E-35 |
| RB1 | SOS1-IT1 | 0.4988 | 5.33E-35 |
| TSC1 | AC087752.4 | 0.498815 | 5.30E-35 |
| CFLAR | AL137779.2 | 0.498824 | 5.28E-35 |
| EIF2AK2 | AL162724.1 | 0.498828 | 5.28E-35 |
| ATG12 | AC073534.1 | 0.498845 | 5.24E-35 |
| TSC1 | AC007216.4 | 0.498853 | 5.23E-35 |
| BIRC6 | AL031673.1 | 0.498878 | 5.18E-35 |
| ATG12 | AL158166.1 | 0.498939 | 5.07E-35 |
| EIF2AK2 | LINC00852 | 0.498948 | 5.06E-35 |
| ATG16L2 | AC008764.2 | 0.49895 | 5.05E-35 |
| RB1 | KLF7-IT1 | 0.498993 | 4.98E-35 |
| FOXO1 | EP300-AS1 | 0.499019 | 4.93E-35 |
| MAPK8 | AC120349.1 | 0.499019 | 4.93E-35 |
| ATG2B | AL359915.2 | 0.49902 | 4.93E-35 |
| BNIP1 | ZFAS1 | 0.499084 | 4.82E-35 |
| SPNS1 | PTOV1-AS2 | 0.499093 | 4.80E-35 |
| PTEN | AC024060.1 | 0.499124 | 4.75E-35 |
| RPS6KB1 | AC108449.2 | 0.499132 | 4.74E-35 |
| PIK3C3 | AC010168.2 | 0.499132 | 4.73E-35 |
| RPS6KB1 | AP000766.1 | 0.499136 | 4.73E-35 |
| RB1CC1 | AC005856.1 | 0.499173 | 4.67E-35 |
| EIF2AK2 | MALAT1 | 0.499196 | 4.63E-35 |
| RB1 | AC063965.1 | 0.499223 | 4.58E-35 |
| EIF2AK2 | AC084824.4 | 0.499237 | 4.56E-35 |
| ATF4 | AL021707.2 | 0.499239 | 4.56E-35 |
| ATG16L2 | AL512791.1 | 0.499241 | 4.55E-35 |
| FOXO1 | AC083843.2 | 0.499247 | 4.55E-35 |
| EIF2AK2 | MACC1-AS1 | 0.499248 | 4.54E-35 |
| ATG16L2 | ZNF32-AS2 | 0.499266 | 4.51E-35 |
| PTEN | AC004067.1 | 0.499268 | 4.51E-35 |
| GOPC | AC012170.2 | 0.499268 | 4.51E-35 |
| GOPC | AC007684.1 | 0.499288 | 4.48E-35 |
| RPS6KB1 | AC016831.4 | 0.499291 | 4.47E-35 |
| RB1 | AC018752.1 | 0.499315 | 4.44E-35 |
| BIRC6 | CCDC18-AS1 | 0.499322 | 4.42E-35 |
| ATG12 | SAMD12-AS1 | 0.499328 | 4.42E-35 |
| FOXO1 | AC016542.1 | 0.499359 | 4.37E-35 |
| CFLAR | AC100830.2 | 0.499414 | 4.28E-35 |
| MAPK8 | AL645568.1 | 0.499446 | 4.23E-35 |
| FOXO1 | AC092794.1 | 0.499447 | 4.23E-35 |
| TSC1 | IGBP1-AS1 | 0.499531 | 4.11E-35 |
| PTEN | AL356356.1 | 0.499534 | 4.10E-35 |
| PIK3C3 | AC005021.1 | 0.499556 | 4.07E-35 |
| KLHL24 | AL137003.2 | 0.499678 | 3.90E-35 |
| IL24 | LINC01857 | 0.499746 | 3.80E-35 |
| CFLAR | ZNF32-AS2 | 0.499778 | 3.76E-35 |
| GOPC | AL031670.1 | 0.499783 | 3.75E-35 |
| CFLAR | AC093110.1 | 0.499788 | 3.75E-35 |
| RPS6KB1 | AL162724.1 | 0.499835 | 3.69E-35 |
| NAF1 | AL391834.1 | 0.499858 | 3.65E-35 |
| TSC1 | AC011462.4 | 0.49986 | 3.65E-35 |
| WDFY3 | FAM160A1-DT | 0.499865 | 3.65E-35 |
| RB1CC1 | ANKRD10-IT1 | 0.499881 | 3.62E-35 |
| GOPC | Z98884.2 | 0.499889 | 3.62E-35 |
| CFLAR | AP001432.1 | 0.499891 | 3.61E-35 |
| ATG12 | AC025171.4 | 0.499963 | 3.52E-35 |
| EIF2AK2 | AL606834.2 | 0.499965 | 3.52E-35 |
| TSC1 | PDXDC2P-NPIPB14P | 0.5 | 3.47E-35 |
| RB1 | AC080162.1 | 0.50003 | 3.44E-35 |
| ATG12 | PSMA3-AS1 | 0.500067 | 3.39E-35 |
| PIK3C3 | TMEM161B-AS1 | 0.500107 | 3.34E-35 |
| RPS6KB1 | AC010834.3 | 0.500111 | 3.34E-35 |
| RPS6KB1 | AC130895.1 | 0.500114 | 3.34E-35 |
| EIF2AK2 | LAMC1-AS1 | 0.500179 | 3.26E-35 |
| CFLAR | AC018752.1 | 0.500182 | 3.26E-35 |
| NAF1 | RBMS3-AS3 | 0.500198 | 3.24E-35 |
| RPS6KB1 | AL355488.1 | 0.50025 | 3.18E-35 |
| NAF1 | AL022067.1 | 0.50028 | 3.14E-35 |
| RB1 | AC131971.1 | 0.50029 | 3.13E-35 |
| CFLAR | AC018809.2 | 0.50038 | 3.03E-35 |
| CFLAR | AL355075.2 | 0.500381 | 3.03E-35 |
| NAF1 | AF117829.1 | 0.500397 | 3.01E-35 |
| EIF2AK2 | AC000123.1 | 0.500421 | 2.99E-35 |
| EIF2AK2 | AC002064.2 | 0.500427 | 2.98E-35 |
| ATG2B | AC084117.1 | 0.500458 | 2.95E-35 |
| GOPC | NPTN-IT1 | 0.500466 | 2.94E-35 |
| KLHL24 | FMR1-IT1 | 0.500468 | 2.94E-35 |
| FOXO1 | AC018926.3 | 0.500496 | 2.91E-35 |
| EIF4EBP1 | AP003119.3 | 0.500514 | 2.89E-35 |
| CFLAR | AC007014.2 | 0.500543 | 2.86E-35 |
| NAF1 | AL355075.2 | 0.500557 | 2.85E-35 |
| TSC1 | AC092611.2 | 0.500558 | 2.85E-35 |
| WDFY3 | TMEM9B-AS1 | 0.500598 | 2.80E-35 |
| CCL2 | LINC01094 | 0.500627 | 2.78E-35 |
| ATG4B | AC132872.1 | 0.500657 | 2.75E-35 |
| RPS6KB1 | AC068790.5 | 0.500686 | 2.72E-35 |
| NAF1 | TMEM161B-AS1 | 0.500689 | 2.72E-35 |
| EIF2AK2 | AL162724.2 | 0.500709 | 2.70E-35 |
| PIK3C3 | AC087392.1 | 0.500719 | 2.69E-35 |
| RB1 | AC138393.3 | 0.500725 | 2.68E-35 |
| RB1 | AC004884.2 | 0.500745 | 2.66E-35 |
| RPS6KB1 | AL359697.1 | 0.500753 | 2.65E-35 |
| ATG16L2 | H1FX-AS1 | 0.500765 | 2.64E-35 |
| PIK3C3 | AC074032.1 | 0.500786 | 2.62E-35 |
| NAF1 | DNM3OS | 0.500792 | 2.62E-35 |
| RB1 | ITCH-IT1 | 0.50084 | 2.57E-35 |
| RB1 | ACAP2-IT1 | 0.500922 | 2.50E-35 |
| MAPK8 | AC013403.2 | 0.500947 | 2.48E-35 |
| EIF2AK2 | LINC00894 | 0.500954 | 2.47E-35 |
| KLHL24 | AC009032.1 | 0.500986 | 2.44E-35 |
| PIK3C3 | AL683813.1 | 0.500987 | 2.44E-35 |
| WDFY3 | LINC01184 | 0.501011 | 2.42E-35 |
| GOPC | AC073487.1 | 0.501013 | 2.42E-35 |
| TSC2 | AL022328.2 | 0.501021 | 2.41E-35 |
| CFLAR | AC018638.7 | 0.501067 | 2.37E-35 |
| NAF1 | AL513550.1 | 0.501087 | 2.35E-35 |
| RB1CC1 | AP001469.2 | 0.501119 | 2.33E-35 |
| FOXO3 | AP000766.1 | 0.501139 | 2.31E-35 |
| ATG16L2 | AP001160.1 | 0.501156 | 2.30E-35 |
| GOPC | LINC01355 | 0.501173 | 2.28E-35 |
| RPS6KB1 | AL138963.1 | 0.501228 | 2.24E-35 |
| CFLAR | FMR1-IT1 | 0.501238 | 2.23E-35 |
| TSC1 | AC025171.2 | 0.501279 | 2.20E-35 |
| TSC1 | AL683813.1 | 0.50129 | 2.19E-35 |
| CFLAR | AC096992.2 | 0.501347 | 2.14E-35 |
| RB1CC1 | AC087286.1 | 0.501372 | 2.13E-35 |
| RPS6KB1 | AL157402.2 | 0.501387 | 2.11E-35 |
| FOXO1 | AL359962.2 | 0.501398 | 2.11E-35 |
| CFLAR | ATP1B3-AS1 | 0.501401 | 2.10E-35 |
| EIF2AK2 | AC078846.1 | 0.501434 | 2.08E-35 |
| RPS6KB1 | N4BP2L2-IT2 | 0.501446 | 2.07E-35 |
| MAPK8 | AL157786.1 | 0.501449 | 2.07E-35 |
| ATG4B | AL109811.3 | 0.50145 | 2.07E-35 |
| NAF1 | AC024075.1 | 0.501453 | 2.06E-35 |
| EIF2AK2 | AC002550.2 | 0.501457 | 2.06E-35 |
| NAF1 | AC007216.4 | 0.501462 | 2.06E-35 |
| NAF1 | AC104695.3 | 0.501512 | 2.02E-35 |
| FOXO1 | AC090198.1 | 0.501531 | 2.01E-35 |
| FOXO1 | HCG18 | 0.501537 | 2.00E-35 |
| TSC1 | AL139289.1 | 0.501543 | 2.00E-35 |
| EIF2AK2 | DNM3OS | 0.50163 | 1.94E-35 |
| RPS6KB1 | AC058791.1 | 0.501674 | 1.91E-35 |
| ATG16L2 | Z69706.1 | 0.501725 | 1.87E-35 |
| CASP1 | TRG-AS1 | 0.501726 | 1.87E-35 |
| EIF2AK2 | SAP30L-AS1 | 0.501735 | 1.87E-35 |
| GOPC | AL049840.2 | 0.501787 | 1.83E-35 |
| PIK3C3 | AC105389.2 | 0.501787 | 1.83E-35 |
| RB1 | AC124283.3 | 0.501808 | 1.82E-35 |
| RB1CC1 | AC074032.1 | 0.501809 | 1.82E-35 |
| ATG7 | KLF7-IT1 | 0.501812 | 1.82E-35 |
| RB1CC1 | AP001178.2 | 0.501845 | 1.79E-35 |
| KLHL24 | AC010201.2 | 0.501847 | 1.79E-35 |
| PTEN | AC073534.1 | 0.501849 | 1.79E-35 |
| RB1 | AL139407.1 | 0.501919 | 1.75E-35 |
| CFLAR | AC124312.4 | 0.501934 | 1.74E-35 |
| PIK3C3 | AC127024.5 | 0.501954 | 1.72E-35 |
| BECN1 | AC005288.1 | 0.50197 | 1.71E-35 |
| GOPC | AL359715.3 | 0.501978 | 1.71E-35 |
| ATG2B | AL442125.2 | 0.501984 | 1.71E-35 |
| PIK3C3 | ADNP-AS1 | 0.501987 | 1.70E-35 |
| PIK3C3 | ATP1A1-AS1 | 0.501989 | 1.70E-35 |
| FOXO3 | AL133371.2 | 0.502026 | 1.68E-35 |
| PIK3C3 | AC078846.1 | 0.502057 | 1.66E-35 |
| MAPK8 | AC093388.1 | 0.502119 | 1.63E-35 |
| ATG2B | AC016957.2 | 0.502156 | 1.60E-35 |
| GOPC | AC087286.4 | 0.502169 | 1.60E-35 |
| RPS6KB1 | AC063965.1 | 0.502182 | 1.59E-35 |
| RB1CC1 | AL645568.1 | 0.502203 | 1.58E-35 |
| RPS6KB1 | AC087284.1 | 0.50222 | 1.57E-35 |
| CFLAR | AC012557.1 | 0.502233 | 1.56E-35 |
| TSC1 | AP001469.3 | 0.502249 | 1.55E-35 |
| ATG12 | KDM4A-AS1 | 0.502251 | 1.55E-35 |
| ATG4B | AC011472.1 | 0.502268 | 1.54E-35 |
| TSC1 | AL355075.2 | 0.502286 | 1.53E-35 |
| PIK3C3 | AC010226.1 | 0.502288 | 1.53E-35 |
| KLHL24 | AC067817.2 | 0.502293 | 1.53E-35 |
| GOPC | AF129075.1 | 0.502301 | 1.52E-35 |
| EIF2AK2 | AL157786.1 | 0.50231 | 1.52E-35 |
| NAF1 | ALMS1-IT1 | 0.502323 | 1.51E-35 |
| PTEN | AC073046.1 | 0.502326 | 1.51E-35 |
| MAPK8 | Z98884.2 | 0.502346 | 1.50E-35 |
| RPS6KB1 | AP001469.2 | 0.502362 | 1.49E-35 |
| KLHL24 | AC004884.2 | 0.502366 | 1.49E-35 |
| GOPC | AL590723.1 | 0.502391 | 1.47E-35 |
| ATG2B | SP2-AS1 | 0.50242 | 1.46E-35 |
| CFLAR | AC090739.1 | 0.502451 | 1.44E-35 |
| ATG2B | AC007991.2 | 0.502458 | 1.44E-35 |
| BIRC6 | AC087752.3 | 0.502497 | 1.42E-35 |
| EIF2AK2 | AC066613.1 | 0.502536 | 1.40E-35 |
| TSC1 | RHOA-IT1 | 0.502546 | 1.39E-35 |
| WDFY3 | AC006001.2 | 0.502557 | 1.39E-35 |
| ATG12 | AP001628.1 | 0.502557 | 1.39E-35 |
| ATG4B | AC012510.1 | 0.502566 | 1.38E-35 |
| CAPN10 | AC020907.4 | 0.502572 | 1.38E-35 |
| RB1CC1 | AL596325.2 | 0.502601 | 1.37E-35 |
| TSC1 | AL136531.1 | 0.502609 | 1.36E-35 |
| ATG12 | AC009090.3 | 0.502617 | 1.36E-35 |
| KLHL24 | MIR181A2HG | 0.502638 | 1.35E-35 |
| MAPK8 | OIP5-AS1 | 0.502734 | 1.30E-35 |
| ATG4B | SNHG11 | 0.50274 | 1.30E-35 |
| PTEN | AC020571.1 | 0.502761 | 1.29E-35 |
| RPS6KB1 | AC048344.4 | 0.502772 | 1.28E-35 |
| CFLAR | AC002064.2 | 0.502792 | 1.28E-35 |
| GOPC | LIMS1-AS1 | 0.502804 | 1.27E-35 |
| TSC1 | AC066613.1 | 0.502842 | 1.25E-35 |
| PIK3C3 | ERVK13-1 | 0.502882 | 1.23E-35 |
| CFLAR | AC048341.1 | 0.502884 | 1.23E-35 |
| EIF2AK2 | AC026202.2 | 0.502889 | 1.23E-35 |
| ATG12 | AC005261.1 | 0.502899 | 1.23E-35 |
| ATG16L2 | AC093495.1 | 0.502929 | 1.21E-35 |
| MAPK8 | AC027097.2 | 0.502997 | 1.18E-35 |
| MAPK8 | AC026470.2 | 0.503076 | 1.15E-35 |
| RPS6KB1 | AL365277.1 | 0.50315 | 1.12E-35 |
| RB1 | AC087286.4 | 0.503153 | 1.12E-35 |
| BIRC6 | GK-IT1 | 0.503171 | 1.11E-35 |
| RPS6KB1 | OSMR-AS1 | 0.503186 | 1.11E-35 |
| RPS6KB1 | AC006270.1 | 0.503187 | 1.11E-35 |
| KLHL24 | AC015849.3 | 0.503315 | 1.06E-35 |
| RPS6KB1 | AC104695.3 | 0.503332 | 1.05E-35 |
| RPS6KB1 | AL031775.2 | 0.503368 | 1.04E-35 |
| KLHL24 | AP001432.1 | 0.503377 | 1.03E-35 |
| PTEN | USP46-AS1 | 0.503395 | 1.03E-35 |
| PTEN | AL136531.1 | 0.5034 | 1.02E-35 |
| ATG16L2 | SNHG20 | 0.503403 | 1.02E-35 |
| ATG12 | NR2F1-AS1 | 0.503404 | 1.02E-35 |
| ATG12 | AC002553.1 | 0.503415 | 1.02E-35 |
| RB1 | AC068790.2 | 0.503489 | 9.91E-36 |
| NAF1 | AC245014.3 | 0.503494 | 9.90E-36 |
| WDFY3 | AC139795.2 | 0.503516 | 9.82E-36 |
| PIK3C3 | C5orf56 | 0.503537 | 9.74E-36 |
| RB1 | AL117381.1 | 0.503558 | 9.67E-36 |
| MAPK8 | AC002553.1 | 0.503571 | 9.62E-36 |
| RPS6KB1 | AC090948.1 | 0.503589 | 9.56E-36 |
| KLHL24 | AC005034.5 | 0.5036 | 9.52E-36 |
| RB1 | AC019080.5 | 0.503688 | 9.23E-36 |
| ATG7 | AL157394.1 | 0.503693 | 9.21E-36 |
| CFLAR | AL359915.2 | 0.503702 | 9.18E-36 |
| GABARAPL2 | SCAMP1-AS1 | 0.503709 | 9.16E-36 |
| NAF1 | AC026356.1 | 0.503716 | 9.13E-36 |
| TSC1 | AC092119.2 | 0.503733 | 9.08E-36 |
| FOXO1 | ANKRD10-IT1 | 0.503742 | 9.05E-36 |
| ATG16L2 | CCDC18-AS1 | 0.503763 | 8.98E-36 |
| TSC1 | AC096586.2 | 0.503802 | 8.85E-36 |
| ATG2B | EGOT | 0.503852 | 8.69E-36 |
| CFLAR | AL031775.2 | 0.503862 | 8.66E-36 |
| ATG4B | LINC01786 | 0.503885 | 8.59E-36 |
| NAF1 | AC020915.2 | 0.503887 | 8.59E-36 |
| RB1 | AL158166.2 | 0.503912 | 8.51E-36 |
| LAMP1 | AC026979.2 | 0.503929 | 8.46E-36 |
| NAF1 | AC099343.2 | 0.503948 | 8.40E-36 |
| RPS6KB1 | AC087286.2 | 0.503972 | 8.33E-36 |
| PTEN | GK-IT1 | 0.503977 | 8.31E-36 |
| CFLAR | AC129510.1 | 0.503978 | 8.31E-36 |
| ATG2B | AC010542.5 | 0.503991 | 8.27E-36 |
| ATG7 | AL162724.1 | 0.504002 | 8.24E-36 |
| WDFY3 | AP001107.4 | 0.50402 | 8.18E-36 |
| RPS6KB1 | AL157392.3 | 0.504033 | 8.14E-36 |
| GOPC | AC008770.3 | 0.504047 | 8.10E-36 |
| ATG7 | AC002064.2 | 0.504095 | 7.96E-36 |
| KLHL24 | AL158166.2 | 0.504104 | 7.94E-36 |
| EIF2AK2 | NUTM2B-AS1 | 0.504135 | 7.85E-36 |
| NAF1 | EP300-AS1 | 0.504159 | 7.78E-36 |
| PIK3C3 | AL592148.3 | 0.504171 | 7.75E-36 |
| MAPK8 | AC011477.2 | 0.504204 | 7.65E-36 |
| GOPC | AC024075.3 | 0.504211 | 7.63E-36 |
| CFLAR | AC093726.2 | 0.504229 | 7.59E-36 |
| KLHL24 | AC002064.2 | 0.50424 | 7.56E-36 |
| ATG16L2 | AP003419.3 | 0.504258 | 7.51E-36 |
| CFLAR | AL157932.1 | 0.504264 | 7.49E-36 |
| ATG12 | AC008124.1 | 0.504289 | 7.42E-36 |
| CFLAR | AL133342.1 | 0.504301 | 7.39E-36 |
| PIK3C3 | AF178030.1 | 0.504303 | 7.39E-36 |
| KLHL24 | AC011472.4 | 0.50431 | 7.37E-36 |
| CFLAR | AL139041.1 | 0.504321 | 7.34E-36 |
| GOPC | Z83843.1 | 0.504324 | 7.33E-36 |
| CAPN10 | AC132872.3 | 0.504364 | 7.22E-36 |
| RB1 | AP001429.1 | 0.504383 | 7.17E-36 |
| RB1 | AL136115.2 | 0.504436 | 7.04E-36 |
| PIK3C3 | AC037198.2 | 0.504543 | 6.77E-36 |
| KLHL24 | SAP30L-AS1 | 0.504565 | 6.71E-36 |
| MAPK8 | AC024933.1 | 0.50457 | 6.70E-36 |
| MAPK8 | AL132989.1 | 0.504571 | 6.70E-36 |
| FOXO1 | AC087284.1 | 0.504591 | 6.65E-36 |
| GOPC | AC092801.1 | 0.504593 | 6.65E-36 |
| ATG12 | EP300-AS1 | 0.50465 | 6.51E-36 |
| MAPK8 | AL158212.3 | 0.504703 | 6.39E-36 |
| FOXO1 | AC068790.2 | 0.504733 | 6.32E-36 |
| ATG2B | AC025165.4 | 0.504854 | 6.05E-36 |
| FOXO1 | AC068790.3 | 0.504884 | 5.98E-36 |
| ATG2B | AC018926.2 | 0.504887 | 5.97E-36 |
| BIRC6 | AC011477.3 | 0.504917 | 5.91E-36 |
| ATG16L2 | AC008870.2 | 0.504923 | 5.90E-36 |
| GOPC | MAL2-AS1 | 0.504945 | 5.85E-36 |
| GOPC | AL136115.2 | 0.504962 | 5.81E-36 |
| EIF2AK2 | AC130650.2 | 0.505009 | 5.72E-36 |
| ATG4B | ARRDC1-AS1 | 0.50501 | 5.71E-36 |
| KLHL24 | AC008906.1 | 0.505046 | 5.64E-36 |
| RB1CC1 | OTUD6B-AS1 | 0.505118 | 5.49E-36 |
| GOPC | AC011939.2 | 0.505127 | 5.48E-36 |
| ATG12 | AC090517.2 | 0.505132 | 5.47E-36 |
| KLHL24 | STARD4-AS1 | 0.505183 | 5.37E-36 |
| FOXO1 | AC004223.3 | 0.505189 | 5.35E-36 |
| RB1CC1 | AC093388.1 | 0.505216 | 5.30E-36 |
| MAPK8 | AC074033.1 | 0.505227 | 5.28E-36 |
| ATG2B | AC092123.1 | 0.505244 | 5.25E-36 |
| RPS6KB1 | AC253536.3 | 0.505297 | 5.15E-36 |
| ATG4B | MHENCR | 0.505308 | 5.13E-36 |
| RB1CC1 | AC004477.3 | 0.505308 | 5.13E-36 |
| TSC1 | AC090198.1 | 0.505318 | 5.11E-36 |
| RB1CC1 | AC007038.2 | 0.505346 | 5.06E-36 |
| TSC1 | LINC01004 | 0.505351 | 5.05E-36 |
| KLHL24 | AL592148.3 | 0.505357 | 5.04E-36 |
| GOPC | AC087286.2 | 0.505373 | 5.01E-36 |
| MAPK8 | AL359076.1 | 0.505394 | 4.97E-36 |
| RPS6KB1 | AL133342.1 | 0.505395 | 4.97E-36 |
| BIRC6 | AC005479.2 | 0.505397 | 4.96E-36 |
| KLHL24 | AC093388.1 | 0.505415 | 4.93E-36 |
| RB1CC1 | AC011477.2 | 0.50543 | 4.91E-36 |
| FOXO1 | AC010536.2 | 0.505433 | 4.90E-36 |
| PIK3C3 | AC019080.5 | 0.50547 | 4.83E-36 |
| FOXO3 | AC024075.3 | 0.505481 | 4.81E-36 |
| ATG4B | AC011462.4 | 0.5055 | 4.78E-36 |
| RB1 | AC090948.2 | 0.505501 | 4.78E-36 |
| GOPC | ABALON | 0.505535 | 4.72E-36 |
| EIF2AK2 | AC008966.2 | 0.505536 | 4.72E-36 |
| ATG4B | AC020558.2 | 0.505546 | 4.70E-36 |
| FOXO3 | AL031716.1 | 0.505597 | 4.62E-36 |
| CFLAR | AC008969.1 | 0.505617 | 4.58E-36 |
| KLHL24 | AC037487.2 | 0.505634 | 4.55E-36 |
| RPS6KB1 | LAMC1-AS1 | 0.50565 | 4.53E-36 |
| MAPK8 | AC002550.2 | 0.505686 | 4.47E-36 |
| PIK3R4 | AC092611.2 | 0.505703 | 4.44E-36 |
| BIRC6 | LANCL1-AS1 | 0.505709 | 4.43E-36 |
| ATG7 | AC084871.1 | 0.505715 | 4.42E-36 |
| CFLAR | AC025287.3 | 0.505739 | 4.38E-36 |
| CFLAR | AC234772.2 | 0.505804 | 4.28E-36 |
| NAF1 | AC009120.3 | 0.505841 | 4.22E-36 |
| CFLAR | AP001486.2 | 0.505841 | 4.22E-36 |
| BIRC6 | AC120053.1 | 0.505844 | 4.22E-36 |
| PTEN | AC139887.2 | 0.505851 | 4.21E-36 |
| WDFY3 | AC005332.3 | 0.50587 | 4.18E-36 |
| RPS6KB1 | AC090425.2 | 0.505892 | 4.15E-36 |
| RPS6KB1 | AL049869.3 | 0.505907 | 4.12E-36 |
| GOPC | FTX | 0.505912 | 4.12E-36 |
| MAPK8 | AC005856.1 | 0.505949 | 4.06E-36 |
| RPS6KB1 | AP001178.2 | 0.505985 | 4.01E-36 |
| WIPI2 | AC091729.3 | 0.505992 | 4.00E-36 |
| TSC1 | AC006017.1 | 0.506025 | 3.95E-36 |
| ATG7 | AL133330.1 | 0.506035 | 3.93E-36 |
| MAPK8 | AC105389.2 | 0.506116 | 3.82E-36 |
| ATG2B | LINC01376 | 0.506205 | 3.70E-36 |
| CFLAR | AC008870.2 | 0.506213 | 3.69E-36 |
| GOPC | AC004832.5 | 0.50625 | 3.64E-36 |
| RB1CC1 | AC107027.3 | 0.506309 | 3.56E-36 |
| FOXO1 | AL157392.4 | 0.506314 | 3.55E-36 |
| RPS6KB1 | AC093788.1 | 0.506324 | 3.54E-36 |
| FOXO3 | AL122035.1 | 0.506337 | 3.52E-36 |
| MAPK8 | AF178030.1 | 0.506375 | 3.48E-36 |
| TSC1 | AC005104.1 | 0.506408 | 3.43E-36 |
| GOPC | AL139407.1 | 0.506428 | 3.41E-36 |
| ATG2B | AC073655.2 | 0.506436 | 3.40E-36 |
| NAF1 | AL122035.1 | 0.506436 | 3.40E-36 |
| KLHL24 | HMGN3-AS1 | 0.506455 | 3.38E-36 |
| GOPC | AP000692.1 | 0.506594 | 3.21E-36 |
| RB1 | AL590723.1 | 0.506632 | 3.16E-36 |
| ATG4B | AL365330.1 | 0.506646 | 3.15E-36 |
| TSC1 | AC012467.1 | 0.506675 | 3.12E-36 |
| ATG7 | AC005632.2 | 0.50671 | 3.08E-36 |
| PIK3C3 | AC018690.1 | 0.506759 | 3.02E-36 |
| PTEN | DUBR | 0.506767 | 3.01E-36 |
| MAPK8 | AL391834.1 | 0.5068 | 2.98E-36 |
| PIK3C3 | AC067852.3 | 0.506802 | 2.97E-36 |
| PIK3C3 | AC091185.1 | 0.50683 | 2.94E-36 |
| NAF1 | AC015849.3 | 0.506863 | 2.91E-36 |
| EIF2AK2 | SCAANT1 | 0.506875 | 2.90E-36 |
| NAF1 | AC093799.1 | 0.506885 | 2.89E-36 |
| GOPC | AC007878.1 | 0.506899 | 2.87E-36 |
| TSC1 | AC097376.2 | 0.506949 | 2.82E-36 |
| ULK3 | PTOV1-AS2 | 0.506953 | 2.81E-36 |
| PTEN | AC092279.1 | 0.506986 | 2.78E-36 |
| GOPC | LINC00216 | 0.507126 | 2.64E-36 |
| RB1CC1 | AL391834.1 | 0.507141 | 2.63E-36 |
| FOXO1 | AL513327.1 | 0.507142 | 2.63E-36 |
| NAF1 | AC018809.2 | 0.507194 | 2.58E-36 |
| CFLAR | AL513327.1 | 0.507206 | 2.57E-36 |
| EIF2AK2 | AL391834.1 | 0.507251 | 2.52E-36 |
| PIK3C3 | ATP1B3-AS1 | 0.507287 | 2.49E-36 |
| GOPC | AC098851.1 | 0.50731 | 2.47E-36 |
| RPS6KB1 | AC090181.2 | 0.50733 | 2.45E-36 |
| BIRC6 | AL139041.1 | 0.507338 | 2.44E-36 |
| ATG4B | ASB16-AS1 | 0.507391 | 2.40E-36 |
| IFNG | LINC02362 | 0.507417 | 2.37E-36 |
| RAF1 | FGD5-AS1 | 0.507428 | 2.37E-36 |
| EIF2AK2 | AC005920.2 | 0.507462 | 2.34E-36 |
| PIK3C3 | RAP2C-AS1 | 0.507466 | 2.33E-36 |
| CFLAR | PWAR6 | 0.507499 | 2.30E-36 |
| MAPK8 | AC124312.2 | 0.50751 | 2.30E-36 |
| FOXO1 | AC253536.3 | 0.50754 | 2.27E-36 |
| BIRC6 | AL121772.3 | 0.507592 | 2.23E-36 |
| NAF1 | AL157392.3 | 0.507603 | 2.22E-36 |
| EIF2AK2 | AL359915.2 | 0.507647 | 2.18E-36 |
| MAPK8 | AC093484.4 | 0.507655 | 2.18E-36 |
| EIF2AK2 | AC100830.2 | 0.50766 | 2.17E-36 |
| RB1CC1 | AC073651.1 | 0.507674 | 2.16E-36 |
| KLHL24 | AC025171.3 | 0.507691 | 2.15E-36 |
| FOXO1 | NDUFV2-AS1 | 0.507709 | 2.13E-36 |
| KLHL24 | AL359715.3 | 0.507726 | 2.12E-36 |
| TSC1 | HCG27 | 0.507732 | 2.12E-36 |
| KLHL24 | AC005261.1 | 0.507752 | 2.10E-36 |
| BIRC6 | AC016957.2 | 0.507803 | 2.06E-36 |
| RPS6KB1 | AC004832.5 | 0.507826 | 2.04E-36 |
| FOXO1 | ACTA2-AS1 | 0.507844 | 2.03E-36 |
| FOXO1 | ADAMTSL4-AS1 | 0.507856 | 2.02E-36 |
| MAPK8 | AC066613.1 | 0.507873 | 2.01E-36 |
| ATG16L2 | AC232271.1 | 0.507941 | 1.96E-36 |
| RPS6KB1 | GMDS-DT | 0.507948 | 1.95E-36 |
| BIRC6 | AC125257.1 | 0.507955 | 1.95E-36 |
| EIF2AK2 | AC007216.4 | 0.507957 | 1.95E-36 |
| NAF1 | AC116366.1 | 0.507959 | 1.95E-36 |
| EIF2AK2 | PSPC1-AS2 | 0.50796 | 1.95E-36 |
| CFLAR | AC010201.2 | 0.507967 | 1.94E-36 |
| KLHL24 | LAMC1-AS1 | 0.508007 | 1.91E-36 |
| RB1CC1 | AC016590.2 | 0.508012 | 1.91E-36 |
| ATG12 | AC084824.5 | 0.508075 | 1.87E-36 |
| GOPC | AP001432.1 | 0.508105 | 1.85E-36 |
| BCL2 | AC007996.1 | 0.50812 | 1.84E-36 |
| CFLAR | AC002550.2 | 0.508121 | 1.83E-36 |
| ATG12 | AC107068.1 | 0.50816 | 1.81E-36 |
| MAPK8 | AL513550.1 | 0.508193 | 1.79E-36 |
| TSC1 | MCCC1-AS1 | 0.508206 | 1.78E-36 |
| TSC1 | AC018809.2 | 0.508252 | 1.75E-36 |
| KLHL24 | AC009318.3 | 0.508262 | 1.74E-36 |
| BIRC6 | AC004908.3 | 0.508273 | 1.74E-36 |
| ATG7 | LINC01943 | 0.508273 | 1.73E-36 |
| RPS6KB1 | AC084824.4 | 0.50828 | 1.73E-36 |
| EIF2AK2 | AC002128.2 | 0.508293 | 1.72E-36 |
| CTSD | AC068580.3 | 0.50832 | 1.71E-36 |
| EIF2AK2 | LINC01376 | 0.508326 | 1.70E-36 |
| ATG16L2 | LINC01786 | 0.508335 | 1.70E-36 |
| CFLAR | MIR155HG | 0.508363 | 1.68E-36 |
| NAF1 | AL137782.1 | 0.508365 | 1.68E-36 |
| PTEN | AL031667.3 | 0.508371 | 1.67E-36 |
| WDFY3 | AC080013.4 | 0.508385 | 1.67E-36 |
| GOPC | AC090948.2 | 0.508393 | 1.66E-36 |
| PIK3C3 | AL157394.1 | 0.508429 | 1.64E-36 |
| GOPC | Z82243.1 | 0.508442 | 1.63E-36 |
| FOXO1 | AC009318.2 | 0.508443 | 1.63E-36 |
| RPS6KB1 | AC092801.1 | 0.508493 | 1.60E-36 |
| RPS6KB1 | AC253576.2 | 0.508503 | 1.59E-36 |
| TP73 | ACBD3-AS1 | 0.50858 | 1.55E-36 |
| TSC1 | AP001458.1 | 0.508595 | 1.54E-36 |
| BIRC6 | DUBR | 0.508618 | 1.53E-36 |
| MAPK8 | AP000786.1 | 0.508671 | 1.50E-36 |
| CFLAR | AC010536.2 | 0.508685 | 1.49E-36 |
| RPS6KB1 | MAL2-AS1 | 0.50869 | 1.49E-36 |
| PIK3C3 | AC093799.1 | 0.508692 | 1.49E-36 |
| ATG16L2 | AC073957.3 | 0.50872 | 1.47E-36 |
| MAPK8 | AC090579.1 | 0.508724 | 1.47E-36 |
| WDFY3 | AC062037.2 | 0.508752 | 1.46E-36 |
| TSC1 | AL162586.1 | 0.508773 | 1.44E-36 |
| PIK3C3 | AC002128.2 | 0.508782 | 1.44E-36 |
| GOPC | RHOA-IT1 | 0.508809 | 1.43E-36 |
| CFLAR | AL139120.1 | 0.50881 | 1.42E-36 |
| TSC1 | AC004771.1 | 0.508811 | 1.42E-36 |
| FOXO1 | PSPC1-AS2 | 0.508856 | 1.40E-36 |
| RB1 | AC008770.3 | 0.508871 | 1.39E-36 |
| PTEN | AC021851.1 | 0.508894 | 1.38E-36 |
| MAPK8 | AC011472.4 | 0.508926 | 1.36E-36 |
| ATG2B | AL391001.1 | 0.508928 | 1.36E-36 |
| FOXO3 | AP005131.7 | 0.508943 | 1.36E-36 |
| TSC1 | LENG8-AS1 | 0.508959 | 1.35E-36 |
| RPS6KB1 | AL049840.5 | 0.508976 | 1.34E-36 |
| ATG4B | PRKCZ-AS1 | 0.508979 | 1.34E-36 |
| MAPK8 | KIF26B-AS1 | 0.509031 | 1.31E-36 |
| MAPK8 | PWAR6 | 0.509053 | 1.30E-36 |
| MAPK8 | AC104695.3 | 0.509067 | 1.30E-36 |
| CFLAR | AC010226.1 | 0.50908 | 1.29E-36 |
| PIK3C3 | AC024075.1 | 0.509084 | 1.29E-36 |
| RPS6KB1 | AL158166.2 | 0.509092 | 1.28E-36 |
| NAF1 | AC008115.3 | 0.509093 | 1.28E-36 |
| CFLAR | AC096586.2 | 0.509096 | 1.28E-36 |
| GOPC | AL592148.3 | 0.509165 | 1.25E-36 |
| PTEN | AC073569.2 | 0.50919 | 1.24E-36 |
| FOXO1 | AC093388.1 | 0.509243 | 1.21E-36 |
| EIF2AK2 | AC007038.2 | 0.509249 | 1.21E-36 |
| ATG2B | AC007406.5 | 0.50926 | 1.21E-36 |
| EIF2AK2 | AC005021.1 | 0.509261 | 1.21E-36 |
| RB1CC1 | AC006270.1 | 0.509269 | 1.20E-36 |
| MAPK8 | AC087752.4 | 0.509311 | 1.18E-36 |
| CFLAR | AL049869.3 | 0.509316 | 1.18E-36 |
| RB1CC1 | PSPC1-AS2 | 0.509318 | 1.18E-36 |
| MAPK8 | ATP1A1-AS1 | 0.509321 | 1.18E-36 |
| KLHL24 | AC092611.2 | 0.509321 | 1.18E-36 |
| WDFY3 | CCDC18-AS1 | 0.509325 | 1.18E-36 |
| CFLAR | AC069023.1 | 0.509371 | 1.16E-36 |
| RB1CC1 | AL049552.1 | 0.509401 | 1.15E-36 |
| RPS6KB1 | AC078778.1 | 0.509493 | 1.11E-36 |
| ATG4B | DICER1-AS1 | 0.509498 | 1.11E-36 |
| TSC1 | AC141002.1 | 0.509501 | 1.10E-36 |
| EIF2AK2 | AC098851.1 | 0.509588 | 1.07E-36 |
| ATG12 | AGAP1-IT1 | 0.509635 | 1.05E-36 |
| RPS6KB1 | AC090948.3 | 0.509641 | 1.05E-36 |
| TSC1 | AC004908.2 | 0.509642 | 1.05E-36 |
| BIRC6 | AC025171.2 | 0.509666 | 1.04E-36 |
| MAPK8 | RAB30-AS1 | 0.509669 | 1.04E-36 |
| CFLAR | AP001160.4 | 0.509706 | 1.02E-36 |
| NAF1 | FAM13A-AS1 | 0.509709 | 1.02E-36 |
| NAF1 | ERVK13-1 | 0.50972 | 1.02E-36 |
| PIK3C3 | ANKRD10-IT1 | 0.50972 | 1.02E-36 |
| RPS6KB1 | AC096586.2 | 0.509733 | 1.01E-36 |
| CFLAR | AL109614.1 | 0.509797 | 9.90E-37 |
| MAPK8 | AP001625.2 | 0.509799 | 9.89E-37 |
| RB1 | AC022973.3 | 0.509823 | 9.81E-37 |
| RPS6KB1 | ITCH-IT1 | 0.509831 | 9.78E-37 |
| RB1 | AL133243.2 | 0.509847 | 9.72E-37 |
| KLHL24 | AC078846.1 | 0.509858 | 9.68E-37 |
| RB1CC1 | AC093788.1 | 0.509859 | 9.68E-37 |
| KLHL24 | AC074032.1 | 0.509863 | 9.66E-37 |
| FOXO3 | AC024075.1 | 0.509903 | 9.52E-37 |
| FOXO3 | AL606834.2 | 0.509908 | 9.50E-37 |
| FOXO1 | AC004918.3 | 0.509935 | 9.41E-37 |
| RB1CC1 | AL513550.1 | 0.510027 | 9.10E-37 |
| ATG12 | AL355488.1 | 0.510041 | 9.05E-37 |
| ATG2B | AC107027.3 | 0.510101 | 8.85E-37 |
| EIF2AK2 | AC063965.1 | 0.510115 | 8.80E-37 |
| NAF1 | AC066613.1 | 0.510122 | 8.78E-37 |
| TSC1 | AC008770.3 | 0.510123 | 8.78E-37 |
| KLHL24 | AC096586.2 | 0.510151 | 8.69E-37 |
| RB1 | AC087276.1 | 0.510167 | 8.64E-37 |
| CAPN10 | AC073335.2 | 0.510193 | 8.55E-37 |
| MAPK8 | RAP2C-AS1 | 0.510212 | 8.50E-37 |
| ATG7 | AF117829.1 | 0.510234 | 8.42E-37 |
| CFLAR | AC138393.3 | 0.510255 | 8.36E-37 |
| ATG12 | MAGI2-AS3 | 0.510257 | 8.35E-37 |
| TSC1 | AC022150.4 | 0.510258 | 8.35E-37 |
| ATG2B | AC133644.2 | 0.510291 | 8.25E-37 |
| ATG2B | AL357060.1 | 0.510384 | 7.97E-37 |
| TSC1 | AL159169.2 | 0.510393 | 7.95E-37 |
| KLHL24 | AC000123.1 | 0.510404 | 7.91E-37 |
| PIK3C3 | AC090425.2 | 0.51041 | 7.89E-37 |
| NAF1 | AC090948.3 | 0.510421 | 7.86E-37 |
| RAB24 | U62317.2 | 0.510476 | 7.70E-37 |
| IL24 | LINC00926 | 0.510479 | 7.70E-37 |
| ATG4B | AP006621.3 | 0.510531 | 7.55E-37 |
| TSC1 | AL354989.1 | 0.510561 | 7.47E-37 |
| NAF1 | LINC-PINT | 0.510622 | 7.30E-37 |
| KLHL24 | AC073534.1 | 0.510622 | 7.30E-37 |
| CFLAR | AC012368.1 | 0.510653 | 7.22E-37 |
| KLHL24 | AL662844.3 | 0.510713 | 7.06E-37 |
| KLHL24 | RFX3-AS1 | 0.510721 | 7.04E-37 |
| CFLAR | MCCC1-AS1 | 0.510738 | 6.99E-37 |
| ATG16L2 | AC245140.2 | 0.510903 | 6.58E-37 |
| NAF1 | AL731566.1 | 0.510914 | 6.55E-37 |
| ATG4B | AC084018.1 | 0.510927 | 6.52E-37 |
| KLHL24 | PAXBP1-AS1 | 0.510933 | 6.51E-37 |
| PIK3C3 | MACC1-AS1 | 0.510962 | 6.44E-37 |
| KLHL24 | AL354733.3 | 0.510993 | 6.36E-37 |
| KLHL24 | AC004223.3 | 0.51103 | 6.27E-37 |
| EIF2AK2 | AL731566.1 | 0.511042 | 6.25E-37 |
| CFLAR | AC130650.2 | 0.511068 | 6.19E-37 |
| NAF1 | LINC00513 | 0.511101 | 6.11E-37 |
| FOXO1 | AC068790.5 | 0.51112 | 6.07E-37 |
| MAPK8 | AC074032.1 | 0.511162 | 5.97E-37 |
| FOXO1 | SAP30L-AS1 | 0.511171 | 5.96E-37 |
| BIRC6 | AC010542.5 | 0.511198 | 5.90E-37 |
| NAF1 | AP000873.2 | 0.511234 | 5.82E-37 |
| PIK3C3 | AC096741.1 | 0.511267 | 5.75E-37 |
| RPS6KB1 | AC130650.2 | 0.511311 | 5.65E-37 |
| BCL2 | FOXN3-AS1 | 0.511313 | 5.65E-37 |
| PTEN | AC004908.3 | 0.511412 | 5.45E-37 |
| EIF2AK2 | AC037487.2 | 0.511425 | 5.42E-37 |
| PIK3C3 | SAP30L-AS1 | 0.511434 | 5.40E-37 |
| RPS6KB1 | AC131971.1 | 0.511439 | 5.39E-37 |
| KLHL24 | CD44-AS1 | 0.51146 | 5.35E-37 |
| ATG4B | MELTF-AS1 | 0.511461 | 5.35E-37 |
| ATG2B | MAGI2-AS3 | 0.511557 | 5.16E-37 |
| EIF2AK2 | RAP2C-AS1 | 0.511559 | 5.16E-37 |
| FOXO1 | AC005838.2 | 0.511582 | 5.11E-37 |
| FOXO1 | AC093788.1 | 0.511622 | 5.04E-37 |
| ATG2B | AC060780.1 | 0.511623 | 5.04E-37 |
| PIK3C3 | AC083843.2 | 0.511624 | 5.03E-37 |
| LAMP1 | AC020558.2 | 0.511683 | 4.93E-37 |
| TSC1 | AC018638.7 | 0.511688 | 4.92E-37 |
| ATG2B | AC124319.1 | 0.511702 | 4.89E-37 |
| GOPC | CR936218.1 | 0.511754 | 4.80E-37 |
| RB1CC1 | DNM3OS | 0.511755 | 4.79E-37 |
| CFLAR | AC024075.1 | 0.511763 | 4.78E-37 |
| EIF2AK2 | ADNP-AS1 | 0.511769 | 4.77E-37 |
| ATG16L2 | AL031717.1 | 0.511781 | 4.75E-37 |
| TSC1 | AC004067.1 | 0.511827 | 4.67E-37 |
| NAF1 | AC090425.2 | 0.511859 | 4.61E-37 |
| SPNS1 | AL031714.1 | 0.511859 | 4.61E-37 |
| CFLAR | AC005046.1 | 0.511864 | 4.61E-37 |
| ATG12 | AC078883.1 | 0.511865 | 4.60E-37 |
| EIF2AK2 | AC087284.1 | 0.511875 | 4.59E-37 |
| NLRC4 | AC145098.1 | 0.511911 | 4.53E-37 |
| ATG2B | AL021878.2 | 0.51202 | 4.35E-37 |
| EIF2AK2 | AC018690.1 | 0.51202 | 4.35E-37 |
| FOXO3 | NORAD | 0.512032 | 4.33E-37 |
| ATG12 | AC020915.3 | 0.51206 | 4.28E-37 |
| PIK3C3 | LINC01578 | 0.51207 | 4.27E-37 |
| CFLAR | OSMR-AS1 | 0.512111 | 4.20E-37 |
| GOPC | AC005070.3 | 0.512126 | 4.18E-37 |
| KLHL24 | AL132780.1 | 0.512127 | 4.18E-37 |
| CFLAR | AL357060.1 | 0.512144 | 4.15E-37 |
| ATG12 | AL606834.2 | 0.512208 | 4.05E-37 |
| ATG7 | AC037198.2 | 0.51224 | 4.00E-37 |
| KLHL24 | AC004918.3 | 0.512257 | 3.98E-37 |
| KLHL24 | NEAT1 | 0.512297 | 3.92E-37 |
| ATG16L2 | AC009065.8 | 0.512302 | 3.91E-37 |
| IFNG | PCED1B-AS1 | 0.512366 | 3.82E-37 |
| ATG16L2 | AC127502.2 | 0.512437 | 3.72E-37 |
| NAF1 | MBNL1-AS1 | 0.512438 | 3.72E-37 |
| MAPK8 | AC024075.3 | 0.512459 | 3.69E-37 |
| RB1CC1 | AC100830.2 | 0.512528 | 3.60E-37 |
| RB1CC1 | AC099343.2 | 0.512535 | 3.59E-37 |
| RPS6KB1 | AC007038.1 | 0.512565 | 3.55E-37 |
| PTEN | AC016957.2 | 0.512579 | 3.53E-37 |
| KLHL24 | AC090948.3 | 0.512604 | 3.50E-37 |
| RPS6KB1 | ARHGEF38-IT1 | 0.51262 | 3.48E-37 |
| RB1 | AC005838.2 | 0.512649 | 3.44E-37 |
| PIK3C3 | AC124319.2 | 0.512655 | 3.43E-37 |
| CFLAR | AL136115.2 | 0.512656 | 3.43E-37 |
| EIF2AK2 | AL049840.2 | 0.51266 | 3.42E-37 |
| NAF1 | AL137779.2 | 0.512664 | 3.42E-37 |
| PIK3C3 | FAM13A-AS1 | 0.512669 | 3.41E-37 |
| FOXO1 | FAM13A-AS1 | 0.512672 | 3.41E-37 |
| TSC1 | PAXBP1-AS1 | 0.512677 | 3.40E-37 |
| RB1 | FTX | 0.512677 | 3.40E-37 |
| NAF1 | AC026202.2 | 0.512701 | 3.37E-37 |
| PIK3C3 | LINC01355 | 0.512706 | 3.37E-37 |
| CFLAR | MCM3AP-AS1 | 0.512723 | 3.35E-37 |
| GOPC | AC087276.1 | 0.51274 | 3.32E-37 |
| RPS6KB1 | AC000123.1 | 0.512752 | 3.31E-37 |
| RB1CC1 | NUTM2B-AS1 | 0.512771 | 3.29E-37 |
| BIRC6 | MIR155HG | 0.512786 | 3.27E-37 |
| PIK3C3 | AP000692.1 | 0.512795 | 3.26E-37 |
| NAF1 | AL157786.1 | 0.512799 | 3.25E-37 |
| KLHL24 | ALMS1-IT1 | 0.512805 | 3.24E-37 |
| ATG16L2 | MMP25-AS1 | 0.512834 | 3.21E-37 |
| RPS6KB1 | AL133330.1 | 0.512841 | 3.20E-37 |
| TSC1 | AC093788.1 | 0.512854 | 3.19E-37 |
| MAPK8 | AC004477.3 | 0.512925 | 3.10E-37 |
| CFLAR | AL158166.2 | 0.512942 | 3.08E-37 |
| FOXO1 | DNM3OS | 0.512943 | 3.08E-37 |
| ATG12 | AL021707.8 | 0.513019 | 3.00E-37 |
| EIF2AK2 | AC079684.1 | 0.513019 | 3.00E-37 |
| BIRC6 | AC005034.3 | 0.513024 | 2.99E-37 |
| KLHL24 | AC138956.1 | 0.513066 | 2.94E-37 |
| EIF2AK2 | AP001178.2 | 0.513077 | 2.93E-37 |
| KIF5B | AC073046.1 | 0.513105 | 2.90E-37 |
| PTEN | AC010542.5 | 0.513108 | 2.90E-37 |
| CFLAR | AC068790.5 | 0.513108 | 2.90E-37 |
| MAPK8 | AL359715.3 | 0.51311 | 2.90E-37 |
| CFLAR | LINC00630 | 0.513164 | 2.84E-37 |
| NAF1 | AC004884.2 | 0.513167 | 2.84E-37 |
| ATG16L2 | AC006547.1 | 0.513174 | 2.83E-37 |
| ATG12 | AC016394.1 | 0.513185 | 2.82E-37 |
| FOXO1 | CD44-AS1 | 0.513224 | 2.78E-37 |
| RB1CC1 | AL513008.1 | 0.513236 | 2.76E-37 |
| RPS6KB1 | AC068790.2 | 0.513248 | 2.75E-37 |
| ATG16L2 | AC008735.4 | 0.51329 | 2.71E-37 |
| TSC1 | Z82243.1 | 0.513308 | 2.69E-37 |
| CFLAR | AC245884.8 | 0.513335 | 2.66E-37 |
| FOXO1 | MAGI2-AS3 | 0.513339 | 2.66E-37 |
| GOPC | AC138932.5 | 0.51335 | 2.65E-37 |
| CFLAR | AC092801.1 | 0.513368 | 2.63E-37 |
| WDFY3 | LINC01655 | 0.513405 | 2.59E-37 |
| PIK3C3 | AC012467.1 | 0.513476 | 2.53E-37 |
| WDFY3 | AC087752.3 | 0.513505 | 2.50E-37 |
| MAPK8 | AC008906.1 | 0.513526 | 2.48E-37 |
| EIF2AK2 | AC011477.2 | 0.513527 | 2.48E-37 |
| EIF2AK2 | AL031775.2 | 0.513585 | 2.43E-37 |
| BIRC6 | AC022784.5 | 0.513587 | 2.42E-37 |
| PIK3C3 | PAXBP1-AS1 | 0.513594 | 2.42E-37 |
| RB1CC1 | AL031775.2 | 0.513604 | 2.41E-37 |
| CXCR4 | AC243960.1 | 0.51364 | 2.38E-37 |
| NAF1 | AC008537.2 | 0.513667 | 2.35E-37 |
| CFLAR | AL590723.1 | 0.513685 | 2.34E-37 |
| ATG12 | ZNF460-AS1 | 0.5137 | 2.32E-37 |
| ATG7 | RRN3P2 | 0.513735 | 2.29E-37 |
| EIF2AK2 | AC096586.2 | 0.513801 | 2.24E-37 |
| ATG4B | AL118558.4 | 0.513825 | 2.22E-37 |
| RB1CC1 | AC012181.2 | 0.513839 | 2.21E-37 |
| TSC1 | ZNF460-AS1 | 0.513845 | 2.20E-37 |
| KLHL24 | ERVK13-1 | 0.513882 | 2.17E-37 |
| MAPK8 | AL031670.1 | 0.513889 | 2.16E-37 |
| ATG16L2 | AC092119.2 | 0.513892 | 2.16E-37 |
| ATG4B | AC009065.8 | 0.513899 | 2.16E-37 |
| FOXO3 | AC234775.3 | 0.513932 | 2.13E-37 |
| NAF1 | GAS5-AS1 | 0.513939 | 2.12E-37 |
| GOPC | AC108010.1 | 0.513979 | 2.09E-37 |
| MAPK8 | AC139887.2 | 0.513985 | 2.09E-37 |
| GOPC | AL138963.1 | 0.514001 | 2.08E-37 |
| KLHL24 | DNM3OS | 0.514027 | 2.06E-37 |
| PIK3C3 | AC006270.1 | 0.514159 | 1.96E-37 |
| FOXO1 | MIATNB | 0.514164 | 1.95E-37 |
| CFLAR | Z98884.2 | 0.514202 | 1.93E-37 |
| MAPK8 | AC108010.1 | 0.514204 | 1.92E-37 |
| RPS6KB1 | AC010761.3 | 0.514221 | 1.91E-37 |
| TSC1 | AC021078.1 | 0.514232 | 1.90E-37 |
| FOXO1 | AP001178.2 | 0.514237 | 1.90E-37 |
| RPS6KB1 | SCARNA9 | 0.514251 | 1.89E-37 |
| RB1 | AL138963.1 | 0.514333 | 1.83E-37 |
| PTEN | AC080013.4 | 0.514347 | 1.82E-37 |
| PTEN | AC008969.1 | 0.514353 | 1.82E-37 |
| ATG2B | AC024060.1 | 0.51436 | 1.81E-37 |
| NAF1 | LINC00641 | 0.514391 | 1.79E-37 |
| KLHL24 | AF117829.1 | 0.514393 | 1.79E-37 |
| RPS6KB1 | AC007038.2 | 0.514415 | 1.78E-37 |
| PTEN | ZKSCAN2-DT | 0.51444 | 1.76E-37 |
| RB1CC1 | AL022067.1 | 0.514471 | 1.74E-37 |
| CFLAR | MIATNB | 0.514487 | 1.73E-37 |
| PTEN | SNHG26 | 0.514499 | 1.72E-37 |
| RB1CC1 | LINC01355 | 0.514518 | 1.71E-37 |
| CFLAR | AC011939.2 | 0.514532 | 1.70E-37 |
| CFLAR | AL121772.3 | 0.514533 | 1.70E-37 |
| CFLAR | AC087286.1 | 0.51455 | 1.69E-37 |
| PTEN | LINC01389 | 0.51455 | 1.69E-37 |
| RB1CC1 | AL359076.1 | 0.514559 | 1.68E-37 |
| CFLAR | AC016831.4 | 0.514603 | 1.66E-37 |
| CFLAR | AC025171.3 | 0.514609 | 1.65E-37 |
| TSC1 | AL031717.1 | 0.514611 | 1.65E-37 |
| PIK3C3 | AC104695.3 | 0.514645 | 1.63E-37 |
| NAF1 | MACC1-AS1 | 0.514766 | 1.56E-37 |
| WDFY3 | HM13-IT1 | 0.514768 | 1.56E-37 |
| GOPC | AC004492.1 | 0.514794 | 1.54E-37 |
| CFLAR | AP001381.1 | 0.514814 | 1.53E-37 |
| RPS6KB1 | AP002336.2 | 0.514816 | 1.53E-37 |
| CCR2 | AC083949.1 | 0.514817 | 1.53E-37 |
| CFLAR | AC004908.2 | 0.514841 | 1.51E-37 |
| RB1CC1 | AC093799.1 | 0.514845 | 1.51E-37 |
| ATG12 | AC005479.2 | 0.514855 | 1.51E-37 |
| GOPC | LINC00630 | 0.514909 | 1.48E-37 |
| CFLAR | LINC00216 | 0.514944 | 1.46E-37 |
| NAF1 | CD44-AS1 | 0.514972 | 1.44E-37 |
| CFLAR | RAP2C-AS1 | 0.514978 | 1.44E-37 |
| KLHL24 | FAM13A-AS1 | 0.514986 | 1.43E-37 |
| GOPC | AC234775.3 | 0.515009 | 1.42E-37 |
| BIRC6 | AL133371.2 | 0.515029 | 1.41E-37 |
| KLHL24 | AC005838.2 | 0.515033 | 1.41E-37 |
| RB1 | AC004832.5 | 0.515091 | 1.38E-37 |
| ATG4B | AC074212.1 | 0.515105 | 1.37E-37 |
| KLHL24 | AC026355.1 | 0.515106 | 1.37E-37 |
| EIF2AK2 | AL133342.1 | 0.515138 | 1.36E-37 |
| KLHL24 | AC027277.2 | 0.515176 | 1.34E-37 |
| TSC1 | SCAANT1 | 0.515223 | 1.31E-37 |
| PTEN | AL158212.3 | 0.515248 | 1.30E-37 |
| RPS6KB1 | AC037487.2 | 0.515267 | 1.29E-37 |
| PIK3C3 | AC006017.1 | 0.515309 | 1.27E-37 |
| EIF2AK2 | AC022400.5 | 0.515318 | 1.27E-37 |
| EIF2AK2 | AL592148.3 | 0.515321 | 1.26E-37 |
| RB1 | AC007878.1 | 0.515381 | 1.24E-37 |
| CFLAR | NARF-IT1 | 0.51539 | 1.23E-37 |
| RB1 | AC005070.3 | 0.515402 | 1.23E-37 |
| FOXO1 | AC009032.1 | 0.515435 | 1.21E-37 |
| ATG12 | AC024060.1 | 0.51547 | 1.20E-37 |
| ATG16L2 | AC103691.1 | 0.515492 | 1.19E-37 |
| ATG2B | AL139289.1 | 0.515525 | 1.17E-37 |
| RPS6KB1 | AC022973.3 | 0.515554 | 1.16E-37 |
| NAF1 | PSPC1-AS2 | 0.515586 | 1.15E-37 |
| NAF1 | AL133330.1 | 0.515594 | 1.14E-37 |
| TSC1 | PSPC1-AS2 | 0.515729 | 1.08E-37 |
| WDFY3 | AC022784.5 | 0.515756 | 1.07E-37 |
| ATG2B | AC008669.1 | 0.51576 | 1.07E-37 |
| KLHL24 | AC002128.1 | 0.515807 | 1.05E-37 |
| PIK3C3 | AC066613.1 | 0.515834 | 1.04E-37 |
| FOXO1 | AC013403.2 | 0.515862 | 1.03E-37 |
| PIK3C3 | AC004223.3 | 0.515898 | 1.02E-37 |
| RB1CC1 | AC026470.2 | 0.515903 | 1.02E-37 |
| WDFY3 | HMGN3-AS1 | 0.516018 | 9.73E-38 |
| MAPK8 | AL513008.1 | 0.516032 | 9.68E-38 |
| CFLAR | AC011477.2 | 0.516036 | 9.67E-38 |
| PIK3C3 | AC008124.1 | 0.516046 | 9.63E-38 |
| WDFY3 | AC120053.1 | 0.516088 | 9.48E-38 |
| HGS | AC132872.3 | 0.516097 | 9.45E-38 |
| TSC1 | AL513218.1 | 0.51612 | 9.36E-38 |
| PIK3C3 | AL021578.1 | 0.516129 | 9.33E-38 |
| TSC1 | AL022328.1 | 0.516132 | 9.32E-38 |
| EIF2AK2 | ANKRD10-IT1 | 0.516158 | 9.23E-38 |
| KLHL24 | Z98884.2 | 0.516167 | 9.20E-38 |
| EIF2AK2 | SNHG26 | 0.516188 | 9.13E-38 |
| KLHL24 | AC120349.1 | 0.516213 | 9.04E-38 |
| ATG7 | AL357060.1 | 0.516247 | 8.93E-38 |
| CAPN10 | AL022328.3 | 0.516303 | 8.74E-38 |
| WDFY3 | SH3BP5-AS1 | 0.516336 | 8.63E-38 |
| ATG2B | AL136295.6 | 0.516361 | 8.55E-38 |
| RPS6KB1 | AL645568.1 | 0.516374 | 8.51E-38 |
| ATG2B | AL139041.1 | 0.516376 | 8.50E-38 |
| KLHL24 | EP300-AS1 | 0.516376 | 8.50E-38 |
| CFLAR | AC004067.1 | 0.516438 | 8.31E-38 |
| ATG12 | AC025287.3 | 0.516447 | 8.28E-38 |
| RB1 | AC011939.2 | 0.516451 | 8.27E-38 |
| CFLAR | AC022211.1 | 0.516454 | 8.26E-38 |
| RPS6KB1 | ADAMTSL4-AS1 | 0.516465 | 8.22E-38 |
| NAF1 | AC093388.1 | 0.516488 | 8.15E-38 |
| ATG2B | XIST | 0.516499 | 8.12E-38 |
| CFLAR | PSPC1-AS2 | 0.516513 | 8.07E-38 |
| TSC1 | AC011477.2 | 0.516568 | 7.91E-38 |
| CFLAR | AC058791.1 | 0.516619 | 7.76E-38 |
| PIK3C3 | STARD4-AS1 | 0.516629 | 7.73E-38 |
| RPS6KB1 | AL513365.2 | 0.516645 | 7.68E-38 |
| EIF2AK2 | AL021578.1 | 0.516646 | 7.68E-38 |
| KLHL24 | NORAD | 0.516661 | 7.63E-38 |
| PTEN | GABPB1-AS1 | 0.516675 | 7.60E-38 |
| KLHL24 | AC253576.2 | 0.516712 | 7.49E-38 |
| CFLAR | AL606834.2 | 0.516754 | 7.37E-38 |
| GOPC | HCG18 | 0.51676 | 7.35E-38 |
| CFLAR | AL157392.4 | 0.516797 | 7.25E-38 |
| ATG16L2 | AP006621.3 | 0.51681 | 7.22E-38 |
| RPS6KB1 | AC002553.2 | 0.516875 | 7.04E-38 |
| WDFY3 | DUBR | 0.516889 | 7.01E-38 |
| PIK3C3 | AC004918.3 | 0.516918 | 6.93E-38 |
| KLHL24 | LINC00852 | 0.516928 | 6.90E-38 |
| EIF2AK2 | AL513008.1 | 0.51695 | 6.85E-38 |
| WDFY3 | AL021878.2 | 0.516952 | 6.84E-38 |
| RB1CC1 | AP000866.6 | 0.516965 | 6.81E-38 |
| RPS6KB1 | AC011815.1 | 0.516969 | 6.80E-38 |
| PIK3R4 | AC107027.3 | 0.51699 | 6.74E-38 |
| CFLAR | AC005070.3 | 0.516998 | 6.72E-38 |
| RB1 | AC007684.1 | 0.517039 | 6.62E-38 |
| RB1CC1 | AL592148.3 | 0.51706 | 6.57E-38 |
| RPS6KB1 | AC022173.1 | 0.517061 | 6.56E-38 |
| WDFY3 | GK-IT1 | 0.51707 | 6.54E-38 |
| EIF2AK2 | AC005856.1 | 0.517187 | 6.26E-38 |
| NAF1 | AC027277.2 | 0.517315 | 5.96E-38 |
| EIF2AK2 | AP000873.2 | 0.517337 | 5.91E-38 |
| ATG4B | AL031709.1 | 0.517339 | 5.91E-38 |
| ATG4B | AP001453.3 | 0.517345 | 5.90E-38 |
| PIK3C3 | AC007546.1 | 0.517376 | 5.83E-38 |
| ATG2B | AC068152.1 | 0.517439 | 5.69E-38 |
| PIK3C3 | AL157402.2 | 0.517474 | 5.61E-38 |
| RB1CC1 | AC078846.1 | 0.517484 | 5.59E-38 |
| CFLAR | AC022150.4 | 0.517502 | 5.56E-38 |
| PIK3C3 | AC093788.1 | 0.517565 | 5.43E-38 |
| ATG12 | AL596325.2 | 0.517569 | 5.42E-38 |
| MAPK8 | AP001469.2 | 0.517579 | 5.40E-38 |
| GOPC | AC007038.1 | 0.517621 | 5.31E-38 |
| ATG12 | AL157786.1 | 0.517628 | 5.30E-38 |
| EIF2AK2 | AC090948.3 | 0.51764 | 5.27E-38 |
| WDFY3 | AC244517.7 | 0.517688 | 5.18E-38 |
| ULK3 | AC069281.2 | 0.517798 | 4.97E-38 |
| ATG12 | AL031666.1 | 0.517867 | 4.84E-38 |
| EIF2AK2 | AC092611.2 | 0.517883 | 4.81E-38 |
| ATG12 | AC007319.1 | 0.517929 | 4.73E-38 |
| RPS6KB1 | AP002907.1 | 0.517956 | 4.68E-38 |
| PIK3C3 | AP001432.1 | 0.517961 | 4.67E-38 |
| ATG12 | AC083843.2 | 0.517963 | 4.67E-38 |
| RPS6KB1 | LINC00216 | 0.518024 | 4.56E-38 |
| RB1CC1 | AC007014.2 | 0.518081 | 4.46E-38 |
| RPS6KB1 | AC012557.1 | 0.518089 | 4.45E-38 |
| KLHL24 | AL031716.1 | 0.518122 | 4.39E-38 |
| PIK3C3 | AP002907.1 | 0.518125 | 4.39E-38 |
| CFLAR | AC073655.2 | 0.518137 | 4.37E-38 |
| ATG12 | AC078846.1 | 0.51817 | 4.31E-38 |
| SPNS1 | AC084018.1 | 0.518247 | 4.19E-38 |
| ATG2B | ALMS1-IT1 | 0.518249 | 4.19E-38 |
| RPS6KB1 | AF129075.1 | 0.518263 | 4.16E-38 |
| CFLAR | ITCH-IT1 | 0.51829 | 4.12E-38 |
| GOPC | AL049552.1 | 0.518298 | 4.11E-38 |
| NAF1 | AC130650.2 | 0.518316 | 4.08E-38 |
| EIF2AK2 | AC007991.2 | 0.518369 | 4.00E-38 |
| EIF2AK2 | AL365277.1 | 0.518376 | 3.99E-38 |
| PTEN | ZNF460-AS1 | 0.518389 | 3.97E-38 |
| ATG2B | RBMS3-AS3 | 0.518421 | 3.92E-38 |
| TSC1 | AL080317.1 | 0.518444 | 3.89E-38 |
| ATG2B | EBLN3P | 0.518448 | 3.88E-38 |
| EIF2AK2 | A2M-AS1 | 0.518448 | 3.88E-38 |
| CFLAR | AL450263.1 | 0.518463 | 3.86E-38 |
| NAF1 | AC005021.1 | 0.518469 | 3.85E-38 |
| ULK3 | AC073335.2 | 0.518471 | 3.85E-38 |
| RPS6KB1 | AC007684.1 | 0.51848 | 3.83E-38 |
| GOPC | AC025917.1 | 0.518493 | 3.82E-38 |
| KLHL24 | AC012181.2 | 0.518528 | 3.77E-38 |
| NAF1 | AC012467.1 | 0.518639 | 3.61E-38 |
| ATG16L2 | RUSC1-AS1 | 0.518639 | 3.61E-38 |
| ATG12 | AC020571.1 | 0.518662 | 3.58E-38 |
| CFLAR | EP300-AS1 | 0.518733 | 3.48E-38 |
| ATG2B | ABALON | 0.518777 | 3.43E-38 |
| BIRC6 | AC008735.4 | 0.518781 | 3.42E-38 |
| RAB24 | AL022328.2 | 0.518816 | 3.37E-38 |
| RPS6KB1 | LINC01355 | 0.518831 | 3.36E-38 |
| RPS6KB1 | Z82243.1 | 0.51887 | 3.31E-38 |
| WDFY3 | ZNF32-AS2 | 0.518878 | 3.30E-38 |
| KLHL24 | MCM3AP-AS1 | 0.51888 | 3.29E-38 |
| CXCR4 | TRBV11-2 | 0.518891 | 3.28E-38 |
| ATG4B | AL031600.1 | 0.518953 | 3.20E-38 |
| BIRC6 | AC024060.1 | 0.51898 | 3.17E-38 |
| MAPK8 | AC007938.3 | 0.519013 | 3.13E-38 |
| MAP2K7 | PTOV1-AS2 | 0.519016 | 3.13E-38 |
| NAF1 | AC124312.4 | 0.51902 | 3.12E-38 |
| CFLAR | AC008735.4 | 0.519028 | 3.11E-38 |
| FOXO1 | AC012557.1 | 0.519039 | 3.10E-38 |
| ATG2B | HM13-IT1 | 0.519045 | 3.09E-38 |
| PTEN | NFYC-AS1 | 0.519049 | 3.09E-38 |
| FOXO1 | AC026356.1 | 0.519082 | 3.05E-38 |
| ATG2B | ATP13A4-AS1 | 0.519139 | 2.98E-38 |
| PIK3C3 | AC084824.4 | 0.519186 | 2.93E-38 |
| ATG4B | AP001160.1 | 0.519194 | 2.92E-38 |
| GOPC | AC053513.1 | 0.519197 | 2.92E-38 |
| MAPK8 | AC037487.2 | 0.519202 | 2.91E-38 |
| PTEN | AC011815.1 | 0.519205 | 2.91E-38 |
| NAF1 | AC096741.1 | 0.519207 | 2.91E-38 |
| RB1CC1 | AC084824.4 | 0.519214 | 2.90E-38 |
| PTEN | AC079907.1 | 0.519241 | 2.87E-38 |
| ATG12 | AC005046.1 | 0.51925 | 2.86E-38 |
| KLHL24 | AC006378.1 | 0.519252 | 2.86E-38 |
| CFLAR | AC010542.5 | 0.519257 | 2.85E-38 |
| RB1CC1 | AL157392.3 | 0.519283 | 2.82E-38 |
| BIRC6 | AL157871.2 | 0.519289 | 2.82E-38 |
| ATG12 | AL122035.1 | 0.519295 | 2.81E-38 |
| RB1 | LINC00216 | 0.519299 | 2.81E-38 |
| GOPC | AC010761.3 | 0.519325 | 2.78E-38 |
| ATG12 | AL354733.3 | 0.519354 | 2.75E-38 |
| RPS6KB1 | AC006059.1 | 0.519377 | 2.73E-38 |
| ATG16L2 | AC087481.3 | 0.519417 | 2.68E-38 |
| PIK3C3 | AC037487.2 | 0.51942 | 2.68E-38 |
| FOXO1 | AC009090.3 | 0.519458 | 2.64E-38 |
| KLHL24 | AL137779.2 | 0.519512 | 2.59E-38 |
| GOPC | SOS1-IT1 | 0.519563 | 2.54E-38 |
| TSC1 | AC024075.2 | 0.519596 | 2.51E-38 |
| RB1CC1 | AP000873.2 | 0.51961 | 2.49E-38 |
| ATG12 | AC024075.3 | 0.519647 | 2.46E-38 |
| PTEN | RPS6KA2-IT1 | 0.519657 | 2.45E-38 |
| WDFY3 | AL080317.1 | 0.519673 | 2.43E-38 |
| ATG4B | AC009065.4 | 0.519692 | 2.42E-38 |
| RPS6KB1 | AC053513.1 | 0.519757 | 2.36E-38 |
| TSC1 | FMR1-IT1 | 0.519767 | 2.35E-38 |
| RPS6KB1 | AC020913.3 | 0.519776 | 2.34E-38 |
| KLHL24 | AL162724.1 | 0.51985 | 2.28E-38 |
| RB1CC1 | AL162724.2 | 0.519864 | 2.26E-38 |
| TSC1 | CR936218.1 | 0.519924 | 2.21E-38 |
| ATG12 | AL139041.1 | 0.519927 | 2.21E-38 |
| KLHL24 | AC087286.1 | 0.519928 | 2.21E-38 |
| RB1CC1 | NEAT1 | 0.519946 | 2.19E-38 |
| MAPK8 | AP001486.2 | 0.51996 | 2.18E-38 |
| GOPC | AC006059.1 | 0.520067 | 2.10E-38 |
| MAPK8 | AP001432.1 | 0.5201 | 2.07E-38 |
| CFLAR | AC005674.2 | 0.520115 | 2.06E-38 |
| ATG16L2 | AC233728.1 | 0.520145 | 2.03E-38 |
| PIK3C3 | AC116366.1 | 0.520188 | 2.00E-38 |
| RB1CC1 | ALG13-AS1 | 0.520204 | 1.99E-38 |
| PTEN | AC092611.2 | 0.520206 | 1.99E-38 |
| GOPC | AL513365.2 | 0.520206 | 1.99E-38 |
| MAPK8 | AGAP1-IT1 | 0.520229 | 1.97E-38 |
| MAPK8 | DLEU1 | 0.520252 | 1.95E-38 |
| FOXO1 | AL117336.2 | 0.520256 | 1.95E-38 |
| ATG16L2 | LINC01176 | 0.520304 | 1.91E-38 |
| ATG2B | AC079907.1 | 0.520322 | 1.90E-38 |
| ATG16L2 | AC010883.1 | 0.52033 | 1.90E-38 |
| ATG12 | AC105389.2 | 0.520335 | 1.89E-38 |
| ATG16L2 | AC108134.1 | 0.520343 | 1.89E-38 |
| KLHL24 | AL139407.1 | 0.520377 | 1.86E-38 |
| GOPC | ACAP2-IT1 | 0.520386 | 1.85E-38 |
| ATG12 | LINC01534 | 0.520445 | 1.81E-38 |
| PIK3C3 | AC020915.2 | 0.520446 | 1.81E-38 |
| ATG2B | AC109460.2 | 0.52051 | 1.77E-38 |
| MAPK8 | MACC1-AS1 | 0.520654 | 1.67E-38 |
| ATG12 | AC007566.1 | 0.52066 | 1.67E-38 |
| FOXO1 | AL513008.1 | 0.52067 | 1.66E-38 |
| PIK3C3 | AC004884.2 | 0.520684 | 1.65E-38 |
| EIF2AK2 | AC012467.1 | 0.520701 | 1.64E-38 |
| CFLAR | AC009318.3 | 0.520724 | 1.63E-38 |
| FOXO1 | LAMC1-AS1 | 0.520736 | 1.62E-38 |
| RB1CC1 | AC024075.3 | 0.520748 | 1.62E-38 |
| CFLAR | AC022173.1 | 0.520768 | 1.60E-38 |
| PTEN | AL359915.2 | 0.520773 | 1.60E-38 |
| EIF2AK2 | AC067852.3 | 0.520792 | 1.59E-38 |
| CFLAR | AC124283.3 | 0.520796 | 1.59E-38 |
| PIK3C3 | AL596325.2 | 0.520836 | 1.56E-38 |
| TSC1 | AC009120.3 | 0.520843 | 1.56E-38 |
| KLHL24 | AC138393.3 | 0.520854 | 1.55E-38 |
| MAPK8 | AL157402.2 | 0.520858 | 1.55E-38 |
| EIF2AK2 | PAXBP1-AS1 | 0.520871 | 1.54E-38 |
| KLHL24 | AC093788.1 | 0.520873 | 1.54E-38 |
| PIK3C3 | AC005632.2 | 0.520879 | 1.54E-38 |
| KLHL24 | AC007038.2 | 0.52088 | 1.54E-38 |
| BIRC6 | EBLN3P | 0.520892 | 1.53E-38 |
| TSC1 | AL731566.1 | 0.520935 | 1.50E-38 |
| KLHL24 | RHOA-IT1 | 0.520949 | 1.50E-38 |
| CAPN10 | AL021707.6 | 0.521029 | 1.45E-38 |
| FOXO1 | AC090181.2 | 0.521035 | 1.45E-38 |
| ATG2B | NUTM2A-AS1 | 0.521051 | 1.44E-38 |
| MAPK8 | AC006270.1 | 0.521068 | 1.43E-38 |
| EIF2AK2 | AP002336.2 | 0.521074 | 1.43E-38 |
| RPS6KB1 | AC005070.3 | 0.521078 | 1.42E-38 |
| RPS6KB1 | AC114760.2 | 0.521119 | 1.40E-38 |
| CFLAR | AC073651.1 | 0.521256 | 1.33E-38 |
| KLHL24 | AL645568.1 | 0.521262 | 1.33E-38 |
| MAPK8 | AC022211.1 | 0.521281 | 1.32E-38 |
| MAPK8 | AC125257.1 | 0.521306 | 1.30E-38 |
| MAPK8 | LINC01376 | 0.521317 | 1.30E-38 |
| EIF2AK2 | AC006017.1 | 0.521319 | 1.30E-38 |
| TSC1 | AL049840.1 | 0.521361 | 1.28E-38 |
| CFLAR | AC067817.2 | 0.521398 | 1.26E-38 |
| FOXO1 | AL133330.1 | 0.521406 | 1.26E-38 |
| NAF1 | AC007038.2 | 0.521434 | 1.24E-38 |
| MAPK8 | AC005034.5 | 0.521436 | 1.24E-38 |
| RPS6KB1 | AC006017.1 | 0.52144 | 1.24E-38 |
| ATG4B | AC233728.1 | 0.521477 | 1.22E-38 |
| RB1CC1 | AC002128.2 | 0.521529 | 1.20E-38 |
| ATG2B | AL359715.3 | 0.521548 | 1.19E-38 |
| MAPK8 | AC010186.3 | 0.521629 | 1.15E-38 |
| TSC1 | AL031670.1 | 0.52165 | 1.14E-38 |
| RB1CC1 | AC090181.2 | 0.521686 | 1.13E-38 |
| CFLAR | GMDS-DT | 0.521692 | 1.12E-38 |
| RB1CC1 | AC012170.2 | 0.521698 | 1.12E-38 |
| FOXO1 | AC073487.1 | 0.521699 | 1.12E-38 |
| RB1CC1 | AC021078.1 | 0.521708 | 1.12E-38 |
| NAF1 | AP001486.2 | 0.52171 | 1.12E-38 |
| PTEN | AL109761.1 | 0.521778 | 1.09E-38 |
| CFLAR | AP000873.2 | 0.521821 | 1.07E-38 |
| FOXO1 | AC005632.2 | 0.52184 | 1.06E-38 |
| CFLAR | AC099343.2 | 0.521848 | 1.06E-38 |
| RB1 | AC092801.1 | 0.521892 | 1.04E-38 |
| RPS6KB1 | AL139120.1 | 0.521915 | 1.03E-38 |
| PIK3C3 | AC012181.1 | 0.521939 | 1.02E-38 |
| PIK3C3 | AP000866.6 | 0.521966 | 1.01E-38 |
| RB1CC1 | AC007546.1 | 0.521994 | 1.00E-38 |
| ATG12 | AC027097.1 | 0.522014 | 9.94E-39 |
| RPS6KB1 | AL136320.1 | 0.522029 | 9.88E-39 |
| GOPC | AL049840.1 | 0.522047 | 9.81E-39 |
| KLHL24 | AL365277.1 | 0.522127 | 9.51E-39 |
| GOPC | AL133243.2 | 0.522196 | 9.27E-39 |
| TSC1 | AC008870.2 | 0.522254 | 9.06E-39 |
| PIK3C3 | AC124312.4 | 0.522307 | 8.88E-39 |
| RB1 | AL163051.2 | 0.522313 | 8.86E-39 |
| CFLAR | AC016590.2 | 0.522321 | 8.83E-39 |
| FOXO1 | AL391834.1 | 0.522341 | 8.76E-39 |
| MAPK8 | AC093799.1 | 0.522364 | 8.69E-39 |
| CFLAR | MAL2-AS1 | 0.522389 | 8.60E-39 |
| CASP1 | AC090559.1 | 0.522391 | 8.60E-39 |
| RPS6KB1 | AF178030.1 | 0.522406 | 8.55E-39 |
| CAPN10 | AC020558.2 | 0.522406 | 8.55E-39 |
| RB1CC1 | AL133342.1 | 0.522413 | 8.52E-39 |
| WDFY3 | AC068792.1 | 0.522416 | 8.52E-39 |
| ULK3 | AL022328.2 | 0.522468 | 8.35E-39 |
| EIF2AK2 | AC073651.1 | 0.522519 | 8.18E-39 |
| FOXO1 | AC004884.2 | 0.522532 | 8.14E-39 |
| GOPC | AC004918.3 | 0.522571 | 8.02E-39 |
| GOPC | AL078581.1 | 0.522589 | 7.96E-39 |
| EIF2AK2 | MIR29B2CHG | 0.522625 | 7.85E-39 |
| RPS6KB1 | AC091057.1 | 0.52267 | 7.72E-39 |
| ATG2B | AC098484.1 | 0.522686 | 7.67E-39 |
| ATG2B | SCAANT1 | 0.522689 | 7.66E-39 |
| TSC1 | AL049840.3 | 0.522717 | 7.58E-39 |
| MAPK8 | AP001160.4 | 0.52272 | 7.57E-39 |
| RB1 | AC022173.1 | 0.522724 | 7.56E-39 |
| PIK3C3 | AC027277.2 | 0.522745 | 7.50E-39 |
| MAPK8 | AC002044.1 | 0.52279 | 7.37E-39 |
| GOPC | AL049840.5 | 0.52289 | 7.09E-39 |
| FOXO1 | AC025171.3 | 0.522904 | 7.06E-39 |
| RB1CC1 | AL162724.1 | 0.522912 | 7.03E-39 |
| CFLAR | AC104695.3 | 0.522965 | 6.89E-39 |
| FOXO1 | Z82243.1 | 0.522975 | 6.86E-39 |
| EIF2AK2 | AC104695.3 | 0.522981 | 6.85E-39 |
| RPS6KB1 | AC026356.1 | 0.522983 | 6.84E-39 |
| BIRC6 | AC093110.1 | 0.522999 | 6.80E-39 |
| KLHL24 | AC007552.2 | 0.523036 | 6.70E-39 |
| KLHL24 | AC090198.1 | 0.523091 | 6.56E-39 |
| RB1CC1 | AC113139.1 | 0.523126 | 6.48E-39 |
| TSC1 | AL132780.1 | 0.52315 | 6.42E-39 |
| FOXO1 | AC002044.1 | 0.523167 | 6.37E-39 |
| CFLAR | AC012181.2 | 0.523206 | 6.28E-39 |
| EIF2AK2 | AC138393.3 | 0.523226 | 6.23E-39 |
| RPS6KB1 | LIMS1-AS1 | 0.52324 | 6.20E-39 |
| WDFY3 | UBE2Q1-AS1 | 0.523258 | 6.15E-39 |
| TSC1 | AC012467.2 | 0.523275 | 6.11E-39 |
| RPS6KB1 | ALG13-AS1 | 0.523285 | 6.09E-39 |
| RPS6KB1 | AC007216.3 | 0.523311 | 6.03E-39 |
| EIF2AK2 | AC027277.2 | 0.523342 | 5.96E-39 |
| PTEN | AC008035.1 | 0.523344 | 5.95E-39 |
| ATG2B | AC074033.1 | 0.523372 | 5.89E-39 |
| MAPK8 | AC005104.1 | 0.523377 | 5.88E-39 |
| PTEN | AC005674.2 | 0.523436 | 5.75E-39 |
| FOXO1 | AC010834.3 | 0.523458 | 5.70E-39 |
| FOXO1 | AF178030.1 | 0.523465 | 5.68E-39 |
| ATG2B | AC092611.2 | 0.523466 | 5.68E-39 |
| CTSB | SMIM25 | 0.523498 | 5.61E-39 |
| BIRC6 | AC009948.4 | 0.523503 | 5.60E-39 |
| RPS6KB1 | MCM3AP-AS1 | 0.52351 | 5.58E-39 |
| RB1CC1 | NORAD | 0.523531 | 5.54E-39 |
| RB1CC1 | AC022211.1 | 0.523538 | 5.52E-39 |
| NAF1 | AL049840.2 | 0.523546 | 5.51E-39 |
| EIF2AK2 | AC099343.2 | 0.523571 | 5.45E-39 |
| MAPK8 | AC004918.3 | 0.523591 | 5.41E-39 |
| CFLAR | NUTM2B-AS1 | 0.523596 | 5.40E-39 |
| PTEN | AL035409.1 | 0.523625 | 5.34E-39 |
| RPS6KB1 | AC097641.2 | 0.523631 | 5.33E-39 |
| TSC1 | AC011468.5 | 0.523648 | 5.29E-39 |
| MAPK8 | AL031775.2 | 0.523665 | 5.26E-39 |
| PIK3C3 | AL137779.2 | 0.523683 | 5.22E-39 |
| FOXO1 | AC093799.1 | 0.5237 | 5.19E-39 |
| EIF2AK2 | AL137779.2 | 0.523737 | 5.11E-39 |
| CFLAR | TMEM161B-AS1 | 0.523815 | 4.96E-39 |
| ATG16L2 | AC004918.1 | 0.523825 | 4.94E-39 |
| KLHL24 | AC026202.2 | 0.523835 | 4.93E-39 |
| PTEN | AC112722.1 | 0.523844 | 4.91E-39 |
| ATG12 | AC018809.2 | 0.523855 | 4.89E-39 |
| RPS6KB1 | AC022150.4 | 0.523859 | 4.88E-39 |
| FOXO1 | AL137779.2 | 0.523899 | 4.81E-39 |
| RAB24 | AL513320.1 | 0.523906 | 4.79E-39 |
| TSC1 | GABPB1-AS1 | 0.523913 | 4.78E-39 |
| TSC1 | NARF-IT1 | 0.523918 | 4.77E-39 |
| MAPK8 | PPP3CB-AS1 | 0.523924 | 4.76E-39 |
| ATG12 | AC007849.1 | 0.523936 | 4.74E-39 |
| TSC1 | AL021878.2 | 0.523947 | 4.72E-39 |
| RPS6KB1 | AC234775.3 | 0.52397 | 4.67E-39 |
| RPS6KB1 | AL133445.2 | 0.523989 | 4.64E-39 |
| KLHL24 | AC092953.2 | 0.523995 | 4.63E-39 |
| MAPK8 | AC083843.2 | 0.523999 | 4.62E-39 |
| EIF2AK2 | AC068790.3 | 0.524022 | 4.58E-39 |
| ATG16L2 | AL513218.1 | 0.524035 | 4.56E-39 |
| NAF1 | AC020913.3 | 0.524057 | 4.52E-39 |
| TSC1 | AC007038.2 | 0.524074 | 4.49E-39 |
| KLHL24 | KIF26B-AS1 | 0.524079 | 4.48E-39 |
| TSC1 | AL359921.1 | 0.524081 | 4.48E-39 |
| RB1CC1 | NDUFV2-AS1 | 0.524094 | 4.46E-39 |
| RB1 | AL049840.5 | 0.524118 | 4.41E-39 |
| RB1CC1 | AC009032.1 | 0.524125 | 4.40E-39 |
| ATG2B | AC020978.3 | 0.524167 | 4.33E-39 |
| EIF2AK2 | AC021078.1 | 0.524171 | 4.33E-39 |
| NAF1 | MAL2-AS1 | 0.524201 | 4.27E-39 |
| KLHL24 | NUTM2B-AS1 | 0.524246 | 4.20E-39 |
| NAF1 | AC037487.2 | 0.524352 | 4.03E-39 |
| NAF1 | AC253536.3 | 0.524355 | 4.03E-39 |
| ATG2B | AC090617.5 | 0.524372 | 4.00E-39 |
| FOXO1 | AC002550.2 | 0.524394 | 3.97E-39 |
| NAF1 | MIR29B2CHG | 0.524432 | 3.91E-39 |
| ATG2B | AC145423.3 | 0.524473 | 3.85E-39 |
| RB1 | DLEU2 | 0.524541 | 3.75E-39 |
| RB1 | AL139120.1 | 0.524544 | 3.74E-39 |
| NAF1 | RHOA-IT1 | 0.524549 | 3.73E-39 |
| BIRC6 | KCCAT333 | 0.524569 | 3.71E-39 |
| ATG12 | AP000240.1 | 0.524572 | 3.70E-39 |
| RB1CC1 | AC090425.2 | 0.524573 | 3.70E-39 |
| ATG4B | LINC00174 | 0.524628 | 3.62E-39 |
| CFLAR | AC108449.2 | 0.524634 | 3.61E-39 |
| RPS6KB1 | AC005632.2 | 0.524645 | 3.60E-39 |
| PTEN | TBILA | 0.524677 | 3.55E-39 |
| CFLAR | AC020915.2 | 0.524683 | 3.55E-39 |
| FOXO1 | AC012181.1 | 0.524689 | 3.54E-39 |
| PIK3C3 | AL049840.2 | 0.524708 | 3.51E-39 |
| NAF1 | DLEU1 | 0.52471 | 3.51E-39 |
| MAPK8 | AC078846.1 | 0.524755 | 3.45E-39 |
| ATG12 | AC002128.2 | 0.524757 | 3.45E-39 |
| KLHL24 | LINC00641 | 0.52488 | 3.29E-39 |
| ATG2B | AC022784.5 | 0.524907 | 3.25E-39 |
| TSC1 | AC145423.3 | 0.524918 | 3.24E-39 |
| EIF2AK2 | AC253576.2 | 0.524918 | 3.24E-39 |
| RB1CC1 | AP005131.7 | 0.52494 | 3.21E-39 |
| ATG12 | AL021707.7 | 0.524959 | 3.19E-39 |
| RPS6KB1 | AL359915.2 | 0.524972 | 3.17E-39 |
| PTEN | AC026368.1 | 0.524977 | 3.16E-39 |
| TSC1 | PPP3CB-AS1 | 0.524989 | 3.15E-39 |
| CFLAR | AC004223.3 | 0.524993 | 3.15E-39 |
| ITGA6 | AC245041.1 | 0.524998 | 3.14E-39 |
| PIK3C3 | AP005899.1 | 0.525 | 3.14E-39 |
| EIF2AK2 | AC007014.2 | 0.525049 | 3.08E-39 |
| ATG12 | HIF1A-AS2 | 0.525061 | 3.06E-39 |
| CFLAR | AL133243.2 | 0.525136 | 2.97E-39 |
| ATG4B | AC073842.2 | 0.525154 | 2.95E-39 |
| TSC1 | AL031775.2 | 0.525174 | 2.93E-39 |
| ATG16L2 | AL731567.1 | 0.525216 | 2.88E-39 |
| CFLAR | ACAP2-IT1 | 0.52525 | 2.85E-39 |
| ATG4B | AL354836.1 | 0.525258 | 2.84E-39 |
| PIK3C3 | AC130650.2 | 0.525267 | 2.83E-39 |
| KLHL24 | AC104695.3 | 0.525328 | 2.76E-39 |
| KLHL24 | AC124312.4 | 0.525404 | 2.68E-39 |
| EIF2AK2 | CD44-AS1 | 0.525409 | 2.68E-39 |
| BIRC6 | ZNF32-AS2 | 0.525432 | 2.65E-39 |
| NAF1 | AP000866.6 | 0.52544 | 2.64E-39 |
| BIRC6 | AL683813.1 | 0.525493 | 2.59E-39 |
| RPS6KB1 | AC005332.3 | 0.525568 | 2.51E-39 |
| KLHL24 | MIR29B2CHG | 0.525597 | 2.49E-39 |
| ATG12 | AC067817.2 | 0.52561 | 2.47E-39 |
| KLHL24 | AC068792.1 | 0.525645 | 2.44E-39 |
| FOXO3 | AC067817.2 | 0.525652 | 2.43E-39 |
| CFLAR | AC018926.3 | 0.525714 | 2.38E-39 |
| MAPK8 | CFLAR-AS1 | 0.525738 | 2.35E-39 |
| MAPK8 | AC002064.2 | 0.525758 | 2.34E-39 |
| MAPK8 | LINC00641 | 0.525785 | 2.31E-39 |
| BIRC6 | SNHG16 | 0.525799 | 2.30E-39 |
| MAPK8 | AP000873.2 | 0.525807 | 2.29E-39 |
| NAF1 | AC026124.2 | 0.525869 | 2.24E-39 |
| PIK3C3 | AP001486.2 | 0.525875 | 2.23E-39 |
| ATG2B | AC073534.1 | 0.5259 | 2.21E-39 |
| PTEN | AC139887.1 | 0.525927 | 2.19E-39 |
| RPS6KB1 | AC124319.1 | 0.525935 | 2.18E-39 |
| KLHL24 | AL133342.1 | 0.525981 | 2.14E-39 |
| WDFY3 | AC124016.2 | 0.525986 | 2.14E-39 |
| FOXO1 | AC099343.2 | 0.525989 | 2.13E-39 |
| RB1CC1 | ARHGEF38-IT1 | 0.525995 | 2.13E-39 |
| RPS6KB1 | AC016394.1 | 0.525996 | 2.13E-39 |
| NAF1 | AC010186.3 | 0.526056 | 2.08E-39 |
| RPS6KB1 | NEAT1 | 0.526096 | 2.05E-39 |
| KLHL24 | AL450263.1 | 0.526162 | 2.00E-39 |
| KLHL24 | AC099343.2 | 0.526171 | 1.99E-39 |
| KLHL24 | AC012170.2 | 0.526226 | 1.95E-39 |
| RB1CC1 | AC026356.1 | 0.52625 | 1.93E-39 |
| RPS6KB1 | AC138393.3 | 0.52627 | 1.91E-39 |
| PIK3C3 | AC120349.1 | 0.526323 | 1.87E-39 |
| RB1CC1 | AC026202.2 | 0.526324 | 1.87E-39 |
| MAPK8 | AC007014.2 | 0.526353 | 1.85E-39 |
| PTEN | MIR155HG | 0.526392 | 1.82E-39 |
| CFLAR | AC005479.2 | 0.526427 | 1.80E-39 |
| ATG12 | AC084871.1 | 0.52648 | 1.76E-39 |
| BIRC6 | AL031667.3 | 0.526514 | 1.74E-39 |
| NAF1 | ABALON | 0.526526 | 1.73E-39 |
| RPS6KB1 | AC026124.2 | 0.526529 | 1.73E-39 |
| MAPK8 | AL513534.1 | 0.526539 | 1.72E-39 |
| ATG2B | AC005479.1 | 0.526567 | 1.70E-39 |
| KLHL24 | AC108010.1 | 0.526589 | 1.69E-39 |
| NAF1 | AC093788.1 | 0.526601 | 1.68E-39 |
| MAPK8 | ALMS1-IT1 | 0.526611 | 1.67E-39 |
| NAF1 | SAP30L-AS1 | 0.526616 | 1.67E-39 |
| CFLAR | AC002128.2 | 0.52665 | 1.65E-39 |
| ATG12 | AC090198.1 | 0.526654 | 1.65E-39 |
| NAF1 | AL021578.1 | 0.526658 | 1.64E-39 |
| PIK3C3 | LINC00513 | 0.526663 | 1.64E-39 |
| ATG2B | CTBP1-AS | 0.526676 | 1.63E-39 |
| EIF2AK2 | AC093788.1 | 0.526699 | 1.62E-39 |
| RAB24 | AC132872.3 | 0.5267 | 1.62E-39 |
| PTEN | ARMCX5-GPRASP2 | 0.526704 | 1.62E-39 |
| RPS6KB1 | AC004918.3 | 0.526751 | 1.59E-39 |
| RB1CC1 | BTBD9-AS1 | 0.526762 | 1.58E-39 |
| ATG4B | LINC00265 | 0.526835 | 1.53E-39 |
| FOXO1 | AC008906.1 | 0.526843 | 1.53E-39 |
| SPNS1 | AL031709.1 | 0.526856 | 1.52E-39 |
| CAPN10 | AC084018.1 | 0.526917 | 1.49E-39 |
| EIF2AK2 | AC073046.1 | 0.526927 | 1.48E-39 |
| ATG2B | AC004067.1 | 0.526947 | 1.47E-39 |
| ATG12 | LINC01184 | 0.526964 | 1.46E-39 |
| TSC1 | AC012170.2 | 0.526989 | 1.45E-39 |
| ATG16L2 | AL390728.6 | 0.527041 | 1.42E-39 |
| FOXO1 | AC024933.1 | 0.527062 | 1.40E-39 |
| RB1 | AC087286.2 | 0.527076 | 1.40E-39 |
| WDFY3 | AL031667.3 | 0.527078 | 1.40E-39 |
| RB1 | AC130895.1 | 0.527095 | 1.39E-39 |
| NLRC4 | LINC01150 | 0.527135 | 1.37E-39 |
| RB1CC1 | ADAMTSL4-AS1 | 0.527135 | 1.36E-39 |
| RPS6KB1 | LINC-PINT | 0.527141 | 1.36E-39 |
| EIF2AK2 | AC018752.1 | 0.527182 | 1.34E-39 |
| ATG16L2 | U62317.2 | 0.527184 | 1.34E-39 |
| RPS6KB1 | AC005104.1 | 0.527208 | 1.33E-39 |
| BIRC6 | AC005332.3 | 0.527211 | 1.32E-39 |
| MAPK8 | SCARNA9 | 0.52722 | 1.32E-39 |
| PTEN | AC087752.4 | 0.527221 | 1.32E-39 |
| FOXO1 | TRAF3IP2-AS1 | 0.527238 | 1.31E-39 |
| ATG2B | AC025178.1 | 0.527263 | 1.30E-39 |
| RPS6KB1 | AC025917.1 | 0.527274 | 1.29E-39 |
| ATG2B | SNHG26 | 0.527299 | 1.28E-39 |
| ATG12 | AC020978.3 | 0.527328 | 1.27E-39 |
| EIF2AK2 | AC083862.2 | 0.527336 | 1.26E-39 |
| RB1 | AC108449.2 | 0.527347 | 1.26E-39 |
| RB1CC1 | PAXBP1-AS1 | 0.52736 | 1.25E-39 |
| WDFY3 | AL049840.3 | 0.527362 | 1.25E-39 |
| ATG2B | NORAD | 0.527395 | 1.23E-39 |
| WDFY3 | AC078883.1 | 0.527426 | 1.22E-39 |
| WDFY3 | AC123595.1 | 0.527474 | 1.20E-39 |
| PTEN | AL031717.1 | 0.527482 | 1.19E-39 |
| CXCR4 | TRG-AS1 | 0.527486 | 1.19E-39 |
| PIK3C3 | AC010834.3 | 0.527521 | 1.17E-39 |
| BIRC5 | RNASEH1-AS1 | 0.527608 | 1.13E-39 |
| TSC1 | PCBP1-AS1 | 0.527611 | 1.13E-39 |
| CFLAR | AC087286.2 | 0.527616 | 1.13E-39 |
| ATG16L2 | DICER1-AS1 | 0.527671 | 1.11E-39 |
| TSC1 | AL021707.7 | 0.527721 | 1.09E-39 |
| BIRC6 | LINC01004 | 0.527789 | 1.06E-39 |
| EIF2AK2 | AL513550.1 | 0.527807 | 1.05E-39 |
| RPS6KB1 | AC073487.1 | 0.527854 | 1.03E-39 |
| MAPK8 | AC000123.1 | 0.527854 | 1.03E-39 |
| PIK3C3 | DLEU1 | 0.527872 | 1.02E-39 |
| NAF1 | DLEU2 | 0.527887 | 1.02E-39 |
| RB1CC1 | AC073046.1 | 0.527966 | 9.86E-40 |
| KLHL24 | AL031775.2 | 0.527975 | 9.83E-40 |
| BIRC6 | AC010245.2 | 0.528031 | 9.61E-40 |
| ATG4B | AL022328.3 | 0.528075 | 9.45E-40 |
| FOXO1 | AL365277.1 | 0.528079 | 9.43E-40 |
| WDFY3 | AC009948.4 | 0.528139 | 9.21E-40 |
| ATG12 | AC127024.5 | 0.528165 | 9.12E-40 |
| ATG12 | AP000786.1 | 0.528171 | 9.10E-40 |
| PIK3C3 | AC087286.1 | 0.528197 | 9.00E-40 |
| PIK3C3 | AC098851.1 | 0.528202 | 8.99E-40 |
| BIRC6 | AC113139.1 | 0.528266 | 8.77E-40 |
| RPS6KB1 | CFLAR-AS1 | 0.528288 | 8.69E-40 |
| PTEN | AC007938.3 | 0.528341 | 8.51E-40 |
| RPS6KB1 | AP001381.1 | 0.528343 | 8.50E-40 |
| ATG12 | AL132989.1 | 0.528366 | 8.43E-40 |
| KLHL24 | AC009318.2 | 0.528396 | 8.33E-40 |
| TSC1 | AC090425.2 | 0.528398 | 8.32E-40 |
| RPS6KB1 | ACAP2-IT1 | 0.528401 | 8.31E-40 |
| TSC1 | AC090948.3 | 0.528404 | 8.30E-40 |
| CAPN10 | AC073896.4 | 0.528418 | 8.26E-40 |
| BIRC6 | ARMCX5-GPRASP2 | 0.528454 | 8.14E-40 |
| CFLAR | AC066613.1 | 0.528484 | 8.04E-40 |
| WDFY3 | AL606489.1 | 0.528518 | 7.94E-40 |
| MAPK8 | AC008115.3 | 0.528562 | 7.80E-40 |
| RPS6KB1 | AC008906.1 | 0.528629 | 7.60E-40 |
| MAPK8 | AC124312.5 | 0.52863 | 7.60E-40 |
| TSC1 | AL157786.1 | 0.528633 | 7.59E-40 |
| EIF2AK2 | AL590723.1 | 0.528698 | 7.40E-40 |
| EIF2AK2 | AL137782.1 | 0.528723 | 7.32E-40 |
| PTEN | AL158166.1 | 0.528743 | 7.27E-40 |
| ATG4B | AL022328.1 | 0.528771 | 7.19E-40 |
| ATG16L2 | AL591895.1 | 0.528878 | 6.89E-40 |
| EIF2AK2 | AL163051.2 | 0.528921 | 6.78E-40 |
| CFLAR | AC013403.2 | 0.528941 | 6.72E-40 |
| PIK3C3 | MIR29B2CHG | 0.529072 | 6.38E-40 |
| NAF1 | AC002553.2 | 0.52912 | 6.27E-40 |
| MAPK8 | AC092794.1 | 0.529124 | 6.26E-40 |
| ATG12 | AC015849.3 | 0.529147 | 6.20E-40 |
| ATG12 | AC079684.1 | 0.529152 | 6.19E-40 |
| NAF1 | AC087392.1 | 0.529181 | 6.12E-40 |
| BIRC6 | AC008870.2 | 0.529205 | 6.06E-40 |
| MAPK8 | AC002128.2 | 0.529218 | 6.03E-40 |
| CAPN10 | AC074212.1 | 0.529222 | 6.02E-40 |
| RB1CC1 | AC018926.3 | 0.52923 | 6.00E-40 |
| ATG16L2 | AC020907.4 | 0.529241 | 5.97E-40 |
| MAPK8 | GAS5-AS1 | 0.529244 | 5.97E-40 |
| CFLAR | AC135050.5 | 0.529315 | 5.80E-40 |
| PIK3C3 | AL157392.3 | 0.529347 | 5.73E-40 |
| WDFY3 | AL133371.2 | 0.529411 | 5.59E-40 |
| ATG12 | STARD4-AS1 | 0.529457 | 5.49E-40 |
| BIRC6 | AL080317.1 | 0.529526 | 5.34E-40 |
| CFLAR | AC087286.4 | 0.529546 | 5.30E-40 |
| NAF1 | AC004223.3 | 0.529593 | 5.20E-40 |
| ATG12 | FMR1-IT1 | 0.5296 | 5.19E-40 |
| EIF2AK2 | AC015849.3 | 0.529601 | 5.18E-40 |
| CFLAR | MALAT1 | 0.529614 | 5.16E-40 |
| CFLAR | AC131971.1 | 0.52964 | 5.10E-40 |
| RPS6KB1 | RHOA-IT1 | 0.529669 | 5.05E-40 |
| RB1CC1 | SMC5-AS1 | 0.529681 | 5.02E-40 |
| CFLAR | AC124312.5 | 0.529714 | 4.96E-40 |
| TSC1 | AC025171.4 | 0.529723 | 4.94E-40 |
| CFLAR | AC010186.3 | 0.52974 | 4.91E-40 |
| EIF2AK2 | RHOA-IT1 | 0.529744 | 4.90E-40 |
| NAF1 | AC253576.2 | 0.529747 | 4.89E-40 |
| KLHL24 | CFLAR-AS1 | 0.529782 | 4.83E-40 |
| KLHL24 | AC087284.1 | 0.52979 | 4.81E-40 |
| ATG4B | AC016773.1 | 0.529796 | 4.80E-40 |
| RB1CC1 | AC087284.1 | 0.529805 | 4.79E-40 |
| PTEN | AC135050.5 | 0.529816 | 4.76E-40 |
| KLHL24 | AL132989.1 | 0.529827 | 4.74E-40 |
| NAF1 | AL162724.1 | 0.529843 | 4.71E-40 |
| KLHL24 | PSPC1-AS2 | 0.529901 | 4.61E-40 |
| RPS6KB1 | AC087276.1 | 0.529925 | 4.56E-40 |
| RPS6KB1 | AC138207.4 | 0.529968 | 4.49E-40 |
| RB1CC1 | AC242426.2 | 0.529992 | 4.44E-40 |
| EIF2AK2 | AP001432.1 | 0.530041 | 4.36E-40 |
| EIF2AK2 | AC022973.3 | 0.530043 | 4.35E-40 |
| PTEN | AC048341.1 | 0.530067 | 4.31E-40 |
| TSC1 | AC139795.2 | 0.530067 | 4.31E-40 |
| RPS6KB1 | AC012170.2 | 0.530097 | 4.26E-40 |
| ATG16L2 | IGBP1-AS1 | 0.5301 | 4.26E-40 |
| KLHL24 | AC096741.1 | 0.530135 | 4.20E-40 |
| ATG2B | MIR222HG | 0.530136 | 4.20E-40 |
| GOPC | DNM3OS | 0.530147 | 4.18E-40 |
| FOXO1 | AL021578.1 | 0.530163 | 4.15E-40 |
| PIK3C3 | AL022067.1 | 0.530206 | 4.08E-40 |
| RPS6KB1 | AL136115.2 | 0.530208 | 4.08E-40 |
| EIF2AK2 | AP002907.1 | 0.530247 | 4.02E-40 |
| PIK3C3 | AC011477.2 | 0.530262 | 3.99E-40 |
| MAPK8 | AC253536.3 | 0.530281 | 3.96E-40 |
| MAPK8 | AC021851.1 | 0.530288 | 3.95E-40 |
| FOXO1 | A2M-AS1 | 0.530314 | 3.91E-40 |
| RB1CC1 | AL133330.1 | 0.530365 | 3.84E-40 |
| TSC1 | KDM4A-AS1 | 0.530392 | 3.79E-40 |
| GOPC | AC108449.2 | 0.53047 | 3.68E-40 |
| EIF2AK2 | AC007546.1 | 0.53049 | 3.65E-40 |
| ATG12 | AC138956.2 | 0.530501 | 3.63E-40 |
| MAPK8 | AC141002.1 | 0.530518 | 3.61E-40 |
| MAPK8 | AC124312.4 | 0.530523 | 3.60E-40 |
| KLHL24 | AC087286.4 | 0.530525 | 3.60E-40 |
| FOXO1 | SMC5-AS1 | 0.530531 | 3.59E-40 |
| NAF1 | AC127024.4 | 0.530571 | 3.54E-40 |
| RB1CC1 | AC006017.1 | 0.530647 | 3.43E-40 |
| EIF2AK2 | C5orf56 | 0.530693 | 3.37E-40 |
| CFLAR | AC007878.1 | 0.530704 | 3.35E-40 |
| CFLAR | AC004918.3 | 0.530727 | 3.32E-40 |
| TSC1 | AC073487.1 | 0.530735 | 3.31E-40 |
| ATG16L2 | LINC00265 | 0.530745 | 3.30E-40 |
| TSC1 | AC010761.3 | 0.530766 | 3.27E-40 |
| FOXO1 | PPP3CB-AS1 | 0.530788 | 3.24E-40 |
| FOXO1 | AC027097.2 | 0.530818 | 3.21E-40 |
| EIF2AK2 | AP000786.1 | 0.530877 | 3.13E-40 |
| TSC1 | AC138956.1 | 0.530893 | 3.11E-40 |
| CFLAR | HCG27 | 0.530914 | 3.09E-40 |
| KLHL24 | AC026356.1 | 0.530972 | 3.02E-40 |
| FOXO1 | AF129075.1 | 0.530977 | 3.01E-40 |
| EIF2AK2 | AC004918.3 | 0.530982 | 3.00E-40 |
| NAF1 | AC131971.1 | 0.531047 | 2.93E-40 |
| PIK3C3 | AP001429.1 | 0.531057 | 2.92E-40 |
| MAPK8 | AC015849.3 | 0.531092 | 2.88E-40 |
| PIK3C3 | LINC00641 | 0.531108 | 2.86E-40 |
| EIF2S1 | AC099850.3 | 0.531119 | 2.85E-40 |
| CAPN10 | AC008735.2 | 0.531154 | 2.81E-40 |
| WDFY3 | AC011477.3 | 0.5312 | 2.76E-40 |
| ATG4B | TMEM147-AS1 | 0.531231 | 2.72E-40 |
| BIRC6 | AC145423.3 | 0.531244 | 2.71E-40 |
| ATG2B | AL049840.4 | 0.531354 | 2.59E-40 |
| CFLAR | AL133445.2 | 0.531363 | 2.58E-40 |
| PIK3C3 | PSPC1-AS2 | 0.531479 | 2.47E-40 |
| ATG12 | AC245884.8 | 0.531494 | 2.45E-40 |
| NAF1 | AC110792.3 | 0.53151 | 2.44E-40 |
| PTEN | INE1 | 0.531547 | 2.40E-40 |
| RB1CC1 | AC005920.2 | 0.531558 | 2.39E-40 |
| CFLAR | AC025171.2 | 0.531597 | 2.35E-40 |
| RB1CC1 | AC105389.2 | 0.531629 | 2.32E-40 |
| MAPK8 | ANKRD10-IT1 | 0.531641 | 2.31E-40 |
| KLHL24 | AC053513.1 | 0.531654 | 2.30E-40 |
| ATG12 | AC012467.1 | 0.531692 | 2.27E-40 |
| MAPK8 | AL355075.2 | 0.531754 | 2.21E-40 |
| RB1CC1 | AL359697.1 | 0.531814 | 2.16E-40 |
| CFLAR | AP001458.1 | 0.531814 | 2.16E-40 |
| WDFY3 | AC024060.1 | 0.531827 | 2.15E-40 |
| PIK3C3 | AC127024.4 | 0.53186 | 2.12E-40 |
| FOXO1 | AC087286.1 | 0.531911 | 2.08E-40 |
| RB1CC1 | AC002064.2 | 0.531954 | 2.04E-40 |
| TSC1 | AC008124.1 | 0.532029 | 1.98E-40 |
| PIK3C3 | AL513550.1 | 0.532104 | 1.92E-40 |
| TSC1 | LINC00894 | 0.532117 | 1.92E-40 |
| EIF2AK2 | MCM3AP-AS1 | 0.532135 | 1.90E-40 |
| CFLAR | AL022067.1 | 0.532135 | 1.90E-40 |
| ATG12 | AC013403.2 | 0.532154 | 1.89E-40 |
| PIK3C3 | A2M-AS1 | 0.532196 | 1.86E-40 |
| NAF1 | AC010834.3 | 0.532225 | 1.83E-40 |
| ATG12 | AC004908.3 | 0.532227 | 1.83E-40 |
| CFLAR | AC245014.3 | 0.532233 | 1.83E-40 |
| NAF1 | AC138932.5 | 0.532244 | 1.82E-40 |
| FOXO1 | ALG13-AS1 | 0.532267 | 1.80E-40 |
| RPS6KB1 | AL590723.1 | 0.532283 | 1.79E-40 |
| RB1CC1 | LAMC1-AS1 | 0.532371 | 1.73E-40 |
| CFLAR | AC087276.1 | 0.532376 | 1.73E-40 |
| PIK3C3 | NEAT1 | 0.532394 | 1.72E-40 |
| PIK3C3 | AC024075.3 | 0.532396 | 1.71E-40 |
| ATG4B | U62317.2 | 0.532413 | 1.70E-40 |
| RPS6KB1 | PAXBP1-AS1 | 0.532441 | 1.68E-40 |
| ATG16L2 | AL390719.2 | 0.532459 | 1.67E-40 |
| RPS6KB1 | AC007878.1 | 0.53246 | 1.67E-40 |
| RAB24 | PTOV1-AS2 | 0.532471 | 1.66E-40 |
| PIK3C3 | AC090579.1 | 0.532503 | 1.64E-40 |
| RB1CC1 | Z83843.1 | 0.53251 | 1.64E-40 |
| KLHL24 | AP002336.2 | 0.532513 | 1.64E-40 |
| TSC1 | AL662844.3 | 0.532537 | 1.62E-40 |
| ATG16L2 | AC003102.1 | 0.532537 | 1.62E-40 |
| WDFY3 | AL357060.1 | 0.532617 | 1.57E-40 |
| FOXO1 | AL162724.1 | 0.532626 | 1.56E-40 |
| ATG2B | AC016542.1 | 0.532676 | 1.53E-40 |
| KLHL24 | AP000786.1 | 0.532755 | 1.49E-40 |
| PIK3C3 | AC027097.1 | 0.532849 | 1.43E-40 |
| TSC1 | C1RL-AS1 | 0.532867 | 1.42E-40 |
| GOPC | AP000766.1 | 0.532911 | 1.40E-40 |
| MAPK8 | AC090948.3 | 0.532914 | 1.39E-40 |
| CFLAR | AC008537.2 | 0.532916 | 1.39E-40 |
| FOXO1 | AL158166.2 | 0.532916 | 1.39E-40 |
| FOXO1 | AC026470.2 | 0.53293 | 1.39E-40 |
| TSC1 | AC096992.2 | 0.532944 | 1.38E-40 |
| TSC1 | AC135050.5 | 0.532982 | 1.36E-40 |
| CAPN10 | AP002807.1 | 0.532996 | 1.35E-40 |
| TSC1 | AC120053.1 | 0.532998 | 1.35E-40 |
| PIK3C3 | KIF26B-AS1 | 0.533004 | 1.35E-40 |
| EIF2AK2 | SMC5-AS1 | 0.533011 | 1.34E-40 |
| TSC1 | AC012615.6 | 0.533054 | 1.32E-40 |
| EIF2AK2 | Z98884.2 | 0.533064 | 1.31E-40 |
| MAPK8 | AC004241.3 | 0.533076 | 1.31E-40 |
| PTEN | AC093495.1 | 0.533095 | 1.30E-40 |
| KLHL24 | AC022973.3 | 0.533114 | 1.29E-40 |
| EIF2AK2 | KLF7-IT1 | 0.533155 | 1.27E-40 |
| WDFY3 | AC073655.2 | 0.533195 | 1.25E-40 |
| CFLAR | AC006017.1 | 0.533211 | 1.24E-40 |
| PTEN | AC053527.1 | 0.533227 | 1.23E-40 |
| EIF2AK2 | AL109614.1 | 0.533253 | 1.22E-40 |
| MAPK8 | AC087286.1 | 0.533328 | 1.18E-40 |
| KLHL24 | AC021078.1 | 0.533349 | 1.17E-40 |
| MAPK8 | AL022067.1 | 0.533402 | 1.15E-40 |
| FOXO1 | ATP1A1-AS1 | 0.53341 | 1.14E-40 |
| NLRC4 | PARAL1 | 0.533429 | 1.14E-40 |
| TSC1 | AC002128.2 | 0.533439 | 1.13E-40 |
| BIRC6 | AC080013.4 | 0.533464 | 1.12E-40 |
| RPS6KB1 | AC138932.5 | 0.533465 | 1.12E-40 |
| FOXO1 | AC124312.5 | 0.533513 | 1.10E-40 |
| MAPK8 | AL596325.2 | 0.533521 | 1.09E-40 |
| FOXO1 | AC007038.1 | 0.533543 | 1.08E-40 |
| EIF2AK2 | AP005131.7 | 0.533559 | 1.08E-40 |
| ATG2B | AC015813.1 | 0.533608 | 1.06E-40 |
| FOXO1 | AC097376.2 | 0.533612 | 1.06E-40 |
| PIK3C3 | AF129075.1 | 0.53363 | 1.05E-40 |
| KLHL24 | AC100830.2 | 0.533632 | 1.05E-40 |
| ATG16L2 | AC004253.1 | 0.533643 | 1.04E-40 |
| RPS6KB1 | AC008115.3 | 0.533669 | 1.03E-40 |
| ATG2B | AC011468.5 | 0.533679 | 1.03E-40 |
| TSC1 | AP006621.2 | 0.533718 | 1.01E-40 |
| CFLAR | AC110792.3 | 0.53373 | 1.01E-40 |
| RPS6KB1 | SP2-AS1 | 0.53374 | 1.00E-40 |
| TSC1 | AC005261.1 | 0.533784 | 9.85E-41 |
| CFLAR | AC005856.1 | 0.533801 | 9.79E-41 |
| KLHL24 | AC073651.1 | 0.533858 | 9.57E-41 |
| FOXO1 | AL157392.3 | 0.533946 | 9.24E-41 |
| KLHL24 | AC010186.3 | 0.53398 | 9.11E-41 |
| CFLAR | LINC01355 | 0.533985 | 9.09E-41 |
| MAPK8 | NEAT1 | 0.534004 | 9.03E-41 |
| RPS6KB1 | NARF-IT1 | 0.534032 | 8.93E-41 |
| MAPK8 | AC021078.1 | 0.534074 | 8.78E-41 |
| RPS6KB1 | AC015813.1 | 0.534098 | 8.69E-41 |
| ATG12 | NUTM2A-AS1 | 0.534118 | 8.62E-41 |
| RB1CC1 | AC018752.1 | 0.534164 | 8.46E-41 |
| PIK3C3 | MCM3AP-AS1 | 0.534218 | 8.28E-41 |
| ATG2B | AL031667.3 | 0.53422 | 8.28E-41 |
| CFLAR | ANKRD10-IT1 | 0.534225 | 8.26E-41 |
| GOPC | AL109614.1 | 0.534232 | 8.24E-41 |
| FOXO1 | AC004466.3 | 0.534258 | 8.15E-41 |
| KLHL24 | MACC1-AS1 | 0.53429 | 8.05E-41 |
| CFLAR | AC006059.1 | 0.534295 | 8.03E-41 |
| TSC1 | AP005899.1 | 0.534421 | 7.64E-41 |
| ATG2B | AC022306.2 | 0.534422 | 7.64E-41 |
| DNAJB9 | AC104699.1 | 0.534429 | 7.61E-41 |
| PIK3C3 | NDUFV2-AS1 | 0.534471 | 7.48E-41 |
| CFLAR | AC007216.4 | 0.534483 | 7.45E-41 |
| RPS6KB1 | AC021078.1 | 0.534519 | 7.34E-41 |
| EIF2AK2 | AC009318.2 | 0.534523 | 7.33E-41 |
| RB1CC1 | AL353804.1 | 0.534575 | 7.18E-41 |
| RPS6KB1 | AC018690.1 | 0.534606 | 7.09E-41 |
| FOXO1 | AC018682.1 | 0.534639 | 7.00E-41 |
| MAPK8 | AL592148.3 | 0.534642 | 6.99E-41 |
| FOXO1 | AL117381.1 | 0.534655 | 6.95E-41 |
| RB1CC1 | AC020915.2 | 0.5347 | 6.83E-41 |
| RPS6KB1 | AC066613.1 | 0.534716 | 6.79E-41 |
| PIK3C3 | AC253576.2 | 0.534806 | 6.55E-41 |
| CFLAR | AL137782.1 | 0.534807 | 6.54E-41 |
| RB1 | AC006059.1 | 0.53482 | 6.51E-41 |
| NAF1 | AL353804.2 | 0.534832 | 6.48E-41 |
| RB1CC1 | AC090948.1 | 0.534864 | 6.39E-41 |
| RPS6KB1 | AC067852.3 | 0.534866 | 6.39E-41 |
| NAF1 | AP001429.1 | 0.534935 | 6.21E-41 |
| PIK3C3 | ADAMTSL4-AS1 | 0.534976 | 6.11E-41 |
| CFLAR | AC124319.1 | 0.534991 | 6.08E-41 |
| ATG2B | AC009120.2 | 0.535012 | 6.03E-41 |
| FOXO1 | AC138393.3 | 0.535043 | 5.95E-41 |
| KLHL24 | AC006059.1 | 0.535064 | 5.90E-41 |
| CFLAR | LINC00426 | 0.535081 | 5.86E-41 |
| RB1CC1 | AC012181.1 | 0.5351 | 5.82E-41 |
| BIRC6 | AL357060.1 | 0.535109 | 5.80E-41 |
| BIRC6 | AC004656.1 | 0.535161 | 5.68E-41 |
| NAF1 | AC022973.3 | 0.535172 | 5.65E-41 |
| RB1CC1 | AC096586.2 | 0.53525 | 5.48E-41 |
| EIF2AK2 | AC020913.3 | 0.535253 | 5.47E-41 |
| RPS6KB1 | AC005021.1 | 0.535285 | 5.40E-41 |
| NAF1 | SNHG14 | 0.535334 | 5.30E-41 |
| ATG12 | AP005899.1 | 0.535376 | 5.21E-41 |
| ATG2B | AC234772.2 | 0.535397 | 5.16E-41 |
| TSC1 | AC091185.1 | 0.535442 | 5.07E-41 |
| KLHL24 | LINC01355 | 0.535464 | 5.03E-41 |
| KLHL24 | AC006270.1 | 0.535505 | 4.94E-41 |
| RB1CC1 | AC110792.3 | 0.535563 | 4.83E-41 |
| FOXO1 | AL162724.2 | 0.535563 | 4.83E-41 |
| ATG12 | ACTA2-AS1 | 0.535587 | 4.78E-41 |
| ATG16L2 | AL008582.1 | 0.535592 | 4.77E-41 |
| ATG16L2 | AL049840.4 | 0.535605 | 4.75E-41 |
| KLHL24 | AC002128.2 | 0.535606 | 4.75E-41 |
| KLHL24 | AC007684.1 | 0.535651 | 4.66E-41 |
| NAF1 | AC002550.2 | 0.535659 | 4.65E-41 |
| ATG4B | CAPN10-DT | 0.535669 | 4.63E-41 |
| CFLAR | AC005104.1 | 0.535674 | 4.62E-41 |
| MAPK8 | AC026202.2 | 0.535676 | 4.62E-41 |
| TSC1 | AC012360.3 | 0.535691 | 4.59E-41 |
| NAF1 | BTBD9-AS1 | 0.535722 | 4.53E-41 |
| KLHL24 | AC024075.1 | 0.535725 | 4.52E-41 |
| ATG12 | AC005034.5 | 0.535735 | 4.51E-41 |
| MAPK8 | AC067852.3 | 0.535735 | 4.51E-41 |
| KLHL24 | AC012181.1 | 0.535743 | 4.49E-41 |
| EIF2AK2 | AL359697.1 | 0.535765 | 4.45E-41 |
| ATG4B | AC006435.2 | 0.535798 | 4.39E-41 |
| ATG16L2 | AC109460.2 | 0.535859 | 4.29E-41 |
| MAPK8 | EBLN3P | 0.535863 | 4.28E-41 |
| ATG16L2 | AC009118.3 | 0.535868 | 4.27E-41 |
| KLHL24 | AL513550.1 | 0.535886 | 4.24E-41 |
| MAPK8 | AF129075.1 | 0.535887 | 4.24E-41 |
| CFLAR | AC096921.2 | 0.535896 | 4.22E-41 |
| CFLAR | AC009090.1 | 0.535913 | 4.19E-41 |
| PTEN | AL139041.1 | 0.535944 | 4.14E-41 |
| BIRC6 | DGUOK-AS1 | 0.535956 | 4.12E-41 |
| PIK3C3 | RRN3P2 | 0.536006 | 4.04E-41 |
| MAPK8 | AC019080.5 | 0.53602 | 4.02E-41 |
| RPS6KB1 | AC010186.3 | 0.53603 | 4.00E-41 |
| PIK3C3 | CD44-AS1 | 0.536049 | 3.97E-41 |
| MAPK8 | AC008124.1 | 0.536062 | 3.95E-41 |
| CFLAR | AL138963.1 | 0.53611 | 3.88E-41 |
| KLHL24 | AC019080.5 | 0.536114 | 3.87E-41 |
| MAPK8 | FLNB-AS1 | 0.536122 | 3.86E-41 |
| ATG2B | AC008982.2 | 0.536132 | 3.84E-41 |
| PIK3C3 | AP002336.2 | 0.536134 | 3.84E-41 |
| MAPK8 | AC020913.3 | 0.536155 | 3.81E-41 |
| CFLAR | AC093788.1 | 0.536181 | 3.77E-41 |
| PIK3C3 | AC026470.2 | 0.536201 | 3.74E-41 |
| FOXO1 | AC026202.2 | 0.536218 | 3.71E-41 |
| PIK3C3 | BTBD9-AS1 | 0.536226 | 3.70E-41 |
| RPS6KB1 | AC004477.3 | 0.536268 | 3.64E-41 |
| MAPK8 | FAM13A-AS1 | 0.536286 | 3.61E-41 |
| RB1CC1 | AL117381.1 | 0.536307 | 3.58E-41 |
| WDFY3 | AC008982.2 | 0.536312 | 3.57E-41 |
| CFLAR | AC002553.2 | 0.536349 | 3.52E-41 |
| PIK3C3 | AC021078.1 | 0.536368 | 3.49E-41 |
| EIF2AK2 | LINC01355 | 0.536375 | 3.48E-41 |
| MAPK8 | ADNP-AS1 | 0.53638 | 3.48E-41 |
| EIF2AK2 | AC127024.4 | 0.536448 | 3.38E-41 |
| ATG16L2 | AC009120.2 | 0.53649 | 3.32E-41 |
| EIF2AK2 | AC090425.2 | 0.5365 | 3.31E-41 |
| BIRC6 | AC112496.1 | 0.536519 | 3.29E-41 |
| RPS6KB1 | AC108727.1 | 0.536642 | 3.13E-41 |
| ATG12 | EGOT | 0.536651 | 3.12E-41 |
| CAPN10 | AC016773.1 | 0.536701 | 3.05E-41 |
| KLHL24 | NDUFV2-AS1 | 0.536713 | 3.04E-41 |
| ATG12 | AC069023.1 | 0.536736 | 3.01E-41 |
| PIK3C3 | AL133342.1 | 0.53678 | 2.96E-41 |
| CFLAR | AC004832.5 | 0.536783 | 2.95E-41 |
| PTEN | UGDH-AS1 | 0.536813 | 2.92E-41 |
| ATG4B | AC020907.4 | 0.536832 | 2.90E-41 |
| TSC1 | AL512791.1 | 0.536833 | 2.90E-41 |
| CTSB | LINC01094 | 0.536843 | 2.88E-41 |
| FOXO1 | AL133342.1 | 0.536899 | 2.82E-41 |
| ATG12 | AL355075.2 | 0.53693 | 2.78E-41 |
| NAF1 | AC138393.3 | 0.537033 | 2.67E-41 |
| EIF2AK2 | AC018926.3 | 0.537135 | 2.56E-41 |
| EIF2AK2 | AC110792.3 | 0.537152 | 2.54E-41 |
| CFLAR | AC090579.1 | 0.53717 | 2.53E-41 |
| NAF1 | AC021078.1 | 0.537232 | 2.46E-41 |
| FOXO1 | ADNP-AS1 | 0.537232 | 2.46E-41 |
| MAPK8 | LINC-PINT | 0.537249 | 2.45E-41 |
| MAPK8 | AC099343.2 | 0.537292 | 2.40E-41 |
| KLHL24 | AL136320.1 | 0.537329 | 2.37E-41 |
| FOXO1 | AC090948.3 | 0.53735 | 2.35E-41 |
| ATG2B | AC025171.2 | 0.537417 | 2.29E-41 |
| ATG2B | AL157932.1 | 0.537442 | 2.26E-41 |
| ATG12 | AC012181.2 | 0.537489 | 2.22E-41 |
| PTEN | LANCL1-AS1 | 0.537574 | 2.15E-41 |
| KLHL24 | LIMS1-AS1 | 0.537601 | 2.12E-41 |
| RPS6KB1 | KLF7-IT1 | 0.537649 | 2.08E-41 |
| TSC1 | AL021707.8 | 0.537651 | 2.08E-41 |
| NAF1 | AC005920.2 | 0.537702 | 2.04E-41 |
| KLHL24 | SCARNA9 | 0.537735 | 2.01E-41 |
| TSC1 | AL355488.1 | 0.537738 | 2.01E-41 |
| PIK3C3 | AC009032.1 | 0.537789 | 1.97E-41 |
| ATG12 | AC242426.2 | 0.537849 | 1.92E-41 |
| RB1CC1 | AC096741.1 | 0.537927 | 1.86E-41 |
| TSC1 | AC097641.2 | 0.537935 | 1.85E-41 |
| RB1CC1 | AC108010.1 | 0.538045 | 1.77E-41 |
| EIF2AK2 | AC053513.1 | 0.538045 | 1.77E-41 |
| KLHL24 | AL117381.1 | 0.538065 | 1.76E-41 |
| PIK3C3 | AC026202.2 | 0.538113 | 1.72E-41 |
| TSC1 | NFYC-AS1 | 0.53815 | 1.70E-41 |
| NAF1 | OIP5-AS1 | 0.538175 | 1.68E-41 |
| KLHL24 | AL163051.2 | 0.538193 | 1.67E-41 |
| PTEN | AP001528.2 | 0.538204 | 1.66E-41 |
| ATG2B | AC004908.3 | 0.538263 | 1.62E-41 |
| PIK3C3 | AC107068.1 | 0.538278 | 1.61E-41 |
| ATG12 | AC037198.2 | 0.538298 | 1.60E-41 |
| ATG2B | AL080317.1 | 0.538307 | 1.59E-41 |
| RB1CC1 | AC073487.1 | 0.538326 | 1.58E-41 |
| PIK3C3 | DLEU2 | 0.538328 | 1.58E-41 |
| ATG2B | AC011815.1 | 0.538352 | 1.57E-41 |
| ATG16L2 | AC008735.2 | 0.538362 | 1.56E-41 |
| TSC1 | AC060780.1 | 0.538407 | 1.53E-41 |
| KLHL24 | AC087286.2 | 0.538429 | 1.52E-41 |
| ATG4B | DM1-AS | 0.538446 | 1.51E-41 |
| RPS6KB1 | AP001432.1 | 0.538456 | 1.50E-41 |
| CFLAR | AC124312.2 | 0.538461 | 1.50E-41 |
| BIRC6 | AC073655.2 | 0.538485 | 1.48E-41 |
| CFLAR | AC020913.3 | 0.538515 | 1.47E-41 |
| FOXO1 | AL031670.1 | 0.538546 | 1.45E-41 |
| ATG4B | AL031714.1 | 0.538563 | 1.44E-41 |
| ATG16L2 | AC011462.4 | 0.538582 | 1.43E-41 |
| CFLAR | Z82243.1 | 0.538607 | 1.41E-41 |
| RB1CC1 | AC037487.2 | 0.538695 | 1.36E-41 |
| MAPK8 | AC004884.2 | 0.538725 | 1.35E-41 |
| KLHL24 | AC130650.2 | 0.538766 | 1.32E-41 |
| CFLAR | AC008063.1 | 0.538853 | 1.28E-41 |
| EIF2AK2 | AC133644.2 | 0.538862 | 1.27E-41 |
| MAPK8 | AC016590.2 | 0.538984 | 1.21E-41 |
| RB1CC1 | CFLAR-AS1 | 0.539001 | 1.20E-41 |
| PTEN | AC055822.1 | 0.539002 | 1.20E-41 |
| EIF2AK2 | AL022067.1 | 0.539045 | 1.18E-41 |
| ATG12 | AC084117.1 | 0.539131 | 1.14E-41 |
| NAF1 | AC005856.1 | 0.539176 | 1.12E-41 |
| TSC1 | AL445222.1 | 0.539187 | 1.11E-41 |
| CFLAR | ADAMTSL4-AS1 | 0.539207 | 1.11E-41 |
| PIK3C3 | AC026124.2 | 0.539217 | 1.10E-41 |
| NAF1 | AC016831.4 | 0.53922 | 1.10E-41 |
| EIF2AK2 | AC016590.2 | 0.539222 | 1.10E-41 |
| TSC1 | AC006435.2 | 0.539226 | 1.10E-41 |
| MAPK8 | NARF-IT1 | 0.539336 | 1.05E-41 |
| RPS6KB1 | AP005899.1 | 0.539365 | 1.04E-41 |
| NAF1 | AC019080.5 | 0.539388 | 1.03E-41 |
| EIF2AK2 | ITCH-IT1 | 0.539396 | 1.02E-41 |
| PTEN | AC092123.1 | 0.539401 | 1.02E-41 |
| CXCR4 | LINC00926 | 0.539517 | 9.75E-42 |
| RB1CC1 | AC245014.3 | 0.539528 | 9.70E-42 |
| ATG2B | RPS6KA2-IT1 | 0.539555 | 9.60E-42 |
| EIF2AK2 | AC005838.2 | 0.539566 | 9.55E-42 |
| TSC1 | LINC00852 | 0.539639 | 9.27E-42 |
| RPS6KB1 | AC107068.1 | 0.539644 | 9.26E-42 |
| NAF1 | AC002064.2 | 0.539684 | 9.11E-42 |
| WDFY3 | LINC01004 | 0.539684 | 9.11E-42 |
| RB1CC1 | AC010186.3 | 0.539742 | 8.89E-42 |
| ATG12 | AC055822.1 | 0.53979 | 8.72E-42 |
| MAPK8 | AC016542.1 | 0.5398 | 8.68E-42 |
| RB1CC1 | Z82243.1 | 0.539926 | 8.25E-42 |
| ATG2B | AC055822.1 | 0.539939 | 8.20E-42 |
| CFLAR | AF117829.1 | 0.539944 | 8.19E-42 |
| MAPK8 | AC027097.1 | 0.539974 | 8.09E-42 |
| ATG2B | AC011468.1 | 0.540052 | 7.83E-42 |
| TSC1 | THUMPD3-AS1 | 0.540063 | 7.80E-42 |
| CCR2 | MIR155HG | 0.540078 | 7.75E-42 |
| WDFY3 | AC009120.2 | 0.540103 | 7.67E-42 |
| RAB24 | AC073335.2 | 0.540126 | 7.60E-42 |
| BIRC6 | GK-AS1 | 0.540126 | 7.60E-42 |
| FOXO1 | AC100830.2 | 0.540157 | 7.51E-42 |
| CFLAR | AC005838.2 | 0.54021 | 7.35E-42 |
| ATG2B | AC092953.2 | 0.540249 | 7.23E-42 |
| CFLAR | AC009120.3 | 0.540337 | 6.98E-42 |
| NAF1 | AP002336.2 | 0.540343 | 6.96E-42 |
| RB1CC1 | AP000786.1 | 0.540355 | 6.92E-42 |
| KLHL24 | AL731566.1 | 0.540367 | 6.89E-42 |
| MAPK8 | AC253576.2 | 0.540386 | 6.84E-42 |
| RB1CC1 | AL731566.1 | 0.540424 | 6.73E-42 |
| TSC1 | ERVK13-1 | 0.540447 | 6.67E-42 |
| WDFY3 | ATP13A4-AS1 | 0.540463 | 6.62E-42 |
| MAPK8 | AL162724.2 | 0.540515 | 6.49E-42 |
| RB1CC1 | TRAF3IP2-AS1 | 0.540567 | 6.35E-42 |
| PTEN | AC138207.4 | 0.540585 | 6.30E-42 |
| MAPK8 | AC012467.1 | 0.540593 | 6.28E-42 |
| CFLAR | AC063965.1 | 0.540624 | 6.20E-42 |
| PIK3C3 | PWAR6 | 0.540625 | 6.20E-42 |
| RPS6KB1 | CR936218.1 | 0.540628 | 6.19E-42 |
| PIK3C3 | AC110792.3 | 0.540679 | 6.06E-42 |
| PIK3C3 | AL049552.1 | 0.540697 | 6.02E-42 |
| FOXO1 | AC005046.1 | 0.540704 | 6.00E-42 |
| TSC1 | LINC00641 | 0.540712 | 5.98E-42 |
| RB1CC1 | AL136320.1 | 0.540712 | 5.98E-42 |
| CFLAR | AC007038.1 | 0.540734 | 5.93E-42 |
| PTEN | AC090617.5 | 0.540764 | 5.86E-42 |
| PIK3C3 | AC253536.3 | 0.540789 | 5.80E-42 |
| CFLAR | PSMA3-AS1 | 0.540848 | 5.66E-42 |
| TSC1 | AP000692.1 | 0.540873 | 5.60E-42 |
| PTEN | AC004253.1 | 0.540886 | 5.57E-42 |
| MAPK8 | PCBP1-AS1 | 0.540913 | 5.51E-42 |
| KLHL24 | AL049552.1 | 0.540916 | 5.50E-42 |
| CFLAR | AP001178.2 | 0.540932 | 5.47E-42 |
| MAPK8 | AF117829.1 | 0.540942 | 5.45E-42 |
| WDFY3 | AC010542.5 | 0.54095 | 5.43E-42 |
| WDFY3 | AC005479.2 | 0.540963 | 5.40E-42 |
| FOXO1 | AL592148.3 | 0.540988 | 5.34E-42 |
| KLHL24 | AC016590.2 | 0.541017 | 5.28E-42 |
| PIK3C3 | AC090948.3 | 0.54117 | 4.96E-42 |
| BIRC6 | UBE2Q1-AS1 | 0.541211 | 4.88E-42 |
| TP73 | AL357093.2 | 0.541237 | 4.83E-42 |
| ATG2B | TPT1-AS1 | 0.541262 | 4.78E-42 |
| NAF1 | AF129075.1 | 0.541293 | 4.72E-42 |
| CFLAR | AC007038.2 | 0.541408 | 4.50E-42 |
| ATG2B | HIF1A-AS2 | 0.541412 | 4.49E-42 |
| CFLAR | AC005519.1 | 0.541515 | 4.31E-42 |
| MAPK8 | AC010201.2 | 0.54155 | 4.24E-42 |
| NAF1 | AC108727.1 | 0.541571 | 4.21E-42 |
| RB1CC1 | AC008906.1 | 0.541575 | 4.20E-42 |
| ATG4B | AC012615.6 | 0.541628 | 4.11E-42 |
| EIF2AK2 | AC010834.3 | 0.541666 | 4.05E-42 |
| KLHL24 | AC007014.2 | 0.541693 | 4.00E-42 |
| KLHL24 | AP005899.1 | 0.541739 | 3.93E-42 |
| CFLAR | AC018926.2 | 0.541783 | 3.86E-42 |
| ATG12 | SNHG26 | 0.541801 | 3.83E-42 |
| WDFY3 | AC093726.2 | 0.54181 | 3.81E-42 |
| CFLAR | AC053513.1 | 0.541815 | 3.81E-42 |
| NAF1 | AC012557.1 | 0.541818 | 3.80E-42 |
| LAMP1 | AL139384.1 | 0.541828 | 3.79E-42 |
| RB1CC1 | AC008115.3 | 0.541874 | 3.72E-42 |
| RB1CC1 | AC068790.3 | 0.541879 | 3.71E-42 |
| RB1CC1 | AC083843.2 | 0.541888 | 3.69E-42 |
| RB1CC1 | LINC00513 | 0.541893 | 3.69E-42 |
| SIRT1 | AC098484.1 | 0.541895 | 3.68E-42 |
| NAF1 | AC009032.1 | 0.541904 | 3.67E-42 |
| KLHL24 | Z83843.1 | 0.541916 | 3.65E-42 |
| PTEN | AC011442.1 | 0.541918 | 3.65E-42 |
| RB1CC1 | LIMS1-AS1 | 0.541927 | 3.64E-42 |
| ATG12 | AC108010.1 | 0.541994 | 3.54E-42 |
| MAPK8 | AL162724.1 | 0.542004 | 3.52E-42 |
| NAF1 | AP001033.2 | 0.542011 | 3.51E-42 |
| NAF1 | AC058791.1 | 0.542014 | 3.51E-42 |
| NAF1 | PWAR6 | 0.54204 | 3.47E-42 |
| KLHL24 | AC005856.1 | 0.542073 | 3.42E-42 |
| RPS6KB1 | AC090948.2 | 0.542083 | 3.41E-42 |
| FOXO1 | RAP2C-AS1 | 0.542116 | 3.36E-42 |
| RB1CC1 | AC087752.4 | 0.542171 | 3.29E-42 |
| BIRC6 | AL159169.2 | 0.542193 | 3.26E-42 |
| PTEN | AC124319.1 | 0.542213 | 3.23E-42 |
| MAPK8 | RHOA-IT1 | 0.542229 | 3.21E-42 |
| FOXO1 | AC130650.2 | 0.542236 | 3.20E-42 |
| MAPK8 | SMC5-AS1 | 0.542326 | 3.09E-42 |
| FOXO1 | AC002064.2 | 0.542361 | 3.04E-42 |
| NAF1 | SDCBP2-AS1 | 0.542369 | 3.03E-42 |
| ATG12 | AC074033.1 | 0.542404 | 2.99E-42 |
| WDFY3 | AC055822.1 | 0.542473 | 2.90E-42 |
| NAF1 | AP001469.2 | 0.542514 | 2.86E-42 |
| MAPK8 | AC048344.4 | 0.542584 | 2.78E-42 |
| RB1CC1 | ADNP-AS1 | 0.542597 | 2.76E-42 |
| PIK3C3 | AC027097.2 | 0.542599 | 2.76E-42 |
| PPP1R15A | AC020916.1 | 0.542609 | 2.75E-42 |
| RB1CC1 | AC127024.4 | 0.542622 | 2.73E-42 |
| RB1CC1 | AC063965.1 | 0.54266 | 2.69E-42 |
| EIF2AK2 | AP005899.1 | 0.542728 | 2.62E-42 |
| WDFY3 | KCCAT333 | 0.54279 | 2.55E-42 |
| CFLAR | AL031717.1 | 0.542894 | 2.44E-42 |
| MAPK8 | MALAT1 | 0.542904 | 2.43E-42 |
| CXCR4 | LINC00426 | 0.542924 | 2.41E-42 |
| PTEN | AL354696.1 | 0.542949 | 2.39E-42 |
| MAPK8 | AC009032.1 | 0.542979 | 2.36E-42 |
| ATG12 | AL137003.2 | 0.54301 | 2.33E-42 |
| NAF1 | AC068790.5 | 0.543055 | 2.29E-42 |
| NAF1 | SMC5-AS1 | 0.54308 | 2.26E-42 |
| TSC1 | AP001486.2 | 0.54309 | 2.25E-42 |
| EIF2AK2 | NEAT1 | 0.5431 | 2.24E-42 |
| ATG2B | AC010245.2 | 0.543122 | 2.22E-42 |
| CFLAR | SAP30L-AS1 | 0.543167 | 2.18E-42 |
| ATG2B | LINC02035 | 0.543169 | 2.18E-42 |
| EIF2AK2 | AL031670.1 | 0.543209 | 2.14E-42 |
| MAPK8 | AC090198.1 | 0.543241 | 2.12E-42 |
| KLHL24 | AC012467.1 | 0.543246 | 2.11E-42 |
| CFLAR | LINC00894 | 0.54326 | 2.10E-42 |
| PTEN | AC027097.1 | 0.543275 | 2.09E-42 |
| NAF1 | N4BP2L2-IT2 | 0.543276 | 2.09E-42 |
| RPS6KB1 | HCG18 | 0.543299 | 2.07E-42 |
| TSC1 | AC020915.2 | 0.543366 | 2.01E-42 |
| RB1CC1 | AP005899.1 | 0.543367 | 2.01E-42 |
| EIF2AK2 | AC006059.1 | 0.543396 | 1.99E-42 |
| PIK3C3 | AC020913.3 | 0.5434 | 1.98E-42 |
| PIK3C3 | PVT1 | 0.543404 | 1.98E-42 |
| TSC1 | AP001107.4 | 0.54341 | 1.97E-42 |
| EIF2AK2 | AC116366.1 | 0.543421 | 1.96E-42 |
| RB1CC1 | EBLN3P | 0.543488 | 1.91E-42 |
| CFLAR | AC005920.2 | 0.543623 | 1.81E-42 |
| MAPK8 | AL359697.1 | 0.543637 | 1.80E-42 |
| EIF2AK2 | AC130895.1 | 0.543651 | 1.79E-42 |
| PTEN | AC025171.4 | 0.543655 | 1.78E-42 |
| EIF2AK2 | ALMS1-IT1 | 0.54371 | 1.74E-42 |
| PTEN | AC093110.1 | 0.543725 | 1.73E-42 |
| ATG12 | AC002044.1 | 0.543761 | 1.71E-42 |
| NAF1 | AL133445.2 | 0.543775 | 1.70E-42 |
| LAMP1 | AC069544.1 | 0.543782 | 1.69E-42 |
| FOXO1 | AC006270.1 | 0.543788 | 1.69E-42 |
| CASP1 | LINC01094 | 0.543805 | 1.68E-42 |
| ATG16L2 | AC108134.3 | 0.543818 | 1.67E-42 |
| PIK3C3 | AC090181.2 | 0.543899 | 1.61E-42 |
| CFLAR | AC019080.5 | 0.543928 | 1.59E-42 |
| FOXO1 | GMDS-DT | 0.543946 | 1.58E-42 |
| ATG2B | AC087752.3 | 0.543961 | 1.57E-42 |
| ATG2B | AP001107.4 | 0.543969 | 1.57E-42 |
| PIK3C3 | AC008115.3 | 0.543978 | 1.56E-42 |
| PTEN | AC005519.1 | 0.543995 | 1.55E-42 |
| EIF2AK2 | BTBD9-AS1 | 0.544035 | 1.53E-42 |
| BIRC6 | AC008982.2 | 0.544036 | 1.52E-42 |
| ATG4B | SNHG12 | 0.544051 | 1.52E-42 |
| PIK3C3 | AC099343.2 | 0.544074 | 1.50E-42 |
| RB1CC1 | AC004884.2 | 0.544104 | 1.48E-42 |
| NAF1 | AC005034.5 | 0.544167 | 1.44E-42 |
| KLHL24 | AC020913.3 | 0.544207 | 1.42E-42 |
| NAF1 | AC008906.1 | 0.544227 | 1.41E-42 |
| EIF2AK2 | AC087286.1 | 0.544254 | 1.39E-42 |
| MAPK8 | PAXBP1-AS1 | 0.54427 | 1.38E-42 |
| CFLAR | AP005131.7 | 0.544303 | 1.36E-42 |
| PIK3C3 | PCBP1-AS1 | 0.544344 | 1.34E-42 |
| RPS6KB1 | AP001033.2 | 0.544344 | 1.34E-42 |
| EIF2AK2 | AC007216.3 | 0.544361 | 1.33E-42 |
| ATG2B | AC037198.1 | 0.544385 | 1.32E-42 |
| PTEN | AL157838.1 | 0.544498 | 1.26E-42 |
| EIF2AK2 | AL049552.1 | 0.544621 | 1.20E-42 |
| KLHL24 | PCBP1-AS1 | 0.544627 | 1.19E-42 |
| PTEN | AC097641.2 | 0.544661 | 1.18E-42 |
| WDFY3 | ARHGAP31-AS1 | 0.544681 | 1.17E-42 |
| NAF1 | KIF26B-AS1 | 0.544686 | 1.16E-42 |
| IFNG | AC015911.3 | 0.544703 | 1.16E-42 |
| CXCR4 | AC018755.4 | 0.544714 | 1.15E-42 |
| MAPK8 | AL136320.1 | 0.544733 | 1.14E-42 |
| MAPK8 | AC096741.1 | 0.544771 | 1.12E-42 |
| ATG16L2 | AC005519.1 | 0.544778 | 1.12E-42 |
| CFLAR | N4BP2L2-IT2 | 0.544785 | 1.12E-42 |
| NLRC4 | AC026369.3 | 0.544857 | 1.08E-42 |
| CFLAR | AC024075.3 | 0.544898 | 1.07E-42 |
| RPS6KB1 | AC019080.5 | 0.54491 | 1.06E-42 |
| NAF1 | AC087284.1 | 0.544926 | 1.05E-42 |
| EIF2AK2 | AC087286.4 | 0.544933 | 1.05E-42 |
| CFLAR | AC005632.2 | 0.544954 | 1.04E-42 |
| ATG4B | AL135999.1 | 0.544956 | 1.04E-42 |
| PIK3C3 | LINC-PINT | 0.544959 | 1.04E-42 |
| EIF2AK2 | AC012170.2 | 0.544964 | 1.04E-42 |
| ATG2B | UBE2Q1-AS1 | 0.545003 | 1.02E-42 |
| RB1CC1 | N4BP2L2-IT2 | 0.545024 | 1.01E-42 |
| ATG2B | AC083949.1 | 0.545085 | 9.87E-43 |
| FOXO1 | AL049869.3 | 0.54509 | 9.85E-43 |
| ATG12 | AC011468.1 | 0.545109 | 9.77E-43 |
| MAPK8 | AC012557.1 | 0.54512 | 9.73E-43 |
| BIRC6 | TAPT1-AS1 | 0.545181 | 9.48E-43 |
| RPS6KB1 | AC108010.1 | 0.545199 | 9.41E-43 |
| PIK3C3 | AC090739.1 | 0.545205 | 9.39E-43 |
| NAF1 | AC090579.1 | 0.545212 | 9.36E-43 |
| BIRC6 | GEMIN7-AS1 | 0.54524 | 9.25E-43 |
| PIK3C3 | AC245014.3 | 0.545272 | 9.13E-43 |
| ATG2B | AL133371.2 | 0.545274 | 9.12E-43 |
| NAF1 | AC007216.3 | 0.545278 | 9.11E-43 |
| RB1CC1 | AC004918.3 | 0.545307 | 9.00E-43 |
| RPS6KB1 | ALMS1-IT1 | 0.545333 | 8.90E-43 |
| BIRC6 | DGCR11 | 0.545334 | 8.90E-43 |
| PTEN | GAS8-AS1 | 0.545351 | 8.84E-43 |
| EIF2AK2 | AC073487.1 | 0.545364 | 8.79E-43 |
| EIF2AK2 | AC096992.2 | 0.54539 | 8.70E-43 |
| EIF2AK2 | AC022211.1 | 0.545408 | 8.63E-43 |
| FOXO1 | Z83843.1 | 0.545412 | 8.62E-43 |
| TSC1 | GEMIN7-AS1 | 0.545456 | 8.46E-43 |
| KLHL24 | NPTN-IT1 | 0.545506 | 8.28E-43 |
| KLHL24 | AC090181.2 | 0.54551 | 8.27E-43 |
| CFLAR | ANKRD44-IT1 | 0.54557 | 8.07E-43 |
| NAF1 | AC067852.3 | 0.545658 | 7.78E-43 |
| CFLAR | AP003486.1 | 0.545669 | 7.74E-43 |
| ATG12 | AP001458.1 | 0.545685 | 7.69E-43 |
| NAF1 | ANKRD44-IT1 | 0.545691 | 7.67E-43 |
| ATG2B | ZNF460-AS1 | 0.545724 | 7.57E-43 |
| KLHL24 | AC127024.4 | 0.545752 | 7.48E-43 |
| CCR2 | AC243960.1 | 0.545817 | 7.28E-43 |
| CFLAR | AL353804.1 | 0.545844 | 7.20E-43 |
| KLHL24 | AL359697.1 | 0.545845 | 7.19E-43 |
| KLHL24 | AC027097.2 | 0.545858 | 7.16E-43 |
| RB1CC1 | AC007216.4 | 0.545877 | 7.10E-43 |
| PIK3C3 | MAL2-AS1 | 0.545892 | 7.06E-43 |
| CFLAR | AC138207.4 | 0.545894 | 7.05E-43 |
| KLHL24 | AL590723.1 | 0.545929 | 6.95E-43 |
| ATG12 | AL391001.1 | 0.54593 | 6.94E-43 |
| ATG2B | AP001628.1 | 0.545955 | 6.87E-43 |
| NAF1 | AL513008.1 | 0.545998 | 6.75E-43 |
| CFLAR | AC090181.2 | 0.545998 | 6.75E-43 |
| NAF1 | AC073487.1 | 0.546004 | 6.73E-43 |
| PTEN | NORAD | 0.546021 | 6.69E-43 |
| RB1CC1 | AC019080.5 | 0.546037 | 6.64E-43 |
| EIF2AK2 | AC007038.1 | 0.546043 | 6.63E-43 |
| KLHL24 | AC073487.1 | 0.546045 | 6.62E-43 |
| NAF1 | AL359076.1 | 0.546047 | 6.61E-43 |
| ATG12 | MCM3AP-AS1 | 0.546127 | 6.40E-43 |
| EIF2AK2 | AC090739.1 | 0.546128 | 6.40E-43 |
| ATG2B | AL157838.1 | 0.546149 | 6.34E-43 |
| ATG12 | AL117336.2 | 0.546202 | 6.20E-43 |
| TSC1 | AC116366.1 | 0.546233 | 6.12E-43 |
| TSC1 | ZKSCAN2-DT | 0.546272 | 6.02E-43 |
| KLHL24 | LINC00513 | 0.546276 | 6.01E-43 |
| CFLAR | AC015871.3 | 0.546302 | 5.95E-43 |
| TSC1 | AC245884.8 | 0.546368 | 5.79E-43 |
| BIRC6 | AC004241.3 | 0.546407 | 5.69E-43 |
| BIRC6 | AC025171.4 | 0.546449 | 5.59E-43 |
| KLHL24 | AC098851.1 | 0.546477 | 5.53E-43 |
| PTEN | AL359715.3 | 0.546487 | 5.51E-43 |
| EIF2AK2 | SDCBP2-AS1 | 0.546494 | 5.49E-43 |
| ATG12 | AL031670.1 | 0.546515 | 5.44E-43 |
| GOPC | AL513550.1 | 0.546526 | 5.42E-43 |
| ATG2B | SH3BP5-AS1 | 0.546547 | 5.37E-43 |
| CFLAR | FTX | 0.546564 | 5.33E-43 |
| PTEN | C1RL-AS1 | 0.546581 | 5.30E-43 |
| RB1CC1 | AP000766.1 | 0.546593 | 5.27E-43 |
| TSC1 | AL049552.1 | 0.546625 | 5.20E-43 |
| PIK3C3 | AL359076.1 | 0.546643 | 5.16E-43 |
| FOXO1 | AC008770.3 | 0.546644 | 5.16E-43 |
| ATG16L2 | AP006621.4 | 0.546675 | 5.09E-43 |
| ATG2B | TAPT1-AS1 | 0.546703 | 5.03E-43 |
| PIK3C3 | ALG13-AS1 | 0.546719 | 5.00E-43 |
| ATG16L2 | AP006284.1 | 0.546744 | 4.95E-43 |
| IFNG | LINC00426 | 0.546816 | 4.80E-43 |
| EIF2AK2 | AL359076.1 | 0.546856 | 4.72E-43 |
| ATG12 | AC083949.1 | 0.546856 | 4.72E-43 |
| FOXO1 | AC021078.1 | 0.546903 | 4.63E-43 |
| CFLAR | INE1 | 0.546925 | 4.59E-43 |
| WDFY3 | PAXIP1-AS2 | 0.546981 | 4.48E-43 |
| PIK3C3 | AC002553.2 | 0.546982 | 4.48E-43 |
| PIK3C3 | AC108727.1 | 0.54699 | 4.46E-43 |
| TSC1 | TPT1-AS1 | 0.546994 | 4.46E-43 |
| PIK3C3 | AL133330.1 | 0.547 | 4.44E-43 |
| MAPK8 | AC022973.3 | 0.547044 | 4.36E-43 |
| KLHL24 | AC011477.3 | 0.547057 | 4.34E-43 |
| WDFY3 | AL109761.1 | 0.547098 | 4.27E-43 |
| MAPK8 | AP000866.6 | 0.547174 | 4.13E-43 |
| CFLAR | AF129075.1 | 0.547217 | 4.06E-43 |
| TSC1 | AC015871.3 | 0.547219 | 4.06E-43 |
| ATG16L2 | LINC00106 | 0.547224 | 4.05E-43 |
| NAF1 | AC096586.2 | 0.547271 | 3.97E-43 |
| RPS6KB1 | AC124283.3 | 0.54731 | 3.90E-43 |
| TSC1 | AC108449.2 | 0.54732 | 3.89E-43 |
| TSC1 | FLNB-AS1 | 0.547358 | 3.83E-43 |
| ATG2B | AL356356.1 | 0.547398 | 3.76E-43 |
| KLHL24 | A2M-AS1 | 0.547415 | 3.74E-43 |
| FOXO1 | AC096741.1 | 0.547447 | 3.69E-43 |
| ATG16L2 | ARHGAP27P1-BPTFP1-KPNA2P3 | 0.547538 | 3.55E-43 |
| ATG2B | OSMR-AS1 | 0.547541 | 3.54E-43 |
| NAF1 | AC012170.2 | 0.54755 | 3.53E-43 |
| EIF2AK2 | AL049840.1 | 0.547567 | 3.51E-43 |
| FOXO1 | AL606834.2 | 0.547571 | 3.50E-43 |
| ATG4B | AC007292.1 | 0.547642 | 3.40E-43 |
| MAPK8 | AC007546.1 | 0.547644 | 3.39E-43 |
| NAF1 | ITCH-IT1 | 0.547649 | 3.39E-43 |
| FOXO1 | RHOA-IT1 | 0.547664 | 3.37E-43 |
| ATG2B | AC113139.1 | 0.547681 | 3.34E-43 |
| RPS6KB1 | SOS1-IT1 | 0.54772 | 3.29E-43 |
| WDFY3 | AC093484.4 | 0.547777 | 3.21E-43 |
| NAF1 | LAMC1-AS1 | 0.547794 | 3.19E-43 |
| PIK3C3 | AC012557.1 | 0.547794 | 3.19E-43 |
| CFLAR | AC108727.1 | 0.547797 | 3.18E-43 |
| MAPK8 | AC005920.2 | 0.54782 | 3.15E-43 |
| PTEN | AC002128.1 | 0.547865 | 3.09E-43 |
| ATG16L2 | AC008760.1 | 0.547874 | 3.08E-43 |
| RB1CC1 | AF178030.1 | 0.547914 | 3.03E-43 |
| TSC1 | AP003486.1 | 0.547933 | 3.01E-43 |
| BIRC6 | GABPB1-AS1 | 0.547936 | 3.00E-43 |
| FOXO1 | MBNL1-AS1 | 0.547936 | 3.00E-43 |
| EIF2AK2 | AC010186.3 | 0.547951 | 2.99E-43 |
| TSC1 | AC005674.2 | 0.547954 | 2.98E-43 |
| MAPK8 | AC138393.3 | 0.547986 | 2.94E-43 |
| PIK3C3 | AL162724.2 | 0.548009 | 2.91E-43 |
| CFLAR | AL356356.1 | 0.548038 | 2.88E-43 |
| TSC1 | GARS-DT | 0.548089 | 2.82E-43 |
| PIK3C3 | AC124312.5 | 0.54813 | 2.77E-43 |
| KLHL24 | AL162724.2 | 0.548185 | 2.71E-43 |
| MAPK8 | AC006017.1 | 0.548312 | 2.57E-43 |
| PTEN | AC112496.1 | 0.548334 | 2.54E-43 |
| KLHL24 | AC124283.3 | 0.548337 | 2.54E-43 |
| TSC1 | AL049840.2 | 0.54835 | 2.52E-43 |
| KLHL24 | AC068790.3 | 0.548351 | 2.52E-43 |
| EIF2AK2 | AC068792.1 | 0.548355 | 2.52E-43 |
| NAF1 | AC004918.3 | 0.548362 | 2.51E-43 |
| PIK3C3 | AC010186.3 | 0.548368 | 2.51E-43 |
| RB1CC1 | AL109614.1 | 0.54839 | 2.48E-43 |
| KLHL24 | AC008124.1 | 0.548398 | 2.48E-43 |
| MAPK8 | KLF7-IT1 | 0.548413 | 2.46E-43 |
| ATG12 | AL157392.4 | 0.548477 | 2.39E-43 |
| KLHL24 | SAMD12-AS1 | 0.548477 | 2.39E-43 |
| RB1CC1 | AC010761.3 | 0.548517 | 2.35E-43 |
| FOXO1 | AC010761.3 | 0.548527 | 2.34E-43 |
| NAF1 | AC010536.2 | 0.548536 | 2.34E-43 |
| BIRC6 | AC073896.3 | 0.548561 | 2.31E-43 |
| RPS6KB1 | AC004223.3 | 0.548569 | 2.30E-43 |
| RB1CC1 | AC108449.2 | 0.548578 | 2.29E-43 |
| KLHL24 | OIP5-AS1 | 0.548602 | 2.27E-43 |
| KLHL24 | ITCH-IT1 | 0.548641 | 2.23E-43 |
| MAPK8 | AC010536.2 | 0.548659 | 2.22E-43 |
| NAF1 | AC092611.2 | 0.548731 | 2.15E-43 |
| MAPK8 | AC004908.2 | 0.548751 | 2.13E-43 |
| NAF1 | AC130895.1 | 0.548753 | 2.13E-43 |
| PIK3C3 | AC087286.4 | 0.548767 | 2.12E-43 |
| ATG16L2 | AL360181.2 | 0.548805 | 2.09E-43 |
| ATG16L2 | MIR600HG | 0.548834 | 2.06E-43 |
| EIF2AK2 | AC024075.3 | 0.54884 | 2.06E-43 |
| EIF2AK2 | AC124319.1 | 0.548878 | 2.02E-43 |
| PIK3C3 | AC005856.1 | 0.548944 | 1.97E-43 |
| KLHL24 | AL391834.1 | 0.548973 | 1.94E-43 |
| ATG2B | AC009318.2 | 0.548973 | 1.94E-43 |
| KLHL24 | MUC20-OT1 | 0.549002 | 1.92E-43 |
| EIF2AK2 | AL359962.2 | 0.549004 | 1.92E-43 |
| RPS6KB1 | AL139407.1 | 0.54901 | 1.91E-43 |
| ATG2B | MIR181A2HG | 0.549039 | 1.89E-43 |
| KLHL24 | ANKRD10-IT1 | 0.549119 | 1.83E-43 |
| NAF1 | AL049869.3 | 0.549123 | 1.83E-43 |
| FOXO1 | AC018752.1 | 0.549163 | 1.79E-43 |
| MAPK8 | AC008770.3 | 0.549177 | 1.78E-43 |
| TSC1 | AC006042.1 | 0.549186 | 1.78E-43 |
| NAF1 | NEAT1 | 0.549212 | 1.76E-43 |
| EIF2AK2 | LIMS1-AS1 | 0.549234 | 1.74E-43 |
| EIF2AK2 | AL117381.1 | 0.549255 | 1.73E-43 |
| BIRC6 | TBILA | 0.549348 | 1.66E-43 |
| PIK3C3 | AC092279.1 | 0.549414 | 1.61E-43 |
| RPS6KB1 | AC015849.3 | 0.549416 | 1.61E-43 |
| ATG12 | AC005540.1 | 0.549444 | 1.59E-43 |
| WDFY3 | AC026368.1 | 0.549467 | 1.58E-43 |
| RB1CC1 | AC124319.2 | 0.549492 | 1.56E-43 |
| EIF2AK2 | AC007684.1 | 0.549501 | 1.56E-43 |
| PIK3C3 | AC022973.3 | 0.549541 | 1.53E-43 |
| WDFY3 | GEMIN7-AS1 | 0.549636 | 1.47E-43 |
| EIF2AK2 | AC068790.5 | 0.549638 | 1.47E-43 |
| MAPK8 | Z68871.1 | 0.549691 | 1.44E-43 |
| NAF1 | AC007684.1 | 0.549767 | 1.39E-43 |
| BIRC6 | AC004067.1 | 0.549783 | 1.38E-43 |
| TSC1 | AC025178.1 | 0.549784 | 1.38E-43 |
| EIF2AK2 | AL353804.1 | 0.549815 | 1.36E-43 |
| CFLAR | Z68871.1 | 0.549846 | 1.35E-43 |
| TSC1 | ANKRD10-IT1 | 0.549855 | 1.34E-43 |
| EIF2AK2 | AL157392.3 | 0.5499 | 1.32E-43 |
| ATG12 | AC011472.4 | 0.549939 | 1.29E-43 |
| PIK3C3 | AC130895.1 | 0.549969 | 1.28E-43 |
| CFLAR | AL513365.2 | 0.549971 | 1.28E-43 |
| PIK3C3 | AL513365.2 | 0.549998 | 1.26E-43 |
| WDFY3 | AC008870.2 | 0.550071 | 1.22E-43 |
| CFLAR | CR936218.1 | 0.550156 | 1.18E-43 |
| MAPK8 | AC093788.1 | 0.550163 | 1.18E-43 |
| EIF2AK2 | AL157402.2 | 0.550169 | 1.17E-43 |
| NAF1 | AP001381.1 | 0.550207 | 1.16E-43 |
| ATG2B | AC093110.1 | 0.550276 | 1.12E-43 |
| PIK3C3 | AC007038.2 | 0.550292 | 1.11E-43 |
| RB1CC1 | AC107068.1 | 0.550308 | 1.11E-43 |
| PTEN | AC245884.8 | 0.550344 | 1.09E-43 |
| ATG16L2 | ASMTL-AS1 | 0.550373 | 1.08E-43 |
| CFLAR | AC127024.4 | 0.550398 | 1.07E-43 |
| RB1CC1 | AC087286.4 | 0.550429 | 1.05E-43 |
| EIF2AK2 | AC083843.2 | 0.5505 | 1.02E-43 |
| ST13 | AL021707.2 | 0.550513 | 1.02E-43 |
| ATG4B | AC010973.2 | 0.550514 | 1.01E-43 |
| EIF2AK2 | LINC-PINT | 0.550536 | 1.01E-43 |
| RPS6KB1 | NPTN-IT1 | 0.550557 | 9.97E-44 |
| KLHL24 | AC110792.3 | 0.55064 | 9.62E-44 |
| PIK3C3 | AC007684.1 | 0.550686 | 9.43E-44 |
| CFLAR | RFX3-AS1 | 0.550715 | 9.32E-44 |
| RB1CC1 | AC005838.2 | 0.550752 | 9.18E-44 |
| NAF1 | AC108010.1 | 0.550779 | 9.07E-44 |
| RB1CC1 | KLF7-IT1 | 0.55078 | 9.07E-44 |
| PIK3C3 | AF117829.1 | 0.550797 | 9.00E-44 |
| NAF1 | AL365277.1 | 0.550802 | 8.98E-44 |
| EIF2AK2 | AC124283.3 | 0.550829 | 8.88E-44 |
| CFLAR | AC007216.3 | 0.550844 | 8.83E-44 |
| KLHL24 | Z82243.1 | 0.550867 | 8.74E-44 |
| MAPK8 | AL133445.2 | 0.550884 | 8.68E-44 |
| RB1CC1 | AC090579.1 | 0.550892 | 8.65E-44 |
| KLHL24 | AL133330.1 | 0.55091 | 8.58E-44 |
| RB1CC1 | AC025917.1 | 0.550929 | 8.52E-44 |
| CFLAR | AC002128.1 | 0.550939 | 8.48E-44 |
| KLHL24 | AC024933.1 | 0.550949 | 8.44E-44 |
| TSC2 | AL031600.1 | 0.55096 | 8.40E-44 |
| RB1CC1 | AC020913.3 | 0.551044 | 8.11E-44 |
| FOXO1 | AC012170.2 | 0.551144 | 7.77E-44 |
| NAF1 | AP005131.7 | 0.551187 | 7.63E-44 |
| ATG16L2 | AC129510.1 | 0.551221 | 7.52E-44 |
| CFLAR | AL117381.1 | 0.551271 | 7.37E-44 |
| RB1CC1 | AC068790.5 | 0.551278 | 7.34E-44 |
| ATG12 | LAMC1-AS1 | 0.551388 | 7.01E-44 |
| NAF1 | AC087286.1 | 0.551409 | 6.95E-44 |
| ATG12 | AC009120.3 | 0.551441 | 6.85E-44 |
| FOXO1 | AC053513.1 | 0.551461 | 6.79E-44 |
| FOXO1 | AC098851.1 | 0.551482 | 6.73E-44 |
| NAF1 | AC087286.4 | 0.551584 | 6.45E-44 |
| RB1CC1 | MCM3AP-AS1 | 0.551611 | 6.38E-44 |
| ATG12 | AC005104.1 | 0.551677 | 6.20E-44 |
| ATG2B | AC087222.1 | 0.551716 | 6.10E-44 |
| MAPK8 | AC234775.3 | 0.55173 | 6.06E-44 |
| RB1CC1 | AC053513.1 | 0.55175 | 6.01E-44 |
| CFLAR | AL049840.5 | 0.551759 | 5.99E-44 |
| PIK3C3 | AC138932.5 | 0.551786 | 5.92E-44 |
| KLHL24 | AC018752.1 | 0.551808 | 5.86E-44 |
| CFLAR | AL645568.1 | 0.551817 | 5.84E-44 |
| NAF1 | PAXBP1-AS1 | 0.551846 | 5.77E-44 |
| PIK3C3 | AL513008.1 | 0.551859 | 5.74E-44 |
| BIRC6 | MAGI2-AS3 | 0.551862 | 5.73E-44 |
| CFLAR | Z83843.1 | 0.551886 | 5.67E-44 |
| EIF2AK2 | Z83843.1 | 0.551997 | 5.41E-44 |
| ATG2B | AL158212.3 | 0.552002 | 5.40E-44 |
| LAMP1 | LINC00261 | 0.552098 | 5.18E-44 |
| CFLAR | AP003392.1 | 0.55212 | 5.14E-44 |
| FOXO1 | AL596325.2 | 0.552191 | 4.98E-44 |
| CFLAR | RHOA-IT1 | 0.5522 | 4.96E-44 |
| PIK3C3 | AC018926.3 | 0.552218 | 4.93E-44 |
| KLHL24 | AC006017.1 | 0.552232 | 4.90E-44 |
| IL24 | AC079949.1 | 0.552262 | 4.84E-44 |
| MAPK8 | AC020915.2 | 0.552299 | 4.76E-44 |
| PIK3C3 | LAMC1-AS1 | 0.552317 | 4.72E-44 |
| RB1CC1 | DLEU2 | 0.552342 | 4.67E-44 |
| EIF2AK2 | AC090579.1 | 0.552449 | 4.47E-44 |
| NAF1 | AP001432.1 | 0.552461 | 4.44E-44 |
| TSC1 | AP001469.2 | 0.552466 | 4.43E-44 |
| ATG12 | OCIAD1-AS1 | 0.552612 | 4.17E-44 |
| ATG12 | AC253536.3 | 0.552625 | 4.14E-44 |
| ATG12 | AC090425.2 | 0.55272 | 3.98E-44 |
| KLHL24 | AL359962.2 | 0.55272 | 3.98E-44 |
| KLHL24 | AC018926.3 | 0.552738 | 3.95E-44 |
| PIK3C3 | ANKRD44-IT1 | 0.552752 | 3.93E-44 |
| MAPK8 | AC090948.1 | 0.552775 | 3.89E-44 |
| NAF1 | AC107068.1 | 0.55279 | 3.86E-44 |
| PIK3C3 | AC016831.4 | 0.552847 | 3.77E-44 |
| EIF2AK2 | AP001429.1 | 0.552904 | 3.68E-44 |
| EIF2AK2 | AF178030.1 | 0.55296 | 3.59E-44 |
| ATG2B | AC016394.1 | 0.552987 | 3.55E-44 |
| TSC1 | MCM3AP-AS1 | 0.552994 | 3.54E-44 |
| KLHL24 | AP001429.1 | 0.553088 | 3.40E-44 |
| ATG12 | AC053527.1 | 0.553096 | 3.39E-44 |
| KLHL24 | AP002907.1 | 0.55312 | 3.36E-44 |
| TSC1 | AC011468.1 | 0.55315 | 3.31E-44 |
| RB1CC1 | AL354989.1 | 0.553242 | 3.19E-44 |
| TSC1 | AC010201.2 | 0.553268 | 3.15E-44 |
| CFLAR | AL049840.2 | 0.55327 | 3.15E-44 |
| RB1CC1 | NPTN-IT1 | 0.553289 | 3.12E-44 |
| CFLAR | SMC5-AS1 | 0.553338 | 3.06E-44 |
| EIF2AK2 | Z82243.1 | 0.553393 | 2.99E-44 |
| RB1CC1 | AC108727.1 | 0.553397 | 2.98E-44 |
| MAPK8 | PSPC1-AS2 | 0.553424 | 2.95E-44 |
| RB1CC1 | AL157402.2 | 0.55343 | 2.94E-44 |
| ATG12 | AC026356.1 | 0.553465 | 2.90E-44 |
| PTEN | AP001628.1 | 0.553491 | 2.86E-44 |
| RB1CC1 | LINC-PINT | 0.553517 | 2.83E-44 |
| CFLAR | AC138956.1 | 0.553572 | 2.77E-44 |
| WDFY3 | AC025165.4 | 0.553578 | 2.76E-44 |
| ATG2B | ZNF32-AS2 | 0.553605 | 2.73E-44 |
| PTEN | AL450263.1 | 0.553607 | 2.73E-44 |
| MAPK8 | AL353804.1 | 0.553624 | 2.71E-44 |
| RB1CC1 | FTX | 0.553645 | 2.68E-44 |
| KLHL24 | SMC5-AS1 | 0.553659 | 2.67E-44 |
| ATG2B | AC129510.1 | 0.553661 | 2.66E-44 |
| MAPK8 | AC108727.1 | 0.553711 | 2.61E-44 |
| CFLAR | AC073487.1 | 0.553711 | 2.61E-44 |
| EIF2AK2 | AL049869.3 | 0.5538 | 2.51E-44 |
| ATG16L2 | LENG8-AS1 | 0.55385 | 2.46E-44 |
| TSC1 | AC015813.1 | 0.553863 | 2.44E-44 |
| MAPK8 | ALG13-AS1 | 0.553909 | 2.40E-44 |
| TSC1 | AC127024.4 | 0.553915 | 2.39E-44 |
| EIF2AK2 | AC245014.3 | 0.553954 | 2.35E-44 |
| RPS6KB1 | AC127024.4 | 0.553965 | 2.34E-44 |
| ATG2B | AC008537.2 | 0.553994 | 2.31E-44 |
| NAF1 | AL163051.2 | 0.554031 | 2.27E-44 |
| PIK3C3 | AC012170.2 | 0.554031 | 2.27E-44 |
| EIF2AK2 | AC058791.1 | 0.554096 | 2.21E-44 |
| CFLAR | AC124319.2 | 0.554104 | 2.20E-44 |
| FOXO1 | NPTN-IT1 | 0.554118 | 2.19E-44 |
| NAF1 | AL513365.2 | 0.55415 | 2.16E-44 |
| ATG2B | AC010168.2 | 0.55419 | 2.12E-44 |
| NAF1 | AL158166.2 | 0.554198 | 2.12E-44 |
| PTEN | AC011676.1 | 0.554206 | 2.11E-44 |
| TSC1 | CAPN10-DT | 0.554221 | 2.10E-44 |
| PIK3C3 | AL365277.1 | 0.554237 | 2.08E-44 |
| MAPK8 | AC078778.1 | 0.554243 | 2.08E-44 |
| PIK3C3 | AC073651.1 | 0.554276 | 2.05E-44 |
| NAF1 | AL049840.1 | 0.55433 | 2.00E-44 |
| NAF1 | AL136115.2 | 0.554334 | 2.00E-44 |
| NAF1 | GMDS-DT | 0.554422 | 1.92E-44 |
| ATG2B | AC027097.1 | 0.554463 | 1.89E-44 |
| ATG16L2 | AC006042.1 | 0.554466 | 1.89E-44 |
| ATG12 | NDUFV2-AS1 | 0.554505 | 1.86E-44 |
| KLHL24 | AC004832.5 | 0.554508 | 1.85E-44 |
| NLRC4 | LINC01094 | 0.554509 | 1.85E-44 |
| ATG12 | AC009948.1 | 0.554522 | 1.84E-44 |
| ATG16L2 | AP006621.2 | 0.554563 | 1.81E-44 |
| RB1CC1 | AP002336.2 | 0.554656 | 1.74E-44 |
| EIF2AK2 | AC092801.1 | 0.554681 | 1.72E-44 |
| PIK3C3 | AC096586.2 | 0.554699 | 1.71E-44 |
| ATG12 | AL031716.1 | 0.554741 | 1.68E-44 |
| PIK3C3 | AC002064.2 | 0.554753 | 1.67E-44 |
| PIK3C3 | AC068790.5 | 0.554801 | 1.64E-44 |
| ATG2B | AC053527.1 | 0.554833 | 1.61E-44 |
| EIF2AK2 | AC011939.2 | 0.554881 | 1.58E-44 |
| EIF2AK2 | AC087286.2 | 0.55489 | 1.57E-44 |
| PIK3C3 | AC097376.2 | 0.554899 | 1.57E-44 |
| PIK3C3 | NUTM2B-AS1 | 0.554901 | 1.57E-44 |
| BIRC6 | AL158166.1 | 0.554908 | 1.56E-44 |
| RB1CC1 | AC007038.1 | 0.55492 | 1.55E-44 |
| CFLAR | LINC00852 | 0.554961 | 1.53E-44 |
| MAPK8 | AC018926.3 | 0.554983 | 1.51E-44 |
| PTEN | AC016727.1 | 0.554987 | 1.51E-44 |
| FAS | AL157394.1 | 0.555036 | 1.48E-44 |
| EIF2AK2 | AC013403.2 | 0.555059 | 1.46E-44 |
| PIK3C3 | AC073487.1 | 0.555077 | 1.45E-44 |
| PIK3C3 | AC058791.1 | 0.555077 | 1.45E-44 |
| ATG2B | AC005674.2 | 0.555083 | 1.45E-44 |
| EIF2AK2 | RFX3-AS1 | 0.555101 | 1.44E-44 |
| CFLAR | AC112722.1 | 0.55514 | 1.41E-44 |
| BIRC6 | AC020915.3 | 0.555232 | 1.36E-44 |
| BIRC6 | AC025165.4 | 0.555233 | 1.36E-44 |
| NAF1 | AC073651.1 | 0.555242 | 1.35E-44 |
| ATG7 | AL133342.1 | 0.555243 | 1.35E-44 |
| FOXO1 | CR936218.1 | 0.555302 | 1.32E-44 |
| KLHL24 | MBNL1-AS1 | 0.555329 | 1.30E-44 |
| ATG12 | OIP5-AS1 | 0.555396 | 1.27E-44 |
| BIRC6 | AC008669.1 | 0.555416 | 1.26E-44 |
| NAF1 | ALG13-AS1 | 0.555452 | 1.24E-44 |
| ATG12 | AC078778.1 | 0.555482 | 1.22E-44 |
| RB1CC1 | SDCBP2-AS1 | 0.555491 | 1.22E-44 |
| NAF1 | AC090948.1 | 0.555565 | 1.18E-44 |
| RB1CC1 | ANKRD44-IT1 | 0.555573 | 1.17E-44 |
| MAPK8 | AP002336.2 | 0.555611 | 1.15E-44 |
| CFLAR | GARS-DT | 0.555653 | 1.13E-44 |
| RB1CC1 | AL049869.3 | 0.555738 | 1.09E-44 |
| RB1CC1 | AL049840.1 | 0.555756 | 1.08E-44 |
| MAPK8 | AC016394.1 | 0.555798 | 1.07E-44 |
| KLHL24 | AC108727.1 | 0.555804 | 1.06E-44 |
| EIF2AK2 | AC005070.3 | 0.555814 | 1.06E-44 |
| WDFY3 | RAB30-AS1 | 0.555827 | 1.05E-44 |
| MAPK8 | NDUFV2-AS1 | 0.555838 | 1.05E-44 |
| RB1CC1 | AC004223.3 | 0.55584 | 1.05E-44 |
| KLHL24 | AP000766.1 | 0.5559 | 1.02E-44 |
| RPS6KB1 | DLEU2 | 0.555902 | 1.02E-44 |
| EIF2AK2 | AC096741.1 | 0.555977 | 9.86E-45 |
| KLHL24 | AL022067.1 | 0.555991 | 9.80E-45 |
| MAPK8 | AC005838.2 | 0.555993 | 9.79E-45 |
| RPS6KB1 | Z68871.1 | 0.556017 | 9.69E-45 |
| PIK3C3 | MALAT1 | 0.556041 | 9.59E-45 |
| WDFY3 | LINC02035 | 0.556102 | 9.34E-45 |
| FOXO1 | LINC00216 | 0.556115 | 9.29E-45 |
| WDFY3 | AC008735.4 | 0.556129 | 9.24E-45 |
| ATG12 | AC011477.2 | 0.556141 | 9.19E-45 |
| PIK3C3 | AC131971.1 | 0.556163 | 9.10E-45 |
| KLHL24 | AC083843.2 | 0.556182 | 9.03E-45 |
| ATG2B | MUC20-OT1 | 0.556203 | 8.95E-45 |
| CFLAR | AC027277.2 | 0.55621 | 8.92E-45 |
| MAPK8 | LINC00513 | 0.55622 | 8.88E-45 |
| RB1CC1 | CR936218.1 | 0.556239 | 8.81E-45 |
| NAF1 | MALAT1 | 0.556298 | 8.59E-45 |
| RPS6KB1 | AC080162.1 | 0.556345 | 8.42E-45 |
| PIK3C3 | OIP5-AS1 | 0.556408 | 8.19E-45 |
| ATG2B | AC091185.1 | 0.556428 | 8.12E-45 |
| NAF1 | KLF7-IT1 | 0.55646 | 8.01E-45 |
| GOPC | AC022150.4 | 0.556478 | 7.95E-45 |
| ATG2B | AC005479.2 | 0.556622 | 7.47E-45 |
| KLHL24 | AL157402.2 | 0.556634 | 7.43E-45 |
| FOXO1 | GAS5-AS1 | 0.556658 | 7.35E-45 |
| TSC1 | AC024060.1 | 0.556675 | 7.30E-45 |
| EIF2AK2 | ALG13-AS1 | 0.556686 | 7.27E-45 |
| KLHL24 | AC007038.1 | 0.556722 | 7.15E-45 |
| TSC1 | AC009120.2 | 0.556723 | 7.15E-45 |
| EIF2AK2 | AL133330.1 | 0.556738 | 7.10E-45 |
| FOXO1 | ATP1B3-AS1 | 0.556766 | 7.02E-45 |
| PIK3C3 | AL731566.1 | 0.556779 | 6.98E-45 |
| RPS6KB1 | AC124319.2 | 0.556792 | 6.94E-45 |
| ATG12 | LINC00641 | 0.556812 | 6.88E-45 |
| CFLAR | AC098851.1 | 0.55683 | 6.83E-45 |
| ATG2B | AC069023.1 | 0.556852 | 6.76E-45 |
| MAPK8 | ANKRD44-IT1 | 0.556856 | 6.75E-45 |
| KLHL24 | FTX | 0.556887 | 6.66E-45 |
| PTEN | MCCC1-AS1 | 0.55689 | 6.65E-45 |
| NAF1 | SCARNA9 | 0.55692 | 6.56E-45 |
| BIRC6 | AC112722.1 | 0.556931 | 6.53E-45 |
| FOXO1 | AC073651.1 | 0.556954 | 6.47E-45 |
| MAPK8 | NPTN-IT1 | 0.556963 | 6.44E-45 |
| ATG12 | RAP2C-AS1 | 0.556974 | 6.41E-45 |
| NAF1 | RAP2C-AS1 | 0.557002 | 6.34E-45 |
| KLHL24 | AL031670.1 | 0.557044 | 6.22E-45 |
| GOPC | Z68871.1 | 0.55706 | 6.18E-45 |
| PIK3C3 | AC008906.1 | 0.557078 | 6.13E-45 |
| TSC1 | AC005253.1 | 0.557121 | 6.02E-45 |
| KLHL24 | AL049869.3 | 0.557128 | 6.00E-45 |
| EIF2AK2 | AC008770.3 | 0.557174 | 5.88E-45 |
| ATG12 | AC009054.2 | 0.557215 | 5.78E-45 |
| MAPK8 | BTBD9-AS1 | 0.557231 | 5.74E-45 |
| BIRC6 | AC048341.2 | 0.557351 | 5.45E-45 |
| PTEN | AC107027.3 | 0.557371 | 5.40E-45 |
| RB1CC1 | AL365277.1 | 0.557378 | 5.39E-45 |
| WDFY3 | AC087752.4 | 0.55746 | 5.20E-45 |
| CCR2 | AC145098.1 | 0.557468 | 5.18E-45 |
| RB1CC1 | AC087286.2 | 0.557475 | 5.16E-45 |
| MAPK8 | AP001178.2 | 0.557483 | 5.15E-45 |
| BIRC6 | AP006621.2 | 0.557486 | 5.14E-45 |
| KLHL24 | AC020915.2 | 0.557549 | 5.00E-45 |
| ATG2B | AC009090.1 | 0.557572 | 4.95E-45 |
| ATG2B | OIP5-AS1 | 0.557572 | 4.95E-45 |
| KLHL24 | AL138963.1 | 0.557591 | 4.91E-45 |
| NAF1 | AC124312.5 | 0.55762 | 4.85E-45 |
| RB1CC1 | AC022173.1 | 0.55769 | 4.71E-45 |
| KLHL24 | AP001033.2 | 0.557817 | 4.45E-45 |
| TSC1 | AC013403.2 | 0.557869 | 4.35E-45 |
| TSC1 | AC002553.1 | 0.557872 | 4.35E-45 |
| PIK3C3 | AL049869.3 | 0.557929 | 4.24E-45 |
| TSC1 | AC007566.1 | 0.557954 | 4.20E-45 |
| MAPK8 | AC004223.3 | 0.55797 | 4.17E-45 |
| KLHL24 | ALG13-AS1 | 0.557988 | 4.13E-45 |
| EIF2AK2 | FAM13A-AS1 | 0.558028 | 4.06E-45 |
| MAPK8 | AC090739.1 | 0.558029 | 4.06E-45 |
| WDFY3 | AC093110.1 | 0.558081 | 3.97E-45 |
| CFLAR | LINC00861 | 0.558127 | 3.89E-45 |
| ATG2B | AGAP1-IT1 | 0.558128 | 3.89E-45 |
| ATG2B | AL117379.1 | 0.558143 | 3.87E-45 |
| KLHL24 | AL049840.1 | 0.558169 | 3.82E-45 |
| ATG4B | AL139287.1 | 0.558198 | 3.78E-45 |
| TSC1 | AL133410.1 | 0.558253 | 3.69E-45 |
| RPS6KB1 | AC127024.5 | 0.558328 | 3.57E-45 |
| ATG2B | ACTA2-AS1 | 0.558333 | 3.56E-45 |
| PTEN | ATP13A4-AS1 | 0.558334 | 3.56E-45 |
| MAPK8 | AP005131.7 | 0.558364 | 3.51E-45 |
| FOXO1 | AL163051.2 | 0.558407 | 3.45E-45 |
| ATG12 | RFX3-AS1 | 0.558411 | 3.44E-45 |
| ATG2B | AC005046.1 | 0.558421 | 3.43E-45 |
| TSC1 | AC127024.5 | 0.558438 | 3.40E-45 |
| PTEN | RFX3-AS1 | 0.558456 | 3.38E-45 |
| BIRC6 | AL606489.1 | 0.558512 | 3.29E-45 |
| EIF2AK2 | AF117829.1 | 0.558512 | 3.29E-45 |
| ATG12 | AC026470.2 | 0.558519 | 3.28E-45 |
| RB1CC1 | AL590723.1 | 0.558561 | 3.22E-45 |
| RB1CC1 | RHOA-IT1 | 0.558565 | 3.22E-45 |
| NAF1 | AC011939.2 | 0.558642 | 3.11E-45 |
| FOXO1 | FTX | 0.558649 | 3.10E-45 |
| EIF2AK2 | AC108727.1 | 0.558733 | 2.99E-45 |
| RB1CC1 | AC008770.3 | 0.559066 | 2.59E-45 |
| EIF2AK2 | AC005034.5 | 0.559094 | 2.56E-45 |
| KLHL24 | AC080162.1 | 0.55913 | 2.52E-45 |
| FOXO1 | AC022973.3 | 0.559157 | 2.49E-45 |
| KLHL24 | AC022173.1 | 0.559188 | 2.45E-45 |
| KLHL24 | LINC01534 | 0.559192 | 2.45E-45 |
| MAPK8 | AC130650.2 | 0.559325 | 2.31E-45 |
| EIF2AK2 | AC068790.2 | 0.559368 | 2.27E-45 |
| PIK3C3 | AC010536.2 | 0.559444 | 2.20E-45 |
| EIF2AK2 | GAS5-AS1 | 0.559462 | 2.18E-45 |
| PIK3C3 | AP005131.7 | 0.55949 | 2.15E-45 |
| MAPK8 | AC110792.3 | 0.559506 | 2.14E-45 |
| ATG16L2 | PRKCZ-AS1 | 0.55957 | 2.08E-45 |
| CFLAR | SCARNA9 | 0.559576 | 2.07E-45 |
| PIK3C3 | AL136320.1 | 0.559586 | 2.06E-45 |
| EIF2AK2 | AC026124.2 | 0.559603 | 2.05E-45 |
| ATG2B | AC004656.1 | 0.559624 | 2.03E-45 |
| WDFY3 | AC016727.1 | 0.559724 | 1.94E-45 |
| FOXO1 | AP002336.2 | 0.559733 | 1.94E-45 |
| CFLAR | AC080162.1 | 0.559752 | 1.92E-45 |
| CFLAR | AC010761.3 | 0.559886 | 1.81E-45 |
| EIF2AK2 | AC015911.3 | 0.559888 | 1.81E-45 |
| PIK3C3 | AP001381.1 | 0.559895 | 1.80E-45 |
| CAPN10 | AC027796.4 | 0.559898 | 1.80E-45 |
| ATG2B | AL137782.1 | 0.559943 | 1.77E-45 |
| NAF1 | AC068790.3 | 0.559994 | 1.73E-45 |
| EIF2AK2 | AC022173.1 | 0.559996 | 1.73E-45 |
| PIK3C3 | AC108010.1 | 0.560044 | 1.69E-45 |
| CFLAR | AC114760.2 | 0.560062 | 1.68E-45 |
| BIRC6 | AC129510.1 | 0.56007 | 1.67E-45 |
| ATG2B | RAB30-AS1 | 0.560074 | 1.67E-45 |
| MAPK8 | AC127024.5 | 0.560099 | 1.65E-45 |
| CFLAR | AL031670.1 | 0.560171 | 1.60E-45 |
| PIK3C3 | AL353804.1 | 0.560176 | 1.60E-45 |
| ATG12 | PCBP1-AS1 | 0.560186 | 1.59E-45 |
| KLHL24 | AC090948.1 | 0.560187 | 1.59E-45 |
| MAPK8 | AL117381.1 | 0.560188 | 1.59E-45 |
| TSC1 | AC048382.2 | 0.560242 | 1.55E-45 |
| CFLAR | ALG13-AS1 | 0.560247 | 1.55E-45 |
| PTEN | TPT1-AS1 | 0.560267 | 1.53E-45 |
| KLHL24 | AC092801.1 | 0.560335 | 1.49E-45 |
| ATG2B | LINC01004 | 0.560338 | 1.49E-45 |
| NAF1 | AC098851.1 | 0.560346 | 1.48E-45 |
| KLHL24 | AC010761.3 | 0.560382 | 1.46E-45 |
| EIF2AK2 | ADAMTSL4-AS1 | 0.560443 | 1.42E-45 |
| FOXO1 | AC037487.2 | 0.560474 | 1.40E-45 |
| KLHL24 | AL049840.5 | 0.560474 | 1.40E-45 |
| CFLAR | AC007546.1 | 0.560482 | 1.40E-45 |
| EIF2AK2 | AC004832.5 | 0.560531 | 1.37E-45 |
| EIF2AK2 | ACAP2-IT1 | 0.560577 | 1.34E-45 |
| PIK3C3 | ITCH-IT1 | 0.560604 | 1.32E-45 |
| MAPK8 | AC090181.2 | 0.560687 | 1.28E-45 |
| BIRC6 | AC009090.1 | 0.560707 | 1.26E-45 |
| MAPK8 | LIMS1-AS1 | 0.560714 | 1.26E-45 |
| RB1CC1 | AL139407.1 | 0.56074 | 1.25E-45 |
| KLHL24 | ARHGEF38-IT1 | 0.560763 | 1.23E-45 |
| EIF2AK2 | AC020915.2 | 0.560784 | 1.22E-45 |
| FOXO1 | AL353804.1 | 0.560845 | 1.19E-45 |
| EIF2AK2 | AF129075.1 | 0.56093 | 1.15E-45 |
| RB1CC1 | AL513365.2 | 0.56094 | 1.14E-45 |
| PIK3C3 | AL133445.2 | 0.560986 | 1.12E-45 |
| RPS6KB1 | AC079684.1 | 0.561006 | 1.11E-45 |
| KLHL24 | ADAMTSL4-AS1 | 0.56101 | 1.11E-45 |
| PIK3C3 | AC005920.2 | 0.561032 | 1.10E-45 |
| PIK3C3 | AC087284.1 | 0.561048 | 1.09E-45 |
| ATG2B | AC025171.4 | 0.5611 | 1.07E-45 |
| MAPK8 | AC127024.4 | 0.561136 | 1.05E-45 |
| ATG12 | AL354696.1 | 0.56114 | 1.05E-45 |
| CFLAR | AC083843.2 | 0.561143 | 1.05E-45 |
| KLHL24 | Z68871.1 | 0.56115 | 1.04E-45 |
| ATG4B | AL928654.2 | 0.561171 | 1.03E-45 |
| EIF2AK2 | AL136320.1 | 0.561243 | 1.00E-45 |
| TSC1 | LINC01355 | 0.561253 | 9.96E-46 |
| KLHL24 | AL133445.2 | 0.561281 | 9.83E-46 |
| RB1CC1 | AC090739.1 | 0.561332 | 9.62E-46 |
| ATG16L2 | AC005253.1 | 0.561333 | 9.62E-46 |
| MAPK8 | AC087284.1 | 0.561365 | 9.48E-46 |
| CFLAR | AC004492.1 | 0.561372 | 9.45E-46 |
| ATG12 | AC037198.1 | 0.561391 | 9.37E-46 |
| ATG4B | AC027796.4 | 0.561417 | 9.27E-46 |
| FOXO1 | AP000766.1 | 0.561441 | 9.17E-46 |
| KLHL24 | AC004492.1 | 0.561477 | 9.03E-46 |
| PIK3C3 | CFLAR-AS1 | 0.56153 | 8.82E-46 |
| FOXO1 | BTBD9-AS1 | 0.561533 | 8.81E-46 |
| ATG12 | AC008035.1 | 0.561562 | 8.70E-46 |
| KLHL24 | MAL2-AS1 | 0.56157 | 8.66E-46 |
| MAPK8 | AC138932.5 | 0.561592 | 8.58E-46 |
| TSC1 | Z98884.2 | 0.561607 | 8.53E-46 |
| PIK3C3 | AC068790.3 | 0.56163 | 8.44E-46 |
| PIK3C3 | AC007216.4 | 0.561632 | 8.43E-46 |
| EIF2AK2 | AL136115.2 | 0.561694 | 8.21E-46 |
| ATG12 | AC004918.3 | 0.561735 | 8.06E-46 |
| TSC2 | AL031709.1 | 0.561745 | 8.03E-46 |
| ATG16L2 | AL021707.3 | 0.561767 | 7.95E-46 |
| EIF2AK2 | AL049840.5 | 0.561779 | 7.91E-46 |
| NAF1 | AC090739.1 | 0.561821 | 7.76E-46 |
| BIRC6 | ARHGAP31-AS1 | 0.561829 | 7.73E-46 |
| KLHL24 | AC013403.2 | 0.561833 | 7.72E-46 |
| EIF2AK2 | AC108449.2 | 0.561848 | 7.67E-46 |
| PTEN | AC084117.1 | 0.561852 | 7.66E-46 |
| EIF2AK2 | FTX | 0.56189 | 7.53E-46 |
| PTEN | AL606834.1 | 0.561926 | 7.41E-46 |
| KLHL24 | TRAF3IP2-AS1 | 0.56199 | 7.21E-46 |
| PIK3C3 | AL163051.2 | 0.562004 | 7.16E-46 |
| MAPK8 | AL355488.1 | 0.562057 | 7.00E-46 |
| CFLAR | MIR29B2CHG | 0.562134 | 6.76E-46 |
| MAPK8 | AL049869.3 | 0.56215 | 6.71E-46 |
| TP73 | ELN-AS1 | 0.562173 | 6.65E-46 |
| ATG12 | AC018682.1 | 0.56218 | 6.63E-46 |
| RB1CC1 | AC004832.5 | 0.562194 | 6.59E-46 |
| ATG12 | ATP1B3-AS1 | 0.562244 | 6.44E-46 |
| CFLAR | AL049840.1 | 0.562258 | 6.40E-46 |
| WDFY3 | TBILA | 0.56226 | 6.40E-46 |
| RB1CC1 | AC090948.2 | 0.562268 | 6.38E-46 |
| EIF2AK2 | AC016831.4 | 0.56233 | 6.20E-46 |
| EIF2AK2 | AC242426.2 | 0.562369 | 6.10E-46 |
| TSC1 | AL139287.1 | 0.562403 | 6.01E-46 |
| ATG12 | RBMS3-AS3 | 0.562465 | 5.85E-46 |
| FOXO1 | AC130895.1 | 0.562474 | 5.82E-46 |
| MAPK8 | AC090948.2 | 0.562491 | 5.78E-46 |
| EIF2AK2 | AP000766.1 | 0.562493 | 5.77E-46 |
| FOXO1 | AC026124.2 | 0.562546 | 5.64E-46 |
| BIRC6 | AC084117.1 | 0.562553 | 5.62E-46 |
| PTEN | GK-AS1 | 0.562561 | 5.60E-46 |
| FOXO1 | AC007216.4 | 0.562565 | 5.59E-46 |
| NAF1 | AC114760.2 | 0.562582 | 5.55E-46 |
| ATG12 | AC067852.3 | 0.562614 | 5.47E-46 |
| NAF1 | AL136320.1 | 0.562774 | 5.10E-46 |
| RB1CC1 | AC011939.2 | 0.562807 | 5.03E-46 |
| BIRC6 | AC093495.1 | 0.562816 | 5.01E-46 |
| ATG12 | AL662844.3 | 0.562819 | 5.00E-46 |
| CFLAR | AL513008.1 | 0.562828 | 4.98E-46 |
| MAPK8 | DLEU2 | 0.562858 | 4.92E-46 |
| PIK3C3 | RHOA-IT1 | 0.562864 | 4.90E-46 |
| KLHL24 | DLEU2 | 0.562925 | 4.77E-46 |
| BIRC6 | AL158212.3 | 0.562948 | 4.73E-46 |
| KLHL24 | LINC-PINT | 0.562986 | 4.65E-46 |
| KLHL24 | AC010834.3 | 0.563021 | 4.58E-46 |
| ATG4B | AC139530.1 | 0.563022 | 4.57E-46 |
| CFLAR | SH3BP5-AS1 | 0.563041 | 4.54E-46 |
| BIRC6 | AC016727.1 | 0.563065 | 4.49E-46 |
| RB1CC1 | AF129075.1 | 0.563076 | 4.47E-46 |
| RB1CC1 | AC114760.2 | 0.563112 | 4.40E-46 |
| RB1CC1 | ITCH-IT1 | 0.563144 | 4.33E-46 |
| PIK3C3 | ACAP2-IT1 | 0.56317 | 4.28E-46 |
| RB1CC1 | AC016831.4 | 0.563172 | 4.28E-46 |
| WDFY3 | ARMCX5-GPRASP2 | 0.563174 | 4.28E-46 |
| FOXO1 | AC048344.4 | 0.563209 | 4.21E-46 |
| EIF2AK2 | AC004223.3 | 0.563211 | 4.21E-46 |
| WDFY3 | AC129510.1 | 0.563214 | 4.20E-46 |
| FOXO1 | AC092801.1 | 0.56324 | 4.15E-46 |
| TSC2 | ZNF213-AS1 | 0.563268 | 4.10E-46 |
| MAPK8 | Z82243.1 | 0.563299 | 4.05E-46 |
| CAPN10 | AC114730.3 | 0.563309 | 4.03E-46 |
| PIK3C3 | AC068790.2 | 0.563315 | 4.02E-46 |
| PIK3C3 | AC138393.3 | 0.563375 | 3.91E-46 |
| EIF2AK2 | AL139407.1 | 0.563458 | 3.77E-46 |
| NAF1 | AC068790.2 | 0.563478 | 3.74E-46 |
| NAF1 | AC018926.3 | 0.563634 | 3.49E-46 |
| BIRC6 | AC087481.3 | 0.563635 | 3.49E-46 |
| ATG2B | AL117336.2 | 0.563745 | 3.32E-46 |
| MAPK8 | AP001381.1 | 0.563771 | 3.29E-46 |
| PIK3C3 | N4BP2L2-IT2 | 0.563805 | 3.24E-46 |
| ATG12 | AP000692.1 | 0.563859 | 3.16E-46 |
| WDFY3 | AL078581.1 | 0.563873 | 3.14E-46 |
| ATG16L2 | AC107464.3 | 0.563877 | 3.14E-46 |
| WDFY3 | AL159169.2 | 0.563922 | 3.07E-46 |
| ATG12 | AC004884.2 | 0.563934 | 3.06E-46 |
| PTEN | AL132780.1 | 0.563972 | 3.01E-46 |
| MAPK8 | AC114760.2 | 0.564038 | 2.92E-46 |
| PIK3C3 | AL162724.1 | 0.564062 | 2.89E-46 |
| FOXO1 | AC124283.3 | 0.564105 | 2.83E-46 |
| CFLAR | AL157392.3 | 0.564111 | 2.83E-46 |
| MAPK8 | AL365277.1 | 0.564144 | 2.79E-46 |
| RB1CC1 | AC006059.1 | 0.564165 | 2.76E-46 |
| WDFY3 | AL445493.3 | 0.564207 | 2.71E-46 |
| PIK3C3 | AL136115.2 | 0.564209 | 2.71E-46 |
| KLHL24 | AC011477.2 | 0.564224 | 2.69E-46 |
| ATG2B | AC120053.1 | 0.564241 | 2.67E-46 |
| ATG2B | AP000240.1 | 0.564263 | 2.64E-46 |
| PIK3C3 | AL133243.2 | 0.564311 | 2.59E-46 |
| IFNG | USP30-AS1 | 0.564344 | 2.55E-46 |
| BIRC6 | EGOT | 0.564381 | 2.51E-46 |
| KLHL24 | AC130895.1 | 0.564395 | 2.49E-46 |
| EIF2AK2 | AC090181.2 | 0.564408 | 2.48E-46 |
| BIRC5 | TMPO-AS1 | 0.56442 | 2.47E-46 |
| KLHL24 | AF129075.1 | 0.564437 | 2.45E-46 |
| PTEN | PSMA3-AS1 | 0.564497 | 2.38E-46 |
| RB1CC1 | AC022150.4 | 0.564507 | 2.37E-46 |
| RPS6KB1 | AL354989.1 | 0.564562 | 2.31E-46 |
| RPS6KB1 | AC004492.1 | 0.564575 | 2.30E-46 |
| PIK3C3 | AC011939.2 | 0.564581 | 2.30E-46 |
| FOXO1 | AC002553.2 | 0.564714 | 2.16E-46 |
| PIK3C3 | AC010761.3 | 0.564766 | 2.11E-46 |
| KLHL24 | AL513365.2 | 0.564806 | 2.08E-46 |
| PTEN | LINC01534 | 0.564813 | 2.07E-46 |
| FOXO1 | AC006059.1 | 0.564861 | 2.03E-46 |
| CFLAR | PAXBP1-AS1 | 0.56489 | 2.00E-46 |
| KLHL24 | AC090948.2 | 0.564932 | 1.96E-46 |
| BIRC6 | AL078581.1 | 0.565011 | 1.90E-46 |
| MAPK8 | AC018752.1 | 0.565012 | 1.90E-46 |
| MAPK8 | MAL2-AS1 | 0.565021 | 1.89E-46 |
| FOXO1 | AL133243.2 | 0.565031 | 1.88E-46 |
| RB1CC1 | AP001432.1 | 0.565113 | 1.81E-46 |
| CFLAR | AL021707.7 | 0.565118 | 1.81E-46 |
| IFNG | LINC01943 | 0.56512 | 1.81E-46 |
| PTEN | AC091185.1 | 0.565127 | 1.80E-46 |
| BIRC6 | AC135050.5 | 0.565153 | 1.78E-46 |
| FOXO1 | AC011472.4 | 0.565173 | 1.77E-46 |
| ATG2B | AC090517.2 | 0.565176 | 1.76E-46 |
| MAPK8 | AC131971.1 | 0.565181 | 1.76E-46 |
| ATG12 | AC234772.2 | 0.565304 | 1.66E-46 |
| PIK3C3 | AC087286.2 | 0.565426 | 1.58E-46 |
| ATG12 | AC027277.2 | 0.565429 | 1.57E-46 |
| ATG16L2 | AP002807.1 | 0.565439 | 1.57E-46 |
| PIK3C3 | AC025917.1 | 0.565485 | 1.54E-46 |
| PIK3C3 | AC004492.1 | 0.565505 | 1.52E-46 |
| RB1CC1 | AC090198.1 | 0.565509 | 1.52E-46 |
| PIK3C3 | AC022173.1 | 0.565511 | 1.52E-46 |
| RB1CC1 | AC012557.1 | 0.565527 | 1.51E-46 |
| BIRC6 | AC008969.1 | 0.565549 | 1.49E-46 |
| WDFY3 | AC020915.3 | 0.565625 | 1.44E-46 |
| MAPK8 | AP001429.1 | 0.565646 | 1.43E-46 |
| NAF1 | CR936218.1 | 0.565722 | 1.38E-46 |
| PIK3C3 | AC124312.2 | 0.565777 | 1.35E-46 |
| FOXO1 | RRN3P2 | 0.565786 | 1.34E-46 |
| ATG2B | AC026355.1 | 0.565861 | 1.30E-46 |
| PIK3C3 | AL138963.1 | 0.565878 | 1.29E-46 |
| EIF2AK2 | AL133445.2 | 0.565883 | 1.29E-46 |
| KLHL24 | AC114760.2 | 0.565914 | 1.27E-46 |
| WDFY3 | AC079907.1 | 0.565918 | 1.27E-46 |
| RB1CC1 | AL133445.2 | 0.565973 | 1.24E-46 |
| FOXO1 | CFLAR-AS1 | 0.565978 | 1.23E-46 |
| ATG12 | AC010201.2 | 0.565981 | 1.23E-46 |
| TSC1 | AP001625.2 | 0.565995 | 1.22E-46 |
| PIK3C3 | Z83843.1 | 0.566003 | 1.22E-46 |
| KLHL24 | ADNP-AS1 | 0.566007 | 1.22E-46 |
| ATG4B | AL021707.6 | 0.566042 | 1.20E-46 |
| MAPK8 | AC073651.1 | 0.566057 | 1.19E-46 |
| NAF1 | AL138963.1 | 0.566062 | 1.19E-46 |
| RB1CC1 | AC058791.1 | 0.566084 | 1.18E-46 |
| PTEN | AC015871.3 | 0.56621 | 1.11E-46 |
| FOXO1 | AL139407.1 | 0.566237 | 1.10E-46 |
| WDFY3 | NORAD | 0.566247 | 1.09E-46 |
| NAF1 | SOS1-IT1 | 0.566258 | 1.09E-46 |
| BIRC6 | AL109761.1 | 0.566272 | 1.08E-46 |
| WDFY3 | AC025178.1 | 0.566286 | 1.08E-46 |
| ATG16L2 | AC004687.1 | 0.566307 | 1.07E-46 |
| ATG12 | ANKRD10-IT1 | 0.566327 | 1.06E-46 |
| PIK3C3 | SMC5-AS1 | 0.566349 | 1.05E-46 |
| RB1CC1 | AC138932.5 | 0.566359 | 1.04E-46 |
| ATG16L2 | AC110285.2 | 0.566366 | 1.04E-46 |
| EIF2AK2 | HCG18 | 0.566371 | 1.04E-46 |
| MAPK8 | AC007216.4 | 0.566577 | 9.44E-47 |
| TSC1 | AC008764.2 | 0.566677 | 9.03E-47 |
| KLHL24 | AL136115.2 | 0.566685 | 9.00E-47 |
| PIK3C3 | GAS5-AS1 | 0.56669 | 8.98E-47 |
| NAF1 | AL049552.1 | 0.566696 | 8.96E-47 |
| TSC1 | AL136295.7 | 0.566755 | 8.72E-47 |
| WDFY3 | AL117379.1 | 0.566762 | 8.69E-47 |
| NAF1 | AC063965.1 | 0.566767 | 8.67E-47 |
| NAF1 | AC008770.3 | 0.566813 | 8.50E-47 |
| EIF2AK2 | AC131971.1 | 0.566832 | 8.43E-47 |
| TSC1 | CTBP1-AS | 0.56692 | 8.10E-47 |
| RB1CC1 | AC253536.3 | 0.566987 | 7.86E-47 |
| NAF1 | AC080162.1 | 0.566993 | 7.84E-47 |
| NAF1 | AC234775.3 | 0.567098 | 7.48E-47 |
| ATG4B | AC087741.1 | 0.567102 | 7.47E-47 |
| WDFY3 | AL132657.1 | 0.567219 | 7.09E-47 |
| MAPK8 | AP000766.1 | 0.567225 | 7.07E-47 |
| PIK3C3 | SDCBP2-AS1 | 0.567238 | 7.03E-47 |
| TSC1 | CCDC18-AS1 | 0.567292 | 6.86E-47 |
| BIRC6 | AC092123.1 | 0.567344 | 6.70E-47 |
| EIF2AK2 | CR936218.1 | 0.567397 | 6.54E-47 |
| CFLAR | AC008770.3 | 0.567403 | 6.53E-47 |
| KLHL24 | AL353804.1 | 0.56743 | 6.45E-47 |
| ATG4B | AL031186.1 | 0.56743 | 6.45E-47 |
| RB1CC1 | AL138963.1 | 0.567447 | 6.40E-47 |
| EIF2AK2 | AC114760.2 | 0.567505 | 6.23E-47 |
| PIK3C3 | LINC00630 | 0.567534 | 6.16E-47 |
| KLHL24 | AC011939.2 | 0.567634 | 5.89E-47 |
| CFLAR | AC138932.5 | 0.567635 | 5.88E-47 |
| CFLAR | AC025171.4 | 0.567648 | 5.85E-47 |
| PIK3C3 | AC114760.2 | 0.5677 | 5.71E-47 |
| ATG12 | AL359697.1 | 0.567838 | 5.37E-47 |
| WDFY3 | LANCL1-AS1 | 0.567941 | 5.13E-47 |
| WDFY3 | AC145423.3 | 0.567994 | 5.01E-47 |
| BIRC6 | AC078883.1 | 0.568013 | 4.96E-47 |
| PIK3C3 | AC124283.3 | 0.568024 | 4.94E-47 |
| NAF1 | AL590723.1 | 0.568089 | 4.80E-47 |
| MAPK8 | AC068790.5 | 0.568094 | 4.79E-47 |
| EIF2AK2 | AL139120.1 | 0.568112 | 4.75E-47 |
| CFLAR | AL049552.1 | 0.568158 | 4.65E-47 |
| MAPK8 | AC025917.1 | 0.568159 | 4.65E-47 |
| RB1CC1 | AC068790.2 | 0.568162 | 4.64E-47 |
| PIK3C3 | SOS1-IT1 | 0.568206 | 4.55E-47 |
| EIF2AK2 | AC093388.1 | 0.568229 | 4.51E-47 |
| KLHL24 | AC026124.2 | 0.568248 | 4.47E-47 |
| NAF1 | AC022173.1 | 0.568294 | 4.38E-47 |
| EIF2AK2 | AP001033.2 | 0.568298 | 4.37E-47 |
| TSC1 | AGBL5-IT1 | 0.568315 | 4.34E-47 |
| EIF2AK2 | AC090948.1 | 0.568323 | 4.32E-47 |
| CFLAR | AC015911.3 | 0.568347 | 4.27E-47 |
| MAPK8 | AC080162.1 | 0.568412 | 4.15E-47 |
| TSC1 | AC015849.3 | 0.568563 | 3.88E-47 |
| EIF2AK2 | AC087276.1 | 0.568589 | 3.83E-47 |
| CFLAR | CD44-AS1 | 0.56871 | 3.63E-47 |
| CFLAR | AC138956.2 | 0.568881 | 3.36E-47 |
| ATG2B | GABPB1-AS1 | 0.568913 | 3.31E-47 |
| ATG2B | STARD4-AS1 | 0.568921 | 3.30E-47 |
| EIF2AK2 | AC010761.3 | 0.569 | 3.19E-47 |
| MAPK8 | AC004492.1 | 0.569036 | 3.14E-47 |
| PTEN | AC011468.5 | 0.569053 | 3.11E-47 |
| NAF1 | AL133243.2 | 0.569056 | 3.11E-47 |
| KLHL24 | AC025917.1 | 0.569104 | 3.04E-47 |
| ATG2B | UGDH-AS1 | 0.569143 | 2.99E-47 |
| KLHL24 | ACAP2-IT1 | 0.569158 | 2.97E-47 |
| CFLAR | AL021707.8 | 0.569216 | 2.89E-47 |
| PIK3C3 | AL139407.1 | 0.569245 | 2.85E-47 |
| ATG2B | AC011442.1 | 0.569253 | 2.84E-47 |
| KLHL24 | AC008115.3 | 0.569289 | 2.80E-47 |
| FOXO1 | AC010186.3 | 0.569355 | 2.72E-47 |
| FOXO1 | NUTM2B-AS1 | 0.569416 | 2.64E-47 |
| MAPK8 | AC068790.2 | 0.569424 | 2.63E-47 |
| ATG2B | AC026356.1 | 0.569432 | 2.62E-47 |
| MAPK8 | AC090517.2 | 0.569491 | 2.55E-47 |
| EIF2AK2 | AC138932.5 | 0.569507 | 2.54E-47 |
| FOXO1 | AL049840.1 | 0.569538 | 2.50E-47 |
| WDFY3 | GK-AS1 | 0.569593 | 2.44E-47 |
| RB1CC1 | AC234775.3 | 0.569651 | 2.38E-47 |
| PIK3C3 | NPTN-IT1 | 0.569679 | 2.35E-47 |
| NAF1 | AC124283.3 | 0.569804 | 2.22E-47 |
| TSC1 | AC018690.1 | 0.56983 | 2.19E-47 |
| CFLAR | AC004466.3 | 0.569835 | 2.19E-47 |
| FOXO1 | AL513365.2 | 0.569876 | 2.15E-47 |
| PTEN | AC087222.1 | 0.569878 | 2.15E-47 |
| ATG2B | AL031666.1 | 0.5699 | 2.12E-47 |
| KLHL24 | AP005131.7 | 0.569903 | 2.12E-47 |
| MAPK8 | Z83843.1 | 0.569905 | 2.12E-47 |
| ATG2B | AC008735.4 | 0.569925 | 2.10E-47 |
| RB1CC1 | AC092801.1 | 0.569966 | 2.06E-47 |
| RB1CC1 | AL139120.1 | 0.569984 | 2.04E-47 |
| NAF1 | CFLAR-AS1 | 0.57 | 2.03E-47 |
| NFE2L2 | UNC5B-AS1 | 0.570001 | 2.03E-47 |
| RB1CC1 | AL049840.5 | 0.570009 | 2.02E-47 |
| PIK3C3 | AC022150.4 | 0.570019 | 2.01E-47 |
| CCR2 | AC090559.1 | 0.570069 | 1.97E-47 |
| PIK3C3 | SCARNA9 | 0.570077 | 1.96E-47 |
| MAPK8 | AC063965.1 | 0.570083 | 1.95E-47 |
| ATG2B | AL157394.1 | 0.570104 | 1.94E-47 |
| CFLAR | PPP3CB-AS1 | 0.570132 | 1.91E-47 |
| TSC1 | AL450384.2 | 0.570231 | 1.83E-47 |
| NAF1 | AC004492.1 | 0.570244 | 1.82E-47 |
| TSC2 | AC108134.1 | 0.570273 | 1.79E-47 |
| KLHL24 | AL109614.1 | 0.570277 | 1.79E-47 |
| ATG12 | AC048344.4 | 0.570277 | 1.79E-47 |
| PIK3C3 | AP001178.2 | 0.570326 | 1.75E-47 |
| TSC1 | UBE2Q1-AS1 | 0.570338 | 1.74E-47 |
| ATG2B | AC009090.3 | 0.570349 | 1.73E-47 |
| ATG2B | AC006378.1 | 0.570356 | 1.73E-47 |
| FOXO1 | AC004492.1 | 0.570406 | 1.69E-47 |
| ATG12 | MIR222HG | 0.570447 | 1.66E-47 |
| LAMP1 | AL442125.2 | 0.570468 | 1.64E-47 |
| MAPK8 | FTX | 0.570473 | 1.64E-47 |
| MAPK8 | AC087286.2 | 0.570513 | 1.61E-47 |
| CFLAR | AL354733.3 | 0.570524 | 1.60E-47 |
| KLHL24 | AC005070.3 | 0.570555 | 1.58E-47 |
| BIRC6 | AC098484.1 | 0.570586 | 1.56E-47 |
| ATG12 | AL731566.1 | 0.570715 | 1.47E-47 |
| PTEN | AC138956.2 | 0.570731 | 1.46E-47 |
| PIK3C3 | AC063965.1 | 0.570738 | 1.45E-47 |
| MAPK8 | AC068790.3 | 0.570745 | 1.45E-47 |
| EIF2AK2 | GMDS-DT | 0.570769 | 1.43E-47 |
| ATG12 | ATP1A1-AS1 | 0.570844 | 1.39E-47 |
| PIK3C3 | AC007878.1 | 0.570849 | 1.38E-47 |
| RB1CC1 | AL136115.2 | 0.570851 | 1.38E-47 |
| TSC1 | AC093495.1 | 0.570869 | 1.37E-47 |
| ATG16L2 | PDXDC2P-NPIPB14P | 0.570947 | 1.32E-47 |
| FOXO1 | AC090948.2 | 0.571025 | 1.28E-47 |
| EIF2AK2 | LINC00216 | 0.571056 | 1.26E-47 |
| BIRC6 | AC005674.2 | 0.571058 | 1.26E-47 |
| MAPK8 | AC130895.1 | 0.571074 | 1.25E-47 |
| RB1CC1 | AC124283.3 | 0.571082 | 1.24E-47 |
| RB1CC1 | SCARNA9 | 0.571111 | 1.23E-47 |
| ATG12 | AC124319.2 | 0.571235 | 1.16E-47 |
| ATG12 | AC133644.2 | 0.571306 | 1.12E-47 |
| TP73 | SRGAP3-AS2 | 0.571313 | 1.12E-47 |
| WDFY3 | AC073896.3 | 0.57137 | 1.09E-47 |
| CFLAR | AL137003.2 | 0.57138 | 1.09E-47 |
| WDFY3 | AL121839.2 | 0.571405 | 1.07E-47 |
| RB1CC1 | GAS5-AS1 | 0.571456 | 1.05E-47 |
| NAF1 | AC087286.2 | 0.571488 | 1.03E-47 |
| RB1CC1 | ACAP2-IT1 | 0.571508 | 1.03E-47 |
| PTEN | LINC01376 | 0.57155 | 1.01E-47 |
| EIF2AK2 | ARHGEF38-IT1 | 0.571554 | 1.00E-47 |
| TSC1 | MUC20-OT1 | 0.571558 | 1.00E-47 |
| KLHL24 | AC087276.1 | 0.571583 | 9.91E-48 |
| EIF2AK2 | AP001381.1 | 0.571645 | 9.63E-48 |
| PIK3C3 | AC007038.1 | 0.571804 | 8.96E-48 |
| EIF2AK2 | AL138963.1 | 0.571813 | 8.92E-48 |
| CCR2 | LINC01094 | 0.571821 | 8.89E-48 |
| PIK3C3 | CR936218.1 | 0.571823 | 8.89E-48 |
| MAPK8 | AC087286.4 | 0.571857 | 8.75E-48 |
| MAPK8 | AC012170.2 | 0.571858 | 8.74E-48 |
| NAF1 | AC004832.5 | 0.571865 | 8.72E-48 |
| RPS6KB1 | LINC00630 | 0.571901 | 8.57E-48 |
| TP73 | AC013264.1 | 0.571903 | 8.57E-48 |
| BIRC6 | AL356356.1 | 0.571903 | 8.57E-48 |
| RB1CC1 | AC002553.2 | 0.571953 | 8.37E-48 |
| WDFY3 | AC084824.5 | 0.572011 | 8.16E-48 |
| EIF2AK2 | AC097376.2 | 0.572016 | 8.14E-48 |
| RB1CC1 | AC131971.1 | 0.57205 | 8.01E-48 |
| EIF4EBP1 | AC087623.1 | 0.572076 | 7.92E-48 |
| PIK3C3 | AC080162.1 | 0.572084 | 7.89E-48 |
| PTEN | AC011468.1 | 0.572086 | 7.88E-48 |
| PIK3C3 | AC090948.1 | 0.572209 | 7.45E-48 |
| ATG12 | AC124312.2 | 0.572231 | 7.38E-48 |
| PIK3C3 | AC007216.3 | 0.572233 | 7.37E-48 |
| MAPK8 | ITCH-IT1 | 0.57225 | 7.32E-48 |
| CFLAR | AC004253.1 | 0.57235 | 6.99E-48 |
| SPNS1 | AL031600.1 | 0.572357 | 6.97E-48 |
| MAPK8 | AP002907.1 | 0.5724 | 6.83E-48 |
| ATG12 | AC010834.3 | 0.572418 | 6.78E-48 |
| ATG12 | AC025171.3 | 0.572429 | 6.74E-48 |
| ATG16L2 | AL136295.7 | 0.572436 | 6.72E-48 |
| ATG2B | AC084871.1 | 0.572467 | 6.63E-48 |
| NAF1 | AL109614.1 | 0.572484 | 6.58E-48 |
| MAPK8 | AL513365.2 | 0.572485 | 6.57E-48 |
| WDFY3 | AC025171.2 | 0.572518 | 6.47E-48 |
| ATG12 | AL360219.1 | 0.572529 | 6.44E-48 |
| NAF1 | ACAP2-IT1 | 0.572621 | 6.18E-48 |
| EIF2AK2 | AC009318.3 | 0.572707 | 5.94E-48 |
| EIF2AK2 | AC080162.1 | 0.572732 | 5.87E-48 |
| EIF2AK2 | ANKRD44-IT1 | 0.572764 | 5.79E-48 |
| RB1CC1 | AP001429.1 | 0.572818 | 5.65E-48 |
| EIF2AK2 | AC025917.1 | 0.572833 | 5.61E-48 |
| NAF1 | AC090181.2 | 0.572855 | 5.55E-48 |
| FOXO1 | KLF7-IT1 | 0.572863 | 5.53E-48 |
| NAF1 | LINC00216 | 0.572864 | 5.53E-48 |
| PIK3C3 | TRAF3IP2-AS1 | 0.573015 | 5.16E-48 |
| ATG12 | AC027097.2 | 0.57302 | 5.15E-48 |
| WDFY3 | AL121772.3 | 0.573025 | 5.14E-48 |
| MAPK8 | AC010834.3 | 0.573062 | 5.05E-48 |
| ATG2B | AL360219.1 | 0.573072 | 5.03E-48 |
| CFLAR | KLF7-IT1 | 0.57311 | 4.94E-48 |
| TSC1 | AC008982.2 | 0.573111 | 4.94E-48 |
| ATG12 | AL513327.1 | 0.573118 | 4.92E-48 |
| ATG12 | AC018638.7 | 0.573158 | 4.84E-48 |
| MAPK8 | AC007038.2 | 0.573212 | 4.72E-48 |
| EIF2AK2 | AL513365.2 | 0.57324 | 4.66E-48 |
| EIF2AK2 | AC124319.2 | 0.573244 | 4.65E-48 |
| MAPK8 | AC022173.1 | 0.573257 | 4.62E-48 |
| FOXO1 | AL590723.1 | 0.573264 | 4.61E-48 |
| PIK3C3 | AP000766.1 | 0.573269 | 4.60E-48 |
| ATG2B | AL683813.1 | 0.573281 | 4.57E-48 |
| WDFY3 | AC112496.1 | 0.573322 | 4.49E-48 |
| PIK3C3 | AL109614.1 | 0.573396 | 4.34E-48 |
| FOXO1 | AP001381.1 | 0.573458 | 4.22E-48 |
| ATG2B | AC009318.3 | 0.573569 | 4.01E-48 |
| TSC1 | AC138956.2 | 0.573654 | 3.86E-48 |
| NAF1 | AC016590.2 | 0.573737 | 3.71E-48 |
| NAF1 | AC087276.1 | 0.573756 | 3.68E-48 |
| NAF1 | AL139407.1 | 0.573802 | 3.60E-48 |
| MAPK8 | AC004832.5 | 0.573846 | 3.53E-48 |
| KLHL24 | AL359076.1 | 0.573975 | 3.33E-48 |
| MAPK8 | AC007038.1 | 0.573976 | 3.33E-48 |
| FOXO1 | AC090739.1 | 0.57398 | 3.32E-48 |
| RB1CC1 | AC007216.3 | 0.574003 | 3.29E-48 |
| FOXO1 | LINC00513 | 0.574029 | 3.25E-48 |
| FOXO1 | AC008115.3 | 0.574056 | 3.21E-48 |
| NAF1 | AC005070.3 | 0.574079 | 3.17E-48 |
| KLHL24 | LINC00216 | 0.574136 | 3.09E-48 |
| FOXO1 | SOS1-IT1 | 0.574279 | 2.90E-48 |
| EIF2AK2 | AC008115.3 | 0.57432 | 2.84E-48 |
| ATG2B | AP003392.1 | 0.574368 | 2.78E-48 |
| PIK3C3 | AC053513.1 | 0.574375 | 2.77E-48 |
| RB1CC1 | AC048344.4 | 0.574402 | 2.74E-48 |
| KLHL24 | AC131971.1 | 0.574412 | 2.72E-48 |
| ATG12 | AC139887.4 | 0.574451 | 2.68E-48 |
| KLHL24 | CR936218.1 | 0.574531 | 2.58E-48 |
| EIF2AK2 | CFLAR-AS1 | 0.574539 | 2.57E-48 |
| ATG2B | AC087752.4 | 0.574553 | 2.55E-48 |
| ATG7 | MIR155HG | 0.574572 | 2.53E-48 |
| ATG2B | AC092279.1 | 0.574595 | 2.51E-48 |
| NAF1 | AL139120.1 | 0.574631 | 2.46E-48 |
| BIRC6 | AC068152.1 | 0.574632 | 2.46E-48 |
| RB1CC1 | AC130895.1 | 0.574635 | 2.46E-48 |
| FOXO1 | AL139120.1 | 0.574637 | 2.46E-48 |
| ATG12 | LINC00861 | 0.574655 | 2.44E-48 |
| PIK3C3 | AC016590.2 | 0.574671 | 2.42E-48 |
| FOXO1 | AC022173.1 | 0.574674 | 2.42E-48 |
| RB1CC1 | AC097376.2 | 0.574696 | 2.39E-48 |
| PIK3C3 | AC234775.3 | 0.574776 | 2.30E-48 |
| ATG16L2 | HCG27 | 0.574791 | 2.29E-48 |
| TSC1 | AC022400.1 | 0.574865 | 2.21E-48 |
| ATG12 | OSMR-AS1 | 0.575023 | 2.06E-48 |
| KLHL24 | AC245014.3 | 0.575028 | 2.05E-48 |
| WDFY3 | AC084117.1 | 0.575051 | 2.03E-48 |
| WDFY3 | AL683813.1 | 0.575096 | 1.99E-48 |
| TP73 | UCKL1-AS1 | 0.575177 | 1.92E-48 |
| BIRC6 | AL117379.1 | 0.575183 | 1.91E-48 |
| MAPK8 | AC124283.3 | 0.575221 | 1.88E-48 |
| PIK3C3 | AC005070.3 | 0.575228 | 1.87E-48 |
| ATG2B | AC022211.1 | 0.575272 | 1.83E-48 |
| TSC1 | AL035587.1 | 0.575289 | 1.82E-48 |
| ATG12 | AC093388.1 | 0.575365 | 1.76E-48 |
| ATG12 | AC005856.1 | 0.5754 | 1.73E-48 |
| RB1CC1 | AP001381.1 | 0.575407 | 1.72E-48 |
| RPS6KB1 | AC022211.1 | 0.575412 | 1.72E-48 |
| KLHL24 | AC138932.5 | 0.575422 | 1.71E-48 |
| CFLAR | AL132989.1 | 0.575502 | 1.65E-48 |
| RB1CC1 | AC080162.1 | 0.575503 | 1.65E-48 |
| TSC1 | AC109460.2 | 0.575557 | 1.61E-48 |
| KLHL24 | AC008770.3 | 0.575591 | 1.58E-48 |
| PIK3C3 | Z82243.1 | 0.575608 | 1.57E-48 |
| FOXO1 | AC104695.3 | 0.575845 | 1.41E-48 |
| MAPK8 | LINC01355 | 0.57585 | 1.41E-48 |
| BIRC6 | USP46-AS1 | 0.575913 | 1.37E-48 |
| NAF1 | AC007878.1 | 0.576144 | 1.23E-48 |
| TSC1 | AC024361.1 | 0.576156 | 1.22E-48 |
| CFLAR | AC234775.3 | 0.576222 | 1.18E-48 |
| ATG12 | PWAR6 | 0.576255 | 1.17E-48 |
| CFLAR | AC005021.1 | 0.576259 | 1.16E-48 |
| PTEN | AC002553.1 | 0.576276 | 1.16E-48 |
| PIK3C3 | KLF7-IT1 | 0.57629 | 1.15E-48 |
| TSC1 | AL139011.1 | 0.576317 | 1.13E-48 |
| ATG12 | AC127024.4 | 0.576376 | 1.10E-48 |
| KLHL24 | AC007546.1 | 0.576382 | 1.10E-48 |
| BIRC6 | UGDH-AS1 | 0.576432 | 1.08E-48 |
| WDFY3 | TAPT1-AS1 | 0.576512 | 1.04E-48 |
| MAPK8 | AL049840.1 | 0.576513 | 1.04E-48 |
| ATG2B | AC008870.2 | 0.576524 | 1.03E-48 |
| ATG12 | AC024933.1 | 0.576669 | 9.63E-49 |
| MAPK8 | AC022150.4 | 0.576775 | 9.18E-49 |
| KLHL24 | ANKRD44-IT1 | 0.576802 | 9.06E-49 |
| PTEN | AC012181.2 | 0.576885 | 8.72E-49 |
| CFLAR | AC010245.2 | 0.576945 | 8.48E-49 |
| MAPK8 | AC007684.1 | 0.577001 | 8.27E-49 |
| FOXO1 | AC110792.3 | 0.577082 | 7.96E-49 |
| CFLAR | AL391834.1 | 0.57712 | 7.82E-49 |
| ATG12 | PAXBP1-AS1 | 0.577222 | 7.46E-49 |
| NAF1 | NUTM2B-AS1 | 0.577338 | 7.07E-49 |
| NAF1 | AC090948.2 | 0.5774 | 6.87E-49 |
| NAF1 | AL353804.1 | 0.577508 | 6.53E-49 |
| BIRC6 | AC079907.1 | 0.577559 | 6.38E-49 |
| FOXO1 | AP000786.1 | 0.577582 | 6.32E-49 |
| EIF2AK2 | N4BP2L2-IT2 | 0.577604 | 6.25E-49 |
| BIRC6 | AC026368.1 | 0.577648 | 6.12E-49 |
| EIF2AK2 | AC107027.3 | 0.577667 | 6.07E-49 |
| ATG16L2 | AL513320.1 | 0.577687 | 6.02E-49 |
| MAPK8 | AC026124.2 | 0.577743 | 5.86E-49 |
| BIRC6 | AC055822.1 | 0.577784 | 5.75E-49 |
| MAPK8 | ACAP2-IT1 | 0.577796 | 5.72E-49 |
| EIF2AK2 | SCARNA9 | 0.577799 | 5.71E-49 |
| FOXO1 | AL049552.1 | 0.577811 | 5.68E-49 |
| MAPK8 | LINC00216 | 0.577865 | 5.54E-49 |
| WDFY3 | AL606834.1 | 0.577923 | 5.39E-49 |
| KLHL24 | AP001381.1 | 0.578029 | 5.13E-49 |
| RPS6KB1 | AF117829.1 | 0.578034 | 5.12E-49 |
| EIF2AK2 | AC002553.2 | 0.57806 | 5.06E-49 |
| ATG16L2 | AC012645.3 | 0.578066 | 5.04E-49 |
| RB1CC1 | AC007878.1 | 0.578088 | 4.99E-49 |
| NAF1 | AC100830.2 | 0.578143 | 4.87E-49 |
| WDFY3 | AC098484.1 | 0.578242 | 4.65E-49 |
| TSC1 | AL117379.1 | 0.578261 | 4.61E-49 |
| EIF2AK2 | AC108010.1 | 0.578348 | 4.42E-49 |
| NAF1 | AC124312.2 | 0.578377 | 4.37E-49 |
| ATG2B | AC005540.1 | 0.578417 | 4.29E-49 |
| ATG2B | AC037198.2 | 0.578515 | 4.09E-49 |
| PIK3C3 | LINC00216 | 0.578526 | 4.07E-49 |
| EIF2AK2 | AC004492.1 | 0.578641 | 3.86E-49 |
| WDFY3 | AC008969.1 | 0.578642 | 3.86E-49 |
| KLHL24 | AC090739.1 | 0.578684 | 3.79E-49 |
| CFLAR | AL136320.1 | 0.578697 | 3.76E-49 |
| MAPK8 | AL157392.3 | 0.578729 | 3.71E-49 |
| KLHL24 | AF178030.1 | 0.578742 | 3.68E-49 |
| PTEN | AL357060.1 | 0.578811 | 3.57E-49 |
| CFLAR | AL133330.1 | 0.578842 | 3.52E-49 |
| WDFY3 | AC004656.1 | 0.578863 | 3.48E-49 |
| MAPK8 | AC006059.1 | 0.578893 | 3.43E-49 |
| ATG12 | AC120349.1 | 0.578942 | 3.36E-49 |
| KLHL24 | AC242426.2 | 0.578994 | 3.28E-49 |
| ATG2B | AP006621.2 | 0.579072 | 3.16E-49 |
| KLHL24 | AC090579.1 | 0.579213 | 2.96E-49 |
| CFLAR | LINC01578 | 0.57922 | 2.95E-49 |
| FOXO1 | AL109614.1 | 0.579225 | 2.94E-49 |
| MAPK8 | N4BP2L2-IT2 | 0.579299 | 2.84E-49 |
| FOXO1 | NEAT1 | 0.579361 | 2.76E-49 |
| WIPI2 | AC092171.2 | 0.57937 | 2.75E-49 |
| ATG2B | AC004477.3 | 0.57937 | 2.75E-49 |
| FOXO1 | AC020913.3 | 0.579389 | 2.73E-49 |
| CFLAR | AP000692.1 | 0.579426 | 2.68E-49 |
| KLHL24 | LINC00630 | 0.579467 | 2.63E-49 |
| FOXO1 | AL133371.2 | 0.57947 | 2.62E-49 |
| ATG16L2 | AL021707.6 | 0.579471 | 2.62E-49 |
| NLRC4 | AL731567.1 | 0.579489 | 2.60E-49 |
| EIF2AK2 | AC008906.1 | 0.579501 | 2.59E-49 |
| PTEN | AL137003.2 | 0.579548 | 2.53E-49 |
| PTEN | KDM4A-AS1 | 0.57961 | 2.46E-49 |
| MAPK8 | AC096586.2 | 0.579629 | 2.44E-49 |
| NAF1 | AC007038.1 | 0.579728 | 2.33E-49 |
| RB1CC1 | AC022973.3 | 0.579745 | 2.31E-49 |
| ATG12 | PPP3CB-AS1 | 0.579772 | 2.28E-49 |
| MAPK8 | AC002553.2 | 0.579772 | 2.28E-49 |
| MAPK8 | AL158166.2 | 0.57981 | 2.24E-49 |
| PTEN | AC090517.2 | 0.579844 | 2.20E-49 |
| KLHL24 | AL354989.1 | 0.579881 | 2.17E-49 |
| WDFY3 | AL158212.3 | 0.579883 | 2.16E-49 |
| MAPK8 | AL139407.1 | 0.57989 | 2.16E-49 |
| ATG12 | AL137782.1 | 0.579896 | 2.15E-49 |
| BIRC6 | AL031716.1 | 0.579914 | 2.13E-49 |
| CFLAR | NEAT1 | 0.579975 | 2.07E-49 |
| ATG16L2 | ZNF213-AS1 | 0.580039 | 2.01E-49 |
| PTEN | AC084824.5 | 0.580051 | 2.00E-49 |
| ATG12 | AC138956.1 | 0.580083 | 1.97E-49 |
| ATG2B | ARHGEF38-IT1 | 0.580094 | 1.96E-49 |
| PIK3C3 | AC018752.1 | 0.580189 | 1.87E-49 |
| PTEN | AC008537.2 | 0.580205 | 1.86E-49 |
| MAPK8 | AL138963.1 | 0.580206 | 1.86E-49 |
| CFLAR | AC009090.3 | 0.580228 | 1.84E-49 |
| PIK3C3 | AL158166.2 | 0.5803 | 1.78E-49 |
| EIF2AK2 | NPTN-IT1 | 0.580331 | 1.75E-49 |
| ATG2B | AL450263.1 | 0.580358 | 1.73E-49 |
| WDFY3 | AL359715.3 | 0.580381 | 1.71E-49 |
| TSC1 | AC232271.1 | 0.580403 | 1.70E-49 |
| PIK3C3 | AL049840.1 | 0.580501 | 1.62E-49 |
| BIRC6 | AC025178.1 | 0.580513 | 1.61E-49 |
| MAPK8 | AC084824.4 | 0.580523 | 1.60E-49 |
| MAPK8 | AC090425.2 | 0.580553 | 1.58E-49 |
| KLHL24 | AC096992.2 | 0.580564 | 1.57E-49 |
| PTEN | AC022306.2 | 0.580573 | 1.57E-49 |
| PTEN | AL139011.1 | 0.580581 | 1.56E-49 |
| MAPK8 | AL049552.1 | 0.580583 | 1.56E-49 |
| BIRC6 | AC048341.1 | 0.580609 | 1.54E-49 |
| PTEN | AC016394.1 | 0.580619 | 1.53E-49 |
| NAF1 | LIMS1-AS1 | 0.580657 | 1.51E-49 |
| PTEN | NARF-IT1 | 0.580666 | 1.50E-49 |
| MAPK8 | MCM3AP-AS1 | 0.580672 | 1.50E-49 |
| WDFY3 | AC092123.1 | 0.580706 | 1.47E-49 |
| RB1CC1 | AL163051.2 | 0.58071 | 1.47E-49 |
| ATG16L2 | AC073335.2 | 0.580745 | 1.45E-49 |
| ATG12 | AC010245.2 | 0.580746 | 1.44E-49 |
| EIF2AK2 | AC012557.1 | 0.580799 | 1.41E-49 |
| WDFY3 | GABPB1-AS1 | 0.580895 | 1.35E-49 |
| EIF2AK2 | MAL2-AS1 | 0.581103 | 1.22E-49 |
| ATG12 | MIATNB | 0.581135 | 1.20E-49 |
| MAPK8 | AL139120.1 | 0.581212 | 1.16E-49 |
| PIK3C3 | FTX | 0.581261 | 1.13E-49 |
| ATG12 | AC020915.2 | 0.581276 | 1.13E-49 |
| ATG12 | AC016590.2 | 0.581346 | 1.09E-49 |
| PIK3C3 | AC100830.2 | 0.581367 | 1.08E-49 |
| ATG16L2 | AL136295.2 | 0.581378 | 1.07E-49 |
| PIK3C3 | AC092801.1 | 0.58138 | 1.07E-49 |
| NAF1 | AC010761.3 | 0.581389 | 1.07E-49 |
| FOXO1 | AC007546.1 | 0.581413 | 1.06E-49 |
| KLHL24 | AC007878.1 | 0.581417 | 1.05E-49 |
| PIK3C3 | AL139120.1 | 0.581427 | 1.05E-49 |
| BIRC6 | AP003486.1 | 0.581468 | 1.03E-49 |
| NAF1 | Z82243.1 | 0.581479 | 1.02E-49 |
| ATG2B | AC073046.1 | 0.581532 | 9.99E-50 |
| PIK3C3 | AC005838.2 | 0.581565 | 9.84E-50 |
| ATG12 | GARS-DT | 0.581577 | 9.78E-50 |
| CFLAR | AC012181.1 | 0.581653 | 9.44E-50 |
| CFLAR | CCDC18-AS1 | 0.581656 | 9.42E-50 |
| FOXO1 | SDCBP2-AS1 | 0.581724 | 9.13E-50 |
| FOXO1 | AP002907.1 | 0.581731 | 9.10E-50 |
| BIRC6 | AC090617.5 | 0.58177 | 8.93E-50 |
| KLHL24 | AL139120.1 | 0.581861 | 8.56E-50 |
| ATG12 | AC022150.4 | 0.581869 | 8.53E-50 |
| NAF1 | AC018752.1 | 0.581919 | 8.32E-50 |
| PIK3C3 | LIMS1-AS1 | 0.582042 | 7.86E-50 |
| ATG12 | AL513550.1 | 0.58205 | 7.83E-50 |
| KLHL24 | AC068790.5 | 0.582075 | 7.74E-50 |
| RB1CC1 | AC004492.1 | 0.582102 | 7.64E-50 |
| EIF2AK2 | AC048344.4 | 0.58223 | 7.19E-50 |
| NAF1 | MCM3AP-AS1 | 0.582314 | 6.91E-50 |
| CFLAR | AC015813.1 | 0.582333 | 6.85E-50 |
| TSC1 | LINC02035 | 0.582341 | 6.83E-50 |
| WDFY3 | AL356356.1 | 0.582349 | 6.80E-50 |
| BIRC6 | AC008537.2 | 0.582363 | 6.75E-50 |
| RPS6KB1 | AL133243.2 | 0.582408 | 6.61E-50 |
| ATG12 | AL359962.2 | 0.582427 | 6.55E-50 |
| PTEN | SNHG14 | 0.582445 | 6.50E-50 |
| PIK3C3 | AC008770.3 | 0.582471 | 6.42E-50 |
| PIK3C3 | AL645568.1 | 0.582492 | 6.36E-50 |
| MAPK8 | AC016831.4 | 0.582493 | 6.35E-50 |
| EIF2AK2 | DLEU2 | 0.582497 | 6.34E-50 |
| MAPK8 | GMDS-DT | 0.582544 | 6.20E-50 |
| WDFY3 | USP46-AS1 | 0.582644 | 5.92E-50 |
| ATG16L2 | AP006623.1 | 0.582661 | 5.87E-50 |
| ATG16L2 | RAD51-AS1 | 0.582742 | 5.65E-50 |
| PTEN | AP001625.2 | 0.582756 | 5.61E-50 |
| WDFY3 | ALMS1-IT1 | 0.582773 | 5.57E-50 |
| EIF2AK2 | AC234775.3 | 0.582845 | 5.38E-50 |
| ATG12 | AC093799.1 | 0.582863 | 5.34E-50 |
| ATG2B | MIATNB | 0.582886 | 5.28E-50 |
| MAPK8 | AC007878.1 | 0.582913 | 5.21E-50 |
| KLHL24 | SDCBP2-AS1 | 0.582947 | 5.13E-50 |
| KLHL24 | AC058791.1 | 0.582985 | 5.04E-50 |
| ATG12 | AL031775.2 | 0.582992 | 5.02E-50 |
| BIRC6 | RAB30-AS1 | 0.583015 | 4.97E-50 |
| PTEN | AC008669.1 | 0.583033 | 4.92E-50 |
| PTEN | AC242426.2 | 0.583045 | 4.90E-50 |
| PTEN | AL359921.1 | 0.583131 | 4.70E-50 |
| PIK3C3 | GMDS-DT | 0.583136 | 4.69E-50 |
| ATG12 | AC087286.1 | 0.583158 | 4.64E-50 |
| CFLAR | AP000766.1 | 0.583214 | 4.52E-50 |
| PIK3C3 | AC087276.1 | 0.583273 | 4.40E-50 |
| EIF2AK2 | AC007878.1 | 0.583287 | 4.37E-50 |
| PIK3C3 | AC006059.1 | 0.583346 | 4.25E-50 |
| DNAJB9 | CARD8-AS1 | 0.583436 | 4.07E-50 |
| FOXO1 | AC005856.1 | 0.58346 | 4.02E-50 |
| FOXO1 | AC025917.1 | 0.583471 | 4.00E-50 |
| RB1CC1 | AC007684.1 | 0.583576 | 3.81E-50 |
| FOXO1 | AC096921.2 | 0.583599 | 3.77E-50 |
| CFLAR | AC093495.1 | 0.58373 | 3.54E-50 |
| RB1CC1 | AP001033.2 | 0.583761 | 3.49E-50 |
| NAF1 | Z83843.1 | 0.583818 | 3.40E-50 |
| ATG12 | AL592148.3 | 0.583822 | 3.39E-50 |
| PIK3C3 | AP001033.2 | 0.58386 | 3.33E-50 |
| KLHL24 | EBLN3P | 0.583883 | 3.29E-50 |
| CASP1 | CARD8-AS1 | 0.583942 | 3.20E-50 |
| ATG12 | AP000873.2 | 0.58395 | 3.19E-50 |
| CFLAR | AC090948.2 | 0.584073 | 3.01E-50 |
| ATG2B | LINC02352 | 0.584124 | 2.94E-50 |
| NLRC4 | MIR3945HG | 0.584136 | 2.92E-50 |
| CAPN10 | CAPN10-DT | 0.584138 | 2.92E-50 |
| ATG2B | LINC01534 | 0.584144 | 2.91E-50 |
| RB1CC1 | AC138393.3 | 0.584169 | 2.88E-50 |
| FOXO1 | AC108449.2 | 0.58419 | 2.85E-50 |
| PTEN | AC018690.1 | 0.584207 | 2.82E-50 |
| PIK3C3 | AL590723.1 | 0.584216 | 2.81E-50 |
| MAPK8 | LAMC1-AS1 | 0.584243 | 2.78E-50 |
| MAPK8 | AL133243.2 | 0.584245 | 2.77E-50 |
| PTEN | AP003486.1 | 0.584276 | 2.73E-50 |
| FOXO1 | AC008966.2 | 0.584328 | 2.67E-50 |
| ATG12 | AC084824.4 | 0.584366 | 2.62E-50 |
| PTEN | AC133644.2 | 0.584378 | 2.60E-50 |
| ATG12 | A2M-AS1 | 0.584407 | 2.57E-50 |
| GOPC | TRAF3IP2-AS1 | 0.584452 | 2.51E-50 |
| WDFY3 | AC008035.1 | 0.584568 | 2.38E-50 |
| FOXO1 | AC007014.2 | 0.584609 | 2.33E-50 |
| CASP1 | PCED1B-AS1 | 0.584647 | 2.29E-50 |
| ATG12 | MIR181A2HG | 0.584678 | 2.26E-50 |
| RB1CC1 | AC026124.2 | 0.584711 | 2.22E-50 |
| CCR2 | LINC00426 | 0.584716 | 2.22E-50 |
| NAF1 | AL049840.5 | 0.584742 | 2.19E-50 |
| PTEN | AL021707.7 | 0.584959 | 1.98E-50 |
| PTEN | AC005540.1 | 0.585015 | 1.92E-50 |
| ATG2B | AL136531.1 | 0.585124 | 1.83E-50 |
| PTEN | AL157932.1 | 0.585259 | 1.71E-50 |
| BIRC6 | AC022306.2 | 0.585282 | 1.69E-50 |
| BIRC6 | AL136531.1 | 0.585304 | 1.68E-50 |
| PIK3C3 | AL117381.1 | 0.585339 | 1.65E-50 |
| PTEN | ALMS1-IT1 | 0.585341 | 1.65E-50 |
| MAPK8 | AL136115.2 | 0.5854 | 1.60E-50 |
| CXCR4 | PCED1B-AS1 | 0.585448 | 1.57E-50 |
| CFLAR | AL354989.1 | 0.585465 | 1.55E-50 |
| PTEN | AC078883.1 | 0.585615 | 1.45E-50 |
| FOXO1 | AC138932.5 | 0.585636 | 1.43E-50 |
| ATG12 | AL157392.3 | 0.585733 | 1.37E-50 |
| TSC1 | AC024075.3 | 0.585796 | 1.33E-50 |
| PIK3C3 | AC098484.1 | 0.585855 | 1.29E-50 |
| ATG2B | AC138207.4 | 0.585956 | 1.23E-50 |
| CAPN10 | AL390719.2 | 0.585967 | 1.22E-50 |
| ATG12 | LINC00852 | 0.586003 | 1.20E-50 |
| PTEN | AL442125.2 | 0.586007 | 1.20E-50 |
| PIK3C3 | AC022400.5 | 0.586085 | 1.16E-50 |
| ITGA6 | AL049555.1 | 0.586106 | 1.14E-50 |
| BIRC6 | AC087752.4 | 0.586155 | 1.12E-50 |
| RB1CC1 | AC087276.1 | 0.586245 | 1.07E-50 |
| PIK3C3 | AC004832.5 | 0.586493 | 9.51E-51 |
| ATG12 | AP000866.6 | 0.586607 | 9.00E-51 |
| ATG2B | AC007938.3 | 0.586637 | 8.88E-51 |
| ATG2B | GEMIN7-AS1 | 0.586662 | 8.77E-51 |
| CASP1 | PSMB8-AS1 | 0.586671 | 8.73E-51 |
| ATG4B | AC132872.3 | 0.586844 | 8.04E-51 |
| CFLAR | NPTN-IT1 | 0.586895 | 7.84E-51 |
| ATG2B | AC093495.1 | 0.586943 | 7.67E-51 |
| MAPK8 | AC079684.1 | 0.586975 | 7.55E-51 |
| MAPK8 | LINC00630 | 0.58699 | 7.50E-51 |
| NAF1 | AL117381.1 | 0.587034 | 7.34E-51 |
| BIRC6 | NORAD | 0.58708 | 7.18E-51 |
| NAF1 | NPTN-IT1 | 0.587109 | 7.08E-51 |
| MAPK8 | AC053513.1 | 0.587178 | 6.85E-51 |
| NAF1 | AC006059.1 | 0.587218 | 6.72E-51 |
| ATG2B | AC120349.1 | 0.587447 | 6.02E-51 |
| EIF2AK2 | AC090948.2 | 0.587451 | 6.01E-51 |
| MAPK8 | AC005070.3 | 0.58755 | 5.73E-51 |
| ATG12 | AC245014.3 | 0.587633 | 5.51E-51 |
| CFLAR | AL662844.3 | 0.587644 | 5.48E-51 |
| ATG2B | AC087286.1 | 0.587672 | 5.40E-51 |
| FOXO1 | AC234775.3 | 0.58768 | 5.38E-51 |
| ATG12 | AC004466.3 | 0.587685 | 5.37E-51 |
| ATG12 | AC087392.1 | 0.587714 | 5.30E-51 |
| BIRC6 | AC020978.3 | 0.587738 | 5.23E-51 |
| WDFY3 | DGCR11 | 0.587798 | 5.09E-51 |
| KLHL24 | AC016831.4 | 0.587808 | 5.06E-51 |
| ATG12 | Z82243.1 | 0.587837 | 4.99E-51 |
| MAPK8 | AL109614.1 | 0.587917 | 4.80E-51 |
| MAPK8 | AC058791.1 | 0.587985 | 4.65E-51 |
| FOXO1 | AC124312.4 | 0.588025 | 4.56E-51 |
| CAPN10 | AC069281.2 | 0.588072 | 4.46E-51 |
| ATG2B | AL157786.1 | 0.58813 | 4.34E-51 |
| ATG16L2 | AC084018.1 | 0.588151 | 4.29E-51 |
| KLHL24 | AC068790.2 | 0.588182 | 4.23E-51 |
| ATG2B | AC021851.1 | 0.588183 | 4.23E-51 |
| CFLAR | AC015849.3 | 0.588251 | 4.09E-51 |
| EIF2AK2 | AC022150.4 | 0.588258 | 4.08E-51 |
| CFLAR | LIMS1-AS1 | 0.588271 | 4.05E-51 |
| PTEN | AC010245.2 | 0.588467 | 3.69E-51 |
| FOXO1 | AC087276.1 | 0.588481 | 3.66E-51 |
| BIRC6 | AC004253.1 | 0.588592 | 3.47E-51 |
| ATG12 | AC004223.3 | 0.588644 | 3.38E-51 |
| PTEN | AL132989.1 | 0.588659 | 3.36E-51 |
| MAPK8 | AC007216.3 | 0.588667 | 3.35E-51 |
| FOXO1 | AL136320.1 | 0.588683 | 3.32E-51 |
| CFLAR | AC025917.1 | 0.588792 | 3.15E-51 |
| ATG12 | LINC00894 | 0.588832 | 3.09E-51 |
| MAPK8 | SDCBP2-AS1 | 0.588887 | 3.01E-51 |
| PTEN | AC009041.4 | 0.588893 | 3.00E-51 |
| ATG2B | AP001625.2 | 0.58895 | 2.92E-51 |
| NAF1 | AC092801.1 | 0.588965 | 2.90E-51 |
| ATG2B | MBNL1-AS1 | 0.588977 | 2.88E-51 |
| MAPK8 | AL049840.5 | 0.58905 | 2.78E-51 |
| PIK3C3 | HCG18 | 0.589075 | 2.75E-51 |
| EIF2AK2 | AC019080.5 | 0.589135 | 2.67E-51 |
| ATG12 | AL137779.2 | 0.589142 | 2.66E-51 |
| WDFY3 | AL158166.1 | 0.589251 | 2.53E-51 |
| MAPK8 | AC100830.2 | 0.589291 | 2.48E-51 |
| ATG16L2 | AC009065.4 | 0.589297 | 2.47E-51 |
| ATG2B | AC048344.4 | 0.58943 | 2.32E-51 |
| PTEN | LINC00894 | 0.589443 | 2.30E-51 |
| ATG12 | ERVK13-1 | 0.589459 | 2.29E-51 |
| ATG2B | MAST4-AS1 | 0.589487 | 2.25E-51 |
| CCR2 | TRG-AS1 | 0.589514 | 2.22E-51 |
| NAF1 | AC005838.2 | 0.589546 | 2.19E-51 |
| ATG4B | AL390719.2 | 0.589656 | 2.08E-51 |
| NAF1 | TRAF3IP2-AS1 | 0.590033 | 1.73E-51 |
| ATG2B | AC245884.8 | 0.590049 | 1.72E-51 |
| MAPK8 | AC010761.3 | 0.590114 | 1.66E-51 |
| IL24 | LINC01781 | 0.590143 | 1.64E-51 |
| ATG2B | AL049840.3 | 0.590174 | 1.62E-51 |
| FOXO1 | OIP5-AS1 | 0.590263 | 1.55E-51 |
| NAF1 | FTX | 0.590291 | 1.53E-51 |
| MAPK8 | TRAF3IP2-AS1 | 0.590314 | 1.51E-51 |
| NAF1 | HCG18 | 0.59034 | 1.49E-51 |
| RB1CC1 | LINC00216 | 0.590371 | 1.47E-51 |
| ATG2B | AC097641.2 | 0.590413 | 1.44E-51 |
| PTEN | AC006378.1 | 0.590459 | 1.41E-51 |
| ATG12 | ARHGEF38-IT1 | 0.590492 | 1.39E-51 |
| RB1CC1 | AC010834.3 | 0.590511 | 1.37E-51 |
| KLHL24 | AC108449.2 | 0.590516 | 1.37E-51 |
| KLHL24 | AC002553.2 | 0.590551 | 1.35E-51 |
| FOXO1 | AC004832.5 | 0.59064 | 1.29E-51 |
| ATG2B | RFX3-AS1 | 0.590695 | 1.26E-51 |
| CFLAR | RRN3P2 | 0.590776 | 1.21E-51 |
| BIRC6 | AC015813.1 | 0.590792 | 1.20E-51 |
| CFLAR | AC090948.1 | 0.590818 | 1.18E-51 |
| ATG16L2 | AC116914.2 | 0.590863 | 1.16E-51 |
| BIRC6 | MCCC1-AS1 | 0.590924 | 1.12E-51 |
| TSC1 | RAD51-AS1 | 0.590972 | 1.10E-51 |
| ATG12 | AC006378.1 | 0.590985 | 1.09E-51 |
| ATG2B | AC141002.1 | 0.591002 | 1.08E-51 |
| KLHL24 | AL157392.3 | 0.591053 | 1.06E-51 |
| MAPK8 | AL590723.1 | 0.591071 | 1.05E-51 |
| ATG2B | AL359697.1 | 0.591088 | 1.04E-51 |
| ATG16L2 | LINC01089 | 0.591146 | 1.01E-51 |
| BIRC6 | AC092953.2 | 0.591269 | 9.50E-52 |
| PTEN | AC009948.1 | 0.591293 | 9.39E-52 |
| ATG12 | AL021578.1 | 0.591299 | 9.36E-52 |
| ATG2B | ATP1A1-AS1 | 0.591319 | 9.27E-52 |
| MAPK8 | AC092801.1 | 0.591323 | 9.26E-52 |
| FOXO1 | AC063965.1 | 0.591342 | 9.17E-52 |
| TSC1 | AC137932.3 | 0.591372 | 9.04E-52 |
| BIRC6 | AP003392.1 | 0.591452 | 8.69E-52 |
| TSC1 | AC004253.1 | 0.591601 | 8.09E-52 |
| KLHL24 | BTBD9-AS1 | 0.591627 | 7.99E-52 |
| FOXO1 | AC108727.1 | 0.591627 | 7.98E-52 |
| BIRC6 | AC068792.1 | 0.591643 | 7.92E-52 |
| FOXO1 | LIMS1-AS1 | 0.591663 | 7.84E-52 |
| CFLAR | AL359076.1 | 0.591698 | 7.71E-52 |
| PTEN | AC079684.1 | 0.591794 | 7.36E-52 |
| PTEN | AP001469.2 | 0.591822 | 7.26E-52 |
| ATG2B | AC007552.2 | 0.591915 | 6.94E-52 |
| WDFY3 | AC135050.5 | 0.591922 | 6.91E-52 |
| TP73 | AL121899.1 | 0.592004 | 6.64E-52 |
| ATG12 | LINC01578 | 0.5921 | 6.34E-52 |
| ATG2B | AC009948.1 | 0.592104 | 6.33E-52 |
| EIF2AK2 | Z68871.1 | 0.592135 | 6.23E-52 |
| ATG12 | AP001432.1 | 0.592145 | 6.20E-52 |
| FOXO1 | AC007684.1 | 0.592261 | 5.86E-52 |
| TSC1 | AL158212.3 | 0.592291 | 5.78E-52 |
| WDFY3 | AP006621.2 | 0.59235 | 5.61E-52 |
| ATG12 | AC002550.2 | 0.592356 | 5.60E-52 |
| FOXO1 | AC080162.1 | 0.592459 | 5.32E-52 |
| ATG12 | AC008669.1 | 0.592525 | 5.15E-52 |
| NAF1 | AP000766.1 | 0.592559 | 5.07E-52 |
| NAF1 | AC025917.1 | 0.592601 | 4.97E-52 |
| RB1CC1 | AP002907.1 | 0.59265 | 4.85E-52 |
| BIRC6 | AF131215.5 | 0.592651 | 4.85E-52 |
| PTEN | AC096992.2 | 0.59267 | 4.80E-52 |
| FOXO1 | AC090948.1 | 0.592714 | 4.70E-52 |
| ATG2B | AC018809.2 | 0.592721 | 4.68E-52 |
| MAPK8 | AC011939.2 | 0.592852 | 4.39E-52 |
| CFLAR | AL139407.1 | 0.592895 | 4.30E-52 |
| RAB24 | AC069281.2 | 0.593078 | 3.93E-52 |
| PIK3C3 | AL049840.5 | 0.593282 | 3.56E-52 |
| ATG12 | AC010536.2 | 0.59331 | 3.51E-52 |
| NAF1 | AP001178.2 | 0.593433 | 3.31E-52 |
| FOXO1 | AC087286.2 | 0.593499 | 3.20E-52 |
| EIF2AK2 | AC253536.3 | 0.593524 | 3.16E-52 |
| KLHL24 | AL133243.2 | 0.593567 | 3.10E-52 |
| RB1CC1 | Z68871.1 | 0.593578 | 3.08E-52 |
| ATG2B | AC007566.1 | 0.593615 | 3.02E-52 |
| BIRC6 | TPT1-AS1 | 0.593757 | 2.82E-52 |
| BIRC6 | AL035409.1 | 0.593768 | 2.80E-52 |
| ATG2B | AC096992.2 | 0.593978 | 2.53E-52 |
| PTEN | AC007566.1 | 0.594018 | 2.48E-52 |
| RB1CC1 | AC005070.3 | 0.594028 | 2.47E-52 |
| BIRC6 | MUC20-OT1 | 0.594084 | 2.40E-52 |
| PIK3C3 | Z68871.1 | 0.594156 | 2.32E-52 |
| WDFY3 | AC011676.1 | 0.594173 | 2.30E-52 |
| NAF1 | AC022150.4 | 0.594292 | 2.17E-52 |
| BIRC6 | AL354696.1 | 0.59438 | 2.08E-52 |
| BIRC6 | AC009948.1 | 0.594384 | 2.07E-52 |
| ATG2B | AC008969.1 | 0.59443 | 2.03E-52 |
| TSC1 | AP003392.1 | 0.594578 | 1.88E-52 |
| ATG2B | AL121839.2 | 0.594691 | 1.78E-52 |
| PTEN | AL117336.2 | 0.594768 | 1.72E-52 |
| ATG12 | AC066613.1 | 0.594777 | 1.71E-52 |
| ATG2B | AC011472.4 | 0.594863 | 1.64E-52 |
| ATG12 | AL365277.1 | 0.594951 | 1.57E-52 |
| ATG12 | AL645568.1 | 0.594955 | 1.56E-52 |
| PTEN | AC009090.1 | 0.594955 | 1.56E-52 |
| KLHL24 | AC007216.3 | 0.594956 | 1.56E-52 |
| CFLAR | AC008966.2 | 0.594963 | 1.56E-52 |
| KLHL24 | AC097376.2 | 0.594986 | 1.54E-52 |
| MAPK8 | AP001033.2 | 0.595043 | 1.50E-52 |
| BIRC6 | AC005519.1 | 0.595068 | 1.48E-52 |
| ATG2B | AL353804.2 | 0.595143 | 1.43E-52 |
| WDFY3 | AC004067.1 | 0.595169 | 1.41E-52 |
| BIRC6 | AL606834.1 | 0.595212 | 1.38E-52 |
| PTEN | AC115989.1 | 0.595317 | 1.31E-52 |
| RB1CC1 | HCG18 | 0.595466 | 1.22E-52 |
| ATG12 | AC007216.4 | 0.595497 | 1.20E-52 |
| CAPN10 | AL513320.1 | 0.595521 | 1.18E-52 |
| ATG2B | AC087481.3 | 0.595565 | 1.16E-52 |
| PIK3C3 | SNHG14 | 0.595617 | 1.13E-52 |
| TSC1 | INE1 | 0.595681 | 1.09E-52 |
| KLHL24 | AC024075.3 | 0.595737 | 1.06E-52 |
| PIK3C3 | AC090948.2 | 0.595743 | 1.06E-52 |
| ATG2B | AL021707.7 | 0.59578 | 1.04E-52 |
| MAPK8 | CR936218.1 | 0.59583 | 1.02E-52 |
| FOXO1 | ACAP2-IT1 | 0.595943 | 9.61E-53 |
| KLHL24 | AC007216.4 | 0.595951 | 9.57E-53 |
| NAF1 | LINC00630 | 0.595979 | 9.44E-53 |
| ATG2B | AP003486.1 | 0.596034 | 9.19E-53 |
| ATG2B | AC096921.2 | 0.596058 | 9.08E-53 |
| BIRC6 | RBMS3-AS3 | 0.596159 | 8.64E-53 |
| ATG2B | AC005261.1 | 0.596205 | 8.44E-53 |
| ATG12 | AC009318.2 | 0.596265 | 8.20E-53 |
| CFLAR | AL157786.1 | 0.596273 | 8.17E-53 |
| ATG12 | AC098851.1 | 0.596286 | 8.11E-53 |
| PIK3C3 | AC021851.1 | 0.596287 | 8.11E-53 |
| MAPK8 | AC073487.1 | 0.596294 | 8.08E-53 |
| NAF1 | AC053513.1 | 0.596399 | 7.67E-53 |
| TSC1 | AC008969.1 | 0.596419 | 7.60E-53 |
| ATG12 | AC004908.2 | 0.596487 | 7.35E-53 |
| ATG2B | AC087392.1 | 0.596505 | 7.28E-53 |
| TP73 | AC108134.4 | 0.59659 | 6.98E-53 |
| WDFY3 | AP003392.1 | 0.5967 | 6.61E-53 |
| BIRC6 | LINC01389 | 0.596716 | 6.56E-53 |
| ATG12 | AC007552.2 | 0.596724 | 6.53E-53 |
| ATG2B | AL137003.2 | 0.59679 | 6.32E-53 |
| LAMP1 | LINC01003 | 0.596821 | 6.22E-53 |
| WDFY3 | AL035409.1 | 0.597005 | 5.68E-53 |
| PTEN | AL354733.3 | 0.597055 | 5.54E-53 |
| KLHL24 | AC012557.1 | 0.597101 | 5.42E-53 |
| ATG16L2 | AC011498.6 | 0.597126 | 5.35E-53 |
| ATG2B | AC093388.1 | 0.597139 | 5.32E-53 |
| BIRC6 | AL137003.2 | 0.597154 | 5.28E-53 |
| PTEN | LINC00641 | 0.597171 | 5.23E-53 |
| MAPK8 | AC097376.2 | 0.597371 | 4.74E-53 |
| FOXO1 | AC005070.3 | 0.597443 | 4.57E-53 |
| PTEN | LINC02352 | 0.597463 | 4.53E-53 |
| PTEN | AC078846.1 | 0.597627 | 4.17E-53 |
| FOXO1 | AL138963.1 | 0.597658 | 4.11E-53 |
| ATG12 | AL354989.1 | 0.597757 | 3.91E-53 |
| FOXO1 | N4BP2L2-IT2 | 0.597843 | 3.75E-53 |
| FOXO1 | AC007878.1 | 0.597895 | 3.65E-53 |
| FOXO1 | AP001033.2 | 0.5979 | 3.64E-53 |
| ATG16L2 | AC132872.3 | 0.597944 | 3.56E-53 |
| WDFY3 | AC068152.1 | 0.598005 | 3.46E-53 |
| MAPK8 | AL163051.2 | 0.598024 | 3.42E-53 |
| ATG12 | KIF26B-AS1 | 0.598063 | 3.36E-53 |
| ATG12 | ADNP-AS1 | 0.598079 | 3.33E-53 |
| BIRC6 | AC067817.2 | 0.598092 | 3.31E-53 |
| LAMP1 | AL356740.1 | 0.598097 | 3.30E-53 |
| PTEN | AC078778.1 | 0.598112 | 3.28E-53 |
| BIRC6 | AC084824.5 | 0.598216 | 3.11E-53 |
| TSC1 | FAM13A-AS1 | 0.598256 | 3.05E-53 |
| WDFY3 | AC004253.1 | 0.598282 | 3.01E-53 |
| CAPN10 | STAG3L5P-PVRIG2P-PILRB | 0.598319 | 2.96E-53 |
| ATG2B | AC084824.5 | 0.598548 | 2.64E-53 |
| ATG12 | AC018926.2 | 0.598578 | 2.60E-53 |
| ATG12 | AP002907.1 | 0.598904 | 2.21E-53 |
| BIRC6 | AL031717.1 | 0.598982 | 2.12E-53 |
| WDFY3 | AC090617.5 | 0.598997 | 2.11E-53 |
| TSC1 | AC002128.1 | 0.599016 | 2.09E-53 |
| PTEN | AL078581.1 | 0.599044 | 2.06E-53 |
| WDFY3 | AC005674.2 | 0.599098 | 2.00E-53 |
| ATG4B | ASMTL-AS1 | 0.599175 | 1.93E-53 |
| ATG2B | SNHG14 | 0.599521 | 1.62E-53 |
| RB1CC1 | GMDS-DT | 0.599579 | 1.58E-53 |
| FOXO1 | AL359076.1 | 0.599593 | 1.56E-53 |
| NAF1 | AC097376.2 | 0.599682 | 1.50E-53 |
| FOXO1 | MAL2-AS1 | 0.59977 | 1.43E-53 |
| PTEN | AC107068.1 | 0.59987 | 1.36E-53 |
| ATG2B | GARS-DT | 0.599973 | 1.29E-53 |
| EIF2AK2 | LINC00630 | 0.599981 | 1.29E-53 |
| ATG12 | HCG18 | 0.599987 | 1.28E-53 |
| WDFY3 | AC073569.2 | 0.600227 | 1.14E-53 |
| FOXO1 | AL157402.2 | 0.600472 | 1.01E-53 |
| ATG12 | AC092794.1 | 0.600549 | 9.68E-54 |
| CFLAR | AL512791.1 | 0.600555 | 9.66E-54 |
| CAPN10 | ASMTL-AS1 | 0.600696 | 8.99E-54 |
| ATG2B | AL359962.2 | 0.600722 | 8.88E-54 |
| MAPK8 | AC087276.1 | 0.600723 | 8.88E-54 |
| NAF1 | Z68871.1 | 0.600724 | 8.87E-54 |
| PTEN | AP003170.3 | 0.600784 | 8.60E-54 |
| CFLAR | AC008906.1 | 0.600853 | 8.31E-54 |
| FOXO1 | LINC-PINT | 0.60087 | 8.24E-54 |
| CFLAR | AC090948.3 | 0.601183 | 7.04E-54 |
| ATG12 | AL513008.1 | 0.60119 | 7.02E-54 |
| RB1CC1 | MAL2-AS1 | 0.601244 | 6.83E-54 |
| ATG2B | AC115989.1 | 0.601357 | 6.45E-54 |
| ATG2B | NFYC-AS1 | 0.601465 | 6.11E-54 |
| PTEN | AC127024.5 | 0.601674 | 5.50E-54 |
| WDFY3 | AC004908.3 | 0.601902 | 4.90E-54 |
| EIF2AK2 | AL354989.1 | 0.602012 | 4.63E-54 |
| ATG2B | AC027277.2 | 0.602036 | 4.58E-54 |
| BIRC6 | ZNF460-AS1 | 0.602109 | 4.41E-54 |
| ATG12 | AC000123.1 | 0.602211 | 4.19E-54 |
| WDFY3 | AC027097.1 | 0.602379 | 3.85E-54 |
| KLHL24 | GMDS-DT | 0.60241 | 3.79E-54 |
| CFLAR | AC012170.2 | 0.602426 | 3.76E-54 |
| ATG12 | AC015911.3 | 0.602528 | 3.57E-54 |
| EIF2AK2 | AC026356.1 | 0.602709 | 3.26E-54 |
| ATG12 | MAST4-AS1 | 0.602772 | 3.16E-54 |
| ATG2B | AC242426.2 | 0.602974 | 2.85E-54 |
| BIRC6 | AC011676.1 | 0.602984 | 2.84E-54 |
| ATG2B | AC079684.1 | 0.603021 | 2.78E-54 |
| ATG12 | MAL2-AS1 | 0.603123 | 2.64E-54 |
| ATG12 | ADAMTSL4-AS1 | 0.603271 | 2.45E-54 |
| FOXO1 | AL049840.5 | 0.603294 | 2.42E-54 |
| ATG12 | AC090579.1 | 0.603338 | 2.37E-54 |
| NLRC4 | AC083949.1 | 0.603355 | 2.35E-54 |
| ATG12 | AC002064.2 | 0.603406 | 2.29E-54 |
| WDFY3 | EGOT | 0.603415 | 2.28E-54 |
| ATG2B | AC104695.3 | 0.603595 | 2.08E-54 |
| PTEN | AC018809.2 | 0.603631 | 2.04E-54 |
| KLHL24 | SOS1-IT1 | 0.603753 | 1.92E-54 |
| MAPK8 | AC022400.5 | 0.603784 | 1.89E-54 |
| TSC1 | AC004148.2 | 0.603847 | 1.83E-54 |
| ATG12 | AC006017.1 | 0.603904 | 1.78E-54 |
| ATG2B | AP001486.2 | 0.603927 | 1.76E-54 |
| PTEN | AC092953.2 | 0.604017 | 1.68E-54 |
| ATG16L2 | AP001107.4 | 0.604145 | 1.57E-54 |
| ATG16L2 | AC024075.2 | 0.604374 | 1.40E-54 |
| WDFY3 | AC020978.3 | 0.604437 | 1.36E-54 |
| FOXO1 | AL136115.2 | 0.604442 | 1.35E-54 |
| WDFY3 | AF131215.5 | 0.604535 | 1.29E-54 |
| WDFY3 | AC007938.3 | 0.604685 | 1.19E-54 |
| BIRC6 | LINC00861 | 0.604795 | 1.13E-54 |
| BIRC6 | AL122035.1 | 0.604895 | 1.07E-54 |
| MTOR | AC073046.1 | 0.604998 | 1.02E-54 |
| WDFY3 | AL136531.1 | 0.605007 | 1.01E-54 |
| PTEN | FMR1-IT1 | 0.605016 | 1.01E-54 |
| ATG16L2 | STAG3L5P-PVRIG2P-PILRB | 0.605023 | 1.01E-54 |
| KLHL24 | N4BP2L2-IT2 | 0.605059 | 9.87E-55 |
| FOXO1 | AC005920.2 | 0.605125 | 9.54E-55 |
| RB1CC1 | LINC00630 | 0.605226 | 9.07E-55 |
| PTEN | SP2-AS1 | 0.605404 | 8.28E-55 |
| PTEN | AC005479.2 | 0.605525 | 7.78E-55 |
| BIRC6 | SNHG14 | 0.605614 | 7.43E-55 |
| ATG4B | AC008735.2 | 0.605724 | 7.03E-55 |
| WDFY3 | AL354696.1 | 0.605934 | 6.31E-55 |
| CFLAR | LINC-PINT | 0.605982 | 6.16E-55 |
| TSC1 | AC073655.2 | 0.606265 | 5.33E-55 |
| PTEN | AL391001.1 | 0.60628 | 5.29E-55 |
| RB1CC1 | AF117829.1 | 0.606312 | 5.20E-55 |
| ATG2B | AL139011.1 | 0.606443 | 4.86E-55 |
| FOXO1 | AL133445.2 | 0.606489 | 4.75E-55 |
| WDFY3 | AL139041.1 | 0.606508 | 4.70E-55 |
| BIRC6 | AL359715.3 | 0.606587 | 4.52E-55 |
| ATG2B | AC112722.1 | 0.606659 | 4.35E-55 |
| BIRC6 | AC011468.5 | 0.606712 | 4.24E-55 |
| BIRC6 | AC027097.1 | 0.606856 | 3.93E-55 |
| ATG12 | AC090739.1 | 0.606985 | 3.68E-55 |
| ATG12 | AC005021.1 | 0.607057 | 3.55E-55 |
| KLHL24 | AC022150.4 | 0.6073 | 3.13E-55 |
| KLHL24 | AC234775.3 | 0.607313 | 3.11E-55 |
| ATG2B | AC009054.2 | 0.607314 | 3.11E-55 |
| ATG16L2 | AC007292.1 | 0.607376 | 3.01E-55 |
| ATG2B | AL606834.1 | 0.607387 | 2.99E-55 |
| ATG12 | MIR155HG | 0.607428 | 2.93E-55 |
| ATG12 | AC007014.2 | 0.607736 | 2.50E-55 |
| WDFY3 | AC092953.2 | 0.607791 | 2.43E-55 |
| WDFY3 | LINC01389 | 0.607889 | 2.31E-55 |
| ATG2B | AL513327.1 | 0.608072 | 2.10E-55 |
| ATG12 | AC010761.3 | 0.608363 | 1.81E-55 |
| PTEN | AL021707.8 | 0.608531 | 1.66E-55 |
| BIRC6 | AP001628.1 | 0.608727 | 1.50E-55 |
| ATG2B | AC018682.1 | 0.608766 | 1.47E-55 |
| ATG2B | GAS8-AS1 | 0.608796 | 1.45E-55 |
| PTEN | ABALON | 0.608884 | 1.38E-55 |
| ATG12 | SDCBP2-AS1 | 0.609156 | 1.20E-55 |
| TSC1 | AC005519.1 | 0.609245 | 1.15E-55 |
| ATG12 | AP001160.4 | 0.609271 | 1.13E-55 |
| PTEN | EGOT | 0.609341 | 1.09E-55 |
| IL24 | LINC02154 | 0.609375 | 1.07E-55 |
| KLHL24 | AC253536.3 | 0.609376 | 1.07E-55 |
| ATG2B | AC093799.1 | 0.609425 | 1.05E-55 |
| ATG2B | KDM4A-AS1 | 0.609443 | 1.04E-55 |
| RB1CC1 | AL133243.2 | 0.609514 | 9.98E-56 |
| ATG2B | AL031717.1 | 0.609567 | 9.71E-56 |
| PIK3R4 | AC073046.1 | 0.609575 | 9.67E-56 |
| RB1CC1 | SOS1-IT1 | 0.609734 | 8.90E-56 |
| CFLAR | AC007566.1 | 0.609982 | 7.82E-56 |
| BIRC6 | LINC01578 | 0.609998 | 7.76E-56 |
| FOXO1 | AC007216.3 | 0.610054 | 7.54E-56 |
| KLHL24 | GAS5-AS1 | 0.610063 | 7.50E-56 |
| WDFY3 | AC004596.1 | 0.610075 | 7.46E-56 |
| WDFY3 | AC087481.3 | 0.610127 | 7.26E-56 |
| KLHL24 | AC090425.2 | 0.610207 | 6.96E-56 |
| PTEN | MAST4-AS1 | 0.610296 | 6.65E-56 |
| PTEN | AL138921.2 | 0.61047 | 6.07E-56 |
| PTEN | AC005261.1 | 0.610482 | 6.03E-56 |
| CFLAR | ERVK13-1 | 0.610828 | 5.04E-56 |
| WDFY3 | AC022306.2 | 0.610846 | 4.99E-56 |
| ATG12 | AL353804.2 | 0.610965 | 4.69E-56 |
| ATG4B | LINC01089 | 0.611007 | 4.59E-56 |
| TSC1 | AC024075.1 | 0.611128 | 4.31E-56 |
| TSC1 | AC087481.3 | 0.611173 | 4.21E-56 |
| CFLAR | AC008115.3 | 0.611177 | 4.20E-56 |
| PTEN | LINC00861 | 0.611234 | 4.08E-56 |
| CFLAR | AL157394.1 | 0.611239 | 4.07E-56 |
| ATG16L2 | AC003070.1 | 0.611275 | 3.99E-56 |
| ATG2B | AC012467.1 | 0.611291 | 3.96E-56 |
| PIK3C3 | AC108449.2 | 0.611371 | 3.80E-56 |
| BIRC6 | PSMA3-AS1 | 0.611497 | 3.56E-56 |
| ATG16L2 | AL365330.1 | 0.611569 | 3.42E-56 |
| CFLAR | C5orf56 | 0.611614 | 3.34E-56 |
| WDFY3 | NUTM2A-AS1 | 0.611633 | 3.31E-56 |
| BIRC6 | NFYC-AS1 | 0.611674 | 3.24E-56 |
| BIRC6 | AL121839.2 | 0.611788 | 3.05E-56 |
| ATG2B | FLNB-AS1 | 0.611933 | 2.83E-56 |
| CCR2 | CARD8-AS1 | 0.612012 | 2.72E-56 |
| TSC1 | AC108010.1 | 0.612013 | 2.71E-56 |
| TSC1 | AC067852.3 | 0.612172 | 2.50E-56 |
| BIRC6 | AC138956.2 | 0.612489 | 2.12E-56 |
| ATG12 | DLEU1 | 0.61263 | 1.96E-56 |
| ATG12 | AF178030.1 | 0.612696 | 1.90E-56 |
| FOXO1 | ANKRD44-IT1 | 0.612729 | 1.87E-56 |
| CFLAR | AC022400.5 | 0.612802 | 1.80E-56 |
| PTEN | AC007552.2 | 0.613103 | 1.53E-56 |
| FOXO1 | AL022067.1 | 0.61313 | 1.51E-56 |
| PTEN | AGAP1-IT1 | 0.613147 | 1.50E-56 |
| EIF2AK2 | AL133243.2 | 0.613316 | 1.37E-56 |
| ATG12 | AC124312.4 | 0.613333 | 1.36E-56 |
| ATG12 | AC138393.3 | 0.613351 | 1.35E-56 |
| WDFY3 | MAGI2-AS3 | 0.613394 | 1.32E-56 |
| TSC1 | ZNF32-AS2 | 0.613665 | 1.14E-56 |
| BIRC6 | MBNL1-AS1 | 0.613705 | 1.12E-56 |
| ATG2B | DLEU1 | 0.613721 | 1.11E-56 |
| ATG16L2 | AC090589.3 | 0.613724 | 1.11E-56 |
| PTEN | MIR181A2HG | 0.613795 | 1.07E-56 |
| BIRC6 | AC007319.1 | 0.6138 | 1.06E-56 |
| BIRC6 | INE1 | 0.613928 | 9.94E-57 |
| ATG16L2 | AC004148.2 | 0.614025 | 9.44E-57 |
| ATG12 | AC104695.3 | 0.614266 | 8.31E-57 |
| CFLAR | AL021578.1 | 0.614365 | 7.89E-57 |
| ATG2B | ATP1B3-AS1 | 0.614418 | 7.67E-57 |
| PTEN | AP005899.1 | 0.614506 | 7.33E-57 |
| PTEN | AL512791.1 | 0.614544 | 7.18E-57 |
| WDFY3 | AC009090.1 | 0.614592 | 7.00E-57 |
| KLHL24 | AC048344.4 | 0.614759 | 6.41E-57 |
| ATG2B | AC078778.1 | 0.614765 | 6.39E-57 |
| WDFY3 | AC008669.1 | 0.61481 | 6.24E-57 |
| ATG12 | AC026355.1 | 0.614867 | 6.05E-57 |
| NLRC4 | AL034397.3 | 0.614899 | 5.95E-57 |
| WDFY3 | AC048341.2 | 0.615109 | 5.32E-57 |
| ATG12 | AC012181.1 | 0.61514 | 5.24E-57 |
| ATG2B | AC107068.1 | 0.615206 | 5.06E-57 |
| ATG12 | AL049840.1 | 0.615282 | 4.86E-57 |
| PTEN | AC004477.3 | 0.615311 | 4.79E-57 |
| ATG12 | AL049552.1 | 0.615355 | 4.68E-57 |
| ATG2B | AC009120.3 | 0.615371 | 4.64E-57 |
| WDFY3 | AL391001.1 | 0.615453 | 4.44E-57 |
| ATG12 | AC022973.3 | 0.615515 | 4.30E-57 |
| BIRC6 | ZKSCAN2-DT | 0.615583 | 4.14E-57 |
| ATG12 | AP001429.1 | 0.615629 | 4.04E-57 |
| KLHL24 | HCG18 | 0.615647 | 4.01E-57 |
| ATG2B | TMEM161B-AS1 | 0.615686 | 3.92E-57 |
| ATG7 | LINC01094 | 0.615688 | 3.92E-57 |
| ATG2B | AP001432.1 | 0.615729 | 3.84E-57 |
| WDFY3 | AL031716.1 | 0.615771 | 3.75E-57 |
| BIRC6 | AC015871.3 | 0.615948 | 3.41E-57 |
| PTEN | ATP1A1-AS1 | 0.616007 | 3.31E-57 |
| PTEN | STARD4-AS1 | 0.616041 | 3.25E-57 |
| FOXO1 | AL031716.1 | 0.616058 | 3.22E-57 |
| ATG12 | LINC00513 | 0.616189 | 3.01E-57 |
| PTEN | OCIAD1-AS1 | 0.616246 | 2.92E-57 |
| ATG12 | Z68871.1 | 0.616292 | 2.84E-57 |
| ATG12 | AL163051.2 | 0.616328 | 2.79E-57 |
| ATG16L2 | AC069281.2 | 0.61643 | 2.64E-57 |
| CCR2 | LINC00996 | 0.616452 | 2.61E-57 |
| TSC1 | RUSC1-AS1 | 0.616594 | 2.42E-57 |
| BIRC6 | AL359915.2 | 0.61704 | 1.91E-57 |
| ATG12 | PSPC1-AS2 | 0.617184 | 1.77E-57 |
| WDFY3 | ZNF460-AS1 | 0.617283 | 1.68E-57 |
| BIRC6 | ACTA2-AS1 | 0.617293 | 1.67E-57 |
| BIRC6 | MIR222HG | 0.617348 | 1.62E-57 |
| BIRC6 | STARD4-AS1 | 0.617396 | 1.58E-57 |
| PTEN | AC009318.2 | 0.61746 | 1.53E-57 |
| MAPK8 | AP005899.1 | 0.61774 | 1.32E-57 |
| BIRC6 | AC025171.3 | 0.617781 | 1.29E-57 |
| ATG2B | AC002128.2 | 0.618136 | 1.06E-57 |
| ATG2B | AC096741.1 | 0.618142 | 1.06E-57 |
| PTEN | GARS-DT | 0.618187 | 1.04E-57 |
| PTEN | AL355488.1 | 0.618243 | 1.01E-57 |
| BIRC6 | AC005046.1 | 0.61835 | 9.50E-58 |
| PTEN | AC020978.3 | 0.618451 | 8.99E-58 |
| WDFY3 | AC025171.4 | 0.618458 | 8.96E-58 |
| ATG12 | AC037487.2 | 0.618528 | 8.63E-58 |
| ATG2B | C1RL-AS1 | 0.618561 | 8.48E-58 |
| BIRC6 | NUTM2A-AS1 | 0.618589 | 8.35E-58 |
| ATG12 | AC093788.1 | 0.618609 | 8.26E-58 |
| MAPK8 | SOS1-IT1 | 0.618662 | 8.03E-58 |
| ATG2B | AP003170.3 | 0.618836 | 7.32E-58 |
| ATG2B | PPP3CB-AS1 | 0.618848 | 7.27E-58 |
| EIF2AK2 | SOS1-IT1 | 0.618943 | 6.91E-58 |
| BIRC6 | ATP1A1-AS1 | 0.618947 | 6.89E-58 |
| MAPK8 | AL731566.1 | 0.619039 | 6.56E-58 |
| ATG12 | AC124312.5 | 0.619072 | 6.45E-58 |
| PTEN | AL122035.1 | 0.619085 | 6.40E-58 |
| PTEN | AC105389.2 | 0.619097 | 6.36E-58 |
| WDFY3 | AC079684.1 | 0.619107 | 6.33E-58 |
| ATG2B | AC018690.1 | 0.619182 | 6.08E-58 |
| ATG12 | AC009032.1 | 0.619236 | 5.90E-58 |
| ATG12 | AC008966.2 | 0.619515 | 5.08E-58 |
| ATG2B | AF178030.1 | 0.619597 | 4.86E-58 |
| BIRC6 | AC053527.1 | 0.61965 | 4.73E-58 |
| FOXO1 | AC087286.4 | 0.619804 | 4.35E-58 |
| WDFY3 | MUC20-OT1 | 0.619875 | 4.19E-58 |
| WDFY3 | AC009948.1 | 0.619939 | 4.05E-58 |
| ATG12 | RRN3P2 | 0.619986 | 3.95E-58 |
| ATG4B | STAG3L5P-PVRIG2P-PILRB | 0.620009 | 3.90E-58 |
| ATG2B | AC013403.2 | 0.620244 | 3.43E-58 |
| ATG2B | AC024933.1 | 0.620308 | 3.32E-58 |
| WDFY3 | UGDH-AS1 | 0.620331 | 3.28E-58 |
| BIRC6 | AC138207.4 | 0.620367 | 3.21E-58 |
| BIRC6 | MIR181A2HG | 0.620459 | 3.06E-58 |
| ATG2B | AC090579.1 | 0.620547 | 2.92E-58 |
| ATG2B | AC025171.3 | 0.620725 | 2.65E-58 |
| CFLAR | FAM13A-AS1 | 0.620757 | 2.60E-58 |
| ATG2B | AC127024.5 | 0.620834 | 2.50E-58 |
| ATG12 | AL109614.1 | 0.620883 | 2.43E-58 |
| CAPN10 | AL022328.2 | 0.621105 | 2.16E-58 |
| BIRC6 | AL512791.1 | 0.621237 | 2.01E-58 |
| WDFY3 | AC138207.4 | 0.621332 | 1.91E-58 |
| ATG2B | EP300-AS1 | 0.621444 | 1.80E-58 |
| PTEN | LINC01578 | 0.621448 | 1.79E-58 |
| WDFY3 | AC010245.2 | 0.621468 | 1.77E-58 |
| ATG2B | AC015871.3 | 0.621546 | 1.70E-58 |
| BIRC6 | AL606834.2 | 0.621598 | 1.65E-58 |
| FOS | AC025259.3 | 0.621678 | 1.58E-58 |
| PTEN | AC083949.1 | 0.621757 | 1.52E-58 |
| WDFY3 | AC133644.2 | 0.621861 | 1.43E-58 |
| ATG2B | AL133342.1 | 0.621861 | 1.43E-58 |
| ATG12 | FAM13A-AS1 | 0.622004 | 1.33E-58 |
| PTEN | PCBP1-AS1 | 0.622045 | 1.30E-58 |
| ATG16L2 | AC004771.1 | 0.622046 | 1.30E-58 |
| MAPK8 | AC108449.2 | 0.622059 | 1.29E-58 |
| FOXO1 | AP001429.1 | 0.622074 | 1.28E-58 |
| PTEN | AC022211.1 | 0.622152 | 1.22E-58 |
| BIRC6 | AL117336.2 | 0.622269 | 1.15E-58 |
| BIRC6 | AC018809.2 | 0.622474 | 1.03E-58 |
| ATG12 | AC096741.1 | 0.622516 | 1.00E-58 |
| WDFY3 | LINC00861 | 0.622635 | 9.41E-59 |
| ATG12 | AP002336.2 | 0.622656 | 9.30E-59 |
| BIRC6 | AL391001.1 | 0.622683 | 9.17E-59 |
| ATG7 | AC083949.1 | 0.622847 | 8.39E-59 |
| ATG12 | AC074032.1 | 0.622854 | 8.36E-59 |
| ATG2B | A2M-AS1 | 0.62296 | 7.89E-59 |
| BIRC6 | AC007991.2 | 0.623105 | 7.29E-59 |
| ATG12 | AC130895.1 | 0.62315 | 7.11E-59 |
| WDFY3 | AC012181.2 | 0.62335 | 6.38E-59 |
| ATG12 | AC005838.2 | 0.623433 | 6.09E-59 |
| WDFY3 | AC007991.2 | 0.623456 | 6.02E-59 |
| ATG12 | AL157402.2 | 0.623471 | 5.97E-59 |
| BIRC6 | AC008035.1 | 0.623488 | 5.92E-59 |
| ATG12 | AL162724.1 | 0.623646 | 5.43E-59 |
| ATG2B | AL355488.1 | 0.623805 | 4.98E-59 |
| ATG12 | AC124283.3 | 0.624021 | 4.42E-59 |
| ATG12 | AC090181.2 | 0.624071 | 4.30E-59 |
| WDFY3 | AL606834.2 | 0.624112 | 4.21E-59 |
| ATG2B | AC027097.2 | 0.624167 | 4.08E-59 |
| FOXO1 | AC114760.2 | 0.624233 | 3.94E-59 |
| PTEN | AC005034.5 | 0.624506 | 3.39E-59 |
| BIRC6 | AL450263.1 | 0.624561 | 3.29E-59 |
| ATG2B | AP000786.1 | 0.624872 | 2.78E-59 |
| TSC1 | AL157392.3 | 0.624966 | 2.64E-59 |
| PTEN | AC141002.1 | 0.625041 | 2.53E-59 |
| TSC1 | SH3BP5-AS1 | 0.625042 | 2.53E-59 |
| BIRC6 | AC093484.4 | 0.62505 | 2.52E-59 |
| CASP1 | USP30-AS1 | 0.625313 | 2.18E-59 |
| BIRC6 | OCIAD1-AS1 | 0.625335 | 2.15E-59 |
| ATG2B | AC002044.1 | 0.625372 | 2.11E-59 |
| ATG12 | AC020913.3 | 0.625427 | 2.05E-59 |
| BIRC6 | AC073534.1 | 0.625437 | 2.04E-59 |
| ATG2B | AC002128.1 | 0.625514 | 1.95E-59 |
| PTEN | AC234772.2 | 0.625545 | 1.92E-59 |
| BIRC6 | AC004596.1 | 0.625567 | 1.90E-59 |
| ATG12 | AC097376.2 | 0.625601 | 1.86E-59 |
| MAPK8 | HCG18 | 0.625606 | 1.86E-59 |
| BIRC6 | AL442125.2 | 0.62562 | 1.84E-59 |
| MAPK8 | NUTM2B-AS1 | 0.625721 | 1.74E-59 |
| TSC1 | AC008735.4 | 0.625742 | 1.72E-59 |
[truncated: 70,129 more chars]
