# Supplementary figures and images for: Long non-coding RNA LINC01559 exerts oncogenic role via enhancing autophagy in lung adenocarcinoma
Source: Cancer Cell Int. 2021 Nov 25;21:624. doi: 10.1186/s12935-021-02338-4 (PMC8614059; doi:10.1186/s12935-021-02338-4)

Figure S1. Kaplan-Meier survival curves of 9 single-lncRNA.


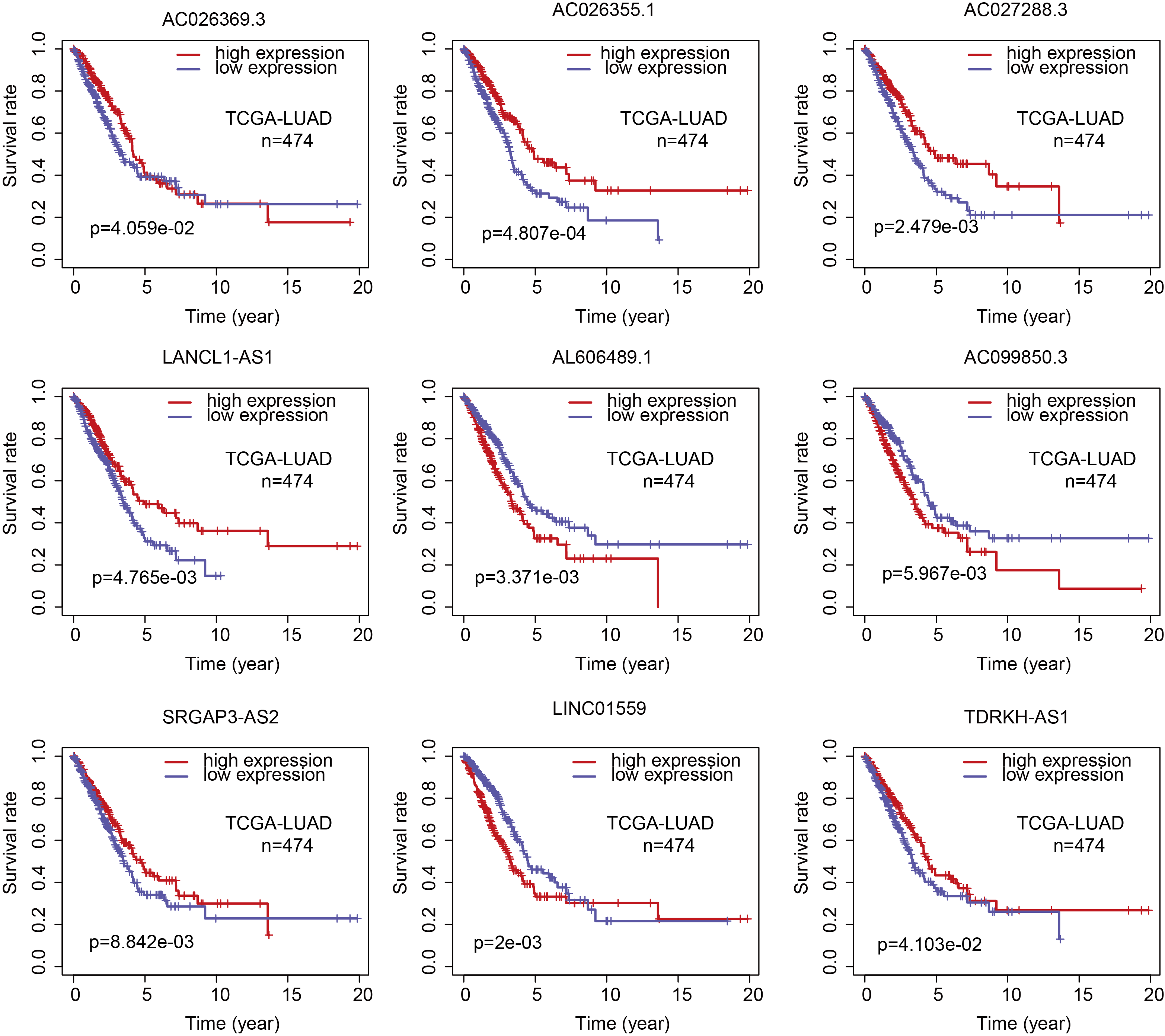

Supplement: Supplementary file 5 — Additional file 5. Figure S1. [file 12935_2021_2338_MOESM5_ESM.docx]

Figure S4. Correlation expression of LINC01559, RAB11A and hsa-miR-1343-3p.


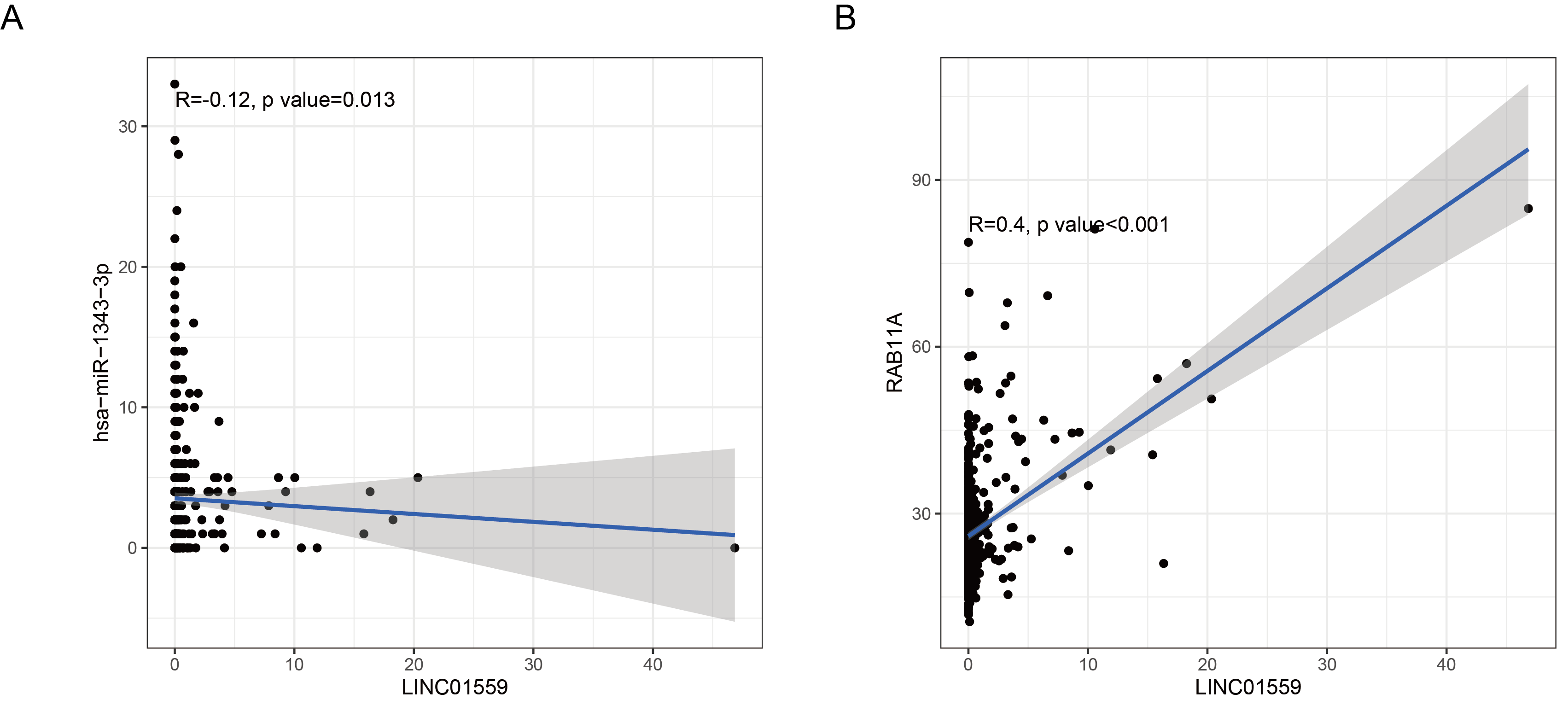

Supplement: Supplementary file 8 — Additional file 8. Figure S4. [file 12935_2021_2338_MOESM8_ESM.docx]
