# Supplementary material for: Long non-coding RNA LINC01559 exerts oncogenic role via enhancing autophagy in lung adenocarcinoma
Source: Cancer Cell Int. 2021 Nov 25;21:624. doi: 10.1186/s12935-021-02338-4 (PMC8614059; doi:10.1186/s12935-021-02338-4)

Figure S2. (A) Differential pathway enrichment between high- and low-risk groups analyzed by GSEA and GSVA.


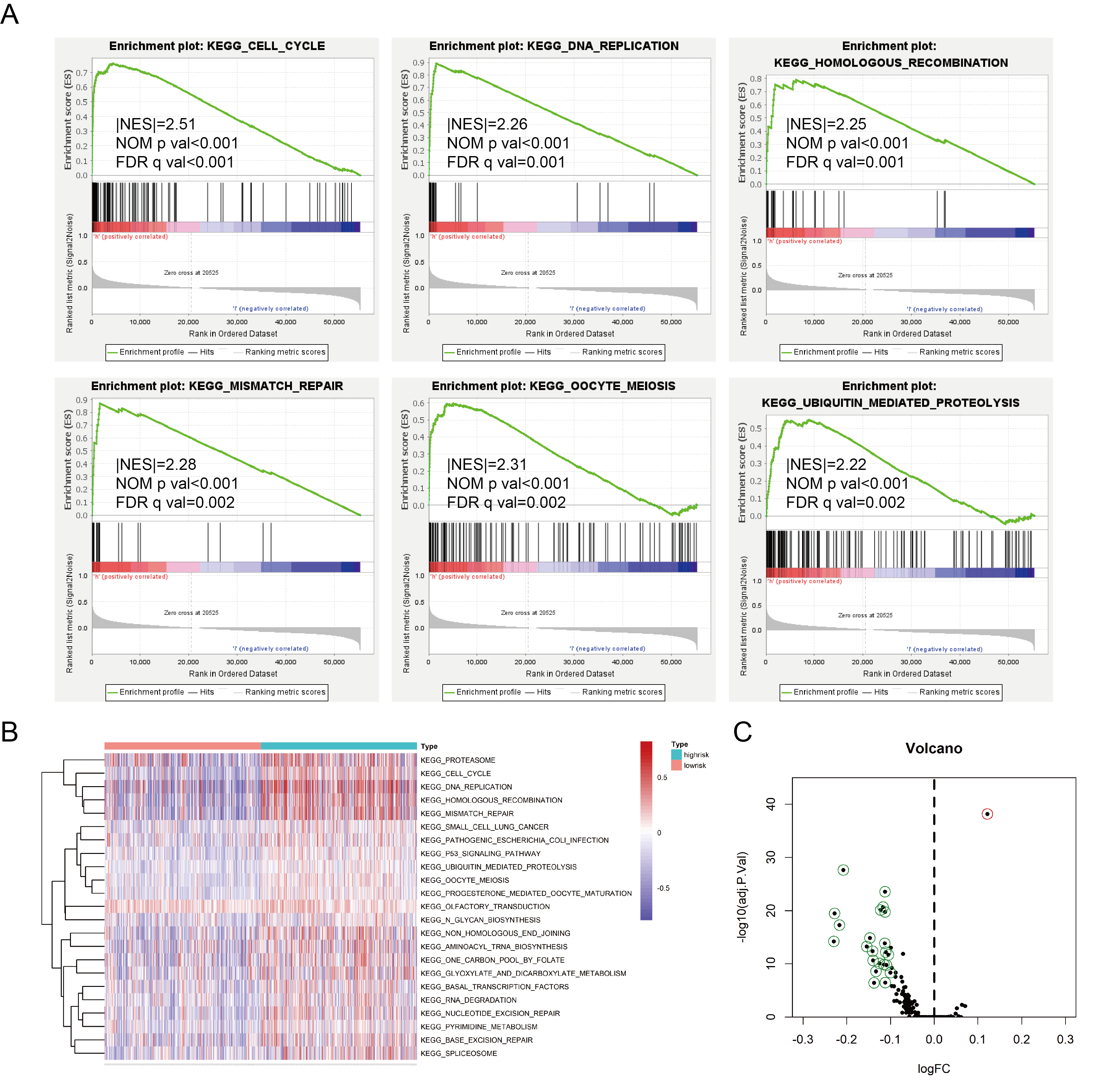

Supplement: Supplementary file 6 — Additional file 6. Figure S2. [file 12935_2021_2338_MOESM6_ESM.docx]
