# Supplementary material for: Long non-coding RNA LINC01559 exerts oncogenic role via enhancing autophagy in lung adenocarcinoma
Source: Cancer Cell Int. 2021 Nov 25;21:624. doi: 10.1186/s12935-021-02338-4 (PMC8614059; doi:10.1186/s12935-021-02338-4)

Figure S3. LINC01559 could inhibit the autophagy promotion function of rapamycin to A549 cell line (The dose of rapamycin was 50nM).


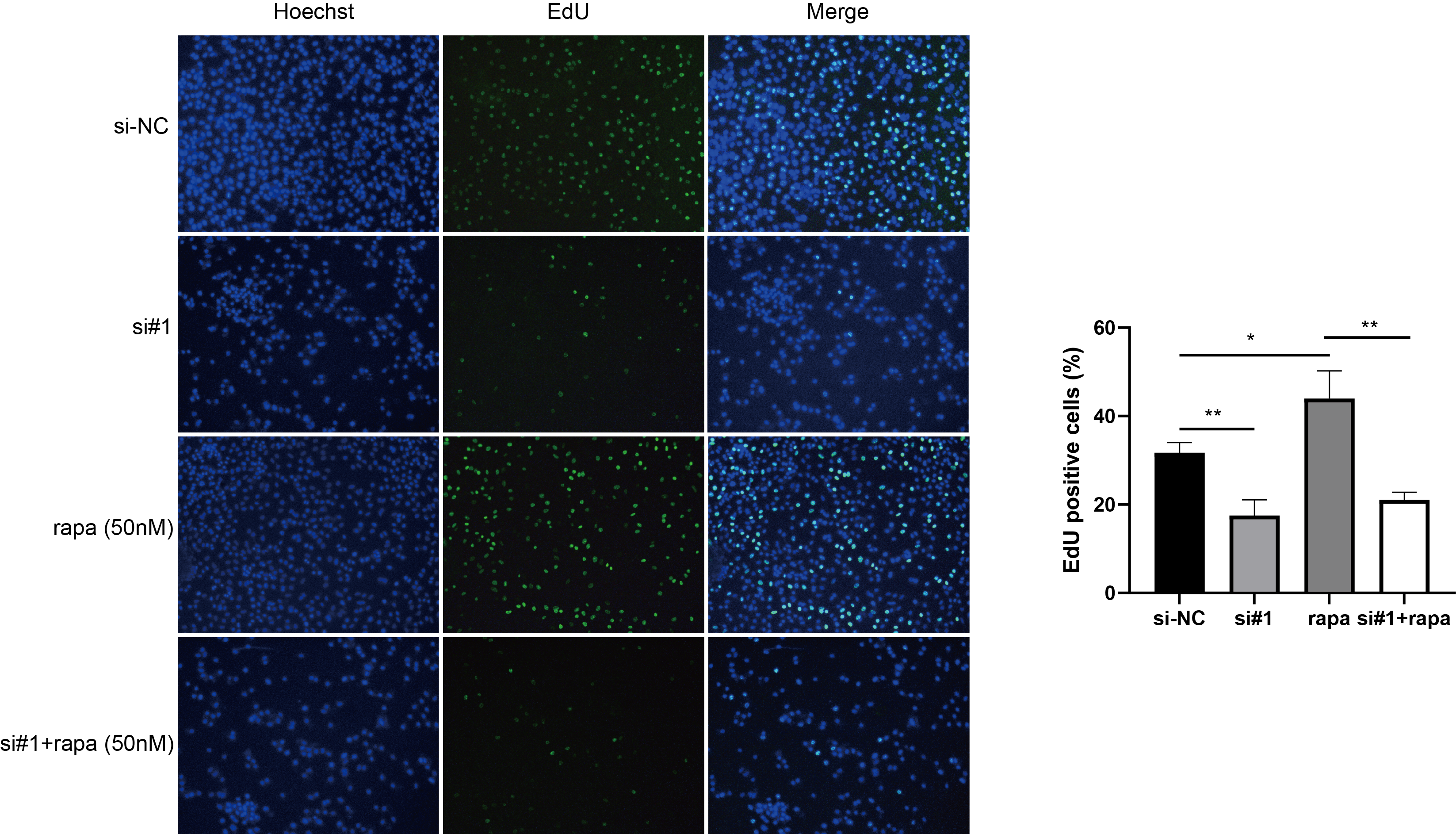

Supplement: Supplementary file 7 — Additional file 7. Figure S3. [file 12935_2021_2338_MOESM7_ESM.docx]
